# Bicyclic Phenyl–Ethynyl Architectures: Synthesis of a 1,4‐Bis(phenylbuta‐1,3‐diyn‐1‐yl) Benzene Banister

**DOI:** 10.1002/chem.202005207

**Published:** 2021-03-03

**Authors:** Linda Maria Bannwart, Thomas Müntener, Michel Rickhaus, Lukas Jundt, Daniel Häussinger, Marcel Mayor

**Affiliations:** ^1^ Department of Chemistry University of Basel St. Johanns-Ring 19 4056 Basel Switzerland; ^2^ Biozentrum University of Basel Klingelbergstrasse 70 4056 Basel Switzerland; ^3^ Department of Chemistry University of Zurich Winterthurerstrasse 190 8057 Zurich Switzerland; ^4^ Institute for Nanotechnology (INT) Karlsruhe Institute of Technology (KIT) P. O. Box 3640 76021 Karlsruhe Germany; ^5^ Lehn Institute of Functional Materials (LIFM) School of Chemistry Sun Yat-Sen University (SYSU) Guangzhou 510275 P. R. China

**Keywords:** chirality, cross-coupling, macrocycles, oligomers, tropos isomer

## Abstract

The novel diacetylene bridged terphenylic macrocycle **1** is presented and discussed in the context of rotationally restricted “Geländer” oligomers. The 1,4‐bis(phenylbuta‐1,3‐diyn‐1‐yl) benzene bridge of diacetylene **1** is significantly longer than its terphenyl backbone, forcing the bridge to bend around the central pylon. The synthesis of molecule **1** is based to a large extent on acetylene scaffolding strategies, profiting from orthogonal alkyne protection groups to close both macrocyclic subunits by oxidative acetylene coupling sequentially. The spatial arrangement and the dynamic enantiomerization process of the bicyclic target structure **1** are analyzed. In‐depth NMR investigations not only reveal an unexpected spatial arrangement with both oligomer strands bent alongside the backbone, but also display the limited stability of the model compound in the presence of molecular oxygen.

## Graphical abstract

**The conjugated** 1,4‐bis(phenylbuta‐1,3‐diyn‐1‐yl) benzene bridge of our terphenylic macrocycle is significantly longer than the backbone it rotates around, thereby forcing itself to bend along the central pylon. The synthesis of the macromolecule is largely based on orthogonal acetylene scaffolding strategies, allowing to sequentially finalize both macrocyclic subunits by oxidative acetylene coupling. The spatial arrangement and the dynamic enantiomerization process of the final, bicyclic compound are analyzed and discussed.

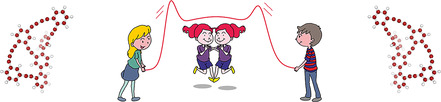

## Introduction

The fascination for conjugated macrocycles stems not only from the combination of structural beauty with well‐defined shape and size, but also from their intrinsic physical (optical, electronic) properties.[^1^, ^2^, ^3^] The synthetic challenges pertaining to shape‐persistent macrocycles such as cyclynes and arenecyclynes also make them attractive from a methods development viewpoint.[^4^, ^5^] A particularly beautiful example is the entirely sp‐hybridized macrocyclic carbon allotrope reported by Anderson and co‐workers recently.^[6]^ While most macrocycles are formally achiral (without a chiral center), their spatial arrangement may induce topological chirality, with axial chirality as a prominent example related to helical structures.[^7^, ^8^, ^9^, ^10^, ^11^]

**Figure 1:**
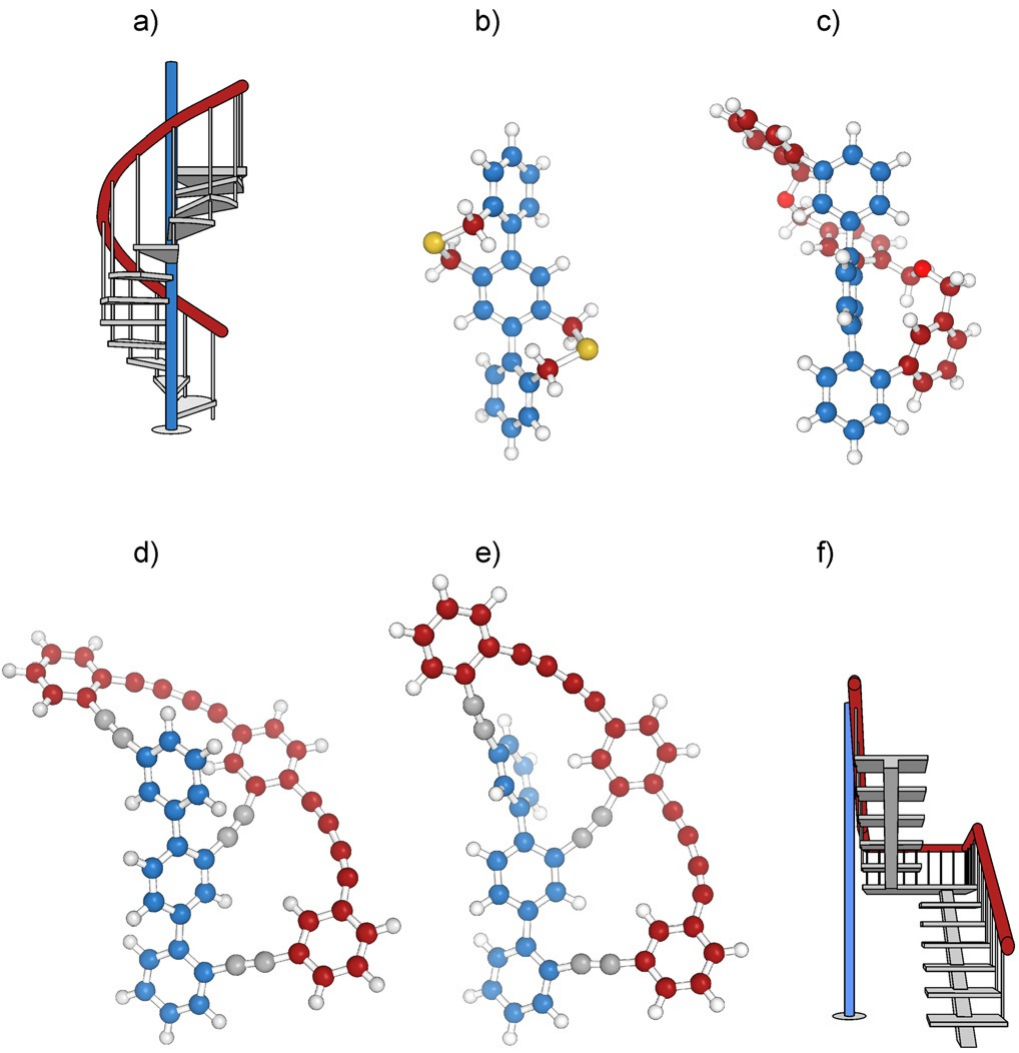
The helical chiral “Geländer” molecules discussed, together with the types of staircases inspiring their design. The structures representing the central axes are blue, and the ones representing the banisters are brick red. a) Spiral staircase. b) The pioneering “Geländer” oligomer described by Vögtle and co‐workers.^[12]^ c) Our initial “Geländer” design avoiding achiral *meso*‐forms.[^14^, ^15^, ^16^] d) The intended helical arrangement of the 1,4‐bis(phenylbuta‐1,3‐diyn‐1‐yl) benzene oligomer in our reported target structure **1**. e) The observed spatial arrangement of the 1,4‐bis(phenylbuta‐1,3‐diyn‐1‐yl) benzene oligomer of **1** according to NMR analyses, resembling rather the banister of a staircase as sketched in f) with an inserted floor.

In 1998, Vögtle and co‐workers presented the concept of “Geländer” molecules as a new class of axial helical structures.^[12]^ “Geländer” is the German word for banister, explaining the intention of the design concept. As displayed in Figure 1 b, a *para*‐terphenyl structure was complemented with two additional linkers between neighboring phenyl subunits, like adding a banister to a spiral staircase (see Figure 1 a). These two phenyl‐interlinking chains were intended to wrap around the terphenyl axis in helical manner. While conceptually pioneering for atropisomers, the molecular design impedes communicating chiral information between the two biphenyl junctions. Consequently, the optically mute *meso* form is often favored over the desired helical arrangement in these pairs of enantiomers.[^12^, ^13^] Our contribution to improved “Geländer” systems was to develop a ladder‐type oligomer where interlinked biphenyl “rungs” are interlinked by structures of different step‐sizes. As sketched in Figure 1 c, the longer oligomer wrapped around the terphenyl backbone and the central biphenyl “rung” acts as relay communicating the chirality information between both biphenyl junctions of the backbone.[^14^, ^15^]

In these new “Geländer” oligomers (Figure 1 c), surprisingly low racemization barriers were observed, although they still qualify as atropisomers. According to Oki's somewhat arbitrary definition, a half‐life of at least 1000 seconds is required for isomers to be labeled as atropos.^[17]^ Consequently, isomers displaying faster racemization are labeled as tropos.[^18^, ^19^] Another challenge observed in the syntheses/characterizations of these “Geländer” oligomers was the formation of different bicyclic systems with varying ring‐sizes.[^16^, ^20^]

**Scheme 1:**
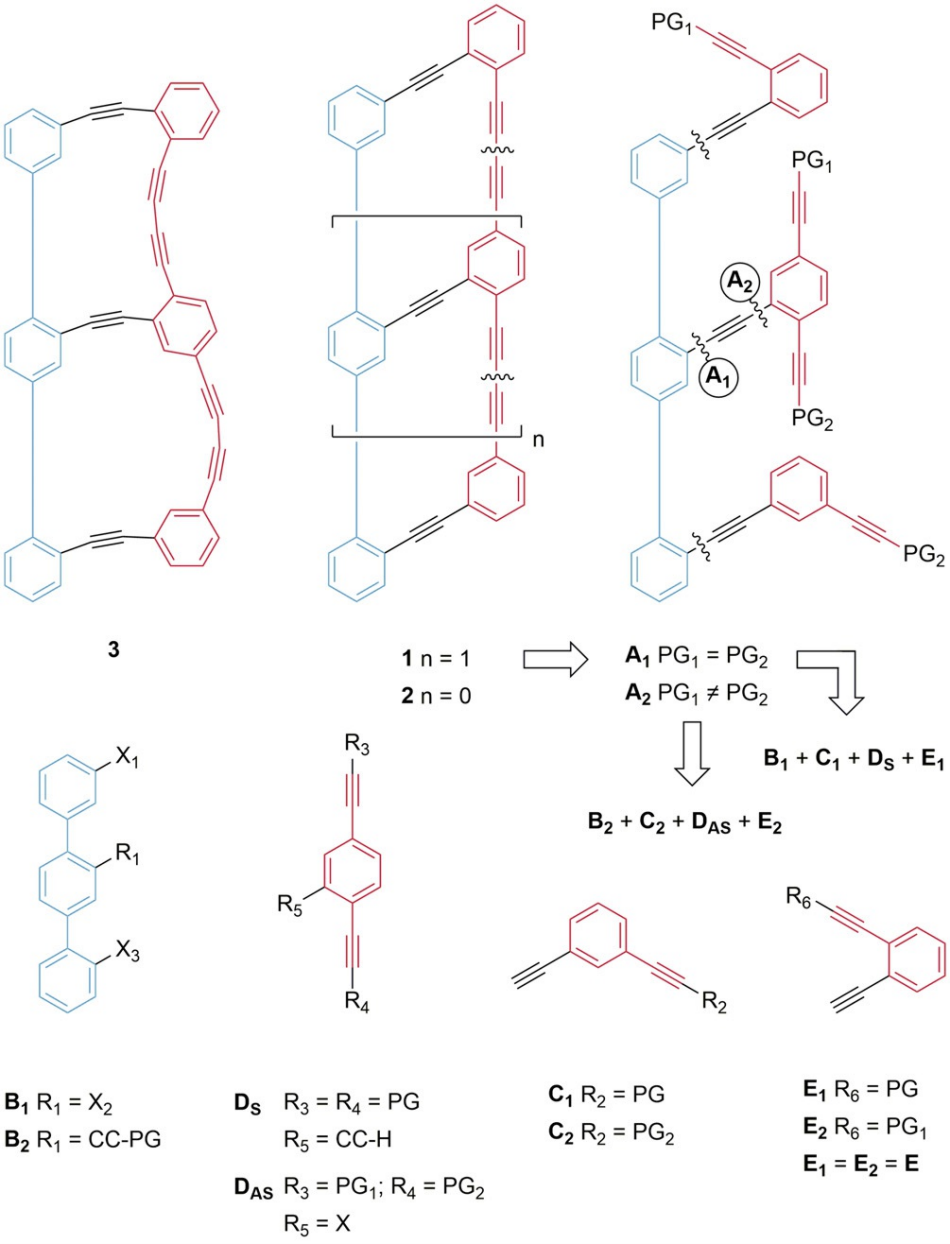
Chemical structures of the series of macrocyclic oligomers **1** and **2**, together with the retrosynthetic considerations for the assembly of **1** from the building blocks **B**‐**E**.

From a materials property perspective, “Geländer” structures with a conjugated banister would be particularly interesting as model compounds with electrons delocalized on a helical subunit. We thus recently reported the synthesis of macrocycle **2** (Scheme 1), the shortest member of a new “Geländer” design with a helical oligo‐*para*‐ phenylene‐ di‐ ethynylenes (OPDE) banister comprising sp‐ and sp^2^‐hybridized carbon atoms exclusively.^[21]^ Even though macrocycle **2** already displayed an enhanced reactivity of the (bent) diacetylene, its room temperature stability suggested the suitability of the molecular design for larger model compounds.

Here the synthesis and structural investigation of the next member of the series, the bicyclic system **1** (Scheme 1) is reported. The investigations allow to draw two main conclusions concerning the molecular design: i) stability of the compounds decreases with increasing number of diacetylenes or the bridge‐length in the oligomer, and ii) the OPDE banister in **1** behaves rather like the banister of a staircase with an inserted floor (Figure 1 f) than a spiral staircase (Figure 1 a)—instead of wrapping helically around the central *para*‐terphenyl axis (Figure 1 d), it bends back on the same side of the axis (Figure 1 e).

## Results and Discussion

### Molecular design

In analogy to our earlier strategy, two oligomers with different lengths were interlinked into a ladder‐type structure, forcing the longer oligomer (OPDE as banister) to wrap around the shorter one (oligophenylenes (OP) as axis). In this work, we intend to increase electron delocalization within the banister through the OPDE oligomer. As discussed previously for monomeric macro‐cycle **2**,^[21]^ strain in the diethynyl subunit should be decreased to an acceptable level by increasing the spacing between both oligomers by an additional ethynyl linker. Using the picture of a ladder again, tolanes were considered as “rungs” instead of biphenyls.

### Retrosynthetic analysis

Retrosynthetic considerations for target “Geländer” oligomer **1** are sketched in Scheme 1. The macrocycles, containing diethynyl subunits, suggest an oxidative acetylene coupling macrocyclization as the final reaction. This has already been used for various macrocycles as a mild and functional group tolerant C−C bond forming reaction.[^22^, ^23^] Interestingly, controlling alkyne protection groups potentially allows for the improved steering of the macrocyclization process. Using **A_1_** as a precursor (bearing four identically protected alkynes), both macrocycles would be closed in the same reaction, most likely resulting in the formation of two bicyclic structures with different ring sizes: apart from target structure **1** (two macrocycles consisting of 18 carbon atoms each), also the undesired, wrongly connected compound **3** with rings comprising 17 and 19 carbon atoms would be expected.

Improved control over the final macrocyclizations should be possible using precursor **A_2_**. Its terminal alkynes are masked pairwise with different protection groups (PGs), enabling the consecutive and controlled closing of both macrocycles and thus the exclusive formation of target compound **1**. In a convergent strategy, precursors **A** can be assembled from building blocks **B**‐**E** by Sonogashira–Hagihara reactions. The backbones **B** can be obtained by Suzuki–Miyaura cross‐coupling reactions. In both of these reactions, the required regioselectivity of the coupling position can be directed with the large difference in reactivity of different halogen substituents.

The syntheses of the two terminal side chains **E** and **C_1_** are reported elsewhere, for the assembly of **2**.^[21]^ Variation of the PG in **C_2_** requires only minor adaptions to this protocol. In the initial strategy geared towards **A_1_**_,_ terphenyl backbone **B_1_** with three different leaving groups was considered. Consequently, a pseudo symmetric central side chain **D_S_** exposing an alkyne group for the coupling with the backbone is required. The two alkynes of **D_S_**, a part of the banister, are masked with the same protection group. The assembly of building block **D_S_** requires two different alkyne protection groups at most and should be easily available from commercial precursors. The challenge of the strategy will be the three differently reactive leaving groups contained in structure **B_1_**. The least reactive one would be substituted last, thus combining its low reactivity with the most sterically demanding reaction, caused by the bulkiness of the previously attached side chains.

This potentially troublesome scenario is avoided in the strategy towards **A_2_**. Here, the terphenyl backbone **B_2_** is functionalized with halogen atoms only at the terminal phenyl rings, while the central phenyl already has the alkyne group attached. The strategy should also ease assembling the central asymmetric middle side chain **D_AS_**, where again only two different protection groups for the alkynes in the target structure's banister are required. In the third position of **D_AS_** (R_5_) a halogen atom enables coupling with the alkyne of **B_2_**. Also, the building blocks required in the assembly of **A_2_** should be available from commercial building blocks in a few steps.

### Synthesis I: Attempts towards A_1_

**Scheme 2:**
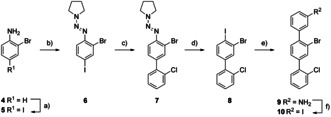
Synthesis of backbone **10**. Reagents and conditions: a) NIS, DMSO, room temp., 18 h, 98 %. b) HCl, NaNO_2_, pyrrolidine, K_2_CO_3_, MeCN, H_2_O, 0 °C→room temp., 2.5 h, 90 %. c) 2‐chlorophenylboronic acid, K_2_CO_3_, (Ph_3_P)_2_PdCl_2_, toluene: EtOH 4:1, 80 °C, 18 h, 87 %. d) I_2_, MeCN, 100 °C, 4 h, 81 %. e) 3‐aminophenylboronic acid monohydrate, K_2_CO_3_, (Ph_3_P)_2_PdCl_2_, toluene: EtOH 4:1, 80 °C, 3.5 h, 89 %. f) *p*TsOH⋅H_2_O, MeCN, NaNO_2_, KI, MeCN, H_2_O, 10 °C→room temp., 16 h, 92 %.

Terphenyl **10** was assembled as backbone building block **B_1_**, containing three different halogen substituents whose reactivities vary (I>Br>Cl) in palladium‐catalyzed cross‐coupling reactions. As displayed in Scheme 2, **10** was obtained in six steps from commercially available 2‐bromoaniline (**4**). Compound **4** was iodinated with *N*‐iodosuccinimide (NIS) in DMSO, providing 2‐bromo‐4‐iodoaniline (**5**) in 98 % yield.^[24]^ The amino group of compound **5** was transformed into triazene **6** in good yields following a protocol reported by Goeminne et al.^[25]^ Triazene **6** acts as a masked and passive leaving group, as it can be converted to an iodine in a later stage of the synthesis. Biphenyl **7** was obtained in a Suzuki–Miyaura reaction of **6** with 2‐chlorophenylboronic acid. The best result was obtained using C_6_H_5_CH_3_/EtOH (4:1) as solvent mixture at 80 °C, in the presence of K_2_CO_3_ and (Ph_3_P)_2_PdCl_2_ as base and catalyst, respectively.[^14^, ^15^] For the transformation of triazene **7** into iodine **8**, a variety of reaction conditions were investigated (e.g. MeI, 120 °C,^[26]^ or I_2_ in hexane, CH_2_Cl_2_ or MeCN^[27]^). The best results (leading to yields up to 99 %) were obtained with MeI at 120 °C in a pressure tube. Unfortunately, these reaction conditions had limited scalability and were efficient on a small scale (<500 mg) exclusively. With larger amounts of starting material, I_2_ in MeCN provided more reliable reaction conditions. For the assembly of terphenyl **9**, reaction conditions similar to the synthesis of biphenyl **7** were applied. The Suzuki–Miyaura cross‐coupling of 3‐aminophenyl boronic acid monohydrate and biphenyl **8** led to compound **9** in reasonable yield (89 %). Finally, applying Sandmeyer‐type reaction conditions to precursor **9** provided terphenyl backbone **10** with three different halogen substituents in a very good 92 % yield.

**Scheme 3:**
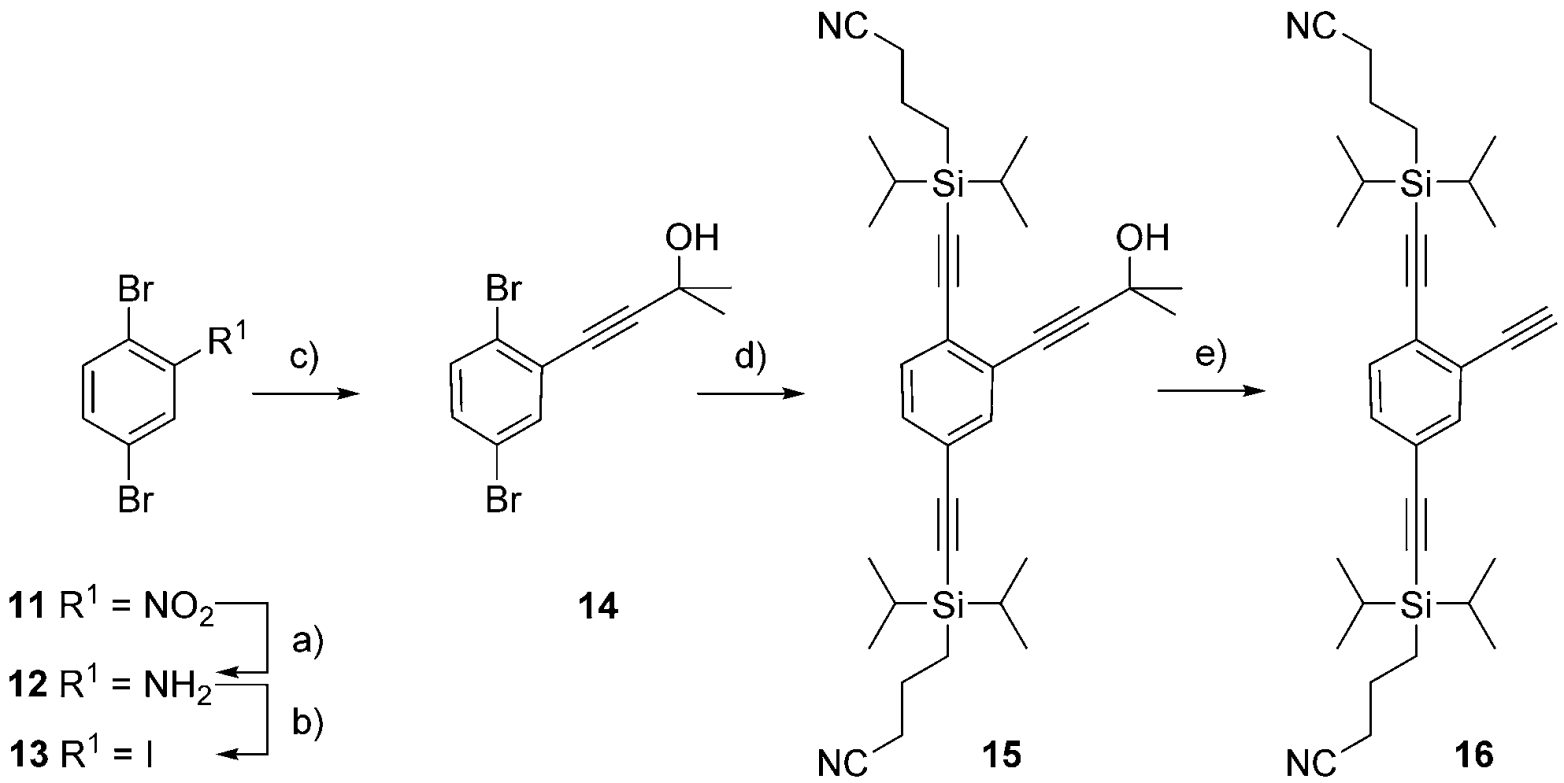
Synthesis of the “pseudo symmetric” central side chain **16**. Reagents and conditions: a) Fe, HCl, EtOH, 50 °C, 4 h, quant. b) *p*TsOH⋅H_2_O, MeCN, NaNO_2_, KI, MeCN, H_2_O, 10 °C→room temp., 1.5 h, 91 %. c) HOP‐CCH, (Ph_3_P)_2_PdCl_2_, CuI, THF: piperidine 3:1, room temp., 1 h, 76 %. d) CPDIPS‐CCH, (Ph_3_P)_2_PdCl_2_, CuI, THF: piperidine 4:1, 60 °C, 1 h, 97 %. e) NaOH, toluene, 120 °C, 1 h, 79 %.

The two terminal side chain building blocks **C_1_** and **E** were both accessible as (3‐cyanopropyl)diisopropylsilyl (CPDIPS) masked acetylenes, as described for compound **2**.^[21]^ This was an ideal protection strategy for the assembly of **A_1_**, due to the group's stability as well as its polarity, facilitating chromatographic isolation of protected derivatives.^[28]^ Thus, the “pseudo symmetric” side chain **16** was synthesized as building block **D_S_** from 1,4‐dibromo‐2‐nitrobenzene (**11**) in five steps (Scheme 3). Amine **12** was obtained in a Béchamp reduction in quantitative yield and was subsequently transformed into 1,4‐dibromo‐2‐iodobenzene (**13**) in a Sandmeyer‐type reaction in excellent yields. Profiting from the superior reactivity of iodine substituents in Pd‐catalyzed cross‐coupling reactions, 2‐hydroxypropyl (HOP) acetylene was introduced in a Sonogashira reaction providing **14** in good yields. Comparable reaction conditions with elevated temperature enabled the subsequent substitution of both bromines by CPDIPS‐acetylenes, affording molecule **15** in excellent 97 % yield. The central side chain building block **16** was obtained as a yellow oil in 79 % yield in a retro‐Favorskii reaction, by refluxing compound **15** with sodium hydroxide in a copper‐free flask.

**Scheme 4:**
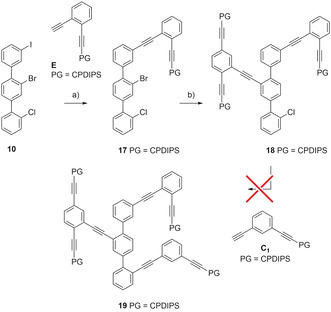
Attempts towards the assembly of **19** as precursor **A_1_**. Reagents and conditions: a) **E**, (Ph_3_P)_2_PdCl_2_, CuI, THF: piperidine 3:1, room temp., 1 h, 93 %. b) **16**, (Ph_3_P)_2_PdCl_2_, CuI, piperidine, 120 °C, 24 h, 40 %.

With all the required building blocks at hand, the assembly of precursor **A_1_** for the macrocyclization was investigated (Scheme 4). First, the iodine in terphenyl **10** was replaced with acetylene **E** using classical Sonogashira cross‐coupling conditions ((Ph_3_P)_2_PdCl_2_, CuI, THF: piperidine 3:1), providing **17** after one hour at room temperature in 93 %. In the subsequent, second Sonogashira reaction, the less reactive bromine in structure **17** required increased reaction temperatures (120 °C) and pure piperidine as a solvent in order to introduce side chain **16**. Despite excessive screening of reaction conditions, (Ph_3_P)_2_PdCl_2_/CuI remained the best performing catalytic system, even though precursor **18** was isolated in only 40 % yield by size‐exclusion chromatography (SEC).

Unfortunately, attempts towards substituting the chlorine substituent of **18** with a phenylacetylene were not successful in our hands. In a variety of explorative model reactions, even conditions optimized for arylchlorines at elevated temperature (150 °C) were not successful and it appeared, that the chlorine of **18** is challenging to be addressed by Sonogashira cross‐coupling conditions.[^29^, ^30^] All attempts to furnish compound **19** resulted either in dehalogenation or decomposition. Considering the only moderate yield in the preceding step, the second approach via structure **A_2_** moved into the focus of interest.

### Synthesis II: Assembly over precursor A_2_

The design of backbone **B_2_** profited from the experiences we collected during the initial approach. In terphenyl **29** not only a masked acetylene is used as a third substituent instead of a halide with limited reactivity. Also, the sterically most difficult *ortho*‐position of the bottom phenyl is bearing an iodine, comparatively facilitating intended coupling reactions.

**Scheme 5:**
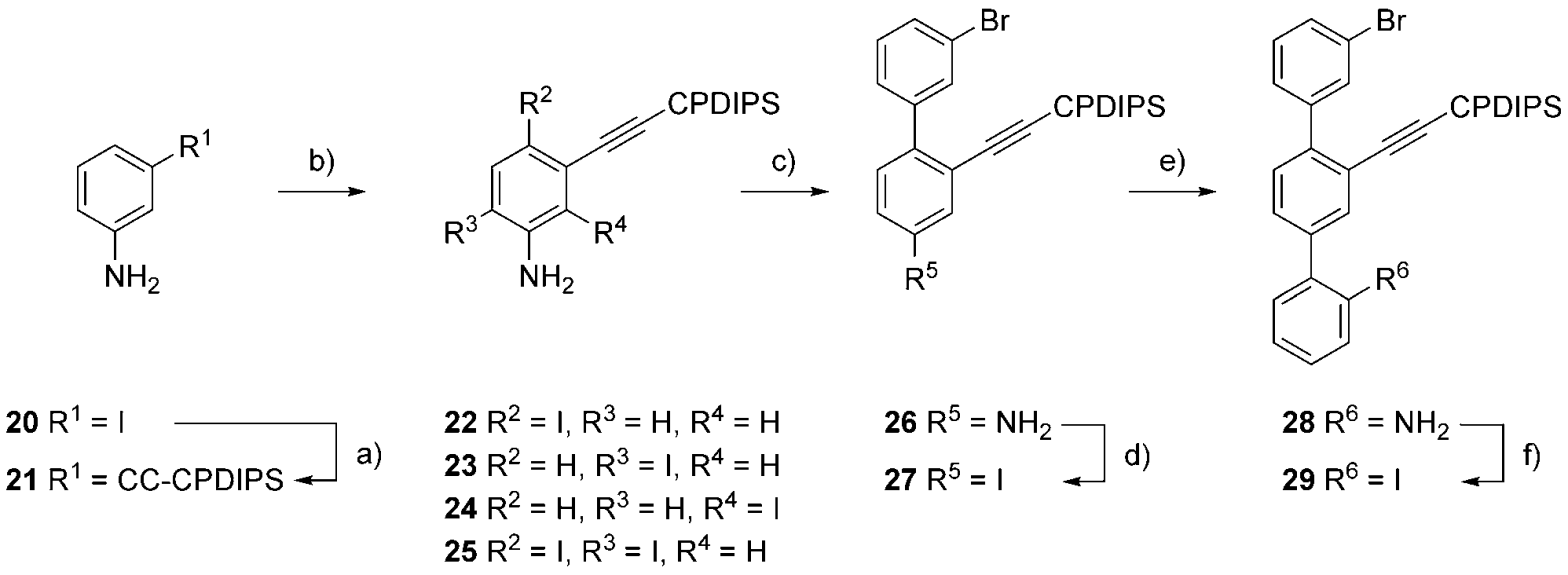
Synthesis of **29** as backbone building block **B_2_**. Reagents and conditions: a) CPDIPS‐acetylene, (Ph_3_P)_2_PdCl_2_, CuI, THF: piperidine 3:1, room temp., 1 h, quant. b) NIS, DMSO, room temp., 1 h, 85 % **22**, 1.9 % **23**, 1.6 % **24** and 4.9 % **25**. c) 3‐bromophenylboronic acid, K_2_CO_3_, (Ph_3_P)_2_PdCl_2_, DME: EtOH: H_2_O 4:1:1, 80 °C, 12 h, 84 %. d) *p*TsOH⋅H_2_O, MeCN, NaNO_2_, KI, MeCN, H_2_O, 10 °C→room temp., 16 h, 82 %. e) 2‐aminophenylboronic acid hydrochloride, K_2_CO_3_, (Ph_3_P)_2_PdCl_2_, DME: EtOH: H_2_O 4:1:1, 80 °C, 12 h, 97 %. f) *p*TsOH⋅H_2_O, MeCN, NaNO_2_, KI, MeCN, H_2_O, 10 °C→room temp., 2 h, 85 %.

Synthesis of backbone **29** is displayed in Scheme 5, starting with introducing a CPDIPS‐acetylene. The Sonogashira reaction between 3‐iodoaniline (**20**) and CPDIPS‐acetylene provided molecule **21** in quantitative yields. In the subsequent iodination reaction^[24]^ not only structure **22** was obtained in 85 % yield, but side products **23**, **24** and **25** were isolated (in 1.9 %, 1.6 %, and 4.9 % yield, respectively) and identified also. After screening for amine‐stable Suzuki–Miyaura reaction conditions, iodo‐aryl **22** was transformed into biphenyl **26** in 84 % isolated yield using dimethoxyethane DME/EtOH/H_2_O (4:1:1) as solvent mixture, K_2_CO_3_ as base, and (Ph_3_P)_2_PdCl_2_ as catalyst. In a Sandmeyer‐type reaction the amino group of biphenyl **26** was converted to an iodine substituent, providing **27** in a good 82 % yield. Interestingly, initial attempts with a similar building block as **26** exposing a HOP‐masked alkyne were low yielding, as all investigated reaction conditions gave mainly the elimination product of the HOP‐protection group (2‐methyl‐ethenyl substituted alkyne) as the main compound. Using similar Suzuki–Miyaura reaction conditions as before resulted in the successful assembly of the terphenyl backbone **28** from biphenyl **27** in excellent yield (97 %). The only variation was an increase of base equivalents (5 equiv. of K_2_CO_3_) in order to compensate for the hydrochloride salt of 2‐aminophenylboronic acid. Finally, a Sandmeyer‐type reaction afforded terphenyl **29** as backbone building block **B_2_** from precursor **28**.

To assemble **A_2_**, the asymmetric middle side chain **D_AS_** exposing two differently masked alkynes was required. Due to their favorable behavior in chromatography, HOP‐ and CPDIPS‐masked alkyne groups were selected. An iodine substituent was favored as leaving group X of **D_AS_**, promising the efficient coupling of the building block.

**Scheme 6:**
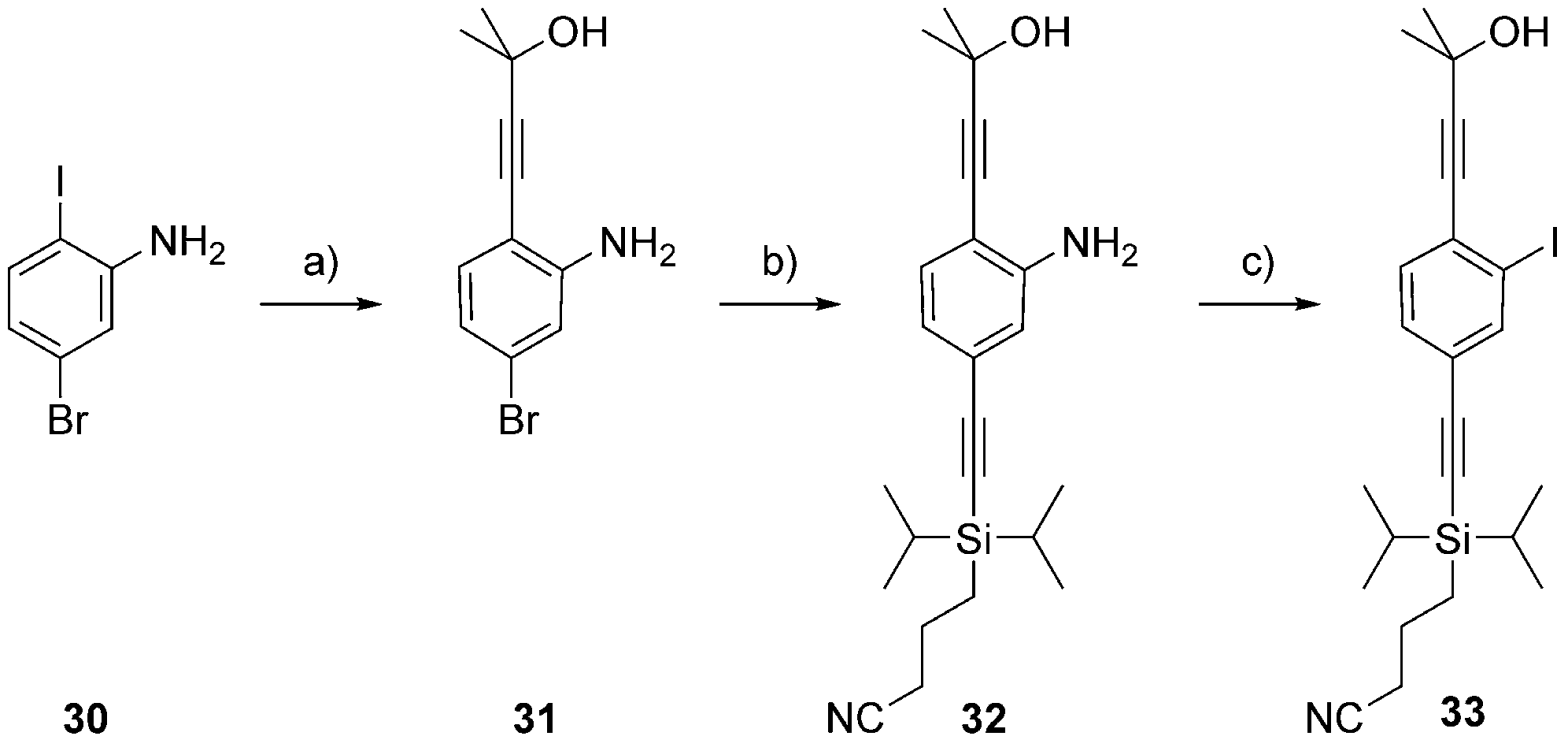
Synthesis of the asymmetric middle side chain building block **33**. Reaction and conditions: a) HOP‐acetylene, (Ph_3_P)_2_PdCl_2_, CuI, THF: piperidine 3:1, room temp., 1 h, 89 %. b) CPDIPS‐acetylene, (Ph_3_P)_2_PdCl_2_, CuI, THF: piperidine 3:1, 80 °C, 1 h, 87 %. c) *p*TsOH⋅H_2_O, NaNO_2_, KI, MeCN, H_2_O, 10 °C→room temp., 14 h, 60 %.

Starting with commercial 5‐bromo‐2‐iodoaniline (**30**; Scheme 6), two consecutive Sonogashira reactions allowed to introduce both acetylenes. First, the iodine in amine **30** reacted at room temperature, giving HOP‐acetylene **31** in very good 89 % isolated yield. For the reaction of molecule **31** with CPDIPS‐acetylene, similar reaction conditions but elevated temperature (80 °C) were applied, providing amine **32** in 87 % isolated yield. Again, the amine **32** was converted into analogous iodide **33** in a Sandmeyer‐type reaction. The latter turned out to be rather challenging, as compound **33**, the middle side chain building block **D_AS_**_,_ was isolated in only 60 % yield.

**Scheme 7:**
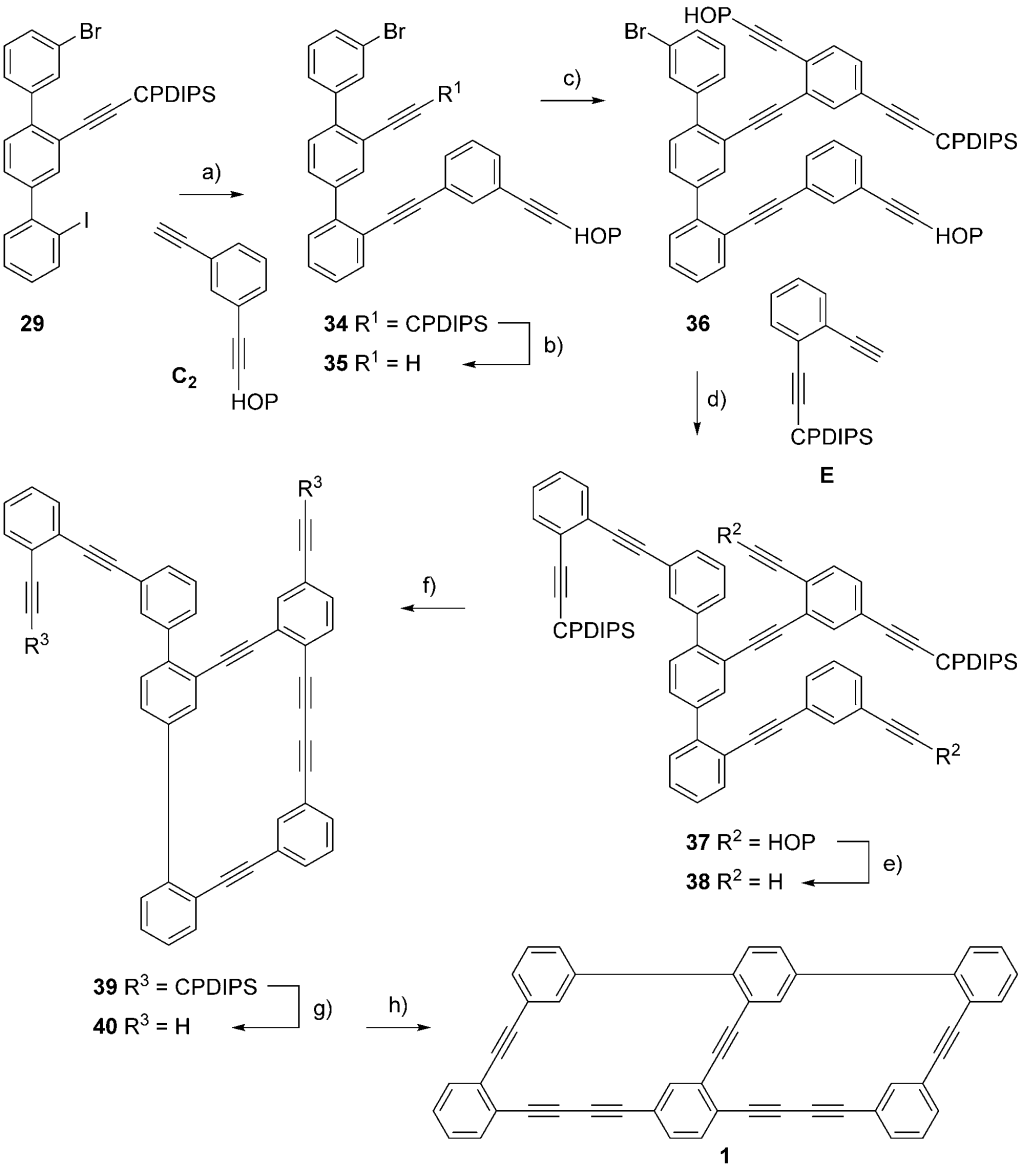
Assembly of the precursor **A_2_** (represented by **37**) and endgame to the bicycle **1**. Reagents and conditions: a) **C_2_**, (Ph_3_P)_2_PdCl_2_, CuI, THF/piperidine: 3/1, room temp., 11 h, 92 %. b) TBAF, THF, room temp., 1 h., 97 %. c) **33**, (Ph_3_P)_2_PdCl_2_, CuI, THF/piperidine: 3/1, room temp., 16 h, 97 %. d) **E**, (Ph_3_P)_2_PdCl_2_, CuI, piperidine, 120 °C, 11 h, 65 %. e) NaOH, toluene, 120 °C, 20 min., 95 %. f) CuCl, Cu(OAc)_2_, pyridine, syringe‐pump, room temp., 10.5 h, 94 %. g) TBAF, THF, room temp., 30 min., 26 %. h) CuCl, Cu(OAc)_2_, pyridine, syringe‐pump, room temp., 3 h, 85 %.

With all necessary building blocks available, the assembly of the bicyclic target structure **1** started by attaching all three side chains (**C_2_**, **33**=**D_AS_**, **E**) to the terphenyl backbone **29** with Sonogashira reactions (Scheme 7). The free acetylene **C_2_** was obtained quantitatively by treating the previously published HOP and CPDIPS protected 1,3‐diethynylbenzene^[21]^ with tetra‐*n*‐butylammonium fluoride (TBAF) in THF. The different reactivity of both halogen substituents (iodine and bromide) of backbone **29** allowed to substitute the iodine at room temperature selectively. Using standard conditions ((Ph_3_P)_2_PdCl_2_, CuI, THF, piperidine), **C_2_** was coupled to the backbone **29**, providing structure **34** in good 92 % yield. The CPDIPS protection group was removed selectively by treating molecule **34** with TBAF in THF, giving intermediate **35** in nearly quantitative yield. The subsequent Sonogashira coupling between molecule **35** and aryliodide **33** turned out to be challenging due to the pronounced tendency of molecule **35** towards dimerization by oxidative homocoupling. However, treating the glassware with concentrated sulfuric acid to remove copper residues and excessive degassing of the solvent mixture (THF/ piperidine: 3/1) with argon enabled the assembly of compound **36** at room temperature in excellent 97 % isolated yield, using ((Ph_3_P)_2_PdCl_2_, CuI) as catalytic system. The third consecutive Sonogashira reaction required elevated reaction temperature, like already reported for the coupling between precursors **16** and **17**. Treating compounds **36** and **E_2_** with the mentioned, usual catalyst combination at 120 °C in piperidine gave structure **37** in reasonable 65 % isolated yield.

The complex phenylene‐ethynylene architecture **37** comprises all carbon atoms of the target structure, and with its two pairs of differently masked acetylenes, it constitutes the desired precursor **A_2_** enabling the consecutive closing of both macrocycles. The orthogonal nature of the acetylene protection groups even allows choosing the order of ring‐closing.

First, both HOP protection groups were removed in a retro‐Favorskii reaction. The selected order of deprotection was due to the harshness of the reaction conditions required. Thus, after exposing structure **37** for 20 minutes to sodium hydroxide in toluene at 120 °C, the doubly deprotected derivative **38** was isolated in excellent 95 % yield. Pseudo high dilution conditions were applied for the first macrocyclization by dropping diacetylene **38** slowly into the reaction mixture with a syringe pump. For this oxidative acetylene coupling, Eglinton‐Breslow conditions (CuCl, Cu(OAc)_2_, pyridine)[^31^, ^32^] were preferred over more common Glaser‐Hay conditions (CuCl, TMEDA, O_2_),^[33]^ and thus, diacetylene **38** was added into copper chloride (CuCl) and copper acetate (Cu(OAc)_2_) in pyridine at room temperature over a period of 9.5 h. The reaction was stirred for one more hour after completing the addition before macrocycle **39** was isolated in a very good 94 % yield after work‐up. Subsequent deprotection of the second pair of acetylenes using TBAF in THF provided the direct precursor **40**. While the deprotection reaction was performed in very good 95 % yield at small scale (20 mg), a surprising scaling behavior was observed with the main batch (150 mg) for which only poor 26 % yield could be isolated. Due to the late stage of the assembly and the lack of additional material, the origin of the drop in yield was not investigated further.

For the macrocyclization of precursor **40** into the desired target structure **1**, similar oxidative acetylene coupling conditions were applied as described before for the macrocyclization of diacetylene **38**. However, for the addition of structure **40** by the syringe pump, 2 h proved to be sufficient. After stirring the reaction mixture at room temperature for another hour, macrocycle **1** was isolated in excellent 85 % yield as brown solid.

### Characterization, conformation and stability of bicycle 1

The bicyclic target structure **1** was characterized by ^1^H and ^13^C NMR spectroscopy (Supporting Information, Figure 1) as well as high‐resolution mass spectrometry (HR‐MS).

2D NMR spectra unambiguously corroborated the topology of the central terphenyl backbone and the three protruding ethynyl‐phenyl side arms. The connectivity of the inner alkyne carbons (C_31_, C_32_, C_49_, C_50_) to the outer alkyne carbon atoms (C_29_, C_30_, C_47_, C_48_) within the buta‐1,3‐diyine units could, however, not be monitored, as there is no suitable long‐range ^3^J or ^4^J H–C coupling constant for an HMBC‐type experiment. Also, natural abundance carbon‐carbon correlation experiments were not feasible due to the low amount and limited stability of compound **1**. Assignment of the inner alkyne carbon atoms to the four 2D‐uncorrelated resonances in the alkyne region of the ^13^C{^1^H} NMR spectrum was achieved to a satisfying degree by comparison with DFT calculated chemical shifts.

**Figure 2:**
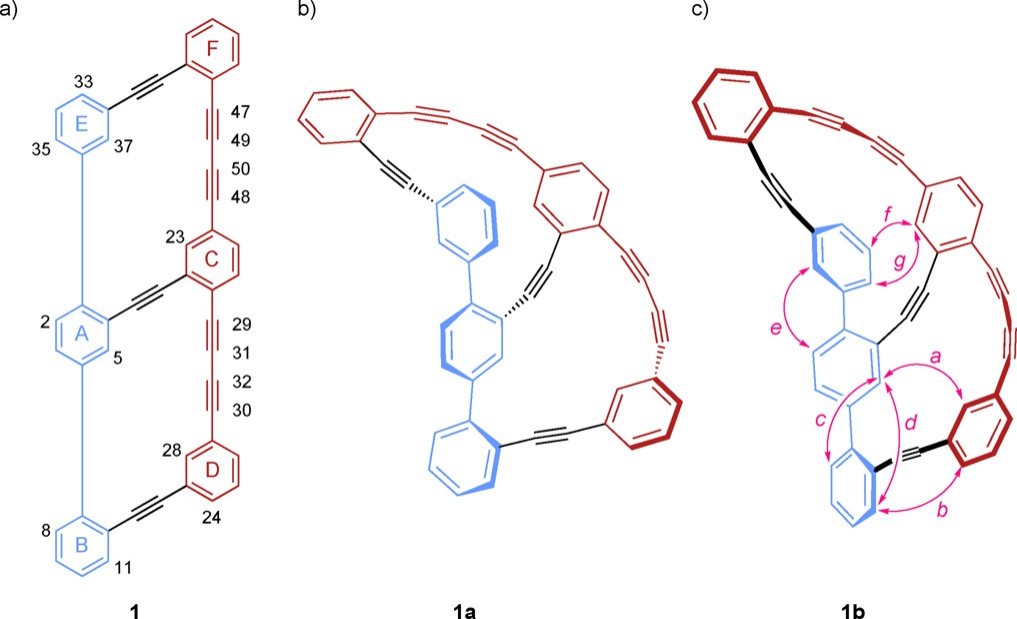
Structure of molecule **1** a) Compound **1** labeled with the most important carbon/proton numbers. b) Structures **1 a** and **1 b** were calculated using the B3LYP functional, a triple‐zeta basis, the RIJCOSX approximation and DFT‐D3BJ dispersion correction (ORCA 4.1.2). c) The structure **1 b** as indicated by NMR studies, NOEs of compound **1** are shown in pink.

The analysis of the three‐dimensional arrangement of the dissolved bicyclic target structure **1** by ^1^H–^1^H NOE spectra was surprising. The intention of the molecular design was to force the longer 1,4‐bis(phenylbuta‐1,3‐diyn‐1‐yl) benzene oligomer to wrap helically around the terphenyl axes resulting in a “Geländer” type arrangement as sketched in Figure 2 b (compound **1 a**). However, the recorded NOEs are not fully consistent with structure **1 a** and a bent 1,4‐bis(phenylbuta‐1,3‐diyn‐1‐yl) benzene oligomer remaining on the same side of the terphenyl subunit as displayed in Figure 2 c (compound **1 b**) seems to be more likely. The relative orientation of the lower four phenyl rings (A to D, c.f. Figure 2 a) is well defined by strong and characteristic NOE between H_5_ and H_28_ (*a*), as well as H_11_ and H_24_ (*b*). The fact that the NOEs from H_5_ to H_8_ (*c*) and H_11_ (*d*) are equally strong, indicates that phenyl rings A and B are not oriented orthogonally, but have a dihedral angle of considerably less than 90°. The distances (*c*) and (*d*) are 4.5 and 4.8 Å in the DFT structure for **1 b**, compared to 4.3 and 4.9 Å respectively in **1 a**, thus clearly pointing towards the structure **1 b** depicted in Figure 2 c. A similar pattern is observed for the relative orientation of rings A and E, where equally strong NOEs are expected from H_2_ to H_35_ and H_37_ for orthogonal phenyl rings **A** and **E**, but only one strong NOE for H_2_ to H_37_ (*e*) is found, while the remaining NOE is weak. This geometrical arrangement is however found for both, **1 a** as well as **1 b**. On the other hand, a comparison of distances H_11_ to H_24_ (*b*) with distances H_5_ to H_8_ (*c*), should yield a more intense NOE for distance (*b*) in structure **1 b** (4.3 vs. 4.5 Å) while structure **1 a** predicts the opposite (4.6 vs. 4.3 Å) and indeed, the NOE for (*b*) is significantly weaker. Unfortunately, the two distances that show the largest differences between structures **1 a** and **1 b**, namely H_23_ to H_34_ (*f*) and H_23_ to H_35_ (*g*) overlap with their diagonal peaks in C_6_D_6_ as well as in toluene‐D_8_ solutions (data not shown), so that no unambiguous assignment to either one of the structures **1 a** or **1 b** is feasible by NOE restraints.

Altogether, the recorded NOEs mildly favor a slightly bent terphenyl backbone with not entirely orthogonal phenyl rings A, B, and E alongside the substantially bent longer 1,4‐bis(phenylbuta‐1,3‐diyn‐1‐yl) benzene oligomer. Thus the dissolved arrangement sketched as **1 b** (Figure 2 c) resembles the banister of a staircase with an inserted floor (Figure 1 f) and not the intended helical staircase. ^13^C NMR chemical shifts depend on the bending of the acetylenic chains and analysis according to the work of Kreuzahler et al.^[34]^ reveal that the lower macrocyclic ring A‐C‐D‐B is considerably more strained than the upper one (A‐C‐F‐E) which is reflected in higher shift differences for the acetylenic carbons (12.3 and 9.0 ppm vs. 7.8 and 4.7 ppm). It also corroborates that the acetylenic moieties at the ends of compound **1** are more strained (12.3 and 7.8 ppm) than the ones adjacent to the central C phenyl ring (9.0 and 4.7 ppm).

It seems, however, difficult to quantify the difference in strain of the upper macrocycle (A‐C‐F‐E) for the two proposed structures **1 a** and **1 b**.

**Figure 3:**
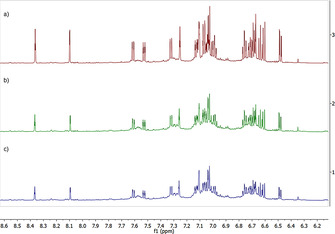
Stability of macrocycle **1** measured in [D_6_]benzene 100 % (the sample was measured under argon, in an NMR tube, sealed with a plastic lid and a Teflon strip, at room temperature). a) measured on the day of synthesis (under Argon) 100 % intensity. b) after all NMR measurements were finished (≈48 h, at room temperature, in the dark, under argon) 54 % intensity compared to the first NMR. c) Further 5 days at −26 °C in the dark, under Argon (43 % intensity compared to the first NMR).

The surprising NOE analysis not only disqualified the molecular design, but also challenged our chemical intuition. Thus, a burning issue was whether or not a simulation of the structure would have been able to predict the observed solution arrangement. Geometry optimization was performed for both arrangements **1 a** and **1 b** using the B3LYP functional, a triple‐zeta basis, the RIJCOSX approximation and DFT‐D3BJ dispersion correction as implemented in the ORCA release version 4.1.2. In contrast to the experimental data for bicycle **1** in solution, the calculations suggested the helical arrangement **1 a** to be more stable than **1 b** by almost 28 kJ mol^−1^ (**1 a**: −1917.896782035197 *E*_h_; **1 b**: −1917.886403451657 *E*_h_). We estimate the interconversion between **1 a** and **1 b** to have a barrier of about 15 kcal mol^−1^ (0.02436632 E_h_, 63.97 KJ mol^−1^, 15.29 kcal mol^−1^, see Supporting Information). While we had every intention to fully assess the conformation in solution by extended NMR investigations and potentially XRD, we faced a very fundamental challenge in the stability of the target compound. Its eagerness to react with molecular oxygen was already observed for the macrocycle **2** as structural synthon of the target structure, but this tendency is substantially more pronounced for bicycle **1**. As displayed in Figure 3, the intensity of the ^1^H NMR signals of **1** decreased to 54 % within 48 h, even though the solvent ([D_6_]benzene) was saturated with argon and the capped NMR tube was additionally sealed with a Teflon strip. While cooling slowed down the decomposition of **1**, it was not able to prevent it.

**Scheme 8:**
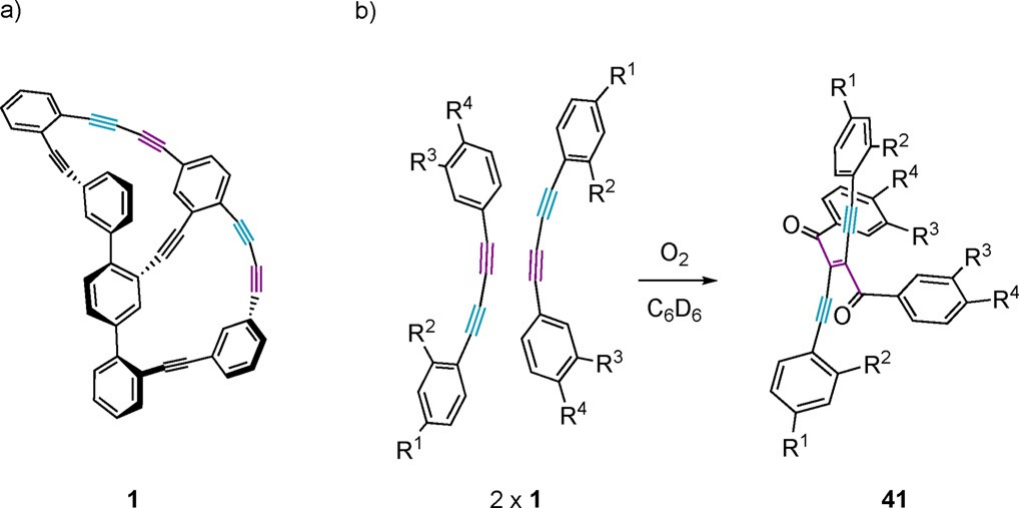
Oxidative dimerization of two molecules **1**. a) The two triple bonds in purple are expected to be the most reactive ones. Assuming exclusively the purple triple bonds to dimerize with O_2_, the presence of two comparably strained diacetylenes in **1** yields in 3 different combinations of oxidative dimers, summarized as **41**.

To identify the decomposition products, the partially degraded NMR sample was separated by SEC. Two main peaks were detected, from which the one with the longer retention time was identified as the parent bicycle **1** (2.4 mg, 3.84 μmol, 12 %). The peak with the shorter retention time (4.6 mg) gave a signal in the high‐resolution mass spectrometer of *m*/*z=*1276.33, which corresponds to the molecular formula C_100_H_44_O_2_, expected for the oxygen triggered dimerization of **1**. The NMR spectrum of the peak agreed with the hypothesized mixture of three compounds with two carbonyls each. In analogy to the macrocyclic model compound **2**, for which regioselectively one of both acetylenes of the diacetylene bridge was engaged in the oxidative dimerization (purple in Scheme 8),^[21]^ we assume similar preferences for **1**, consisting of two merged molecules **2**. However, the two diacetylene bridges of **1** allow three different combinations to form dimers, yielding in a mixture of oxidative dimers as structural isomers. The bifunctionality of **1** with respect to oxygen triggered dimerization most likely also results in larger oligo‐ and polymers, which explains the loss of material during the analysis of the degrading NMR sample.

This hypothesis of oxidative dimers **41** as degradation products was further supported by diffusion ordered spectroscopy (DOSY). The diffusion coefficient of product **1** in C_6_D_6_ was determined to be 6.34(1)×10^−10^ m^2^ s^−1^, while the diffusion coefficient of **41** was with 4.28(1)×10^−10^ m^2^ s^−1^ significantly lower. With a simple model assuming spherical moieties, the volume of the later including the first shell of solvation was calculated via the *Stokes Einstein* equation to be 3.25 times the volume of **1**, which is in reasonable agreement with the suggested dimeric structures **41**.

Of particular interest for “Geländer” structures are their racemization barriers. Even though the NOE‐NMR investigations challenge the helical arrangement of the banister, the suggested strongly bent alongside arrangement of both oligomer strands results in a pair of enantiomers also. Thus the question concerning the activation energy involved in the molecular enantiomerization process, macroscopically observed as racemization of the sample, remains valid. Freshly purified samples of **1** were thus subjected to variable‐temperature (VT) HPLC on a chiral stationary phase. (Chiralpak IA, eluent *n*‐hexane:*i*PrOH, 98:2, 1.0 mL min^−1^, column oven temperature: *T*=15–22 °C). Over the entire temperature range, the elution profile displayed the separation of both enantiomers as peaks, with a substantial fraction of the sample as plateau in between both peaks, indicating structural flexibility of macrocycle **1** and making the isolation of pure enantiomers under ambient conditions impossible (a representative HPLC trace is displayed as skipping rope in the TOC graphic). To be able to estimate the racemization barrier of **1**, its elution profiles in the temperature range from 288 to 298 K in steps of 1 K were recorded and analyzed (DCXplorer software packages).[^35^, ^36^] A half‐life of the enantiomerization of only *t*_1/2_^293 K=^159±1 s with an activation free energy of Δ*G* ^≠^_293 K_≈86.6±1.8 kJ mol^−1^ was obtained. In spite of the very limited applied temperature window guaranteeing the survival of the chiral column, an Eyring plot enabled to estimate the composition of the activation free energy into its enthalpy (Δ*H*_e_^≠^≈75.1±0.9 kJ mol^−1^) and entropy (Δ*S*_e_^≠^≈−39.3±3 J mol^−1^ K^−1^) contributions.

**Figure 4:**
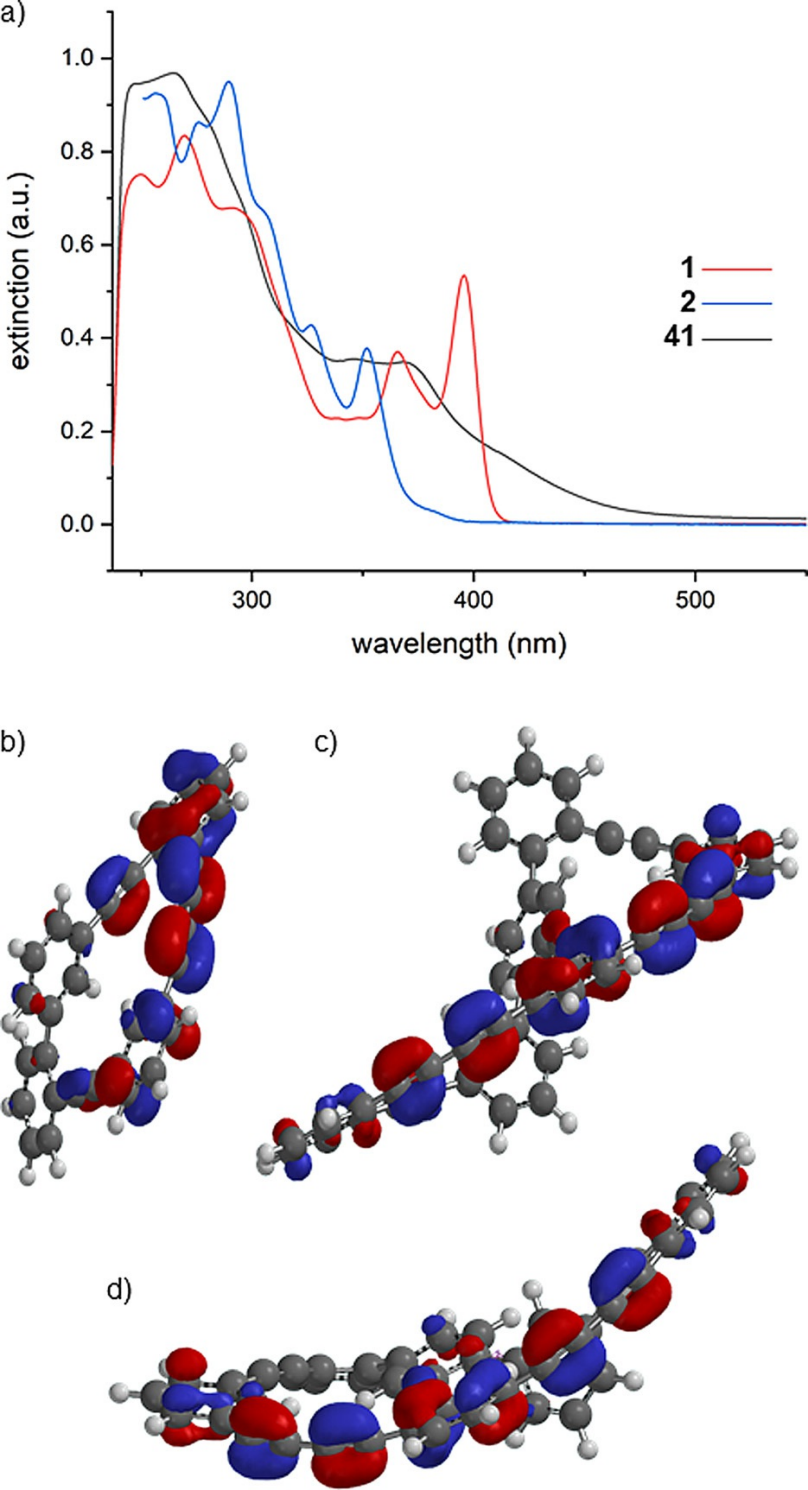
a) UV/Vis spectra of the bicycle **1**, its O_2_‐triggered dimer fraction **41**, and the macrocycle **2** (**1** and **41** recorded in CHCl_3_, **2** in CH_2_Cl_2_). b) HOMO of macrocycle **2**. c) HOMO of the bicycle **1 a** in the intended “Geländer”‐type helical arrangement. d) HOMO of the bicycle **1 b** in the arrangement observed for the dissolved molecule by NOE NMR experiments.

Qualitative UV/VIS spectra of the target structure **1**, its O_2_‐triggered degradation dimers **41**, and its macrocyclic subunit **2** are displayed in Figure 4 a). Unfortunately, macrocycle **2** and bicycle **1**, as members of a series of oligomers, are not recorded in the same solvent. Due to the intrinsic lability of the target structure **1**, its electronic absorption spectrum was recorded directly after separation by SEC dissolved in CHCl_3_. During the same process also the dimer fraction **41** was recorded. The UV/VIS spectrum of macrocycle **2**, on the other hand, was previously recorded in CH_2_Cl_2_.^[21]^ Again, the poor storing stability of **2** avoided the later recording of a spectrum in CHCl_3_.

Fortunately, UV/VIS spectra recorded in CH_2_Cl_2_ and CHCl_3_ should at least be qualitatively comparable.^[37]^ As expected, the extended conjugated π‐system in the banister of **1** is reflected in a large bathochromic shift compared to **2**. While compound **2** has a maximum at 289 nm and two additional maxima at 326 nm and 351 nm, the bicyclic system **1** has a maximum at 270 nm, and two additional maxima at 366 nm and 396 nm. To illustrate the delocalization of the π‐system in the banister, the HOMOs of the two members of the series are displayed in Figure 4 b–d. The UV/VIS spectrum of the dimer fraction **41** hardly displays well‐defined peaks, but a broad absorption is tailing out to values above 450 nm.

## Conclusions

In summary, bicycle **1** as a potentially new “Geländer”‐type structure with a conjugated helical banister (Figure 1 a) was successfully synthesized over 14 linear steps (5.5 % overall yield) and fully characterized by NMR and HR‐MS. In the convergent synthetic strategy, both macrocyclic subunits were closed consecutively, avoiding structural isomers of different ring sizes. In spite of a calculated energy penalty Δ*E* of about 28 kJ mol^−1^, the analysis of the spatial arrangement of the dissolved target structure by NOE NMR experiments suggests an alternative arrangement with both oligomer strands bent alongside, referred to as **1 b**, and resembling the banister of a staircase with an inserted floor (Figure 1 f). The target structures’ racemization barrier was estimated to be Δ*G* ^≠^_293 K_≈86.6±1.8 kJ mol^−1^ based on variable‐temperature HPLC experiments on a chiral stationary phase. In analogy to the macrocycle **2**, the bicyclic system **1** with its strained 1,4‐bis(phenylbuta‐1,3‐diyn‐1‐yl) benzene banister displayed a pronounced tendency to dimerize with O_2_.

The limited stability of the target structure, along with the oxidative reactivity and the observed, unexpected spatial arrangement of the dissolved bicyclic system, disqualifies the investigated oligomer combinations for both, extended and/or additionally functionalized “Geländer” molecules.

We are currently working on an alternative, more modular approach to “Geländer” systems comprising a conjugated banister.

## Experimental Section

CPDIPS‐acetylene,[^21^, ^28^] 4‐(((2‐(3‐Hydroxy‐3‐methylbut‐1‐yn‐1‐yl)phenyl)ethynyl)diisopropylsilyl) butanenitrile^[21]^ and 4‐(((3‐(3‐Hydroxy‐3‐methylbut‐1‐yn‐1‐yl)phenyl)ethynyl)diisopropylsilyl) butanenitrile^[21]^ were synthesized according to already published methods. The syntheses of compounds **5**, **6**, **7**, **8**, **9**, **10**, **12**, **13**, **14**, **15**, **16**, **E**, **17**, **18**, **C1**, **21**, **22**, **23**, **24**, **25**, **26**, **27**, **28**, **29**, **31**, **32**, **33** and **C2** are described in the Supporting Information.

### General remarks

All chemicals were directly used for the synthesis without further purification if nothing else stated. Dry solvents were used as crown cap and purchased from Acros, Aldrich, and Fluorochem. NMR solvents were obtained from CIL Cambridge Isotope Laboratories, Inc. (Andover, MA, USA) or Aldrich. All NMR experiments were performed on Bruker Avance III or III HD, two or four‐channel NMR spectrometer operating at 400.13, 500.13, or 600.13 MHz proton frequency. The instruments were equipped with a direct‐observe 5 mm BBFO smart probe (400, 500, and 600 MHz), an indirect‐detection 5 mm BBI probe (500 MHz), or a five‐channel cryogenic 5 mm QCI probe (600 MHz). All probes were equipped with actively shielded *z*‐gradients (10 A). The experiments were performed at 298 K. All chemical shifts (*δ*) are reported in ppm relative to the used solvent, and coupling constants (*J*) are given in Hertz (Hz). The measurements are performed at room temperature. The multiplicities are written as: s=singlet, d=doublet, t=triplet, q=quartet, quint=quintet, dd=doublet of doublet, m=multiplet. DEPT‐135 experiments were performed twice for samples containing terminal alkynes using INEPT delays corresponding to ^1^ *J*_CH_ coupling constants of 145 and 200 Hz (Reported in the supporting information). Compound **1** was fully assigned using standard COSY, TOCSY, HSQC, HMBC, and NOESY (mixing time 1 s) experiments. The diffusion coefficients were determined in an PFGSE (pulsed field gradient spin echo) diffusion experiment using a bipolar gradient pulse sequence.^[38]^ The diffusion time was set to 35 ms, the Eddy current time to 5 ms, and the gradient length to 1.5 ms. Gradients with a smoothed square shape (SMSQ10.100) were increased linearly in 8 steps from 5 to 95 % (2.41 to 45.74 G cm^−1^). The sigmoidal intensity decrease was fitted with a two‐parameter fit (I0 and diffusion coefficient D) with the dosy routine implemented in topspin 3.5 [Bruker Biospin GmbH, 2017]. A Shimadzu GC‐MS‐QP2010 SE gas chromatograph system, with a ZB‐5HT inferno column (30 m×0.25 mm×0.25 mm), at 1 mL min^−1^ He‐flow rate (split=20:1) with a Shimadzu mass detector (EI 70 eV) was used. For column chromatography, SilicaFlashR P60 from SILICYCLE was used with a particle size of 40–63 μm (230–400 mesh). For neutral column chromatography, SilicaFlashR P60 from SILICYCLE was used with a particle size of 40–63 μm (230–400 mesh) and modified by adding Buffer solution pH7 (Fluka). Therefore, a mixture of 1 kg of silica gel and 100 mL of diluted buffer solution (1:25, buffer: H_2_O) was subjected to rotation overnight. Recycling size‐exclusion chromatography (SEC) was performed with a Shimadzu Prominence System equipped with SDV preparative columns from Polymer Standards Service (two Showdex columns in series, 20×600 mm each, exclusion limit: 30 000 g mol^−1^) with chloroform as solvent. UV/Vis absorption spectra were recorded on a Jasco V‐770 Spectrophotometer. The UV/Vis spectra were measured in a 1 cm quartz glass cuvettes directly after the SEC purification. For HPLC, a Shimadzu LC‐20AT HPLC was used equipped with a diodearray UV/Vis detector (SPD‐M20A VP from Shimadzu, *λ*=200–600 nm) and a column oven Shimadzu CTO‐20AC. The used column for separation on chiral stationary phase was a Chiralpak IA, 5 μm, 4.6×250 mm, Daicel Chemical Industries Ltd. High‐resolution mass spectra (HRMS) were measured with a Bruker Maxis 4G ESI‐TOF instrument, a Bruker solariX spectrometer with a MALDI source or EI spectra were measured on a Waters Micromass AutoSpec Ultima (EI‐Sector).

### Synthesis and characterization

**4‐(((3‐Bromo‐2′′‐((3‐(3‐hydroxy‐3‐methylbut‐1‐yn‐1‐yl)phenyl)ethynyl)‐[1,1′:4′,1′′‐terphenyl]‐2′‐yl)ethynyl)diisopropylsilyl)butanenitrile (34)**: An oven‐dried and argon flushed Schlenk tube was charged with **29** (1.92 mg, 3.00 μmol, 1.0 equiv), **C_2_** (691 mg, 3.75 mmol, 1.25 equiv.) dry THF (13.5 mL) and piperidine (4.5 mL) and the mixture degassed by passing argon through for 10 min. Then (Ph_3_P)_2_PdCl_2_ (105 mg, 150 μmol, 0.05 equiv.) and CuI (35.0 mg, 180 μmol, 0.06 equiv.) were added. The thick suspension was degassed by passing argon through for a further 5 min. The dark brown suspension was stirred at room temperature for 11 h. After the reaction was completed according to TLC, the solution was diluted with EtOAc (100 mL) and washed with H_2_O (50 mL), brine (50 mL), and dried over Na_2_SO_4_. The organic phase was concentrated under reduced pressure and subjected to column chromatography (340 g SiO_2_, Cy:EtOAc 94:6→60:40). Compound **34** (1.93 g, 2.77 mmol, 92 %) was obtained as a colorless foam. *R*_f_=0.22 (Cy/EtOAc, 3:1). ^1^H NMR (500 MHz, C_6_D_6_): *δ*=8.06 (d, *J=*1.8, H_Ar_, 1 H), 7.71 (t, *J=*1.8, H_Ar_, 1 H), 7.62 (td, *J=*1.6, 0.6, H_Ar_, 1 H), 7.61–7.58 (m, H_Ar_, 1 H), 7.54 (dd, *J=*8.0, 1.9, H_Ar_, 1 H), 7.32 (ddd, *J=*8.1, 2.0, 1.0, H_Ar_, 1 H), 7.29–7.25 (m, H_Ar_, 2 H), 7.22 (dt, *J=*7.8, 1.4, H_Ar_, 1 H), 7.17–7.14 (m, H_Ar_, 1 H), 7.04–6.97 (m, H_Ar_, 3 H), 6.95 (t, *J=*7.9, H_Ar_, 1 H), 6.84 (td, *J=*7.8, 0.6, H_Ar_, 1 H), 1.56 (t, *J=*6.9, CH_2_, 2 H), 1.44 (s, 2× CH_3_, 6 H), 1.33–1.25 (m, CH_2_, 2 H), 1.01 (d, *J=*7.3, 2× CH_3_, 6 H), 0.96 (d, *J=*7.3, 2 *x* CH_3_, 6 H), 0.94–0.81 (m, 2× CH, 2 H), 0.51–0.45 ppm (m, CH_2_, 2 H). ^13^C NMR (126 MHz, C_6_D_6_): *δ*=142.7 (1C, C_Ar_), 142.7 (1C, C_Ar_), 142.3 (1C, C_Ar_), 140.4 (1C, C_Ar_), 134.9 (1C, C_Ar_), 134.9 (1C, C_Ar_), 133.6 (1C, C_Ar_), 132.8 (1C, C_Ar_), 131.7 (1C, C_Ar_), 131.4 (1C, C_Ar_), 130.8 (1C, C_Ar_), 130.4 (1C, C_Ar_), 130.1 (1C, C_Ar_), 129.7 (1C, C_Ar_), 129.5 (1C, C_Ar_), 129.2 (1C, C_Ar_), 128.8 (1C, C_Ar_), 124.1 (1C, C_Ar_), 124.0 (1C, C_Ar_), 122.3 (1C, C_Ar_), 121.8 (1C, C_Ar_), 121.8 (1C, C_Ar_), 119.4 (1C, CN), 107.2 (1C, C_alkyne_), 95.8 (1C, C_alkyne_), 93.7 (1C, C_alkyne_), 92.6 (1C, C_alkyne_), 90.2 (1C, C_alkyne_), 81.4 (1C, C_alkyne_), 65.2 (1C, C_tert_), 31.6 (2C, CH_3_), 21.4 (1C, CH_2_), 20.4 (1C, CH_2_), 18.4 (2C, CH_3_), 18.1 (2C, CH_3_), 12.0 (2C, CH), 9.6 ppm (1C, CH_2_). The peaks of 128.3 and 127.9 are only visible in the DEPT‐135 experiment, as the signals are overlain by C_6_D_6_. HR‐MS (ESI, MeOH) calcd For C_43_H_42_BrNNaOSi^+^: [*M*+Na]^+^, 718.2111; found [*M*+Na]^+^, 718.2098.

**4‐(3‐((3′′‐Bromo‐3′‐ethynyl‐[1,1′:4′,1′′‐terphenyl]‐2‐yl)ethynyl)phenyl)‐2‐methylbut‐3‐yn‐2‐ol (35)**: A two‐neck round‐bottom flask was cleaned by the following treatment to remove all copper‐ions from previous reactions. The flask was filled with conc. H_2_SO_4_ and sonicated for 10 min, followed by washing with H_2_O, NaOH (1 m), H_2_O, and acetone. The flask was dried in the heating oven overnight and flushed with argon. To a solution of **34** (447 mg, 642 μmol, 1.0 equiv.) in THF (33 mL) was added TBAF (1 m in THF, 1.3 mL, 1.28 mmol, 5.0 equiv) and the reaction mixture stirred at room temperature for 1 h under argon. After the reaction was completed according to TLC, the solution was diluted by the addition of water (100 mL), and the aqueous phase was extracted with EtOAc (200 mL). The organic layer was washed with water (100 mL) and brine (100 mL) and dried over Na_2_SO_4_. The organic phase was concentrated under reduced pressure and subjected to column chromatography (100 g SiO_2_, Cy:EtOAc 94:6→65:35). Compound **35** (320 mg, 621 μmol, 97 %) was obtained as white solid. *R*_f_=0.26 (Cy/EtOAc, 3:1). ^1^H NMR (500 MHz, C_6_D_6_): *δ*=8.10 (dd, *J=*2.0, 0.5, H_Ar_, 1 H), 7.75 (td, *J=*1.7, 0.6, H_Ar_, 1 H), 7.73–7.70 (m, H_Ar_, 1 H), 7.58 (ddd, *J=*7.5, 1.6, 0.5, H_Ar_, 1 H), 7.44 (dd, *J=*8.0, 2.0, H_Ar_, 1 H), 7.36 (ddd, *J=*7.7, 1.7, 1.0, H_Ar_, 1 H), 7.28–7.23 (m, H_Ar_, 2 H), 7.17 (ddd, *J=*7.8, 1.7, 1.2, H_Ar_, 1 H), 7.13–7.11 (m, H_Ar_, 1 H), 7.01 (td, *J=*7.5, 1.6, H_Ar_, 1 H), 6.99–6.94 (m, H_Ar_, 2 H), 6.86–6.81 (m, H_Ar_, 1 H), 6.76 (td, *J=*7.8, 0.6, H_Ar_, 1 H), 2.82 (s, H_alkyne_, 1 H), 1.37 ppm (s, 2×CH_3_, 6 H). ^13^C NMR (126 MHz, C_6_D_6_): *δ*=142.7 (1C, C_Ar_), 142.5 (1C, C_Ar_), 142.2 (1C, C_Ar_), 140.2 (1C, C_Ar_), 135.3 (1C, C_Ar_), 135.3 (1C, C_Ar_), 133.4 (1C, C_Ar_), 132.7 (1C, C_Ar_), 131.5 (1C, C_Ar_), 131.3 (1C, C_Ar_), 130.8 (1C, C_Ar_), 130.3 (1C, C_Ar_), 129.8 (1C, C_Ar_), 129.7 (1C, C_Ar_), 129.6 (1C, C_Ar_), 129.2 (1C, C_Ar_), 128.7 (1C, C_Ar_), 128.3 (1C, C_Ar_), 124.1 (1C, C_Ar_), 124.0 (1C, C_Ar_), 122.5 (1C, C_Ar_), 121.9 (1C, C_Ar_), 120.9 (1C, C_Ar_), 95.6 (1C, C_alkyne_), 92.8 (1C, C_alkyne_), 90.3 (1C, C_alkyne_), 82.9 (1C, C_alkyne_), 81.7 (1C, C_alkyne_), 81.5 (1C, C_alkyne_), 65.2 (1C, C_tert_), 31.5 ppm (2C, CH_3_). The peak of 127.8 is only visible in the DEPT‐135 experiment, as the signal is overlain by C_6_D_6_. HR‐MS (ESI, MeOH) calcd For C_33_H_23_BrNaO^+^: [*M*+Na]^+^, 537.0824; found [*M*+Na]^+^, 537.0821.

**4‐(((3‐((3‐Bromo‐2′′‐((3‐(3‐hydroxy‐3‐methylbut‐1‐yn‐1‐yl)phenyl)ethynyl)‐[1,1′:4′,1′′‐terphenyl]‐2′‐yl)ethynyl)‐4‐(3‐hydroxy‐3‐methylbut‐1‐yn‐1‐yl)phenyl)ethynyl)diisopropylsilyl)butanenitrile (36)**: A Schlenk tube was cleaned by the following treatment to remove all copper‐ions from previous reactions. The flask was filled with conc. H_2_SO_4_ and sonicated for 10 min, followed by washing with H_2_O, NaOH (1 m), H_2_O, and acetone. The oven‐dried and argon flushed Schlenk tube was charged with **35** (298 mg, 578 μmol, 1.2 equiv), **33** (237 mg, 482 μmol, 1.0 equiv.) dry THF (9.0 mL) and piperidine (3.0 mL), and the mixture degassed by passing argon through for 30 min. Then (Ph_3_P)_2_PdCl_2_ (11.8 mg, 16.8 μmol, 0.05 equiv.) and CuI (3.99 mg, 20.2 μmol, 0.06 equiv.) were added. The light‐yellow solution was degassed by passing argon through for a further 10 min. The reaction mixture was stirred at room temperature for 16 h. After the reaction was completed according to TLC, the turbid yellow mixture was diluted with EtOAc (100 mL), and the organic phase was washed with water (100 mL) and brine (100 mL) and dried over Na_2_SO_4_. The organic phase was concentrated under reduced pressure and subjected to column chromatography (340 g SiO_2_, Cy:EtOAc 92:8→35:65). Compound **36** (320 mg, 621 μmol, 97 %) was obtained as white solid. *R*_f_=0.26 (Cy/EtOAc, 2:1). ^1^H NMR (500 MHz, C_6_D_6_): *δ*=8.33 (d, *J=*1.9, H_Ar_, 1 H), 7.99 (t, *J=*1.8, H_Ar_, 1 H), 7.73 (dd, *J=*1.7, 0.6, H_Ar_, 1 H), 7.70 (td, *J=*1.7, 0.6, H_Ar_, 1 H), 7.66–7.63 (m, H_Ar_, 1 H), 7.62–7.59 (m, H_Ar_, 1 H), 7.48 (ddd, *J=*7.7, 1.8, 1.0, H_Ar_, 1 H), 7.41 (dt, *J=*2.1, 1.1, H_Ar_, 1 H), 7.40 (t, *J=*1.4, H_Ar_, 1 H), 7.34–7.32 (m, H_Ar_, 1 H), 7.18 (ddd, *J=*7.7, 1.7, 1.2, H_Ar_, 1 H), 7.15–7.12 (m, H_Ar_, 2 H), 7.11–7.08 (m, H_Ar_, 2 H), 7.05–7.01 (m, H_Ar_, 1 H), 6.97 (t, *J=*7.9, H_Ar_, 1 H), 6.83–6.79 (m, H_Ar_, 1 H), 1.69 (s, 2× OH, 2 H), 1.61–1.57 (m, CH_2_, 2 H), 1.50–1.44 (m, CH_2_, 2 H), 1.42 (s, 2 *x* CH_3_, 6 H), 1.41 (s, 2 *x* CH_3_, 6 H), 1.13 (d, *J=*7.3, 2× CH_3_, 6 H), 1.07 (d, *J=*7.3, 2 *x* CH_3_, 6 H), 0.99–0.90 (m, 2 *x* CH, 2 H), 0.60–0.55 ppm (m, CH_2_, 2 H). ^13^C NMR (126 MHz, C_6_D_6_): *δ*=142.8 (1C, C_Ar_), 142.4 (1C, C_Ar_), 141.6 (1C, C_Ar_), 140.5 (1C, C_Ar_), 136.0 (1C, C_Ar_), 135.0 (1C, C_Ar_), 134.9 (1C, C_Ar_), 133.4 (1C, C_Ar_), 132.8 (1C, C_Ar_), 132.3 (1C, C_Ar_), 131.7 (1C, C_Ar_), 131.6 (1C, C_Ar_), 131.4 (1C, C_Ar_), 131.0 (1C, C_Ar_), 130.6 (1C, C_Ar_), 130.1 (1C, C_Ar_), 129.8 (1C, C_Ar_), 129.5 (1C, C_Ar_), 129.2 (1C, C_Ar_), 128.9 (1C, C_Ar_), 128.5 (1C, C_Ar_), 128.0 (1C, C_Ar_), 126.6 (1C, C_Ar_), 125.9 (1C, C_Ar_), 124.1 (1C, C_Ar_), 124.0 (1C, C_Ar_), 123.2 (1C, C_Ar_), 122.5 (1C, C_Ar_), 121.9 (1C, C_Ar_), 121.5 (1C, C_Ar_), 119.4 (1C, CN), 107.0 (1C, C_alkyne_), 101.1 (1C, C_alkyne_), 95.8 (1C, C_alkyne_), 94.0 (1C, C_alkyne_), 92.7 (1C, C_alkyne_), 92.3 (1C, C_alkyne_), 91.5 (1C, C_alkyne_), 90.4 (1C, C_alkyne_), 81.4 (1C, C_alkyne_), 80.7 (1C, C_alkyne_), 65.5 (1C, C_tert_), 65.2 (1C, C_tert_), 31.5 (2C, CH_3_), 31.5 (2C, CH_3_), 21.5 (1C, CH_2_), 20.4 (1C, CH_2_), 18.4 (2C, CH_3_), 18.2 (2C, CH_3_), 12.0 (2C, CH), 9.7 ppm (1C, CH_2_). HR‐MS (ESI, MeOH) calcd For C_56_H_52_BrNNaO_2_Si^+^: [*M*+Na]^+^, 900.2843; found [*M*+Na]^+^, 900.2830.

**4‐(((2‐((2′‐((5‐(((3‐Cyanopropyl)diisopropylsilyl)ethynyl)‐2‐(3‐hydroxy‐3‐methylbut‐1‐yn‐1‐yl)phenyl)ethynyl)‐2′′‐((3‐(3‐hydroxy‐3‐methylbut‐1‐yn‐1‐yl)phenyl)ethynyl)‐[1,1′:4′,1′′‐terphenyl]‐3‐yl)ethynyl)phenyl)ethynyl)diisopropylsilyl)butanenitrile (37)**: A Schlenk tube was cleaned by the following treatment to remove all copper‐ions from previous reactions. The flask was filled with conc. H_2_SO_4_ and sonicated for 10 min, followed by washing with H_2_O, NaOH (1 m), H_2_O, and acetone. The oven‐dried and argon flushed Schlenk tube was charged with **36** (410 mg, 466 μmol, 1.0 equiv), **E** (215 mg, 699 μmol, 1.5 equiv) and piperidine (15 mL), and the mixture degassed by passing argon through for 30 min. Then (Ph_3_P)_2_PdCl_2_ (16.4 mg, 23.3 μmol, 0.05 equiv) and CuI (5.43 mg, 28.0 μmol, 0.06 equiv) were added. The thick suspension was degassed by passing argon through for a further 5 min. The dark brown suspension was stirred at 120 °C for 11 h. After the reaction was completed according to TLC, the turbid yellow mixture was diluted with EtOAc (100 mL), and the organic phase was washed with water (100 mL) and brine (100 mL) and dried over Na_2_SO_4_. The organic phase was concentrated under reduced pressure and subjected to column chromatography (350 g SiO_2_, Cy:EtOAc 92:8→45:55) and SEC (CHCl_3_). Compound **37** (334 mg, 302 μmol, 65 %) was obtained as a brown foam. *R*_f_=0.18 (Cy/EtOAc, 2:1). ^1^H NMR (500 MHz, C_6_D_6_): *δ*=8.40 (d, *J=*1.9, H_Ar_, 1 H), 8.05 (t, *J=*1.7, H_Ar_, 1 H), 7.81 (ddd, *J=*7.8, 1.9, 1.2, H_Ar_, 1 H), 7.74–7.70 (m, H_Ar_, 2 H), 7.66 (dd, *J=*8.0, 2.0, H_Ar_, 1 H), 7.65–7.63 (m, H_Ar_, 2 H), 7.46 (t, *J=*1.1, H_Ar_, 1 H), 7.44 (t, *J=*1.1, H_Ar_, 1 H), 7.41–7.37 (m, H_Ar_, 2 H), 7.34 (dd, *J=*7.9, 5.7, H_Ar_, 2 H), 7.20 (dt, *J=*7.8, 1.4, H_Ar_, 1 H), 7.17–7.15 (m, H_Ar_, 1 H), 7.13–7.08 (m, H_Ar_, 2 H), 7.02 (td, *J=*7.6, 1.3, H_Ar_, 1 H), 6.88–6.83 (m, H_Ar_, 2 H), 6.81 (dd, *J=*7.7, 1.4, H_Ar_, 1 H), 1.62 (t, *J=*6.9, CH_2_, 2 H), 1.56 (m, CH_2_, 2 H), 1.53–1.48 (m, CH_2_, 2 H), 1.47–1.40 (m, CH_2_, 2 H), 1.46 (s, 2× CH_3_, 6 H), 1.42 (s, 2× CH_3_, 6 H), 1.10 (d, *J=*7.3, 4× CH_3_, 12 H), 1.04 (d, *J=*7.7, 2 *x* CH_3_, 6 H), 1.02 (d, *J=*7.7, 2× CH_3_, 6 H), 0.97–0.87 (m, 4 *x* CH, 4 H), 0.59–0.52 ppm (m, 2× CH_2_, 4 H). ^13^C NMR (126 MHz, C_6_D_6_): *δ*=142.8 (1C, C_Ar_), 142.5 (1C, C_Ar_), 140.9 (1C, C_Ar_), 140.5 (1C, C_Ar_), 135.9 (1C, C_Ar_), 135.0 (1C, C_Ar_), 134.9 (1C, C_Ar_), 133.5 (1C, C_Ar_), 133.3 (1C, C_Ar_), 133.0 (1C, C_Ar_), 132.5 (1C, C_Ar_), 132.4 (1C, C_Ar_), 131.7 (1C, C_Ar_), 131.5 (1C, C_Ar_), 131.5 (1C, C_Ar_), 131.1 (1C, C_Ar_), 130.6 (1C, C_Ar_), 130.4 (1C, C_Ar_), 129.8 (1C, C_Ar_), 129.6 (1C, C_Ar_), 129.2 (1C, C_Ar_), 128.9 (1C, C_Ar_), 128.6 (1C, C_Ar_), 128.6 (1C, C_Ar_), 126.7 (1C, C_Ar_), 126.6 (1C, C_Ar_), 126.0 (1C, C_Ar_), 126.0 (1C, C_Ar_), 124.1 (1C, C_Ar_), 124.1 (1C, C_Ar_), 123.9 (1C, C_Ar_), 123.2 (1C, C_Ar_), 121.8 (1C, C_Ar_), 121.6 (1C, C_Ar_), 119.5 (1C, CN), 119.4 (1C, CN), 107.0 (1C, C_alkyne_), 106.8 (1C, C_alkyne_), 101.3 (1C, C_alkyne_), 95.8 (1C, C_alkyne_), 94.5 (1C, C_alkyne_), 94.2 (1C, C_alkyne_), 93.9 (1C, C_alkyne_), 92.6 (1C, C_alkyne_), 92.4 (1C, C_alkyne_), 91.3 (1C, C_alkyne_), 90.4 (1C, C_alkyne_), 89.2 (1C, C_alkyne_), 81.3 (1C, C_alkyne_), 80.6 (1C, C_alkyne_), 65.5 (1C, C_tert_), 65.2 (1C, C_tert_), 31.5 (2C, CH_3_), 31.5 (2C, CH_3_), 21.6 (1C, CH_2_), 21.5 (1C, CH_2_), 20.4 (1C, CH_2_), 20.4 (1C, CH_2_), 18.5 (2C, CH_3_), 18.4 (2C, CH_3_), 18.2 (2C, CH_3_), 18.2 (2C, CH_3_), 12.1 (2C, CH), 12.0 (2C, CH), 9.7 (1C, CH_2_), 9.6 ppm (1C, CH_2_). The peaks of 128.0 and 128.3 are only visible in the DEPT‐135 experiment, as the signals are overlain by C_6_D_6_. HR‐MS (ESI, MeOH) calcd for C_76_H_76_N_2_NaO_2_Si_2_^+^: [*M*+Na]^+^, 1127.5338; found [*M*+Na]^+^, 1127.5324.

**4‐(((2‐((2′‐((5‐(((3‐Cyanopropyl)diisopropylsilyl)ethynyl)‐2‐ethynylphenyl)ethynyl)‐2′′‐((3‐ethynylphenyl)ethynyl)‐[1,1′:4′,1′′‐terphenyl]‐3‐yl)ethynyl)phenyl)ethynyl)diisopropylsilyl)butanenitrile (38)**: A two‐neck round‐bottom flask was cleaned by the following treatment to remove all copper‐ions from previous reactions. The flask was filled with conc. H_2_SO_4_ and sonicated for 10 min, followed by washing with H_2_O, NaOH (1 m), H_2_O, and acetone. The flask was dried in the heating oven overnight and flushed with argon. From a mixture of NaOH (57.6 mg, 1.44 mmol, 8.0 equiv) in toluene (35 mL) was distilled off the water for 15 min. Then **37** (199 mg, 180 μmol, 1.0 equiv) in toluene (5.0 mL) was added, and the mixture refluxed for 20 min. After the reaction was completed according to TLC (Cy: EtOAc; 4:1), the reaction mixture was cooled down to room temperature, and diluted with EtOAc (50 mL) washed successively with NH_4_Cl (2×50 mL) and brine (50 mL), and dried over Na_2_SO_4_. The organic phase was concentrated under reduced pressure and subjected to column chromatography (100 g SiO_2_, Cy:EtOAc 98:2→82:18). Compound **38** (169 mg, 171 μmol, 95 %) was obtained as a yellow oil. *R*_f_=0.21 (Cy/EtOAc, 5:1). ^1^H NMR (500 MHz, C_6_D_6_): *δ*=8.29 (d, *J=*1.9, H_Ar_, 1 H), 8.06–8.03 (m, H_Ar_, 1 H), 7.77‐ 7.73 (m, H_Ar_, 2 H), 7.67 (dt, *J=*7.7, 1.4, H_Ar_, 1 H), 7.63 (dd, *J=*1.7, 0.5, H_Ar_, 1 H), 7.61 (dd, *J=*7.7, 1.4, H_Ar_, 1 H), 7.57 (dd, *J=*8.0, 1.9, H_Ar_, 1 H), 7.44–7.42 (m, H_Ar_, 1 H), 7.42–7.37 (m, H_Ar_, 2 H), 7.31 (d, *J=*8.0, H_Ar_, 1 H), 7.28–7.23 (m, H_Ar_, 2 H), 7.18 (dt, *J=*7.8, 1.4, H_Ar_, 1 H), 7.13–7.10 (m, H_Ar_, 1 H), 7.10–7.07 (m, H_Ar_, 1 H), 7.04–7.00 (m, H_Ar_, 2 H), 6.84 (td, *J=*7.6, 1.5, H_Ar_, 1 H), 6.81–6.76 (m, H_Ar_, 2 H), 3.02 (s, H_Alkyne_, 1 H), 2.71 (s, H_Alkyne_, 1 H), 1.60–1.47 (m, 3× CH_2_, 6 H), 1.45–1.36 (m, CH_2_, 2 H), 1.11 (d, *J=*7.3, 2× CH_3_, 6 H), 1.08 (d, *J=*7.3, 2× CH_3_, 6 H), 1.03 (d, *J=*7.3, 2× CH_3_, 6 H), 1.02 (d, *J=*7.3, 2× CH_3_, 6 H), 0.97–0.85 (m, 4× CH, 4 H), 0.60–0.50 ppm (m, 2× CH_2_, 4 H). ^13^C NMR (126 MHz, C_6_D_6_): *δ*=142.9 (1C, C_Ar_), 142.7 (1C, C_Ar_), 141.0 (1C, C_Ar_), 140.3 (1C, C_Ar_), 135.8 (1C, C_Ar_), 135.6 (1C, C_Ar_), 135.0 (1C, C_Ar_), 133.4 (1C, C_Ar_), 133.3 (1C, C_Ar_), 133.0 (1C, C_Ar_), 132.9 (1C, C_Ar_), 132.4 (1C, C_Ar_), 132.1 (1C, C_Ar_), 131.9 (1C, C_Ar_), 131.4 (1C, C_Ar_), 131.1 (1C, C_Ar_), 130.5 (1C, C_Ar_), 130.5 (1C, C_Ar_), 129.8 (1C, C_Ar_), 129.7 (1C, C_Ar_), 129.2 (1C, C_Ar_), 128.8 (1C, C_Ar_), 128.7 (1C, C_Ar_), 128.6 (1C, C_Ar_), 127.2 (1C, C_Ar_), 126.6 (1C, C_Ar_), 126.0 (1C, C_Ar_) 125.2 (1C, C_Ar_), 124.1 (1C, C_Ar_) 123.8 (1C, C_Ar_), 123.6 (1C, C_Ar_), 123.3 (1C, C_Ar_), 121.9 (1C, C_Ar_), 121.5 (1C, C_Ar_), 119.4 (1C, CN), 119.3 (1C, CN), 106.9 (1C, C_alkyne_), 106.8 (1C, C_alkyne_), 94.5 (1C, C_alkyne_), 94.3 (1C, C_alkyne_), 94.1 (1C, C_alkyne_), 92.6 (1C, C_alkyne_), 92.6 (1C, C_alkyne_), 90.9 (1C, C_alkyne_), 90.4 (1C, C_alkyne_), 89.1 (1C, C_alkyne_), 84.0 (1C, C_alkyne_), 83.0 (1C, C_alkyne_), 81.9 (1C, C_alkyne_), 78.6 (1C, C_alkyne_), 21.6 (1C, CH_2_), 21.4 (1C, CH_2_), 20.4 (1C, CH_2_), 20.4 (1C, CH_2_), 18.5 (2C, CH_3_), 18.4 (2C, CH_3_), 18.2 (2C, CH_3_), 18.2 (2C, CH_3_), 12.1 (2C, CH), 12.0 (2C, CH), 9.7 (1C, CH_2_), 9.6 ppm (1C, CH_2_). The peaks of 127.9 and 128.3 are only visible in the DEPT‐135 experiment, as the signals are overlain by C_6_D_6_. HR‐MS (ESI, MeOH) calcd For C_70_H_64_N_2_NaSi_2_^+^: [*M*+Na]^+^, 1011.4500; found [*M*+Na]^+^, 1011.4489.

**Macrocycle 39**: CuCl, Cu(OAc)_2,_ and pyridine were purified according to a protocol by Scott et al.[^21^, ^31^, ^32^] Cu(OAc)_2_ **⋅**H_2_O (10 g) was dried by refluxing in Ac_2_O (50 mL) overnight, the salt was filtered under N_2_ and washed with anhydrous THF and dried under vacuum. CuCl (15 g) was washed with HCl aq. (1 m, until the green color disappeared), H_2_O, EtOH, and THF are activated by heating under reduced pressure (170 °C, 14 h, 0.1 mbar). Pyridine (600 mL) was refluxed over CaH_2_ for 18 h before distillation. A 1 L round bottom flask with baffles was filled with pyridine (470 mL) and purged with vacuum and argon cycles in the sonicator for 15 min. CuCl (253 mg, 2.48 mmol, 15 equiv.) and Cu(OAc)_2_ (629 mg, 3.47 mmol, 21 equiv.) were added in one portion (dark green solution), and the solution was purged again with vacuum and argon cycles in the sonicator for other 10 min. To this mixture, a solution of **38** (163 mg, 165 μmol, 1.0 equiv) in pyridine (18 mL, 5.6 cm) was added with a syringe‐pump over 9.5 h (with 0.1 mm min^−1^). One hour after full addition, the reaction was completed according to TLC (1 mL reaction mixture was evaporated and mixed with HCl 1 m (1 mL) and EtOAc (1 mL)). The pyridine was removed under reduced pressure, and the green residue was dissolved in DCM (200 mL). The yellow organic phase was washed with HCl (1 m, 200 mL, light blue) and brine (100 mL), dried over Na_2_SO_4_. The organic phase was concentrated under reduced pressure and subjected to column chromatography (100 g SiO_2_, Cy:EtOAc 97:3→72:28). Compound **39** (153 mg, 155 μmol, 94 %) was obtained as a yellow glassy solid. *R*_f_=0.28 (Cy/EtOAc, 5:1). ^1^H NMR (500 MHz, C_6_D_6_): *δ*=8.10 (t, *J=*1.6, H_Ar_, 1 H), 7.89 (ddd, *J=*7.8, 1.9, 1.1, H_Ar_, 1 H), 7.62 (dd, *J=*1.7, 0.6, H_Ar_, 1 H), 7.55 (dt, *J=*7.7, 1.3, H_Ar_, 1 H), 7.51–7.48 (m, H_Ar_, 1 H), 7.46 (dt, *J=*8.1, 1.0, H_Ar_, 1 H), 7.45 (d, *J=*2.0, H_Ar_, 1 H), 7.40 (ddd, *J=*7.8, 1.4, 0.6, H_Ar_, 1 H), 7.37 (dd, *J=*8.0, 1.9, H_Ar_, 1 H), 7.23 (t, *J=*7.8, H_Ar_, 1 H), 7.20–7.17 (m, H_Ar_, 1 H), 7.09 (d, *J=*8.0, H_Ar_, 1 H), 7.07–7.05 (m, H_Ar_, 1 H), 7.04–6.99 (m, H_Ar_, 3 H), 6.96 (dd, *J=*8.1, 1.6, H_Ar_, 1 H), 6.90 (td, *J=*7.6, 1.4, H_Ar_, 1 H), 6.85 (dd, *J=*8.0, 0.6, H_Ar_, 1 H), 6.82 (td, *J=*7.6, 1.3, H_Ar_, 1 H), 6.76 (ddd, *J=*7.8, 1.8, 1.0, H_Ar_, 1 H), 6.62 (td, *J=*7.7, 0.6, H_Ar_, 1 H), 1.59–1.46 (m, 3×*x* CH_2_, 6 H), 1.44–1.36 (m, CH_2_, 2 H), 1.11 (d, *J=*7.3, 2× CH_3_, 6 H), 1.08 (d, *J=*7.3, 2× CH_3_, 6 H), 1.05 (d, *J=*7.3, 2 *x* CH_3_, 6 H), 1.02 (d, *J=*7.3, 2× CH_3_, 6 H), 0.99–0.86 (m, 4× CH, 4 H), 0.63–0.57 (m, CH_2_, 2 H), 0.57–0.51 ppm (m, CH_2_, 2 H). ^13^C NMR (126 MHz, C_6_D_6_): *δ*=146.4 (1C, C_Ar_), 145.0 (1C, C_Ar_), 144.1 (1C, C_Ar_), 141.5 (1C, C_Ar_), 141.0 (1C, C_Ar_), 134.7 (1C, C_Ar_), 133.5 (1C, C_Ar_), 132.9 (1C, C_Ar_), 132.5 (1C, C_Ar_), 132.0 (1C, C_Ar_), 131.6 (1C, C_Ar_), 131.6 (1C, C_Ar_), 131.0 (1C, C_Ar_), 130.6 (1C, C_Ar_), 130.4 (1C, C_Ar_), 130.2 (1C, C_Ar_), 129.8 (1C, C_Ar_), 129.5 (1C, C_Ar_), 129.2 (1C, C_Ar_), 129.0 (1C, C_Ar_), 128.8 (1C, C_Ar_), 128.6 (1C, C_Ar_), 128.5 (1C, C_Ar_), 128.0 (1C, C_Ar_), 127.3 (1C, C_Ar_), 126.9 (1C, C_Ar_), 125.9 (1C, C_Ar_), 125.6 (1C, C_Ar_), 124.8 (1C, C_Ar_), 124.0 (1C, C_Ar_), 123.5 (1C, C_Ar_), 123.0 (1C, C_Ar_), 122.9 (1C, C_Ar_), 121.7 (1C, C_Ar_), 119.4 (1C, CN), 119.3 (1C, CN), 106.9 (1C, C_alkyne_), 106.9 (1C, C_alkyne_), 95.1 (1C, C_alkyne_), 95.0 (1C, C_alkyne_), 94.4 (1C, C_alkyne_), 94.2 (1C, C_alkyne_), 94.1 (1C, C_alkyne_), 93.3 (1C, C_alkyne_), 90.9 (1C, C_alkyne_), 90.0 (1C, C_alkyne_), 88.9 (1C, C_alkyne_), 87.1 (1C, C_alkyne_), 82.1 (1C, C_alkyne_), 79.0 (1C, C_alkyne_), 21.6 (1C, CH_2_), 21.4 (1C, CH_2_), 20.4 (1C, CH_2_), 20.4 (1C, CH_2_), 18.5 (2C, CH_3_), 18.4 (2C, CH_3_), 18.2 (2C, CH_3_), 18.1 (2C, CH_3_), 12.1 (2C, CH), 12.0 (2C, CH), 9.6 (1C, CH_2_), 9.6 ppm (1C, CH_2_). The peaks of 128.1 and 128.2 are only visible in the DEPT‐135 experiment, as the signals are overlain by C_6_D_6_. HR‐MS (ESI, MeOH) calcd for C_70_H_62_N_2_NaSi_2_^+^: [*M*+Na]^+^, 1009.4344; found [*M*+Na]^+^, 1009.4335.

**Macrocycle 40**: A two‐neck round‐bottom flask was cleaned by the following treatment to remove all copper‐ions from previous reactions. The flask was filled with conc. H_2_SO_4_ and sonicated for 10 min, followed by washing with H_2_O, NaOH (1 m), H_2_O, and acetone. The flask was dried in the heating oven overnight and flushed with argon. To a solution of **39** (150 mg, 152 μmol, 1.0 equiv) in THF (70 mL) was added TBAF (1 m in THF, 304 μL, 304 μmol, 2.0 equiv) and the reaction mixture stirred at room temperature for 30 min. under argon. The reaction was diluted by the addition of water (150 mL), and the aqueous phase was extracted with CH_2_Cl_2_ (250 mL). The organic layer was washed with water (150 mL) and brine (150 mL) and dried over Na_2_SO_4_. The organic phase was concentrated under reduced pressure and subjected to column chromatography (100 g SiO_2_, Cy:EtOAc 98:2→86:14). Compound **40** (25 mg, 40.0 μmol, 26 %) was obtained as a yellow oil. *R*_f_=0.27 (Cy/EtOAc, 10:1). ^1^H NMR (500 MHz, C_6_D_6_): *δ*=8.15 (t, *J=*1.8, H_Ar_, 1 H), 7.77 (ddd, *J=*7.8, 1.8, 1.1, H_Ar_, 1 H), 7.53–7.48 (m, H_Ar_, 3 H), 7.46–7.43 (m, H_Ar_, 2 H), 7.39–7.33 (m, H_Ar_, 2 H), 7.15–7.14 (m, H_Ar_, 1 H), 7.11 (t, *J=*7.7, H_Ar_, 1 H), 7.05–6.98 (m, H_Ar_, 5 H), 6.88–6.83 (m, H_Ar_, 2 H), 6.80–6.75 (m, H_Ar_, 2 H), 6.73 (dd, *J=*8.0, 0.5, H_Ar_, 1 H), 6.65–6.60 (m, H_Ar_, 1 H), 2.99 (s, H_alkyne_, 1 H), 2.65 ppm (s, H_alkyne_, 1 H). ^13^C NMR (126 MHz, C_6_D_6_): *δ*=146.1 (1C, C_Ar_), 144.6 (1C, C_Ar_), 144.0 (1C, C_Ar_), 140.9 (1C, C_Ar_), 140.5 (1C, C_Ar_), 134.2 (1C, C_Ar_), 133.2 (1C, C_Ar_), 132.4 (1C, C_Ar_), 131.9 (1C, C_Ar_), 131.6 (1C, C_Ar_), 131.3 (1C, C_Ar_), 131.1 (1C, C_Ar_), 130.9 (1C, C_Ar_), 130.0 (1C, C_Ar_), 129.9 (1C, C_Ar_), 129.8 (1C, C_Ar_), 129.3 (1C, C_Ar_), 129.2 (1C, C_Ar_), 128.8 (1C, C_Ar_), 128.5 (1C, C_Ar_), 128.4 (1C, C_Ar_), 128.2 (1C, C_Ar_), 127.0 (1C, C_Ar_), 126.8 (1C, C_Ar_), 125.3 (1C, C_Ar_), 125.0 (1C, C_Ar_), 124.4 (1C, C_Ar_), 123.1 (1C, C_Ar_), 122.9 (1C, C_Ar_), 122.7 (1C, C_Ar_), 122.6 (1C, C_Ar_), 121.4 (1C, C_Ar_), 94.8 (1C, C_alkyne_), 94.7 (1C, C_alkyne_), 94.1 (1C, C_alkyne_), 94.0 (1C, C_alkyne_), 90.7 (1C, C_alkyne_), 89.2 (1C, C_alkyne_), 88.2 (1C, C_alkyne_), 86.5 (1C, C_alkyne_), 82.2 (1C, C_alkyne_), 82.2 (1C, C_alkyne_), 81.6 (1C, C_alkyne_), 81.5 (1C, C_alkyne_), 80.1 (1C, C_alkyne_), 78.8 ppm (1C, C_alkyne_). The peaks of 128.0, 127.7, 127.6 and 127.5 are only visible in the DEPT‐135 experiment, as the signals are overlain by C_6_D_6_. HR‐MS (ESI, MeOH) calcd for C_50_H_22_Na^+^: [*M*+Na]^+^, 647.1770; found [*M*+Na]^+^, 647.1764.

**Bicycle 1**: CuCl, Cu(OAc)_2,_ and pyridine were purified according to a protocol by Scott et al.[^21^, ^31^, ^32^] Cu(OAc)_2_ **⋅**H_2_O (10 g) was dried by refluxing in Ac_2_O (50 mL) overnight, the salt was filtered under N_2_ and washed with anhydrous THF and dried under vacuum. CuCl (15 g) was washed with HCl aq. (1 m, until the green color disappeared), H_2_O, EtOH, and THF an activated by heating under reduced pressure (170 °C, 14 h, 0.1 mbar). Pyridine (600 mL) was refluxed over CaH_2_ for 18 h before distillation. A 1 L round bottom flask with baffles was filled with pyridine (95 mL) and purged with vacuum and argon cycles in the sonicator for 15 min. CuCl (49 mg, 480 μmol, 15 equiv) and Cu(OAc)_2_ (122 mg, 672 μmol, 21 equiv.) were added in one portion (dark green solution), and the solution was purged again with vacuum and argon cycles in the sonicator for other 10 min. To this mixture a solution of **40** (20.0 mg, 32.0 μmol, 1.0 equiv) in pyridine (3.5 mL, 2.8 cm) was added with a syringe‐pump over 2.0 h (with 0.23 mm min^−1^). One hour after full addition, the reaction was completed according to TLC (1 mL reaction mixture was evaporated and mixed with HCl 1 m (1 mL) and EtOAc (1 mL)). The pyridine was removed under reduced pressure, and the green residue was dissolved in DCM (50 mL). The yellow organic phase was washed with HCl (1 m, 50 mL, light blue) and brine (50 mL), dried over Na_2_SO_4_. The organic phase was concentrated under reduced pressure and subjected to column chromatography (10 g SiO_2_, Cy:EtOAc 98:2→82:18). Compound **1** (17 mg, 27.0 μmol, 85 %) was obtained as a brown solid. *R*_f_=0.33 (Cy/EtOAc, 10:1). ^1^H NMR (600 MHz, C_6_D_6_): *δ*=8.42 (td, *J=*1.7, 0.6, H_Ar_, H_28_, 1 H), 8.15 (dd, *J=*1.8, 0.6, H_Ar_, H_5_, 1 H), 7.67 (ddd, *J=*7.6, 1.5, 0.5, H_Ar_, H_11_, 1 H), 7.58 (ddd, *J=*7.6, 1.7, 1.2, H_Ar_, H_33_, 1 H), 7.38 (ddd, *J=*7.7, 1.3, 0.6, H_Ar_, H_46_, 1 H), 7.31 (td, *J=*1.7, 0.6, H_Ar_, H_37_, 1 H), 7.18 (ddd, *J=*7.8, 1.8, 1.1, H_Ar_, H_35_,1 H), 7.16 (ddd, *J=*7.5, 1.4, 0.4, H_Ar_, H_8_,1 H), 7.13–7.11 (m, H_Ar_, H_43_ and H_24_, 2 H), 7.10, (dd, *J=*7.9, 1.8, H_Ar_, H_3_, 1 H), 7.09 (td, *J=*7.5, 1.5, H_Ar_, H_9_, 1 H), 7.07 (dd, *J=*7.9, 0.5, H_Ar_, H_2_, 1 H), 7.04 (td, *J=*7.5, 1.4, H_Ar_, H_10_, 1 H), 6.81 (td, *J=*7.6, 1.3, H_Ar_, H_45_, 1 H), 6.77 (ddd, *J=*7.7, 1.7, 1.1, H_Ar_, H_26_, 1 H), 6.75 (td, *J=*7.7, 0.4, H_Ar_, H_34_, 1 H), 6.74 (td, *J=*7.7, 1.3, H_Ar_, H_44_, 1 H), 6.73 (dd, *J=*1.7, 0.5, H_Ar_, H_23_, 1 H), 6.69 (td, *J=*7.7, 0.6, H_Ar_, H_25_, 1 H), 6.66 (dd, *J=*8.0, 0.6, H_Ar_, H_20_, 1 H), 6.54 ppm (dd, *J=*8.0, 1.7, H_Ar_, H_21_, 1 H). ^13^C NMR (126 MHz, C_6_D_6_): *δ*=147.4 (1C, C_Ar_, C_1_), 145.2 (1C, C_Ar_, C_23_), 144.5 (1C, C_Ar_, C_28_), 142.8 (1C, C_Ar_, C_7_), 142.1 (1C, C_Ar_, C_36_), 141.4 (1C, C_Ar_, C_4_), 134.8 (1C, C_Ar_, C_11_), 132.9 (1C, C_Ar_, C_33_), 132.0 (1C, C_Ar_, C_8_), 131.9 (1C, C_Ar_, C_17_), 131.5 (1C, C_Ar_, C_3_), 131.5 (1C, C_Ar_, C_35_), 131.4 (1C, C_Ar_, C_46_), 131.2 (1C, C_Ar_, C_37_), 131.1 (1C, C_Ar_, C_41_), 130.3 (1C, C_Ar_, C_2_), 130.2 (1C, C_Ar_, C_24_), 130.1 (1C, C_Ar_, C_5_), 130.0 (1C, C_Ar_, C_43_), 129.6 (1C, C_Ar_, C_45_), 129.6 (1C, C_Ar_, C_9_), 129.4 (1C, C_Ar_, C_25_), 129.2 (1C, C_Ar_, C_20_), 128.1 (1C, C_Ar_, C_10_), 127.6 (1C, C_Ar_, C_21_), 127.0 (1C, C_Ar_, C_26_), 126.3 (1C, C_Ar_, C_42_), 125.0 (1C, C_Ar_, C_18_), 124.1 (1C, C_Ar_, C_38_), 123.5 (1C, C_Ar_, C_22_), 123.5 (1C, C_Ar_, C_27_), 122.9 (1C, C_Ar_, C_6_), 122.1 (1C, C_Ar_, C_19_), 121.5 (1C, C_Ar_, C_12_), 98.6 (1C, C_alkyne_, C_13_), 96.2 (1C, C_alkyne_, C_39_), 95.7 (1C, C_alkyne_, C_15_), 94.2 (1C, C_alkyne_, C_30_), 92.7 (1C, C_alkyne_, C_16_), 91.9 (1C, C_alkyne_, C_14_), 90.5 (1C, C_alkyne_, C_29_), 88.9 (1C, C_alkyne_, C_40_), 88.4 (1C, C_alkyne_, C_47_), 87.5 (1C, C_alkyne_, C_48_), 82.8 (1C, C_alkyne_, C_50_), 81.9 (1C, C_alkyne_, C_32_), 81.5 (1C, C_alkyne_, C_31_), 80.6 ppm (1C, C_alkyne_, C_49_). The peaks of 128.6 (1C, C_alkyne_, C_44_) and 128.5 (1C, C_alkyne_, C_34_) are only visible in the DEPT‐135 experiment, as the signals are overlain by C_6_D_6_. The inner diacetylene peaks (C_31_, C_32_, C_49_ and C_50_) cannot be assigned from the 2D‐NMRs, but were assigned according to the calculations (see Supporting Information). HR‐MS (ESI, MeOH) calcd for C_50_H_22_Na^+^: [*M*+Na]^+^, 645.1614; found [*M*+Na]^+^, 645.1604. HR‐MS (MALDI‐TOF, DCTB Matrix+HP‐Mix (pos)): calcd for C_50_H_22_ [*M*]^+^ 622.1716; found 622.1717 (3.3).

## Conflict of interest

The authors declare no conflict of interest.

## Supporting information

As a service to our authors and readers, this journal provides supporting information supplied by the authors. Such materials are peer reviewed and may be re‐organized for online delivery, but are not copy‐edited or typeset. Technical support issues arising from supporting information (other than missing files) should be addressed to the authors.

Supplementary
