# Supplementary material for: Bicyclic Phenyl–Ethynyl Architectures: Synthesis of a 1,4‐Bis(phenylbuta‐1,3‐diyn‐1‐yl) Benzene Banister
Source: Chemistry. 2021 Mar 3;27(20):6295–307. doi: 10.1002/chem.202005207 (PMC8048618; doi:10.1002/chem.202005207)
Supplement: Supplementary file 1 — Supplementary [file CHEM-27-6295-s001.pdf]

Chemistry–A European Journal

Supporting Information

Bicyclic Phenyl–Ethynyl Architectures: Synthesis of a 1,4-Bis(phenylbuta-1,3-diyn-1-yl) Benzene Banister

Linda Maria Bannwart,[a] Thomas Müntener,[b] Michel Rickhaus,[c] Lukas Jundt,[a] Daniel Häussinger,[a] and Marcel Mayor*[a, d, e]

Table of Contents

1. Organization of the Supporting Information 3

2. Experimental Section 3

3. Further Information on Compound **1** and Compound **41** 12

3.1 Measured NMR Signals of Compound **1** 12

3.2 Calculated NMR Signals of Compound **1** 13

3.3 NOEs comparison with DFT structures 15

3.4 DOSY of Compound **1** and Compound **41** 17

3.5 HMBC of Compound **41** 18

3.6 Three Possible Structures of Compound **41** 18

3.7 HOMO and LUMO of Compound **1a** and **1b** 19

4. Dynamic HPLC 23

4.1 Data evaluation with DCXplorer 24

5. ^{1}H-, ^{13}C-, DEPT-135 NMR and HR-MS 28

5.1 Compound **5** 28

5.2 Compound **6** 29

5.3 Compound **7** 34

5.4 Compound **8** 39

5.5 Compound **9** 43

5.6 Compound **10** 48

5.7 Compound **12** 52

5.8 Compound **13** 53

5.9 Compound **14** 54

5.10 Compound **15** 59

5.11 Compound **16** 64

5.12 Compound **E** 69

5.13 Compound **17** 70

5.14 Compound **18** 77

5.15 Compound $\mathbf{C_1}$ 83

5.16 Compound **21** 84

5.17 Compound **22** 89

5.18 Compound **23** 94

5.19 Compound **24** 99

5.20 Compound **25** 104

5.21 Compound **26** 109

5.22 Compound **27** 114

5.23 Compound **28** 119

5.24 Compound **29** 124

5.25 Compound **31** ... 129
5.26 Compound **32** ... 134
5.27 Compound **33** ... 139
5.28 Compound **C_2** ... 144
5.29 Compound **34** ... 149
5.30 Compound **35** ... 155
5.31 Compound **36** ... 161
5.32 Compound **37** ... 166
5.33 Compound **38** ... 172
5.34 Compound **39** ... 178
5.35 Compound **40** ... 184
5.36 Compound **1** ... 190
5.37 Compound **41** ... 201
5.38 List of DEPT-135 Signals ... 206
6. Summary Structure Calculation ... 208
6.1 Calculation with Spartan ('18 V 1.2.0, DFT, B3LYP/6-31G*) ... 208
6.1.1 Compound 1a ... 208
6.1.2 Compound 1b ... 282
6.2 Calculation with ORCA (B3LYP RIJCOSX D3BJ def2-TZVP def2/J TIGHTSCF Opt NumFreq Grid5 FinalGrid6) Program Version 4.1.2 ... 294
7.Literature ... 305

1. Organization of the Supporting Information

The order of the items in the supporting information file follows the order of their discussion in the main manuscript.

2. Experimental Section

2-Bromo-4-iodoaniline (5)

To 2-bromoaniline (**4**, 5.00 g, 29.1 mmol, 1.0 eq) in DMSO (250 mL) was added NIS (7.09 g, 30.6 mmol, 1.05 eq.) and the mixture stirred at room temperature for 18 h. After the reaction was completed according to TLC and GC–MS, the mixture was cooled to 5°C and quenched with aqueous $NaHCO_3$ (500 mL) (exothermic for the first 100 mL), and the brown suspension was filtered off and washed with water (100 mL). The filtrate was dried under reduced pressure and subjected to column chromatography (75 g SiO_2, Cy:EtOAc 91:9). Compound **5** (8.50 g, 28.5 mmol, 98 %) was obtained as brown solid.

R_f = 0.18 (Cy/EtOAc, 10:1).

^{1}H NMR (500 MHz, CD_2Cl_2) δ = 7.60 (d, *J*=2.0, H_{Ar}, 1H), 7.28 (dd, *J*=8.4, 2.0, H_{Ar}, 1H), 6.47 (d, *J*=8.4, H_{Ar}, 1H), 4.01 (s, NH_2, 2H).

The spectra data of this compound was identical to those reported in the literature.[1]

(E)- 1- ((2- Bromo- 4- iodophenyl) diazenyl) pyrrolidine (6)

A two-neck round-bottom flask was dried in the heating oven overnight and flushed with argon. **5** (6.0 g, 20.1 mmol, 1.0 eq.) was dissolved in a mixture of MeCN (20 mL) and water (10 mL) and then cooled to 0 °C. To this solution conc. HCl (6.66 mL, 80.4 mmol, 4.0 eq.) was added dropwise at 0°C. This solution was cooled to -5°C, and a solution of $NaNO_2$ (2.12 mg, 30.2 mmol, 1.5 eq.) in water (3 mL) was added in such a way that the reaction mixture maintained below 0°C. After the full addition, the reaction mixture was stirred for 30 min. at 0°C. This mixture was added slowly to a stirred solution of pyrrolidine (4.13 mL, 50.3 mmol, 2.5 eq.) and K_2CO_3 (14.4 g, 105 mmol, 5.2 eq.) in MeCN (80 mL) and water (40 mL) at 0°C. The reaction mixture was stirred for 1 h at 5 °C, then allowed to warm to room temperature and stirred at this temperature for 1 h. After the reaction was completed according to TLC, the reaction mixture was extracted with Et_2O (3 x150 mL), and the combined organic extracts were washed with brine (150 mL) and dried over Na_2SO_4. The organic phase was concentrated under reduced pressure and subjected to column chromatography (350 g SiO_2, Cy:EtOAc 100:0 -> 91:9). Compound **6** (6.88 g, 18.1 mmol, 90 %) was obtained as a red-brown solid.

R_f = 0.32 (Cy/EtOAc, 20:1).

Mp =75 °C

^{1}H NMR (500 MHz, CD_2Cl_2) δ = 7.89 (d, *J*=2.0, H_{Ar}, 1H), 7.53 (dd, *J*=8.5, 2.0, H_{Ar}, 1H), 7.17 (d, *J*=8.6, H_{Ar}, 1H), 3.93 (t, *J*=6.6, CH_2, 2H), 3.67 (t, *J*=6.9, CH_2, 2H), 2.11 – 1.97 (m, 2 x CH_2, 4H).

^{13}C NMR (126 MHz, CD_2Cl_2) δ = 149.2 (1C, C_{Ar}), 141.3 (1C, C_{Ar}), 137.3 (1C, C_{Ar}), 120.6 (1C, C_{Ar}), 120.4 (1C, C_{Ar}), 88.6 (1C, C_{Ar}), 51.9 (1C, CH_2), 47.6 (1C, CH_2), 24.5 (1C, CH_2), 24.0 (1C, CH_2).

HR-MS (ESI, MeOH) calcd. For $C_{10}H_{12}BrIN_3^+$: [M + H]$^+$, 379.9254; found [M + H]$^+$, 379.9257.

(E)- 1- ((3- Bromo- 2'- chloro- [1,1'- biphenyl]- 4-yl) diazenyl) pyrrolidine (7)

An oven-dried and argon flushed Schlenk tube was charged with **6** (5.74 g, 15.1 mmol, 1.00 eq), 2-chlorophenylboronic acid (2.59 g, 16.6 mmol, 1.1 eq) and K_2CO_3 (6.32 g, 45.3 mmol, 3.0 eq) and placed under vacuum for 5 min. Then dry toluene (57 mL) and EtOH (14 mL) were added, and the mixture degassed by passing argon through fur further 5 min. $(Ph_3P)_2PdCl_2$ (214 mg, 302 µmol, 0.02 eq) was added, and the mixture was heated to 80 °C for 18 h. After the reaction was completed according to TLC, the reaction mixture was diluted with DCM (550 mL) and washed with H_2O (250 mL) and brine (250 mL), and dried over Na_2SO_4. The organic phase was concentrated under reduced pressure and subjected to column chromatography (700 g SiO_2, pentane:Tol 80:20). Compound **7** (4.76 g, 13.1 mmol, 87%) was obtained as yellow solid.

R_f = 0.28 (Cy/EtOAc, 20:1).

^{1}H NMR (500 MHz, CD_2Cl_2) δ = 7.67 (d, *J*=1.9, H_{Ar}, 1H), 7.49 – 7.46 (m, H_{Ar}, 2H), 7.38 – 7.27 (m, H_{Ar}, 4H), 3.96 (t, *J*=6.6, CH_2, 2H), 3.72 (t, *J*=6.9, CH_2, 2H), 2.12 – 2.00 (m, 2 x CH_2, 4H).

^{13}C NMR (126 MHz, CD_2Cl_2) δ = 148.7 (1C, C_{Ar}), 139.6 (1C, C_{Ar}), 137.5 (1C, C_{Ar}), 134.3 (1C, C_{Ar}), 132.9 (1C, C_{Ar}), 131.8 (1C, C_{Ar}), 130.5 (1C, C_{Ar}), 129.7 (1C, C_{Ar}), 129.3 (1C, C_{Ar}), 127.5 (1C, C_{Ar}), 119.2 (1C, C_{Ar}), 118.4 (1C, C_{Ar}), 51.8 (1C, CH_2), 47.5 (1C, CH_2), 24.5 (1C, CH_2), 24.1 (1C, CH_2).

HR-MS (ESI, MeOH) calcd. for $C_{16}H_{16}BrClN_3^+$: [M + H]$^+$, 364.0211; found [M + H]$^+$, 364.0211.

3'-bromo-2-chloro-4'-iodo-1,1'-biphenyl (8)

Four 25 mL Microwave tubes were charged with each a quarter of **7** (3.247 g, 8.90 mmol, 1.0 eq) and I_2 (2.26 g, 8.90 mmol, 1.0 eq.) in MeCN (65 mL) and degassed by bubbling argon through and then sealed and stirred at 100 °C for 4 h. After the reaction was completed according to TLC, the brown-black solutions were combined and dissolved in DCM (250 mL), and the organic phase was washed with sat. $Na_2S_2O_3$ (250 mL). The aqueous phase was extracted again with DCM (250 mL), and the combined organic phases were washed with brine (250 mL) and dried over Na_2SO_4. The organic phase was concentrated under reduced pressure and subjected to column chromatography (600 g SiO_2, Cy 100%). Compound **8** (2.84 g, 7.22 mmol, 81%) was obtained as white solid.

R_f = 0.43 (Cy 100%).

Mp = 88 °C.

^{1}H NMR (500 MHz, CD_2Cl_2) δ = 7.94 (d, *J*=8.2, H_{Ar}, 1H), 7.72 (d, *J*=2.0, H_{Ar}, 1H), 7.50 – 7.47 (m, H_{Ar}, 1H), 7.37 – 7.30 (m, H_{Ar}, 3H), 7.11 (dd, *J*=8.0, 2.0, H_{Ar}, 1H).

^{13}C NMR (126 MHz, CD_2Cl_2) δ = 141.6 (1C, C_{Ar}), 140.5 (1C, C_{Ar}), 138.6 (1C, C_{Ar}), 134.0 (1C, C_{Ar}), 132.7 (1C, C_{Ar}), 131.6 (1C, C_{Ar}), 130.6 (1C, C_{Ar}), 130.3 (1C, C_{Ar}), 130.0 (1C, C_{Ar}), 129.8 (1C, C_{Ar}), 127.7 (1C, C_{Ar}), 100.6 (1C, C_{Ar}).

HR-MS (EI, 70 eV): calcd. for $C_{12}H_7BrClI$ $[M]^+$ 391.8459, found 391.8454.

3'-Bromo-3''-iodo-[1,1':4',1''-terphenyl]-2-amine 2'-bromo-2''-chloro-[1,1':4',1''-terphenyl]-3-amine (9)

To an oven-dried and argon flushed Schlenk tube was consecutively added potassium carbonate (2.26 mg, 16.2 mmol, 3.0 equiv.) and 3-aminophenylboronic acid monohydrate (939 mg, 5.94 mmol, 1.1 eq) and placed under vacuum for 5 min. **8** (2.13 g, 5.40 mmol, 1.0 eq) in dry toluene (40 mL) and dry EtOH (10 mL) was added and the solution degassed for 10 min before adding $PdCl_2(PPh_3)_2$ (115 mg, 162 µmol, 3 mol%). The reaction mixture was heated to 80 °C for 3.5 h. After the reaction was completed according to TLC, then the reaction mixture was cooled to room temperature, the reaction mixture was diluted with EtOAc (250 mL) and washed with H_2O (200 mL) and brine (200 mL), and dried over Na_2SO_4. The organic phase was concentrated under reduced pressure and subjected to column chromatography (600 g neutral SiO_2, Cy:EtOAc 83:17). Compound **9** (1.72 g, 4.80 mmol, 89%) was obtained as a yellow oil.

R_f = 0.19 (Cy/EtOAc, 4:1).

^{1}H NMR (500 MHz, CD_2Cl_2) δ = 7.76 (d, *J*=1.8, H_{Ar}, 1H), 7.53 – 7.49 (m, H_{Ar}, 1H), 7.45 (dd, *J*=7.9, 1.8, H_{Ar}, 1H), 7.42 – 7.32 (m, H_{Ar}, 4H), 7.23 (t, *J*=7.9, H_{Ar}, 1H), 6.82 (ddd, *J*=7.6, 1.7, 1.0, H_{Ar}, 1H), 6.77 (td, *J*=1.7, 0.8, H_{Ar}, 1H), 6.73 (ddd, *J*=8.1, 2.4, 1.0, H_{Ar}, 1H), 3.84 (s, NH_2, 2H).

^{13}C NMR (126 MHz, CD_2Cl_2) δ = 147.0 (1C, C_{Ar}), 142.5 (1C, C_{Ar}), 142.3 (1C, C_{Ar}), 140.4 (1C, C_{Ar}), 139.2 (1C, C_{Ar}), 134.3 (1C, C_{Ar}), 132.8 (1C, C_{Ar}), 131.9 (1C, C_{Ar}), 131.3 (1C, C_{Ar}), 130.6 (1C, C_{Ar}), 129.7 (1C, C_{Ar}), 129.5 (1C, C_{Ar}), 129.1 (1C, C_{Ar}), 127.7 (1C, C_{Ar}), 122.4 (1C, C_{Ar}), 120.0 (1C, C_{Ar}), 116.4 (1C, C_{Ar}), 114.8 (1C, C_{Ar}).

HR-MS (ESI, MeOH) calcd. for $C_{18}H_{14}BrClN^+$: $[M + H]^+$, 357.9993; found $[M + H]^+$, 357.9992.

3'-bromo-2-chloro-3''-iodo-1,1':4',1''-terphenyl (10)

The reaction only works well in scales below a gram; otherwise, the yield is drastically reduced. **9** (642 mg, 1.43 mmol, 1.0 eq.) in MeCN (4.0 mL) was added at room temperature to a stirred solution of *p*TsOH×H_2O (828 mg, 4.29 mmol, 3.0 eq.) in MeCN (12 mL), and the resulting suspension was cooled to 10 °C. Afterward, a solution of $NaNO_2$ (201 mg, 2.86 mmol, 2.0 eq.) and KI (599 mg, 3.58 mmol, 2.5 eq.) in water (4.0 mL) was added drop-wise over 5 min (gas evolution). After additional stirring for 10 min. at 10 °C, the solution was allowed to reach room temperature and stirred for another 16 h. After the reaction was completed according to TLC, the reaction was quenched with a mixture of H_2O (20 mL), sat. aq. $NaHCO_3$-sol. (12 mL) and sat. aq. $Na_2S_2O_3$-sol. (12 mL). The aqueous phase was extracted with EtOAc (2 x 20 mL), and the combined organic extracts were dried over Na_2SO_4. The organic phase was concentrated under reduced pressure and subjected to column chromatography (160 g SiO_2, pentane 100%). Compound **10** (617 mg, 1.314 mmol, 92 %) was obtained as white solid.

R_f = 0.33 (Cy 100%).

Mp= 117 °C.

^{1}H NMR (500 MHz, CD_2Cl_2) δ = 7.84 (t, *J*=1.7, H_{Ar}, 1H), 7.79 – 7.75 (m, H_{Ar}, 2H), 7.53 – 7.50 (m, H_{Ar}, 1H), 7.49 – 7.46 (m, H_{Ar}, 2H), 7.40 – 7.33 (m, H_{Ar}, 4H), 7.22 (t, *J*=7.8, H_{Ar}, 1H).

^{13}C NMR (126 MHz, CD_2Cl_2) δ = 143.3 (1C, C_{Ar}), 141.1 (1C, C_{Ar}), 140.6 (1C, C_{Ar}), 139.0 (1C, C_{Ar}), 138.8 (1C, C_{Ar}), 137.3 (1C, C_{Ar}), 134.5 (1C, C_{Ar}), 132.8 (1C, C_{Ar}), 131.8 (1C, C_{Ar}), 131.2 (1C, C_{Ar}), 130.6 (1C, C_{Ar}), 130.3 (1C, C_{Ar}), 129.8 (1C, C_{Ar}), 129.4 (1C, C_{Ar}), 129.3 (1C, C_{Ar}), 127.7 (1C, C_{Ar}), 122.3 (1C, C_{Ar}), 94.1 (1C, C_{Ar}).

HR-MS (EI, 70 eV): calcd. for $C_{18}H_{11}BrClI$ $[M]^+$ 467.8772, found 467.8763.

2,5-Dibromoaniline (12)

A four-necked sulfonation flask was equipped with a reflux condenser, a thermometer, and a KGB stirrer. 1,4-dibromo-2-nitrobenzol (**11**, 5.00 g, 17.8 mmol, 1.0 eq) and iron (10.0 g, 178 mmol, 10.0 eq) were suspended in EtOH (125 mL) and HCl (10 mL, 37 %) was added dropwise. The reaction mixture was stirred at 50°C for 4 h. After the reaction was completed according to GC-MS and TLC, Celite was added to the red reaction mixture and stirred for 10 min. and filtered through a Celite pad and washed with EtOAc. The mixture was concentrated under reduced pressure and diluted with EtOAc (250 mL), and washed with H_2O (250 mL). The aqueous phase was extracted again with EtOAc (125 mL), and the combined organic phases were washed with $NaHCO_3$ (2x 125 mL) and brine (125 mL). After drying over Na_2SO_4, the solvent was concentrated under reduced pressure. Compound **12** (4.45 g, 17.7 mmol, quant.) was obtained as brown solid.

R_f = 0.26 (Cy/EtOAc, 10:1)

^{1}H NMR (400 MHz, $CDCl_3$) δ = 7.23 (d, *J*=8.4, H_{Ar}, 1H), 6.89 (d, *J*=2.2, H_{Ar}, 1H), 6.73 (dd, *J*=8.5, 2.2, H_{Ar}, 1H), 4.06 (s, NH_2, 2H). The spectra data of this compound was identical to those reported in the literature.[2]

1,4-Dibromo-2-iodobenzene (13)

12 (4.45 g, 17.7 mmol, 1.0 eq.) was added at room temperature to a stirred solution of *p*TsOH× H_2O (17.1 g, 88.5 mmol, 5.0 eq.) in MeCN (180 mL), and the resulting suspension was cooled to 10 °C. Afterward, a solution of $NaNO_2$ (2.49 g, 35.4 mmol, 2.0 eq.) and KI (7.42 g, 44.3 mmol, 2.5 eq.) in water (22 mL) was added dropwise over 15 min (gas evolution) and stirred for another 10 min. at 10 °C. Then the solution was allowed to reach room temperature and stirred for another 1 h. After the reaction was completed according to GC-MS and TLC, the reaction was quenched with a mixture of H_2O (240 mL), sat. aq. $NaHCO_3$-sol. (90 mL) and sat. aq. $Na_2S_2O_3$-sol. (60 mL). The aqueous phase was extracted with EtOAc (2 x 250 mL), and the combined organic phases were dried over Na_2SO_4. The mixture was concentrated under reduced pressure. Compound **13** (5.84 g, 16.1 mmol, 91 %) was obtained as a light brown solid.

R_f = 0.49 (Cy/EtOAc, 10:1)

^{1}H NMR (400 MHz, $CDCl_3$) δ = 7.99 (d, *J*=2.3, H_{Ar}, 1H), 7.46 (d, *J*=8.5, H_{Ar}, 1H), 7.32 (dd, *J*=8.5, 2.3, H_{Ar}, 1H). The spectra data of this compound was identical to those reported in the literature.[3]

4-(2,5-Dibromophenyl)-2-methylbut-3-yn-2-ol (14)

To an oven-dried and argon flushed Schlenk tube (50 mL) was charged with **13** (5.84 g, 16.1 mmol, 1.0 eq), dry THF (18 mL) and piperidine (6.0 mL), and the mixture degassed by passing argon through for 10 min. Then HOP-acetylene (1.8 mL, 17.7 mmol, 1.1 eq.) was added, and the reaction mixture was again bubbled for 5 min. $(Ph_3P)_2PdCl_2$ (571 mg, 805 µmol, 0.05 eq.) and CuI (188 mg, 966 µmol, 0.06 eq.) were added, the exothermic mixture was cooled with a water bath while degassing by passing argon through for a further 5 min. The yellow suspension was stirred at room temperature for 1 h until it was no longer exothermic. After the reaction was completed according to GC-MS and TLC, the reaction was dissolved in EtOAc (200 mL). The organic layer was washed with water (200 mL) and brine (200 mL) and dried over Na_2SO_4. The mixture was concentrated under reduced pressure and subjected to column chromatography. (600 g SiO_2, Cy:EtOAc 10:1). Compound **14** (3.89 g, 12.2 mmol, 76%) was obtained as an orange oil.

R_f = 0.11 (Cy/EtOAc, 10:1)

^{1}H NMR (400 MHz, $CDCl_3$) δ =7.57 (d, *J*=2.4, H_{Ar}, 1H), 7.42 (d, *J*=8.6, H_{Ar}, 1H), 7.29 – 7.26 (m, H_{Ar}, 1H), 2.08 (s, OH, 1H), 1.64 (s, 2 x CH_3, 6H).

^{13}C NMR (101 MHz, $CDCl_3$) δ = 135.8 (1C, C_{Ar}), 133.7 (1C, C_{Ar}), 132.6 (1C, C_{Ar}), 126.8 (1C, C_{Ar}), 124.5 (1C, C_{Ar}), 120.7 (1C, C_{Ar}), 99.9 (1C, $C_{alkyine}$), 79.9 (1C, $C_{alkyine}$), 65.9 (C, C_{tert}), 31.4 (2C, CH_3).

HR-MS (ESI, MeOH) calcd. For $C_{11}H_{10}Br_2NaO^+$: [M + Na]$^+$, 338.8991; found [M + Na]$^+$, 338.8990.

The provided reference does not report the corresponding experimental data.[4]

4,4’- (((2- (3- Hydroxy- 3- methylbut- 1- yn- 1- yl)- 1,4-phenylene) bis (ethyne- 2,1-diyl)) bis (diisopropylsilanediyl)) dibutanenitrile (15)

An oven-dried and argon flushed Schlenk tube was charged with **14** (2.00 g, 6.29 mmol, 1.0 eq), dry THF (20 mL), and piperidine (5 mL), and the mixture degassed by passing argon through for 10 min. Then CPDIPS-acetylene (3.5 mL, 15.1 mmol, 2.4 eq.) was added, and the reaction mixture was again bubbled for 5 min and then $(Ph_3P)_2PdCl_2$ (223 mg, 315 µmol, 0.05 eq.) and CuI (73.3 mg, 377 µmol, 0.06 eq.) were added. The yellow suspension was stirred at 60°C for 1 h. After the reaction was completed according to GC-MS and TLC, the reaction was dissolved in EtOAc (100 mL). The organic layer was washed with water (100 mL) and brine (100 mL) and dried over Na_2SO_4. The mixture was concentrated under reduced pressure and subjected to column chromatography. (600 g SiO_2, Cy:EtOAc 4:1). Compound **15** (3.47 g, 6.07 mmol, 97%) was obtained as a brown oil.

R_f = 0.14 (Cy/EtOAc, 4:1)

^{1}H NMR (500 MHz, CD_2Cl_2) δ = 7.54 (d, *J*=1.4, H_{Ar}, 1H), 7.43 (d, *J*=8.0, H_{Ar}, 1H), 7.35 (dd, *J*=8.1, 1.7, H_{Ar}, 1H), 2.43 (t, *J*=7.0, CH_2, 2H), 2.42 (t, *J*=7.0, CH_2, 2H), 1.91 – 1.81 (m, 2 x CH_2, 4H), 1.59 (s, 2 x CH_3, 6H), 1.16 – 1.06 (m, 4 x *i*Pr, 28H), 0.89 – 0.81 (m, 2 x CH_2, 4H).

^{13}C NMR (126 MHz, CD_2Cl_2) δ = 136.3 (1C, C_{Ar}), 133.5 (1C, C_{Ar}), 131.7 (1C, C_{Ar}), 126.0 (1C, C_{Ar}), 125.6 (1C, C_{Ar}), 123.8 (1C, C_{Ar}), 120.4 (1C, CN), 120.3 (1C, CN), 106.7 (1C, $C_{alkyine}$), 106.0 (1C, $C_{alkyine}$), 99.3 (1C, $C_{alkyine}$), 96.5 (1C, $C_{alkyine}$), 93.1 (1C, $C_{alkyine}$), 80.2 (1C, $C_{alkyine}$), 66.0 (C, C_{tert}), 31.8 (2C, CH_3), 21.9 (2C, CH_2), 21.3 (2C, CH_2), 18.6 (2C, CH_3), 18.5 (2C, CH_3), 18.4 (2C, CH_3), 18.3 (2C, CH_3), 12.3 (2C, CH), 12.3 (2C, CH), 10.1 (1C, CH_2), 10.1 (1C, CH_2).

HR-MS (ESI, MeOH) calcd. For $C_{35}H_{50}N_2NaOSi_2^+$: [M + Na]$^+$, 593.3354; found [M + Na]$^+$, 593.3358.

4,4’- (((2- Ethynyl- 1,4- phenylene) bis (ethyne- 2,1-diyl)) bis (diisopropylsilanediyl)) dibutanenitrile (16)

A two-neck round-bottom flask was cleaned by the following treatment to remove all copper-ions from previous reactions. The flask was filled with conc. H_2SO_4 and sonicated for 10 min, followed by washing with H_2O, NaOH (1*M*), H_2O, and acetone. The flask was dried in the heating oven overnight and flushed with argon.

From a mixture of NaOH (336 mg, 8.40 mmol, 3.0 eq) in toluene (100 mL) was distilled off the H_2O for 15 min. **15** (1.60 g, 2.80 mmol, 1.0 eq) in toluene (20 mL) was added, and the mixture refluxed for 1 h. After the reaction was completed according to TLC and GC–MS, the mixture was cooled to room temperature and diluted with EtOAc (300 mL). The organic phase was washed successively with sat. NH_4Cl (2 × 300 mL), brine (300 mL), and dried over Na_2SO_4. The organic phase was concentrated under reduced pressure and subjected to column chromatography (300 g SiO_2, Cy:EtOAc 92:8). Compound **16** (1.14 g, 2.22 mmol, 79%) was obtained as a yellow oil.

R_f = 0.34 (Cy/EtOAc, 4:1). ^{1}H NMR (500 MHz, CD_2Cl_2) δ = 7.60 (dd, *J*=1.6, 0.5, H_{Ar}, 1H), 7.44 (dd, *J*=8.0, 0.6, H_{Ar}, 1H), 7.40 (dd, *J*=8.1, 1.7, H_{Ar}, 1H), 3.39 (s, H_{alkyne}, 1H), 2.43 (t, *J*=7.0, CH_2, 2H), 2.42 (t, *J*=7.0, CH_2, 2H), 1.92 – 1.79 (m, 2 x CH_2, 4H), 1.17 – 1.05 (m, 4 x *i*Pr, 28H), 0.88 – 0.80 (m, 2 x CH_2, 4H) ^{13}C NMR (126 MHz, CD_2Cl_2) δ = 136.4 (1C, C_{Ar}), 132.9 (1C, C_{Ar}), 132.3 (1C, C_{Ar}), 126.5 (1C, C_{Ar}), 125.7 (1C, C_{Ar}), 123.8 (1C, C_{Ar}), 120.4 (1C, CN), 120.3 (1C, CN), 106.5 (1C, $C_{alkyine}$), 105.6 (1C, $C_{alkyine}$), 97.1 (1C, $C_{alkyine}$), 93.4 (1C, $C_{alkyine}$), 82.4 (1C, $C_{alkyine}$), 81.7 (1C, $C_{alkyine}$), 21.9 (2C, CH_2), 21.8 (2C, CH_2), 21.3 (2C, CH_2), 21.2 (2C, CH_2), 18.5 (2C, CH_3), 18.5 (2C, CH_3), 18.3 (2C, CH_3), 18.3 (2C, CH_3), 12.3 (2C, CH), 12.2 (2C, CH), 10.1 (2C, CH_2), 10.1 (2C, CH_2).

HR-MS (ESI, MeOH) calcd. For $C_{32}H_{44}N_2NaSi_2^+$: [M + Na]$^+$, 535.2935; found [M + Na]$^+$, 535.2938.

4- (((2- Ethynylphenyl) ethynyl) diisopropylsilyl) butanenitrile (E)

A two-neck round-bottom flask was cleaned by the following treatment to remove all copper-ions from previous reactions. The flask was filled with conc. H_2SO_4 and sonicated for 10 min, followed by washing with H_2O, NaOH (1*M*), H_2O, and acetone. The flask was dried in the heating oven overnight and flushed with argon. From a mixture of NaOH (1.39 g, 34.7 mmol, 4.0 eq.) in toluene (250 mL) was distilled of the H_2O for 15 min. 4- (((2- (3- hydroxy- 3- methylbut- 1- yn- 1- yl) phenyl) ethynyl) diisopropylsilyl) butanenitrile[5] (3.17 g, 8.67 mmol, 1.0 eq) in toluene (10 mL) was added and the mixture refluxed for 20 min. After the reaction was completed (TLC and GC–MS), the mixture was cooled to room temperature and diluted with EtOAc (300 mL). The organic phase was washed successively with sat. NH_4Cl (2 × 300 mL), brine (300 mL), and dried over Na_2SO_4. The organic phase was concentrated under reduced pressure and subjected to column chromatography (340 g SiO_2, Cy:EtOAc 92:8->85:15). Compound **E** (2.48 g, 8.06 mmol, 93%) was obtained as a yellow oil.

R_f = 0.36 (Cy/EtOAc, 10:1).

^{1}H NMR (500 MHz, CD_2Cl_2) δ = 7.53 – 7.48 (m, H_{Ar}, 2H), 7.35 – 7.29 (m, H_{Ar}, 2H), 3.37 (s, $H_{alkyine}$, 1H), 2.43 (t, *J*=7.0, CH_2, 2H), 1.95 – 1.84 (m, CH_2, 2H), 1.18 – 1.04 (m, 2 x iPr, 14H), 0.89 – 0.80 (m, CH_2, 2H).

The spectra data of this compound was identical to those reported in the literature.[5]

4- (((2- ((2'- Bromo- 2''- chloro- [1,1':4',1''- terphenyl]- 3-yl) ethynyl) phenyl) ethynyl) diisopropylsilyl) butanenitrile (17)

An oven-dried and argon flushed Schlenk tube was charged with **10** (1.15 g, 2.46 mmol, 1.0 eq), dry THF (9 mL), and piperidine (3 mL), and the mixture degassed by passing argon through for 10 min. Then **E** (946 mg, 3.08 mmol, 1.25 eq.) was added, and the reaction mixture was again degassed for 5 min. $(Ph_3P)_2PdCl_2$ (86.3 mg, 123 µmol, 0.05 eq.) and CuI (28.7 mg, 148 µmol, 0.06 eq.) were added. The exothermic mixture was cooled with a water bath while degassing by passing argon through for a further 5 min. The yellow suspension was stirred at room temperature for 1 h. After the reaction was completed according to TLC, the mixture was cooled to room temperature and diluted with EtOAc (300 mL) and washed with H_2O (200 mL) and brine (200 mL), and dried over Na_2SO_4. The organic phase was concentrated under reduced pressure and subjected to column chromatography (370 g SiO_2, Cy:EtOAc 91:9). Compound **17** was obtained as an orange oil, which was repeatedly dissolved in CH_2Cl_2 and the solvent removed by high vacuum (1.49 g, 2.30 mmol, 93%).

R_f = 0.25 (Cy/EtOAc, 10:1).

^{1}H NMR (500 MHz, C_6D_6) δ = 7.72 (t, *J*=1.7, H_{Ar}, 1H), 7.70 (t, *J*=1.5, H_{Ar}, 1H), 7.59 (dt, *J*=7.6, 1.4, H_{Ar}, 1H), 7.43 – 7.39 (m, H_{Ar}, 2H), 7.26 – 7.21 (m, H_{Ar}, 3H), 7.13 (t, *J*=7.8, H_{Ar}, 1H), 7.09 (dd, *J*=7.9, 1.3, H_{Ar}, 1H), 7.00 (dt, *J*=7.5, 1.6, H_{Ar}, 1H), 6.89 (tt, *J*=7.4, 1.4, H_{Ar}, 1H), 6.86 – 6.77 (m, H_{Ar}, 3H), 1.55 – 1.49 (m, 2 x CH_2, 4H), 1.12 (dd, *J*=6.4, 2.0, 2 x CH_3, 6H), 1.06 (d, *J*=7.3, 2 x CH_3, 6H), 1.01 – 0.89 (m, 2 x CH, 2H), 0.60 (ddd, *J*=9.5, 5.4, 1.7, CH_2, 2H).

^{13}C NMR (126 MHz, C_6D_6) δ = 141.5 (1C, C_{Ar}), 141.3 (1C, C_{Ar}), 140.8 (1C, C_{Ar}), 139.0 (1C, C_{Ar}), 134.3 (1C, C_{Ar}), 133.3 (1C, C_{Ar}), 133.0 (1C, C_{Ar}), 132.9 (1C, C_{Ar}), 132.4 (1C, C_{Ar}), 131.6 (1C, C_{Ar}), 131.1 (1C, C_{Ar}), 131.0 (1C, C_{Ar}), 130.3 (1C, C_{Ar}), 130.1 (1C, C_{Ar}), 129.3 (1C, C_{Ar}), 129.0 (1C, C_{Ar}), 128.6 (1C, C_{Ar}), 128.5 (1C, C_{Ar}), 127.2 (1C, C_{Ar}), 126.5 (1C, C_{Ar}), 126.1 (1C, C_{Ar}), 123.7 (1C, C_{Ar}), 122.6 (1C, C_{Ar}),

119.4 (1C, CN), 106.8 (1C, $C_{alkyine}$), 94.4 (1C, $C_{alkyine}$), 93.8 (1C, $C_{alkyine}$), 89.2 (1C, $C_{alkyine}$), 21.6 (1C, CH_2), 20.4 (1C, CH_2), 18.5 (2C, CH_3), 18.2 (2C, CH_3), 12.1 (2C, CH), 9.7 (1C, CH_2). The peak of 128.3 is only visible in the DEPT-135 experiment, as the signal is overlain by C_6D_6.

^{1}H NMR (500 MHz, CD_2Cl_2) δ = 7.80 (d, *J*=1.7, H_{Ar}, 1H), 7.65 – 7.63 (m, H_{Ar}, 1H), 7.61 – 7.57 (m, H_{Ar}, 1H), 7.57 – 7.55 (m, H_{Ar}, 1H), 7.54 – 7.53 (m, H_{Ar}, 1H), 7.51 (t, *J*=1.8, H_{Ar}, 1H), 7.50 – 7.48 (m, H_{Ar}, 2H), 7.43 – 7.38 (m, H_{Ar}, 3H), 7.38 – 7.36 (m, H_{Ar}, 1H), 7.36 (d, *J*=1.9, H_{Ar}, 1H), 7.34 (dd, *J*=1.9, 1.2, H_{Ar}, 1H), 7.32 (d, *J*=1.3, H_{Ar}, 1H), 2.26 (t, *J*=6.9, CH_2, 2H), 1.87 – 1.78 (m, CH_2, 2H), 1.12 – 1.02 (m, 2 x *i*Pr,14H), 0.83 – 0.78 (m, CH_2, 2H).

^{13}C NMR (126 MHz, CD_2Cl_2) δ = 141.6 (1C, C_{Ar}), 141.3 (1C, C_{Ar}), 141.0 (1C, C_{Ar}), 139.1 (1C, C_{Ar}), 134.4 (1C, C_{Ar}), 133.3 (1C, C_{Ar}), 133.2 (1C, C_{Ar}), 132.9 (1C, C_{Ar}), 132.6 (1C, C_{Ar}), 131.9 (1C, C_{Ar}), 131.3 (1C, C_{Ar}), 131.2 (1C, C_{Ar}), 130.6 (1C, C_{Ar}), 130.3 (1C, C_{Ar}), 129.8 (1C, C_{Ar}), 129.3 (1C, C_{Ar}), 129.0 (1C, C_{Ar}), 128.8 (1C, C_{Ar}), 128.7 (1C, C_{Ar}), 127.7 (1C, C_{Ar}), 126.2 (1C, C_{Ar}), 126.0 (1C, C_{Ar}), 123.6 (1C, C_{Ar}), 122.5 (1C, C_{Ar}), 120.3 (1C, CN), 106.5 (1C, $C_{alkyine}$), 94.7 (1C, $C_{alkyine}$), 93.4 (1C, $C_{alkyine}$), 88.9 (1C, $C_{alkyine}$), 21.9 (1C, CH_2), 21.2 (1C, CH_2), 18.6 (2C, CH_3), 18.3 (2C, CH_3), 12.3 (2C, CH), 10.1 (1C, CH_2).

HR-MS (ESI, MeOH) calcd. for $C_{38}H_{39}BrClN_2Si^+$: [M + NH_4]$^+$, 665.1749; found [M + NH_4]$^+$, 665.1751.

4,4'- (((2- ((2''- chloro- 3- ((2- (((3- cyanopropyl) diisopropylsilyl) ethynyl) phenyl) ethynyl)- [1,1':4',1''- terphenyl]- 2'- yl) ethynyl)- 1,4-phenylene) bis (ethyne- 2,1- diyl)) bis (diisopropylsilanediyl)) dibutanenitrile (18)

An oven-dried and argon flushed Schlenk tube was charged with **17** (300 mg, 462 µmol, 1.0 eq) and piperidine (5 mL), and the mixture degassed by passing argon through for 10 min. Then **16** (284 mg, 554 µmol, 1.2 eq.) in piperidine (5 mL) was added, and the reaction mixture was again bubbled for 5 min and then $(Ph_3P)_2PdCl_2$ (16.2 mg, 23.1 µmol, 0.05 eq.) and CuI (5.39 mg, 27.7 µmol, 0.06 eq.) were added. The mixture was degassed by passing argon through for a further 5 min. The yellow suspension was stirred at 120 °C for 18 h. As the reaction was not completed according to TLC, more **16** (284 mg, 554 µmol, 1.2 eq.) and $(Ph_3P)_2PdCl_2$ (16.2 mg, 23.1 µmol, 0.05 eq.) were added in two portions over 6 h. After the reaction was completed according to TLC, the mixture was cooled to room temperature and diluted with EtOAc (50 mL) and washed with H_2O (50 mL) and brine (50 mL), and dried over Na_2SO_4. The organic phase was concentrated under reduced pressure and subjected to column chromatography (200 g SiO_2, Tol: CH_2Cl_2 100:1), followed by further purification by SEC ($CHCl_3$). Compound **18** (146 mg, 182 µmol, 40%) was obtained as a colorless oil.

R_f = 0.20 (Cy/EtOAc, 4:1).

^{1}H NMR (500 MHz, C_6D_6) δ = 7.97 (h, *J*=0.9, H_{Ar}, 1H), 7.94 (p, *J*=0.8, H_{Ar}, 1H), 7.73 (ddq, *J*=7.8, 1.9, 1.0, H_{Ar}, 1H), 7.69 (ddq, *J*=7.7, 1.9, 1.0, H_{Ar}, 1H), 7.58 (dq, *J*=1.8, 0.8, H_{Ar}, 1H), 7.48 – 7.44 (m, H_{Ar}, 1H), 7.41 (ddd, *J*=7.6, 1.4, 0.7, H_{Ar}, 1H), 7.34 (dd, *J*=8.0, 1.9, H_{Ar}, 1H), 7.32 (dd, *J*=8.0, 1.2, H_{Ar}, 1H), 7.30 – 7.25 (m, H_{Ar}, 2H), 7.24 – 7.20 (m, H_{Ar}, 2H), 7.10 (dd, *J*=8.1, 1.7, H_{Ar}, 1H), 7.01 (td, *J*=7.5, 1.2, H_{Ar}, 1H), 6.92 – 6.89 (m, H_{Ar}, 1H), 6.89 – 6.85 (m, H_{Ar}, 1H), 6.81 (tt, *J*=7.6, 0.9, H_{Ar}, 1H), 1.63 – 1.38 (m, 6 x CH_2, 12H), 1.15 – 0.99 (m,6 x CH_3, 36H), 0.99 – 0.86 (m, 6 x CH, 6H), 0.62 – 0.51 (m, 3 x CH_2, 6H).

^{13}C NMR (126 MHz, C_6D_6) δ = 142.7 (1C, C_{Ar}), 140.7 (1C, C_{Ar}), 139.7 (1C, C_{Ar}), 139.3 (1C, C_{Ar}), 135.9 (1C, C_{Ar}), 134.5 (1C, C_{Ar}), 133.2 (1C, C_{Ar}), 133.1 (1C, C_{Ar}), 133.0 (1C, C_{Ar}), 132.9 (1C, C_{Ar}), 132.5 (1C, C_{Ar}), 131.6 (1C, C_{Ar}), 131.6 (1C, C_{Ar}), 131.2 (1C, C_{Ar}), 130.7 (1C, C_{Ar}), 130.4 (1C, C_{Ar}), 130.2 (1C, C_{Ar}), 129.7 (1C, C_{Ar}), 129.5 (1C, C_{Ar}), 128.7 (1C, C_{Ar}), 128.4 (1C, C_{Ar}), 127.3 (1C, C_{Ar}), 126.8 (1C, C_{Ar}), 126.6 (1C, C_{Ar}), 126.0 (1C, C_{Ar}), 125.7 (1C, C_{Ar}), 123.9 (1C, C_{Ar}), 123.7 (1C, C_{Ar}), 121.7 (1C, C_{Ar}), 119.4 (1C, CN), 119.3 (1C, CN), 119.3 (1C, CN), 106.8 (2C, $C_{alkyine}$), 106.2 (1C, $C_{alkyine}$), 96.8 (1C, $C_{alkyine}$), 94.4 (1C, $C_{alkyine}$), 94.1 (1C, $C_{alkyine}$), 93.8 (1C, $C_{alkyine}$), 93.0 (1C, $C_{alkyine}$), 91.3 (1C, $C_{alkyine}$), 89.2 (1C, $C_{alkyine}$), 21.6 (1C, CH_2), 21.5 (1C, CH_2), 21.4 (1C, CH_2), 20.4 (1C, CH_2), 20.4 (1C, CH_2), 20.3 (1C, CH_2), 18.5 (2C, CH_3), 18.4 (2C, CH_3), 18.4 (2C, CH_3), 18.2 (2C, CH_3), 18.2 (2C, CH_3), 18.2 (2C, CH_3), 12.1 (2C, CH), 12.0(2C, CH), 12.0(2C, CH), 9.7 (1C, CH_2), 9.7 (1C, CH_2), 9.6 (1C, CH_2).

^{1}H NMR (500 MHz, CD_2Cl_2) δ = 7.85 (d, *J*=1.7, H_{Ar}, 1H), 7.81 (dt, *J*=7.5, 1.6, H_{Ar}, 1H), 7.72 (t, *J*=1.2, H_{Ar}, 1H), 7.62 – 7.48 (m, H_{Ar}, 7H), 7.47 – 7.29 (m, H_{Ar}, 8H), 2.40 (t, *J*=6.9, CH_2, 2H), 2.24 (dt, *J*=10.0, 6.9, 2 x CH_2, 4H), 1.89 – 1.68 (m, 3 x CH_2, 6H), 1.05 (dddd, *J*=10.6, 9.3, 5.3, 2.7, 6 x *i*Pr 42H), 0.78 (dddd, *J*=12.3, 8.8, 5.8, 3.2, 3 x CH_2, 6H)

^{13}C NMR (126 MHz, CD_2Cl_2) δ = 142.5 (1C, C_{Ar}), 140.6 (1C, C_{Ar}), 139.5 (1C, C_{Ar}), 139.2 (1C, C_{Ar}), 135.6 (1C, C_{Ar}), 134.4 (1C, C_{Ar}), 133.1 (1C, C_{Ar}), 133.0 (1C, C_{Ar}), 133.0 (1C, C_{Ar}), 132.7 (1C, C_{Ar}), 132.5 (1C, C_{Ar}), 131.6 (1C, C_{Ar}), 131.6 (1C, C_{Ar}), 131.1 (1C, C_{Ar}), 130.6 (1C, C_{Ar}), 130.4 (1C, C_{Ar}), 130.0 (1C, C_{Ar}), 129.6 (1C, C_{Ar}), 129.5 (1C, C_{Ar}), 128.8 (1C, C_{Ar}), 128.5 (1C, C_{Ar}), 128.4 (1C, C_{Ar}), 127.5 (1C, C_{Ar}), 126.2 (1C, C_{Ar}), 126.1 (1C, C_{Ar}), 125.7 (1C, C_{Ar}), 125.4 (1C, C_{Ar}), 123.6 (1C, C_{Ar}), 123.6 (1C, C_{Ar}), 121.4 (1C, C_{Ar}), 120.1 (1C, CN), 120.1 (1C, CN), 120.0 (1C, CN), 106.4 (1C, $C_{alkyine}$), 106.3 (1C, $C_{alkyine}$), 105.8 (1C, $C_{alkyine}$), 96.7 (1C, $C_{alkyine}$), 94.4 (1C, $C_{alkyine}$), 93.5 (1C, $C_{alkyine}$), 93.4 (1C, $C_{alkyine}$), 93.0 (1C, $C_{alkyine}$), 90.9 (1C, $C_{alkyine}$), 88.7 (1C, $C_{alkyine}$), 21.7 (1C, CH_2), 21.6 (2C, CH_2), 21.1 (1C, CH_2), 20.9 (1C, CH_2), 20.9 (1C, CH_2), 18.4 (2C, CH_3), 18.3 (2C, CH_3), 18.3 (2C, CH_3), 18.1 (2C, CH_3), 18.1 (2C, CH_3), 18.1 (2C, CH_3), 12.2 (2C, CH), 12.1 (2C, CH), 12.1 (2C, CH), 9.9 (1C, CH_2), 9.9 (2C, CH_2).

HR-MS (ESI, MeOH) calcd. for $C_{70}H_{82}ClN_4Si_3^+$: [M + NH_4]$^+$, 1097.5530; found [M + NH_4]$^+$, 1097.5523.

4- (((3-Ethynylphenyl) ethynyl) diisopropylsilyl) butanenitrile (C_1)

A two-neck round-bottom flask was cleaned by the following treatment to remove all copper-ions from previous reactions. The flask was filled with conc. H_2SO_4 and sonicated for 10 min, followed by washing with H_2O, NaOH (1*M*), H_2O, and acetone. The flask was dried in the heating oven overnight and flushed with argon. From a mixture of NaOH (1.04 mg, 26.1 mmol, 4.0 eq) in toluene (190 mL) was distilled off the H_2O for 15 min. 4- (((3- (3-hydroxy- 3- methylbut- 1- yn- 1-yl) phenyl) ethynyl) diisopropylsilyl) butanenitrile[5] (2.38 g, 6.52 mmol, 1.0 eq) in toluene (20 mL) was added, and the mixture refluxed for 1 h. After the reaction was completed according to TLC and GC-MS, the mixture was cooled to room temperature and diluted with EtOAc (250 mL). The organic phase was washed

successively with sat. NH_4Cl (200 mL), brine (200 mL), and dried over Na_2SO_4. The organic phase was concentrated under reduced pressure and subjected to column chromatography (300 g SiO_2, Cy:EtOAc 92:8). Compound **C_1** (1.69 g, 5.50 mmol, 84%) was obtained as a yellow oil.

R_f = 0.30 (Cy/EtOAc, 10:1).

^{1}H NMR (400 MHz, CD_2Cl_2) δ = 7.60 (t, *J*=1.6, H_{Ar}, 1H), 7.47 (p, *J*=1.4, H_{Ar}, 1H), 7.45 (p, *J*=1.3, H_{Ar}, 1H), 7.30 (t, *J*=7.8, H_{Ar}, 1H), 3.16 (s, $H_{alkyine}$, 1H), 2.42 (t, *J*=7.0, 2 x CH_2, 2H), 1.90 – 1.80 (m, 2 x CH_2, 2H), 1.14 – 1.06 (m, 2 x *i*Pr, 14H), 0.86 – 0.80 (m, 2 x CH_2, 2H). The spectra data of this compound was identical to those reported in the literature.[5]

4- (((3- Aminophenyl) ethynyl) diisopropylsilyl) butanenitrile (21)

An oven-dried and argon flushed Schlenk tube (100 mL) was charged with 3-iodoaniline (**20**, 2.75 mL, 22.8 mmol, 1.0 eq), dry THF (30 mL) and piperidine (10 mL), and the yellow mixture degassed by passing argon through for 10 min. Then CPDIPS-acetylene (8.0 mL, 34.2 mmol, 1.5 eq.) was added, and the reaction mixture was again bubbled with argon for 5 min and then $(Ph_3P)_2PdCl_2$ (800 mg, 1.14 mmol, 0.05 eq.) and CuI (266 mg, 1.37 mmol, 0.06 eq.) were added. The exothermic mixture was degassed by passing argon through for a further 5 min. The yellow suspension was stirred at room temperature for 1 h until it was no longer exothermic. After the reaction was completed according to GC-MS and TLC, the mixture was dissolved in EtOAc (200 mL). The organic layer was washed with water (200 mL) and brine (200 mL) and dried over Na_2SO_4. The mixture was concentrated under reduced pressure and subjected to column chromatography. (340 g SiO_2, Cy:EtOAc 9:1->7:3). Compound **21** (6.81 g, 22.8 mmol, quant.) was obtained as a light brown solid.

R_f = 0.26 (Cy/EtOAc, 3:1).

^{1}H NMR (500 MHz, CD_2Cl_2) δ = 7.09 (t, *J* = 7.8 Hz, H_{Ar}, 1H), 6.85 (dt, *J* = 7.6, 1.2 Hz, H_{Ar}, 1H), 6.78 (dd, *J* = 2.4, 1.5 Hz, H_{Ar}, 1H), 6.66 (ddd, *J* = 8.1, 2.4, 1.0 Hz, H_{Ar}, 1H), 3.68 (s, NH_2, 2H), 2.42 (t, *J* = 7.0 Hz, CH_2, 2H), 1.90 – 1.81 (m, CH_2, 2H), 1.17 – 1.01 (m, 2 x iPr, 14H), 0.85 – 0.79 (m, CH_2, 2H).

^{13}C NMR (126 MHz, CD_2Cl_2) δ = 147.3 (1C, C_{Ar}), 129.7 (1C, C_{Ar}), 124.2 (1C, C_{Ar}), 122.5 (1C, C_{Ar}), 120.4 (1C, CN), 118.4 (1C, C_{Ar}), 116.0 (1C, C_{Ar}), 108.7 (1C, $C_{alkyine}$), 89.1 (1C, $C_{alkyine}$), 21.9 (1C, CH_2), 21.2 (1C, CH_2), 18.5 (2C, CH_3), 18.3 (2C, CH_3), 12.3 (2C, CH), 10.2 (1C, CH_2).

HR-MS (ESI, MeOH) calcd. for $C_{18}H_{27}N_2Si^+$: [M + H]$^+$, 299.1938; found [M + H]$^+$, 299.1935.

4- (((5- Amino- 2- iodophenyl) ethynyl) diisopropylsilyl) butanenitrile (22)

To a solution of **21** (4.62 g, 15.0 mmol, 1.0 eq) in DMSO (150 mL) was added NIS (3.51 mg, 15.2 mmol, 1.01 eq.), and the mixture stirred at room temperature for 1 h. After the reaction was completed according to GC-MS and TLC, the mixture was cooled to 5 °C and quenched with aqueous $NaHCO_3$ (200 mL) (exothermic for the first 50 mL), and the brown suspension was filtered off and washed with water (50 mL). The rose filtrate was dissolved in DCM (50 mL) and washed with water (50 mL). The aqueous phase was extracted with DCM (50 mL), and the combined organic phases were dried over Na_2SO_4 and concentrated under reduced pressure, and subjected to column chromatography. (340 g SiO_2, Cy:EtOAc 9:1->7:3)

Compound **22** (5.39 g, 12.7 mmol, 85%) was obtained as a brown solid.

R_f = 0.18 (Cy/EtOAc, 3:1).

^{1}H NMR (500 MHz, CD_2Cl_2) δ = 7.53 (d, *J*=8.5, H_{Ar}, 1H), 6.86 (d, *J*=2.8, H_{Ar}, 1H), 6.42 (dd, *J*=8.5, 2.9, H_{Ar}, 1H), 3.75 (s, NH_2, 2H), 2.43 (t, *J*=7.0, CH_2, 2H), 1.95 – 1.85 (m, CH_2, 2H), 1.18 – 1.07 (m, 2 x iPr, 14H), 0.89 – 0.82 (m, CH_2, 2H).

^{13}C NMR (126 MHz, CD_2Cl_2) δ = 147.2 (1C, C_{Ar}), 139.6 (1C, C_{Ar}), 130.2 (1C, C_{Ar}), 120.4 (1C, CN), 119.9 (1C, C_{Ar}), 117.9 (1C, C_{Ar}), 109.4 (1C, C_{Ar}), 93.7 (1C, $C_{alkyine}$), 85.7 (1C, $C_{alkyine}$), 21.9 (1C, CH_2), 21.3 (1C, CH_2), 18.6 (2C, CH_3), 18.3 (2C, CH_3), 12.3 (2C, CH), 10.1 (1C, CH_2).

HR-MS (ESI, MeOH) calcd. for $C_{18}H_{26}IN_2Si^+$: [M + H]$^+$, 425.0904; found [M + H]$^+$, 425.0910.

Compound **23** (122 mg, 288 µmol, 1.9%) was obtained as a red oil.

R_f = 0.38 (Cy/EtOAc, 3:1).

^{1}H NMR (500 MHz, CD_2Cl_2) δ = 7.58 (d, *J*=8.1, H_{Ar}, 1H), 6.85 (d, *J*=1.8, H_{Ar}, 1H), 6.56 (dd, *J*=8.1, 1.9, H_{Ar}, 1H), 4.15 (s, NH_2, 2H), 2.42 (t, *J*=7.0, CH_2, 2H), 1.88 – 1.80 (m, CH_2, 2H), 1.13 – 1.04 (m, 2 x iPr, 14H), 0.85 – 0.79 (m, CH_2, 2H).

^{13}C NMR (126 MHz, CD_2Cl_2) δ = 147.5 (1C, C_{Ar}), 139.4 (1C, C_{Ar}), 124.5 (1C, C_{Ar}), 123.5 (1C, C_{Ar}), 120.3 (1C, CN), 117.9 (1C, C_{Ar}), 107.6 (1C, C_{Ar}), 90.6 (1C, $C_{alkyine}$), 84.8 (1C, $C_{alkyine}$), 21.9 (1C, CH_2), 21.2 (1C, CH_2), 18.5 (2C, CH_3), 18.3 (2C, CH_3), 12.3 (2C, CH), 10.1 (1C, CH_2).

HR-MS (ESI, MeOH) calcd. for $C_{18}H_{26}IN_2Si^+$: [M + H]$^+$, 425.0904; found [M + H]$^+$, 425.0909.

Compound **24** and compound **25** were inseparable by column chromatography and therefore separated by SEC ($CHCl_3$) from each other.

Compound **24** (102 mg, 240 µmol, 1.6%) was obtained as a brown oil.

R_f = 0.30 (Cy/EtOAc, 3:1).

^{1}H NMR (500 MHz, CD_2Cl_2) δ = 7.08 (t, *J*=7.9, H_{Ar}, 1H), 6.90 (dd, *J*=7.5, 1.5, H_{Ar}, 1H), 6.71 (dd, *J*=8.1, 1.5, H_{Ar}, 1H), 4.30 (s, NH_2, 2H), 2.43 (t, *J*=7.0, CH_2, 2H), 1.96 – 1.86 (m, CH_2, 2H), 1.21 – 1.08 (m, 2 x iPr, 14H), 0.90 – 0.81 (m, CH_2, 2H).

^{13}C NMR (126 MHz, CD_2Cl_2) δ = 148.4 (1C, C_{Ar}), 130.9 (1C, C_{Ar}), 129.2 (1C, C_{Ar}), 123.7 (1C, C_{Ar}), 120.4 (1C, CN), 114.9 (1C, C_{Ar}), 110.2 (1C, C_{Ar}), 93.9 (1C, $C_{alkyine}$), 90.1 (1C, $C_{alkyine}$), 22.0 (1C, CH_2), 21.3 (1C, CH_2), 18.6 (2C, CH_3), 18.3 (2C, CH_3), 12.3 (2C, CH), 10.1 (1C, CH_2).

HR-MS (ESI, MeOH) calcd. for $C_{18}H_{26}IN_2Si^+$: [M + H]$^+$, 425.0904; found [M + H]$^+$, 425.0907.

Compound **25** (408 mg, 741 µmol, 4.9%) was obtained as a brown oil.

R_f = 0.30 (Cy/EtOAc, 3:1).

^{1}H NMR (500 MHz, CD_2Cl_2) δ = 7.55 (d, *J*=8.5, H_{Ar}, 1H), 6.46 (d, *J*=8.6, H_{Ar}, 1H), 4.36 (s, NH_2, 2H), 2.44 (t, *J*=7.0, CH_2, 2H), 2.01 - 1.91 (m, CH_2, 2H), 1.23 – 1.12 (m, 2 x iPr, 14H), 0.92 – 0.85 (m, CH_2, 2H).

^{13}C NMR (126 MHz, CD_2Cl_2) δ = 148.4 (1C, C_{Ar}), 139.1 (1C, C_{Ar}), 135.1 (1C, C_{Ar}), 120.4 (1C, CN), 116.1 (1C, C_{Ar}), 112.0 (1C, C_{Ar}), 98.6 (1C, C_{Ar}), 89.7 (1C, $C_{alkyine}$), 84.7 (1C, $C_{alkyine}$), 22.0 (1C, CH_2), 21.3 (1C, CH_2), 18.7 (2C, CH_3), 18.4 (2C, CH_3), 12.4 (2C, CH), 10.0 (1C, CH_2).

HR-MS (ESI, MeOH) calcd. for $C_{18}H_{25}I_2N_2NaSi^+$: [M + Na]$^+$, 572.9690; found [M + Na]$^+$, 572.9690.

4- (((4- Amino- 3'- bromo- [1,1'- biphenyl]- 2- yl) ethynyl) diisopropylsilyl) butanenitrile (26)

An oven-dried and argon flushed Schlenk tube was charged with **22** (4.88 g, 11.5 mol, 1.0 eq), 3-bromophenylboronic acid (4.16 g, 20.7 mmol, 1.8 eq), and K_2CO_3 (4.82 g, 34.5 mmol, 3.0 eq) and placed under vacuum for 5 min. Then 1,2-dimethoxyethane (DME, 50 mL), EtOH (12.5 mL), and H_2O (12.5 mL) were added and the mixture degassed by passing argon through for another 5 min. $(Ph_3P)_2PdCl_2$ (163 mg, 230 µmol, 0.02 eq) was added, and the mixture was heated to 80 °C for 12 h. After the reaction was completed according to GC-MS and TLC, the mixture was dissolved in DCM (250 mL) and washed with H_2O (200 mL) and brine (200 mL). The organic phase was dried over Na_2SO_4 and concentrated under reduced pressure, and subjected to column chromatography (340 g SiO_2, Cy:EtOAc 95:5->60:40). Compound **26** (4.40 g, 9.70 mmol, 84%) was obtained as an orange oil.

R_f = 0.21 (Cy/EtOAc, 4:1).

^{1}H NMR (500 MHz, CD_2Cl_2) δ = 7.70 (t, *J*=1.8, H_{Ar}, 1H), 7.47 – 7.42 (m, H_{Ar}, 2H), 7.26 (t, *J*=7.9, H_{Ar}, 1H), 7.13 (d, *J*=8.3, H_{Ar}, 1H), 6.90 (d, *J*=2.5, H_{Ar}, 1H), 6.73 (dd, *J*=8.3, 2.5, H_{Ar}, 1H), 3.79 (s, NH_2, 2H), 2.31 (t, *J*=7.0, CH_2, 2H), 1.70 – 1.61 (m, CH_2, 2H), 1.01 (m, 2 x iPr, 14H), 0.76 – 0.69 (m, CH_2, 2H).

^{13}C NMR (126 MHz, CD_2Cl_2) δ = 146.7 (1C, C_{Ar}), 143.4 (1C, C_{Ar}), 133.4 (1C, C_{Ar}), 132.7 (1C, C_{Ar}), 130.9 (1C, C_{Ar}), 130.1 (1C, C_{Ar}), 130.1 (1C, C_{Ar}), 128.5 (1C, C_{Ar}), 122.4 (1C, C_{Ar}), 122.2 (1C, C_{Ar}), 120.3(1C, CN), 119.7 (1C, C_{Ar}), 116.3 (1C, C_{Ar}), 107.5 (1C, $C_{alkyine}$), 93.1 (1C, $C_{alkyine}$), 21.8 (1C, CH_2), 21.2 (1C, CH_2), 18.5 (2C, CH_3), 18.2 (2C, CH_3), 12.2 (2C, CH), 10.0 (1C, CH_2).

HR-MS (ESI, MeOH) calcd. for $C_{24}H_{30}BrN_2Si^+$: [M + H]$^+$, 453.1356; found [M + H]$^+$, 453.1356.

4- (((3'- Bromo- 4- iodo- [1,1'- biphenyl]- 2- yl) ethynyl) diisopropylsilyl) butanenitrile (27)

An oven-dried and argon-flushed three-neck round-bottom flask was charged with *p*TsOH (7.41 g, 43.1 mmol, 7.5 eq.) in MeCN (31 mL). A solution of **26** (2.60 g, 5.74 mmol, 1.0 eq.) in MeCN (26 mL) was added at room temperature, and the resulting suspension was cooled to 10 °C. Afterward, a solution of $NaNO_2$ (1.21 mg, 17.2 mmol, 3.0 eq.) and KI (3.61 g, 21.5 mmol, 3.75 eq.) in water (16 mL) was added dropwise over 5 min (gas evolution) at 4-5 °C. After additional stirring for 30 min. at 10 °C, the solution was allowed to reach room temperature and stirred for 16 h. After the reaction was completed according to GC-MS and TLC, the reaction was quenched with a mixture of H_2O (260 mL), sat. aq. $NaHCO^3$-sol. (160 mL) and sat. aq. $Na_2S_2O_3$-sol. (160 mL). The aqueous phase was extracted with EtOAc (2 x 250 mL), and the combined organic phases were washed with brine (200 mL), dried over Na_2SO_4, concentrated under reduced pressure, and subjected to column chromatography (340 g SiO_2, Cy:EtOAc 98:2->85:15). Compound **27** (2.65 g,5.74 mmol, 82%) was obtained as a light-yellow oil.

R_f = 0.34 (Cy/EtOAc, 10:1).

^{1}H NMR (500 MHz, CD_2Cl_2) δ = 7.95 (d, J=1.8, H_{Ar}, 1H), 7.74 (dd, J=8.2, 1.9, H_{Ar}, 1H), 7.70 (t, J=1.9, H_{Ar}, 1H), 7.52 (ddd, J=8.0, 2.0, 1.0, H_{Ar}, 1H), 7.45 (dt, J=7.8, 1.3, H_{Ar}, 1H), 7.31 (t, J=7.9, H_{Ar}, 1H), 7.08 (d, J=8.2, H_{Ar}, 1H), 2.31 (t, J=7.0, CH_2, 2H), 1.66 – 1.59 (m, CH_2, 2H), 1.03 – 0.96 (m, 2 x iPr, 14H), 0.77 – 0.69 (m, CH_2, 2H).

^{13}C NMR (126 MHz, CD_2Cl_2) δ = 142.8 (1C, C_{Ar}), 142.4 (1C, C_{Ar}), 142.2 (1C, C_{Ar}), 138.5 (1C, C_{Ar}), 132.6 (1C, C_{Ar}), 131.4 (1C, C_{Ar}), 131.3 (1C, C_{Ar}), 130.4 (1C, C_{Ar}), 128.4 (1C, C_{Ar}), 124.1 (1C, C_{Ar}), 122.4 (1C, C_{Ar}), 120.2 (1C, CN), 105.2 (1C, C_{Ar}), 95.8 (1C, $C_{alkyine}$), 92.9 (1C, $C_{alkyine}$), 21.7 (1C, CH_2), 21.2 (1C, CH_2), 18.4 (2C, CH_3), 18.2 (2C, CH_3), 12.2 (2C, CH), 9.9 (1C, CH_2).

HR-MS (ESI, MeOH) calcd. For $C_{24}H_{27}BrINNaSi^+$: [M + Na]$^+$, 586.0033; found [M + Na]$^+$, 586.0022.

4- (((2''- Amino- 3- bromo- [1,1':4',1''- terphenyl]- 2'- yl) ethynyl) diisopropylsilyl) butanenitrile (28)

An oven-dried and argon flushed Schlenk tube was charged with 2-aminophenylboronicacid hydrochloride (1.02 g, 5.86 mmol, 1.3 eq) and K_2CO_3 (3.15 g, 22.6 mmol, 5.0 eq) and placed under vacuum for 5 min. Then **27** (2.55 g, 4.52 mmol, 1.0 eq) in DME (25 mL), EtOH (6.3 mL), and H_2O (6.3 mL) were added and the mixture degassed by passing argon through for a further 5 min. $(Ph_3P)_2PdCl_2$ (64.0 mg, 90.2 µmol, 0.02 eq) was added, and the mixture was stirred at room temperature for 12 h. After the reaction was completed acco$_r$ding to TLC, the solution was diluted with EtOAc (250 mL) and washed with H_2O (100 mL), brine (100 mL), and dried over Na_2SO_4. The organic phase was concentrated under reduced pressure and subjected to column chromatography (340 g SiO_2, Cy:EtOAc 95:5->70:30). Compound **28** (2.31 g, 4.36 mmol, 97%) was obtained as an orange oil.

R_f = 0.27 (Cy/EtOAc, 4:1).

^{1}H NMR (500 MHz, CD_2Cl_2) δ = 7.79 (t, *J*=1.8, H_{Ar}, 1H), 7.69 (d, *J*=1.8, H_{Ar}, 1H), 7.55 – 7.52 (m, H_{Ar}, 2H), 7.51 (dd, *J*=7.9, 1.8, H_{Ar}, 1H), 7.43 (d, *J*=7.9, H_{Ar}, 1H), 7.34 (t, *J*=7.8, H_{Ar}, 1H), 7.19 – 7.13 (m, H_{Ar}, 2H), 6.83 (td, *J*=7.5, 1.2, H_{Ar}, 1H), 6.80 (dd, *J*=8.0, 1.1, H_{Ar}, 1H), 4.04 (s, NH_2, 2H), 2.31 (t, *J*=7.0, CH_2, 2H), 1.71 – 1.59 (m, CH_2, 2H), 1.03 – 0.96 (m, 2 x iPr, 14H), 0.76 – 0.70 (m, CH_2, 2H).

^{13}C NMR (126 MHz, CD_2Cl_2) δ = 144.3 (1C, C_{Ar}), 142.9 (1C, C_{Ar}), 142.0 (1C, C_{Ar}), 139.7 (1C, C_{Ar}), 134.7 (1C, C_{Ar}), 132.8 (1C, C_{Ar}), 131.0 (1C, C_{Ar}), 130.8 (1C, C_{Ar}), 130.4 (1C, C_{Ar}), 130.4 (1C, C_{Ar}), 130.2 (1C, C_{Ar}), 129.5 (1C, C_{Ar}), 128.6 (1C, C_{Ar}), 126.5 (1C, C_{Ar}), 122.5 (1C, C_{Ar}), 122.4 (1C, C_{Ar}), 120.3 (1C, CN), 119.1 (1C, C_{Ar}), 116.2 (1C, C_{Ar}), 106.9 (1C, $C_{alkyine}$), 94.4 (1C, $C_{alkyine}$), 21.8 (1C, CH_2), 21.2 (1C, CH_2), 18.5 (2C, CH_3), 18.2 (2C, CH_3), 12.2 (2C, CH), 10.0 (1C, CH_2).

HR-MS (ESI, MeOH) calcd. for $C_{30}H_{34}BrN_2Si^+$: [M + H]$^+$, 529.1669; found [M + H]$^+$, 529.1675.

4- (((3- Bromo- 2''- iodo- [1,1':4',1''- terphenyl]- 2'- yl) ethynyl) diisopropylsilyl) butanenitrile (29)

An oven-dried and argon-flushed three-neck round-bottom flask was charged with *p*TsOH (5.45 g, 31.7 mmol, 7.5 eq.) in MeCN (24 mL). A solution of **28** (2.23 g, 4.22 mmol, 1.0 eq.) in MeCN (22 mL) was added at room temperature, and the resulting suspension was cooled to 10 °C. Afterward, a solution of $NaNO_2$ (891 mg, 12.7 mmol, 3.0 eq.) and KI (2.65 g, 15.8 mmol, 3.75 eq.) in water (14 mL) was added dropwise over 5 min (gas evolution) at 4-5 °C. After additional stirring for 30 min. at 10 °C, the solution was allowed to reach room temperature and stirred for 2 h. After the reaction was completed according to TLC, the reaction was quenched with a mixture of H_2O (260 mL), sat. aq. $NaHCO^3$-sol. (160 mL) and sat. aq. $Na_2S_2O_3$-sol. (160 mL). The aqueous phase was extracted with EtOAc (2 x 250 mL), and the combined organic phases were washed with brine (200 mL), dried over Na_2SO_4, concentrated under reduced pressure, and subjected to column chromatography (340 g SiO_2, Cy:EtOAc 98:2->85:15). Compound **29** (2.30 g, 3.60mol, 85%) was obtained as a colorless oil.

R_f = 0.30 (Cy/EtOAc, 10:1).

^{1}H NMR (500 MHz, CD_2Cl_2) δ = 8.00 (dd, *J*=8.0, 1.2, H_{Ar}, 1H), 7.82 (t, *J*=1.8, H_{Ar}, 1H), 7.59 (t, *J*=1.2, H_{Ar}, 1H), 7.57 (ddd, *J*=7.7, 1.7, 1.1, H_{Ar}, 1H), 7.54 (ddd, *J*=8.0, 2.0, 1.0, H_{Ar}, 1H), 7.45 (td, *J*=7.5, 1.2, H_{Ar}, 1H), 7.42 – 7.41 (m, H_{Ar}, 2H), 7.37 – 7.33 (m, H_{Ar}, 2H), 7.09 (ddd, *J*=8.0, 7.4, 1.7, H_{Ar}, 1H), 2.32 (t, *J*=7.0, CH_2, 2H), 1.70 – 1.61 (m, CH_2, 2H), 1.05 – 0.98 (m, 2 x iPr, 14H), 0.77 – 0.71 (m, CH_2, 2H).

^{13}C NMR (126 MHz, CD_2Cl_2) δ = 145.6 (1C, C_{Ar}), 144.1 (1C, C_{Ar}), 142.8 (1C, C_{Ar}), 142.4 (1C, C_{Ar}), 140.2 (1C, C_{Ar}), 134.8 (1C, C_{Ar}), 132.8 (1C, C_{Ar}), 131.1 (1C, C_{Ar}), 130.7 (1C, C_{Ar}), 130.6 (1C, C_{Ar}), 130.3 (1C, C_{Ar}), 129.8 (1C, C_{Ar}), 129.5 (1C, C_{Ar}), 128.9 (1C, C_{Ar}), 128.6 (1C, C_{Ar}), 122.4 (1C, C_{Ar}), 121.8 (1C, C_{Ar}), 120.3 (1C, CN), 106.8 (1C, C_{Ar}), 98.6 (1C, $C_{alkyine}$), 94.4 (1C, $C_{alkyine}$), 21.7 (1C, CH_2), 21.2 (1C, CH_2), 18.5 (2C, CH_3), 18.2 (2C, CH_3), 12.2 (2C, CH), 10.0 (1C, CH_2).

HR-MS (ESI, MeOH) calcd. For $C_{30}H_{31}BrINNaSi^+$: [M + Na]$^+$, 662.0346; found [M + Na]$^+$, 662.0343.

4-(2-Amino-4-bromophenyl)-2-methylbut-3-yn-2-ol (31)

An oven-dried and argon flushed Schlenk tube was charged with 5-bromo-2-iodoaniline (**30**, 5.00 g, 16.8 mmol, 1.0 eq), dry THF (30 mL), and piperidine (10 mL), and the yellow mixture degassed by passing argon through for 10 min. Then HOP-acetylene (2.0 mL, 20.2 mmol, 1.2 eq.) was added, and the reaction mixture was again bubbled for 5 min. and then $(Ph_3P)_2PdCl_2$ (596 mg, 840 µmol, 0.05 eq.) and CuI (193 mg, 1.01 µmol, 0.06 eq.) were added. The exothermic mixture was degassed by passing argon through for a further 5 min. The yellow suspension was stirred at room temperature for 1 h until it was no longer exothermic. After the reaction was completed according to TLC and GC-MS, the solution was diluted by the addition of EtOAc (200 mL). The organic layer was washed with water (100 mL) and brine (100 mL) and dried over Na_2SO_4. The organic phase was concentrated under reduced pressure and subjected to column chromatography (680 g SiO_2, Cy:EtOAc 92:8->45:55). Compound **31** (3.80 g, 15.0 mmol, 89%) was obtained as brown solid.

R_f = 0.21 (Cy/EtOAc, 2:1).

Mp: 95 °C

^{1}H NMR (500 MHz, CD_2Cl_2) δ = 7.09 (d, *J*=8.2, H_{Ar}, 1H), 6.86 (d, *J*=1.9, H_{Ar}, 1H), 6.78 (dd, *J*=8.2, 1.8, H_{Ar}, 1H), 4.29 (s, NH_2, 2H), 2.15 (s, OH 1H), 1.60 (s, 2 x CH_3, 6H).

^{13}C NMR (126 MHz, CD_2Cl_2) δ = 149.6 (1C, C_{Ar}), 133.7 (1C, C_{Ar}), 123.8 (1C, C_{Ar}), 121.1 (1C, C_{Ar}), 117.3 (1C, C_{Ar}), 106.8 (1C, C_{Ar}), 101.1 (1C, $C_{alkyine}$), 78.0 (1C, $C_{alkyine}$), 66.2 (1C, C_{tert}), 32.0 (2C, CH_3).

HR-MS (ESI, MeOH) calcd. For $C_{11}H_{12}BrNNaO^+$: [M + Na]$^+$, 275.9994; found [M + Na]$^+$, 275.9993.

4-(((3-Amino- 4- (3- hydroxy- 3- methylbut- 1- yn -1 -yl) phenyl) ethynyl) diisopropylsilyl) butanenitrile (32)

An oven-dried and argon flushed Schlenk tube was charged with **31** (3.52 g, 13.9 mmol, 1.0 eq), dry THF (21 mL), and piperidine (7.0 mL), and the mixture degassed by passing argon through for 10 min. CPDIPS-acetylene (4.9 mL, 20.9 mmol, 1.5 eq.) was added, and the reaction mixture was again bubbled for 5 min. and then $(Ph_3P)_2PdCl_2$ (493 mg, 695 µmol, 0.05 eq.) and CuI (162 mg, 834 µmol, 0.06 eq.) were added. The mixture was degassed by passing argon through for a further 5 min. The yellow suspension was refluxed for 1 h. After the reaction was completed according to TLC and GC-MS, the solution was diluted by the addition of EtOAc (200 mL). The organic layer was washed with water (100 mL) and brine (100 mL) and dried over Na_2SO_4. The organic phase was concentrated under reduced pressure and subjected to column chromatography (640 g SiO_2, Cy:EtOAc 92:8->40:60). Compound **32** (4.61 g, 12.1 mmol, 87%) was obtained as an orange oil.

R_f = 0.28 (Cy/EtOAc, 1:1).

^{1}H NMR (500 MHz, CD_2Cl_2) δ = 7.17 (dd, *J*=7.9, 0.5, H_{Ar}, 1H), 6.81 (dd, *J*=1.5, 0.4, H_{Ar}, 1H), 6.77 (dd, *J*=7.9, 1.5, H_{Ar}, 1H), 4.09 (s, NH_2, 2H), 2.42 (t, *J*=7.0, NH_2, 2H), 1.88 – 1.80 (m, CH_2, 2H), 1.61 (s, 2 x CH_3, 6H), 1.14 – 1.06 (m, 2 x iPr, 14H), 0.85 – 0.79 (m, CH_2, 2H).

^{13}C NMR (126 MHz, CD_2Cl_2) δ = 148.2 (1C, C_{Ar}), 132.5 (1C, C_{Ar}), 124.4 (1C, C_{Ar}), 121.8 (1C, C_{Ar}), 120.4 (1C, CN), 117.7 (1C, C_{Ar}), 108.4 (1C, C_{Ar}), 108.2 (1C, $C_{alkyine}$), 101.8 (1C, $C_{alkyine}$), 90.9 (1C, $C_{alkyine}$), 78.5 (1C, $C_{alkyine}$), 66.2 (1C, C_{tert}), 32.0 (2C, CH_3), 21.9 (1C, CH_2), 21.2 (1C, CH_2), 18.5 (2C, CH_3), 18.3 (2C, CH_3), 12.3 (2C, CH), 10.1 (1C, CH_2).

HR-MS (ESI, MeOH) calcd. For $C_{23}H_{32}N_2NaOSi^+$: $[M + Na]^+$, 403.2176; found $[M + Na]^+$, 403.2182.

4- (((4- (3- Hydroxy- 3- methylbut- 1- yn- 1- yl)- 3- iodophenyl) ethynyl) diisopropylsilyl) butanenitrile (33)

An oven-dried and argon-flushed three-neck round-bottom flask was charged with *p*TsOH (15.6 g, 90.8 mmol, 7.5 eq.) in MeCN (65 mL). A solution of **32** (4.6 g, 12.1 mmol, 1.0 eq.) in MeCN (55 mL) was added at room temperature, and the resulting suspension was cooled to 10 °C. A solution of $NaNO_2$ (2.56 mg, 36.3 mmol, 3.0 eq.) and KI (7.61 g, 45.4 mmol, 3.75 eq.) in water (33 mL) was added dropwise over 5 min (gas evolution) at 4-5 °C. After additional stirring for 30 min. at 10 °C, the solution was allowed to reach room temperature and stirred for 13 h. After the reaction was completed according to TLC and GC-MS, the reaction was quenched with a mixture of H_2O (250 mL), sat. aq. $NaHCO_3$-sol. (150 mL) and sat. aq. $Na_2S_2O_3$-sol. (150 mL). The aqueous phase was extracted with EtOAc (2 x 250 mL), and the combined organic phases were washed with brine (150 mL) and dried over Na_2SO_4. The organic phase was concentrated under reduced pressure and subjected to column chromatography (680 g SiO_2, Cy:EtOAc 92:8->55:45). Compound **33** (3.55 g, 7.22 mmol, 60%) was obtained as a yellow oil.

R_f = 0.23 (Cy/EtOAc, 3:1).

^{1}H NMR (500 MHz, CD_2Cl_2) δ = 7.96 (dd, *J*=1.5, 0.4, H_{Ar}, 1H), 7.40 (dd, *J*=8.0, 1.6, H_{Ar}, 1H), 7.36 (dd, *J*=8.0, 0.5, H_{Ar}, 1H), 2.42 (t, *J*=7.0, CH_2, 2H), 2.14 (s, OH, 1H), 1.88 – 1.79 (m, CH_2, 2H), 1.62 (s, 6H), 1.13 – 1.06 (m, 2 x iPr, 14H), 0.85 – 0.80 (m, CH_2, 2H).

^{13}C NMR (126 MHz, CD_2Cl_2) δ = 142.2 (1C, C_{Ar}), 132.3 (1C, C_{Ar}), 131.8 (1C, C_{Ar}), 130.0 (1C, C_{Ar}), 124.6 (1C, C_{Ar}), 120.3 (1C, CN), 105.8 (1C, C_{Ar}), 100.9 (1C, $C_{alkyine}$), 100.3 (1C, $C_{alkyine}$), 93.6 (1C, $C_{alkyine}$), 84.5 (1C, $C_{alkyine}$), 66.1 (1C, C_{tert}), 31.6 (2C, CH_3), 21.8 (1C, CH_2), 21.3 (1C, CH_2), 18.5 (2C, CH_3), 18.3 (2C, CH_3), 12.2 (2C, CH), 10.1 (1C, CH_2).

HR-MS (ESI, MeOH) calcd. For $C_{23}H_{30}INNaOSi^+$: $[M + Na]^+$, 514.1034; found $[M + Na]^+$, 514.1037.

4- (3- Ethynylphenyl)- 2- methylbut- 3- yn- 2- ol (C_2)

A two-neck round-bottom flask was cleaned by the following treatment to remove all copper-ions from previous reactions. The flask was filled with conc. H_2SO_4 and sonicated for 10 min, followed by washing with H_2O, NaOH (1*M*), H_2O, and acetone. The flask was dried in the heating oven overnight and flushed with argon. To a solution of 4- (((3- (3- hydroxy- 3- methylbut- 1- yn- 1-yl) phenyl) ethynyl) diisopropylsilyl) butanenitrile[5] (5.00 g, 13.7 mmol, 1.0 eq.) in THF (200 mL) was added TBAF (1 M in THF, 20.5 mL ,20.5 mmol, 1.5 eq) and the reaction mixture stirred at room temperature for 15 min under argon. The reaction was diluted by the addition of water (250 mL), and the aqueous phase was extracted with CH_2Cl_2 (500 mL). The organic layer was washed with water (250 mL) and brine (250 mL) and dried over Na_2SO_4, concentrated under reduced pressure, and subjected to column chromatography (750 g SiO_2, Cy:EtOAc 95:5->70:30). Compound **C_2** (2.52 g, 13.7 µmol, quant.) was obtained as a yellow oil.

R_f = 0.18 (Cy/EtOAc, 4:1).

^{1}H NMR (500 MHz, CD_2Cl_2) δ = 7.54 (td, *J*=1.7, 0.6, H_{Ar}, 1H), 7.43 (dt, *J*=7.7, 1.5, H_{Ar}, 1H), 7.40 (dt, *J*=7.8, 1.4, H_{Ar}, 1H), 7.29 (td, *J*=7.8, 0.6, H_{Ar}, 1H), 3.15 (s, $H_{alkyine}$, 1H), 2.19 (s, OH, 1H), 1.59 (s, 2 x CH_3, 6H).

^{13}C NMR (126 MHz, CD_2Cl_2) δ = 135.5 (1C, C_{Ar}), 132.4 (1C, C_{Ar}), 132.3 (1C, C_{Ar}), 129.0 (1C, C_{Ar}), 123.8 (1C, C_{Ar}), 122.9 (1C, C_{Ar}), 95.4 (1C, $C_{alkyine}$), 83.1 (1C, $C_{alkyine}$), 81.3 (1C, $C_{alkyine}$), 78.3 (1C, $C_{alkyine}$), 65.9 (1C, C_{tert}), 31.8 (2C, CH_3).

HR-MS (ESI, MeOH) calcd. for $C_{13}H_{12}NaO^+$: $[M + Na]^+$, 207.0780; found $[M + Na]^+$, 207.0783.

3. Further Information on Compound **1** and Compound **41**

3.1 Measured NMR Signals of Compound **1**

Figure 1: 1 was fully characterized by ^{1}H- and ^{13}C-NMR spectroscopy.

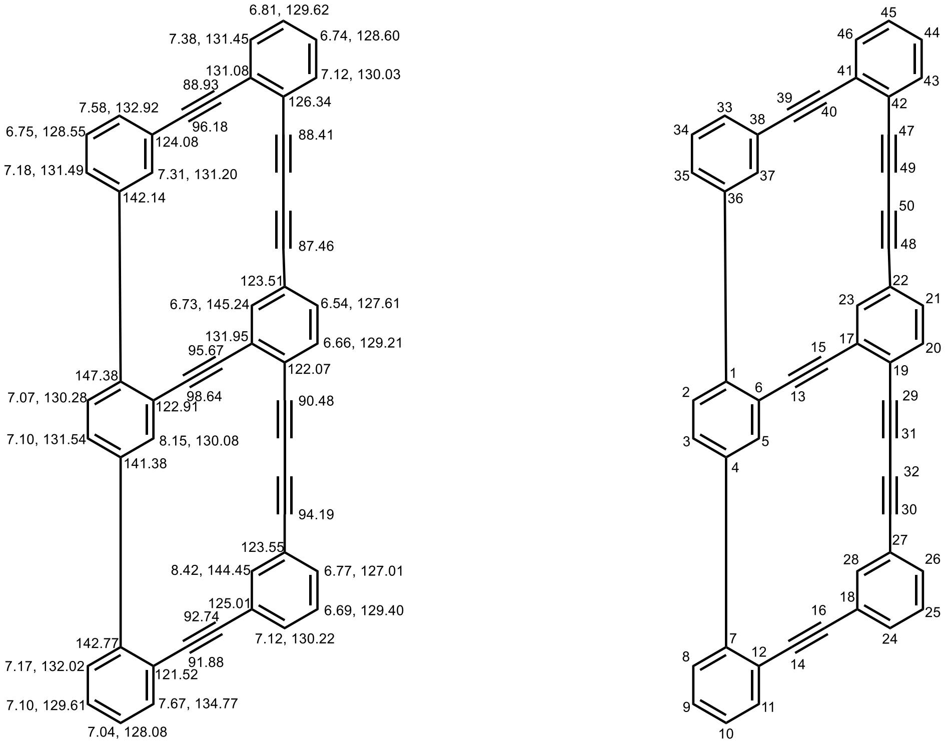

3.2 Calculated NMR Signals of Compound **1**

To assign the missing inner acetylene signals, thenmolecule was calculated with Spartan (’18 V 1.2.0, DFT, B3LYP/6-31G*) and compared to the measured 1H and 13C NMR values.

1H or 13C Number	Measured C	Calculated C	Measured H	Calculated H	Difference C	Absolute C	Difference H	Absolute H
49		86,1			-86,1	86,1	0	0
31		87,8			-87,8	87,8	0	0
32		87,8			-87,8	87,8	0	0
50		88,2			-88,2	88,2	0	0
48	87,46	91,3			-3,84	3,84	0	0
47	88,41	92,6			-4,19	4,19	0	0
40	88,93	93,9			-4,97	4,97	0	0
29	90,48	94,5			-4,02	4,02	0	0
14	91,88	96,2			-4,32	4,32	0	0
16	92,74	96,6			-3,86	3,86	0	0
30	94,19	97,6			-3,41	3,41	0	0
15	95,67	99,7			-4,03	4,03	0	0
39	96,18	100,3			-4,12	4,12	0	0
13	98,64	101			-2,36	2,36	0	0
12	121,52	121,6			-0,08	0,08	0	0
19	122,07	122,2			-0,13	0,13	0	0
6	122,91	123,5			-0,59	0,59	0	0
22	123,51	123,9			-0,39	0,39	0	0
38	124,08	124,3			-0,22	0,22	0	0
27	123,55	124,9			-1,35	1,35	0	0
18	125,01	125,2			-0,19	0,19	0	0
26	127,01	125,4	6,77	6,8	1,61	1,61	-0,03	0,03
21	127,61	125,7	6,54	6,6	1,91	1,91	-0,06	0,06
44	128,6	126,2	6,74	7,1	2,4	2,4	-0,36	0,36
10	128,08	126,4	7,04	7,2	1,68	1,68	-0,16	0,16
34	128,55	126,8	6,75	7,2	1,75	1,75	-0,45	0,45
45	129,62	127	6,81	7,1	2,62	2,62	-0,29	0,29
42	126,34	127,2			-0,86	0,86	0	0
9	129,61	127,4	7,1	7,2	2,21	2,21	-0,1	0,1
25	129,4	127,5	6,69	7,1	1,9	1,9	-0,41	0,41
20	129,21	128,5	6,66	6,8	0,71	0,71	-0,14	0,14
24	130,22	128,8	7,12	7,1	1,42	1,42	0,02	0,02
43	130,03	129,8	7,12	7,1	0,23	0,23	0,02	0,02
2	130,28	129,9	7,07	7,2	0,38	0,38	-0,13	0,13
3	131,54	130	7,1	7,2	1,54	1,54	-0,1	0,1
37	131,2	130,4	7,31	7,3	0,8	0,8	0,01	0,01
35	131,49	130,5	7,18	7,4	0,99	0,99	-0,22	0,22
5	130,08	130,6	8,15	7,9	-0,52	0,52	0,25	0,25
8	132,02	131,2	7,17	7,2	0,82	0,82	-0,03	0,03

46	131,45	131,4	7,38	7,3	0,05	0,05	0,08	0,08
41	131,08	132,1			-1,02	1,02	0	0
33	132,92	133	7,58	7,5	-0,08	0,08	0,08	0,08
17	131,95	133,2			-1,25	1,25	0	0
11	134,77	134,9	7,67	7,5	-0,13	0,13	0,17	0,17
4	141,38	141,5			-0,12	0,12	0	0
36	142,14	142,3			-0,16	0,16	0	0
7	142,77	143,2			-0,43	0,43	0	0
28	144,45	147	8,42	8	-2,55	2,55	0,42	0,42
23	145,24	148,1	6,73	6,8	-2,86	2,86	-0,07	0,07
1	147,38	148,2			-0,82	0,82	0	0

The values are ordered by the calculated carbon shifts. As seen, the calculated values for all acetylenes fits well, and the order of the signal is in agreement with the measured ones.

1H or 13C Number	Measured C	Calculated C	Measured H	Calculated H	Difference C	Absolute C	Difference H	Absolute H
49	80,6	86,1			-5,5	5,5	0	0
31	81,5	87,8			-6,3	6,3	0	0
32	81,9	87,8			-5,9	5,9	0	0
50	82,8	88,2			-5,4	5,4	0	0

3.3 NOEs comparison with DFT structures

	d/Å in **1b**	NOE	d/Å in **1a**
H5-H28	2.7	a	2.7
H11-H24	4.3	b	4.6
H5-H8	4.5	c	4.3
H5-H11	4.8	d	4.9
H2-H37	2.6	e	2.8
H23-H33	3.5	f	3.8
H23-H37	3.7	g	3.7
H23-H35	3.3	h	3.9
H3-H8	2.2	i	2.4
H2-H35	4.2	k	4.1
H23-H34	3.3		3.9

Correlation between ^{13}C shift difference of alkyne carbon atoms with strain

	triple bond	13C shift difference in ppm
	□(C47-C49)	7.8
	□(C48-C50)	4.7
	□(C29-C31)	9.0
	□(C30-C32)	12.3

Compound **1a**

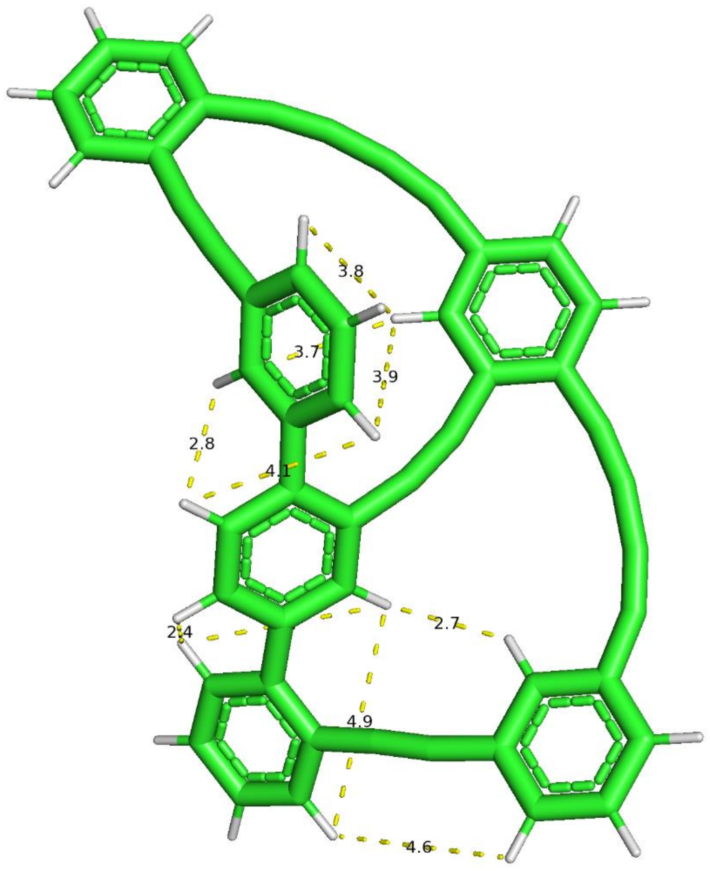

Compound **1b**

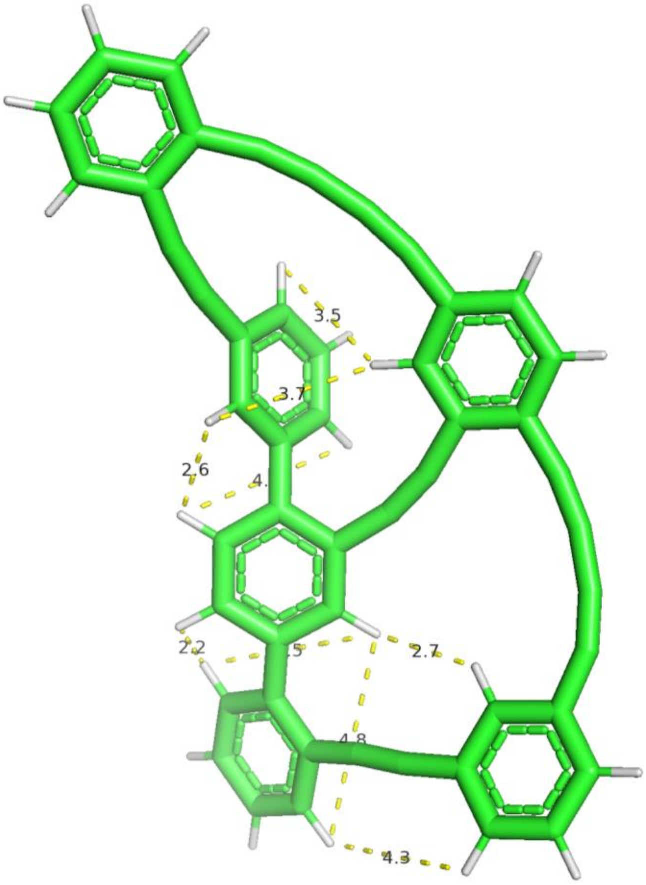

3.4 DOSY of Compound **1** and Compound **41**

The diffusion coefficient of **1** in C_6D_6 was determined as 6.34(1) x 10^{-10} m^2/s while the reaction products **41** of the oxidative dimerization showed a significantly lower diffusion coefficient of 4.28(1) x 10^{-10} m^2/s. In a first approximation of spherical moieties, the volume of the later one including the first shell of solvation was calculated via the Stokes Einstein equation to be 3.25 times the volume of **1**, which is in reasonable agreement with the suggested dimeric structure for **41**.

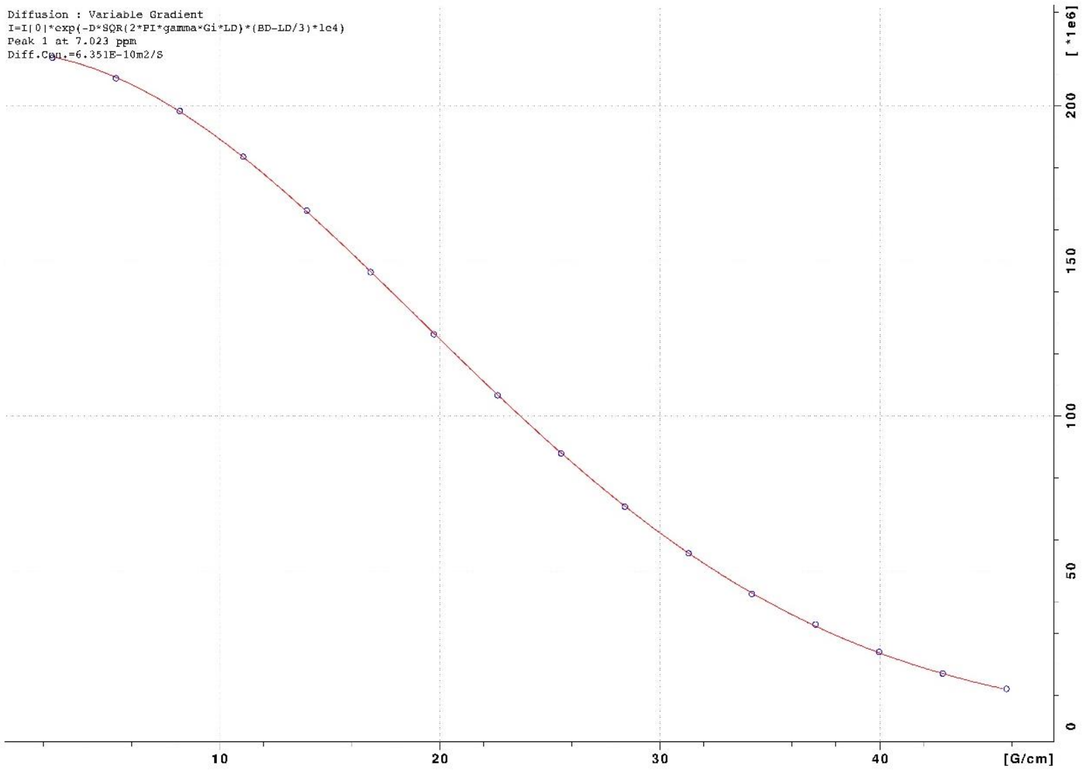

DOESY of compound **1,** (in C_6D_6, 298 K, 600 MHz)

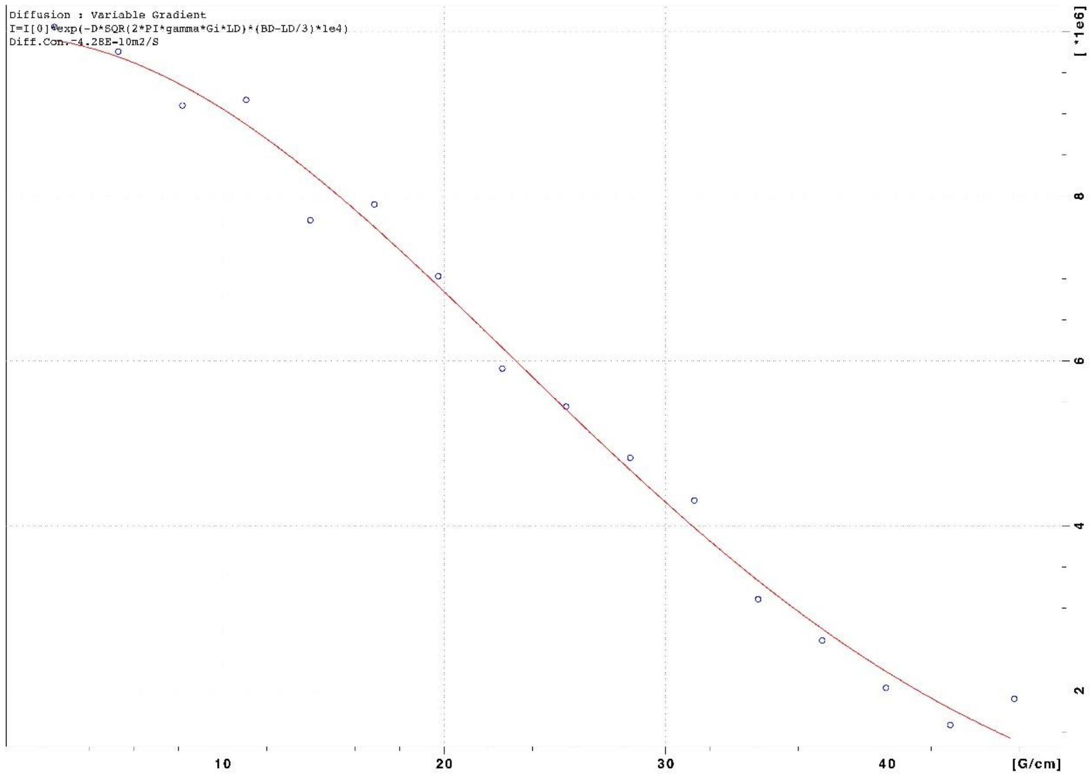

DOESY of compound **41,** (in C_6D_6, 298 K, 600 MHz)

3.5 HMBC of Compound **41**

Carbonyl area of Compond **41** in the HMBC.

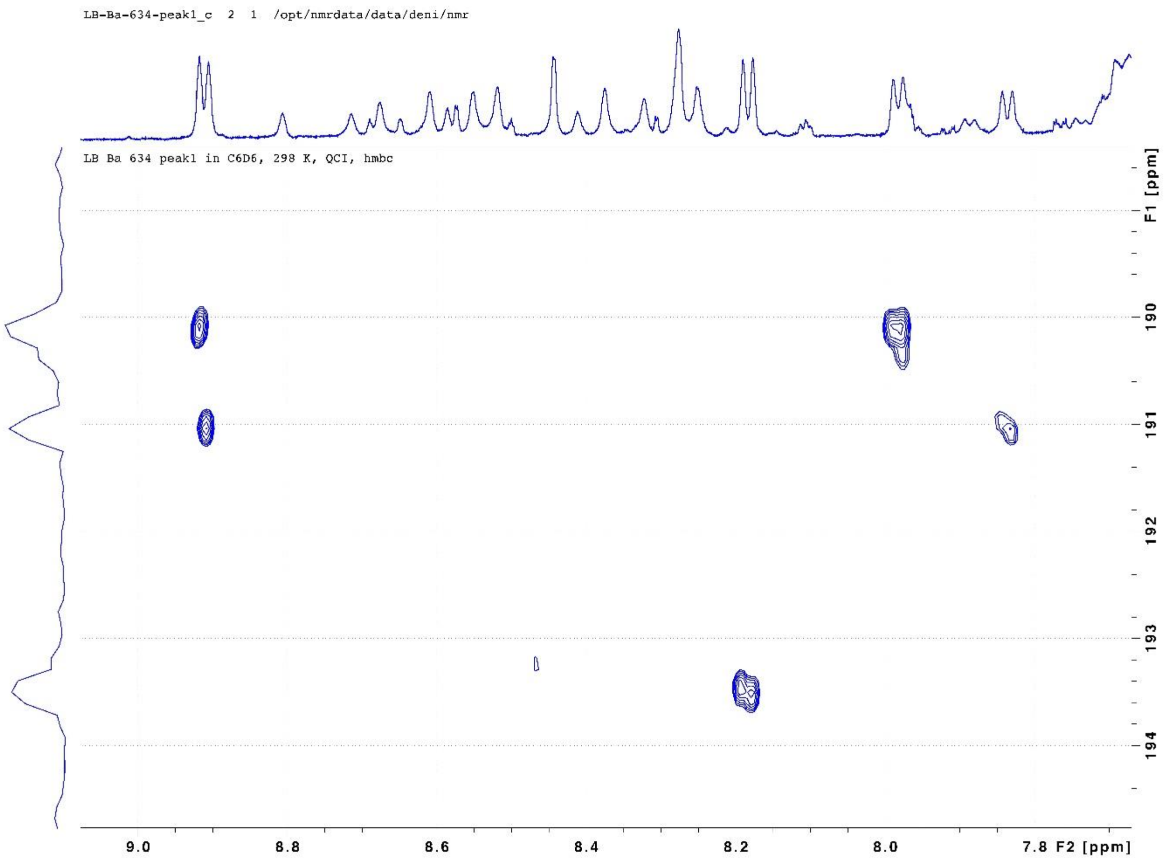

3.6 Three Possible Structures of Compound **41**

Thr carbonyl area of compond **41** in the HMBC sugested a mixture of three compounds with two carbonyls each. In analogy to the macrocyclic model compound **2**, for which regioselectively one of both acetylenes of the diacetylene bridge was engaged in the oxidative dimerization.[5]

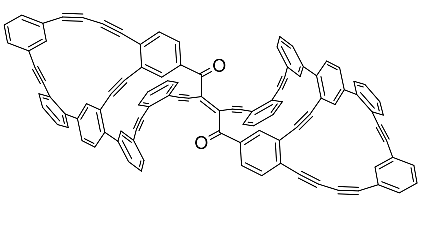

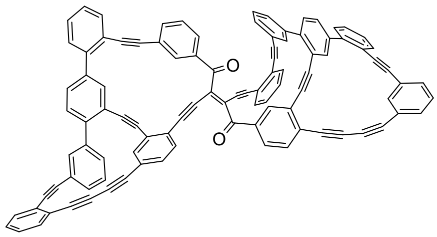

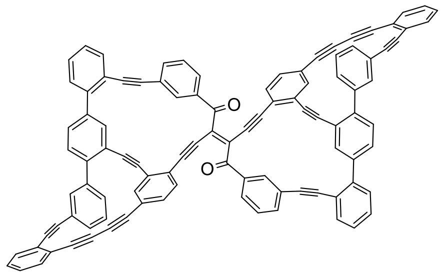

41a **41b** **41c**

Three possible structures of compound **41**. **41a** and **41c** are to two symmetric structures, while compound **41b** is the asymmetric.

3.7 HOMO and LUMO of Compound **1a** and **1b**

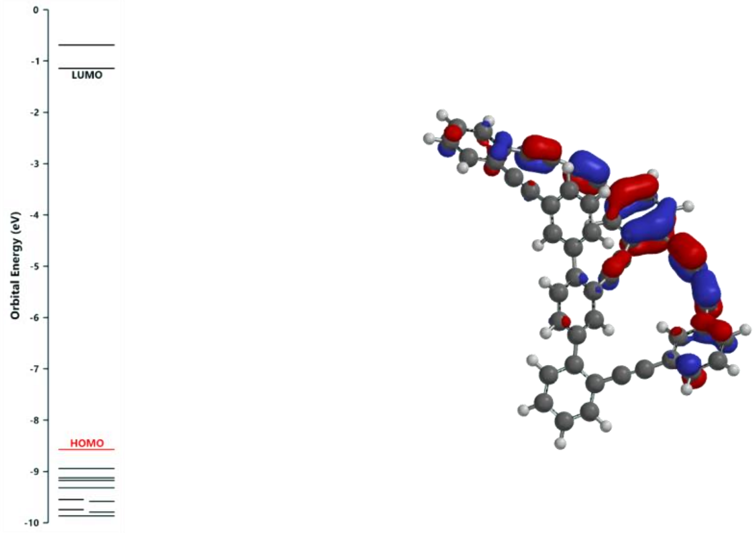

Homo of Compound **1a** calculated with Spartan (’18 V 1.2.0, DFT, B3LYP/6-31G*)

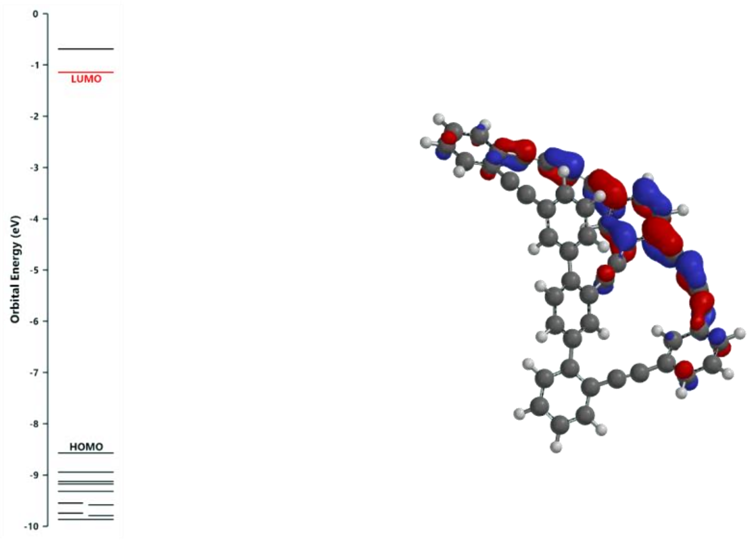

LUMO of Compound **1a** calculated with Spartan (’18 V 1.2.0, DFT, B3LYP/6-31G*)

-0.70 eV, -1.1 eV (LUMO), -8.6 eV (HOMO), -8.9 eV, -9.1 eV, -9.2 eV, -9.3 eV, -9.5 eV, -9.6 eV, -9.7 eV, -9.8 eV, -9.9 eV

For compound **1a** the HOMO-LUMO gap is 7.5 eV

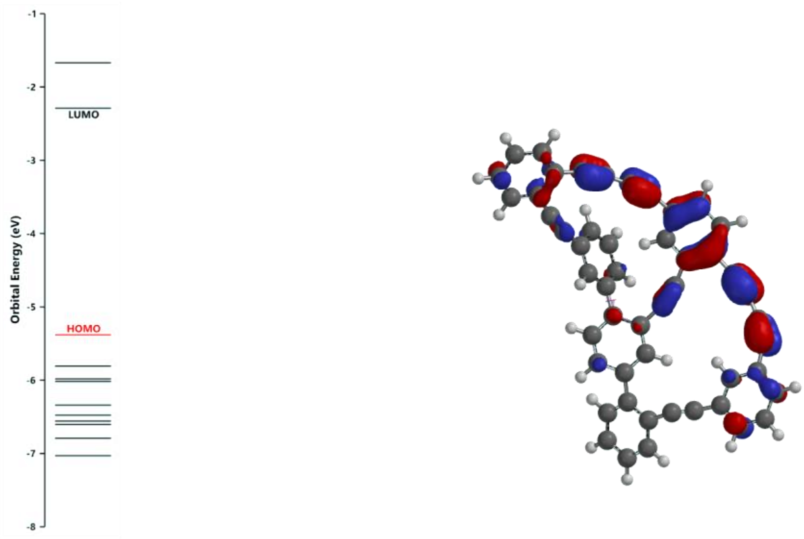

HOMO of Compound **1b** calculated with Spartan (’18 V 1.2.0, DFT, B3LYP/6-31G*)

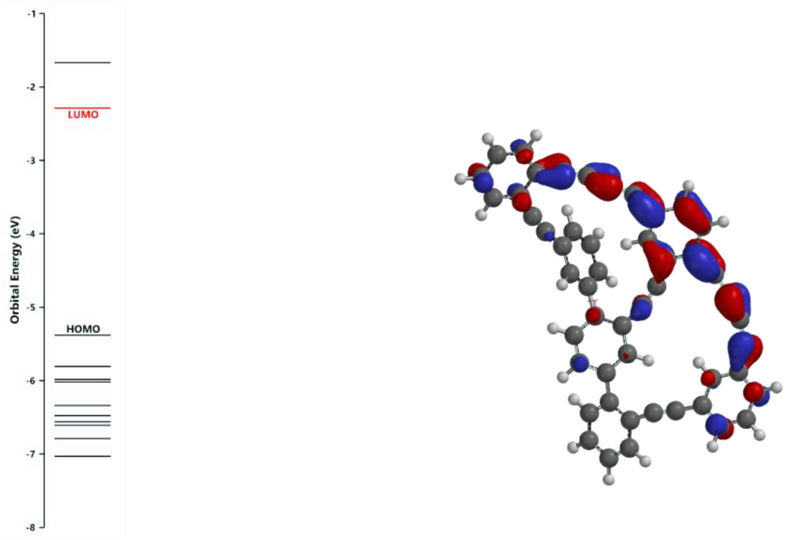

LUMO of Compound **1b** calculated with Spartan (’18 V 1.2.0, DFT, B3LYP/6-31G*)

-1.70 eV, -2.3 eV (LUMO), -5.4 eV (HOMO), -5.8 eV, -6.0 eV, -6.0 eV, -6.3 eV, -6.5 eV, -6.6 eV, -6.6 eV, -6.8 eV, -7.0 eV

For compound **1b** the HOMO-LUMO gap is 3.1 eV.

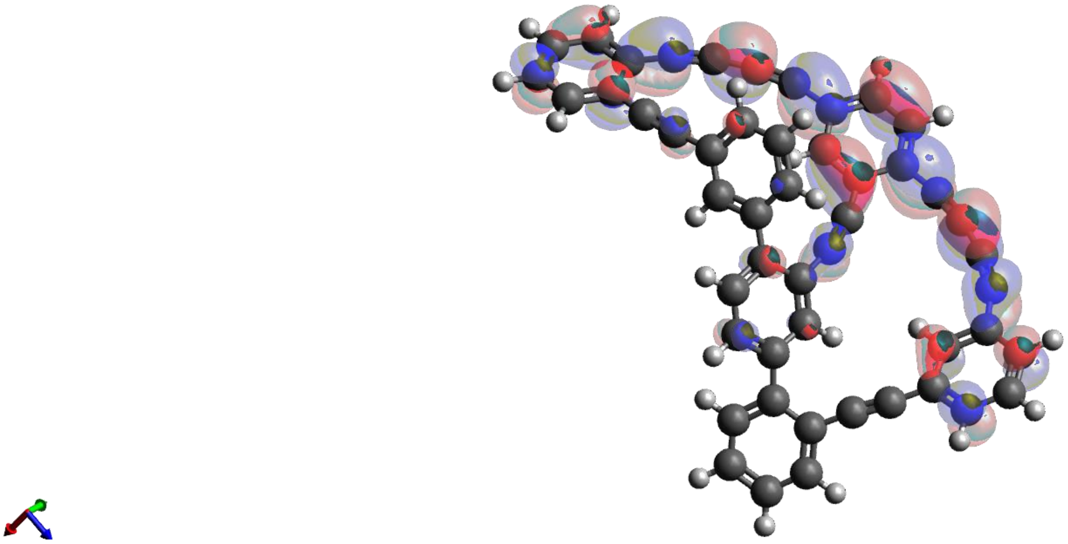

Homo of Compound **1a** calculated with B3LYP RIJCOSX D3BJ def2-TZVP def2/J TIGHTSCF Opt NumFreq Grid5 FinalGrid6

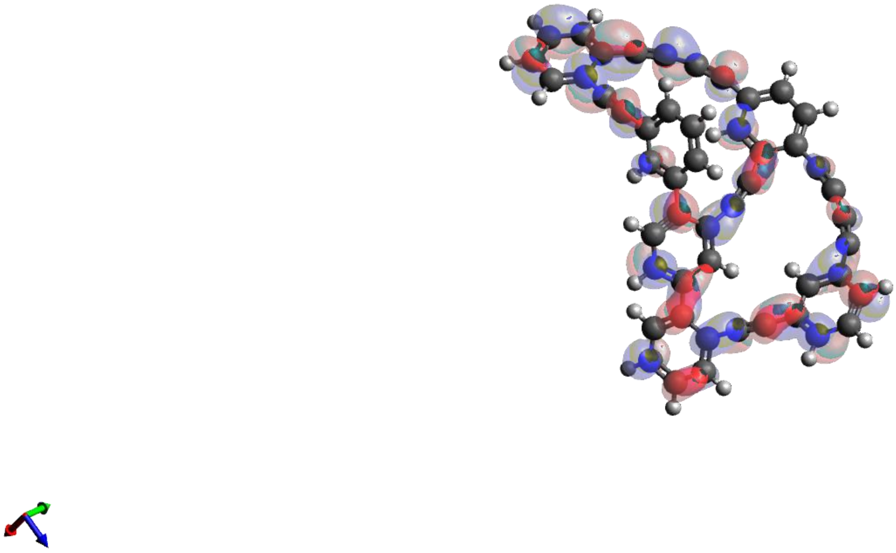

Lumo of Compound **1a** calculated with B3LYP RIJCOSX D3BJ def2-TZVP def2/J TIGHTSCF Opt NumFreq Grid5 FinalGrid6

-5.6581 eV (HOMO, -0.20793), -2.4871 (LUMO, -0.0914 Ha)

For compound **1a** the HOMO-LUMO gap is 3.1710 eV.

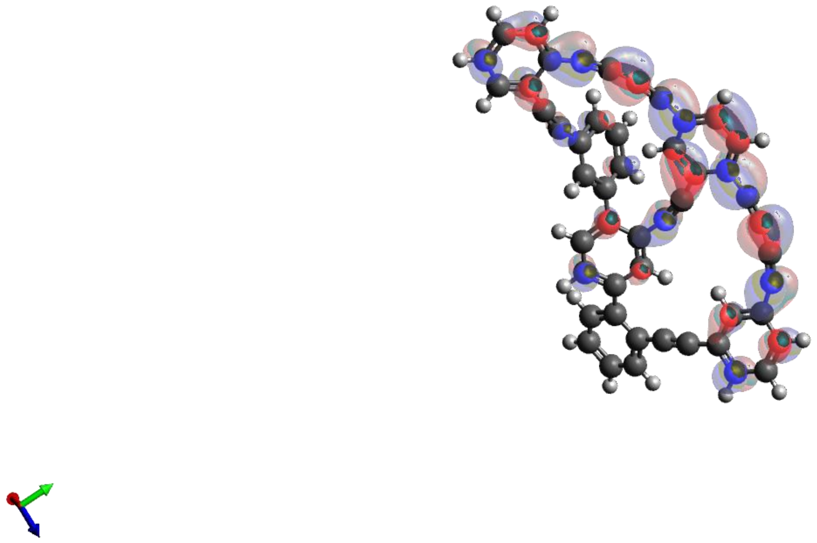

Homo of Compound **1b** calculated with B3LYP RIJCOSX D3BJ def2-TZVP def2/J TIGHTSCF Opt NumFreq Grid5 FinalGrid6

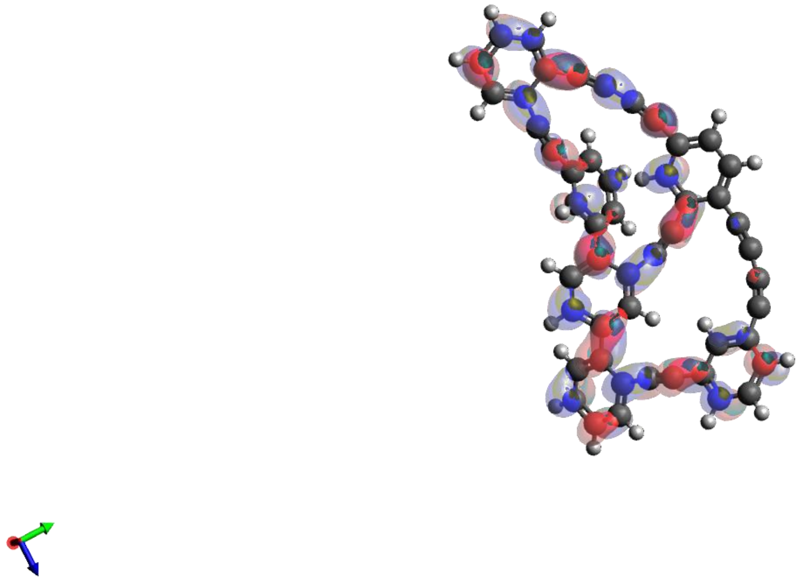

Lumo of Compound **1b** calculated with B3LYP RIJCOSX D3BJ def2-TZVP def2/J TIGHTSCF Opt NumFreq Grid5 FinalGrid6

-5.6150 eV (HOMO, -0.206347), -2.5288 (LUMO, -0.09293 Ha)

For compound **1b** the HOMO-LUMO gap is 3.0862 eV.

4. Dynamic HPLC

Elution profiles for the dynamic HPLC experiment of Compound **1** (Chiralpack IA, *n*heptane:*i*PrOH 98:2, 1 mL/min, column oven: 15-25 °C). All experiments were repeated three time at each temp. All chromatograms were analyzed by DCXploter.[6–8] From the first chromatogram, the time from 10-15 min. is shown below.

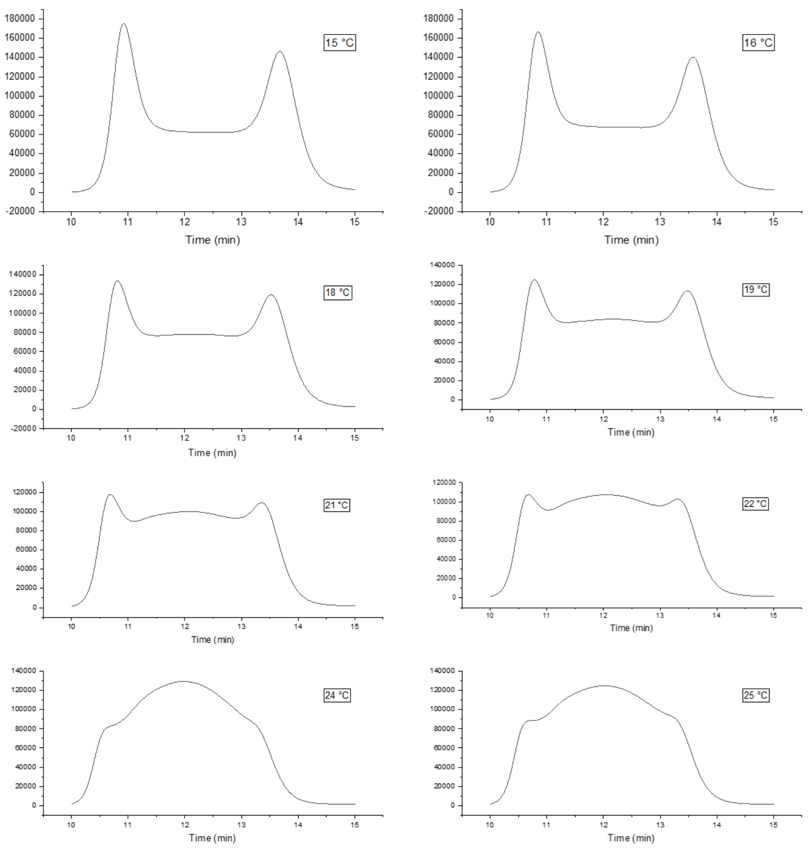

Figure 2: Elution profiles obtained from the dynamic HPLC experiment, from 15-25 °C.

The skipping rope displayed in the TOC graphic is the eluation profile of compound **1** at 19°C.

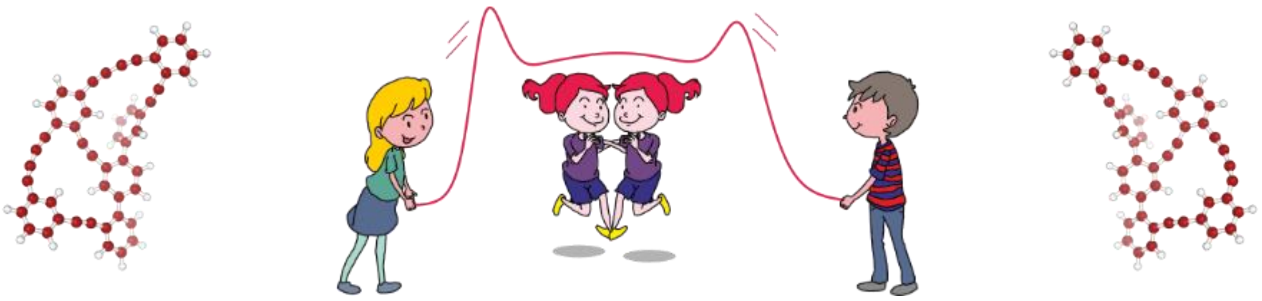

4.1 Data evaluation with DCXplorer

The rate constants are obtained directly from the parameters given by the DCXplorer by Oliver Trapp. For 24 and 25 °C no rate constant could be calculated. The shown error is the *"standard error of the mean value"* calculated with Origin.

25°C

No rate constant calculated!

24°C

No rate constant calculated!

23°C

tR1 [min]	tR2 [min]	wh1 [s]	wh2 [s]	h1 [%]	hp [%]	h2 [%]	A1	A2	k1 [1/s]
10.667	13.227	30.7	49.9	85.43	100.00	84.55	47.11	52.89	2.593E-3
10.677	13.205	32.0	49.9	84.45	100.00	83.85	47.20	52.80	2.528E-3
10.677	13.195	32.0	49.9	84.12	100.00	84.21	46.73	53.27	2.225E-3
									2.449E-3 ±1.13E-4

22°C

tR1 [min]	tR2 [min]	wh1 [s]	wh2 [s]	h1 [%]	hp [%]	h2 [%]	A1	A2	k1 [1/s]
10.677	13.312	29.4	44.8	100.00	99.44	94.96	47.82	52.18	2.703E-3
10.677	13.280	29.4	46.1	99.10	100.00	95.10	47.37	52.63	2.678E-3
10.656	13.237	29.4	44.8	99.20	100.00	95.41	47.52	52.48	2.681E-3
									2.687E-3 ±7.88E-6

21°C

tR1 [min]	tR2 [min]	wh1 [s]	wh2 [s]	h1 [%]	hp [%]	h2 [%]	A1	A2	k1 [1/s]
10.688	13.355	28.2	43.5	100.00	84.41	92.05	48.25	51.75	2.418E-3
10.656	13.280	29.4	44.8	100.00	86.41	92.82	47.81	52.19	2.381E-3
10.656	13.269	28.2	43.5	100.00	85.92	93.52	47.81	52.19	2.418E-3
									2.406E-3 ±1.23E-5

20°C

tR1 [min]	tR2 [min]	wh1 [s]	wh2 [s]	h1 [%]	hp [%]	h2 [%]	A1	A2	k1 [1/s]
10.752	13.440	28.2	43.5	100.00	76.53	91.88	47.84	52.16	2.202E-3
10.709	13.408	26.9	42.2	100.00	71.51	89.17	48.50	51.50	2.170E-3
10.677	13.312	26.9	42.2	100.00	73.38	91.19	48.06	51.94	2.179E-3
									2.184E-3 ±9.53E-6

19°C

tR1 [min]	tR2 [min]	wh1 [s]	wh2 [s]	h1 [%]	hp [%]	h2 [%]	A1	A2	k1 [1/s]
10.784	13.483	28.2	43.5	100.00	66.46	89.85	48.18	51.82	1.965E-3
10.741	13.440	26.9	42.2	100.00	61.49	87.73	48.48	51.52	1.908E-3
10.709	13.355	26.9	42.2	100.00	63.31	89.40	47.93	52.07	1.920E-3
									1.931E-3 ±1.73E-5

18°C

tR1 [min]	tR2 [min]	wh1 [s]	wh2 [s]	h1 [%]	hp [%]	h2 [%]	A1	A2	k1 [1/s]
10.816	13.525	28.2	43.5	100.00	57.95	88.26	48.17	51.83	1.751E-3
10.763	13.483	26.9	41.0	100.00	52.95	86.21	48.42	51.58	1.690E-3

10.741	13.408	25.6	41.0	100.00	54.73	87.72	48.17	51.83	1.763E-3
									1.735E-3 ±2.26E-5

17 °C

tR1 [min]	tR2 [min]	wh1 [s]	wh2 [s]	h1 [%]	hp [%]	h2 [%]	A1	A2	k1 [1/s]
10.848	13.568	26.9	43.5	100.00	50.77	86.67	48.47	51.53	1.621E-3
10.795	13.515	26.9	41.0	100.00	46.18	84.46	48.86	51.14	1.516E-3
10.773	13.429	26.9	42.2	100.00	47.83	86.48	48.44	51.56	1.530E-3
									1.556E-3 ±3.29E-5

16 °C

tR1 [min]	tR2 [min]	wh1 [s]	wh2 [s]	h1 [%]	hp [%]	h2 [%]	A1	A2	k1 [1/s]
10.912	13.632	28.2	44.8	100.00	45.06	85.74	48.49	51.51	1.418E-3
10.848	13.579	26.9	41.0	100.00	40.26	83.54	49.13	50.87	1.356E-3
10.827	13.493	26.9	42.2	100.00	41.94	85.35	48.47	51.53	1.369E-3
									1.381E-3 ±1.89E-5

15°C

tR1 [min]	tR2 [min]	wh1 [s]	wh2 [s]	h1 [%]	hp [%]	h2 [%]	A1	A2	k1 [1/s]
10.944	13.675	28.2	44.8	100.00	40.51	84.83	48.72	51.28	1.301E-3
10.923	13.675	26.9	41.0	100.00	34.92	82.65	49.34	50.66	1.207E-3
10.880	13.557	26.9	42.2	100.00	37.09	84.01	49.08	50.92	1.245E-3
									1.251E-3 ±2.73E-5

The Gibbs free energy of racemization can be calculated directly through the Eyring equation.[9] R = gas constant, T = absolute temperature, k_B= Boltzmann constant, h = Planck's constant.

$$\Delta G^{\ddagger}_{293} = RT\left[\ln\left(\frac{k_B T}{h}\right) - \ln(k_e)\right] \quad (1)$$

$$\Delta G^{\ddagger}_{293} = 8.31 \cdot 10^{-3} \cdot 293\left[\ln\left(\frac{1.38 \cdot 10^{-23} \cdot 293}{6.63 \cdot 10^{-34}}\right) - \ln(2.184 \cdot 10^{-3})\right] = 86.60 \pm 0.01 \text{ kJ/mol}$$

For all elution profiles $A = ln\frac{k_eT}{T}$ is plotted against $\frac{1}{T}$ and the linear regression of the obtained graph ($y = ax + B$) gives access to the full thermodynamic analysis.

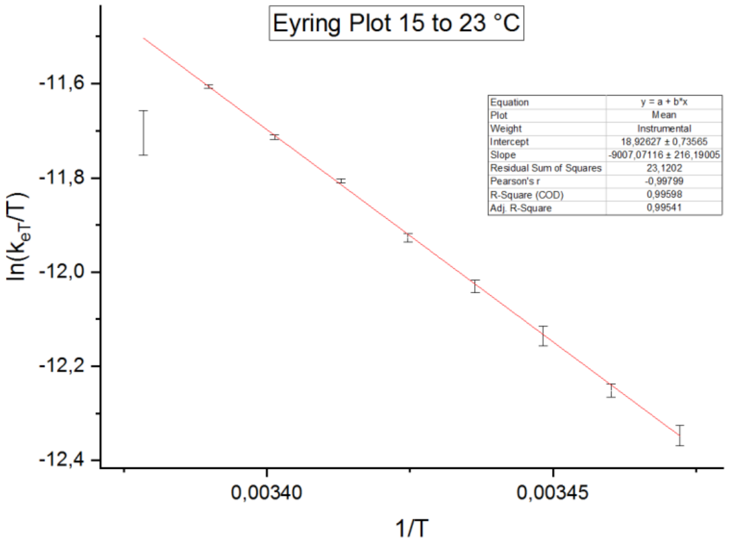

Figure 3: Eyring plot of $A = ln\frac{k_{eT}}{T}$ against $\frac{1}{T}$ with linear regression (($y = ax + B$) from 15 to 23 °C.

As the last value at 23 °C **(Figure 3)**, is no longer in the linear range, the value is not taken in consideration for the following calculations. The Eyring plot for 15 to 22 °C is more accurate **(Figure 4)**.

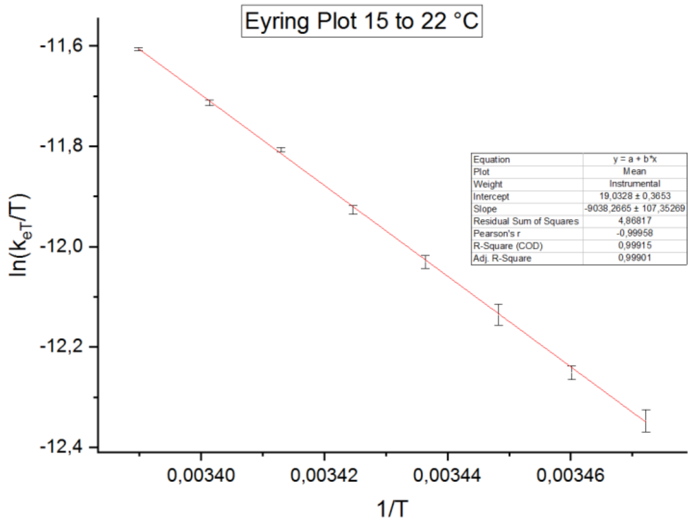

Figure 4: Eyring plot of $A = ln\frac{k_{eT}}{T}$ against $\frac{1}{T}$ with linear regression (($y = ax + B$) from 15 to 22 °C.

The entropy and enthalpy of the enantiomerization process can be calculated from that: a = slope, b = intercept

$$\Delta H_e^{\ddagger} = -aR \quad (2)$$

$$\Delta H_e^{\ddagger} = -(-9038.2665 \cdot 8.31 \cdot 10^{-3}) = 75.11 \pm 0.89\ kJ/mol$$

$$\Delta S_e^{\ddagger} = R \cdot \left[b - \ln(\frac{k_B}{h})\right] \quad (3)$$

$$\Delta S_e^{\ddagger} = 8.31 \cdot [19.0328 - 23.75891472] = -39.27 \pm 3.04\ J/(mol \cdot K)$$

$$\Delta G^{\ddagger}_{e\,293} = \Delta H^{\ddagger}_{e} - T\Delta S^{\ddagger}_{e} \qquad (4)$$

$\Delta G^{\ddagger}_{e\,293} = 75.11 - (293$ · (-39.27/1000)) =86.62 ± 1.78 kJ/mol

The value obtained directly from $k_{e\ 293\ K}$ is in good agreement with the value obtained from the full thermodynamical analysis with $\Delta\Delta G^{\ddagger}_{e\,293} = 0.02\ kJ/mol$.

The half-life of the pure enantiomer is given by:

$$t_{½} = \frac{ln2}{2k_e} \approx \frac{0.693147181}{2k_e} \qquad (5)$$

$$t_{½} = \frac{0.693147181}{2 \cdot 0.002184} = 158.7 \pm 0.69\ s = 2\min 39\ s$$

5. ^{1}H-, ^{13}C-, DEPT-135 NMR and HR-MS

5.1 Compound **5**

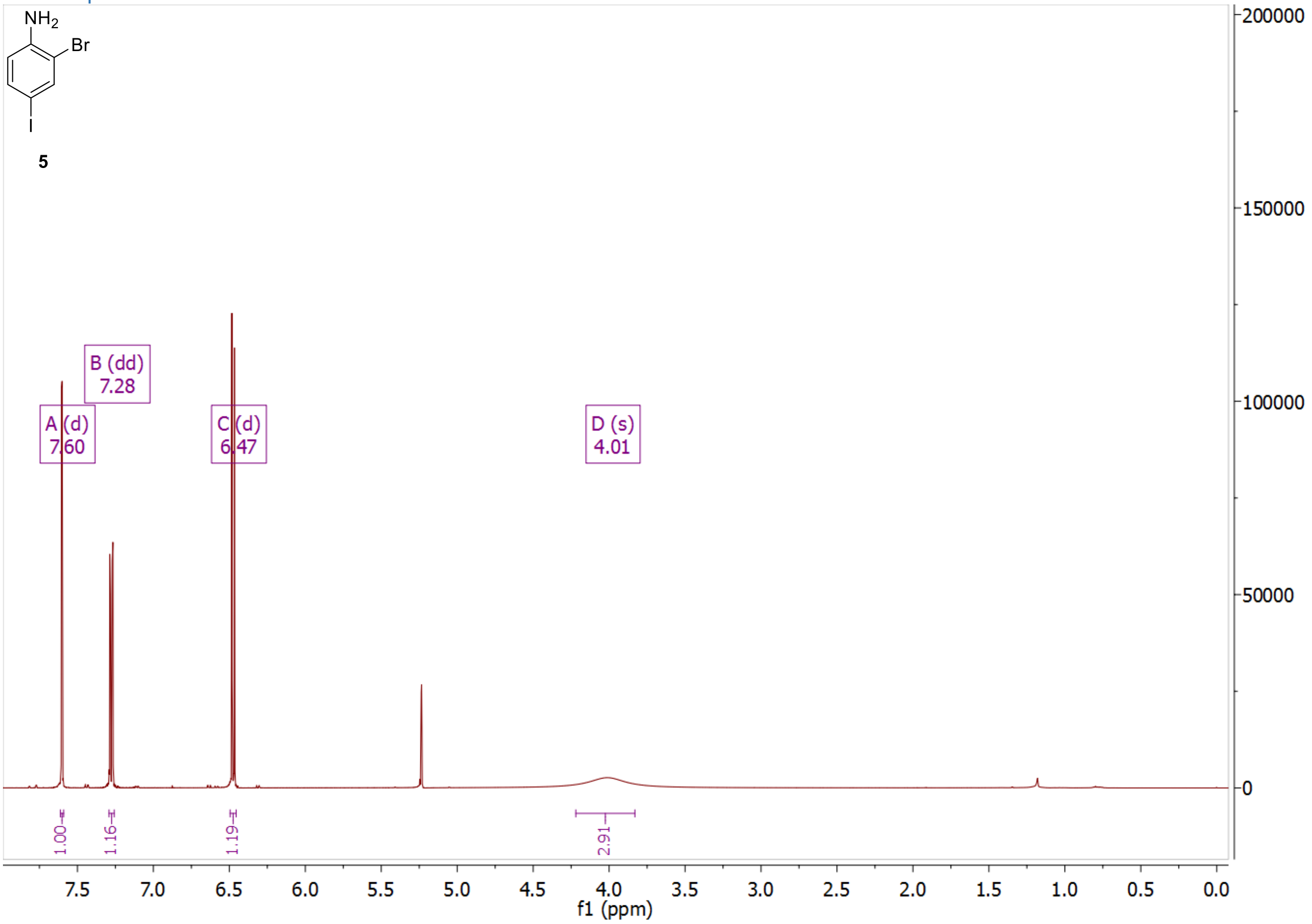

The spectra data of this compound was identical to those reported in the literature.[1]

5.2 Compound **6**

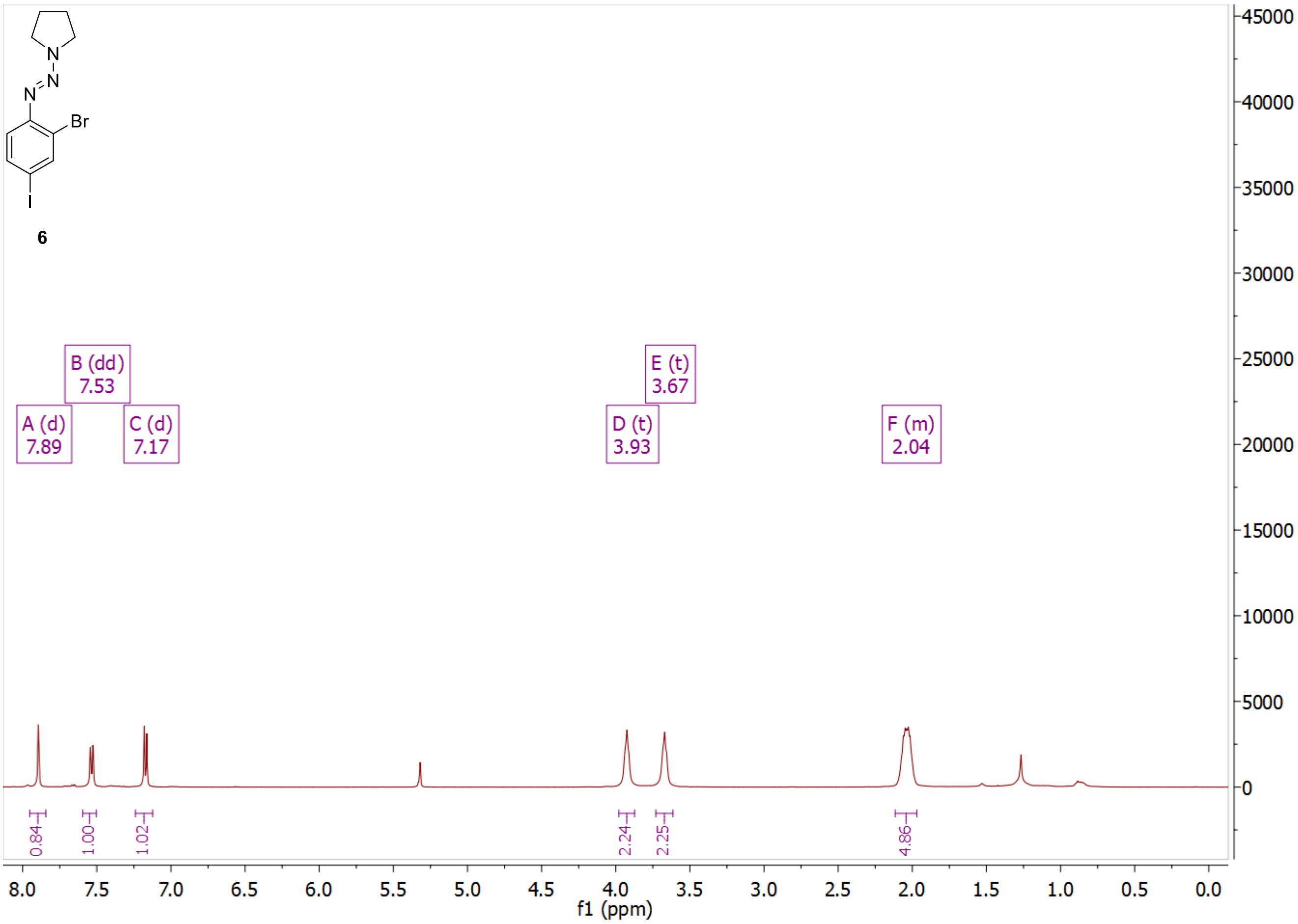

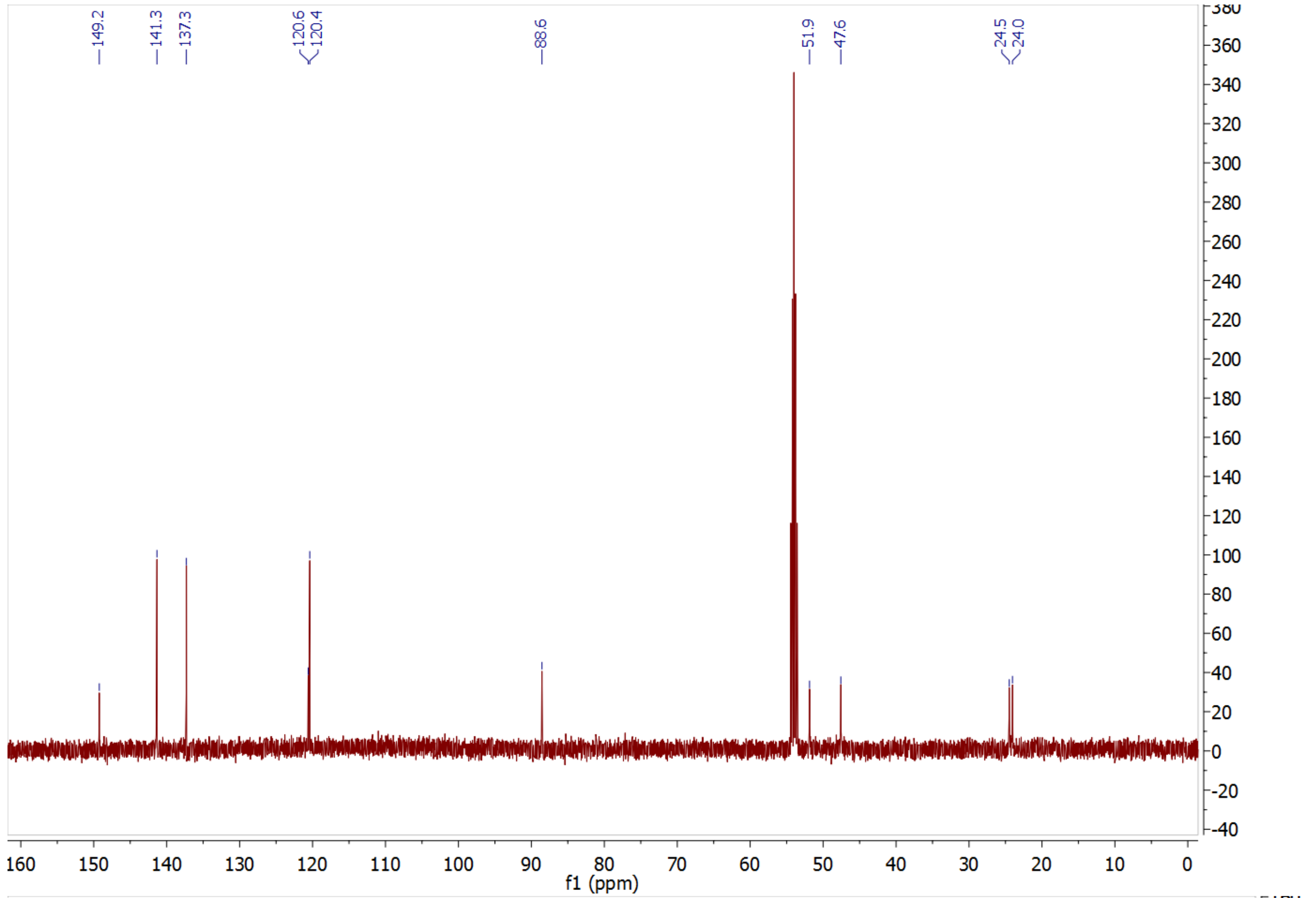
149.2
141.3
137.3
120.6
120.4
88.6
51.9
47.6
24.5
24.0
f1 (ppm)

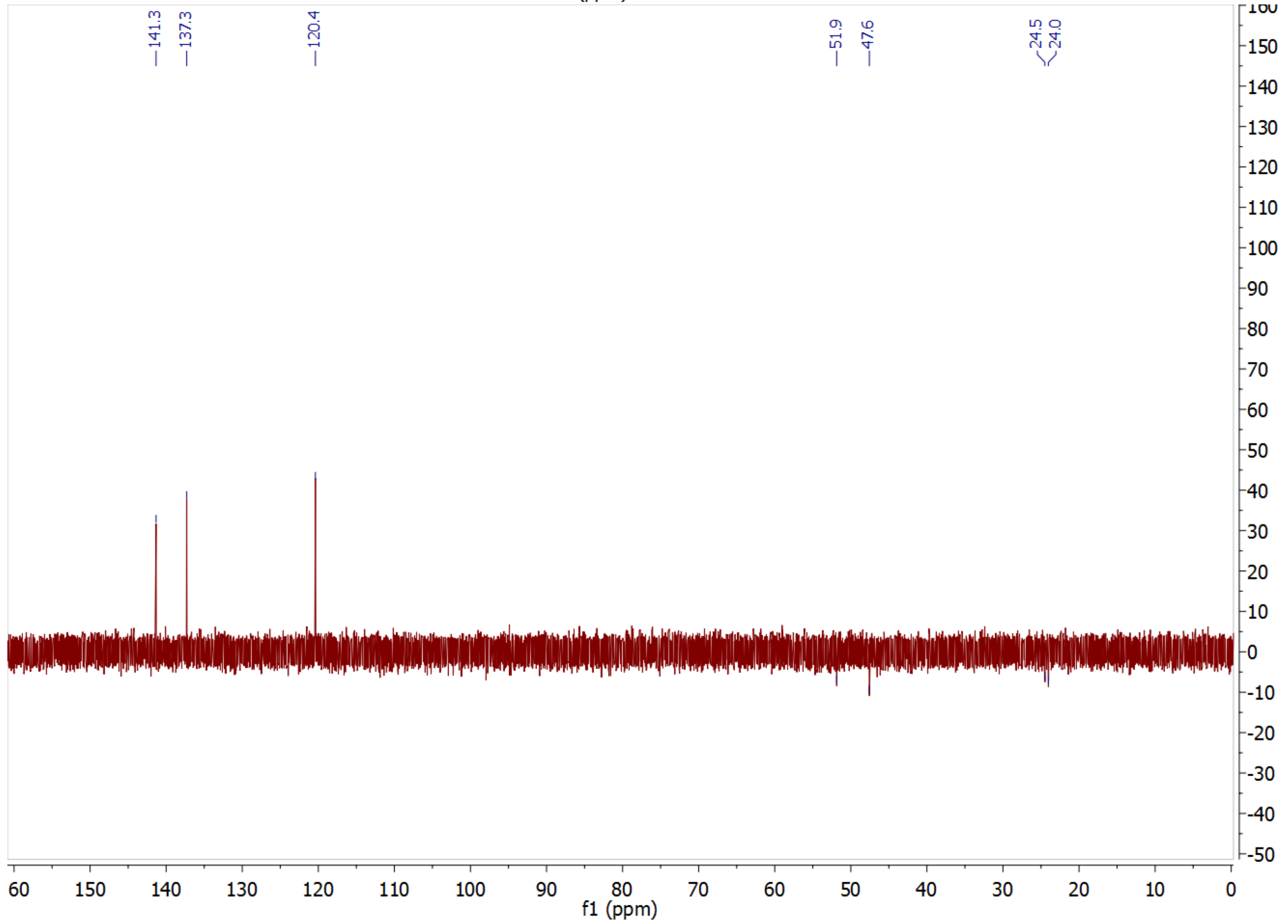
141.3
137.3
120.4
51.9
47.6
24.5
24.0
f1 (ppm)

Mass Spectrum SmartFormula Report

Analysis Info

Analysis Name	N:\new acq data\Ba149 chr 1_1 001.d	Acquisition Date	11.04.2016 17:30:19
Method	hn Direct_Infusion_pos mode_75-1700 mid 4eV.m	Operator	hn
Sample Name	Linda Bannwart	Instrument / Ser#	maXis 4G 21243
Comment	Ba149 chr 1#1, ca. 10 ug/ml MeCN		

Acquisition Parameter

Source Type	ESI	Ion Polarity	Positive	Set Nebulizer	0.4 Bar
Focus	Not active	Set Capillary	3600 V	Set Dry Heater	180 °C
Scan Begin	75 m/z	Set End Plate Offset	-500 V	Set Dry Gas	4.0 l/min
Scan End	1700 m/z	Set Collision Cell RF	350.0 Vpp	Set Ion Energy (MS only)	4.0 eV

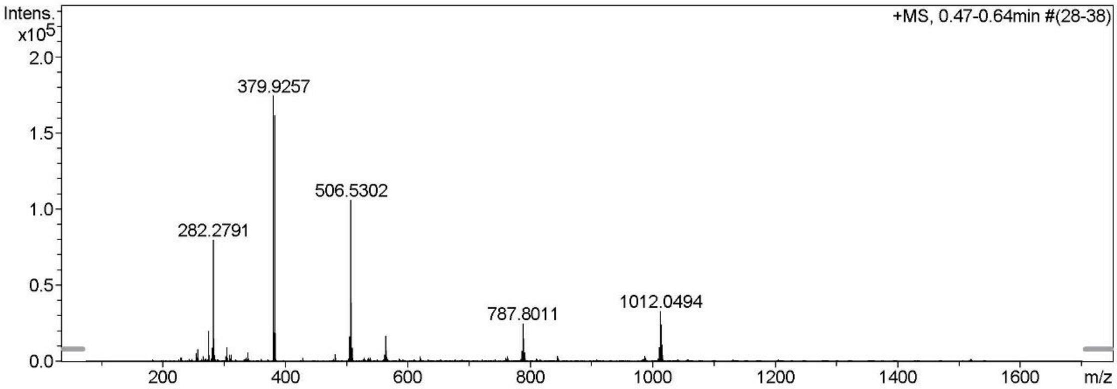

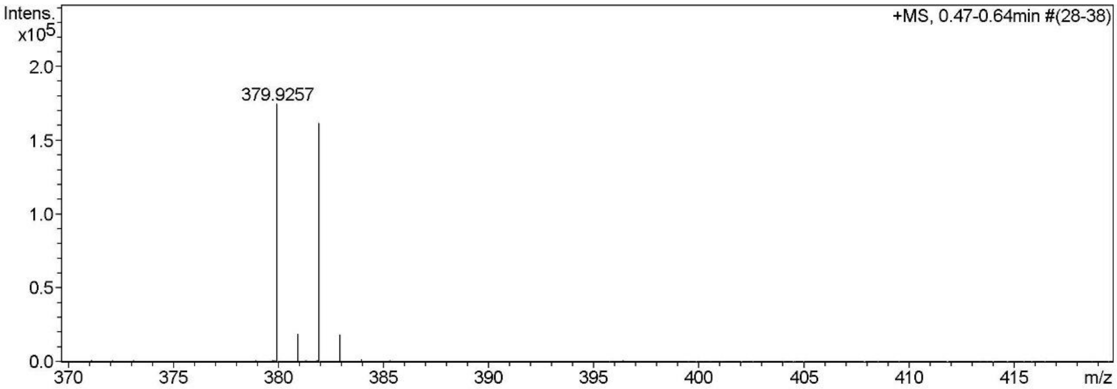

Meas. m/z	#	Formula	Score	m/z	err [mDa]	err [ppm]	mSigma	rdb	e⁻ Conf	N-Rule	z
379.9257	1	C 10 H 12 Br I N 3	100.00	379.9254	-0.4	-1.0	25.7	5.5	even	ok	1+

Mass Spectrum List Report

Analysis Info

Analysis Name	N:\new acq data\Ba149 chr 1_1 001.d	Acquisition Date	11.04.2016 17:30:19
Method	hn Direct_Infusion_pos mode_75-1700 mid 4eV.m	Operator	hn
Sample Name	Linda Bannwart	Instrument / Ser#	maXis 4G 21243
Comment	Ba149 chr 1#1, ca. 10 ug/ml MeCN		

Acquisition Parameter

Source Type	ESI	Ion Polarity	Positive	Set Nebulizer	0.4 Bar
Focus	Not active	Set Capillary	3600 V	Set Dry Heater	180 °C
Scan Begin	75 m/z	Set End Plate Offset	-500 V	Set Dry Gas	4.0 l/min
Scan End	1700 m/z	Set Collision Cell RF	350.0 Vpp	Set Ion Energy (MS only)	4.0 eV

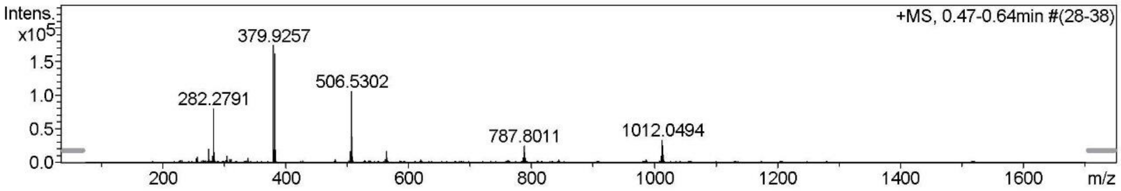

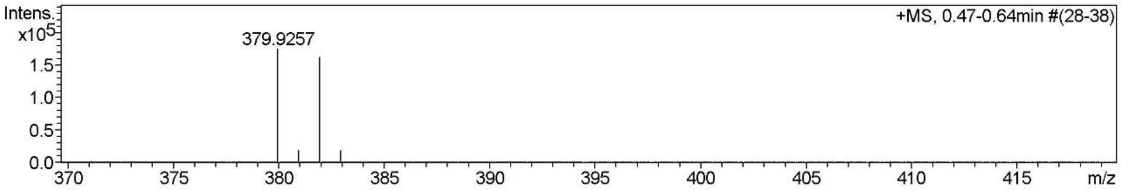

#	m/z	I %	I
1	228.2319	1.3	2284
2	230.2476	1.3	2282
3	242.2477	0.5	931
4	247.2418	0.6	996
5	254.0286	1.4	2399
6	254.2476	2.8	4917
7	255.2504	0.5	866
8	256.0264	1.3	2225
9	256.2632	4.2	7357
10	257.2666	0.8	1321
11	263.2366	0.7	1215
12	265.2524	1.6	2706
13	268.2629	0.7	1233
14	270.2783	0.8	1373
15	272.2582	0.5	789
16	274.2737	11.2	19494
17	275.2769	2.0	3453
18	278.2473	0.7	1269
19	280.2633	4.9	8597
20	281.2664	1.0	1697
21	282.2791	45.5	79128
22	283.2822	8.3	14471
23	284.2928	2.0	3460
24	288.2893	0.4	767
25	290.2684	0.4	767
26	302.2450	1.6	2765
27	304.2608	5.1	8952
28	305.2643	1.0	1807
29	308.8515	2.2	3868
30	310.3102	0.7	1255
31	310.8494	2.1	3739
32	318.3001	0.4	721
33	333.9368	0.7	1196
34	335.9758	1.1	1953
35	338.3416	3.1	5355
36	339.3447	0.8	1431
37	360.3237	0.5	946

Mass Spectrum List Report

#	m/z	I %	I
38	379.9257	100.0	174005
39	380.9284	10.5	18343
40	381.9238	92.5	160964
41	382.9263	10.3	17981
42	383.9290	0.5	918
43	427.9119	0.9	1599
44	478.4983	0.6	1069
45	480.5138	2.5	4335
46	481.5171	1.0	1665
47	502.4978	0.9	1552
48	504.5141	9.2	15964
49	505.5174	3.6	6231
50	506.5302	60.5	105320
51	507.5333	21.7	37758
52	508.5383	4.8	8324
53	509.5438	1.0	1654
54	528.5119	1.2	2148
55	529.5142	0.5	875
56	534.5608	0.7	1210
57	535.5195	1.1	1927
58	536.5230	0.4	748
59	537.5349	1.1	1966
60	538.5389	0.4	733
61	559.5195	0.4	709
62	561.5356	2.3	3925
63	562.5385	0.9	1573
64	563.5513	9.3	16226
65	564.5544	3.8	6576
66	565.5619	1.3	2307
67	566.5648	0.6	1019
68	585.5327	0.7	1287
69	591.5821	0.5	812
70	610.1846	0.5	823
71	619.6137	1.6	2864
72	620.6161	0.8	1359
73	688.5994	0.4	728
74	759.7700	1.1	1835
75	760.7732	0.6	996
76	761.7850	1.6	2751
77	762.7880	0.8	1452
78	783.7692	0.6	1123
79	785.7852	3.6	6222
80	786.7889	2.0	3502
81	787.8011	14.0	24307
82	788.8042	8.3	14403
83	789.8094	3.1	5347
84	790.8156	0.9	1631
85	809.7830	0.7	1266
86	815.8320	0.5	860
87	843.8631	1.7	3015
88	844.8667	1.0	1777
89	984.0180	0.6	963
90	985.0225	0.5	789
91	986.0337	1.8	3116
92	987.0364	1.3	2192
93	988.0420	0.5	902
94	1008.0182	0.9	1521
95	1009.0215	0.6	1082
96	1010.0338	5.1	8948
97	1011.0373	3.8	6680
98	1012.0494	18.7	32555
99	1013.0530	13.7	23858
100	1014.0572	6.2	10704
101	1015.0626	2.0	3487
102	1016.0666	0.6	962
103	1040.0807	0.5	818
104	1517.5640	0.6	1032
105	1518.5669	0.6	964

5.3 Compound **7**

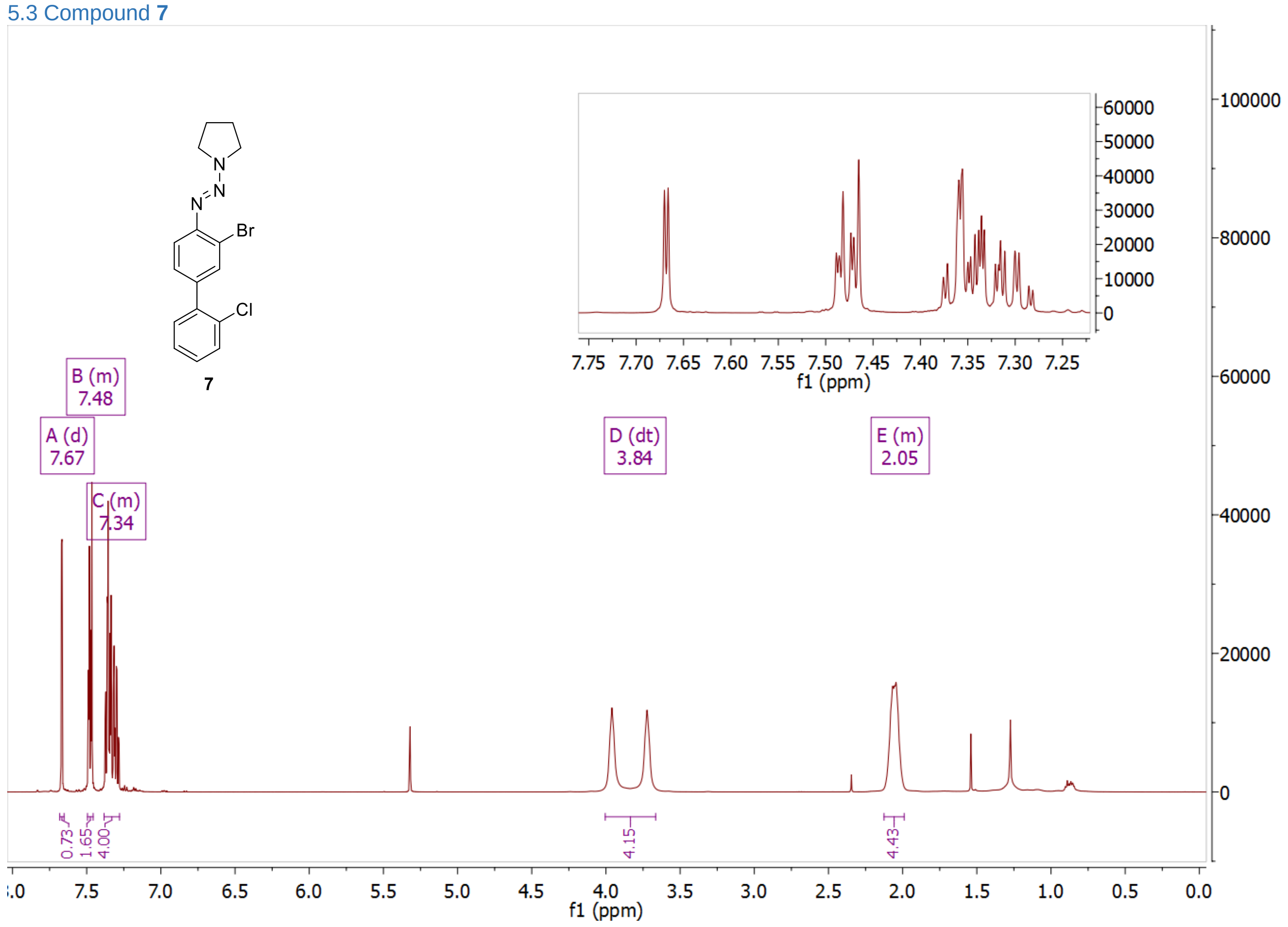

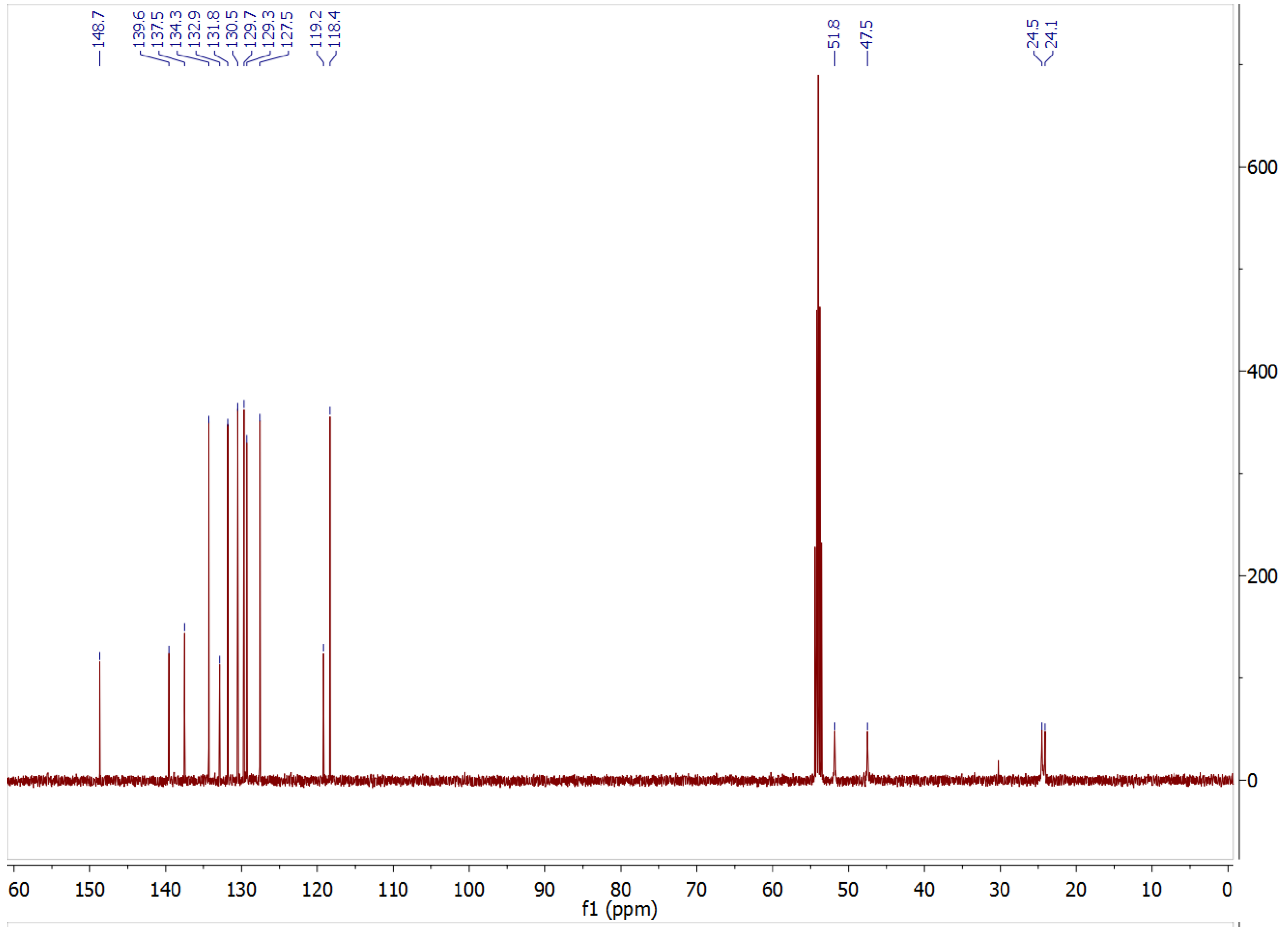

148.7
139.6
137.5
134.3
132.9
131.8
130.5
129.7
129.3
127.5
119.2
118.4
51.8
47.5
24.5
24.1
f1 (ppm)

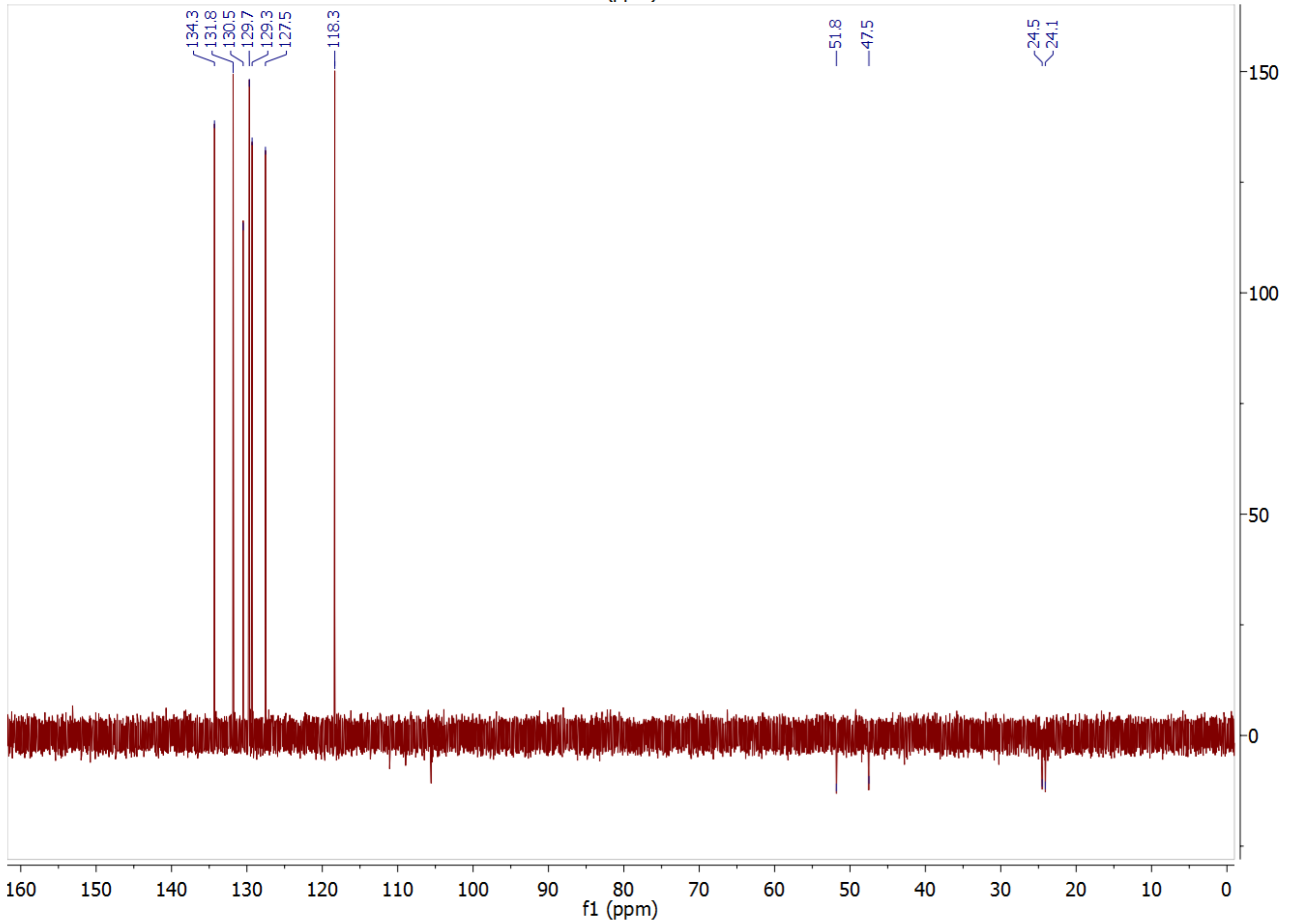

134.3
131.8
130.5
129.7
129.3
127.5
118.3
51.8
47.5
24.5
24.1
f1 (ppm)

Mass Spectrum SmartFormula Report

Analysis Info

Analysis Name	N:\new acq data\Ba185 chr 1_3 001.d	Acquisition Date	11.04.2016 17:03:17
Method	hn Direct_Infusion_pos mode_75-1700 mid 4eV.m	Operator	hn
Sample Name	Linda Bannwart	Instrument / Ser#	maXis 4G 21243
Comment	Ba185 chr 1#3, ca. 10 ug/ml MeCN		

Acquisition Parameter

Source Type	ESI	Ion Polarity	Positive	Set Nebulizer	0.4 Bar
Focus	Not active	Set Capillary	3600 V	Set Dry Heater	180 °C
Scan Begin	75 m/z	Set End Plate Offset	-500 V	Set Dry Gas	4.0 l/min
Scan End	1700 m/z	Set Collision Cell RF	350.0 Vpp	Set Ion Energy (MS only)	4.0 eV

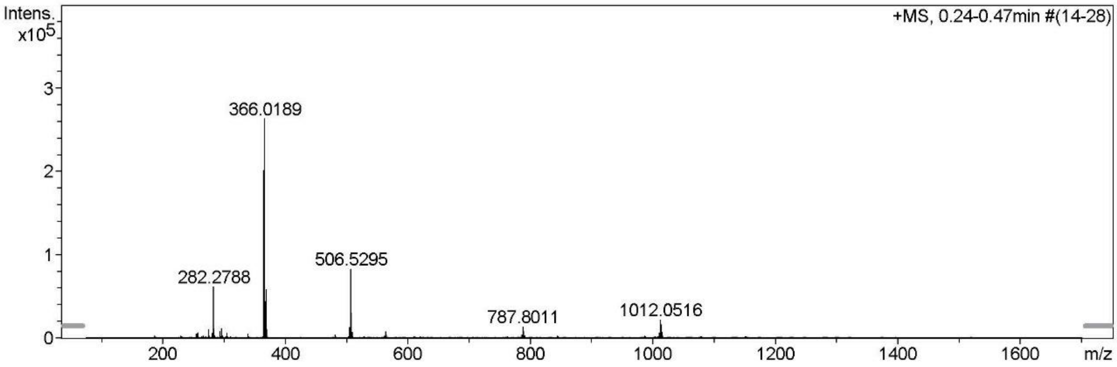

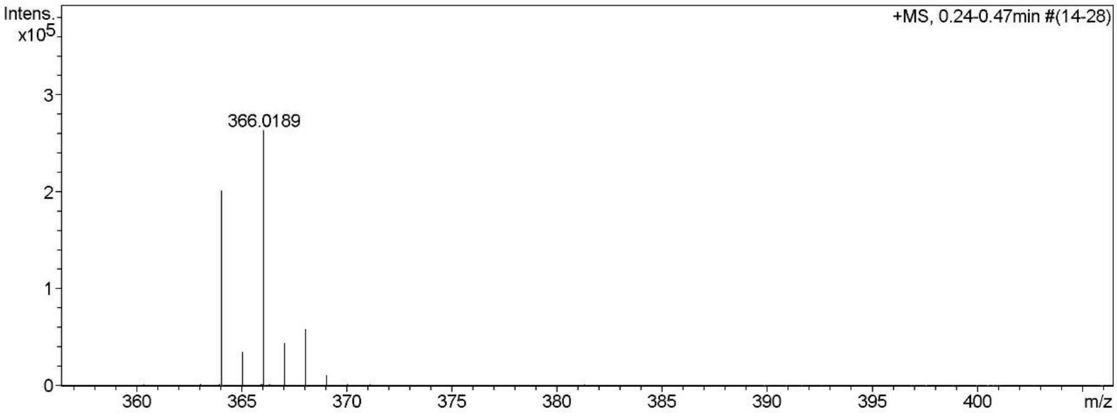

Meas. m/z	#	Formula	Score	m/z	err [mDa]	err [ppm]	mSigma	rdb	e⁻ Conf	N-Rule	z
364.0211	1	C 16 H 16 Br Cl N 3	100.00	364.0211	-0.0	-0.1	15.4	9.5	even	ok	1+

Mass Spectrum List Report

Analysis Info

Analysis Name	N:\new acq data\Ba185 chr 1_3 001.d	Acquisition Date	11.04.2016 17:03:17
Method	hn Direct_Infusion_pos mode_75-1700 mid 4eV.m	Operator	hn
Sample Name	Linda Bannwart	Instrument / Ser#	maXis 4G 21243
Comment	Ba185 chr 1#3, ca. 10 ug/ml MeCN		

Acquisition Parameter

Source Type	ESI	Ion Polarity	Positive	Set Nebulizer	0.4 Bar
Focus	Not active	Set Capillary	3600 V	Set Dry Heater	180 °C
Scan Begin	75 m/z	Set End Plate Offset	-500 V	Set Dry Gas	4.0 l/min
Scan End	1700 m/z	Set Collision Cell RF	350.0 Vpp	Set Ion Energy (MS only)	4.0 eV

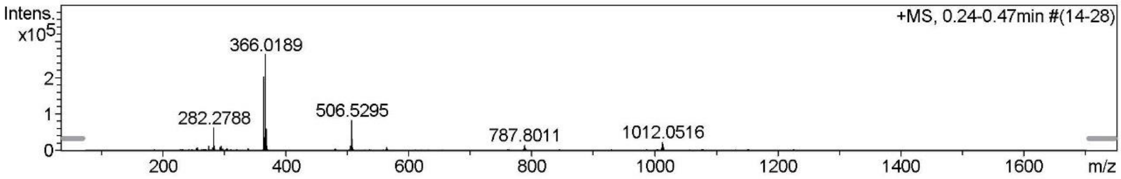

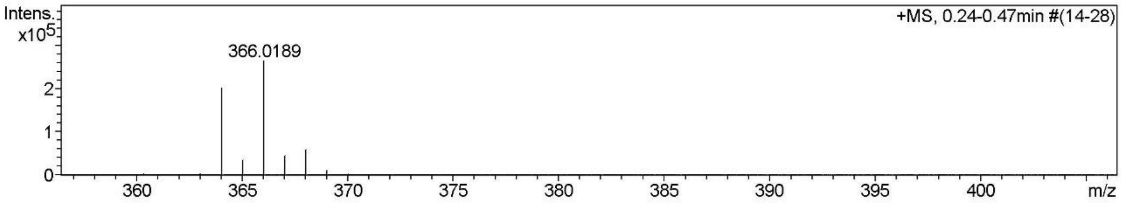

#	m/z	I %	I
1	186.0226	0.7	1800
2	228.2319	0.7	1837
3	230.2475	0.6	1475
4	231.9699	0.3	765
5	247.2415	0.3	815
6	254.0283	1.7	4520
7	254.2475	1.4	3776
8	256.0263	1.7	4497
9	256.2631	2.2	5756
10	257.2664	0.4	1086
11	263.2365	0.4	1047
12	265.2521	0.8	2193
13	266.9386	0.3	790
14	268.2627	0.3	903
15	270.2783	0.4	1081
16	274.2735	3.8	9941
17	275.2768	0.7	1784
18	278.2470	0.3	775
19	280.2629	2.1	5420
20	280.9596	0.3	879
21	281.2663	0.4	1077
22	282.2788	23.2	61021
23	282.9570	0.4	1104
24	283.2820	4.3	11283
25	284.2926	1.0	2722
26	292.9472	3.0	7941
27	293.9501	0.4	1081
28	294.9449	4.1	10783
29	295.9480	0.6	1522
30	296.9421	1.0	2662
31	302.2448	0.5	1351
32	304.2606	1.9	4920
33	305.2639	0.4	1062
34	310.3100	0.4	1091
35	320.2345	0.3	784
36	338.3409	1.8	4714
37	339.3445	0.5	1196

Mass Spectrum List Report

#	m/z	I %	I
38	363.0129	0.5	1309
39	364.0211	76.3	200685
40	365.0236	12.8	33651
41	366.0189	100.0	263024
42	367.0216	16.5	43475
43	368.0161	21.9	57585
44	369.0189	3.8	9958
45	370.0221	0.4	933
46	478.4974	0.3	840
47	480.5131	1.3	3496
48	481.5167	0.5	1235
49	502.4977	0.5	1325
50	504.5136	4.7	12420
51	505.5169	1.8	4863
52	506.5295	31.2	82084
53	507.5327	11.3	29774
54	508.5378	2.5	6580
55	509.5433	0.5	1250
56	528.5109	0.6	1525
57	534.5603	0.3	853
58	535.5191	0.3	858
59	537.5344	0.4	965
60	557.0943	0.3	801
61	561.5351	0.7	1757
62	562.5378	0.3	808
63	563.5506	2.8	7297
64	564.5539	1.2	3096
65	565.5611	0.4	924
66	610.1838	0.4	1010
67	619.6133	0.6	1450
68	653.4037	0.3	763
69	759.7693	0.4	949
70	761.7858	0.6	1485
71	762.7894	0.3	838
72	785.7853	1.2	3194
73	786.7891	0.7	1788
74	787.8011	4.9	12815
75	788.8044	2.9	7584
76	789.8105	1.1	2797
77	790.8159	0.3	849
78	843.8634	0.7	1710
79	844.8672	0.4	1128
80	929.1749	0.3	840
81	986.0354	0.8	2048
82	987.0390	0.6	1492
83	1001.2082	0.3	787
84	1003.1994	0.4	1113
85	1004.1970	0.3	835
86	1005.1921	0.3	776
87	1008.0201	0.4	991
88	1010.0359	2.3	5945
89	1011.0395	1.7	4512
90	1012.0516	8.2	21654
91	1013.0551	6.0	15759
92	1014.0598	2.6	6712
93	1015.0656	0.9	2280
94	1056.2925	0.3	802
95	1075.2272	0.3	911
96	1076.2277	0.4	932
97	1077.2223	0.5	1221
98	1078.2201	0.4	973
99	1079.2120	0.3	820
100	1130.3133	0.3	786
101	1150.2456	0.4	979
102	1151.2411	0.5	1185
103	1152.2387	0.3	846
104	1224.2653	0.3	813
105	1225.2632	0.4	924

5.4 Compound **8**

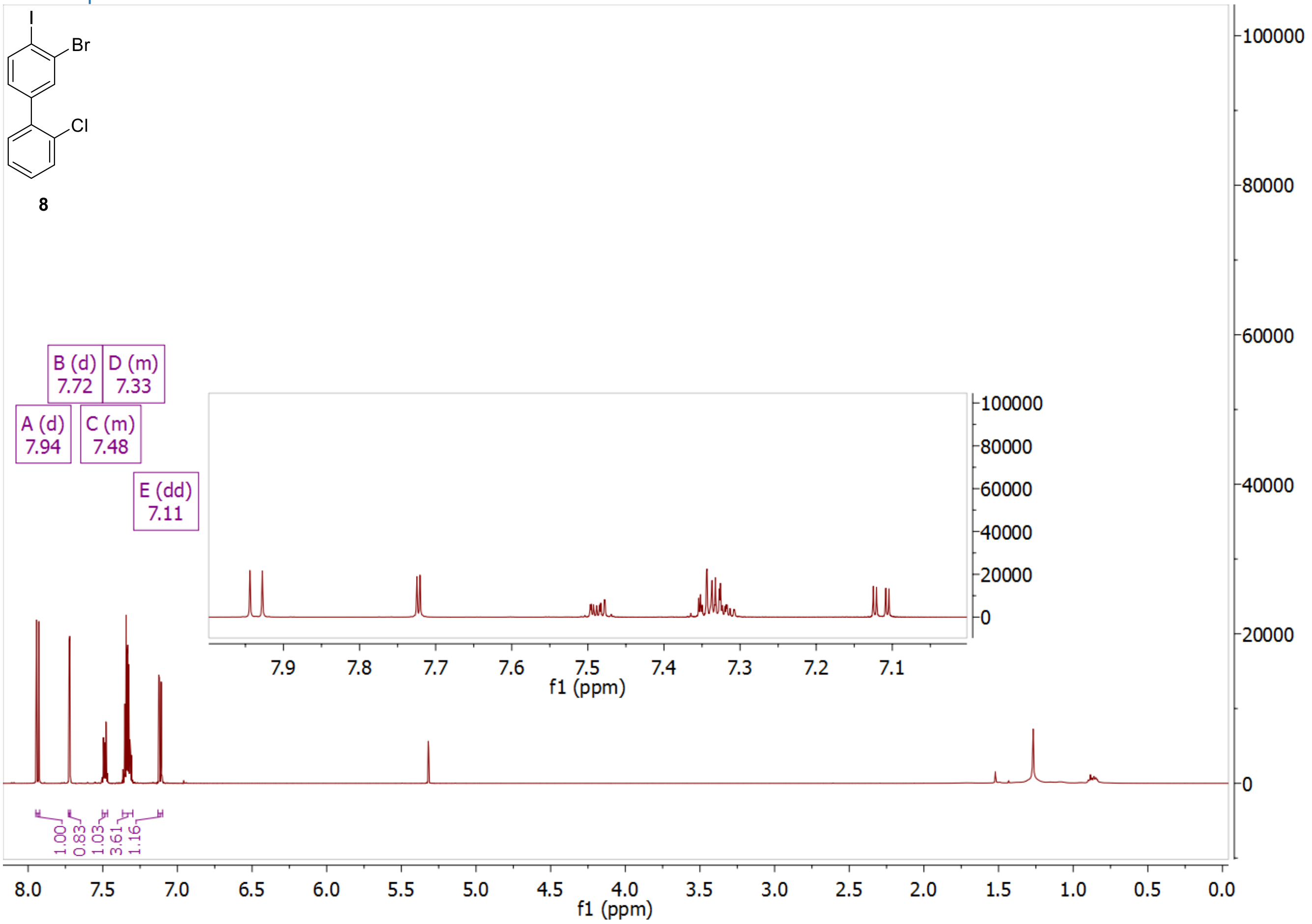

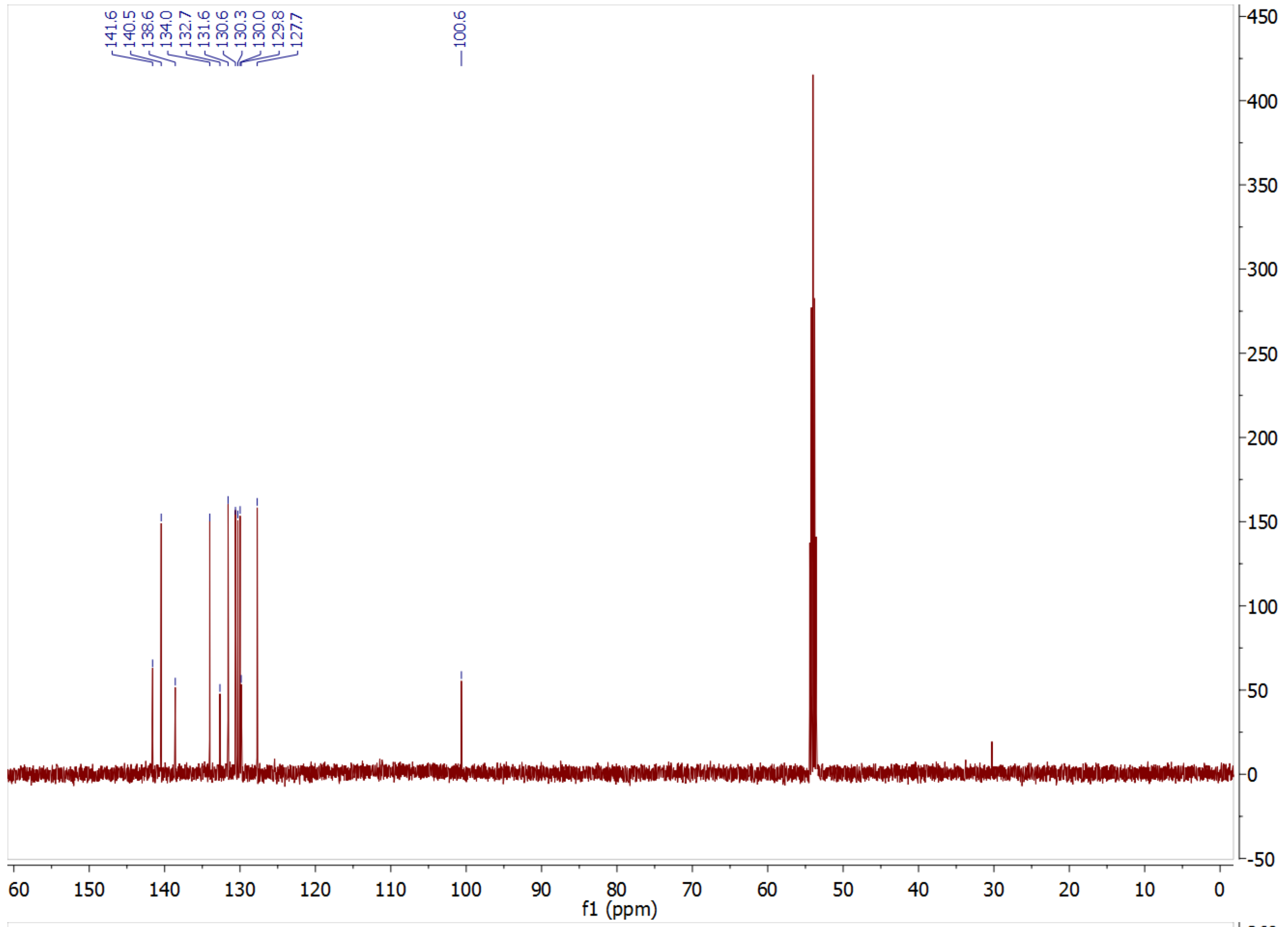
141.6
140.5
138.6
134.0
132.7
131.6
130.6
130.3
130.0
129.8
127.7
100.6
f1 (ppm)

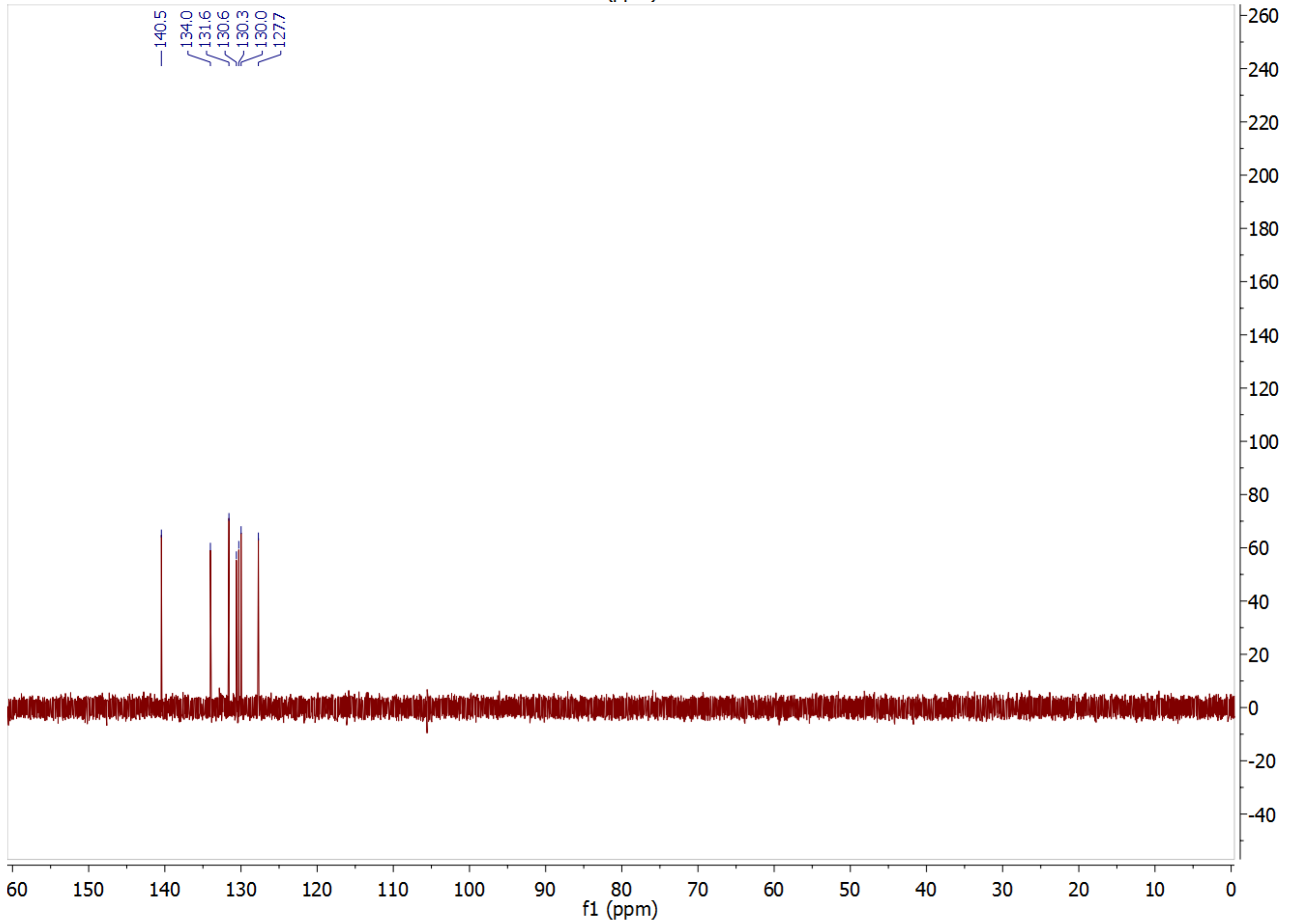
140.5
134.0
131.6
130.6
130.3
130.0
127.7
f1 (ppm)

D:\Data\TGC0003xx\TGCO000307_GC 09/30/19 12:08:09
TGCO000307;TGCO000307 Bannwart Linda/ - Ba187_chr1_1 - DCM
RT: 0.00 - 22.00

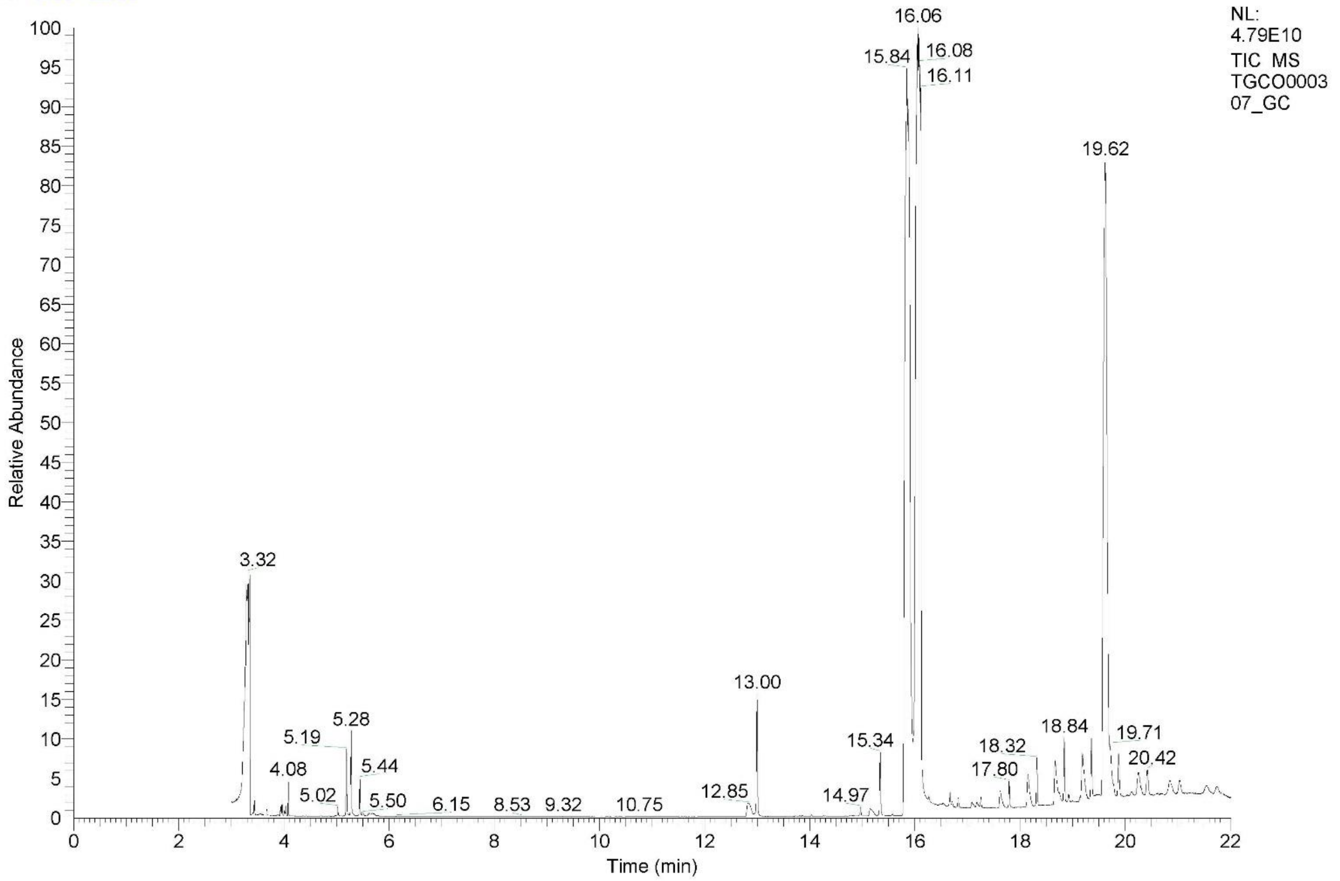

D:\Data\TGC0003xx\TGCO000307_GC 09/30/19 12:08:09
TGCO000307;TGCO000307 Bannwart Linda/ - Ba187_chr1_1 - DCM
TGCO000307_GC #2766-3681 RT: 15.67-19.80 AV: 916 NL: 4.44E8
T: FTMS + p EI Full ms [30.0000-600.0000]

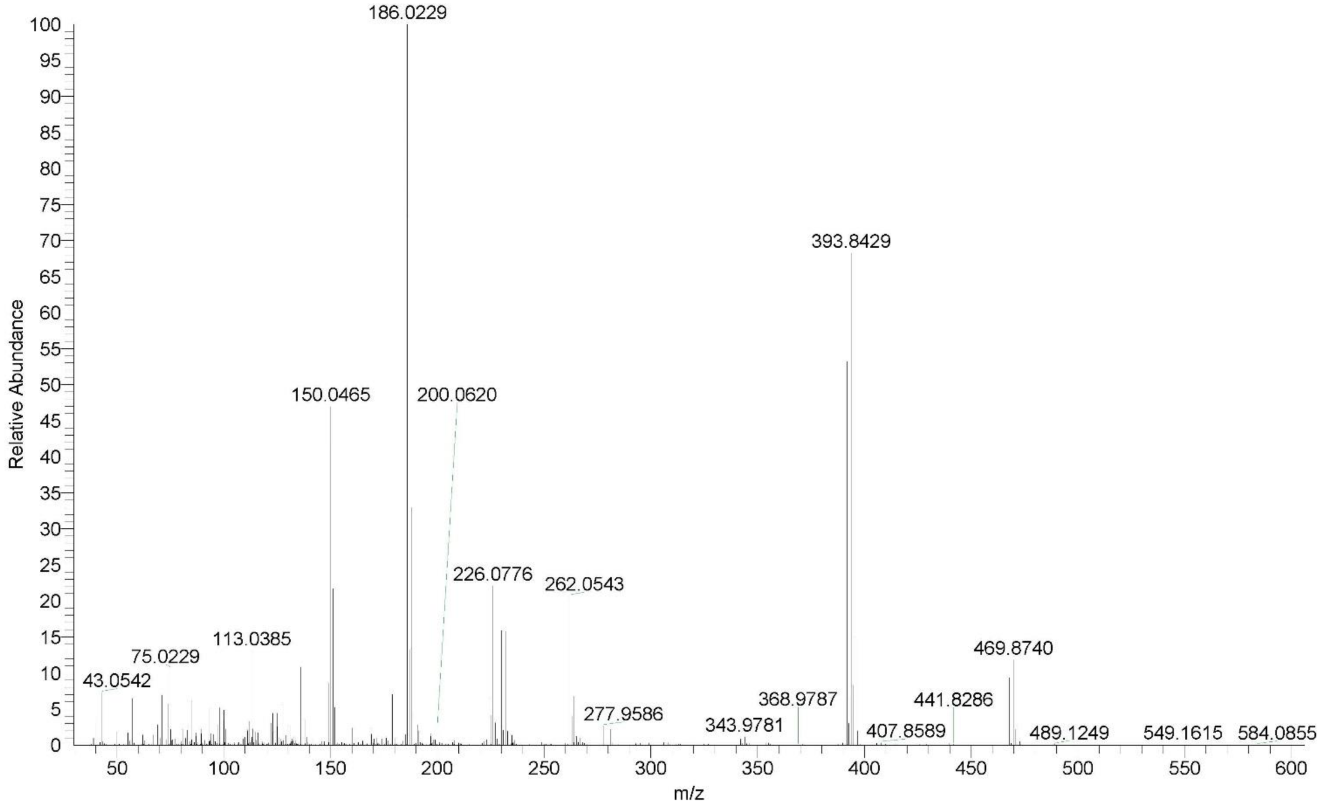

D:\Data\TGC0003xx\TGCO000307_GC 09/30/19 12:08:09
TGCO000307;TGCO000307 Bannwart Linda/ - Ba187_chr1_1 - DCM

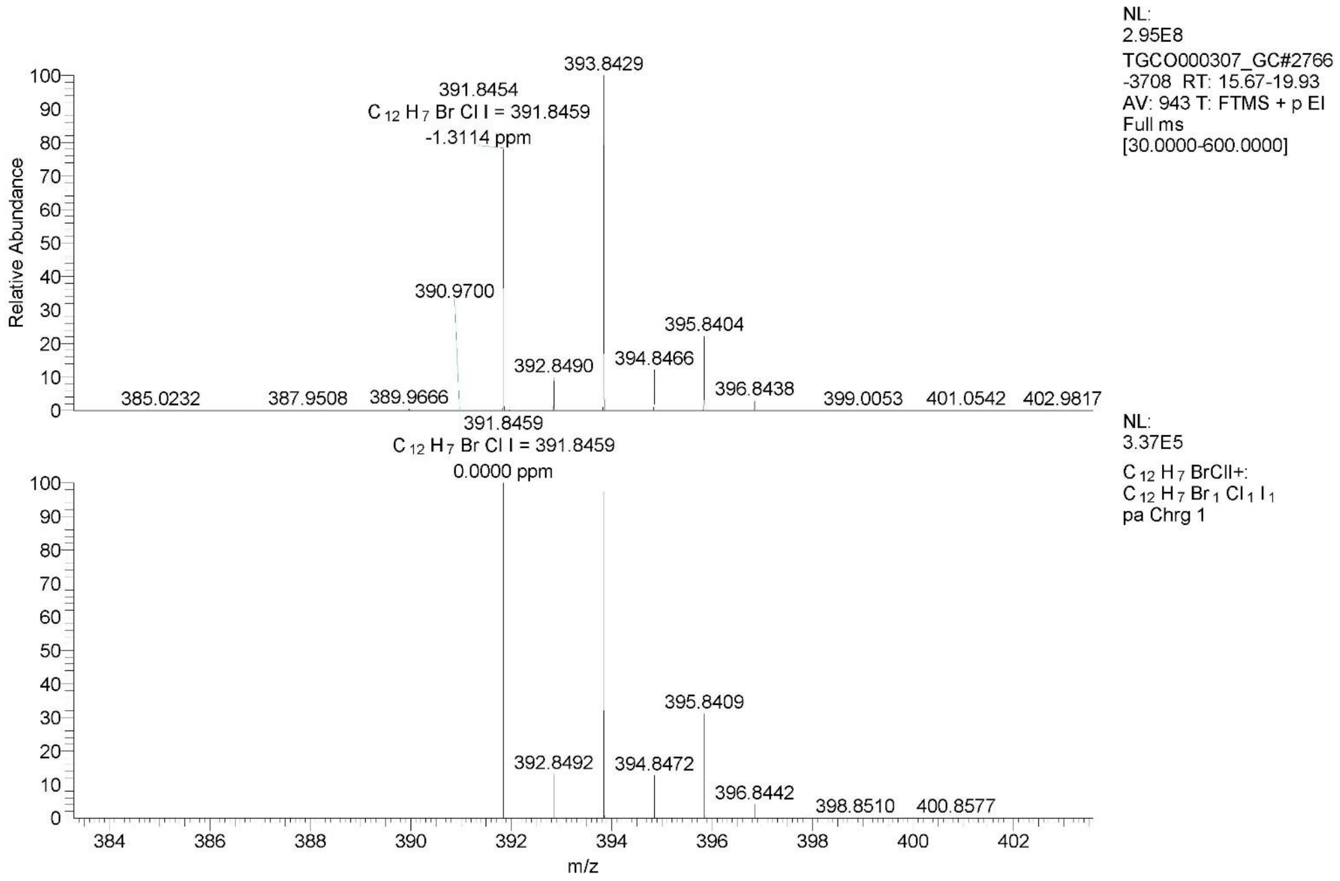

5.5 Compound **9**

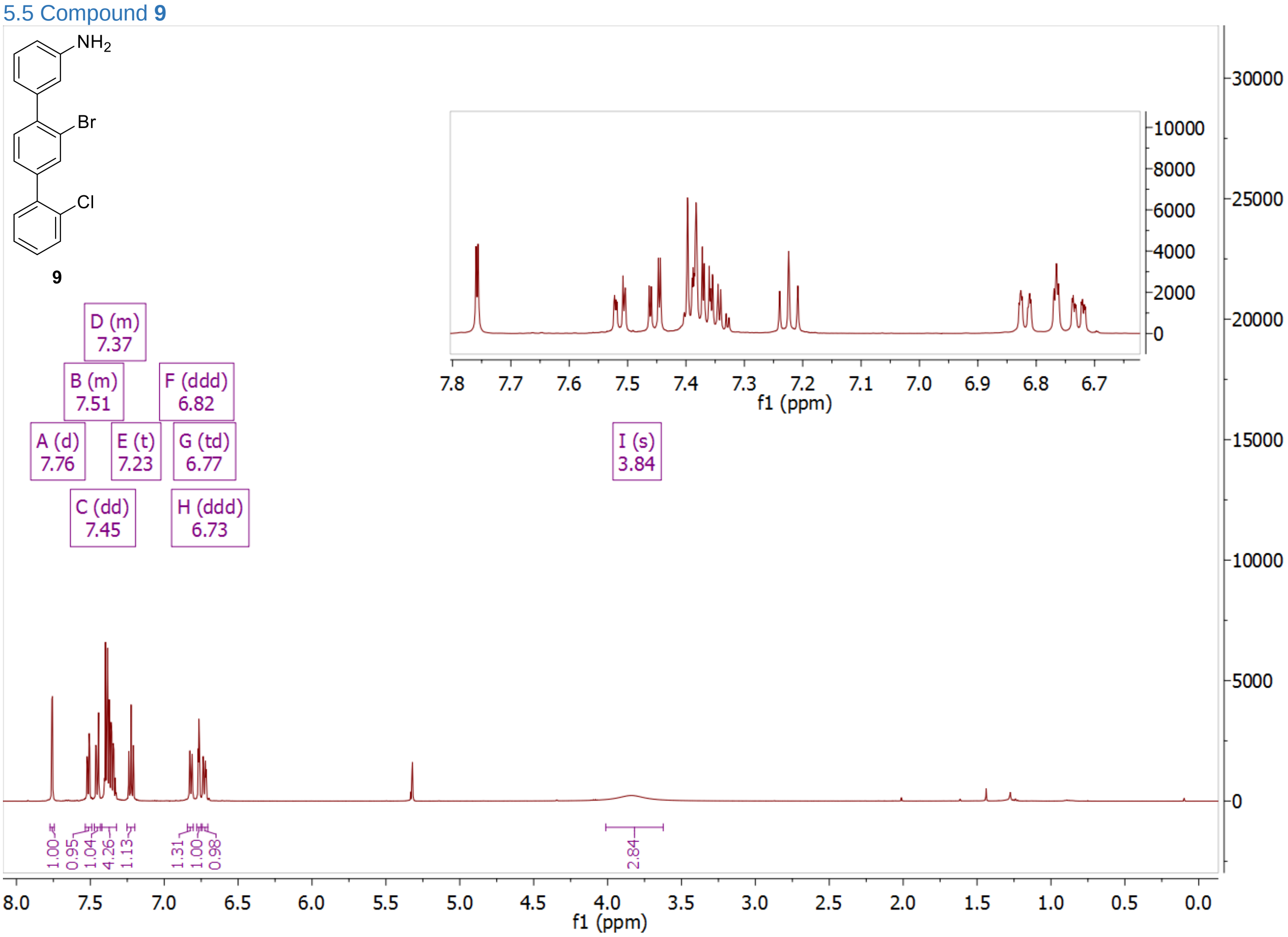

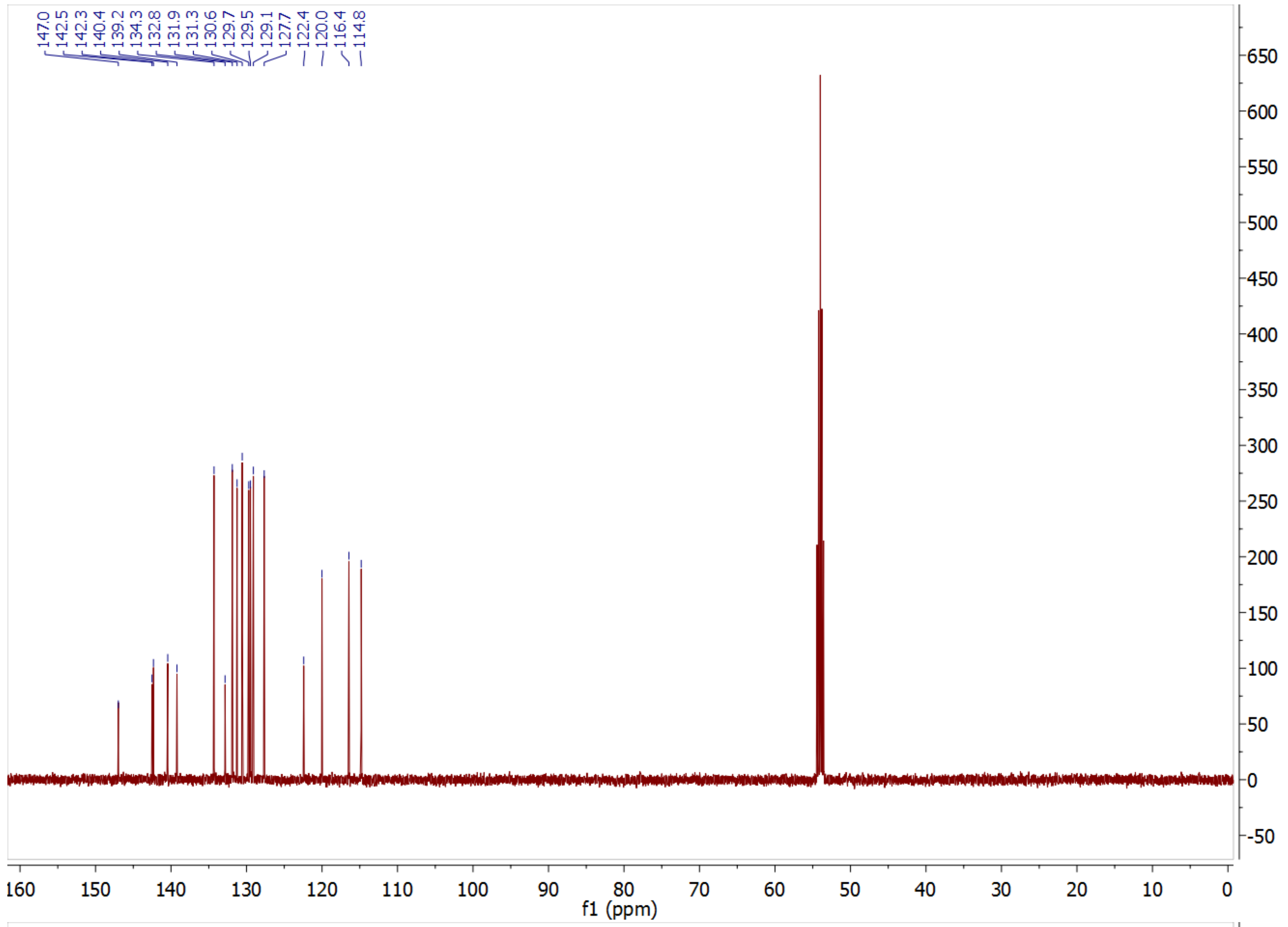
147.0
142.5
142.3
140.4
139.2
134.3
132.8
131.9
131.3
130.6
129.7
129.5
129.1
127.7
122.4
120.0
116.4
114.8
f1 (ppm)

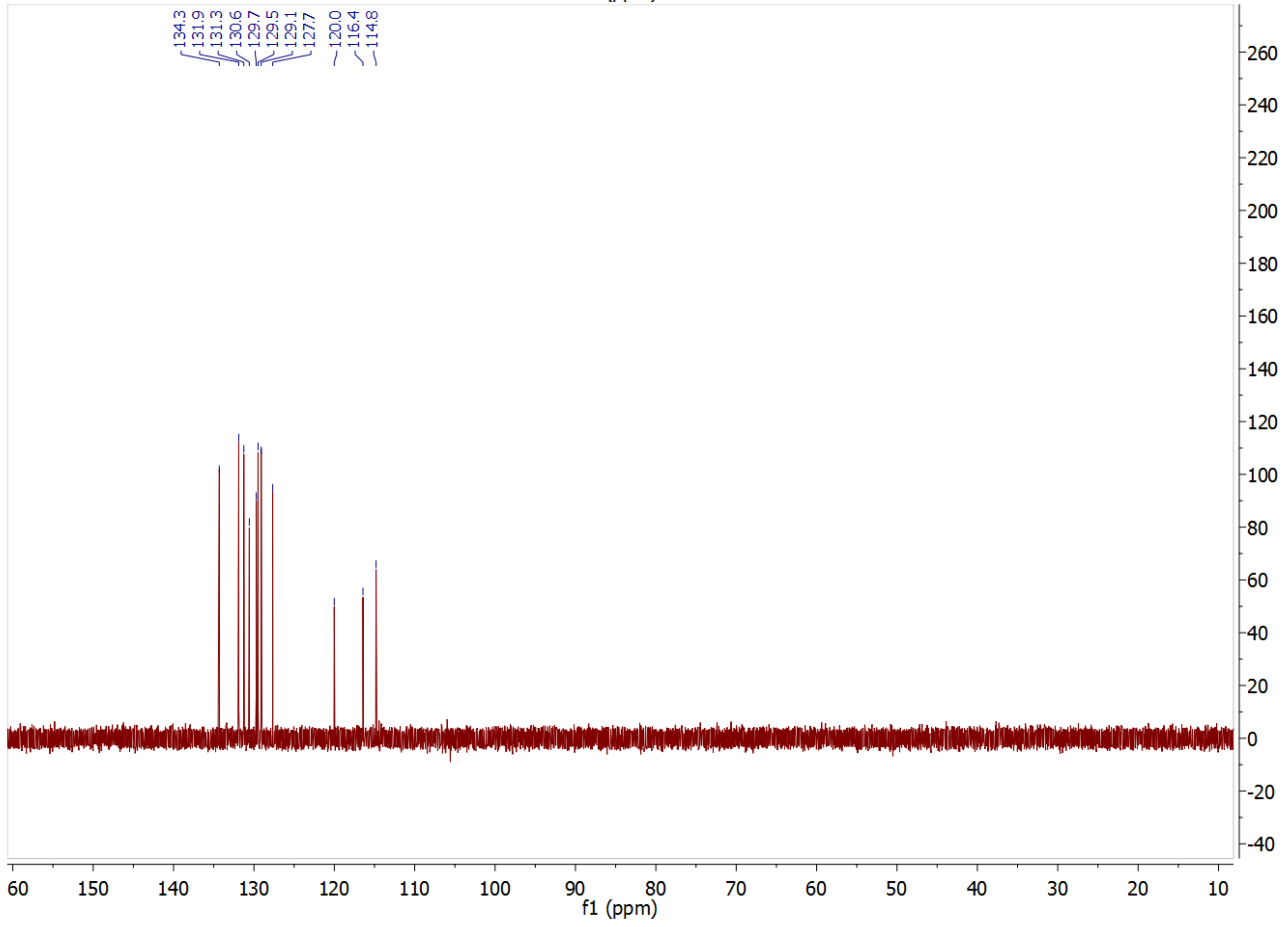
134.3
131.9
131.3
130.6
129.7
129.5
129.1
127.7
120.0
116.4
114.8
f1 (ppm)

Mass Spectrum SmartFormula Report

Analysis Info

Analysis Name	N:\new acq data\Ba205 chr 1_1 001.d	Acquisition Date	11.04.2016 14:21:12
Method	hn Direct_Infusion_pos mode_75-1700 mid 4eV.m	Operator	hn
Sample Name	Linda Bannwart	Instrument / Ser#	maXis 4G 21243
Comment	Ba205 chr 1#1, ca. 12 ug/ml MeCN		

Acquisition Parameter

Source Type	ESI	Ion Polarity	Positive	Set Nebulizer	0.4 Bar
Focus	Not active	Set Capillary	3600 V	Set Dry Heater	180 °C
Scan Begin	75 m/z	Set End Plate Offset	-500 V	Set Dry Gas	4.0 l/min
Scan End	1700 m/z	Set Collision Cell RF	350.0 Vpp	Set Ion Energy (MS only)	4.0 eV

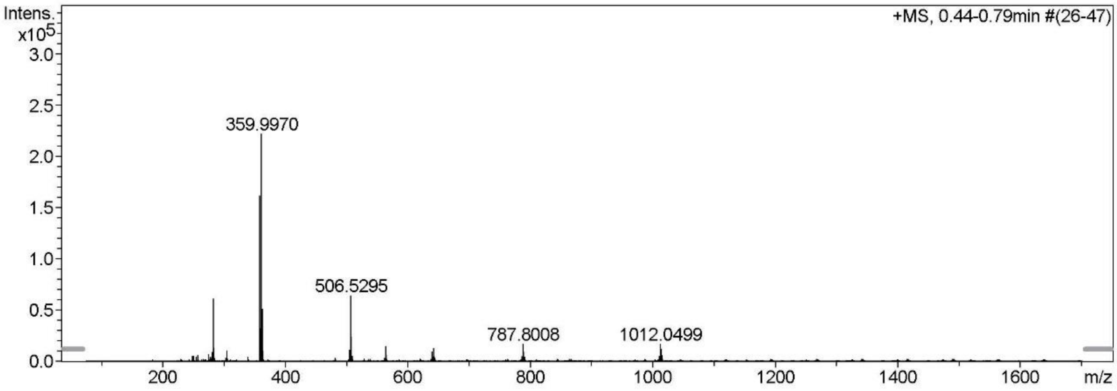

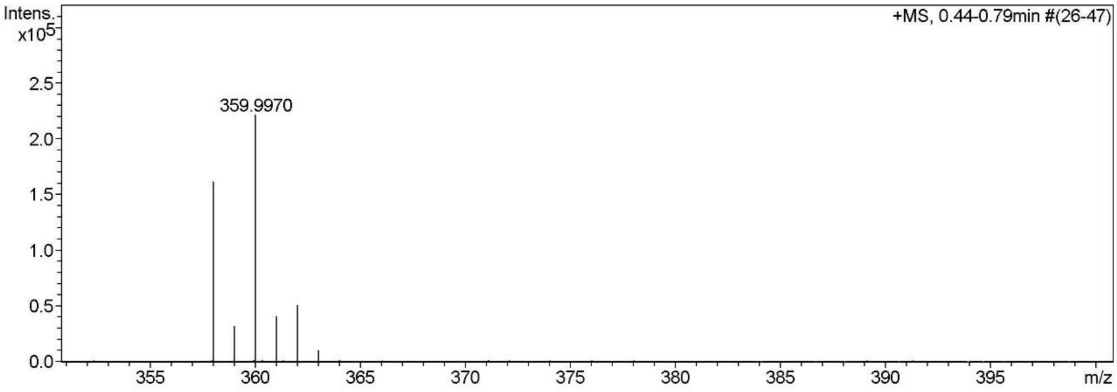

Meas. m/z	#	Formula	Score	m/z	err [mDa]	err [ppm]	mSigma	rdb	e⁻ Conf	N-Rule	z
357.9992	1	C 18 H 14 Br Cl N	100.00	357.9993	0.1	0.3	18.0	11.5	even	ok	1+

Mass Spectrum List Report

Analysis Info

Analysis Name	N:\new acq data\Ba205 chr 1_1 001.d	Acquisition Date	11.04.2016 14:21:12
Method	hn Direct_Infusion_pos mode_75-1700 mid 4eV.m	Operator	hn
Sample Name	Linda Bannwart	Instrument / Ser#	maXis 4G 21243
Comment	Ba205 chr 1#1, ca. 12 ug/ml MeCN		

Acquisition Parameter

Source Type	ESI	Ion Polarity	Positive	Set Nebulizer	0.4 Bar
Focus	Not active	Set Capillary	3600 V	Set Dry Heater	180 °C
Scan Begin	75 m/z	Set End Plate Offset	-500 V	Set Dry Gas	4.0 l/min
Scan End	1700 m/z	Set Collision Cell RF	350.0 Vpp	Set Ion Energy (MS only)	4.0 eV

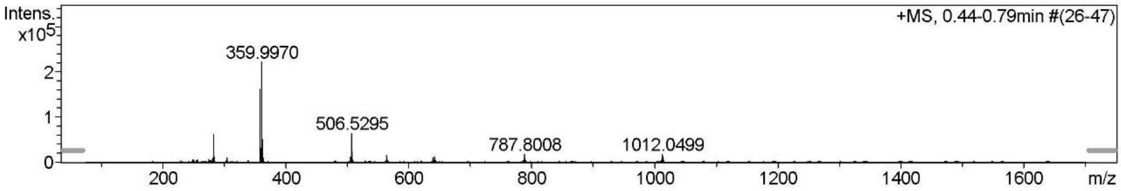

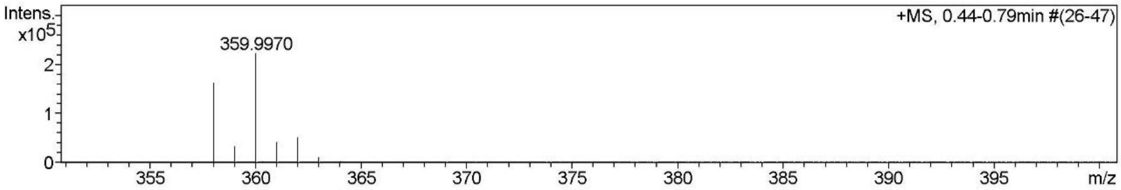

#	m/z	I %	I
1	183.0778	0.5	1090
2	228.2316	0.8	1817
3	230.2472	0.6	1257
4	242.2473	0.5	1075
5	248.0064	2.0	4528
6	250.0044	2.1	4655
7	254.2473	1.8	3905
8	256.2628	2.3	5192
9	265.2520	0.8	1703
10	270.2785	0.5	1019
11	274.2734	2.9	6305
12	275.2767	0.5	1212
13	277.0646	1.4	3177
14	278.0719	1.7	3735
15	278.2467	0.7	1465
16	279.0632	0.5	1158
17	279.0923	0.9	1967
18	280.0691	0.6	1270
19	280.2629	3.9	8640
20	281.2662	0.8	1767
21	282.2787	27.3	60419
22	283.2819	5.6	12400
23	284.2922	1.1	2383
24	302.2446	1.3	2780
25	304.2605	4.4	9773
26	305.2638	1.0	2154
27	320.2344	0.5	1069
28	338.3411	1.7	3676
29	357.9992	72.8	161042
30	359.0021	14.2	31306
31	359.9970	100.0	221079
32	361.0000	18.2	40233
33	361.9942	22.7	50206
34	362.9971	4.4	9628
35	480.5137	1.2	2749
36	502.4976	0.5	1130
37	504.5136	4.8	10529

Mass Spectrum List Report

#	m/z	I %	I
38	505.5168	1.8	3965
39	506.5295	28.7	63493
40	507.5327	10.4	23100
41	508.5377	2.2	4955
42	528.5109	0.9	1956
43	535.5195	0.7	1630
44	537.5348	0.8	1715
45	561.5350	1.3	2826
46	562.5385	0.5	1094
47	563.5507	6.4	14167
48	564.5539	2.6	5749
49	565.5610	0.8	1835
50	585.5328	0.5	1138
51	619.6132	1.0	2161
52	637.2546	0.8	1719
53	639.2698	4.1	8967
54	640.2731	1.6	3622
55	641.2688	5.6	12304
56	642.2718	2.1	4635
57	643.2683	1.5	3305
58	644.2703	0.5	1175
59	697.3309	0.5	1096
60	759.7696	0.5	1215
61	761.7847	0.8	1765
62	762.7875	0.5	1070
63	785.7848	1.9	4097
64	786.7885	1.1	2344
65	787.8008	7.3	16200
66	788.8042	4.1	9149
67	789.8100	1.6	3433
68	790.8157	0.5	1112
69	843.8632	0.7	1627
70	844.8665	0.5	1100
71	863.5193	0.6	1387
72	865.5186	0.8	1781
73	970.1582	0.5	1029
74	986.0344	0.7	1478
75	987.0386	0.5	1016
76	1010.0345	2.1	4666
77	1011.0383	1.6	3541
78	1012.0499	7.3	16108
79	1013.0536	5.3	11697
80	1014.0583	2.3	5027
81	1015.0643	0.8	1720
82	1044.1769	0.5	1050
83	1118.1941	0.6	1280
84	1119.1956	0.5	1030
85	1192.2135	0.7	1445
86	1193.2133	0.6	1243
87	1194.2123	0.5	1080
88	1266.2322	0.7	1489
89	1267.2330	0.6	1332
90	1268.2305	0.6	1216
91	1340.2509	0.7	1537
92	1341.2503	0.6	1350
93	1342.2490	0.6	1347
94	1414.2674	0.6	1430
95	1415.2695	0.6	1367
96	1416.2668	0.6	1375
97	1417.2679	0.5	1012
98	1488.2875	0.6	1359
99	1489.2870	0.6	1345
100	1490.2852	0.6	1317
101	1562.3048	0.5	1160
102	1563.3059	0.5	1192
103	1564.3036	0.6	1270
104	1637.3241	0.5	1051
105	1638.3226	0.5	1087

5.6 Compound **10**

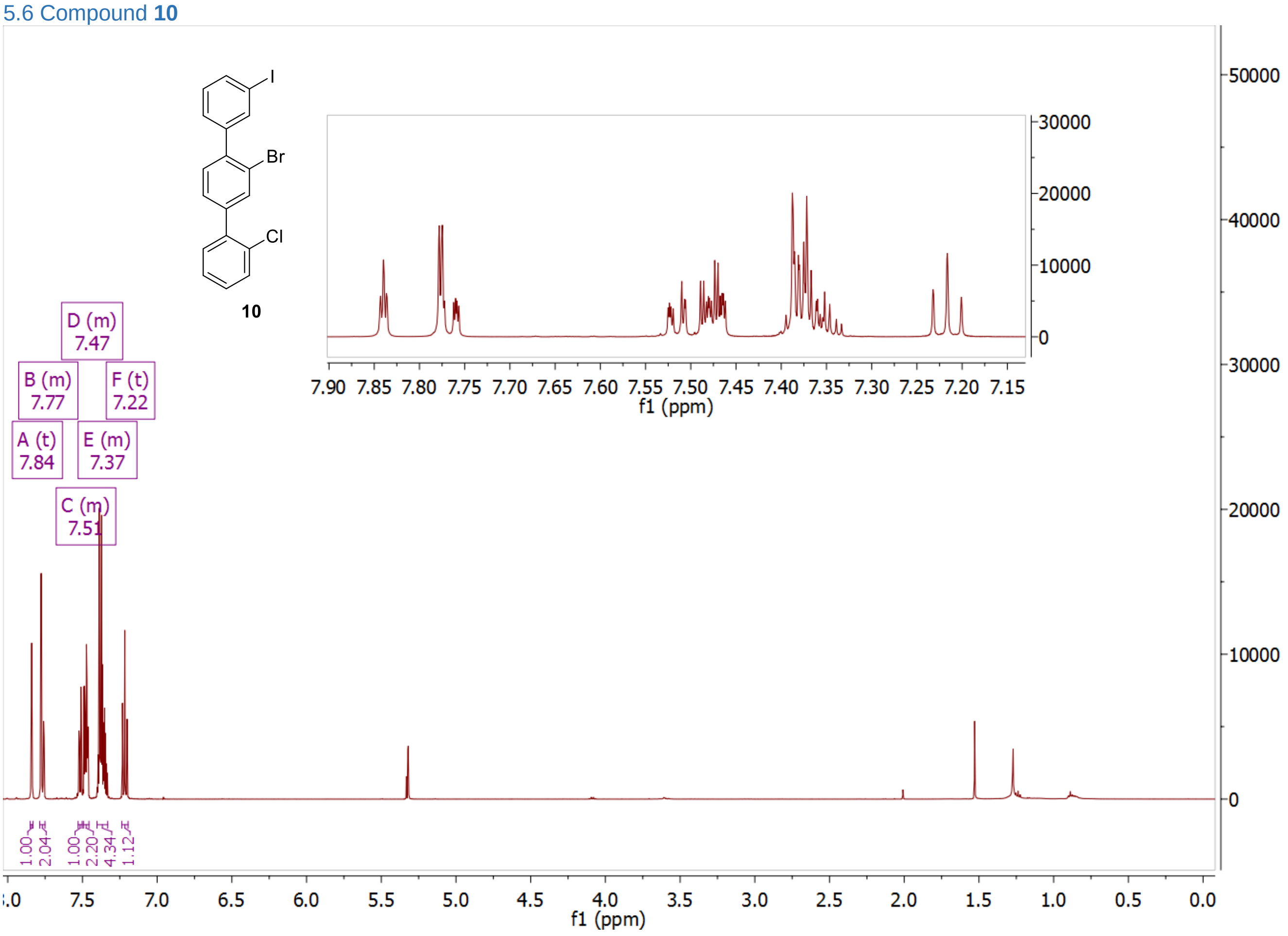

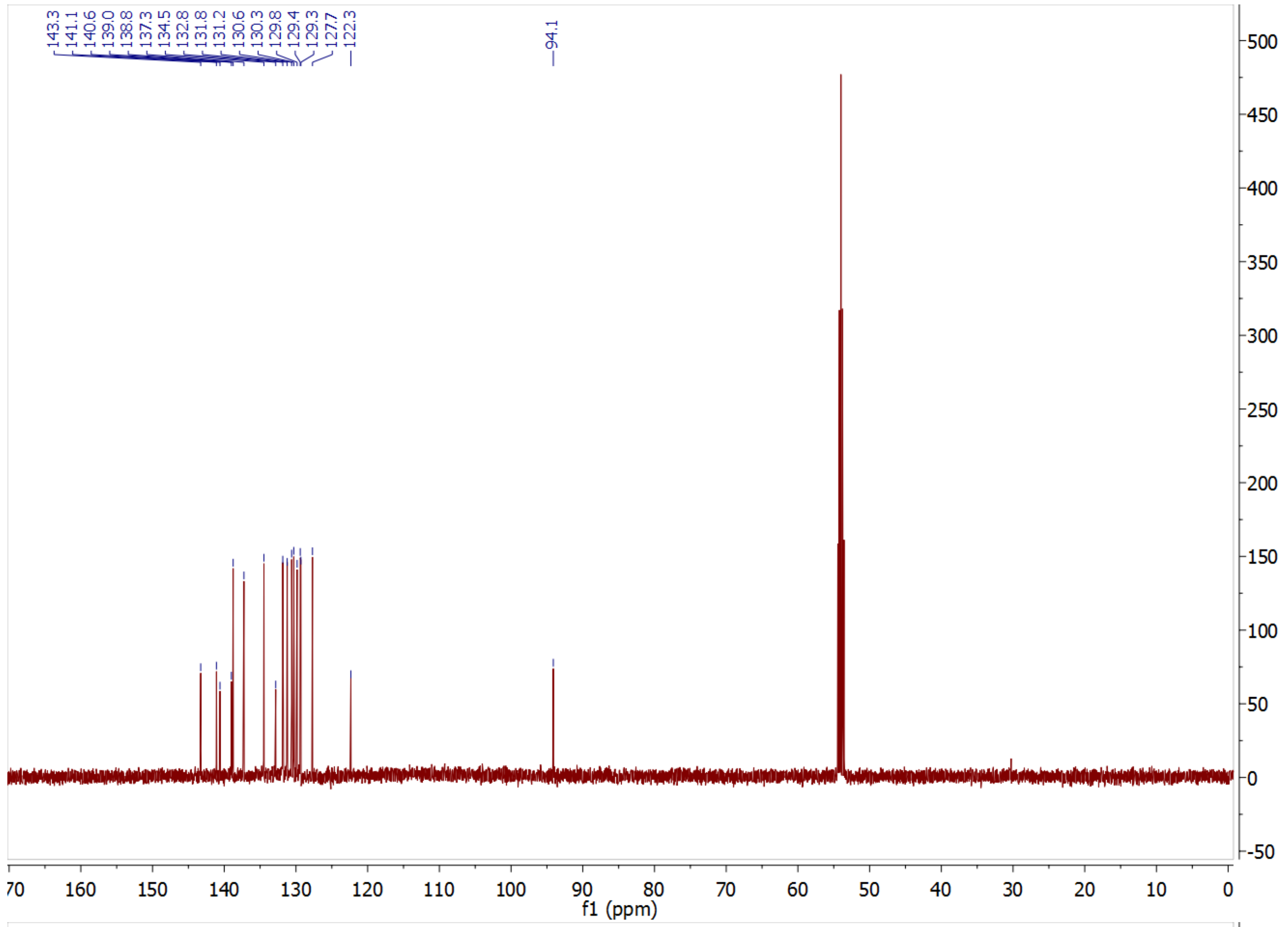
143.3
141.1
140.6
139.0
138.8
137.3
134.5
132.8
131.8
131.2
130.6
130.3
129.8
129.4
129.3
127.7
122.3
94.1
f1 (ppm)

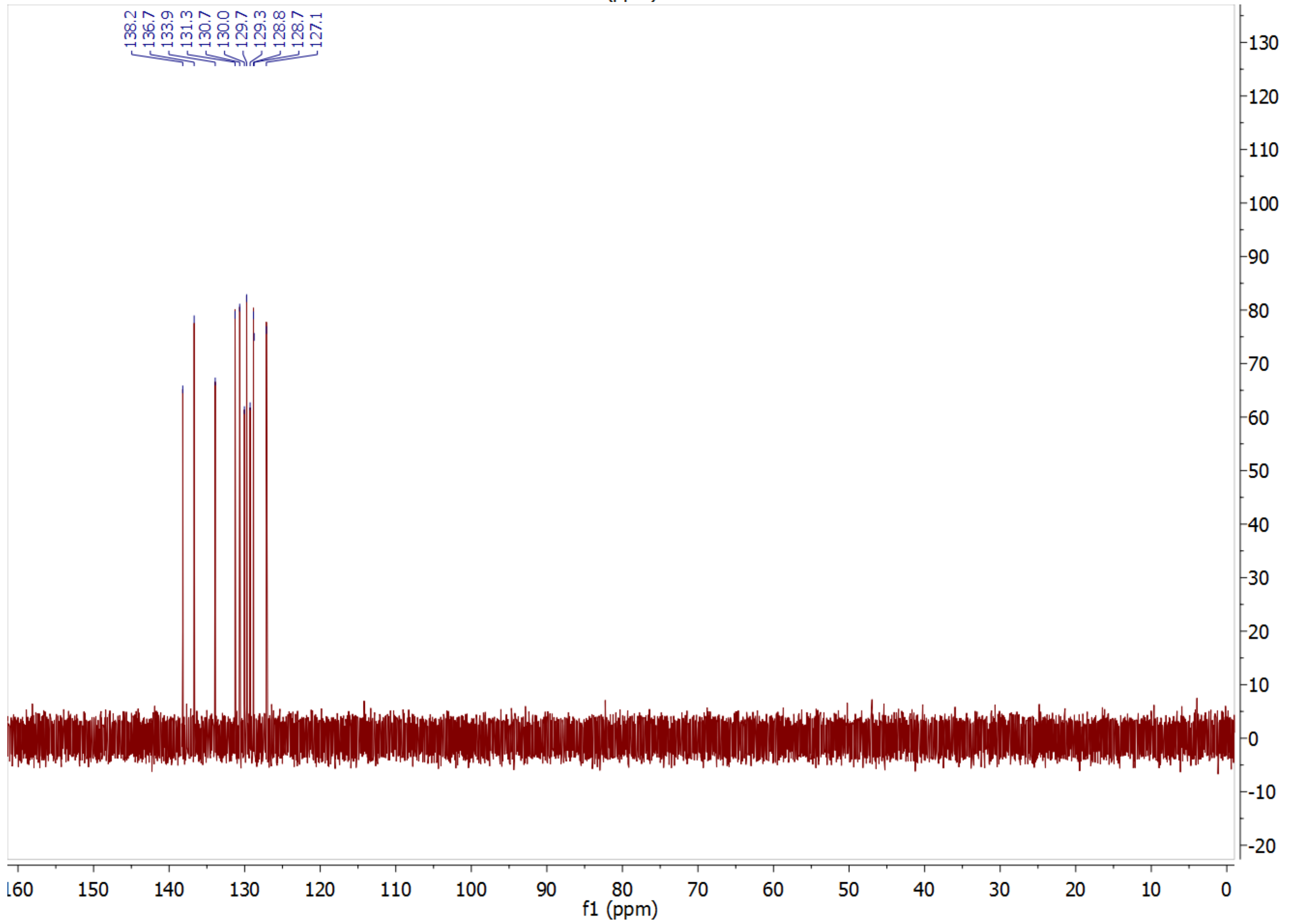
138.2
136.7
133.9
131.3
130.7
130.0
129.7
129.3
128.8
128.7
127.1
f1 (ppm)

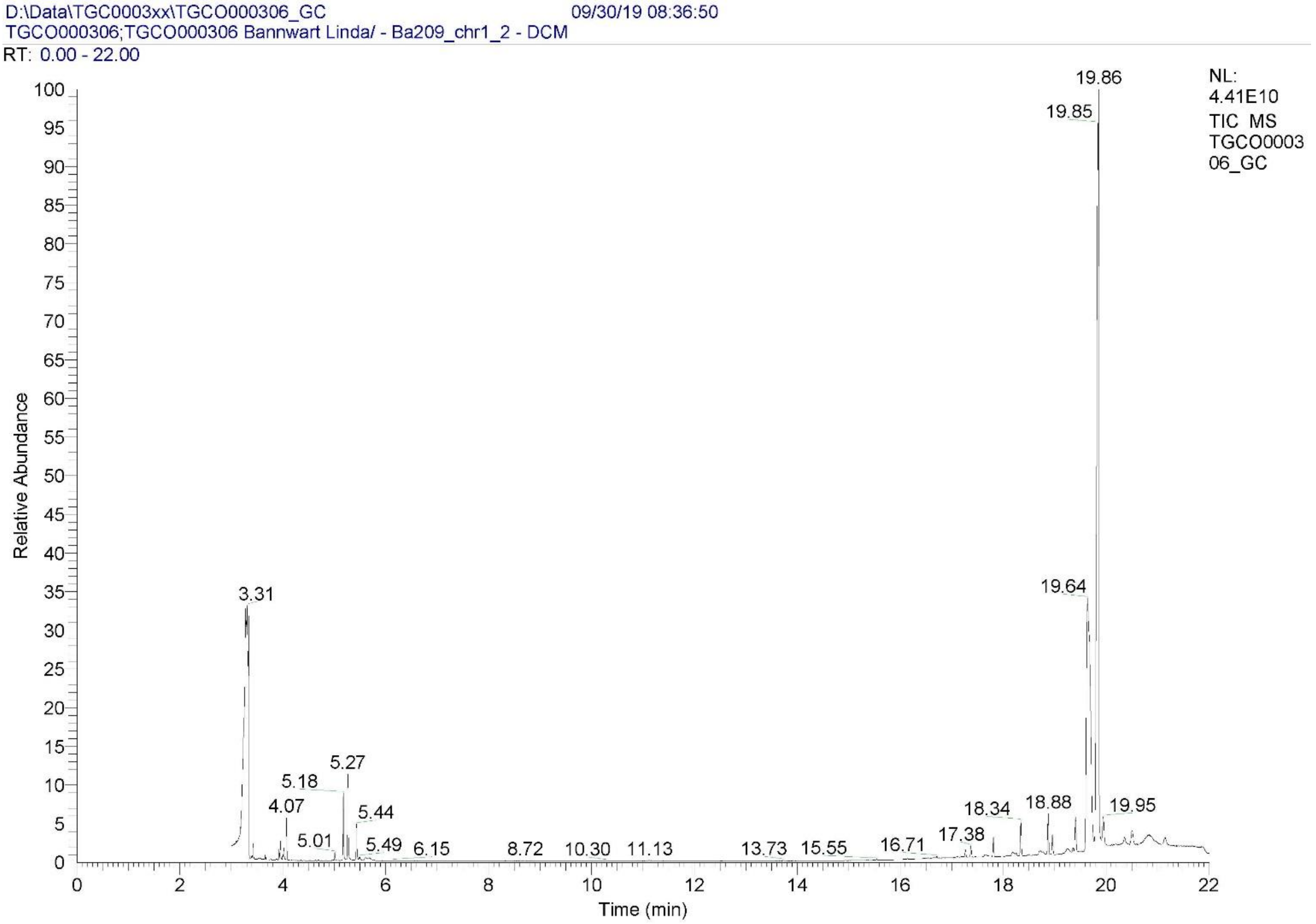
D:\Data\TGC0003xx\TGCO000306_GC 09/30/19 08:36:50
TGCO000306;TGCO000306 Bannwart Linda/ - Ba209_chr1_2 - DCM
RT: 0.00 - 22.00
NL: 4.41E10 TIC MS TGCO000306_GC
19.86
19.85
3.31
19.64
5.27
5.18
4.07
5.44
5.01
5.49
6.15
8.72
10.30
11.13
13.73
15.55
16.71
17.38
18.34
18.88
19.95
Relative Abundance
Time (min)

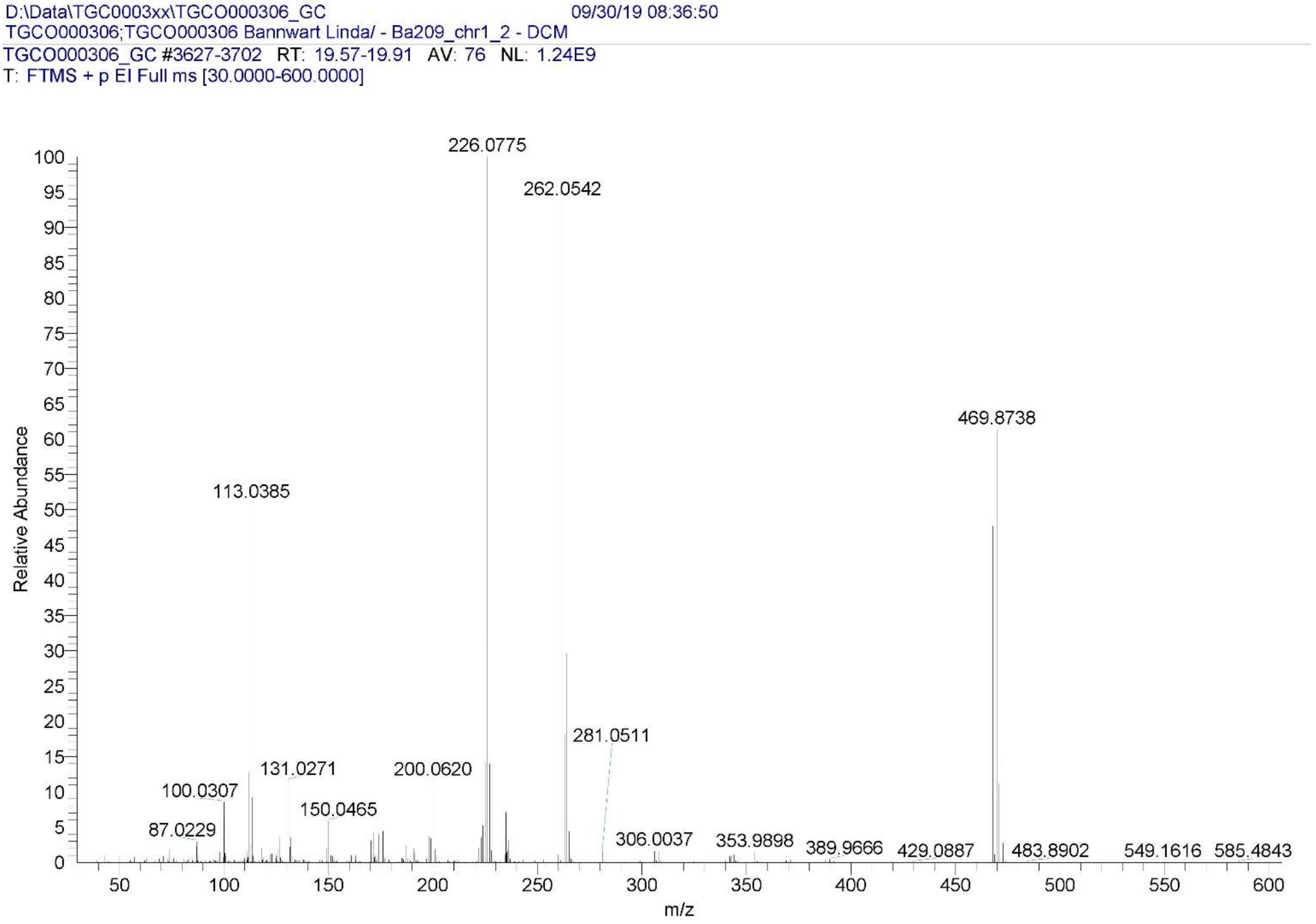
D:\Data\TGC0003xx\TGCO000306_GC 09/30/19 08:36:50
TGCO000306;TGCO000306 Bannwart Linda/ - Ba209_chr1_2 - DCM
TGCO000306_GC #3627-3702 RT: 19.57-19.91 AV: 76 NL: 1.24E9
T: FTMS + p EI Full ms [30.0000-600.0000]
226.0775
262.0542
469.8738
113.0385
281.0511
131.0271
200.0620
100.0307
150.0465
87.0229
306.0037
353.9898
389.9666
429.0887
483.8902
549.1616
585.4843
Relative Abundance
m/z

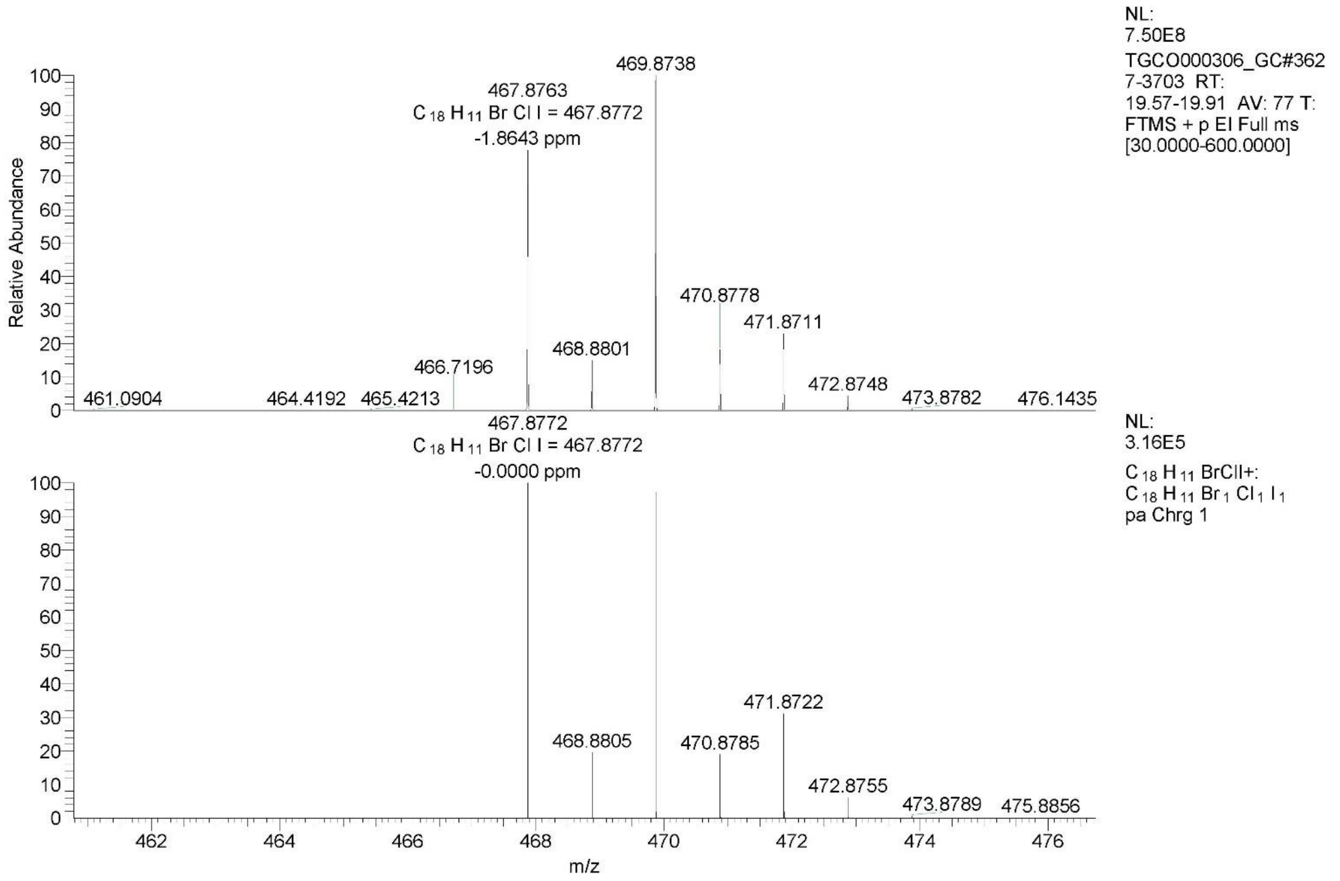
NL:
7.50E8
TGCO000306_GC#362
7-3703 RT:
19.57-19.91 AV: 77 T:
FTMS + p EI Full ms
[30.0000-600.0000]
469.8738
467.8763
C 18 H 11 Br Cl I = 467.8772
-1.8643 ppm
470.8778
471.8711
468.8801
466.7196
472.8748
461.0904
464.4192
465.4213
473.8782
476.1435
NL:
3.16E5
C 18 H 11 BrClI+:
C 18 H 11 Br 1 Cl 1 I 1
pa Chrg 1
467.8772
C 18 H 11 Br Cl I = 467.8772
-0.0000 ppm
471.8722
468.8805
470.8785
472.8755
473.8789
475.8856
Relative Abundance
m/z

5.7 Compound **12**

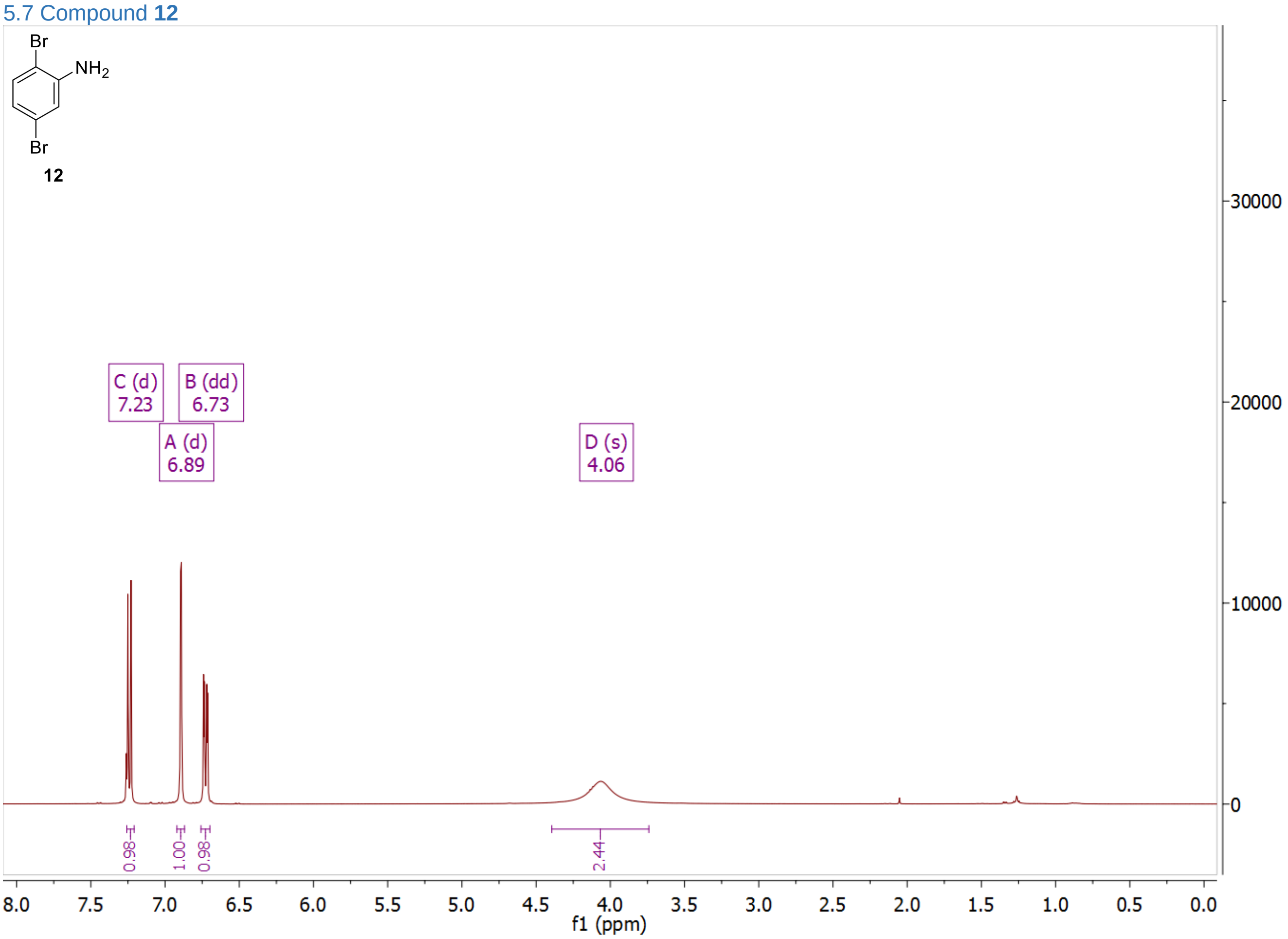

The spectra data of this compound was identical to those reported in the literature.[2]

5.8 Compound **13**

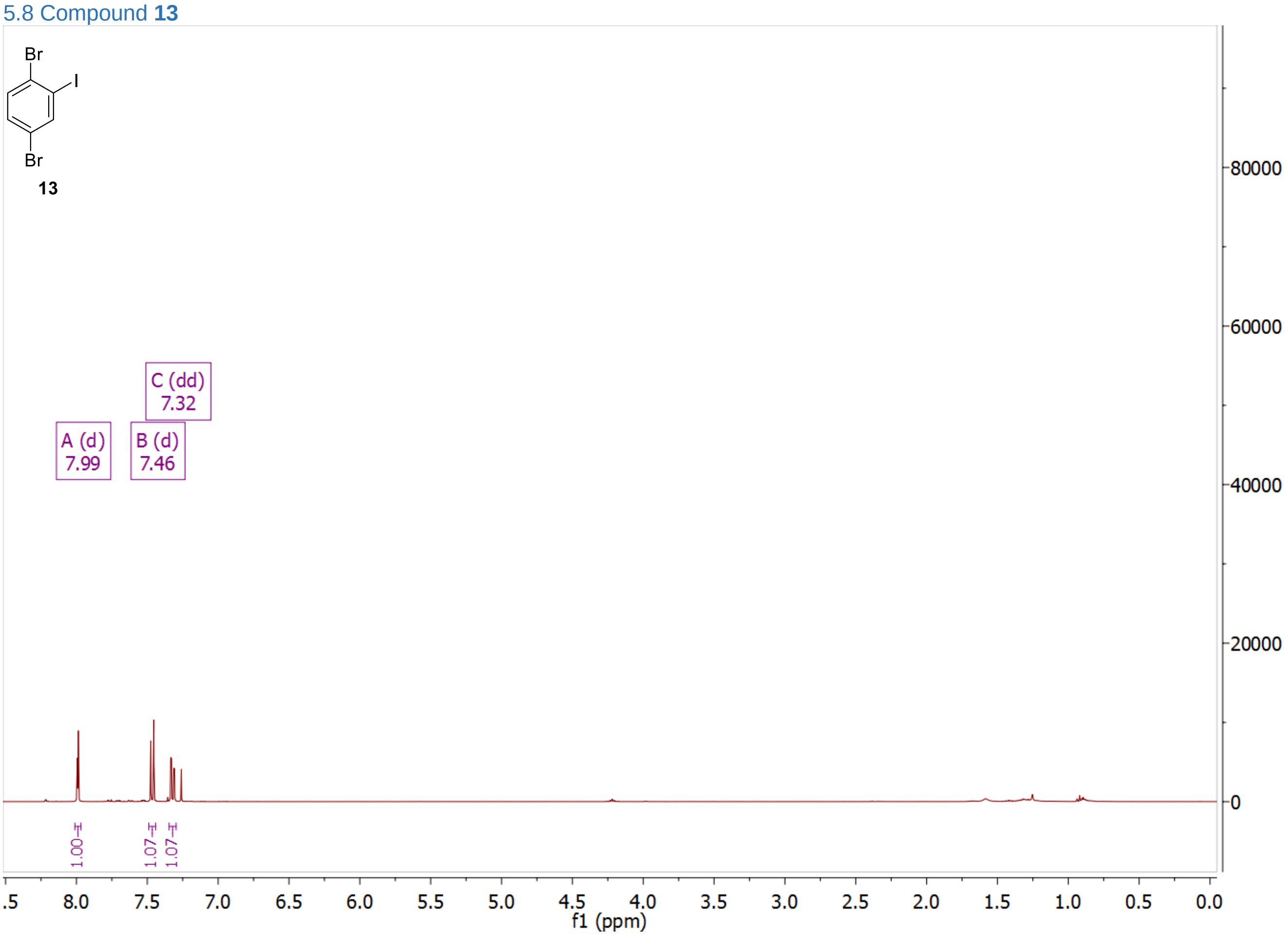

The spectra data of this compound was identical to those reported in the literature.[3]

5.9 Compound **14**

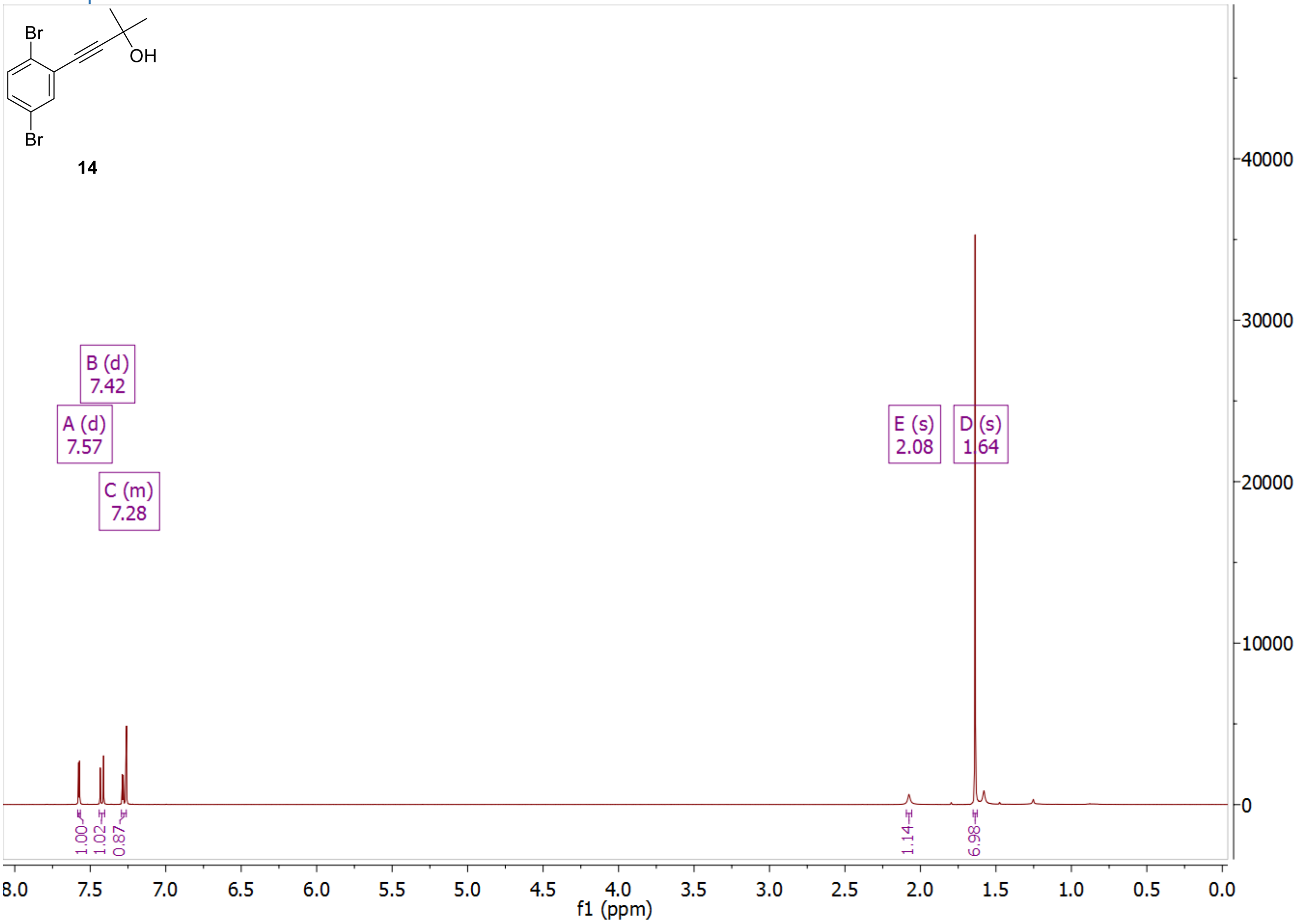

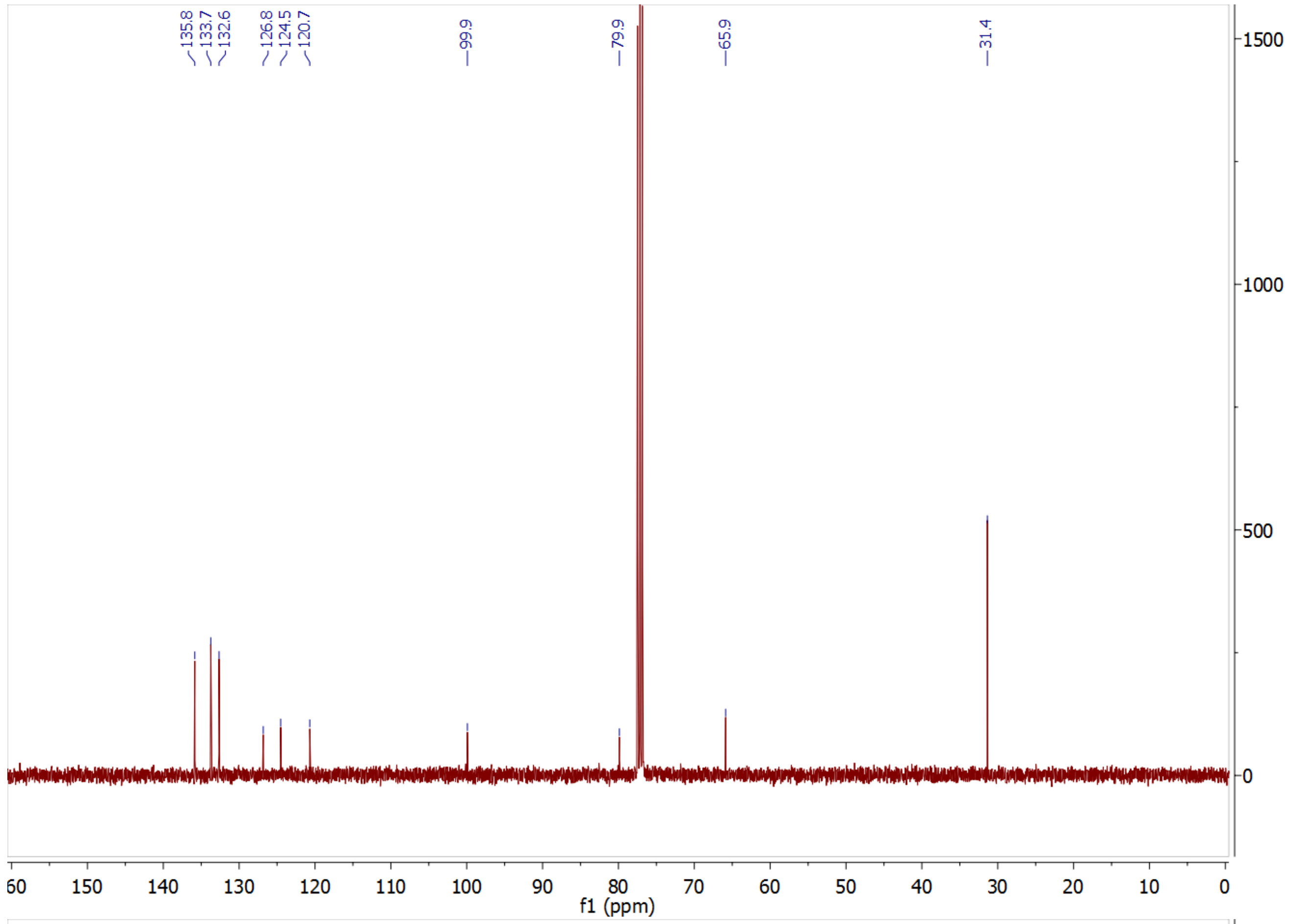
135.8
133.7
132.6
126.8
124.5
120.7
99.9
79.9
65.9
31.4
1500
1000
500
0
60
150
140
130
120
110
100
90
80
70
60
50
40
30
20
10
0
f1 (ppm)

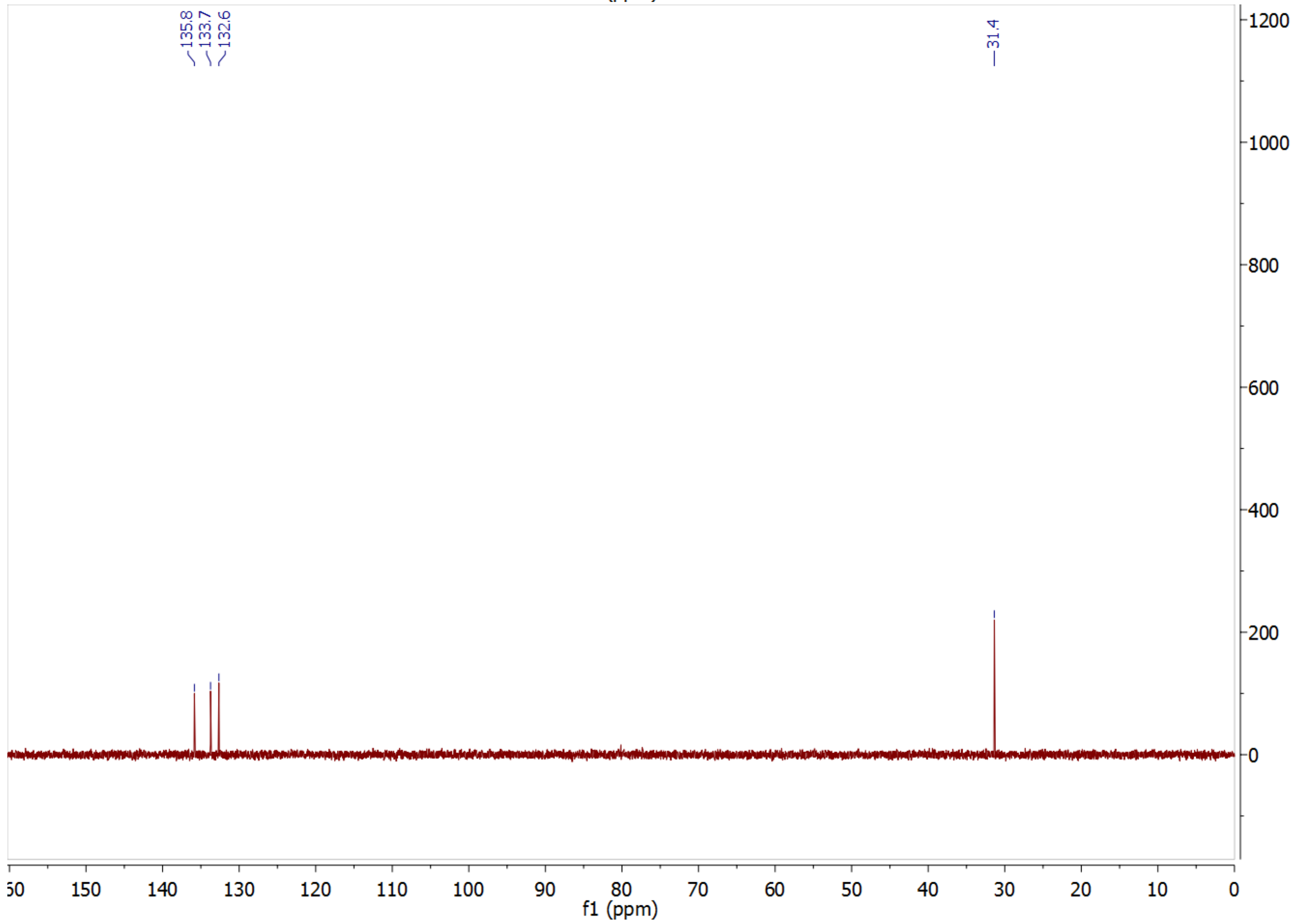
135.8
133.7
132.6
31.4
1200
1000
800
600
400
200
0
50
150
140
130
120
110
100
90
80
70
60
50
40
30
20
10
0
f1 (ppm)

Mass Spectrum SmartFormula Report

Analysis Info

Analysis Name	N:\new acq data\Ba202 chr 1_1 001.d	Acquisition Date	11.04.2016 15:55:11
Method	hn Direct_Infusion_pos mode_75-1700 mid 4eV.m	Operator	hn
Sample Name	Linda Bannwart	Instrument / Ser#	maXis 4G 21243
Comment	Ba202 chr 1#1, ca. 15 ug/ml MeCN		

Acquisition Parameter

Source Type	ESI	Ion Polarity	Positive	Set Nebulizer	0.4 Bar
Focus	Not active	Set Capillary	3600 V	Set Dry Heater	180 °C
Scan Begin	75 m/z	Set End Plate Offset	-500 V	Set Dry Gas	4.0 l/min
Scan End	1700 m/z	Set Collision Cell RF	350.0 Vpp	Set Ion Energy (MS only)	4.0 eV

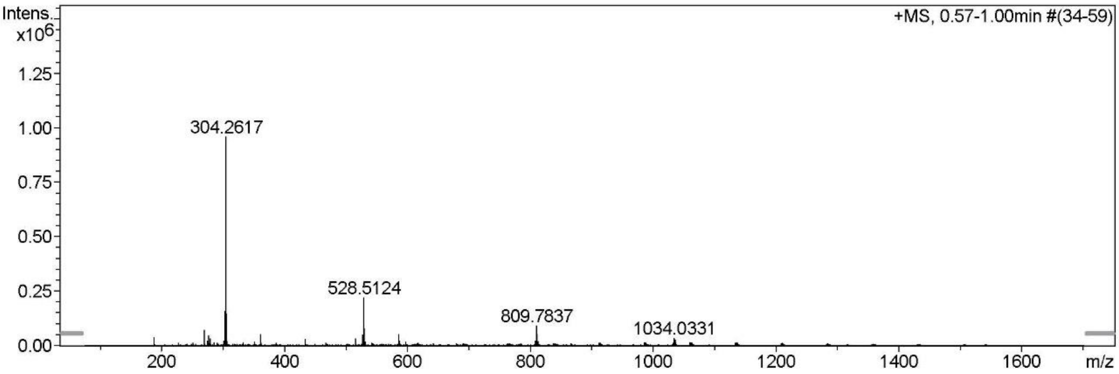

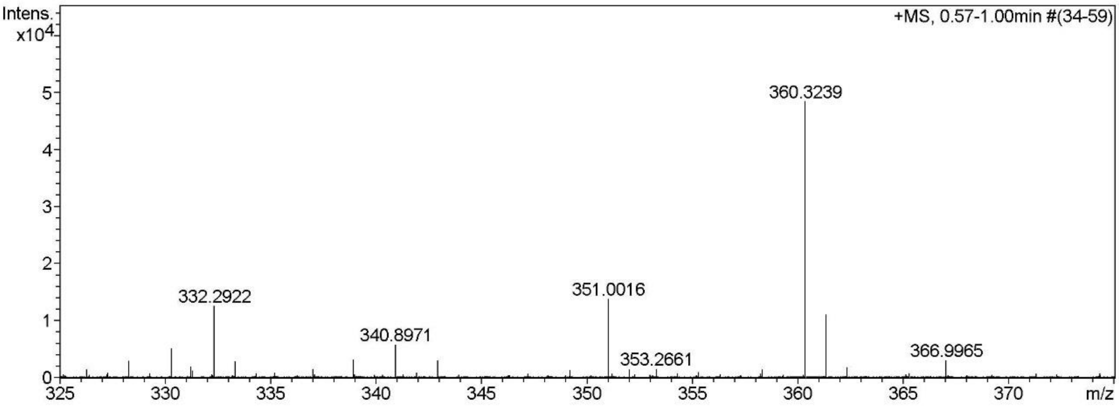

Meas. m/z	#	Formula	Score	m/z	err [mDa]	err [ppm]	mSigma	rdb	e⁻ Conf	N-Rule	z
338.8990	1	C 11 H 10 Br 2 Na O	100.00	338.8991	0.1	0.2	16.5	5.5	even	ok	1+

Mass Spectrum List Report

Analysis Info

Analysis Name	N:\new acq data\Ba202 chr 1_1 001.d	Acquisition Date	11.04.2016 15:55:11
Method	hn Direct_Infusion_pos mode_75-1700 mid 4eV.m	Operator	hn
Sample Name	Linda Bannwart	Instrument / Ser#	maXis 4G 21243
Comment	Ba202 chr 1#1, ca. 15 ug/ml MeCN		

Acquisition Parameter

Source Type	ESI	Ion Polarity	Positive	Set Nebulizer	0.4 Bar
Focus	Not active	Set Capillary	3600 V	Set Dry Heater	180 °C
Scan Begin	75 m/z	Set End Plate Offset	-500 V	Set Dry Gas	4.0 l/min
Scan End	1700 m/z	Set Collision Cell RF	350.0 Vpp	Set Ion Energy (MS only)	4.0 eV

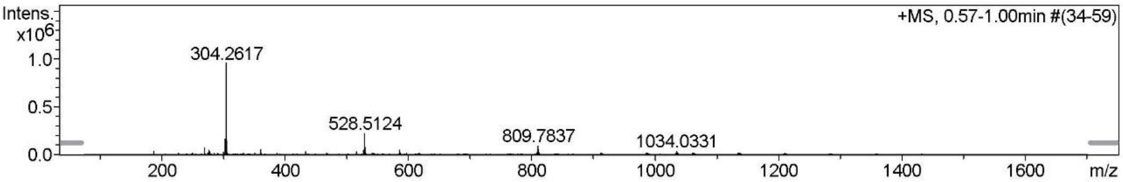

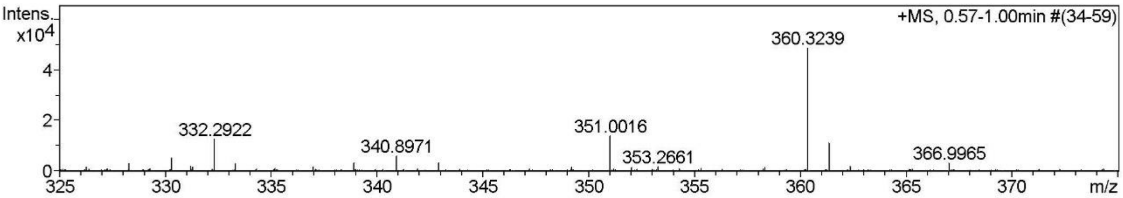

#	m/z	I %	I
1	186.9954	3.5	33517
2	226.9878	0.9	8864
3	250.2139	1.0	9365
4	268.9985	7.2	68547
5	274.2740	2.0	18982
6	276.2298	4.7	44660
7	277.2330	0.8	7875
8	278.2454	3.1	29494
9	279.2486	0.6	5427
10	284.9933	1.3	12162
11	290.2454	1.1	10439
12	292.2609	0.6	5620
13	300.2297	2.1	19760
14	301.1409	0.5	5061
15	302.2457	16.4	156946
16	303.2488	3.2	30423
17	304.2617	100.0	955497
18	305.2647	15.0	143001
19	306.2723	1.8	16807
20	322.9701	0.6	5933
21	330.2767	0.5	4971
22	332.2922	1.3	12455
23	340.8971	0.6	5669
24	351.0016	1.4	13650
25	360.3239	5.1	48303
26	361.3269	1.1	10941
27	386.2642	1.0	9372
28	433.0049	2.8	26326
29	467.1023	1.1	10277
30	502.4960	0.6	5733
31	515.0083	3.1	29392
32	526.4963	5.0	47562
33	527.4996	1.8	17474
34	528.5124	22.8	217865
35	529.5156	7.9	75554
36	530.5198	1.6	15550
37	531.0030	0.9	8251

Mass Spectrum List Report

#	m/z	I %	I
38	541.1213	1.1	10884
39	542.1221	0.6	5482
40	543.1020	0.9	8497
41	559.5171	0.6	5339
42	568.9802	0.5	4834
43	585.5335	5.1	49115
44	586.5367	2.0	19332
45	587.5439	0.7	6420
46	597.0113	1.5	13977
47	610.5149	0.5	4919
48	613.0064	0.6	5911
49	615.1401	0.8	7747
50	617.1210	0.9	8756
51	618.1211	0.5	5032
52	641.5955	0.8	7777
53	679.0144	0.8	7941
54	689.1589	0.6	5867
55	691.1401	0.9	8328
56	692.1400	0.5	5180
57	761.0173	0.6	5868
58	763.1774	0.7	6326
59	765.1608	0.8	7598
60	766.1602	0.5	5228
61	781.7520	0.6	5670
62	783.7669	0.9	8382
63	807.7678	2.1	19809
64	808.7713	1.2	11702
65	809.7837	9.2	88288
66	810.7870	5.1	48991
67	811.7920	1.9	17717
68	812.7977	0.5	5115
69	837.1963	0.8	7405
70	838.1968	0.6	5943
71	839.1836	0.8	7705
72	840.1806	0.6	5497
73	865.8456	0.8	7410
74	911.2149	1.0	9248
75	912.2154	0.8	7988
76	913.2078	0.9	8733
77	914.2040	0.6	6176
78	985.2335	1.2	11206
79	986.2341	1.1	10539
80	987.2289	1.2	11169
81	988.2263	0.8	7668
82	989.2216	0.6	5488
83	1032.0175	1.1	10566
84	1033.0208	0.8	7735
85	1034.0331	3.4	32857
86	1035.0367	2.5	23582
87	1036.0409	1.0	9835
88	1059.2522	1.2	11420
89	1060.2529	1.2	11654
90	1061.2489	1.4	13031
91	1062.2467	0.9	8819
92	1063.2424	0.6	6136
93	1133.2713	1.0	9934
94	1134.2719	1.2	11021
95	1135.2682	1.2	11894
96	1136.2664	0.9	8804
97	1137.2631	0.6	6104
98	1207.2903	0.8	7346
99	1208.2905	0.9	8320
100	1209.2870	1.0	9785
101	1210.2858	0.8	7577
102	1211.2820	0.6	5551
103	1282.3100	0.6	5678
104	1283.3057	0.7	6878
105	1284.3040	0.6	5587

5.10 Compound **15**

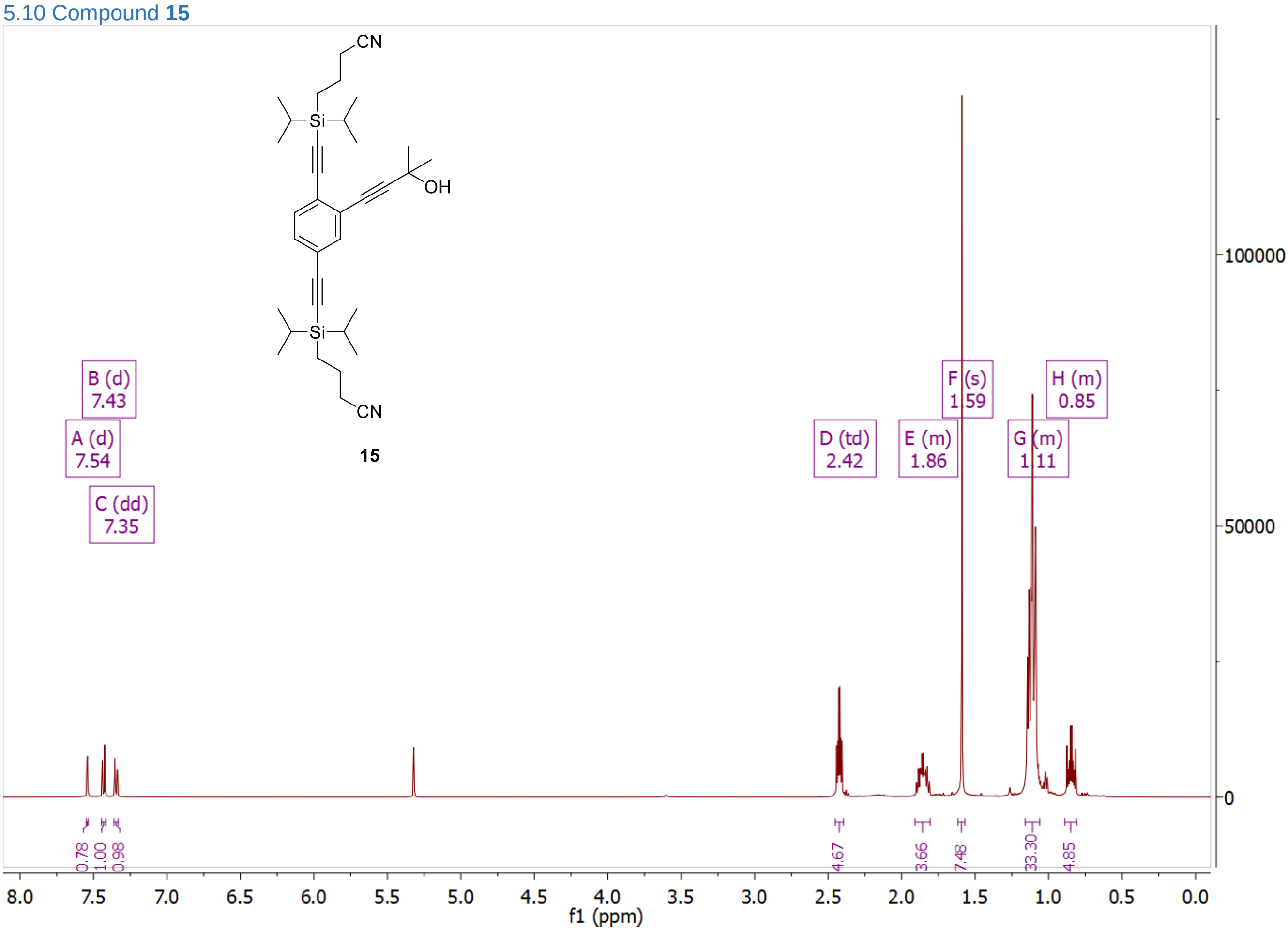

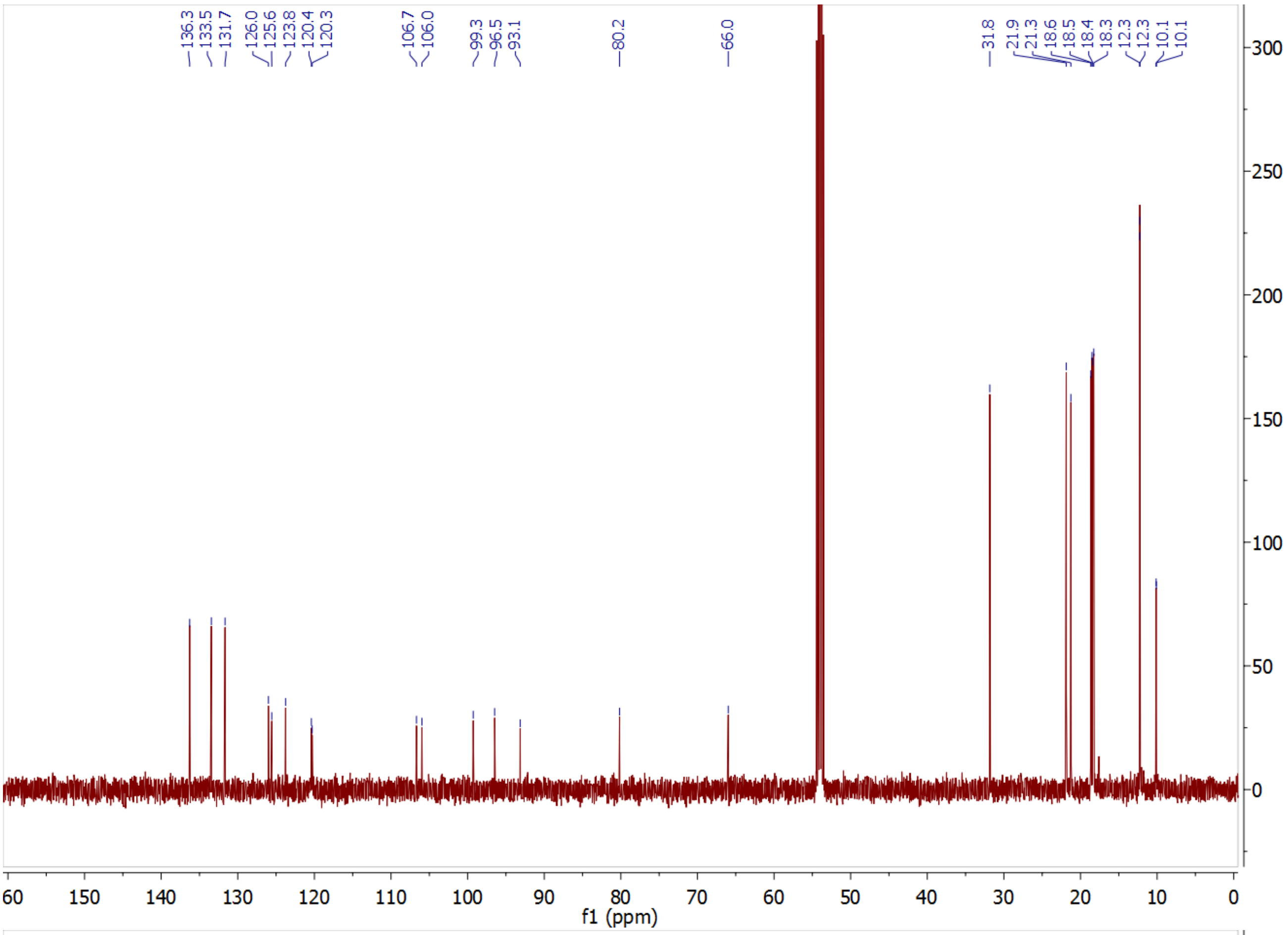
136.3
133.5
131.7
126.0
125.6
123.8
120.4
120.3
106.7
106.0
99.3
96.5
93.1
80.2
66.0
31.8
21.9
21.3
18.6
18.5
18.4
18.3
12.3
12.3
10.1
10.1
f1 (ppm)

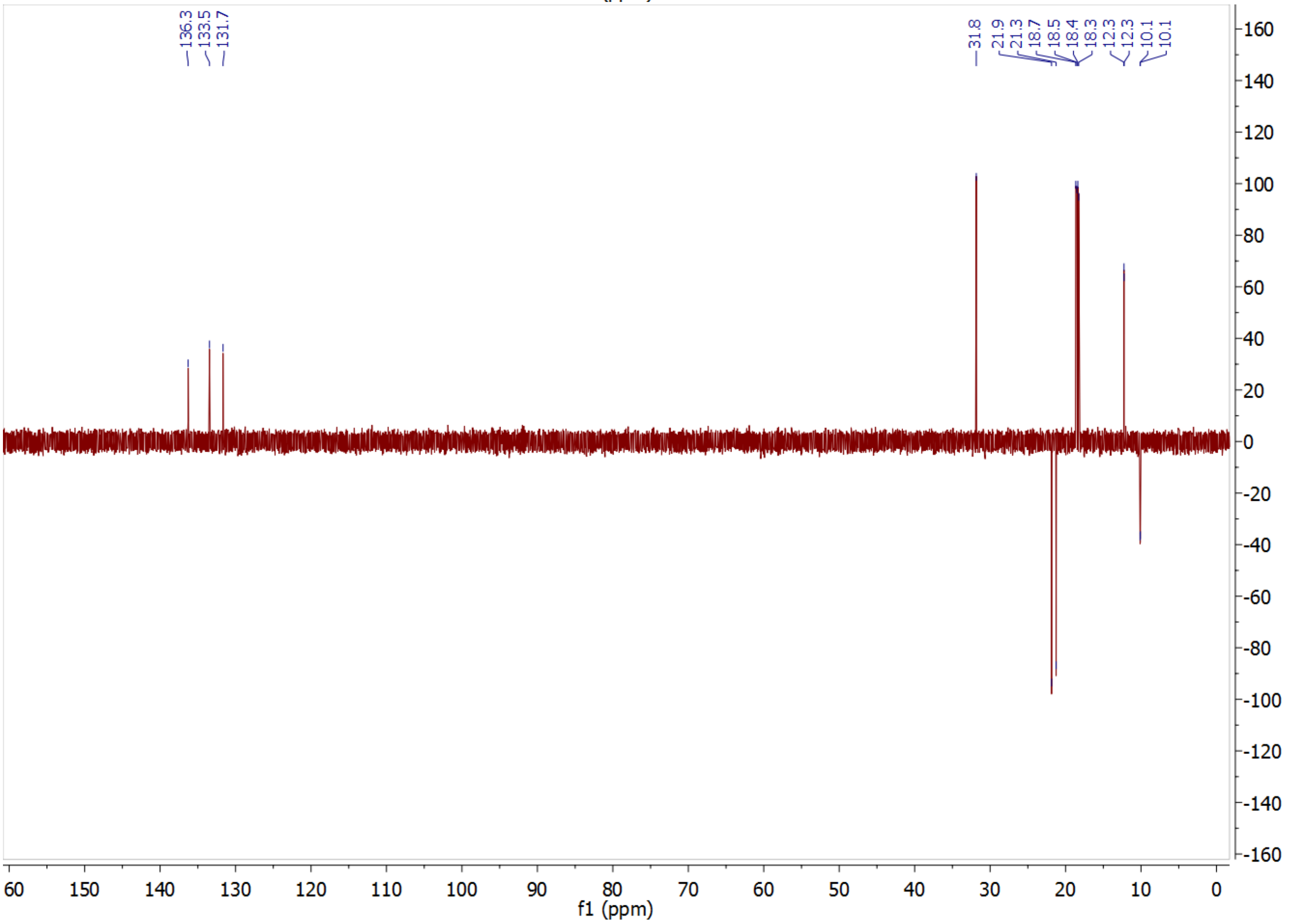
136.3
133.5
131.7
31.8
21.9
21.3
18.7
18.5
18.4
18.3
12.3
12.3
10.1
10.1
f1 (ppm)

High Resolution Mass Spectrometry Report

Sample Name **Ba217 chr1#1**
Comment

Instrument maXis 4G
Method ms_nocolumn_300-600_pos.m

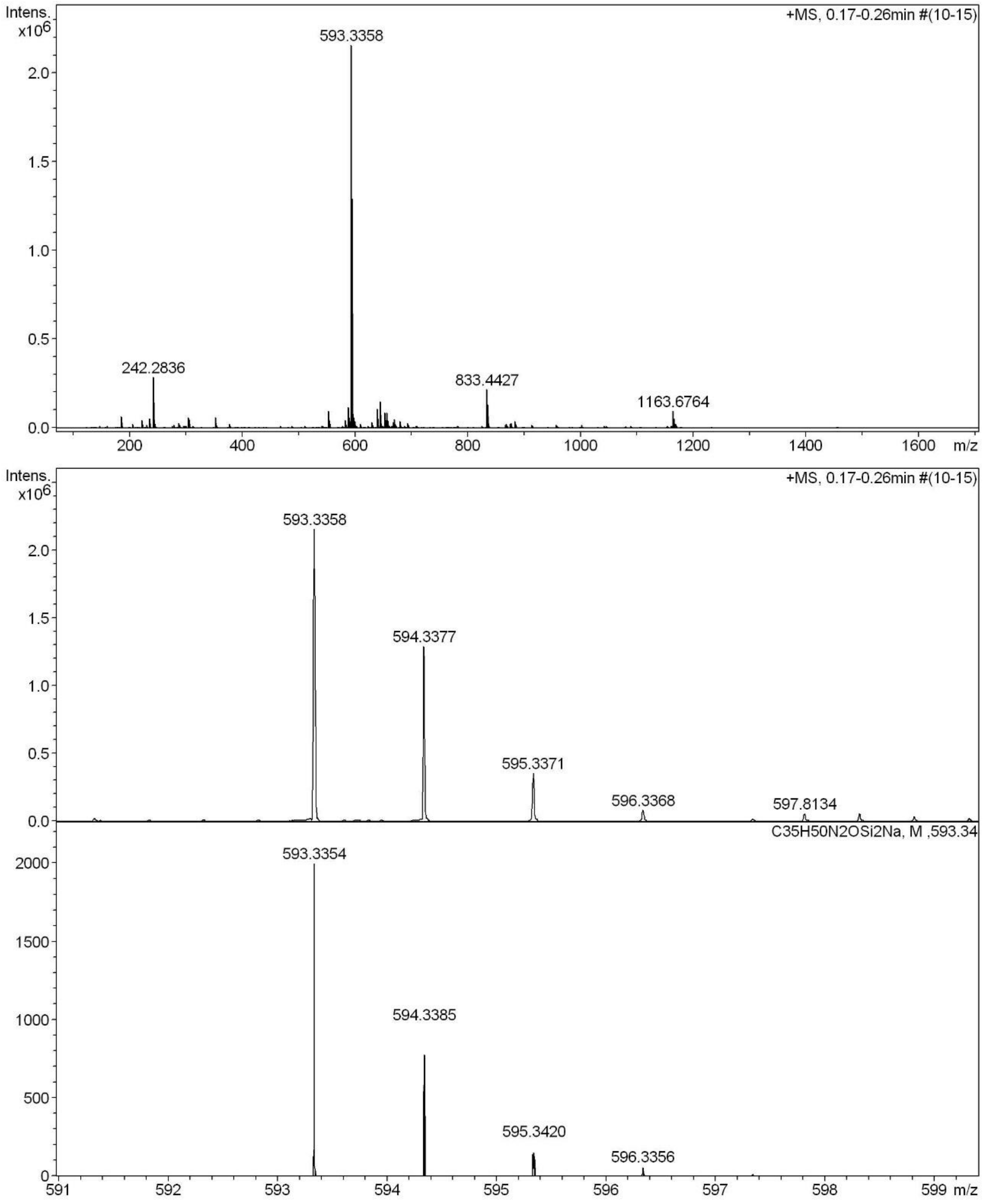

High Resolution Mass Spectrometry Report

Measured m/z vs. theoretical m/z

Meas. m/z	#	Formula	Score	m/z	err [mDa]	err [ppm]	mSigma	rdb	e⁻ Conf	z
593.3358	1	C 35 H 50 N 2 Na O Si 2	100.00	593.3354	-0.4	-0.6	48.6	13.5	even	1+

Mass list

#	m/z	I %	I
1	185.1142	2.9	62888
2	205.0591	0.9	20126
3	222.1275	2.0	42458
4	229.8924	0.8	16310
5	236.0706	2.6	55587
6	242.2836	13.3	286833
7	243.2866	2.3	50618
8	244.8677	0.6	13590
9	279.0921	0.8	17527
10	288.1590	1.2	24843
11	288.6598	0.7	14239
12	297.1639	0.6	13931
13	303.6355	2.8	60935
14	304.1366	1.3	27421
15	305.1533	2.2	46805
16	305.6541	0.9	20452
17	353.1441	2.6	57006
18	354.1473	0.7	15943
19	377.2039	1.1	24285
20	553.3414	4.5	96807
21	554.3439	2.0	44080
22	555.3432	0.7	14757
23	582.3368	1.7	37156
24	582.8380	2.0	42867
25	583.3380	1.4	30686
26	583.8380	0.8	17621
27	588.3785	5.5	119073
28	589.3809	2.7	57400
29	590.3256	1.9	40865
30	590.3804	0.9	19144
31	590.8270	1.8	38192
32	591.3269	1.2	25255
33	593.2971	1.1	24146
34	593.3358	100.0	2156668
35	594.3377	60.0	1294440
36	595.3371	16.6	358333
37	596.3368	3.8	82207
38	597.3370	0.8	17104
39	597.8134	2.8	59918
40	598.3149	2.7	59044
41	598.8153	1.7	36031
42	599.3166	0.9	19777
43	600.3244	0.7	14321
44	609.3072	1.0	21019
45	622.3390	0.6	13408
46	630.4246	1.6	33817
47	631.4273	0.8	17825
48	639.3264	1.1	22916
49	639.3892	5.0	107552
50	640.3283	0.7	14658
51	640.3916	2.6	55200
52	641.3918	0.9	19051
53	644.3517	0.7	14517
54	644.4413	6.8	146892
55	645.4436	3.5	75943
56	646.4433	1.2	26277
57	653.4049	4.0	85346
58	654.4072	2.1	44436
59	655.3039	0.8	17583
60	655.4073	0.8	16268
61	656.4408	4.1	87480
62	657.4435	2.1	44463

#	m/z	I %	I
63	658.4434	0.8	17282
64	667.4196	1.4	30064
65	668.4223	0.8	16218
66	670.2794	2.3	49829
67	670.4561	1.4	30625
68	671.2818	1.1	24492
69	671.4586	0.8	17045
70	679.4205	1.7	37166
71	680.4216	1.0	21706
72	693.4354	1.3	28596
73	694.4353	0.8	16285
74	833.4427	10.0	216517
75	834.4452	6.1	132445
76	835.4503	3.2	69242
77	836.4554	1.3	28880
78	867.5089	0.7	14955
79	868.0103	1.1	23403
80	868.5108	1.0	22504
81	869.0110	0.8	16957
82	869.4792	0.6	13629
83	875.4978	0.9	19790
84	875.9991	1.2	26757
85	876.4996	1.1	22986
86	876.9997	0.7	15684
87	882.9855	1.2	25329
88	883.4868	1.6	35372
89	883.9872	1.4	31063
90	884.4877	0.9	20037
91	913.5035	0.7	15177
92	957.5295	0.7	14829
93	1001.5562	0.8	16556
94	1163.6764	4.4	95047
95	1164.6792	4.1	88644
96	1165.6798	2.4	52166
97	1166.6800	1.1	23394
98	1168.6596	0.9	19733
99	1169.1594	0.9	19577
100	1169.6607	0.8	17271

Acquisition Parameter

**General**	Fore Vacuum	2.60e+000 mBar	High Vacuum	1.36e-007 mBar	Source Type	ESI
	Scan Begin	75 m/z	Scan End	1700 m/z	Ion Polarity	Positive
**Source**	Set Nebulizer	2.0 Bar	Set Capillary	4500 V	Set Dry Gas	8.0 l/min
	Set Dry Heater	200 °C	Set End Plate Offset	-500 V		
**Quadrupole**	Set Ion Energy (MS only)	4.0 eV				
**Coll. Cell**	Collision Energy	8.0 eV	Set Collision Cell RF	350.0 Vpp		
**Ion Cooler**	Set Ion Cooler Transfer Time	75.0 µs	Set Ion Cooler Pre Pulse Storage Time	10.0 µs		

5.11 Compound **16**

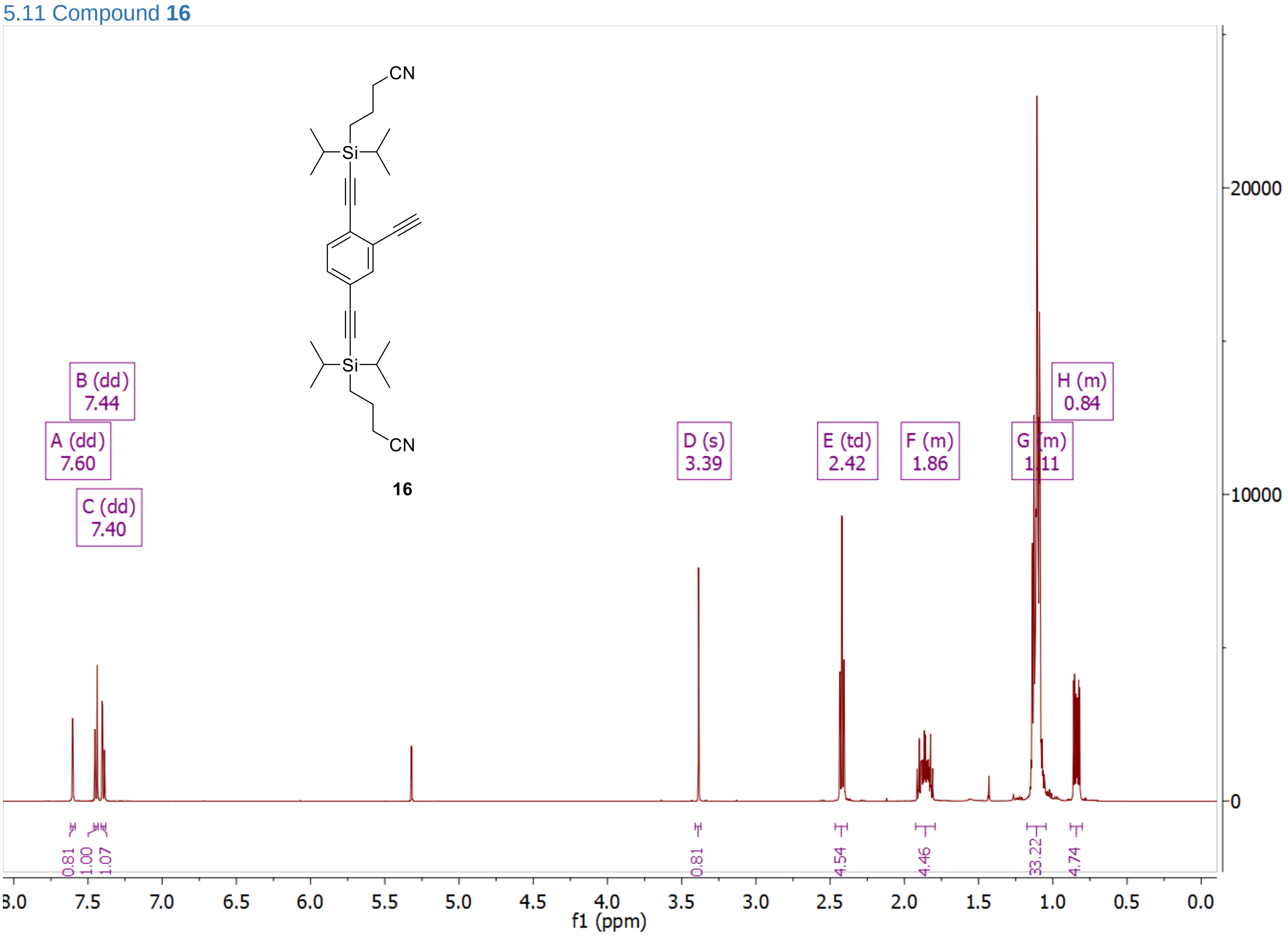

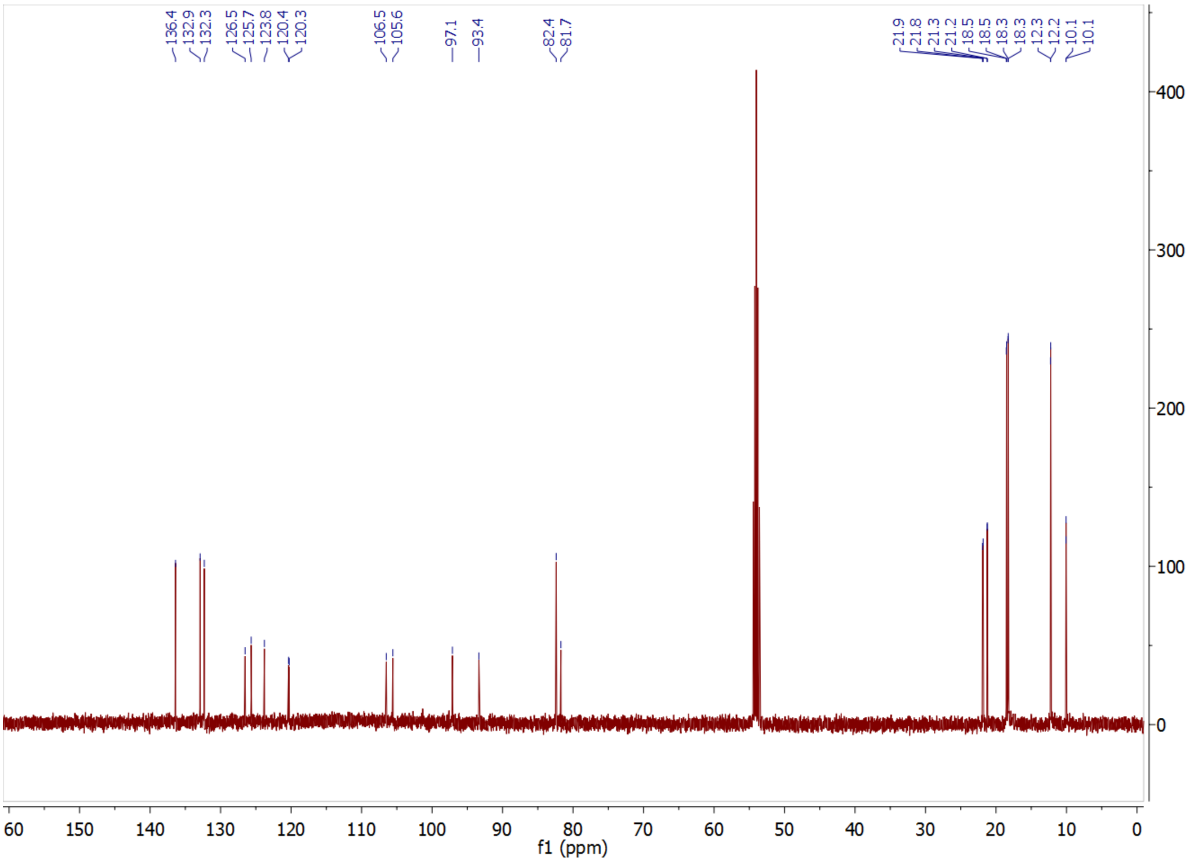

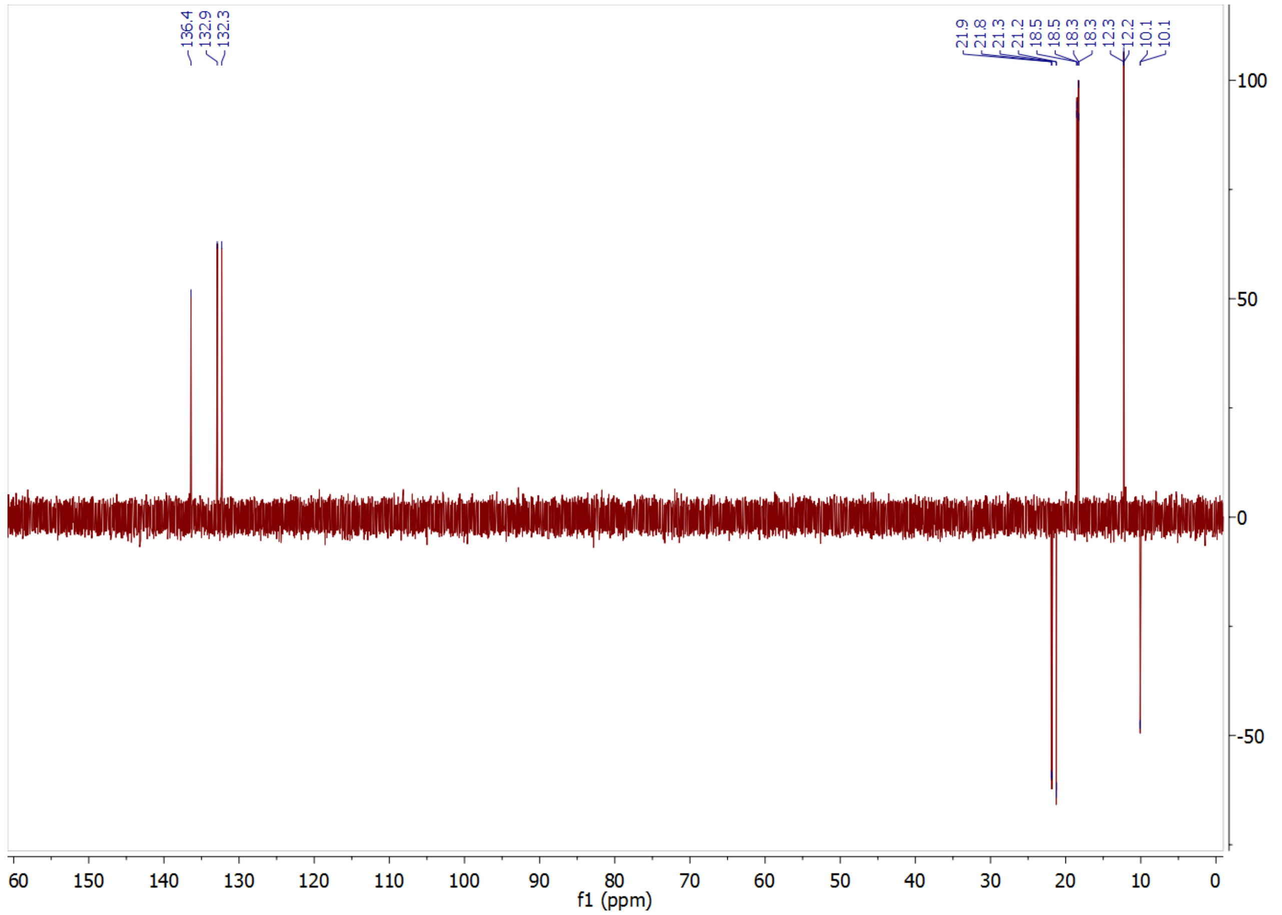

DEPT-135: $^{1}J_{CH}$ coupling= 145 Hz

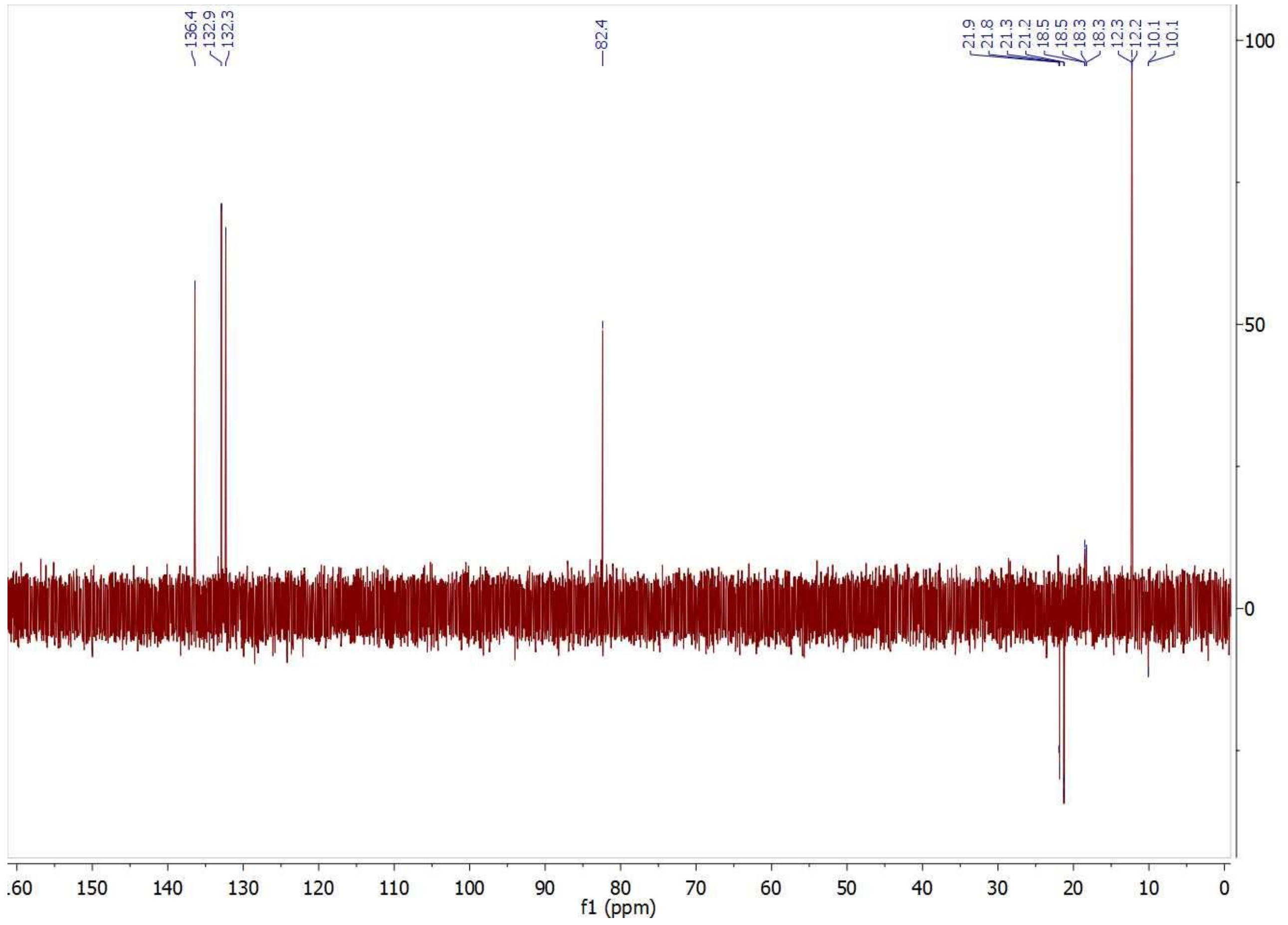

DEPT-135: $^{1}J_{CH}$ coupling= 200 Hz

High Resolution Mass Spectrometry Report

Sample Name **Ba221 chr1#2**
Comment

Instrument maXis 4G
Method ms_nocolumn_300-600_pos.m

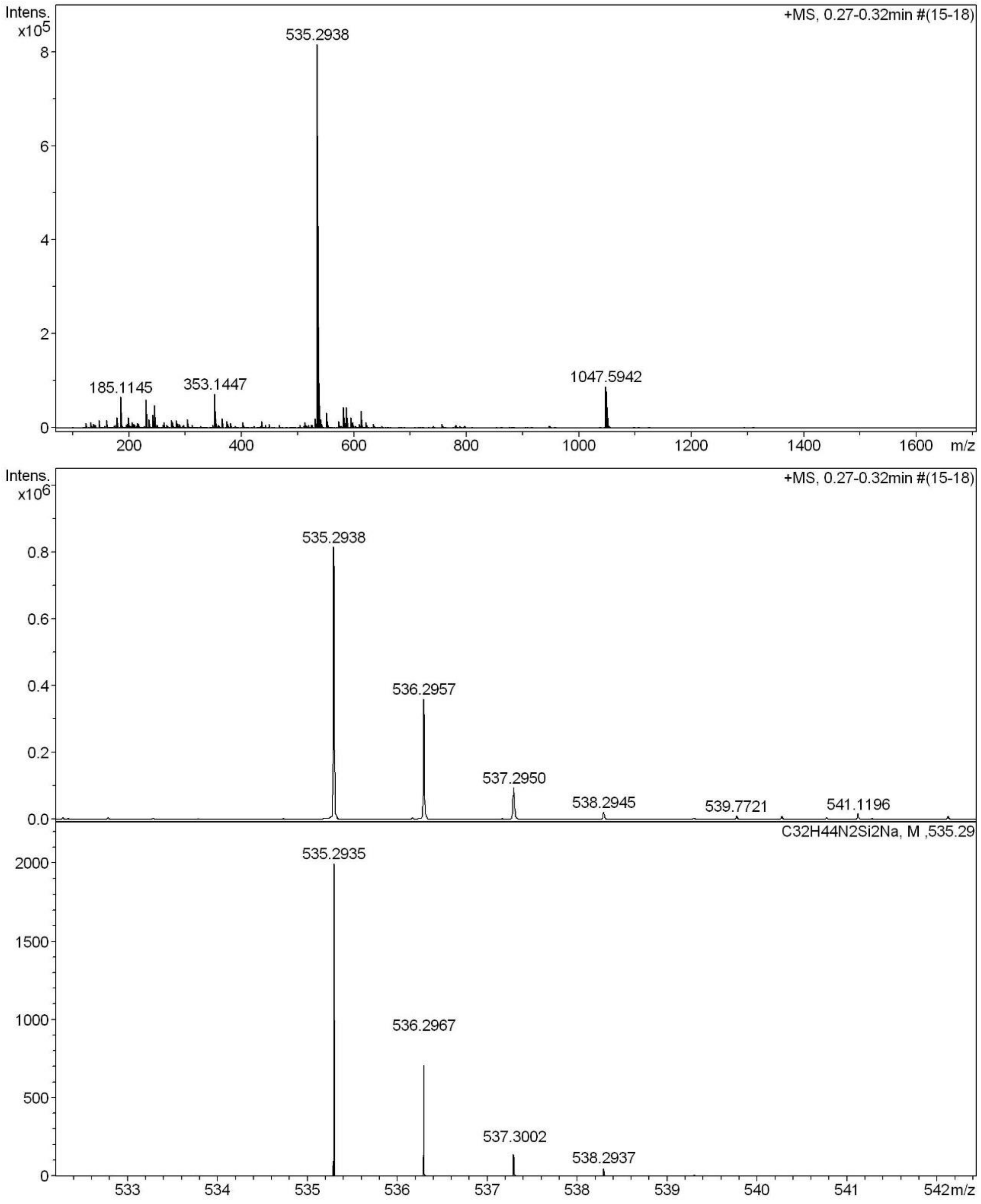

High Resolution Mass Spectrometry Report

Measured m/z vs. theoretical m/z

Meas. m/z	#	Formula	Score	m/z	err [mDa]	err [ppm]	mSigma	rdb	e¯ Conf	z
535.2938	1	C 32 H 44 N 2 Na Si 2	100.00	535.2935	-0.3	-0.6	24.2	13.5	even	1+

Mass list

#	m/z	I %	I
1	123.0922	1.2	9748
2	131.9618	1.6	13093
3	137.1076	1.0	8362
4	138.0916	0.8	6652
5	147.0919	2.0	16662
6	161.1073	2.0	16647
7	178.9953	2.8	22677
8	185.1145	8.0	65582
9	187.1226	1.1	8696
10	197.0058	0.9	7504
11	199.1300	2.6	21500
12	201.1381	0.8	6552
13	205.0596	1.6	12746
14	207.0167	0.8	6545
15	209.0059	1.0	8313
16	214.9170	1.2	9524
17	217.1040	0.9	7353
18	229.8927	7.5	61201
19	231.1070	1.0	8418
20	236.0709	2.3	18767
21	242.2836	3.5	28998
22	244.8680	5.9	48010
23	247.0850	1.1	8793
24	247.9034	1.0	8122
25	262.0604	1.0	8365
26	262.8785	1.5	12614
27	268.1437	0.9	7161
28	275.1609	1.5	12184
29	276.1324	2.0	16516
30	276.6335	0.8	6733
31	276.8942	1.6	13249
32	283.6201	2.0	16472
33	284.1214	1.0	7769
34	285.1376	1.2	9625
35	288.9215	1.0	7834
36	291.1559	0.8	6559
37	297.8799	0.8	6616
38	303.8969	2.3	18494
39	312.8554	0.9	7027
40	353.1447	9.0	73294
41	354.1481	2.4	19499
42	359.8501	0.9	7163
43	365.1046	2.5	20119
44	374.8255	1.7	13822
45	375.8224	0.9	7758
46	380.8425	1.2	9856
47	403.2560	1.6	12847
48	435.2611	1.7	13593
49	448.8298	0.9	7598
50	467.1005	0.9	6957
51	504.7831	0.9	6970
52	513.3104	1.6	13028
53	519.7589	0.9	7258
54	524.2950	0.9	7081
55	524.7965	0.8	6413
56	530.3369	2.5	20532
57	531.3395	1.2	9991
58	532.2846	1.0	7877
59	532.7856	0.9	7649
60	535.2938	100.0	817029
61	536.2957	44.4	362495
62	537.2950	11.8	96198

High Resolution Mass Spectrometry Report

#	m/z	I %	I
63	538.2945	2.8	22897
64	539.7721	1.4	11512
65	540.2736	1.2	9853
66	541.1196	2.1	17456
67	542.1203	1.1	8725
68	551.2662	3.9	31752
69	552.2688	1.8	14455
70	553.2667	0.8	6881
71	572.3592	1.8	14299
72	572.3822	1.6	12953
73	573.3616	0.8	6735
74	581.2850	2.6	21177
75	581.3480	5.5	44797
76	582.2872	1.6	12685
77	582.3503	2.6	21644
78	583.2856	1.0	8061
79	583.3500	0.9	7375
80	586.3997	5.5	44785
81	587.4021	2.7	21676
82	588.4014	0.8	6813
83	595.3632	2.7	21736
84	596.3654	1.4	11092
85	597.2627	1.6	12727
86	598.2649	0.8	6426
87	598.3988	0.9	7384
88	609.3784	1.0	7909
89	612.2382	4.5	36982
90	613.2405	2.0	16463
91	621.3290	0.9	7366
92	621.3792	1.5	12064
93	622.3802	0.8	6620
94	635.3938	1.1	8912
95	757.1710	0.9	7639
96	1047.5942	10.9	89213
97	1048.5967	9.7	79244
98	1049.5970	5.4	44098
99	1050.5972	2.2	18054
100	1051.5975	0.8	6829

Acquisition Parameter

**General**	Fore Vacuum	2.60e+000 mBar	High Vacuum	1.26e-007 mBar	Source Type	ESI
	Scan Begin	75 m/z	Scan End	1700 m/z	Ion Polarity	Positive
**Source**	Set Nebulizer	2.0 Bar	Set Capillary	4500 V	Set Dry Gas	8.0 l/min
	Set Dry Heater	200 °C	Set End Plate Offset	-500 V		
**Quadrupole**	Set Ion Energy (MS only)	4.0 eV				
**Coll. Cell**	Collision Energy	8.0 eV	Set Collision Cell RF	350.0 Vpp		
**Ion Cooler**	Set Ion Cooler Transfer Time	75.0 µs	Set Ion Cooler Pre Pulse Storage Time	10.0 µs		

5.12 Compound **E**

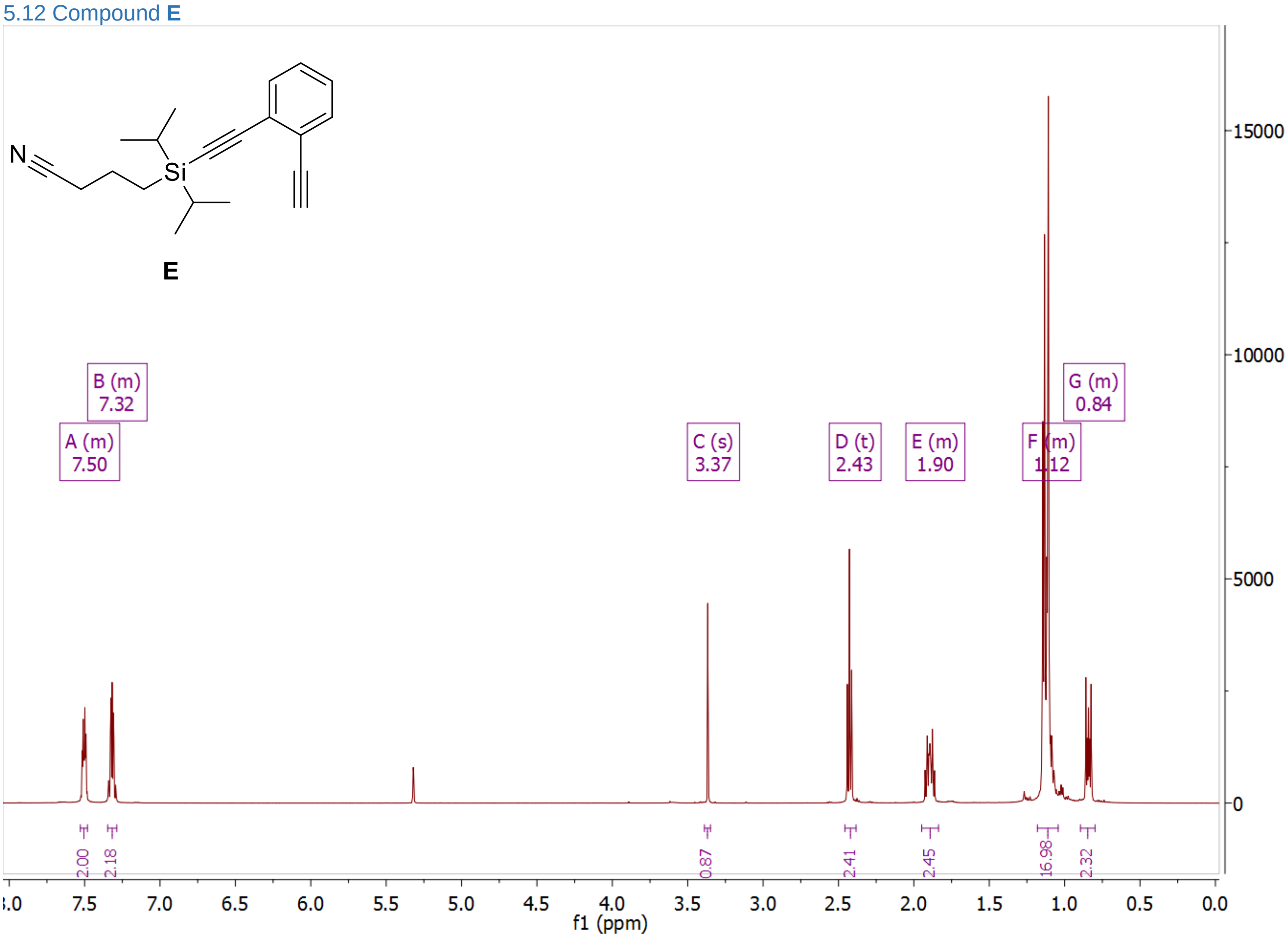

The spectra data of this compound was identical to those reported in the literature.[5]

5.13 Compound **17**

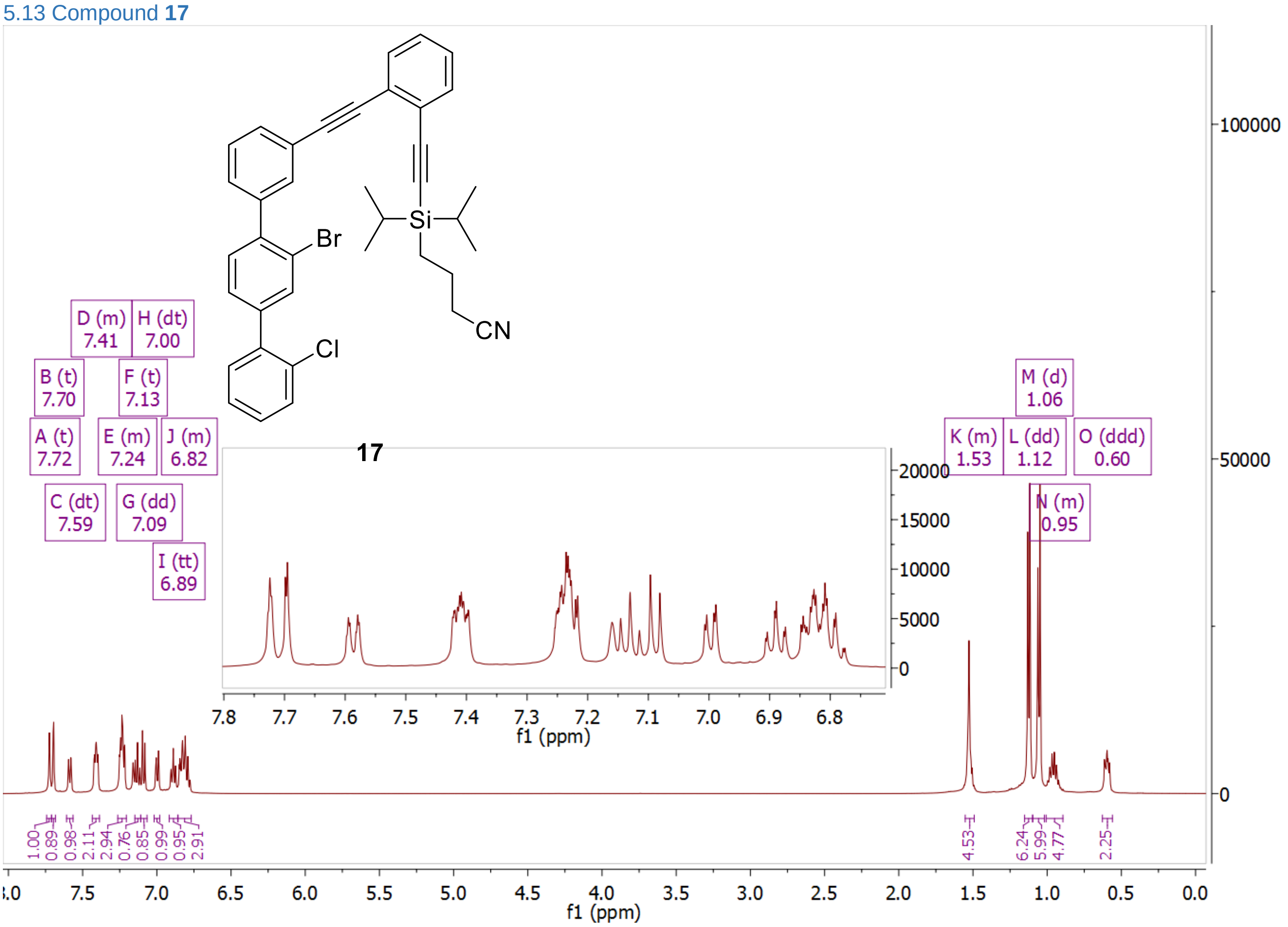

[1]H in benzene-d_6 (above) and ^{13}C in benzene-d_6 (below)

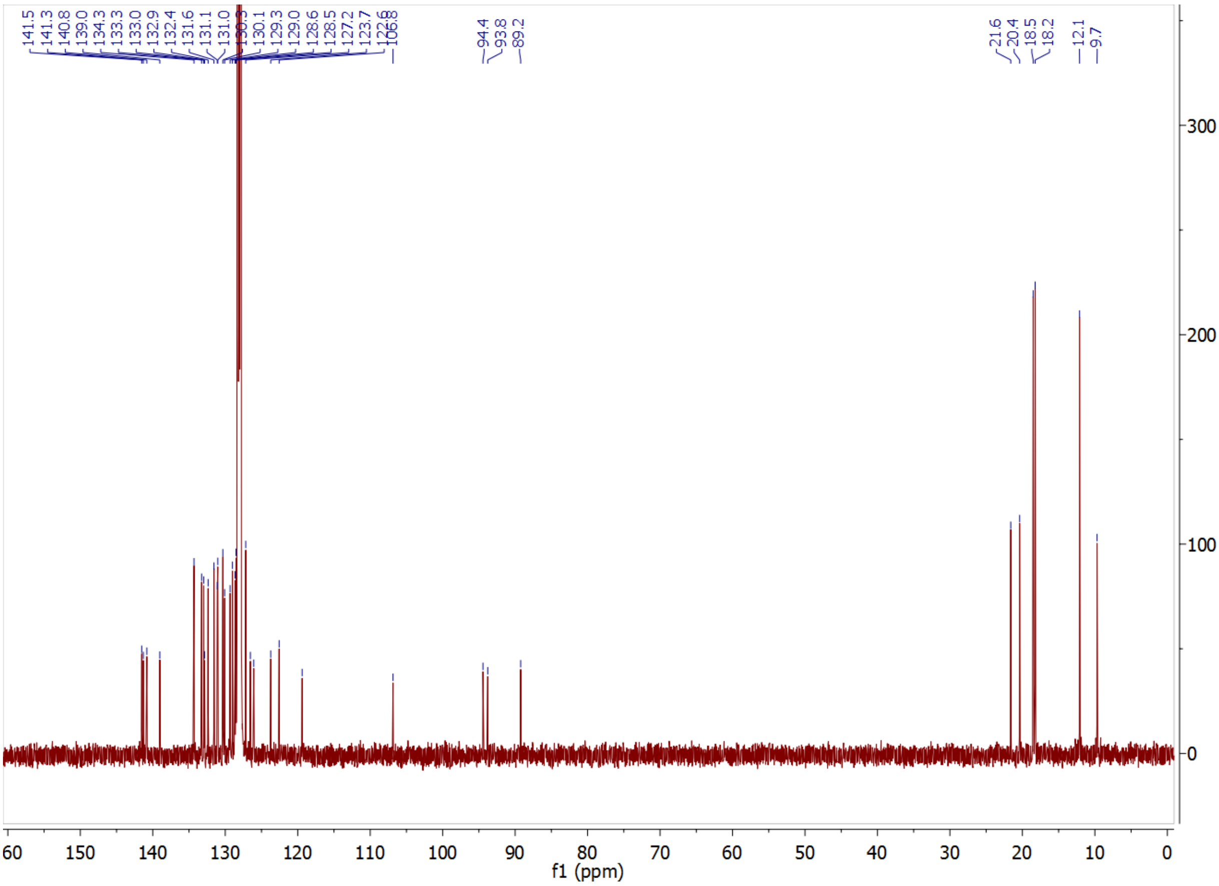

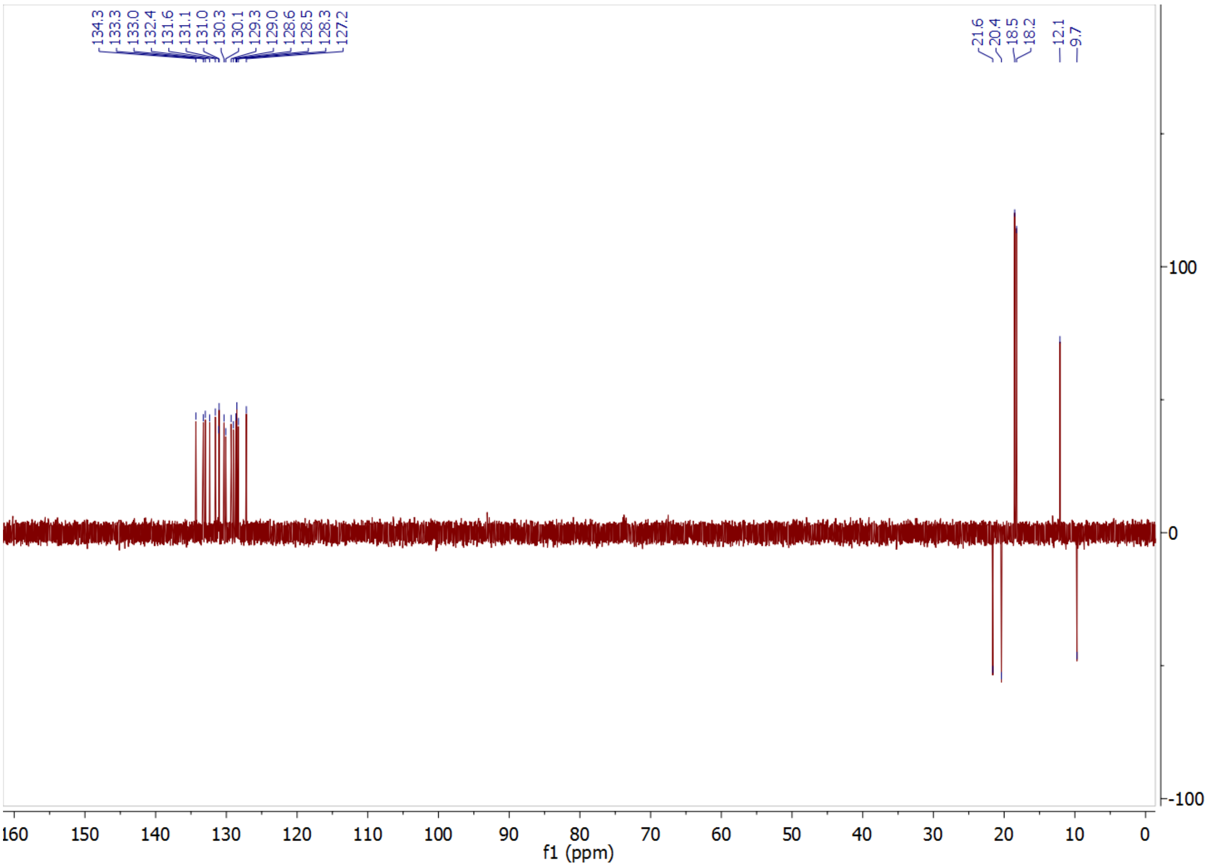

DEPT-135 in benzene-d_6

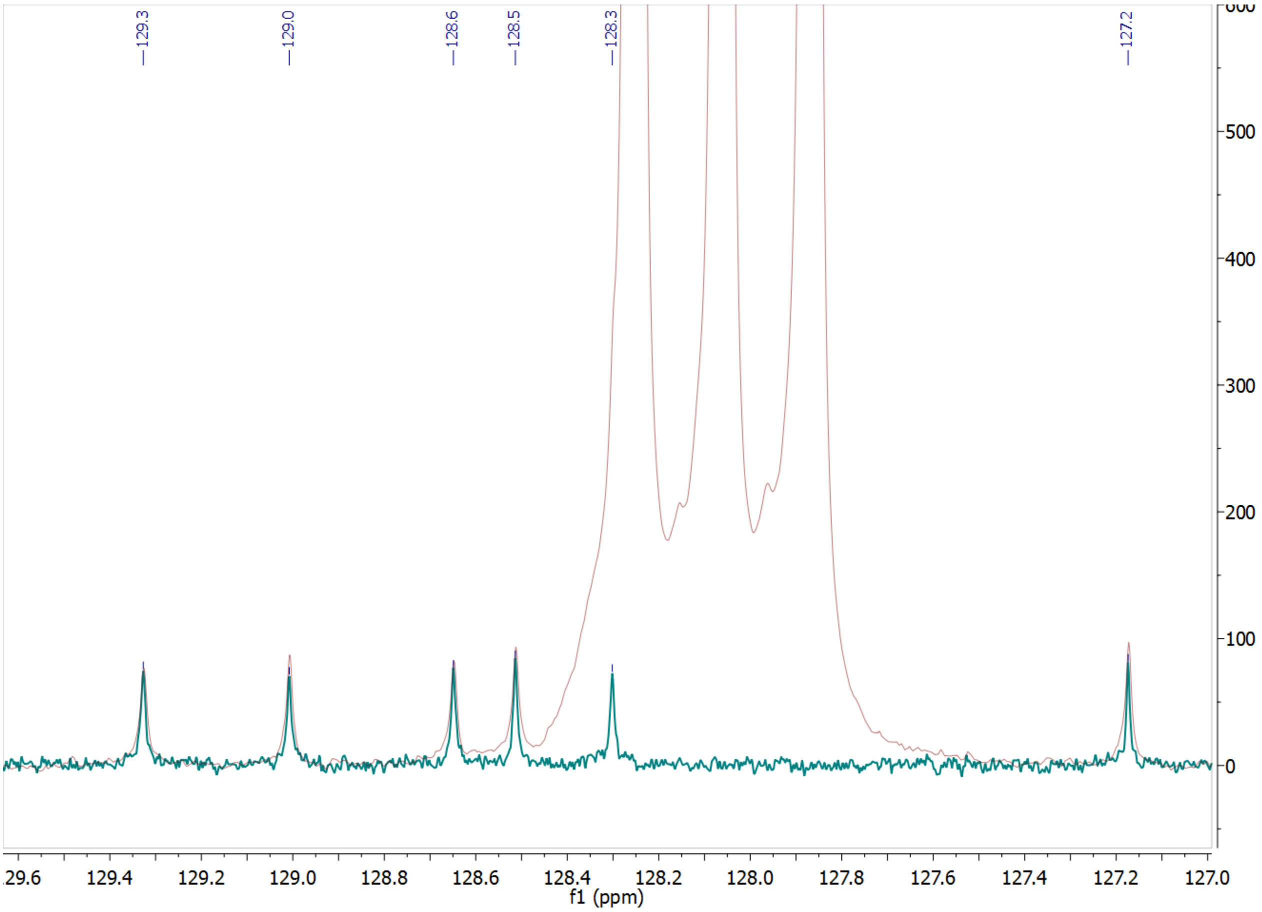

^{13}C-NMR (in red) is superimposed by DEPT-135 (in turquois). The peak of 128.3 is only visible in the DEPT-135 experiment, as the signal is overlain by C_6D_6.

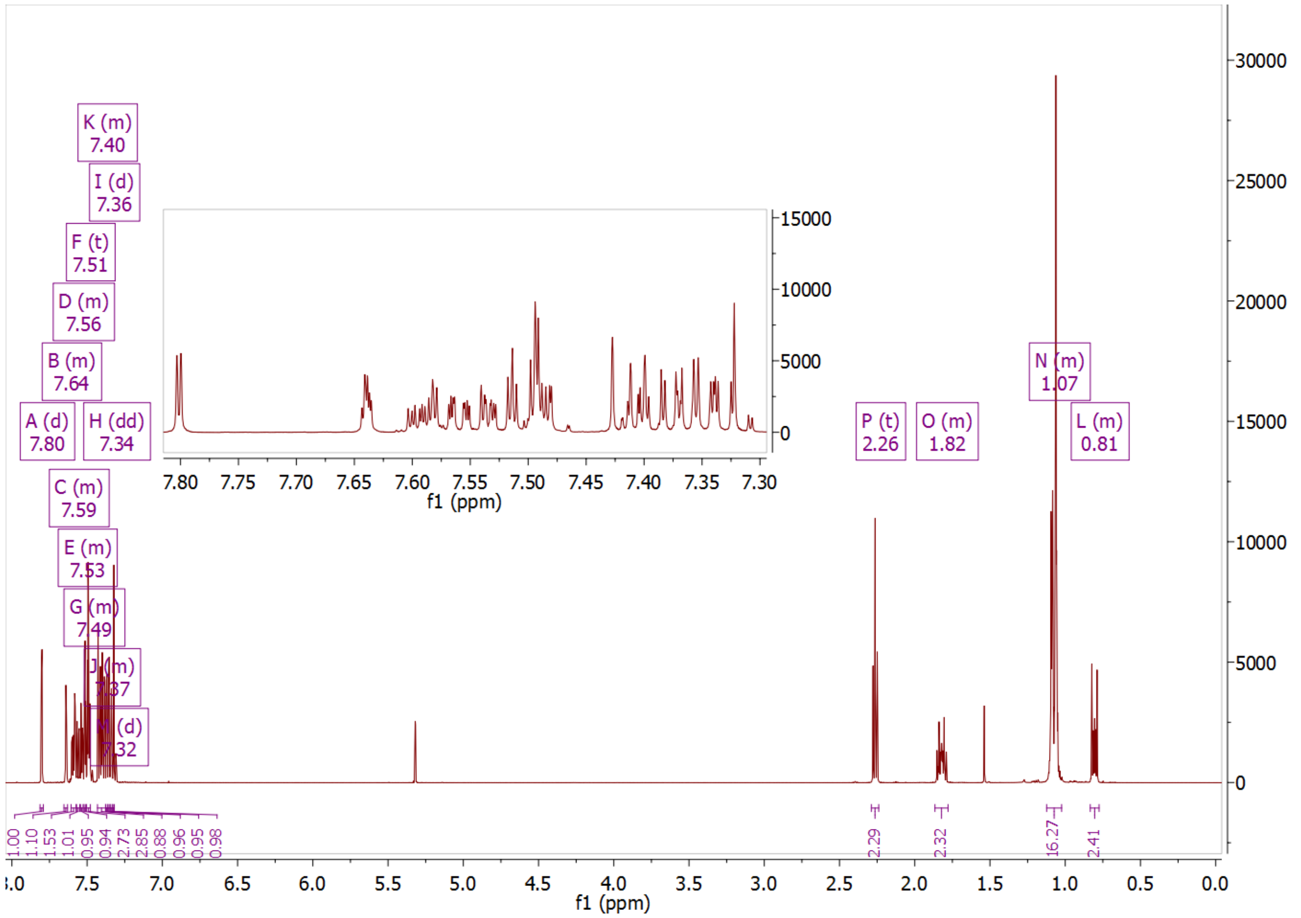

^{1}H in CD_2Cl_2 (above) and ^{13}C in CD_2Cl_2 (below)

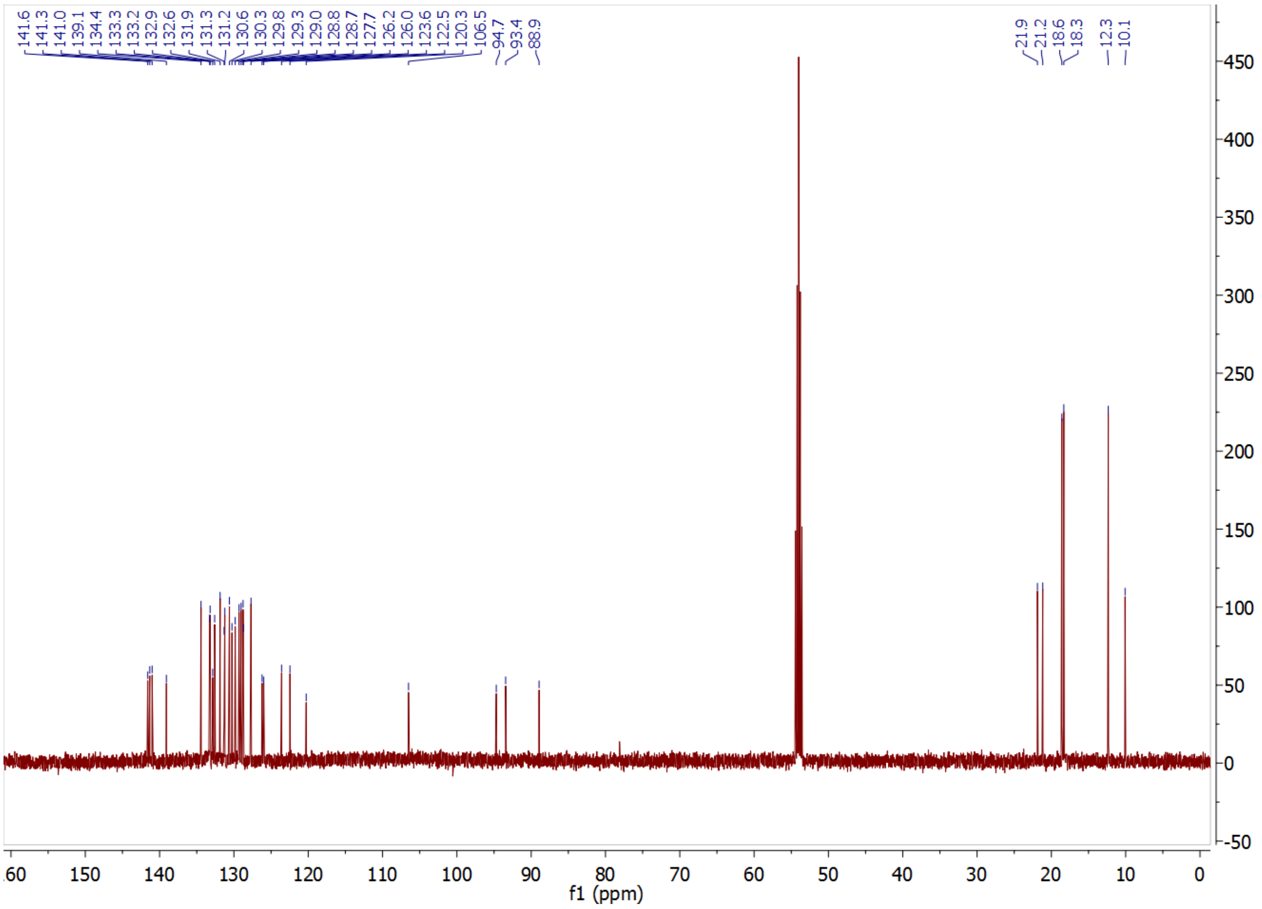

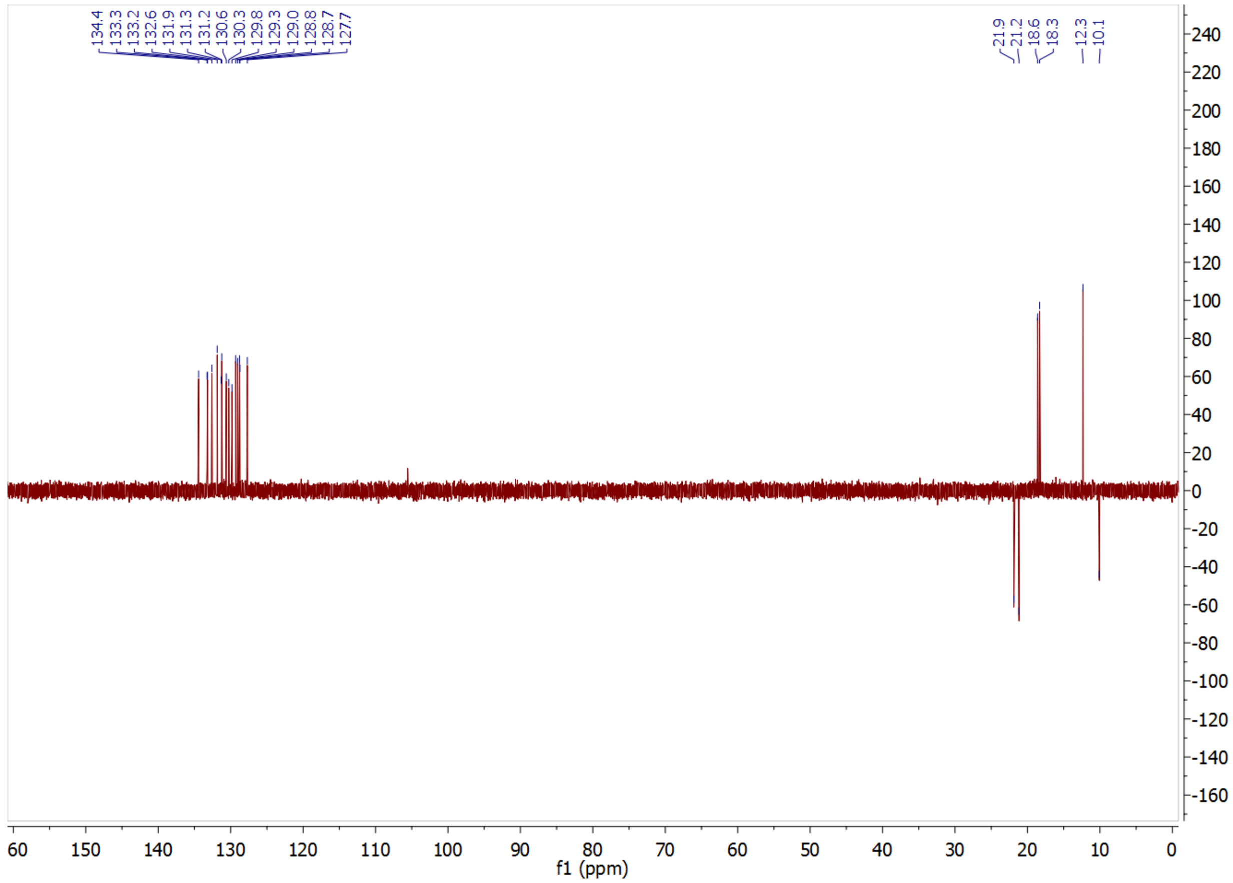

DEPT-135 in CD_2Cl_2

Mass Spectrum SmartFormula Report

Analysis Info

Analysis Name	N:\new acq data\Ba220 chr2-11 001.d	Acquisition Date	09.05.2016 14:41:18
Method	hn Direct_Infusion_pos mode_75-1700 mid 4eV.m	Operator	hn
Sample Name	Linda Bannwart	Instrument / Ser#	maXis 4G 21243
Comment	Ba220 chr 2-11, ca. 11 ug/ml MeCN mit ACN/TFA		

Acquisition Parameter

Source Type	ESI	Ion Polarity	Positive	Set Nebulizer	0.4 Bar
Focus	Not active	Set Capillary	3600 V	Set Dry Heater	180 °C
Scan Begin	75 m/z	Set End Plate Offset	-500 V	Set Dry Gas	4.0 l/min
Scan End	1700 m/z	Set Collision Cell RF	350.0 Vpp	Set Ion Energy (MS only)	4.0 eV

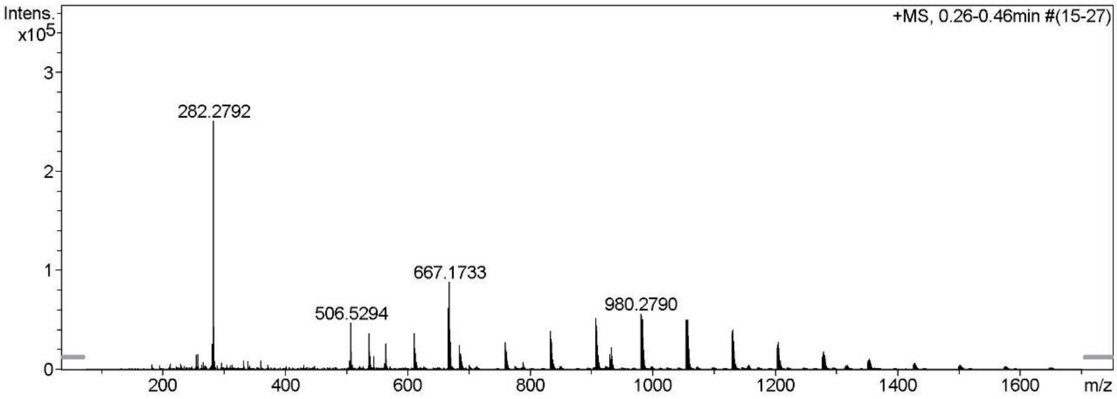

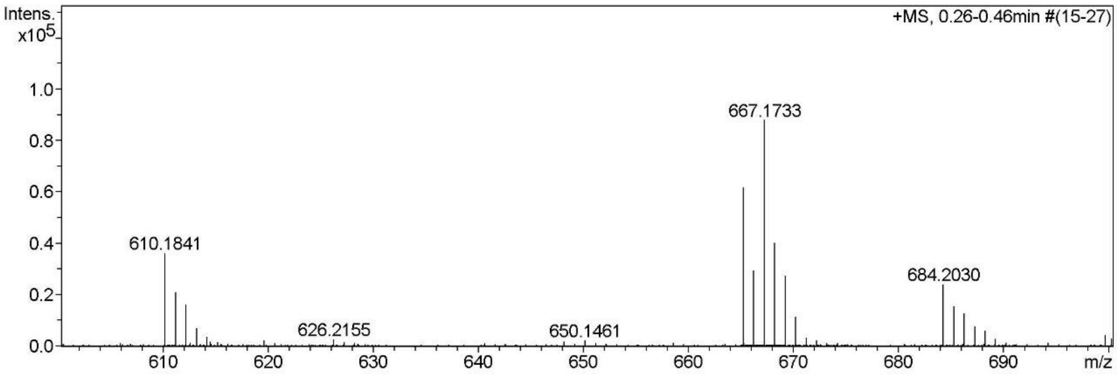

Meas. m/z	#	Formula	Score	m/z	err [mDa]	err [ppm]	mSigma	rdb	e⁻ Conf	N-Rule	z
648.1478	1	C 38 H 36 Br Cl N Si	100.00	648.1483	0.6	0.9	28.4	21.5	even	ok	1+
665.1751	1	C 38 H 39 Br Cl N 2 Si	100.00	665.1749	-0.2	-0.4	13.7	20.5	even	ok	

Mass Spectrum List Report

Analysis Info

Analysis Name	N:\new acq data\Ba220 chr2-11 001.d	Acquisition Date	09.05.2016 14:41:18
Method	hn Direct_Infusion_pos mode_75-1700 mid 4eV.m	Operator	hn
Sample Name	Linda Bannwart	Instrument / Ser#	maXis 4G 21243
Comment	Ba220 chr 2-11, ca. 11 ug/ml MeCN mit ACN/TFA		

Acquisition Parameter

Source Type	ESI	Ion Polarity	Positive	Set Nebulizer	0.4 Bar
Focus	Not active	Set Capillary	3600 V	Set Dry Heater	180 °C
Scan Begin	75 m/z	Set End Plate Offset	-500 V	Set Dry Gas	4.0 l/min
Scan End	1700 m/z	Set Collision Cell RF	350.0 Vpp	Set Ion Energy (MS only)	4.0 eV

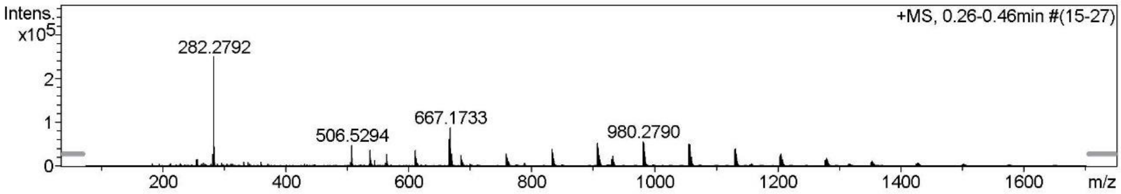

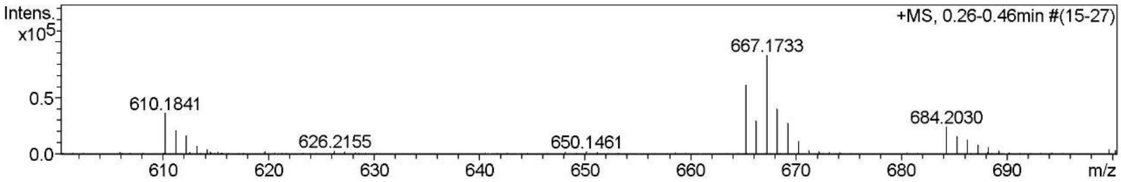

#	m/z	I %	I
1	182.1358	1.8	4525
2	211.9840	2.1	5150
3	228.2319	1.9	4703
4	254.2476	5.6	14041
5	256.2631	6.0	14895
6	265.2523	2.6	6459
7	280.2631	10.1	25230
8	281.2664	2.0	4953
9	282.2792	100.0	250007
10	283.2822	17.1	42692
11	284.2921	3.0	7611
12	295.1900	2.4	6047
13	331.2839	3.3	8223
14	338.3413	2.9	7361
15	359.3150	3.3	8172
16	504.5133	3.2	7982
17	506.5294	18.6	46495
18	507.5325	6.9	17233
19	536.1653	14.4	36125
20	537.1658	6.9	17184
21	538.1631	4.9	12331
22	543.9430	5.0	12578
23	561.5350	2.2	5564
24	563.5507	10.2	25430
25	564.5542	4.0	9984
26	610.1841	14.4	35919
27	611.1847	8.2	20563
28	612.1809	6.3	15853
29	613.1797	2.7	6684
30	665.1751	24.5	61365
31	666.1777	11.7	29164
32	667.1733	35.0	87578
33	668.1757	15.9	39824
34	669.1719	10.9	27126
35	670.1734	4.5	11195
36	684.2030	9.5	23802
37	685.2035	6.1	15168

Mass Spectrum List Report

#	m/z	I %	I
38	686.1946	5.0	12470
39	687.1882	3.0	7416
40	688.1833	2.2	5605
41	758.2220	10.7	26826
42	759.2226	7.8	19500
43	760.2153	7.0	17378
44	761.2098	4.0	10017
45	762.2034	3.1	7746
46	787.8009	2.7	6632
47	832.2410	15.3	38216
48	833.2415	11.9	29856
49	834.2367	10.7	26812
50	835.2340	6.3	15818
51	836.2265	3.8	9433
52	837.2237	2.0	4999
53	906.2599	20.5	51189
54	907.2605	17.4	43582
55	908.2570	15.5	38866
56	909.2555	9.6	23970
57	910.2511	5.5	13758
58	911.2479	2.7	6782
59	929.4188	5.9	14720
60	930.4223	3.9	9793
61	931.4188	8.7	21745
62	932.4213	5.2	13052
63	933.4199	3.6	9011
64	980.2790	22.2	55422
65	981.2797	21.3	53278
66	982.2769	20.1	50297
67	983.2755	12.6	31461
68	984.2726	7.6	19067
69	985.2701	3.6	9076
70	1054.2980	19.7	49242
71	1055.2987	19.7	49257
72	1056.2962	19.9	49687
73	1057.2948	13.2	32971
74	1058.2920	8.0	20125
75	1059.2897	4.2	10405
76	1060.2869	2.0	5053
77	1128.3170	14.1	35254
78	1129.3176	15.2	38025
79	1130.3151	15.8	39590
80	1131.3137	11.1	27630
81	1132.3110	7.1	17859
82	1133.3085	4.0	9985
83	1134.3054	2.0	4999
84	1202.3361	8.4	21020
85	1203.3370	9.8	24455
86	1204.3340	10.9	27173
87	1205.3329	8.3	20808
88	1206.3294	5.5	13794
89	1207.3273	3.2	8107
90	1276.3556	4.8	12031
91	1277.3558	5.9	14737
92	1278.3528	6.9	17304
93	1279.3510	5.4	13604
94	1280.3483	4.1	10303
95	1281.3466	2.5	6369
96	1350.3737	2.6	6548
97	1351.3753	3.4	8475
98	1352.3720	4.2	10566
99	1353.3703	3.5	8833
100	1354.3670	2.8	7006
101	1355.3647	1.9	4773
102	1425.3952	2.0	4911
103	1426.3914	2.5	6212
104	1427.3891	2.2	5597
105	1428.3851	1.8	4551

5.14 Compound **18**

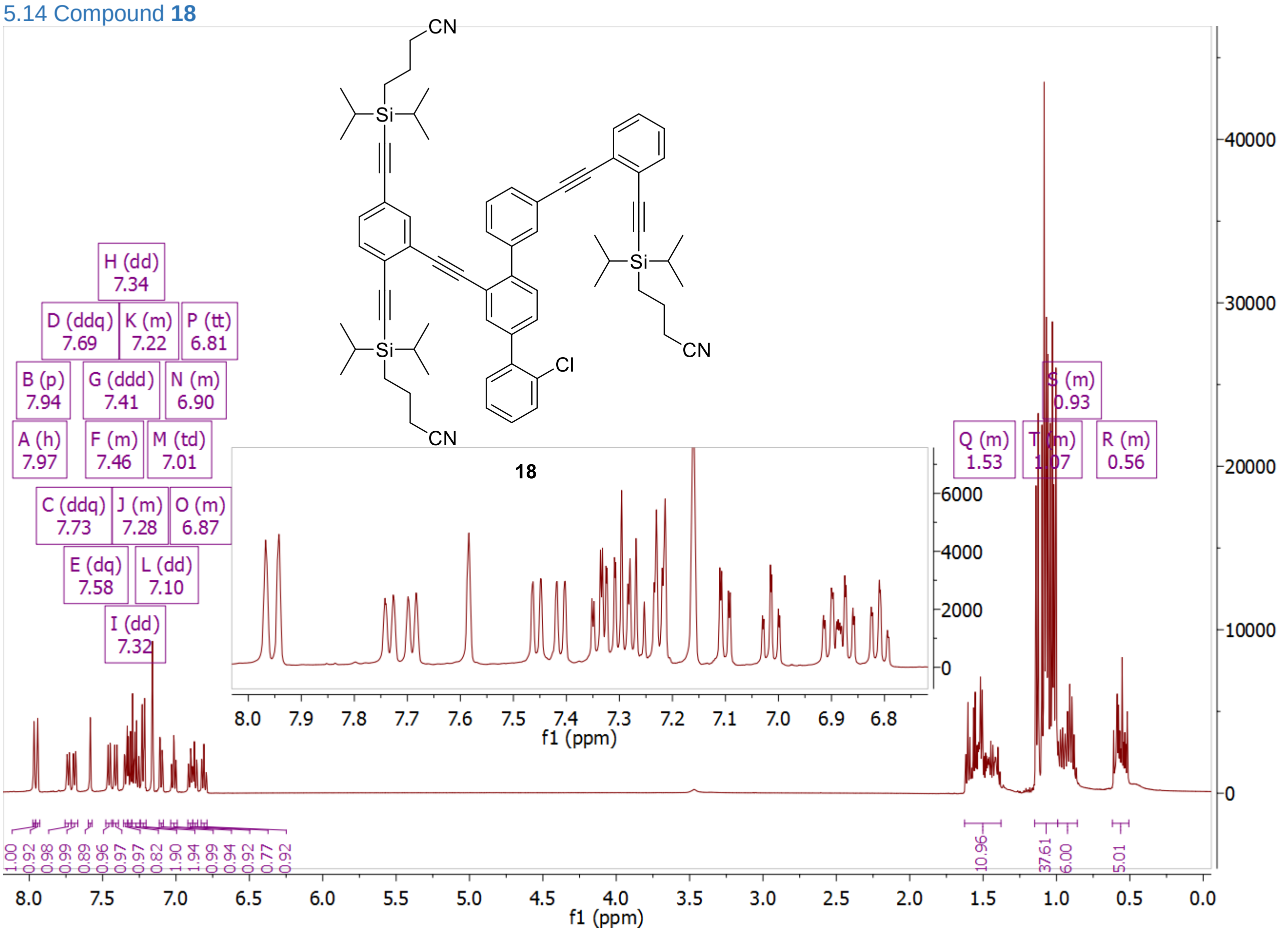

^{1}H in benzene-d_6 (above) and ^{13}C in benzene-d_6 (below)

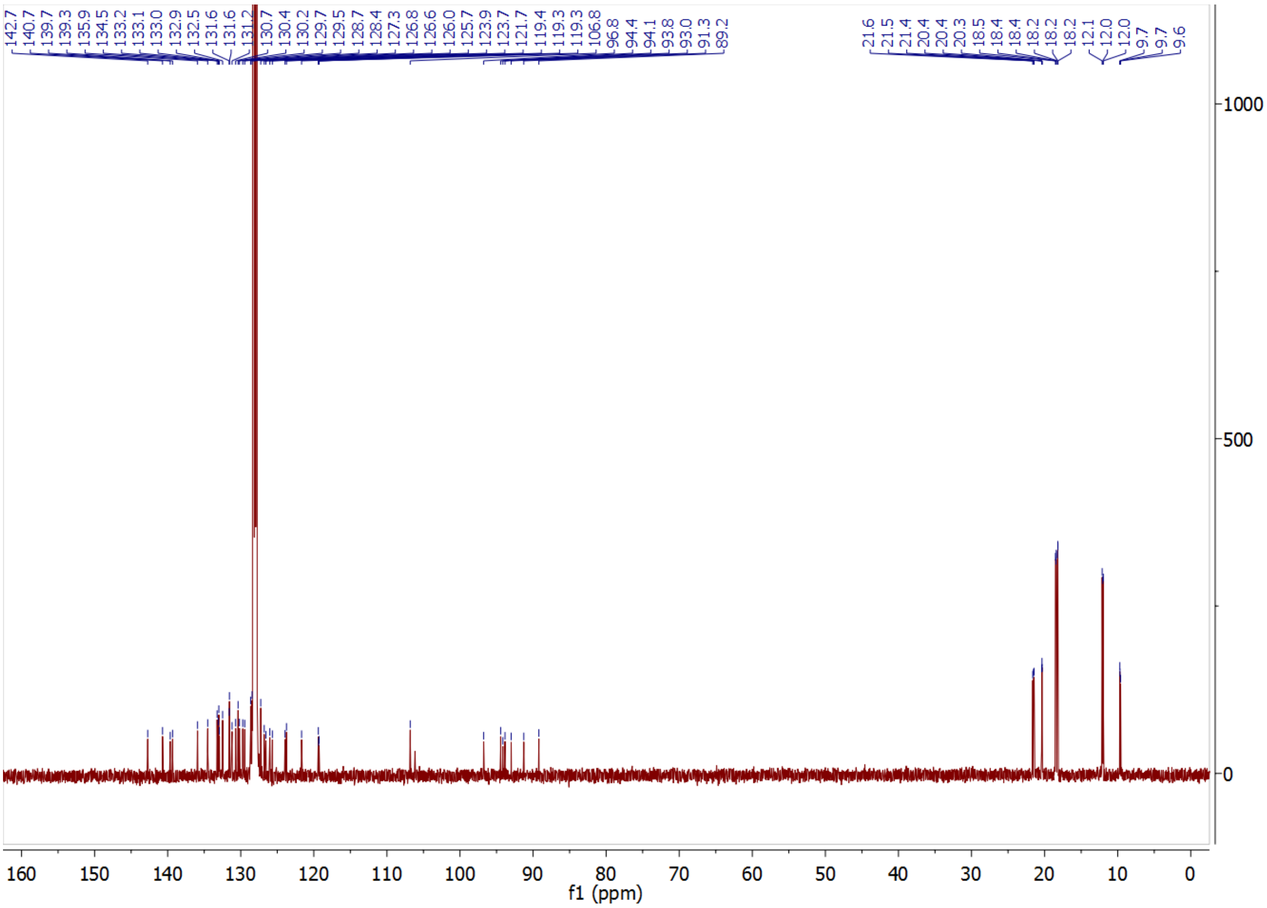

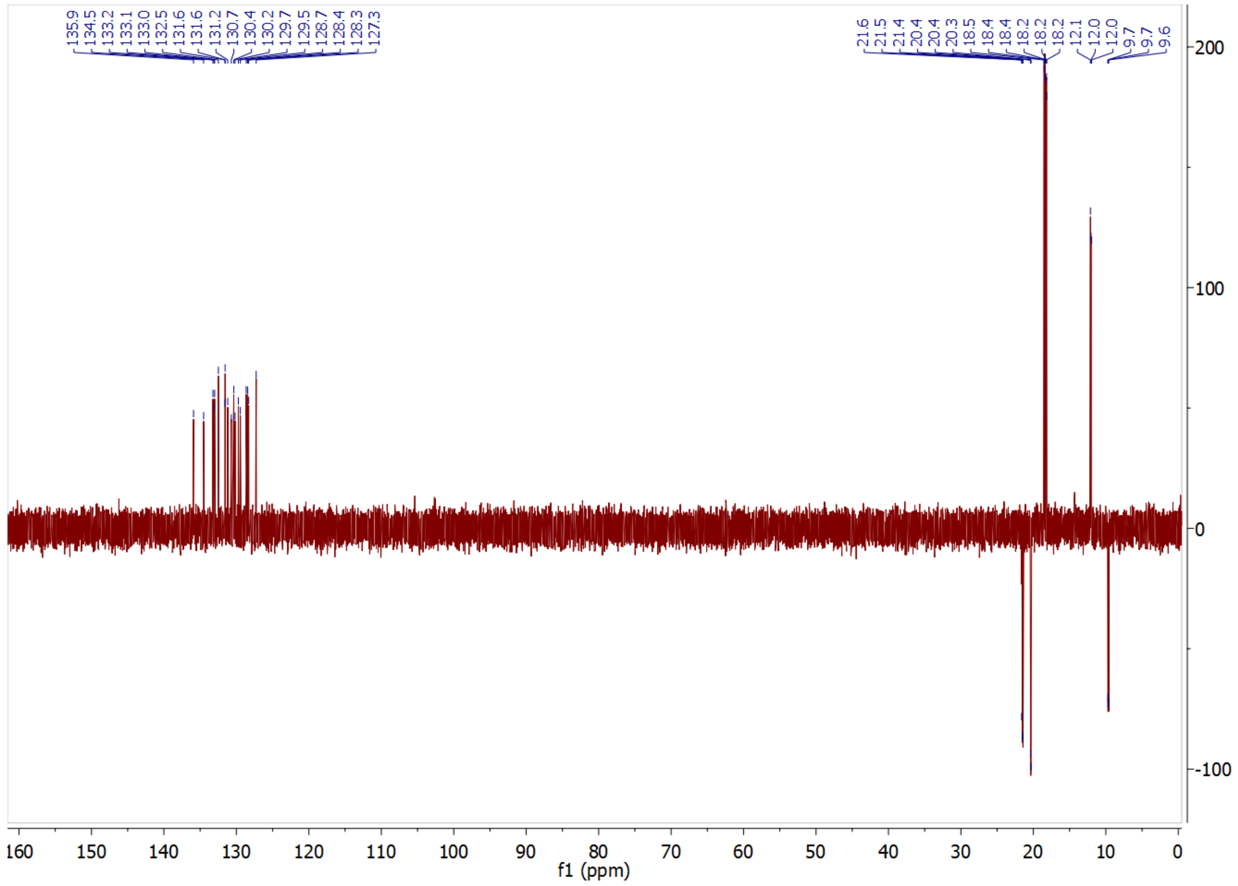

DEPT-135 in benzene-d_6

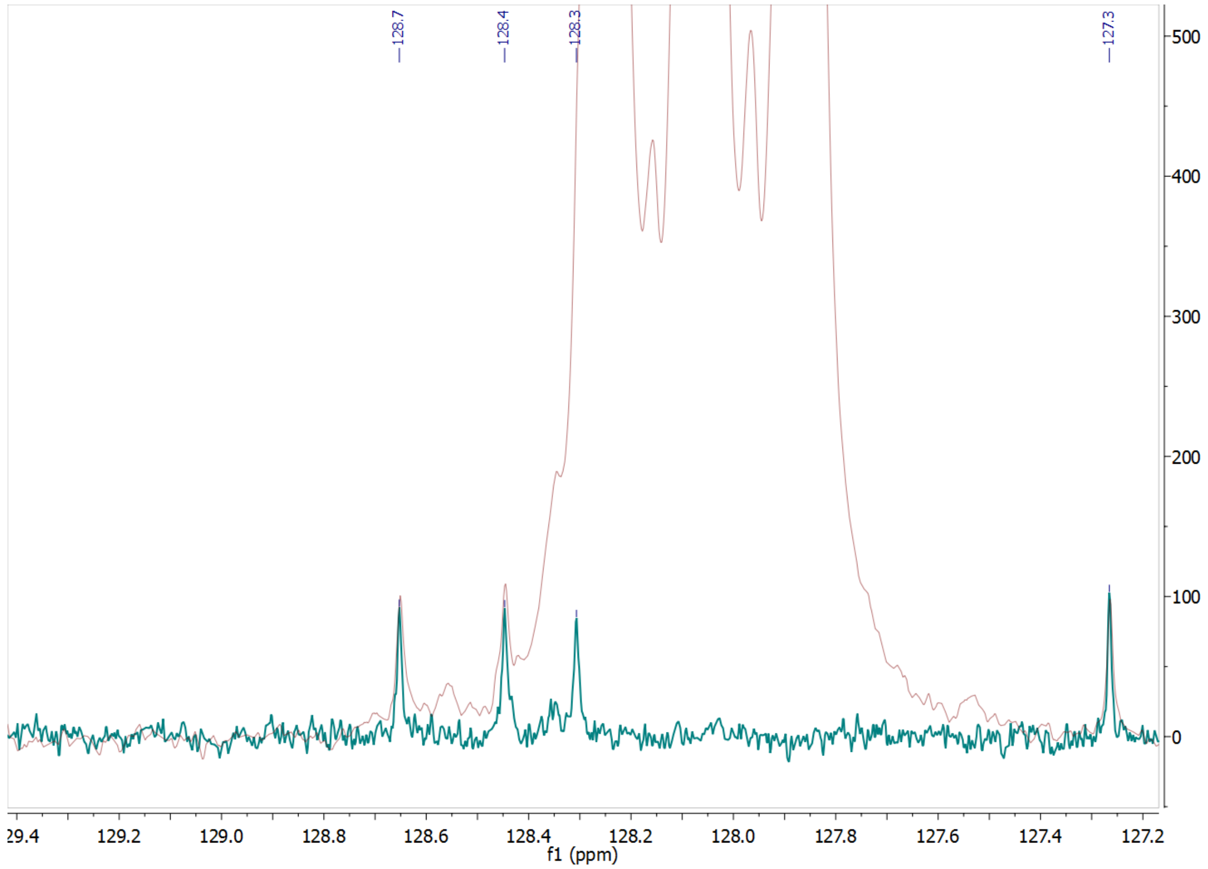

^{13}C-NMR (in red) is superimposed by DEPT-135 (in turquois). The peak of 128.3 is only visible in the DEPT-135 experiment, as the signal is overlain by C_6D_6.

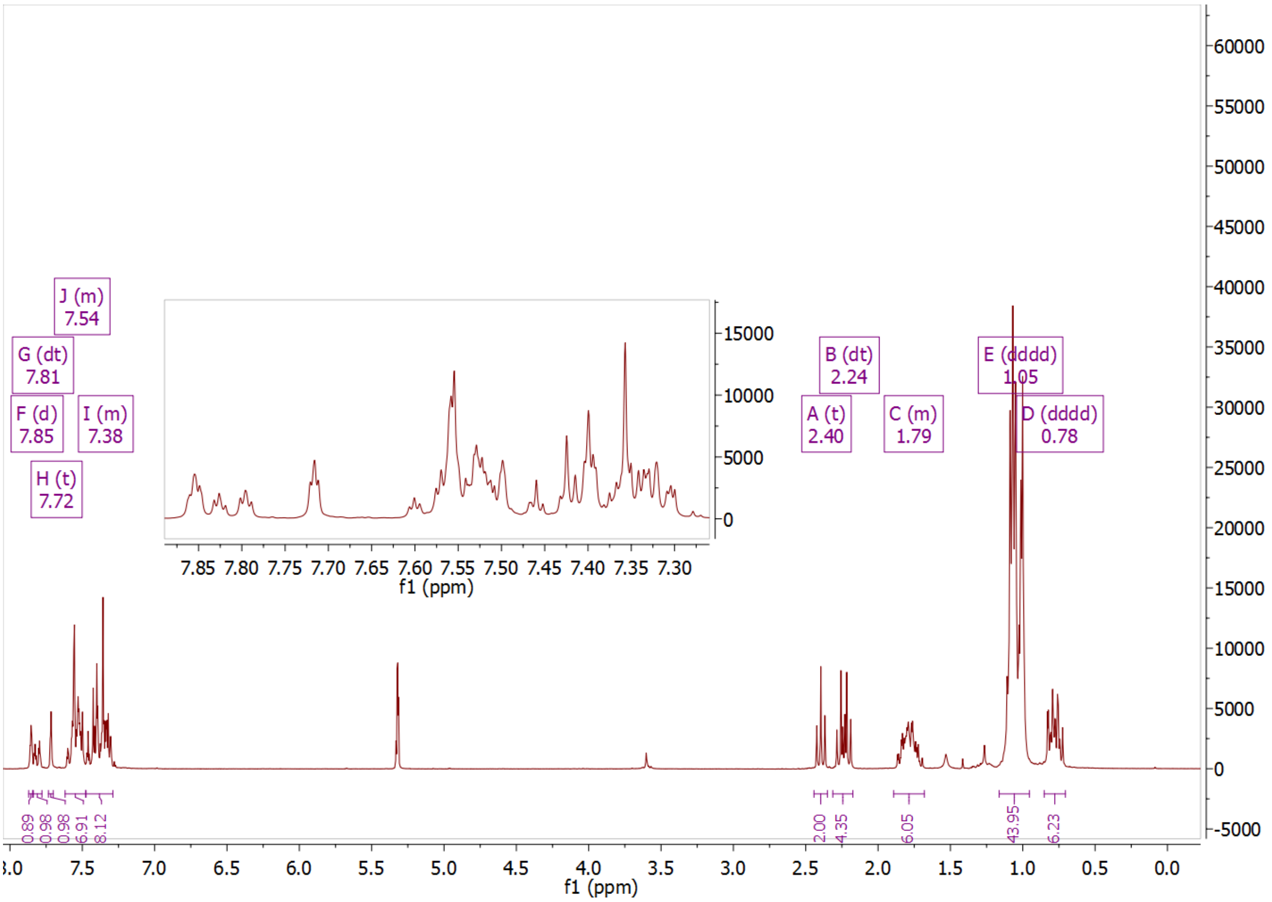

In CD_2Cl_2

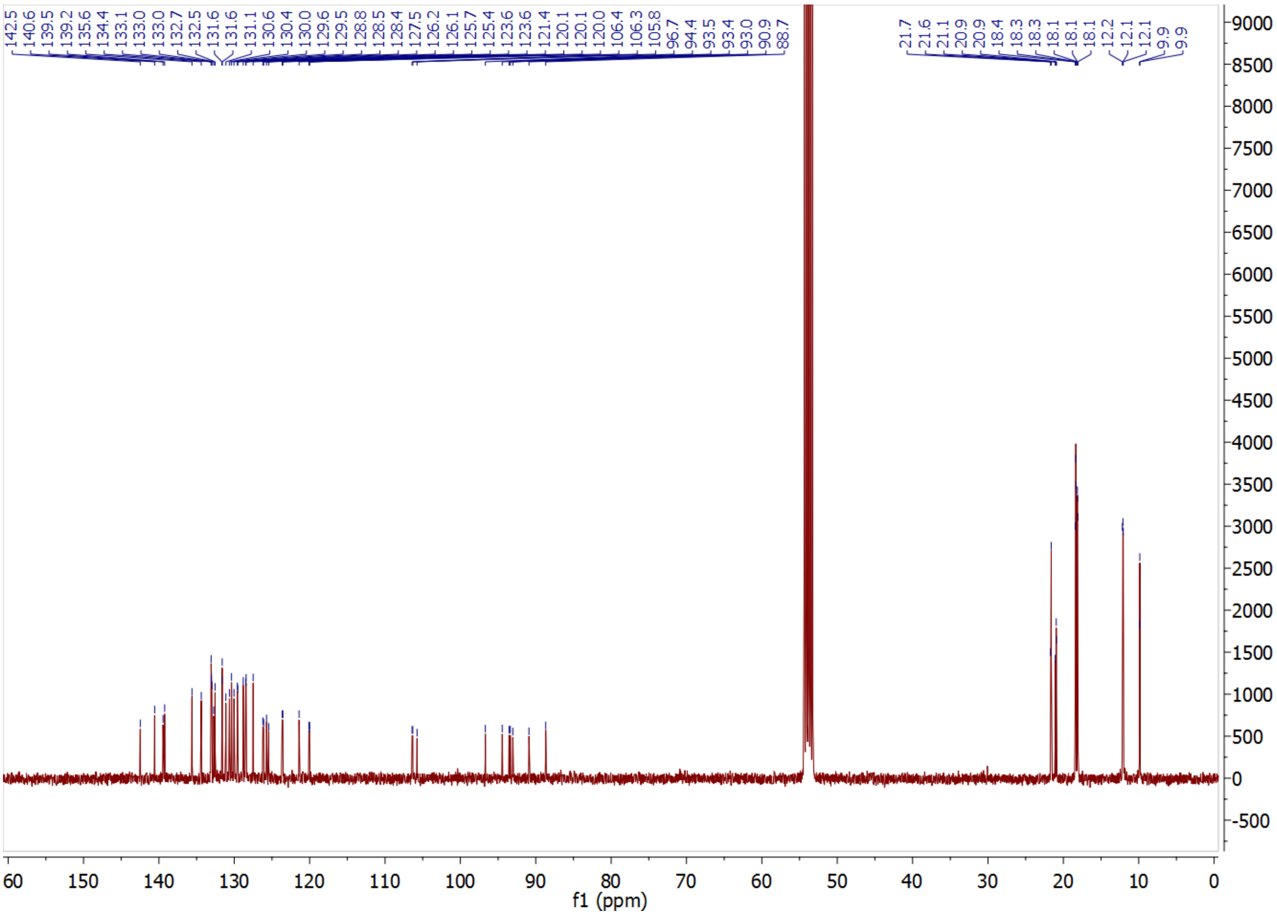

In CD_2Cl_2

Mass Spectrum SmartFormula Report

Analysis Info

Analysis Name	N:\new acq data\Ba223 chr 2-4 001.d	Acquisition Date	09.05.2016 15:06:35
Method	hn Direct_Infusion_pos mode_75-1700 mid 4eV.m	Operator	hn
Sample Name	Linda Bannwart	Instrument / Ser#	maXis 4G 21243
Comment	Ba223 chr 2-4, ca. 10 ug/ml MeCN mit ACN/TFA		

Acquisition Parameter

Source Type	ESI	Ion Polarity	Positive	Set Nebulizer	0.4 Bar
Focus	Not active	Set Capillary	3600 V	Set Dry Heater	180 °C
Scan Begin	75 m/z	Set End Plate Offset	-500 V	Set Dry Gas	4.0 l/min
Scan End	1700 m/z	Set Collision Cell RF	350.0 Vpp	Set Ion Energy (MS only)	4.0 eV

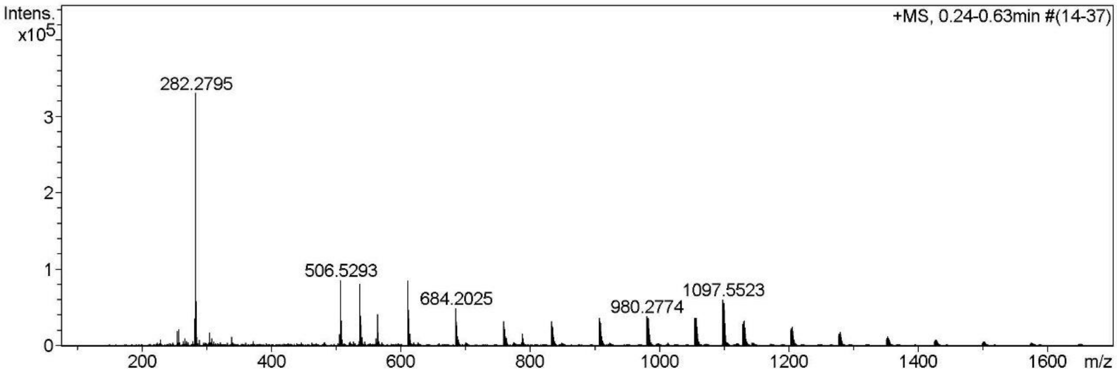

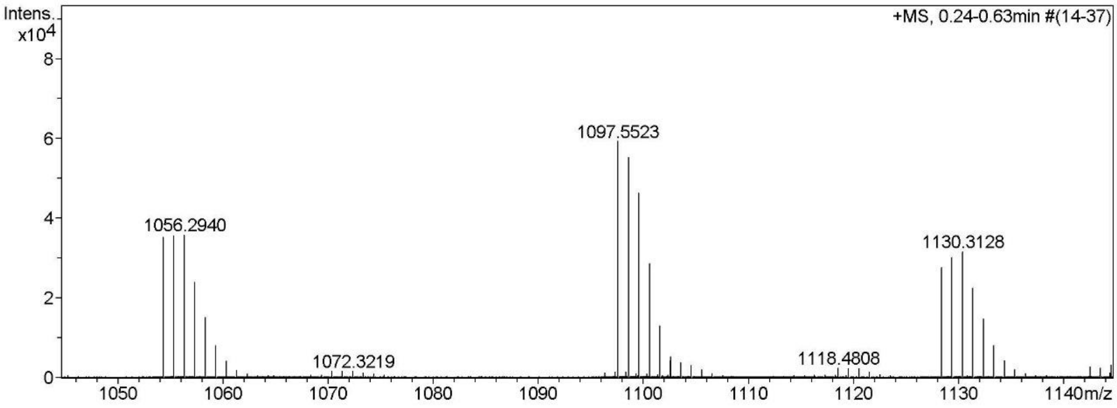

Meas. m/z	#	Formula	Score	m/z	err [mDa]	err [ppm]	mSigma	rdb	e¯ Conf	N-Rule	z
1097.5523	1	C 70 H 82 Cl N 4 Si 3	100.00	1097.5530	0.7	0.7	30.1	34.5	even	ok	1+

Mass Spectrum List Report

Analysis Info

Analysis Name	N:\new acq data\Ba223 chr 2-4 001.d	Acquisition Date	09.05.2016 15:06:35
Method	hn Direct_Infusion_pos mode_75-1700 mid 4eV.m	Operator	hn
Sample Name	Linda Bannwart	Instrument / Ser#	maXis 4G 21243
Comment	Ba223 chr 2-4, ca. 10 ug/ml MeCN mit ACN/TFA		

Acquisition Parameter

Source Type	ESI	Ion Polarity	Positive	Set Nebulizer	0.4 Bar
Focus	Not active	Set Capillary	3600 V	Set Dry Heater	180 °C
Scan Begin	75 m/z	Set End Plate Offset	-500 V	Set Dry Gas	4.0 l/min
Scan End	1700 m/z	Set Collision Cell RF	350.0 Vpp	Set Ion Energy (MS only)	4.0 eV

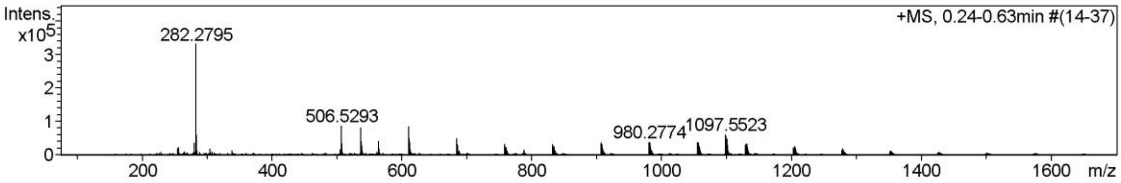

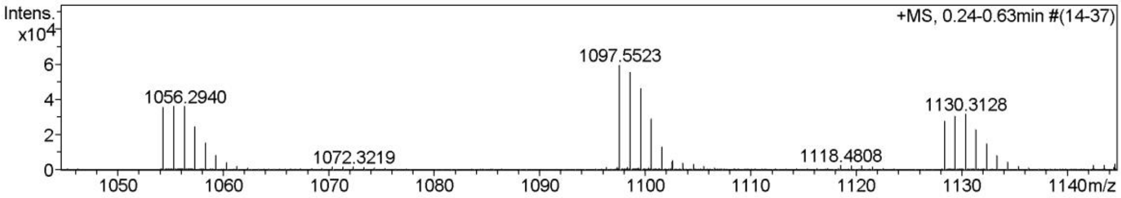

#	m/z	I %	I
1	228.2325	2.0	6722
2	254.2479	5.5	18219
3	256.2635	6.1	20218
4	263.2368	1.5	4954
5	265.2527	2.6	8684
6	280.2635	10.4	34262
7	281.2667	2.1	6763
8	282.2795	100.0	329728
9	283.2826	17.4	57482
10	284.2924	3.2	10507
11	288.2896	2.0	6617
12	304.2609	5.0	16420
13	307.1245	2.7	8756
14	338.3414	3.2	10526
15	371.1008	1.6	5191
16	504.5133	4.3	14215
17	505.5165	1.6	5420
18	506.5293	25.8	84916
19	507.5324	9.5	31474
20	508.5372	2.1	6974
21	535.5191	1.5	5086
22	536.1653	24.2	79817
23	537.1657	11.6	38125
24	537.5342	1.5	4870
25	538.1631	8.0	26286
26	539.1627	2.9	9641
27	561.5345	2.6	8664
28	563.5504	12.2	40344
29	564.5536	4.8	15959
30	565.5610	1.5	5005
31	610.1840	25.4	83797
32	611.1844	13.9	45989
33	612.1814	10.5	34762
34	613.1804	4.4	14536
35	614.1762	1.9	6394
36	684.2025	14.6	47983
37	685.2031	9.1	30130

Mass Spectrum List Report

#	m/z	I %	I
38	686.1981	7.6	25195
39	687.1951	3.5	11400
40	688.1857	2.2	7411
41	758.2211	9.5	31344
42	759.2218	7.0	23069
43	760.2145	6.2	20539
44	761.2093	3.5	11643
45	762.2029	2.7	8858
46	787.8001	4.6	15130
47	788.8036	2.7	8773
48	832.2397	9.5	31311
49	833.2405	7.4	24394
50	834.2346	7.1	23304
51	835.2308	4.1	13528
52	836.2237	3.0	9982
53	837.2218	1.5	4879
54	906.2584	10.8	35460
55	907.2591	9.2	30326
56	908.2549	8.6	28503
57	909.2526	5.3	17475
58	910.2468	3.4	11141
59	911.2435	1.7	5691
60	980.2774	11.4	37692
61	981.2781	10.7	35241
62	982.2748	10.3	33951
63	983.2732	6.5	21538
64	984.2690	4.1	13617
65	985.2658	2.1	6945
66	1054.2961	10.6	35088
67	1055.2967	10.8	35509
68	1056.2940	10.8	35599
69	1057.2927	7.3	23934
70	1058.2894	4.5	14965
71	1059.2867	2.4	7900
72	1097.5523	17.9	59120
73	1098.5549	16.7	55100
74	1099.5534	14.0	46137
75	1100.5535	8.6	28511
76	1101.5541	3.9	12863
77	1102.5527	1.6	5127
78	1128.3147	8.4	27552
79	1129.3156	9.1	30025
80	1130.3128	9.5	31433
81	1131.3115	6.8	22393
82	1132.3088	4.4	14503
83	1133.3062	2.4	7989
84	1202.3336	5.8	19213
85	1203.3342	6.7	22098
86	1204.3318	7.3	24180
87	1205.3303	5.6	18539
88	1206.3275	3.7	12174
89	1207.3252	2.1	7060
90	1276.3522	3.6	11916
91	1277.3529	4.4	14623
92	1278.3501	5.2	17030
93	1279.3488	4.1	13649
94	1280.3463	3.0	9798
95	1281.3439	1.8	5867
96	1350.3708	2.1	6874
97	1351.3721	2.8	9258
98	1352.3691	3.4	11236
99	1353.3674	2.9	9510
100	1354.3642	2.1	7070
101	1355.3623	1.4	4741
102	1425.3907	1.6	5420
103	1426.3877	2.2	7093
104	1427.3863	1.9	6186
105	1428.3833	1.5	4873

5.15 Compound **C₁**

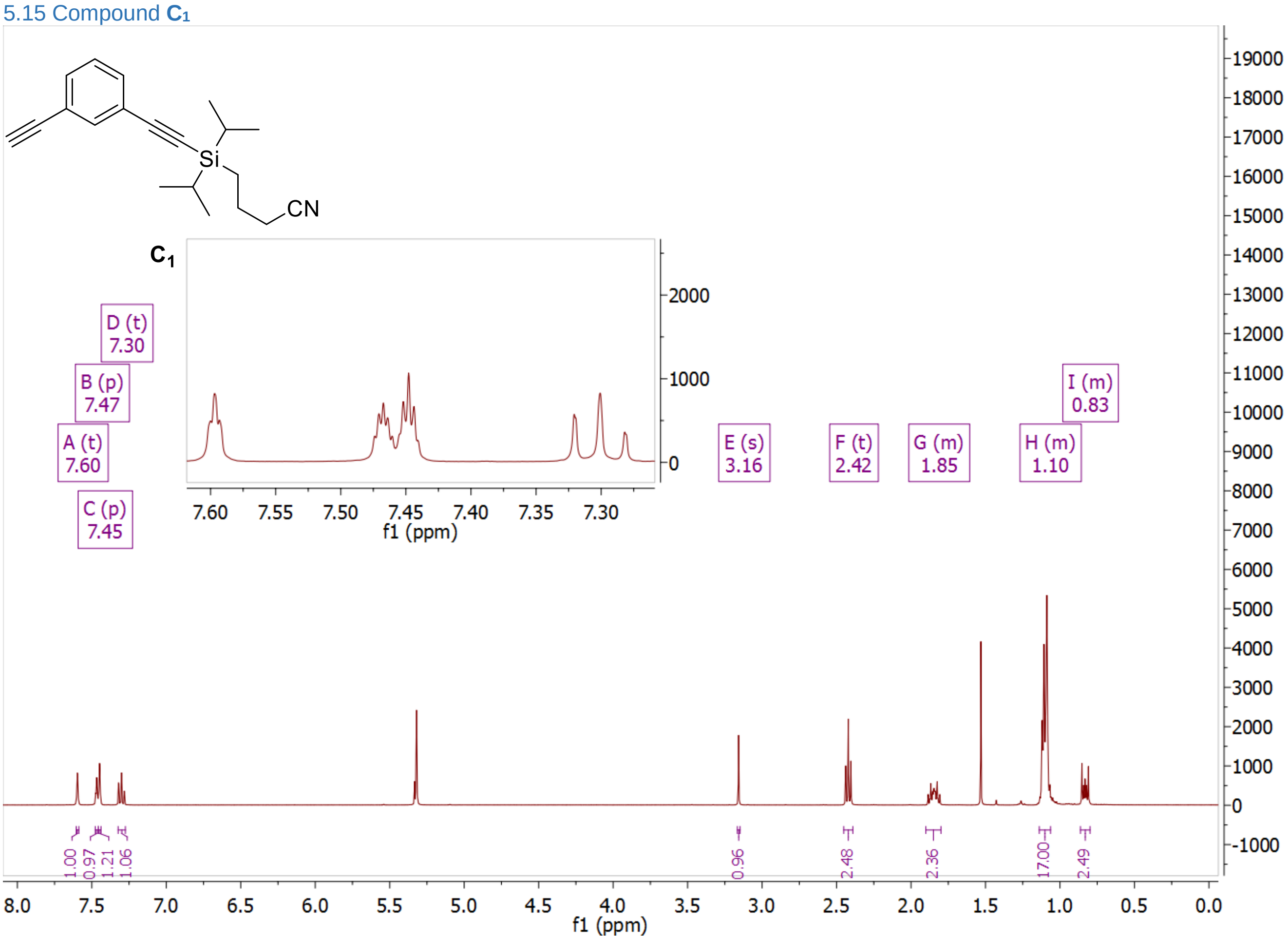

The spectra data of this compound was identical to those reported in the literature.[5]

5.16 Compound **21**

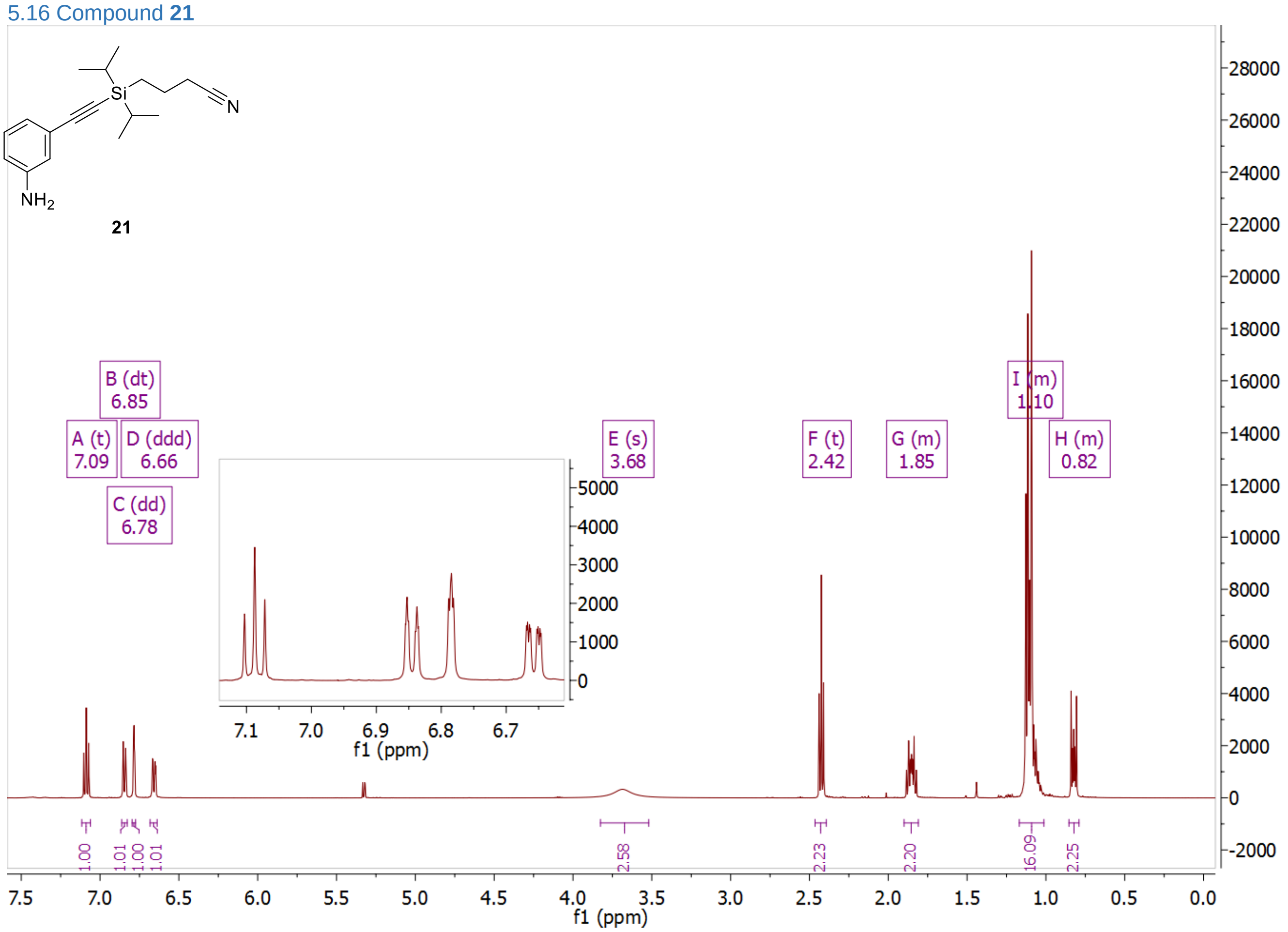

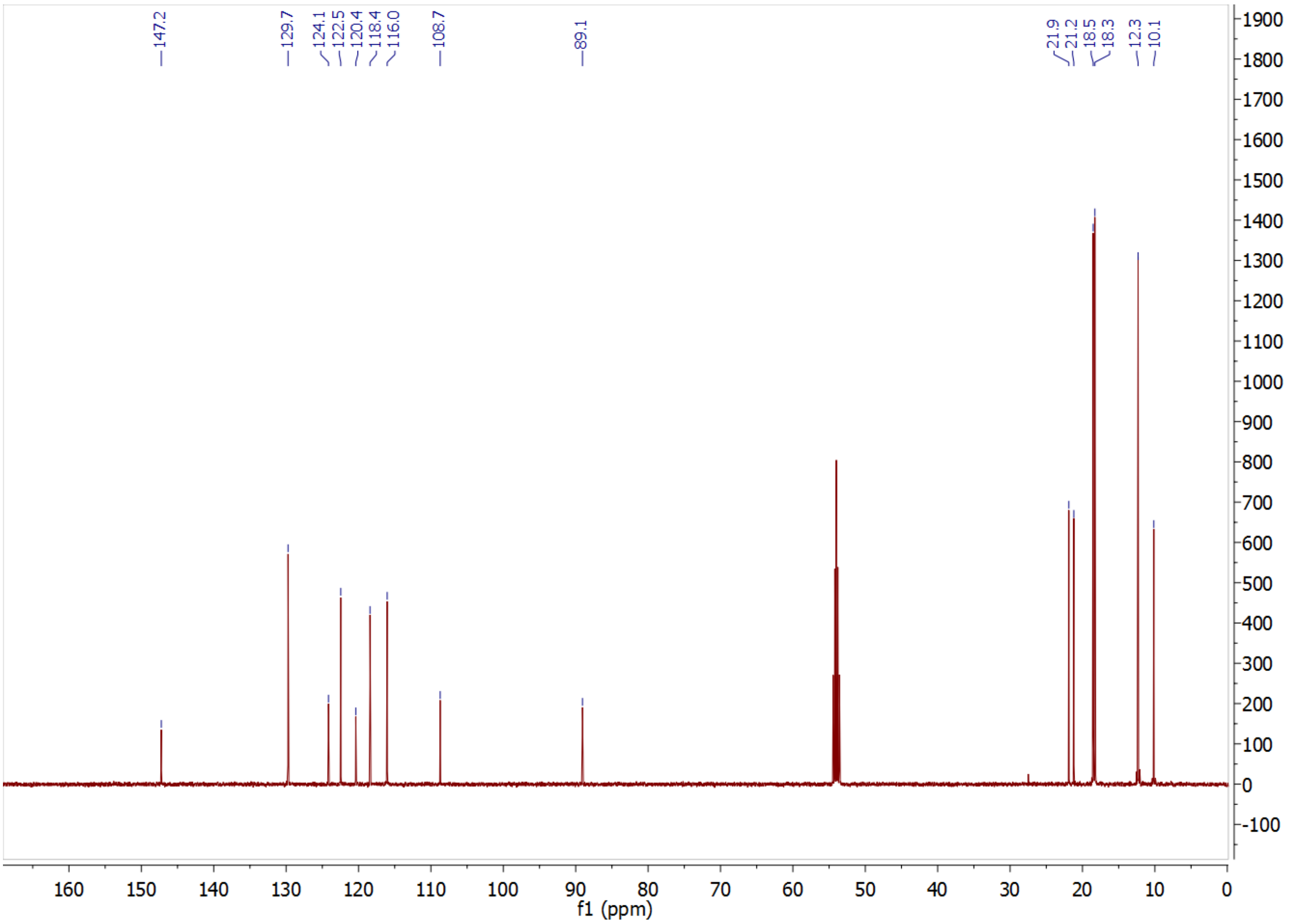

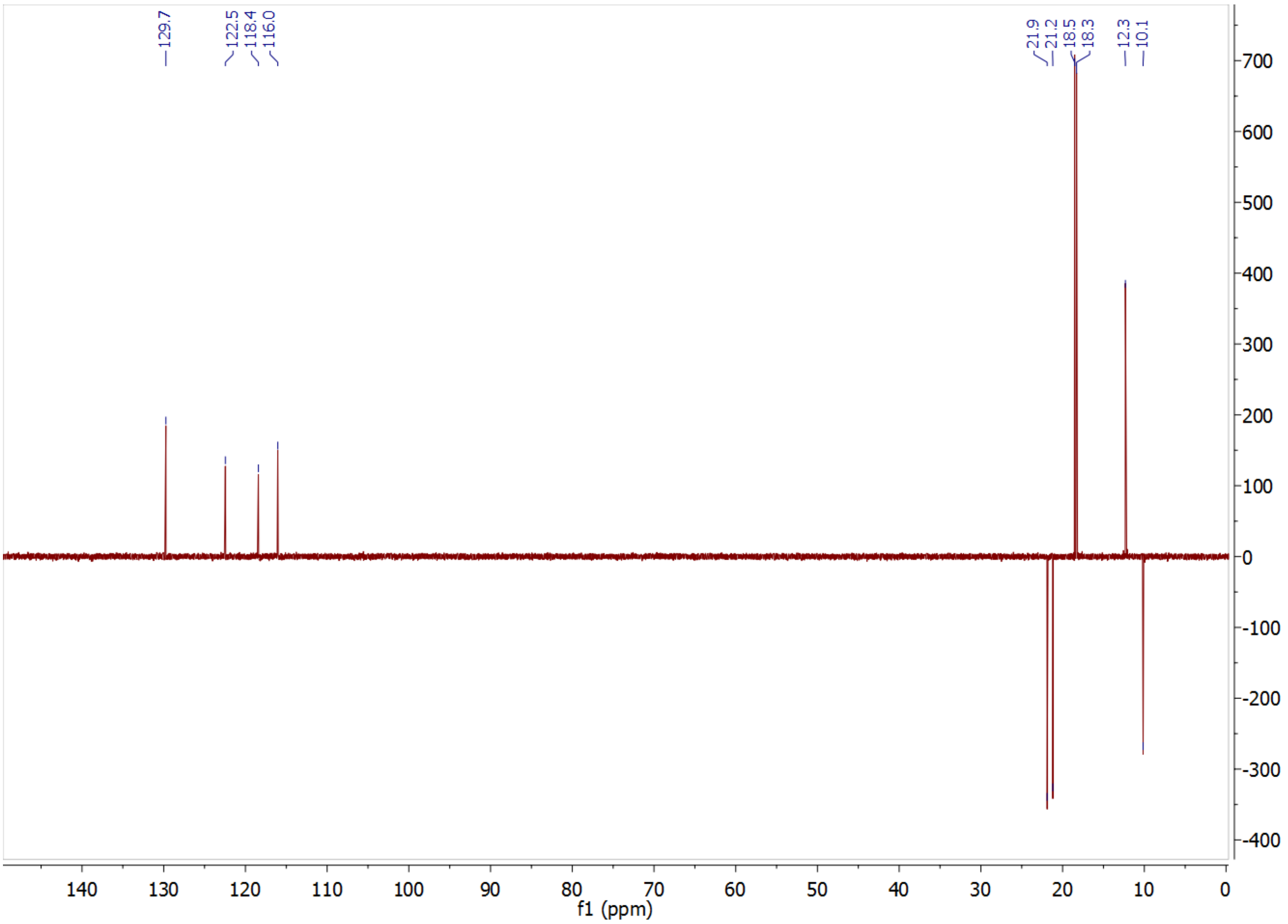

129.7
122.5
118.4
116.0
21.9
21.2
18.5
18.3
12.3
10.1
700
600
500
400
300
200
100
0
-100
-200
-300
-400
140
130
120
110
100
90
80
70
60
50
40
30
20
10
0
f1 (ppm)

High Resolution Mass Spectrometry Report

Sample Name	**Linda Bannwart / Ba567**	Instrument	maXis 4G
Comment	10 ug/mL in MeOH, analyzed in MeOH	Method	22 Direct_pos_mid.m

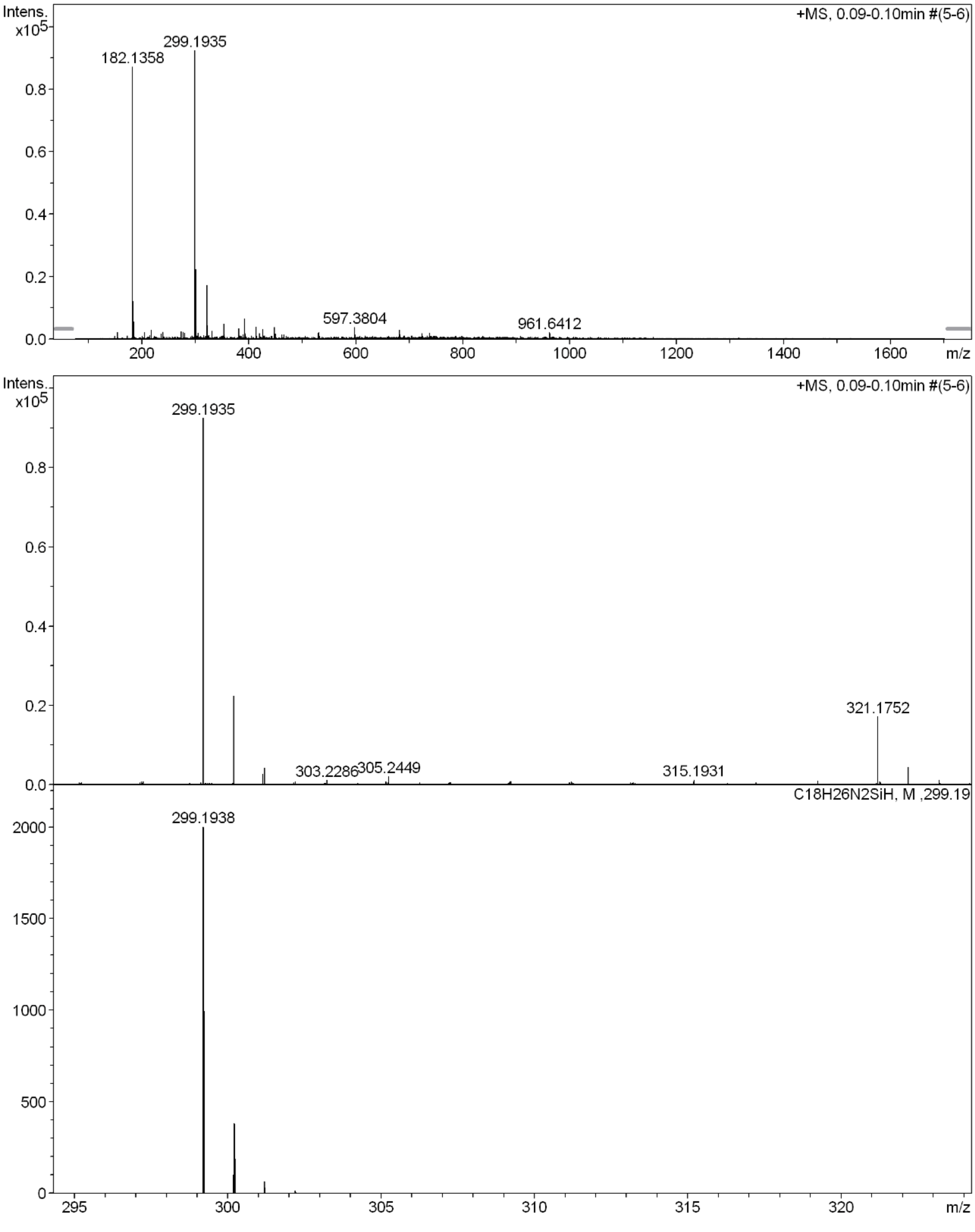

High Resolution Mass Spectrometry Report

Measured m/z vs. theoretical m/z

Meas. m/z	#	Formula	Score	m/z	err [mDa]	err [ppm]	mSigma	rdb	e⁻ Conf	z
299.1935	1	C 18 H 27 N 2 Si	100.00	299.1938	0.3	0.8	10.0	7.5	even	1+

Mass list

#	m/z	I %	I
1	149.0234	1.0	950
2	154.1041	2.2	2037
3	173.0780	1.2	1122
4	182.1358	94.2	87016
5	183.0779	4.2	3880
6	183.1381	13.1	12130
7	184.1329	3.8	3531
8	185.1145	5.9	5495
9	201.1025	0.9	838
10	205.0597	2.3	2163
11	215.1250	1.4	1266
12	217.1046	3.2	2913
13	223.0945	0.9	852
14	225.1097	0.9	817
15	236.0716	1.8	1644
16	239.0883	2.4	2201
17	273.1666	2.6	2382
18	277.2134	2.4	2262
19	279.1586	2.1	1898
20	279.2283	2.0	1825
21	293.2072	1.1	1023
22	299.1935	100.0	92389
23	300.1960	24.2	22374
24	301.1405	2.8	2570
25	301.1939	4.6	4258
26	303.2286	1.2	1101
27	305.2449	2.2	1994
28	309.2033	1.0	882
29	309.2268	0.9	828
30	315.1931	1.3	1192
31	319.2243	1.0	962
32	321.1752	18.6	17159
33	322.1785	4.8	4408
34	323.1771	1.2	1115
35	325.2070	1.2	1140
36	331.2085	2.8	2550
37	348.9897	1.0	889
38	349.0606	0.9	863
39	350.9858	1.2	1075
40	351.2493	0.9	855
41	353.1455	1.4	1303
42	353.2658	5.2	4850
43	354.2695	1.4	1274
44	381.2973	3.6	3318
45	382.3019	1.0	933
46	383.1399	1.2	1092
47	385.2914	0.9	867
48	389.2504	1.6	1466
49	391.2838	7.0	6440
50	392.2870	1.9	1785
51	393.2970	1.4	1277
52	405.1217	1.1	1021
53	413.2663	4.2	3853
54	414.2692	1.0	960
55	419.3145	2.0	1872
56	425.3622	3.5	3200
57	426.3657	1.2	1092
58	429.3178	1.2	1115
59	441.2959	1.0	947
60	443.2064	0.9	852
61	447.2924	1.0	941
62	447.3461	4.0	3725

High Resolution Mass Spectrometry Report

#	m/z	I %	I
63	448.3485	1.2	1063
64	449.3744	1.8	1641
65	461.3205	1.4	1308
66	465.2495	1.4	1303
67	465.3704	0.9	828
68	469.3261	1.0	907
69	505.3353	0.9	867
70	529.1676	2.1	1918
71	531.1650	2.3	2126
72	532.1664	1.0	924
73	597.3804	4.1	3780
74	598.3839	1.4	1300
75	607.4107	0.9	834
76	617.4701	1.1	985
77	631.4793	1.0	905
78	661.4957	1.0	954
79	681.3266	3.0	2792
80	682.3309	1.5	1424
81	689.4706	1.2	1095
82	691.5042	1.1	997
83	703.5254	1.1	985
84	705.5239	1.1	1025
85	723.5503	1.9	1724
86	724.5500	1.0	901
87	733.5462	1.1	979
88	737.3912	2.1	1940
89	738.3909	1.0	968
90	741.4547	1.0	955
91	745.5307	1.1	971
92	747.5486	0.9	846
93	749.5413	0.9	825
94	751.5081	0.9	823
95	786.5366	1.0	926
96	797.5853	0.9	865
97	837.5780	0.9	831
98	907.6691	1.0	902
99	961.6412	2.2	2075
100	962.6442	2.0	1855

Acquisition Parameter

Source Type	ESI	Ion Polarity	Positive	Set Nebulizer	0.4 Bar
Focus	Not active	Set Capillary	3600 V	Set Dry Heater	180 °C
Scan Begin	75 m/z	Set End Plate Offset	-500 V	Set Dry Gas	4.0 l/min
Scan End	1700 m/z	Set Collision Cell RF	350.0 Vpp	Set Ion Energy (MS only)	4.0 eV

5.17 Compound **22**

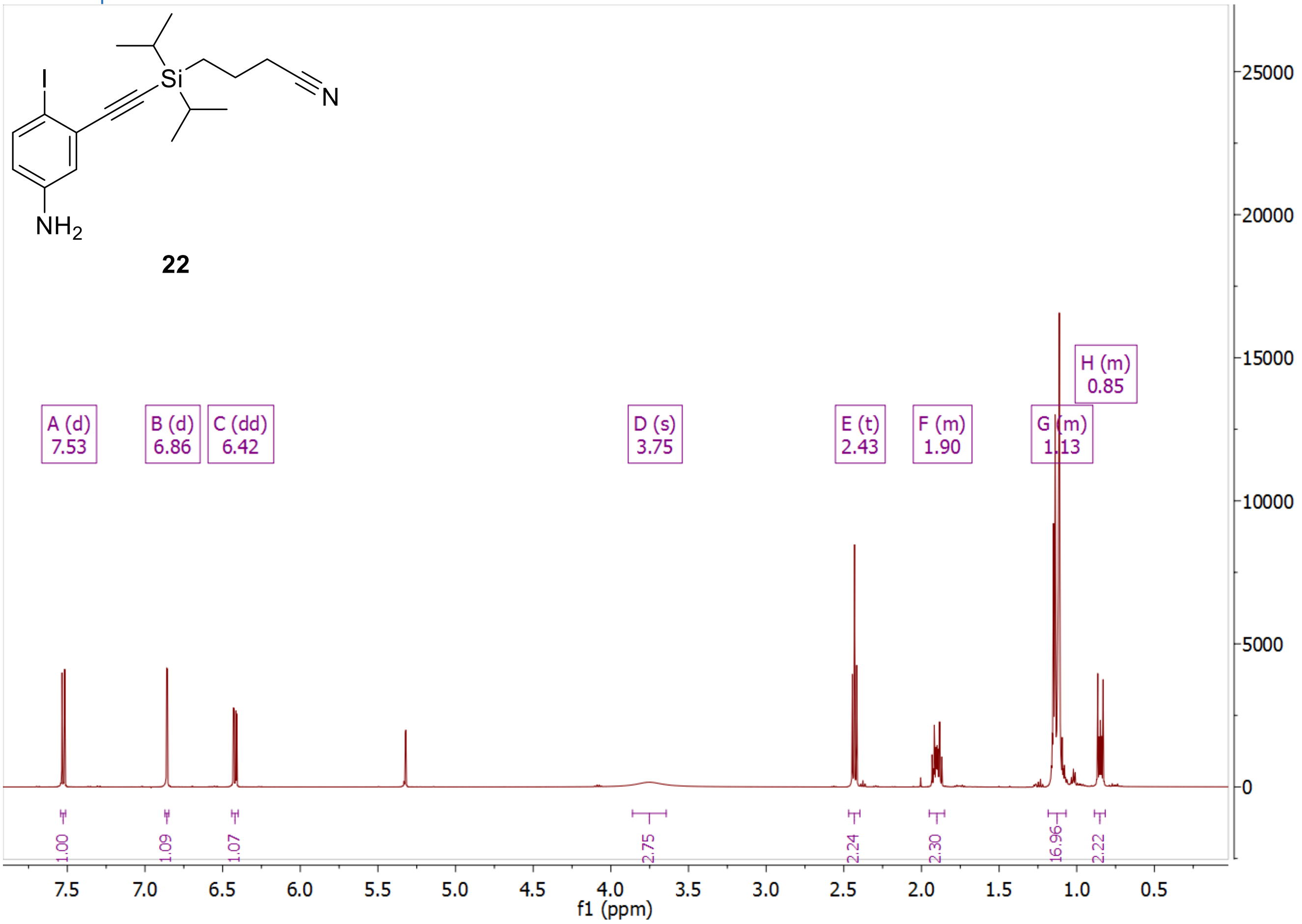

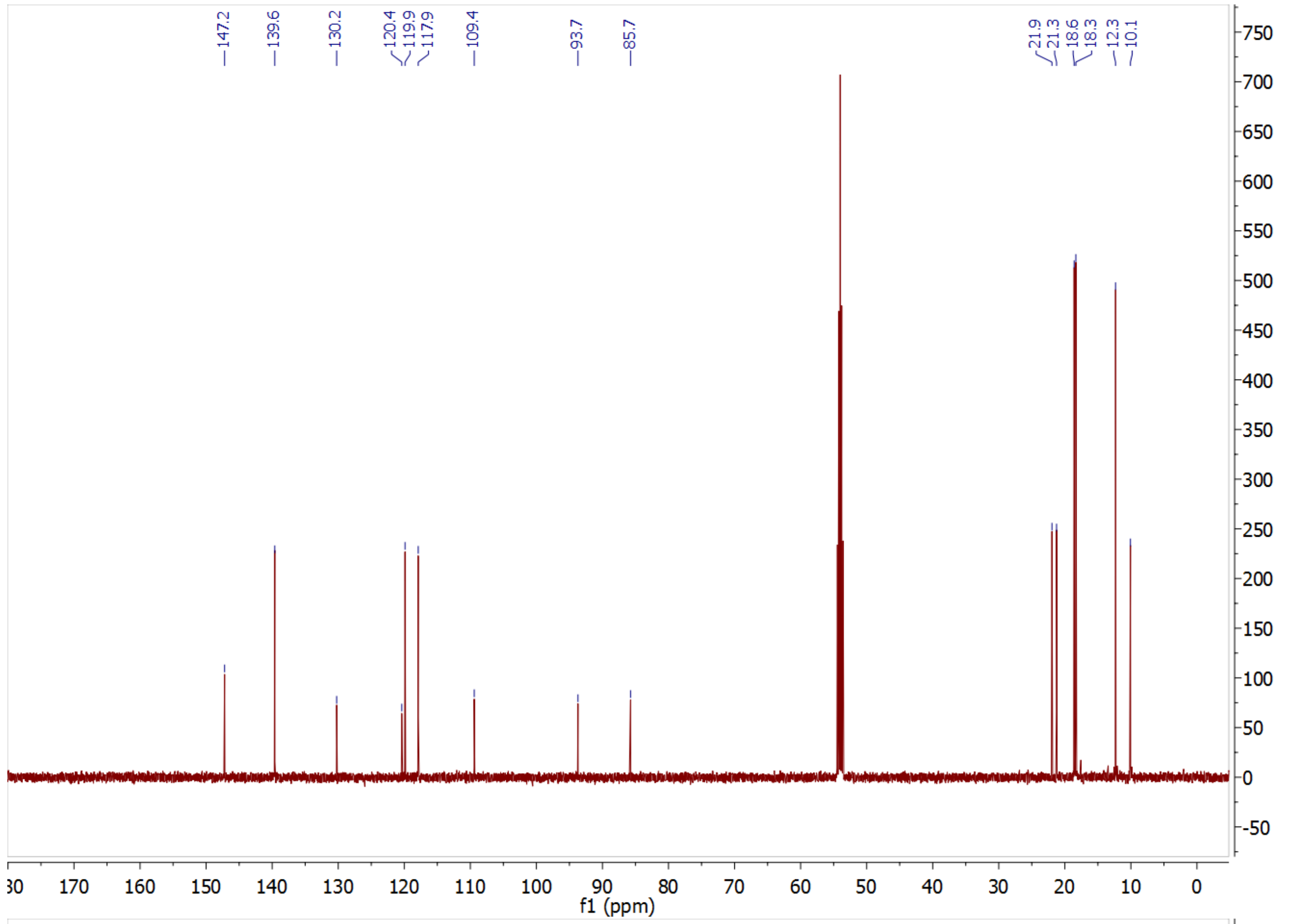
147.2
139.6
130.2
120.4
119.9
117.9
109.4
93.7
85.7
21.9
21.3
18.6
18.3
12.3
10.1
f1 (ppm)

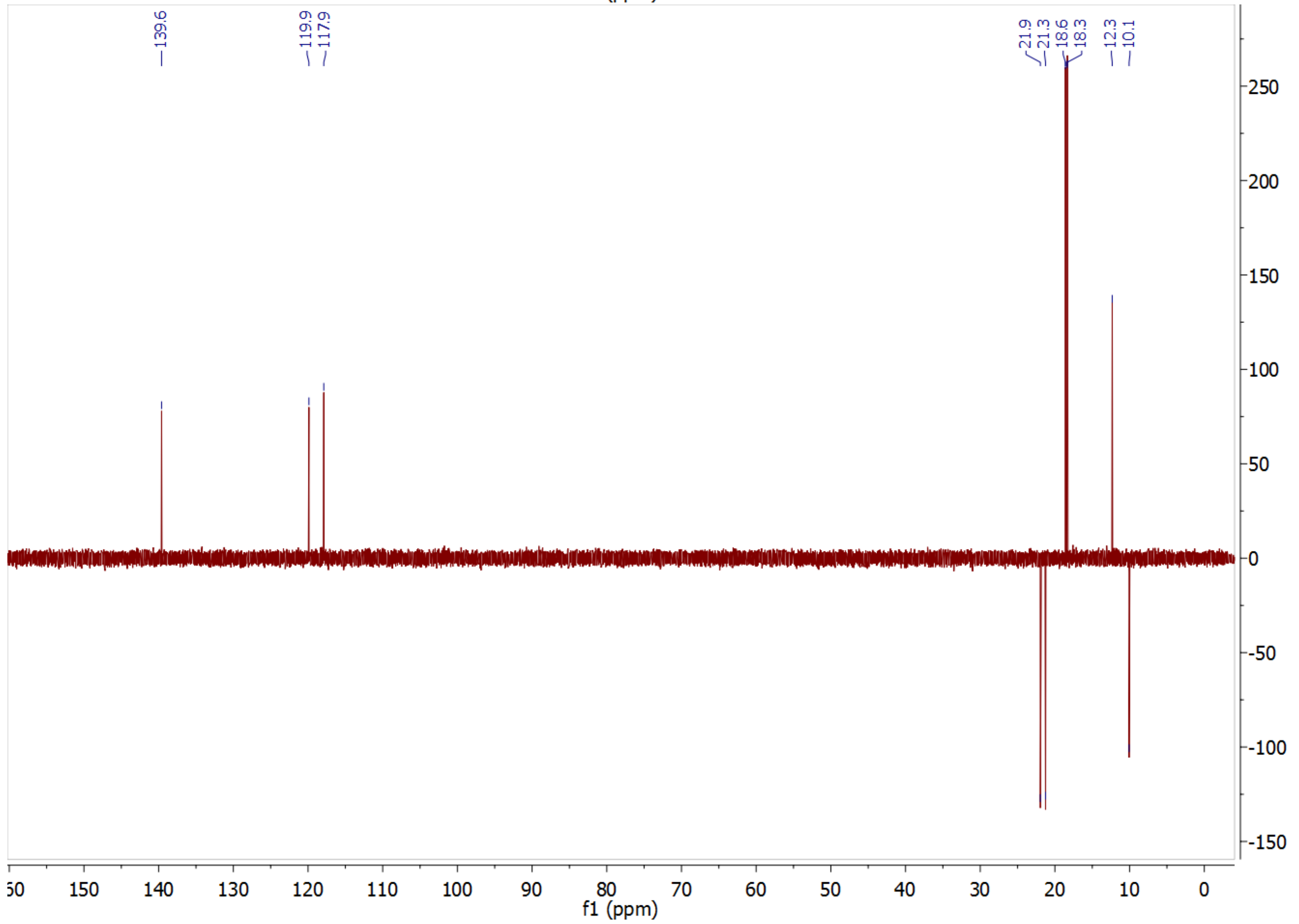
139.6
119.9
117.9
21.9
21.3
18.6
18.3
12.3
10.1
f1 (ppm)

High Resolution Mass Spectrometry Report

Sample Name	**Linda Bannwart / Ba569 chr1 4**	Instrument	maXis 4G
Comment	10 mg/mL in MeOH, analyzed in MeOH	Method	22 Direct_pos_mid.m

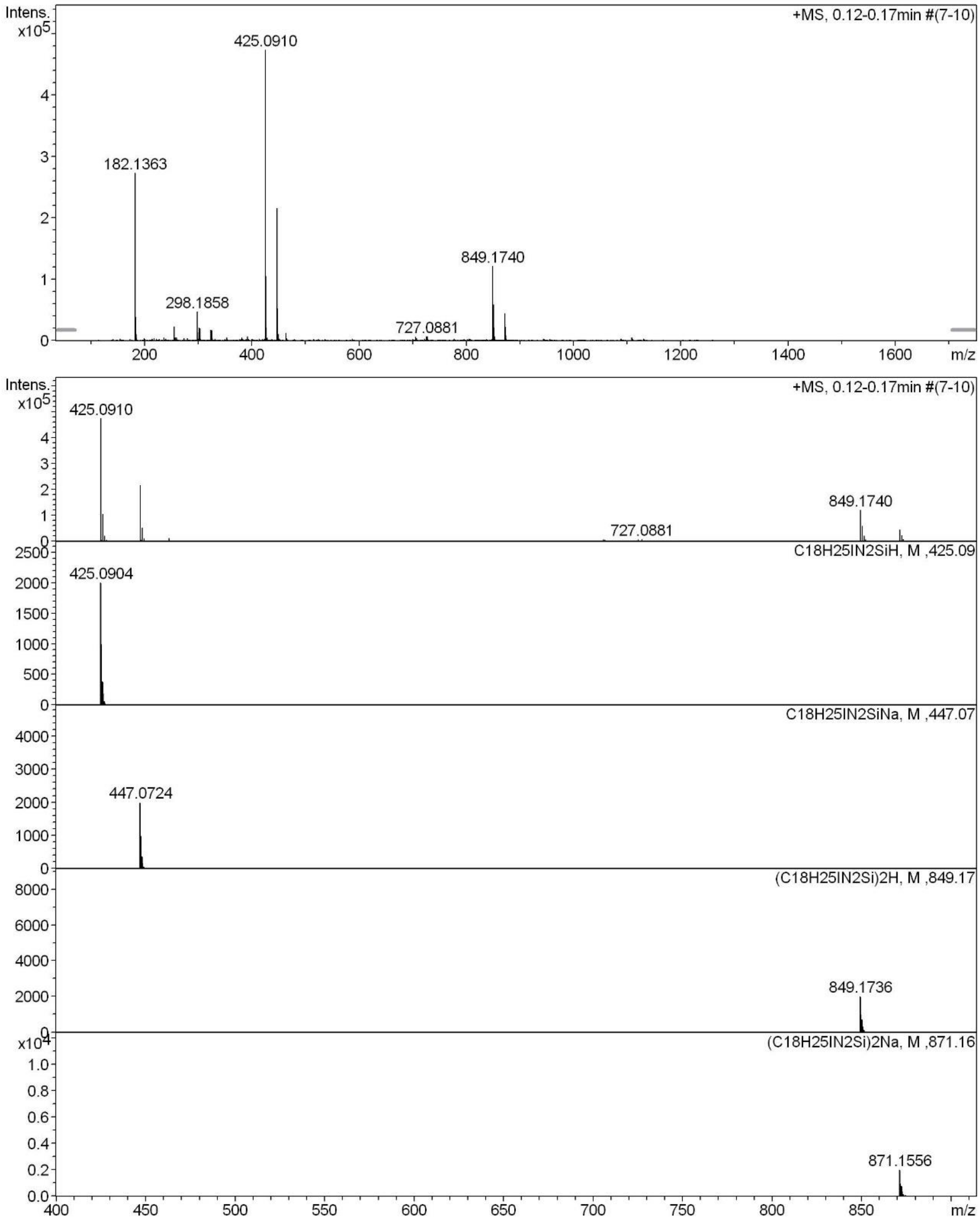

High Resolution Mass Spectrometry Report

Measured m/z vs. theoretical m/z

Meas. m/z	#	Formula	Score	m/z	err [mDa]	err [ppm]	mSigma	rdb	e⁻ Conf	z
425.0910	1	C 18 H 26 I N 2 Si	100.00	425.0904	-0.5	-1.2	19.3	7.5	even	1+
447.0726	1	C 18 H 25 I N 2 Na Si	100.00	447.0724	-0.2	-0.5	11.5	7.5	even	
849.1740	1	C 36 H 51 I 2 N 4 Si 2	100.00	849.1736	-0.4	-0.5	16.1	14.5	even	
871.1554	1	C 36 H 50 I 2 N 4 Na Si 2	100.00	871.1556	0.1	0.1	10.9	14.5	even	

Mass list

#	m/z	I %	I
1	140.9618	0.3	1383
2	154.1049	0.5	2582
3	173.0784	0.3	1358
4	182.1363	57.6	272443
5	183.0783	0.6	2866
6	183.1383	7.9	37333
7	184.1335	2.1	9814
8	185.1152	0.9	4024
9	199.0688	0.6	2805
10	214.0894	0.4	2032
11	217.1051	0.6	2854
12	222.1284	0.4	2050
13	227.0998	0.4	1683
14	236.0714	0.9	4233
15	239.0889	0.4	1922
16	239.5097	0.3	1548
17	255.1309	4.7	22050
18	256.1336	1.0	4508
19	256.9808	1.0	4833
20	258.9786	1.0	4641
21	273.1414	0.6	2685
22	273.1668	0.4	1790
23	279.1585	0.6	2825
24	298.1858	9.9	46984
25	299.1893	2.7	12761
26	300.1897	0.4	2109
27	301.0068	4.4	20949
28	301.1410	0.5	2269
29	302.0100	0.6	2880
30	303.0045	4.1	19490
31	304.0080	0.5	2307
32	322.9886	3.6	16805
33	323.9921	0.5	2157
34	324.9868	3.5	16330
35	325.9898	0.6	2731
36	331.2088	0.5	2568
37	353.1452	0.4	1785
38	353.2658	1.0	4720
39	354.2698	0.3	1355
40	377.1034	0.3	1551
41	379.1022	0.3	1409
42	381.2971	1.0	4935
43	383.0432	0.4	2046
44	391.2838	1.3	6159
45	392.2874	0.4	2120
46	393.2092	0.3	1444
47	393.2972	0.3	1607
48	413.2656	0.6	2642
49	419.3152	0.3	1400
50	423.0746	0.6	2637
51	425.0910	100.0	473159
52	425.3619	0.8	3952
53	426.0931	22.0	104035
54	426.3655	0.4	1723
55	427.0911	4.3	20432
56	428.0918	0.8	3603
57	446.9888	0.3	1607
58	447.0726	45.5	215408
59	447.3455	0.6	2988

High Resolution Mass Spectrometry Report

#	m/z	I %	I
60	448.0750	10.8	51094
61	449.0727	2.1	9967
62	450.0740	0.5	2161
63	451.1069	0.4	1960
64	463.0459	2.5	11733
65	464.0488	0.6	2699
66	465.0460	0.3	1563
67	465.1218	0.3	1636
68	479.1369	0.4	1892
69	524.0174	0.3	1495
70	536.1648	0.4	1679
71	587.2159	0.5	2389
72	699.5962	0.3	1432
73	705.5821	1.2	5622
74	706.5852	0.6	2782
75	721.5761	0.5	2458
76	725.0897	1.3	6320
77	726.0920	0.5	2479
78	727.0881	1.3	6372
79	728.0907	0.5	2427
80	777.3485	0.5	2382
81	805.3809	0.6	2632
82	807.2244	0.3	1391
83	849.1740	25.5	120700
84	849.4479	0.4	1843
85	850.1762	12.3	58281
86	851.1760	4.4	20770
87	852.1767	1.2	5838
88	853.1765	0.3	1462
89	871.1554	9.2	43522
90	872.1580	4.6	21600
91	873.1590	1.6	7647
92	874.1567	0.4	2114
93	943.2222	0.4	2033
94	1087.5366	0.6	2629
95	1088.5403	0.4	2078
96	1107.6828	1.0	4816
97	1108.6862	0.7	3542
98	1109.6868	0.3	1580
99	1129.6656	0.5	2212
100	1130.6680	0.4	1718

Acquisition Parameter

**General**	Fore Vacuum	2.68e+000 mBar	High Vacuum	9.80e-008 mBar	Source Type	ESI
	Scan Begin	75 m/z	Scan End	1700 m/z	Ion Polarity	Positive
**Source**	Set Nebulizer	0.4 Bar	Set Capillary	3600 V	Set Dry Gas	4.0 l/min
	Set Dry Heater	180 °C	Set End Plate Offset	-500 V		
**Quadrupole**	Set Ion Energy (MS only)	4.0 eV				
**Coll. Cell**	Collision Energy	8.0 eV	Set Collision Cell RF	350.0 Vpp		
**Ion Cooler**	Set Ion Cooler Transfer Time	75.0 µs	Set Ion Cooler Pre Pulse Storage Time	10.0 µs		

5.18 Compound **23**

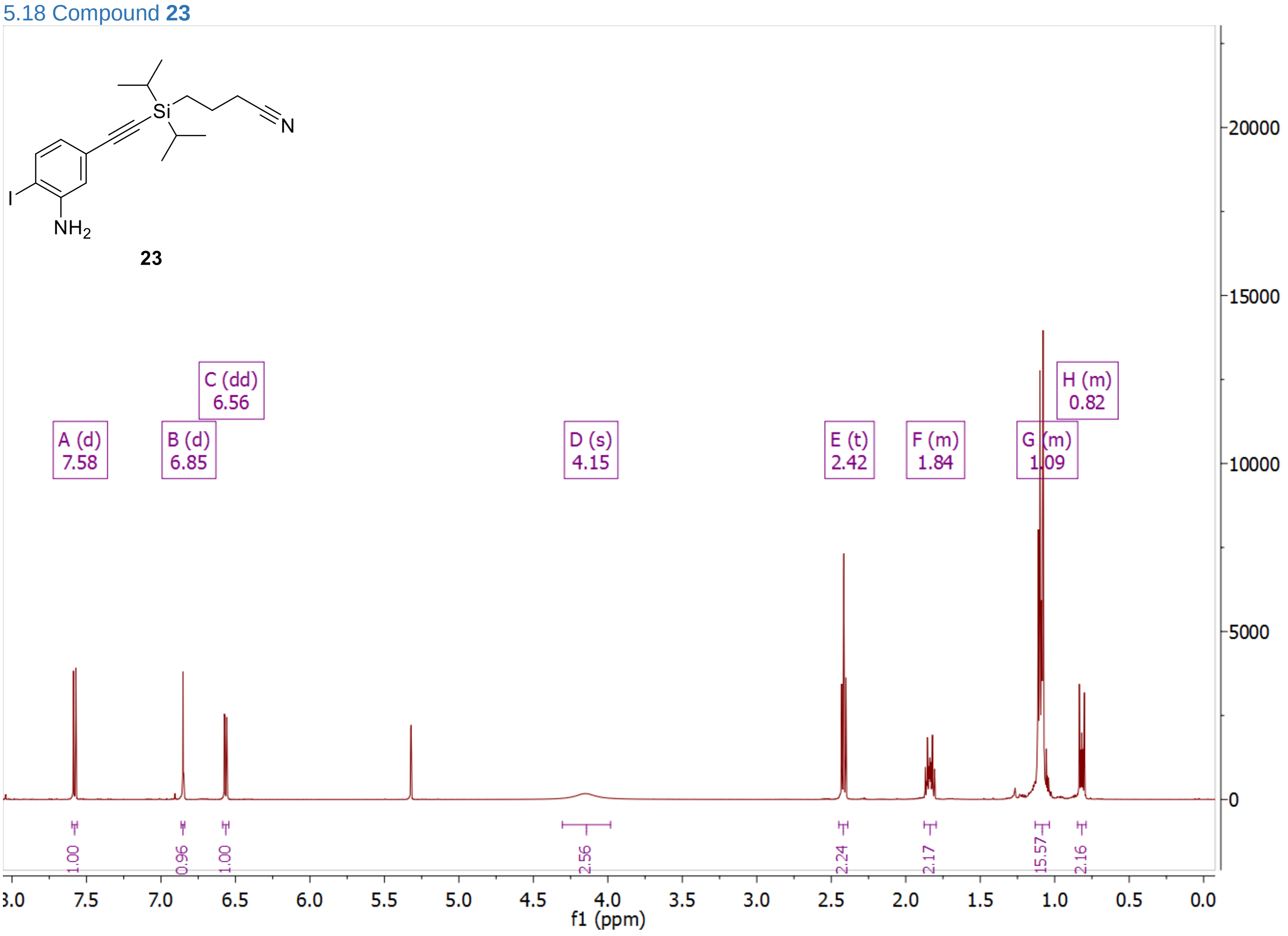

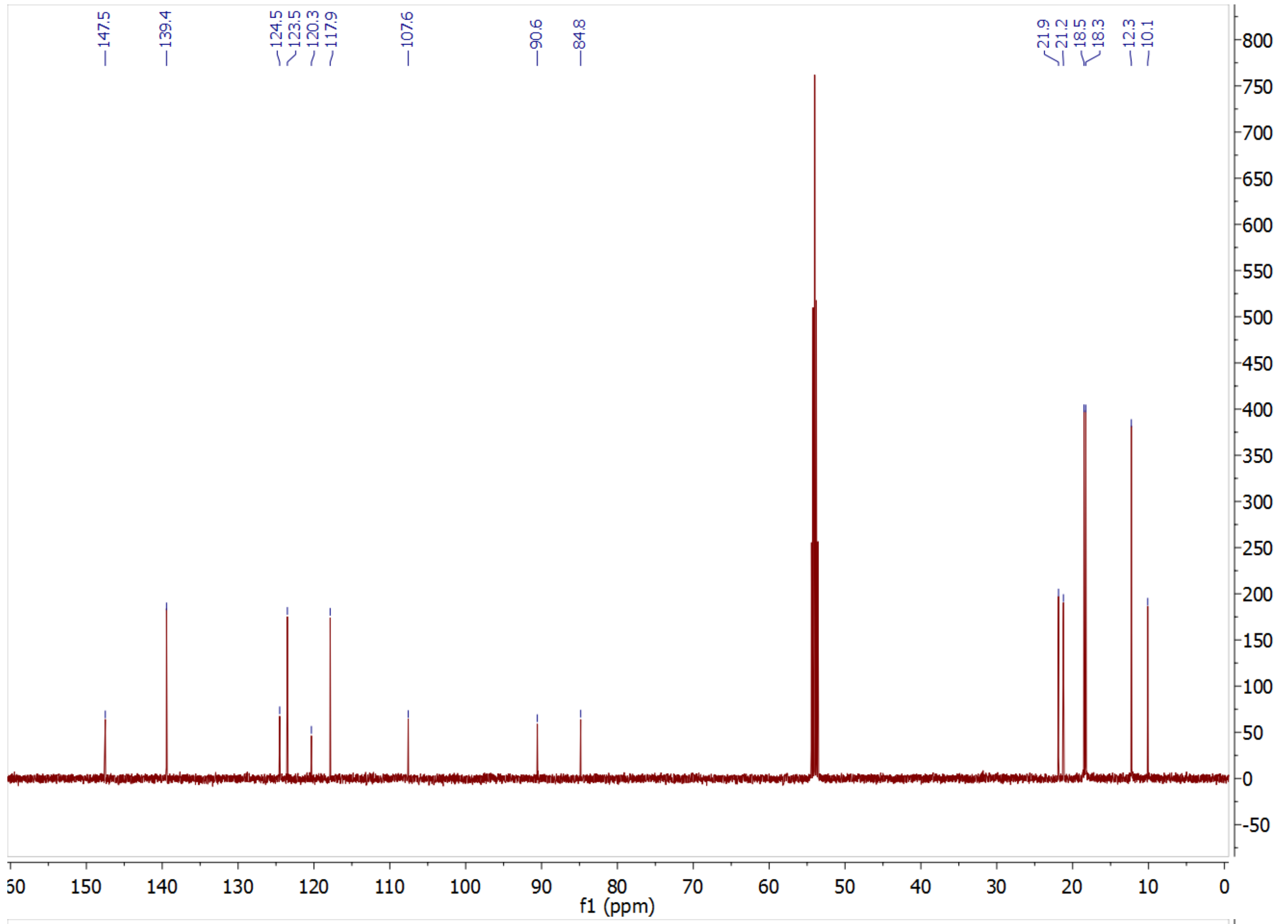
147.5
139.4
124.5
123.5
120.3
117.9
107.6
90.6
84.8
21.9
21.2
18.5
18.3
12.3
10.1
f1 (ppm)

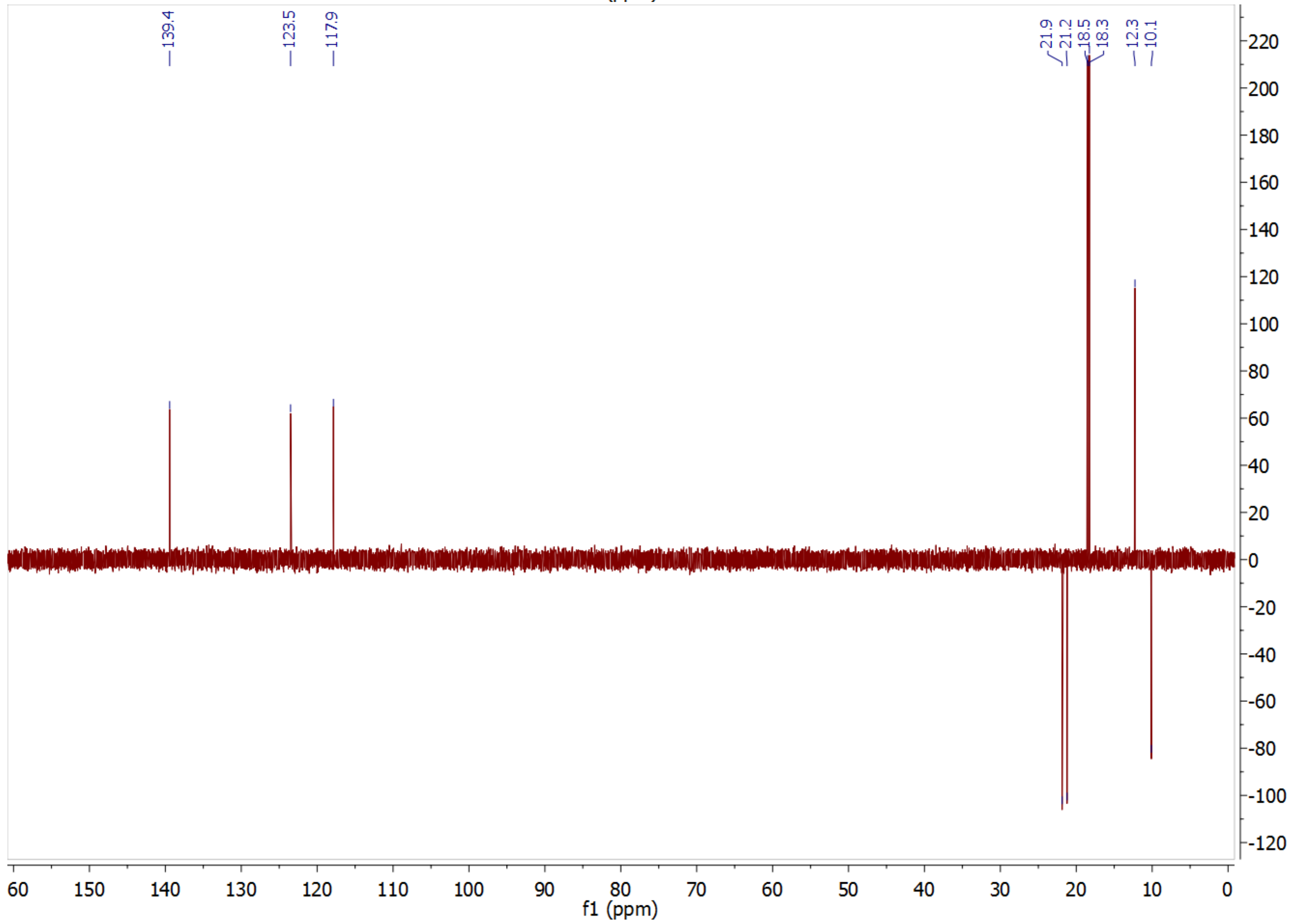
139.4
123.5
117.9
21.9
21.2
18.5
18.3
12.3
10.1
f1 (ppm)

High Resolution Mass Spectrometry Report

Sample Name	**Linda Bannwart / Ba569 chr1 1**	Instrument	maXis 4G
Comment	10 mg/mL in MeOH, analyzed in MeOH	Method	22 Direct_pos_mid.m

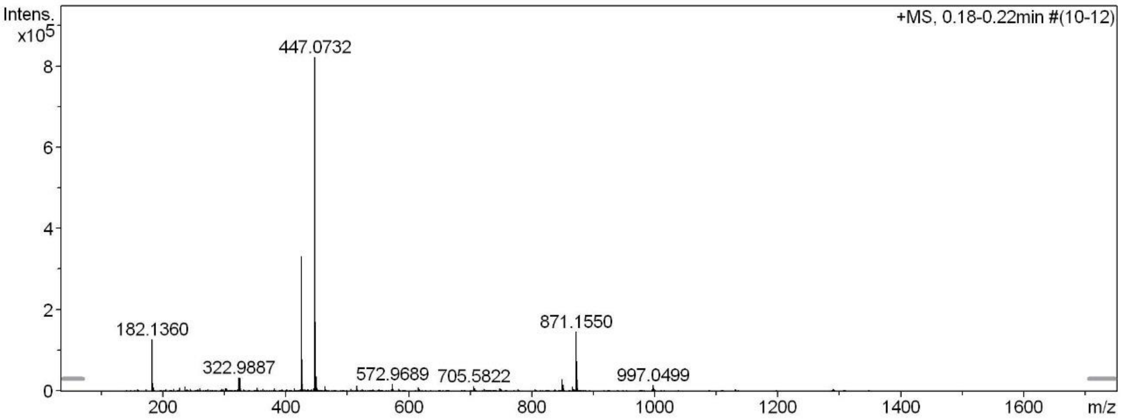

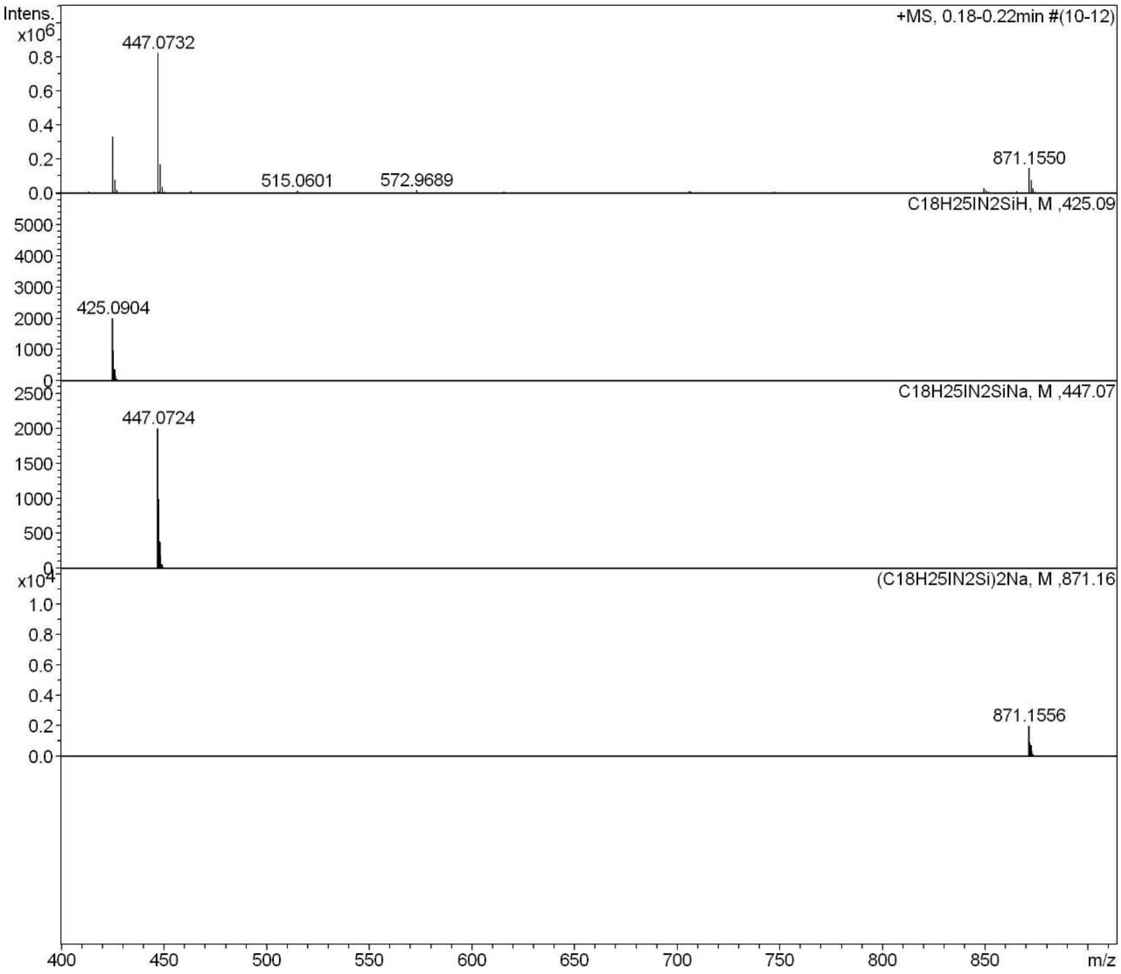

Measured m/z vs. theoretical m/z

Meas. m/z	#	Formula	Score	m/z	err [mDa]	err [ppm]	mSigma	rdb	e⁻ Conf	z
425.0909	1	C 18 H 26 I N 2 Si	100.00	425.0904	-0.5	-1.2	12.0	7.5	even	1+
447.0732	1	C 18 H 25 I N 2 Na Si	100.00	447.0724	-0.8	-1.8	25.9	7.5	even	
871.1550	1	C 36 H 50 I 2 N 4 Na Si 2	100.00	871.1556	0.5	0.6	8.5	14.5	even	

Mass list

#	m/z	I %	I
1	158.9644	0.2	2037
2	173.0787	0.3	2159
3	182.1360	15.4	126376
4	183.1382	2.2	18253
5	184.1333	0.5	4398
6	185.1144	0.9	7467
7	205.0596	0.3	2524
8	217.1042	0.5	3958
9	226.9512	0.9	7275
10	236.0713	1.1	9281
11	239.0884	0.4	3240
12	244.5460	0.5	3792
13	256.9798	0.2	2045
14	260.0229	0.7	5375
15	273.1668	0.3	2380
16	279.2287	0.2	1990
17	294.9381	0.5	4007
18	298.1854	0.4	2976
19	301.0066	0.7	5561
20	301.1404	0.6	5003
21	303.0043	0.7	5859
22	304.2607	0.3	2116
23	321.2765	0.3	2266
24	322.9887	3.8	31563
25	323.9919	0.5	4475
26	324.9867	3.7	30153
27	325.9902	0.6	4921
28	331.2086	0.4	2908
29	353.1440	0.3	2417
30	353.2661	0.8	6165
31	362.9262	0.5	3758
32	381.2972	0.7	5932
33	393.2976	0.4	3230
34	401.0837	0.2	2040
35	413.2657	0.7	5778
36	423.0741	0.4	2910
37	425.0909	40.2	330204
38	426.0932	9.4	77131
39	427.0907	2.0	16150
40	428.0905	0.4	2893
41	430.9141	0.4	3011
42	435.2615	0.4	3009
43	436.0750	0.2	2039
44	441.2973	0.3	2679
45	445.0565	0.7	5923
46	446.9684	0.3	2649
47	446.9956	0.4	2876
48	447.0732	100.0	820805
49	447.2965	0.3	2320
50	447.3446	0.7	5451
51	448.0753	20.5	168523
52	449.0732	4.2	34732
53	449.3656	0.3	2604
54	450.0731	0.7	6078
55	451.5519	0.3	2285
56	463.0461	1.3	10519
57	464.0483	0.3	2596
58	505.1531	0.4	3571
59	515.0601	1.5	12505
60	516.0625	0.4	3118

High Resolution Mass Spectrometry Report

#	m/z	I %	I
61	524.0186	0.4	3058
62	541.1195	0.4	2965
63	550.9864	0.3	2619
64	570.9535	0.3	2526
65	572.9689	1.9	15283
66	573.9720	0.5	3926
67	583.0465	0.5	4461
68	615.3308	0.9	7412
69	616.3336	0.5	4084
70	705.5822	1.3	10855
71	706.5859	0.6	5095
72	721.5771	0.5	3702
73	722.5809	0.2	2026
74	747.0717	0.6	4895
75	749.0699	0.5	4171
76	750.0723	0.3	2076
77	777.3497	0.4	2902
78	805.3808	0.4	3066
79	837.3511	0.3	2236
80	849.1726	3.4	28158
81	850.1751	1.7	14338
82	851.1738	0.7	5485
83	865.1083	1.2	9625
84	866.1108	0.6	4535
85	867.1188	0.5	4209
86	868.1234	0.3	2282
87	869.1395	0.4	3159
88	871.1550	17.6	144418
89	871.4264	0.3	2279
90	872.1575	8.8	71999
91	873.1577	3.2	26024
92	874.1582	0.8	6800
93	995.0340	0.3	2705
94	997.0499	1.6	13413
95	998.0520	0.7	6032
96	999.0530	0.3	2413
97	1129.6639	0.4	3101
98	1130.6649	0.2	2044
99	1289.1870	0.5	3757
100	1290.1866	0.4	2898

Acquisition Parameter

**General**	Fore Vacuum	2.68e+000 mBar	High Vacuum	9.87e-008 mBar	Source Type	ESI
	Scan Begin	75 m/z	Scan End	1700 m/z	Ion Polarity	Positive
**Source**	Set Nebulizer	0.4 Bar	Set Capillary	3600 V	Set Dry Gas	4.0 l/min
	Set Dry Heater	180 °C	Set End Plate Offset	-500 V		
**Quadrupole**	Set Ion Energy (MS only)	4.0 eV				
**Coll. Cell**	Collision Energy	8.0 eV	Set Collision Cell RF	350.0 Vpp		
**Ion Cooler**	Set Ion Cooler Transfer Time	75.0 µs	Set Ion Cooler Pre Pulse Storage Time	10.0 µs		

5.19 Compound **24**

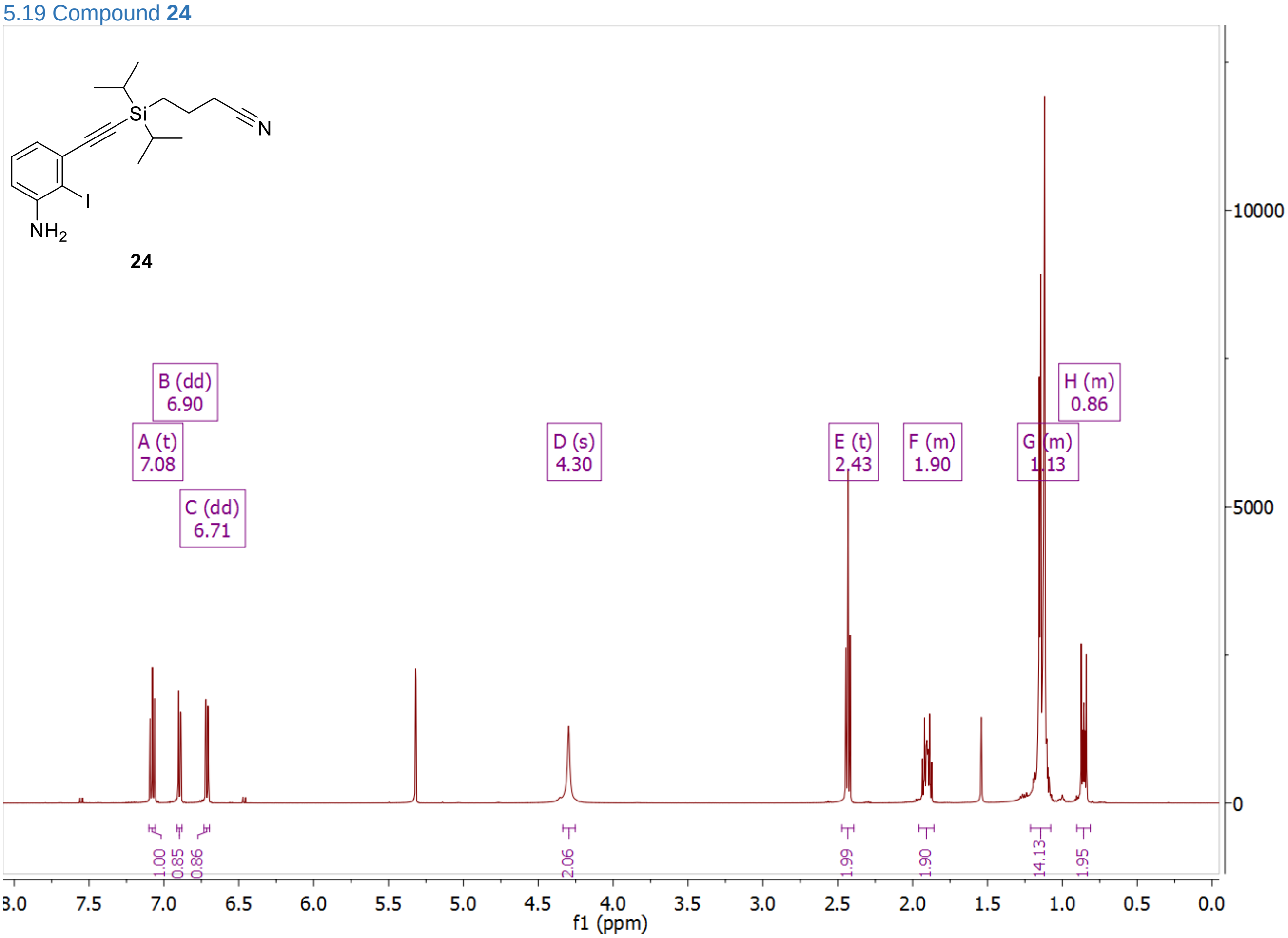

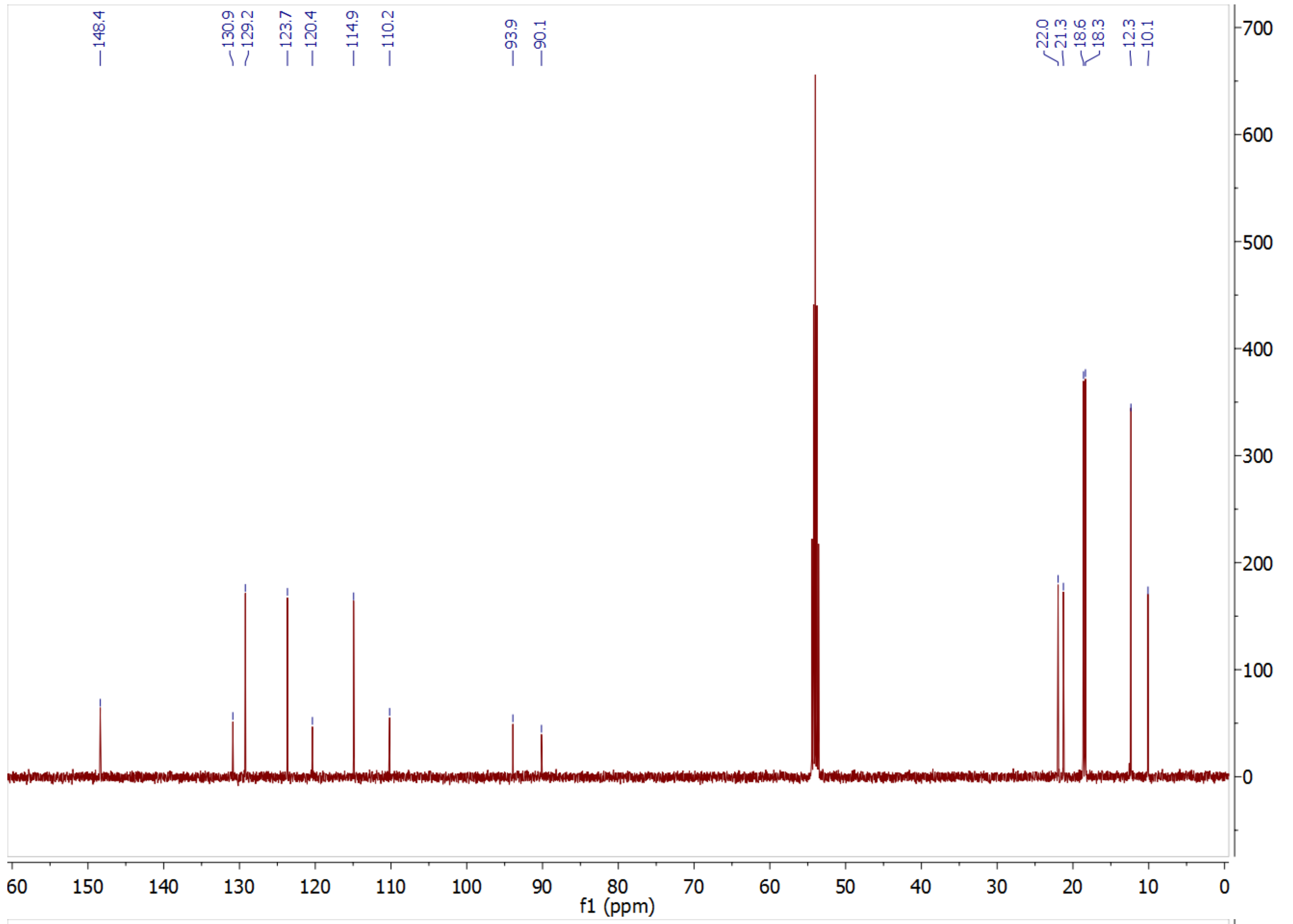
148.4
130.9
129.2
123.7
120.4
114.9
110.2
93.9
90.1
22.0
21.3
18.6
18.3
12.3
10.1
f1 (ppm)

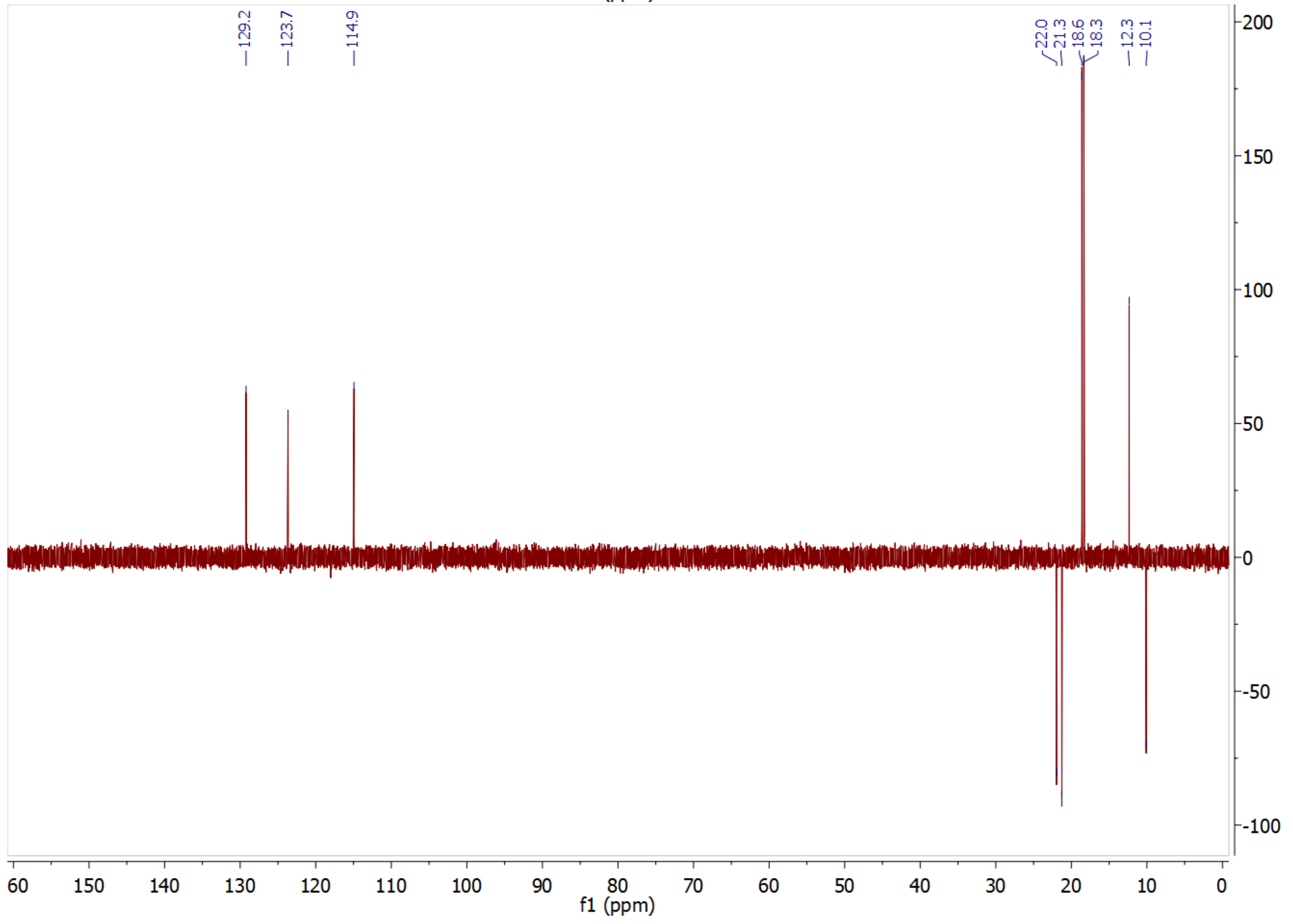
129.2
123.7
114.9
22.0
21.3
18.6
18.3
12.3
10.1
f1 (ppm)

High Resolution Mass Spectrometry Report

Sample Name	**Linda Bannwart / Ba569 GPC1-2**	Instrument	maXis 4G
Comment	10 ug/mL in MeOH, analyzed in MeOH	Method	1 CALIB_702.m

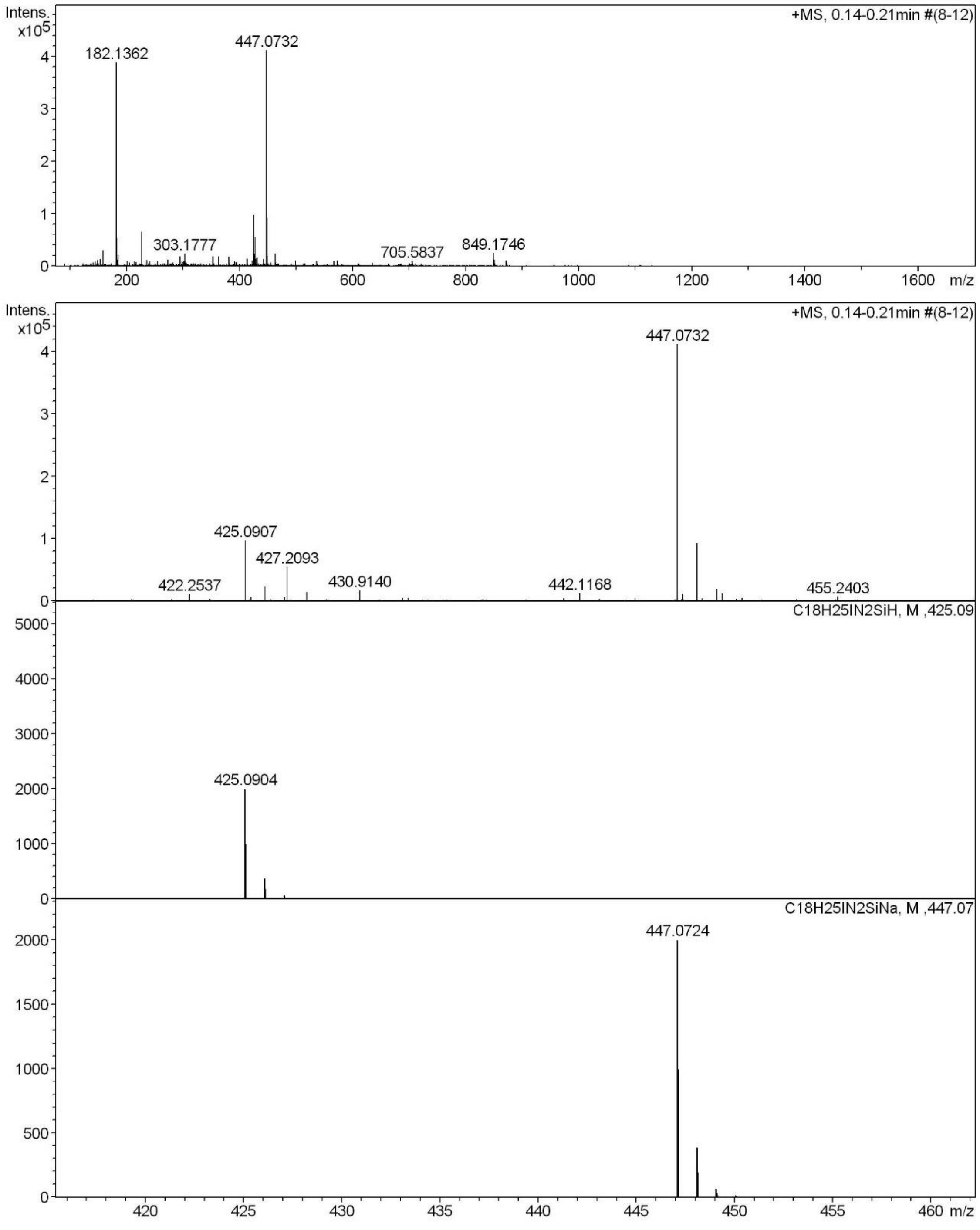

High Resolution Mass Spectrometry Report

Measured m/z vs. theoretical m/z

Meas. m/z	#	Formula	Score	m/z	err [mDa]	err [ppm]	mSigma	rdb	e⁻ Conf	z
425.0907	1	C 18 H 26 I N 2 Si	100.00	425.0904	-0.3	-0.6	11.9	7.5	even	1+
447.0732	1	C 18 H 25 I N 2 Na Si	100.00	447.0724	-0.9	-1.9	17.2	7.5	even	

Mass list

#	m/z	I %	I
1	123.0918	1.1	4321
2	136.1123	1.0	4015
3	140.9611	1.6	6455
4	144.9569	1.9	7959
5	147.0916	1.0	3998
6	149.0232	2.5	10353
7	150.1275	1.1	4708
8	154.1046	3.1	12628
9	158.9641	7.3	29905
10	173.0784	1.2	4834
11	182.1362	94.4	387608
12	183.0778	1.4	5572
13	183.1382	12.9	53046
14	184.1334	2.9	11904
15	185.1148	5.0	20610
16	201.1039	2.1	8627
17	205.0607	1.5	6241
18	214.0894	2.0	8186
19	217.1052	1.7	6977
20	223.1320	0.9	3671
21	226.9515	15.7	64563
22	236.0715	2.5	10360
23	239.0892	1.1	4451
24	240.9671	1.9	7666
25	255.1307	2.2	8897
26	255.1815	1.8	7283
27	265.1775	1.1	4432
28	267.1566	1.0	4073
29	273.1918	2.7	11207
30	279.1583	0.9	3592
31	282.2789	1.6	6384
32	294.9389	4.4	18189
33	298.1860	1.9	7694
34	299.1614	0.9	3765
35	299.1930	1.7	6846
36	301.1410	1.9	7681
37	301.2115	1.2	4762
38	303.1777	5.6	23075
39	304.2612	2.2	9135
40	305.2087	1.3	5412
41	314.2182	1.0	3928
42	319.2245	1.2	4757
43	322.1912	1.2	4766
44	331.2096	1.1	4523
45	347.2010	1.2	4899
46	347.2556	0.9	3655
47	353.1453	1.2	4810
48	353.2662	4.4	18222
49	354.2703	1.1	4428
50	362.9264	4.3	17673
51	376.9419	0.9	3657
52	381.2975	4.1	16991
53	382.3009	1.1	4532
54	391.2842	1.9	7638
55	393.2973	1.8	7496
56	395.1813	1.3	5393
57	413.1935	2.1	8423
58	413.2664	3.3	13544
59	414.2698	0.9	3697
60	422.2537	2.5	10180
61	425.0907	23.5	96661

High Resolution Mass Spectrometry Report

#	m/z	I %	I
62	425.3632	1.4	5590
63	426.0931	5.4	22340
64	427.0911	1.3	5153
65	427.2093	13.2	54358
66	428.2126	3.3	13426
67	430.9140	4.0	16555
68	433.1026	1.1	4324
69	433.3804	1.1	4359
70	441.2976	0.9	3753
71	442.1168	3.0	12403
72	444.9292	1.0	4296
73	447.0732	100.0	410738
74	447.3454	2.6	10586
75	448.0753	22.3	91428
76	449.0733	4.7	19173
77	449.3750	2.7	11137
78	450.3792	1.1	4468
79	455.2403	1.5	6030
80	463.0466	5.7	23451
81	464.0489	1.4	5826
82	498.9014	2.5	10097
83	515.0598	1.1	4658
84	536.1651	2.1	8778
85	537.1667	1.1	4445
86	566.8884	2.2	9071
87	572.9688	2.4	9921
88	610.1843	1.2	4724
89	634.8760	1.3	5513
90	663.4535	1.0	3932
91	685.4358	1.0	4020
92	700.6269	1.1	4410
93	705.5837	2.2	9108
94	706.5861	1.0	4196
95	711.3504	0.9	3621
96	849.1746	5.8	23786
97	850.1766	2.9	11846
98	851.1782	1.0	4262
99	871.1559	2.4	9907
100	872.1591	1.4	5597

Acquisition Parameter

**General**	Fore Vacuum	2.79e+000 mBar	High Vacuum	9.59e-008 mBar	Source Type	ESI
	Scan Begin	75 m/z	Scan End	1700 m/z	Ion Polarity	Positive
**Source**	Set Nebulizer	0.4 Bar	Set Capillary	3600 V	Set Dry Gas	3.0 l/min
	Set Dry Heater	180 °C	Set End Plate Offset	-500 V		
**Quadrupole**	Set Ion Energy (MS only)	4.0 eV				
**Coll. Cell**	Collision Energy	8.0 eV	Set Collision Cell RF	350.0 Vpp		
**Ion Cooler**	Set Ion Cooler Transfer Time	55.0 µs	Set Ion Cooler Pre Pulse Storage Time	7.0 µs		

5.20 Compound **25**

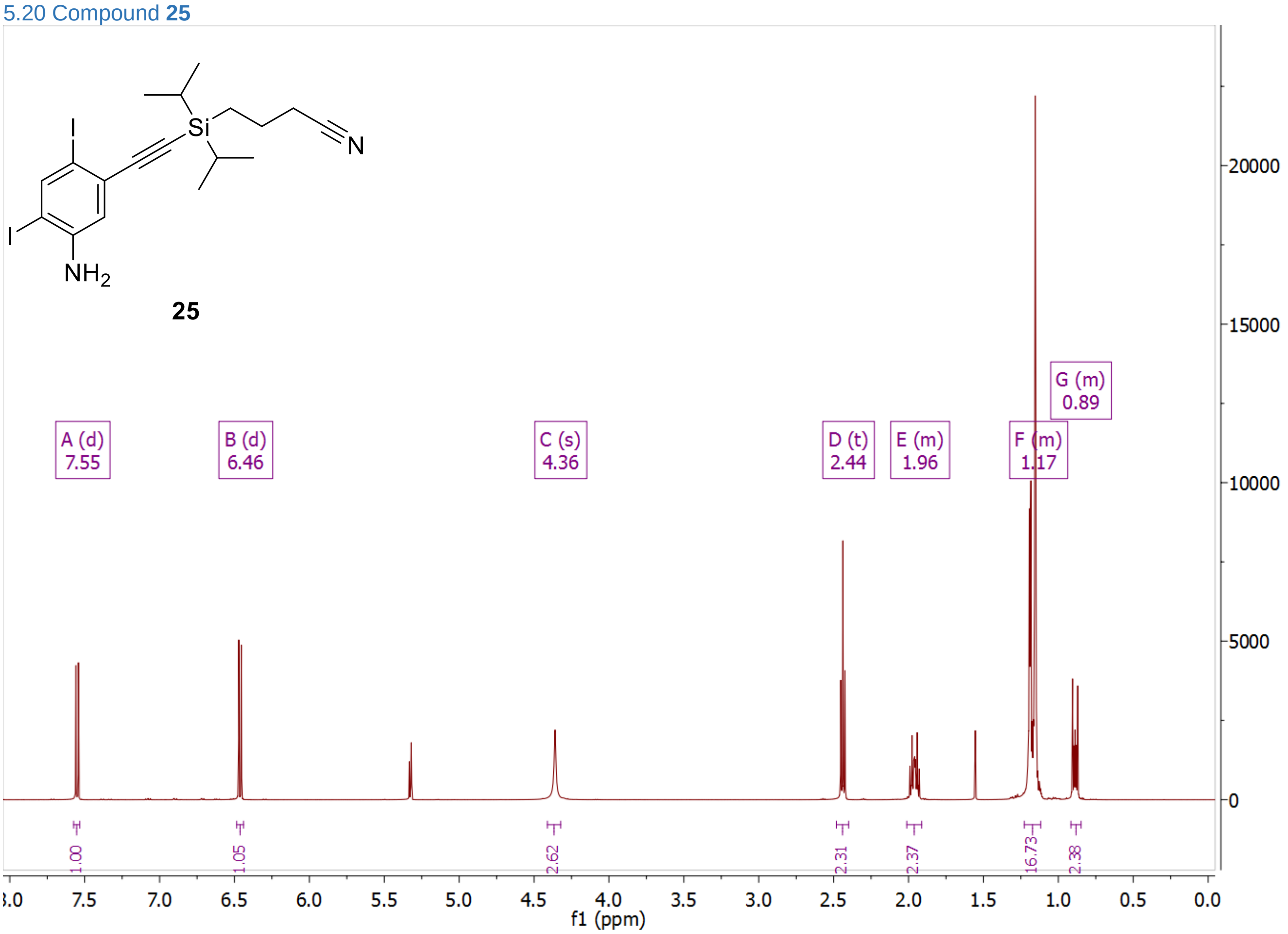

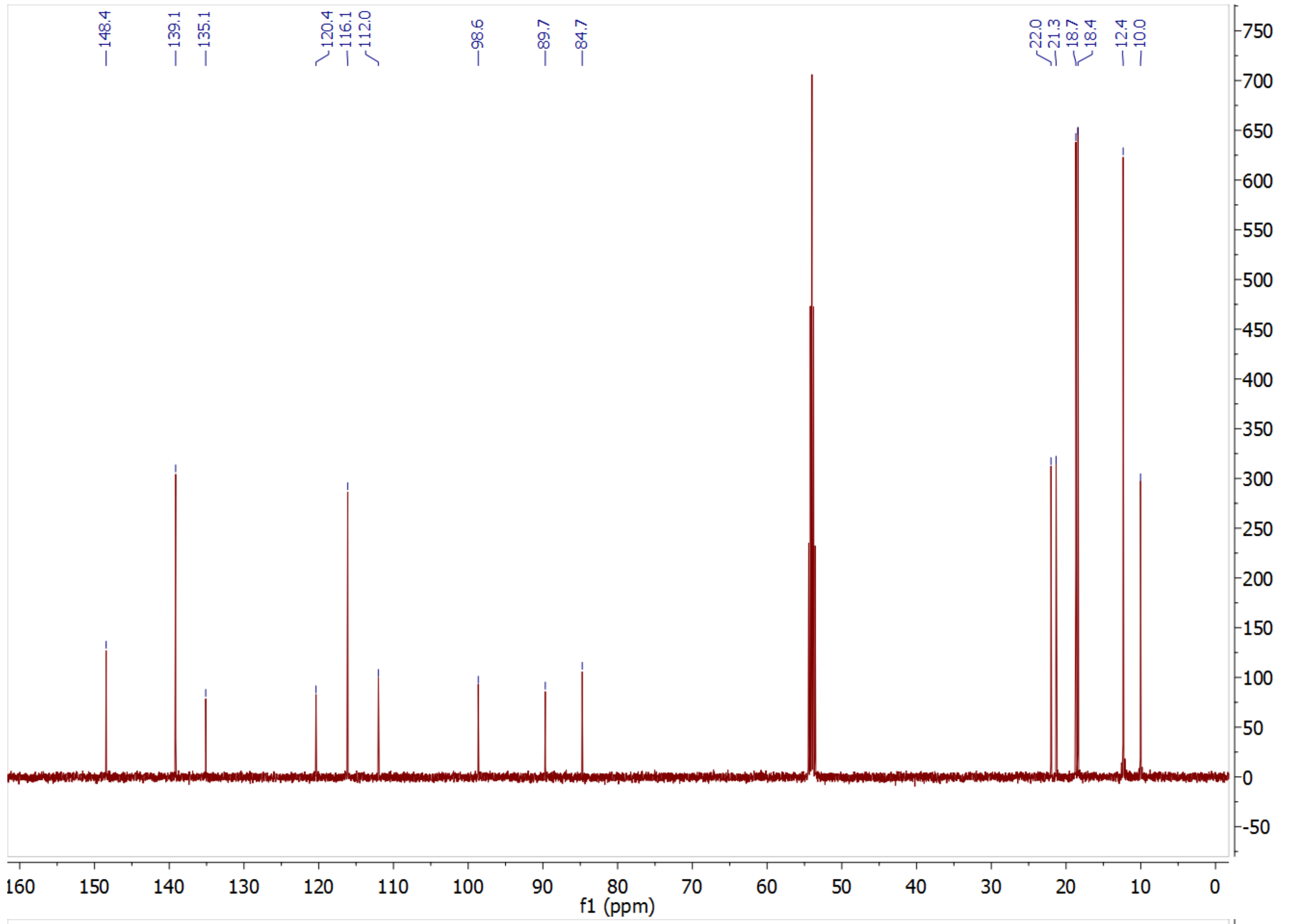
148.4
139.1
135.1
120.4
116.1
112.0
98.6
89.7
84.7
22.0
21.3
18.7
18.4
12.4
10.0
f1 (ppm)

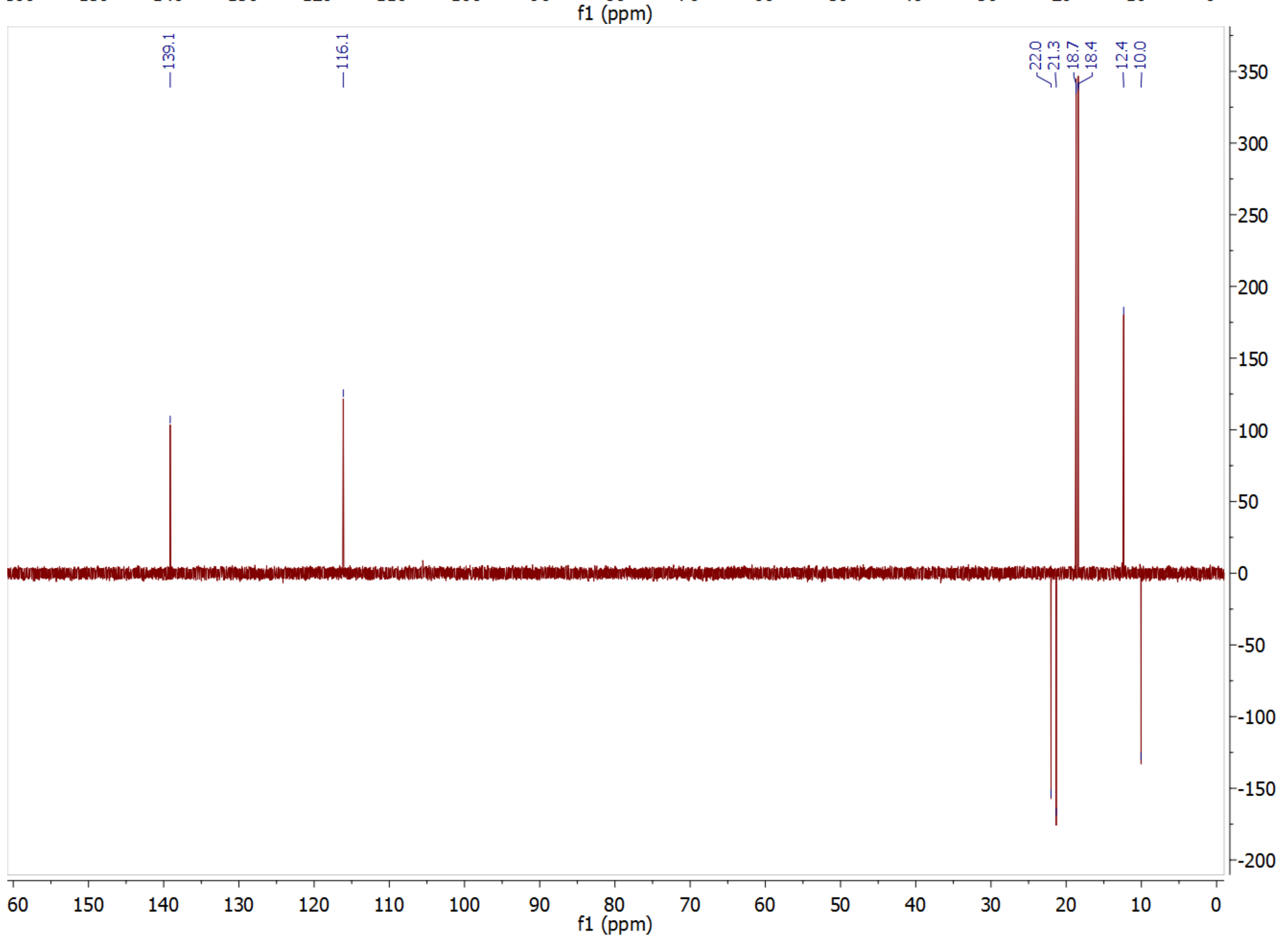
139.1
116.1
22.0
21.3
18.7
18.4
12.4
10.0
f1 (ppm)

High Resolution Mass Spectrometry Report

Sample Name	**Linda Bannwart / Ba569 GPC1-3**	Instrument	maXis 4G
Comment	10 ug/mL in MeOH, analyzed in MeOH	Method	22 Direct_pos_mid.m

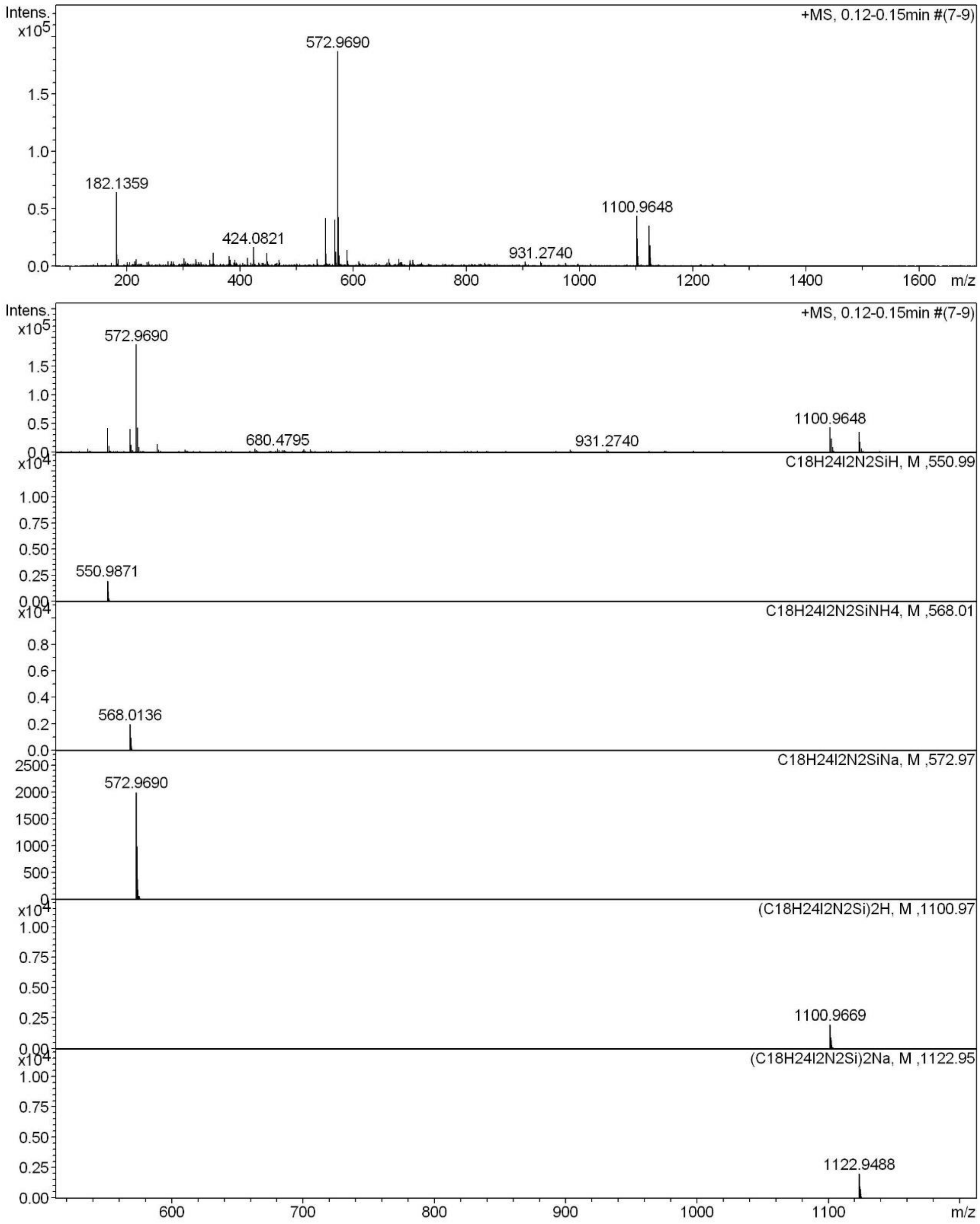

High Resolution Mass Spectrometry Report

Measured m/z vs. theoretical m/z

Meas. m/z	#	Formula	Score	m/z	err [mDa]	err [ppm]	mSigma	rdb	e⁻ Conf	z
550.9867	1	C 18 H 25 I 2 N 2 Si	100.00	550.9871	0.4	0.7	1.9	7.5	even	1+
568.0134	1	C 18 H 28 I 2 N 3 Si	100.00	568.0136	0.2	0.4	26.5	6.5	even	
572.9690	1	C 18 H 24 I 2 N 2 Na Si	100.00	572.9690	0.0	0.0	14.8	7.5	even	
588.9427	1	C 18 H 24 I 2 K N 2 Si	100.00	588.9430	0.3	0.5	31.1	7.5	even	
1100.9648	1	C 36 H 49 I 4 N 4 Si 2	100.00	1100.9669	2.1	1.9	15.6	14.5	even	

Mass list

#	m/z	I %	I
1	149.0231	1.4	2635
2	173.0787	1.4	2658
3	182.1359	34.3	64152
4	183.0780	2.2	4108
5	183.1381	5.2	9723
6	184.1331	1.2	2208
7	185.1146	3.1	5841
8	201.1029	1.7	3102
9	205.0603	1.7	3188
10	214.0890	2.2	4185
11	217.1055	3.0	5524
12	236.0712	1.8	3454
13	239.0886	1.9	3633
14	273.1667	2.2	4199
15	279.1581	2.2	4110
16	279.2289	1.3	2422
17	282.2784	2.1	3846
18	293.2079	1.2	2200
19	299.1606	1.1	2073
20	301.1402	3.6	6658
21	304.2606	2.0	3810
22	307.4949	1.4	2566
23	315.1935	1.2	2183
24	322.1903	3.0	5646
25	322.9713	2.0	3790
26	327.0777	1.7	3175
27	331.2087	1.8	3457
28	347.2006	2.9	5414
29	353.1463	1.4	2657
30	353.2654	6.1	11439
31	354.2703	1.1	2120
32	365.1055	1.2	2180
33	381.0279	2.9	5338
34	381.2969	4.7	8714
35	382.3004	1.3	2395
36	383.1404	2.8	5325
37	389.2502	1.5	2851
38	391.2834	2.9	5446
39	393.2966	1.5	2794
40	395.2762	1.1	2077
41	405.1227	1.4	2558
42	413.2660	3.7	6932
43	419.3142	1.4	2710
44	424.0821	8.7	16252
45	425.0873	2.9	5336
46	425.3629	2.5	4727
47	433.1019	1.5	2829
48	439.2028	1.2	2339
49	441.2964	1.3	2387
50	447.0715	2.3	4313
51	447.3455	6.0	11180
52	448.3495	2.2	4115
53	449.3736	2.0	3785
54	463.3742	1.2	2201
55	465.3698	1.2	2257
56	469.3280	2.8	5291
57	470.3319	1.4	2679
58	536.1649	3.0	5539

High Resolution Mass Spectrometry Report

#	m/z	I %	I
59	537.1661	1.2	2245
60	550.9867	22.4	41851
61	551.9890	5.7	10605
62	552.9868	1.4	2643
63	568.0134	21.4	39986
64	569.0148	6.6	12330
65	570.0134	1.6	2946
66	572.9690	100.0	187108
67	573.9712	22.8	42658
68	574.9688	4.7	8797
69	588.9427	7.5	14058
70	589.9451	2.3	4368
71	610.1848	2.1	3928
72	611.1837	1.3	2339
73	640.9561	1.3	2422
74	659.5030	1.4	2683
75	663.4530	3.2	5947
76	664.4566	1.6	2945
77	680.4795	3.2	6000
78	681.4834	1.5	2872
79	684.2022	1.7	3244
80	685.4350	1.8	3364
81	699.5955	1.1	2121
82	700.6270	2.6	4828
83	701.6291	1.3	2429
84	705.5822	2.8	5266
85	706.5839	1.5	2763
86	721.5754	1.5	2825
87	758.2201	1.2	2164
88	832.2572	1.3	2370
89	903.2463	2.0	3744
90	931.2740	2.0	3776
91	932.2795	1.3	2526
92	975.0677	1.3	2441
93	1100.9648	23.3	43564
94	1101.9675	12.6	23595
95	1102.9655	4.6	8660
96	1103.9664	1.2	2267
97	1122.9460	18.8	35136
98	1123.9484	9.6	17936
99	1124.9484	3.7	6858
100	1125.9467	1.1	2069

Acquisition Parameter

**General**	Fore Vacuum	2.80e+000 mBar	High Vacuum	9.59e-008 mBar	Source Type	ESI
	Scan Begin	75 m/z	Scan End	1700 m/z	Ion Polarity	Positive
**Source**	Set Nebulizer	0.4 Bar	Set Capillary	3600 V	Set Dry Gas	4.0 l/min
	Set Dry Heater	180 °C	Set End Plate Offset	-500 V		
**Quadrupole**	Set Ion Energy (MS only)	4.0 eV				
**Coll. Cell**	Collision Energy	8.0 eV	Set Collision Cell RF	350.0 Vpp		
**Ion Cooler**	Set Ion Cooler Transfer Time	75.0 µs	Set Ion Cooler Pre Pulse Storage Time	10.0 µs		

5.21 Compound **26**

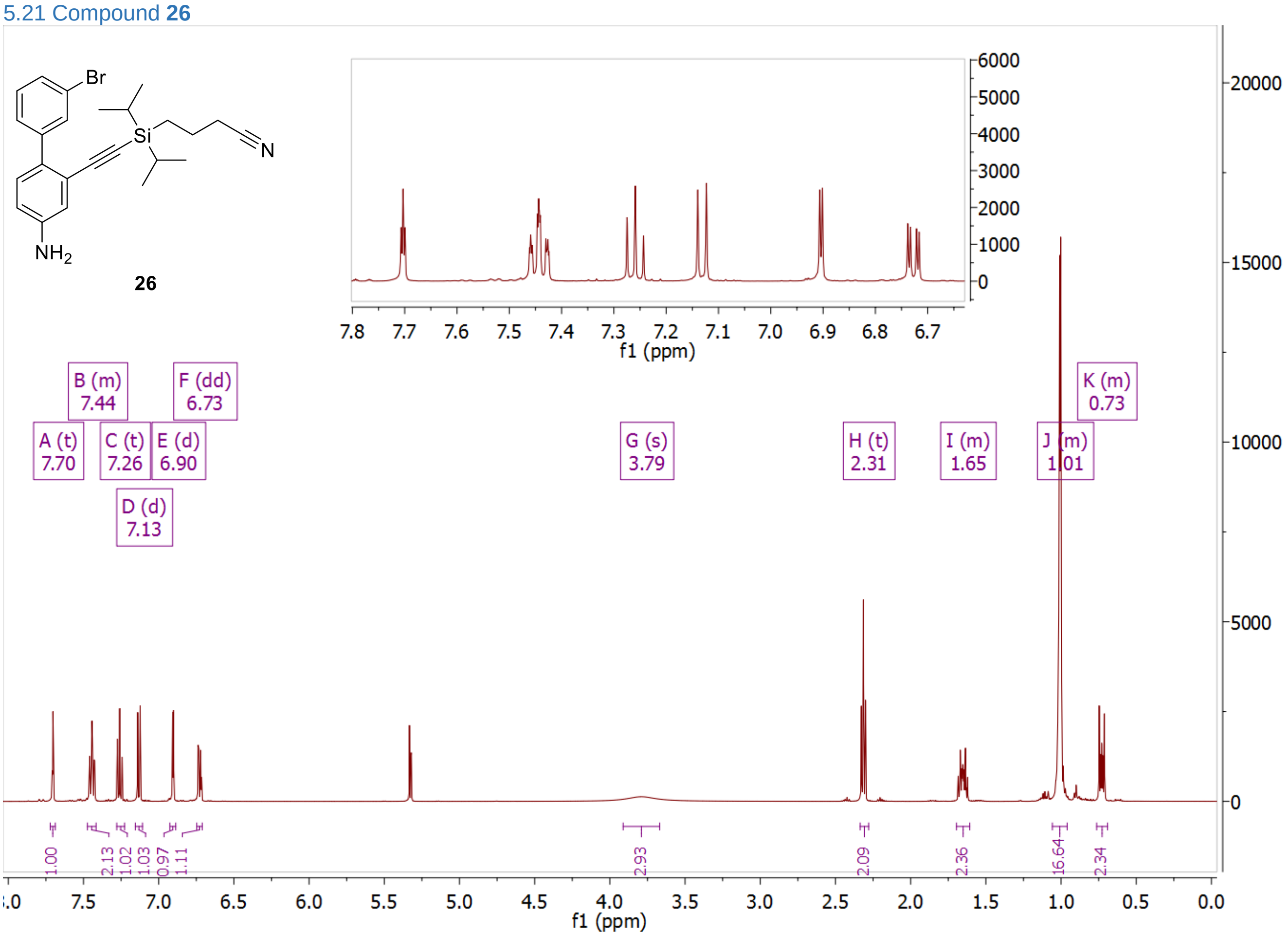

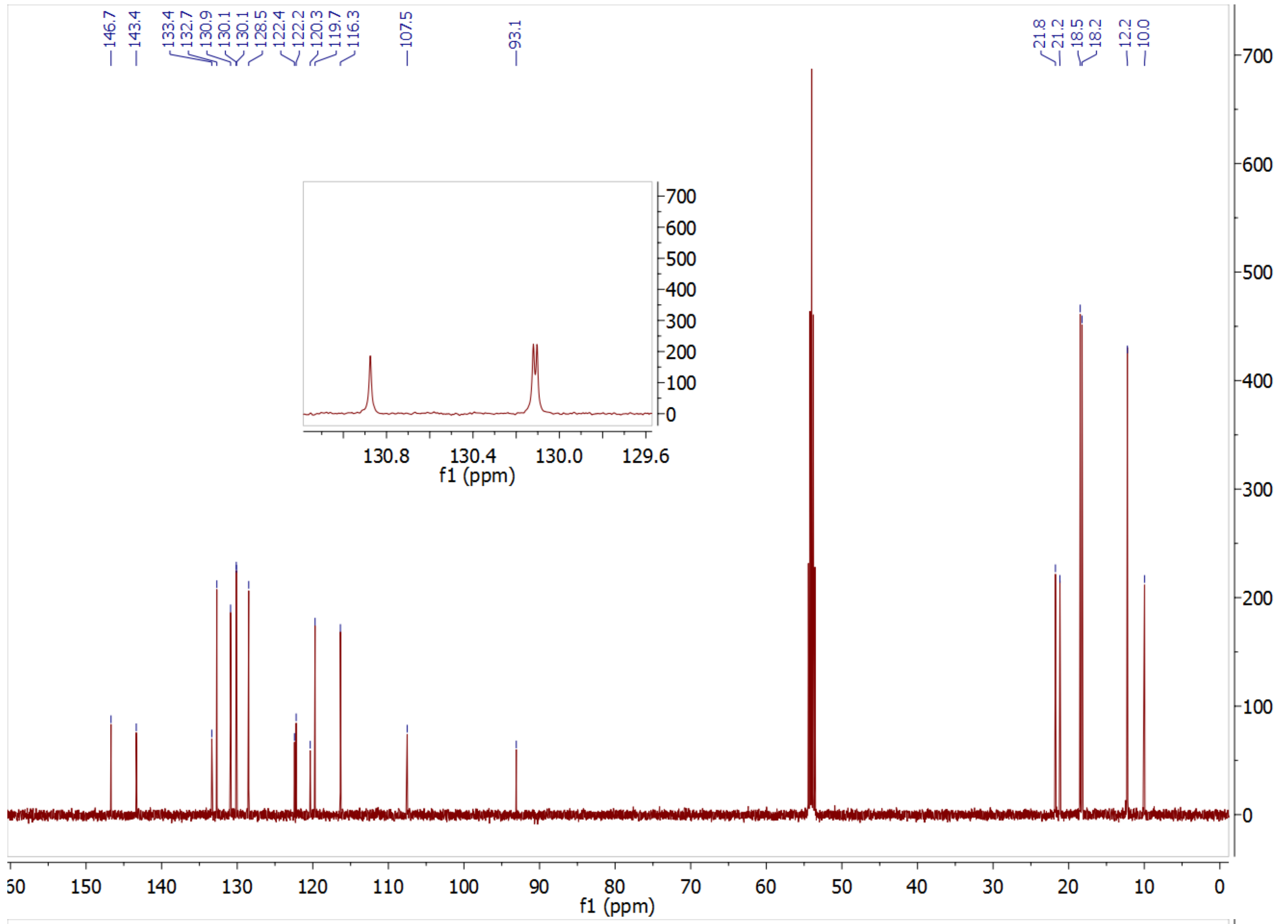
146.7
143.4
133.4
132.7
130.9
130.1
130.1
128.5
122.4
122.2
120.3
119.7
116.3
107.5
93.1
21.8
21.2
18.5
18.2
12.2
10.0
f1 (ppm)

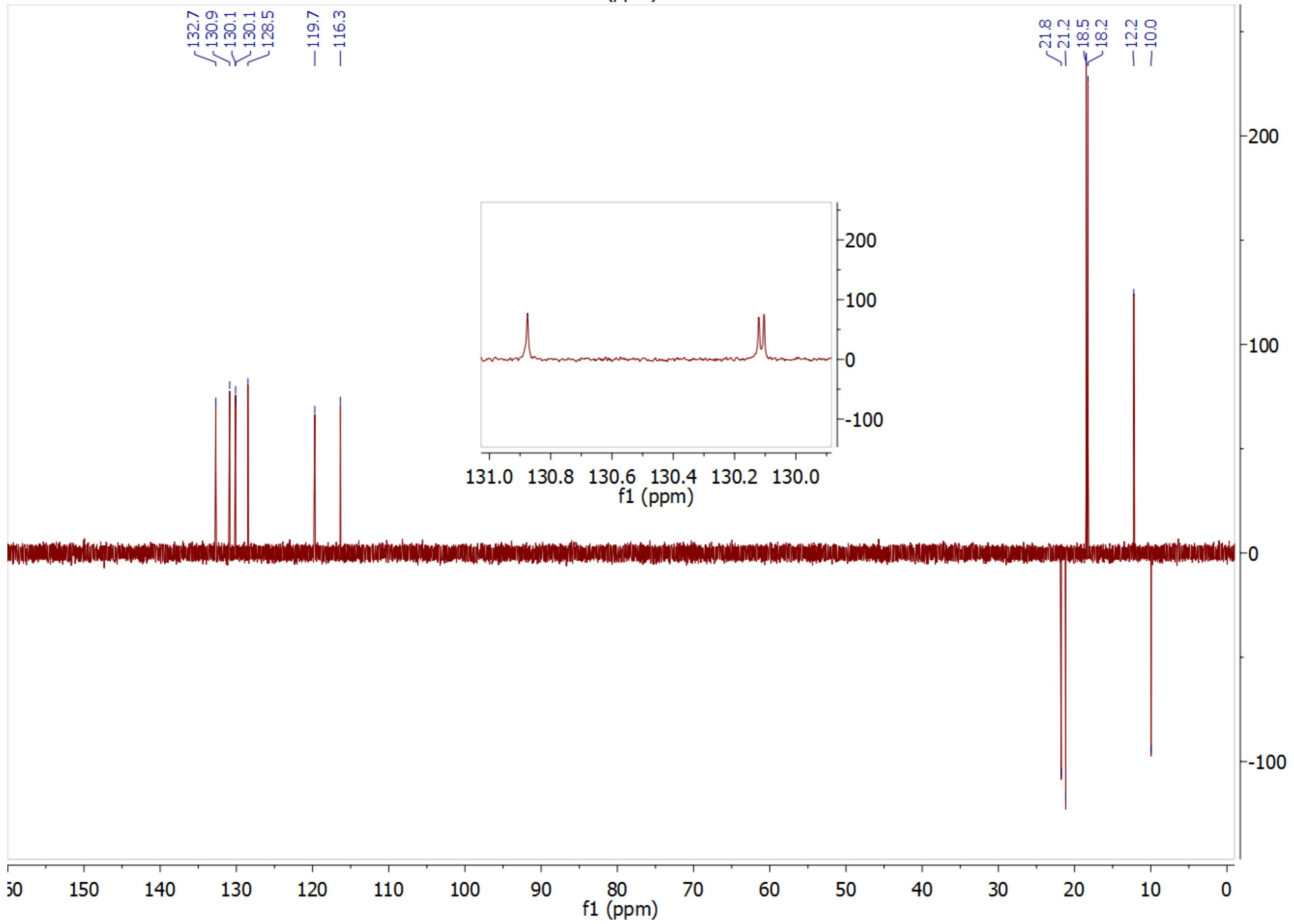
132.7
130.9
130.1
130.1
128.5
119.7
116.3
21.8
21.2
18.5
18.2
12.2
10.0
f1 (ppm)

High Resolution Mass Spectrometry Report

Sample Name	**Linda Bannwart / Ba521 chr1#2**	Instrument	maXis 4G
Comment	1.2 ug/mL in MeOH, analyzed in MeOH	Method	22 Direct_pos_mid.m

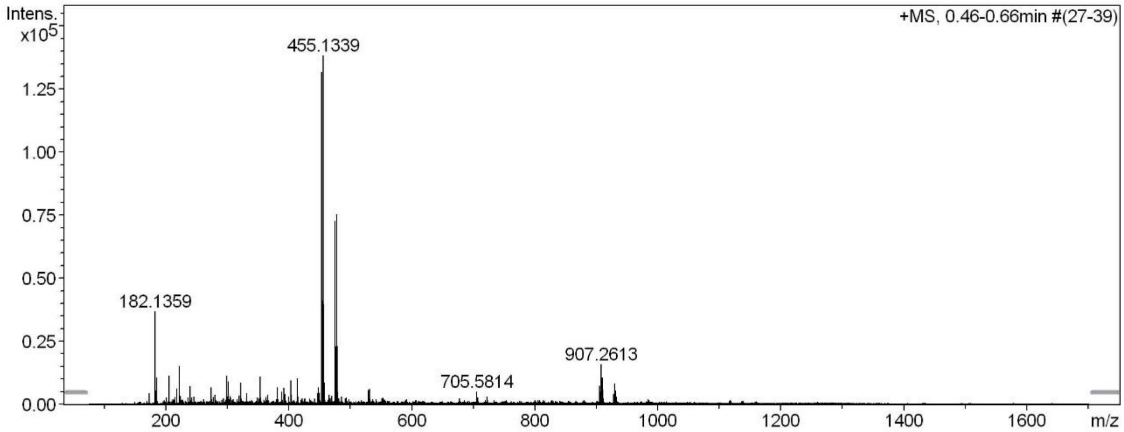

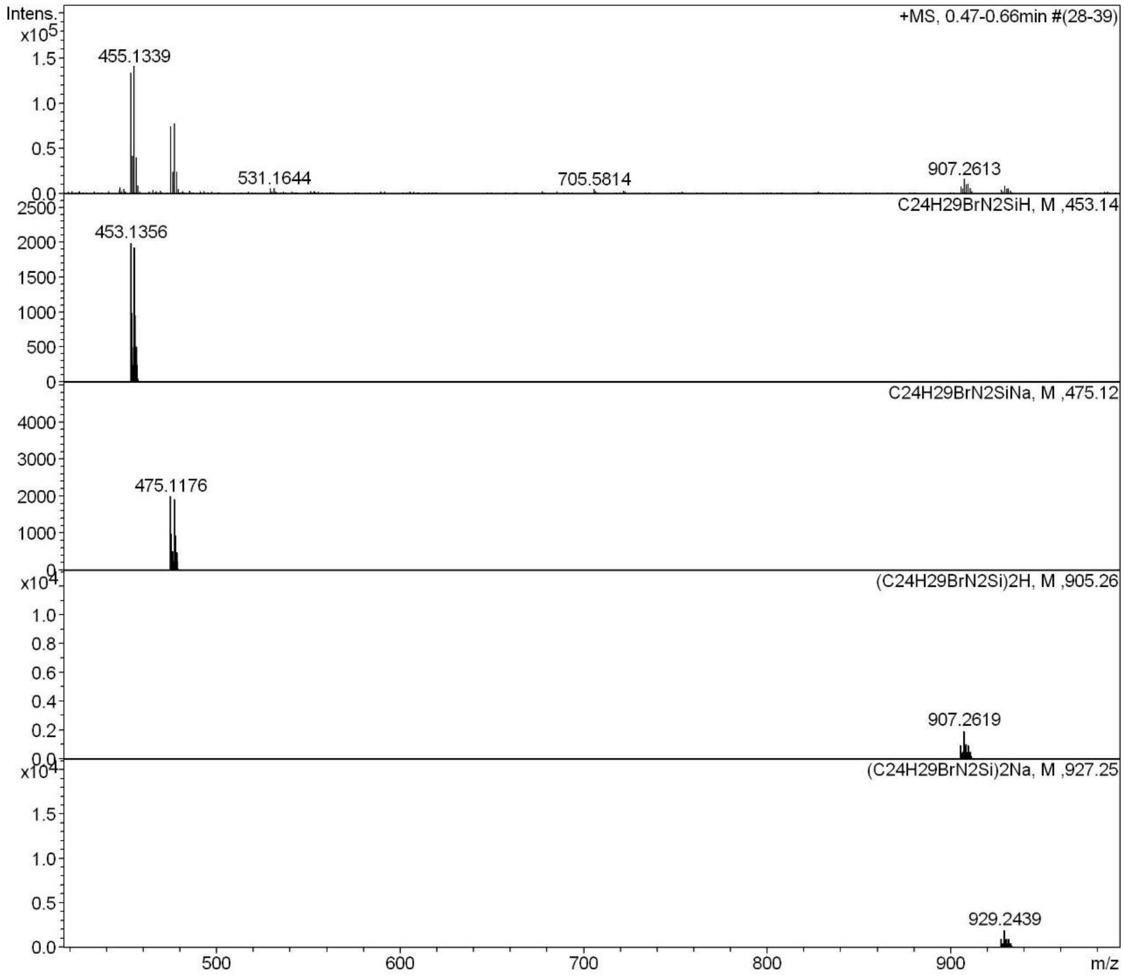

High Resolution Mass Spectrometry Report

Measured m/z vs. theoretical m/z

Meas. m/z	#	Formula	Score	m/z	err [mDa]	err [ppm]	mSigma	rdb	e⁻ Conf	z
453.1356	1	C 24 H 30 Br N 2 Si	100.00	453.1356	-0.0	-0.0	9.7	11.5	even	1+
475.1174	1	C 24 H 29 Br N 2 Na Si	100.00	475.1176	0.2	0.4	7.0	11.5	even	
905.2629	1	C 48 H 59 Br 2 N 4 Si 2	100.00	905.2640	1.1	1.2	18.3	22.5	even	
927.2444	1	C 48 H 58 Br 2 N 4 Na Si 2	100.00	927.2459	1.5	1.7	33.7	22.5	even	

Mass list

#	m/z	I %	I
1	173.0787	3.0	4178
2	182.1359	26.8	37638
3	183.0780	2.9	4139
4	183.1381	3.9	5529
5	185.1149	7.4	10354
6	201.1030	1.8	2528
7	205.0598	8.1	11388
8	215.1251	1.9	2690
9	217.1047	4.3	6104
10	222.1284	11.1	15567
11	223.1305	2.3	3292
12	236.0712	1.9	2633
13	239.0886	5.0	7085
14	242.2843	1.8	2518
15	245.0783	2.1	2937
16	273.1669	4.6	6435
17	277.2133	2.0	2744
18	279.1585	2.0	2800
19	279.2293	2.7	3776
20	293.2082	1.7	2388
21	299.1932	8.1	11403
22	300.1958	2.1	2989
23	301.1406	6.3	8893
24	301.2101	1.5	2137
25	304.2610	2.3	3197
26	305.2445	1.9	2617
27	315.1906	1.4	2000
28	319.2235	2.5	3476
29	321.1752	6.3	8824
30	321.2394	1.6	2217
31	322.1776	1.6	2286
32	331.1877	1.8	2559
33	331.2087	3.0	4150
34	348.9898	1.7	2447
35	350.9872	1.6	2227
36	353.2659	7.8	10985
37	354.2688	1.9	2644
38	363.1564	2.0	2822
39	365.1046	2.5	3582
40	365.2675	1.5	2098
41	381.1668	2.5	3502
42	381.2350	2.5	3463
43	381.2970	4.8	6706
44	388.2065	3.5	4933
45	391.2836	4.6	6436
46	393.2971	2.8	3962
47	399.1774	2.1	2969
48	403.2174	6.9	9646
49	404.2196	2.6	3651
50	413.2658	7.2	10181
51	414.2690	2.1	2945
52	421.3284	1.7	2451
53	425.3629	1.6	2199
54	433.3800	1.5	2060
55	441.2975	1.5	2174
56	447.0720	3.0	4279
57	447.3456	4.7	6578
58	448.3489	1.6	2221
59	449.3740	3.3	4600

High Resolution Mass Spectrometry Report

#	m/z	I %	I
60	453.1356	95.2	133734
61	454.1382	29.8	41834
62	455.1339	100.0	140537
63	456.1361	28.5	40050
64	457.1357	6.2	8770
65	465.3700	2.6	3694
66	467.1012	1.7	2342
67	469.3277	2.2	3108
68	475.1174	53.1	74588
69	476.1201	16.8	23563
70	477.1157	55.3	77676
71	478.1178	16.9	23769
72	479.1179	3.6	5024
73	481.3636	1.6	2234
74	485.3289	2.1	2999
75	491.0910	1.7	2342
76	493.0885	1.9	2627
77	529.1663	4.1	5795
78	530.1690	1.7	2396
79	531.1644	4.3	6078
80	532.1675	1.5	2066
81	541.1207	1.4	2023
82	551.1485	1.7	2438
83	553.1469	1.8	2579
84	677.3379	1.7	2374
85	705.5814	3.5	4945
86	706.5847	1.8	2555
87	721.5750	2.2	3034
88	905.2629	5.4	7549
89	906.2647	3.6	5089
90	907.2613	11.6	16251
91	908.2636	7.3	10212
92	909.2605	7.7	10810
93	910.2619	4.2	5877
94	911.2622	1.8	2500
95	927.2444	2.9	4143
96	928.2468	1.9	2615
97	929.2429	6.0	8465
98	930.2453	3.8	5366
99	931.2428	3.9	5527
100	932.2436	2.3	3184

Acquisition Parameter

Source Type	ESI	Ion Polarity	Positive	Set Nebulizer	0.4 Bar
Focus	Not active	Set Capillary	3600 V	Set Dry Heater	180 °C
Scan Begin	75 m/z	Set End Plate Offset	-500 V	Set Dry Gas	4.0 l/min
Scan End	1700 m/z	Set Collision Cell RF	350.0 Vpp	Set Ion Energy (MS only)	4.0 eV

5.22 Compound **27**

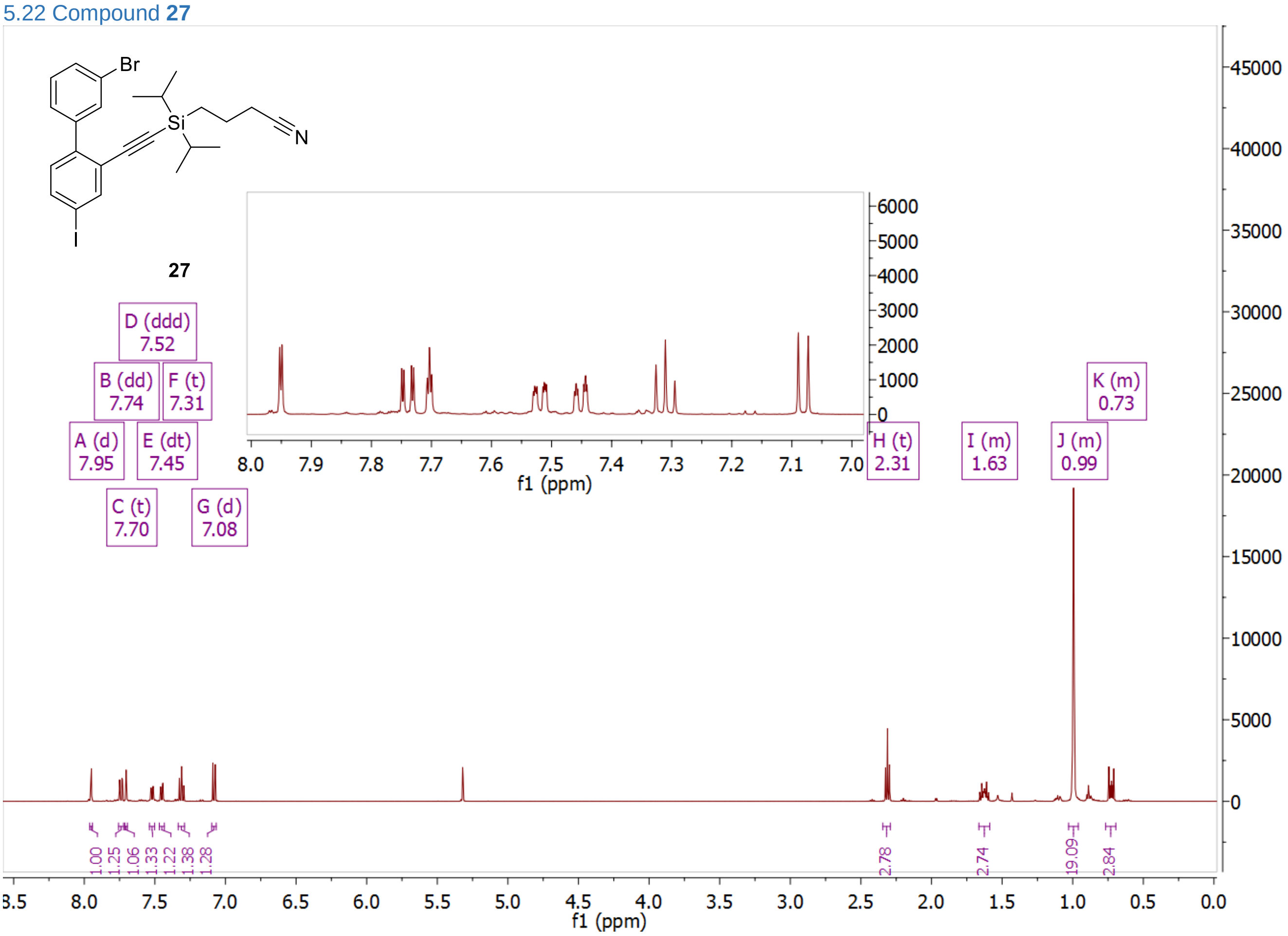

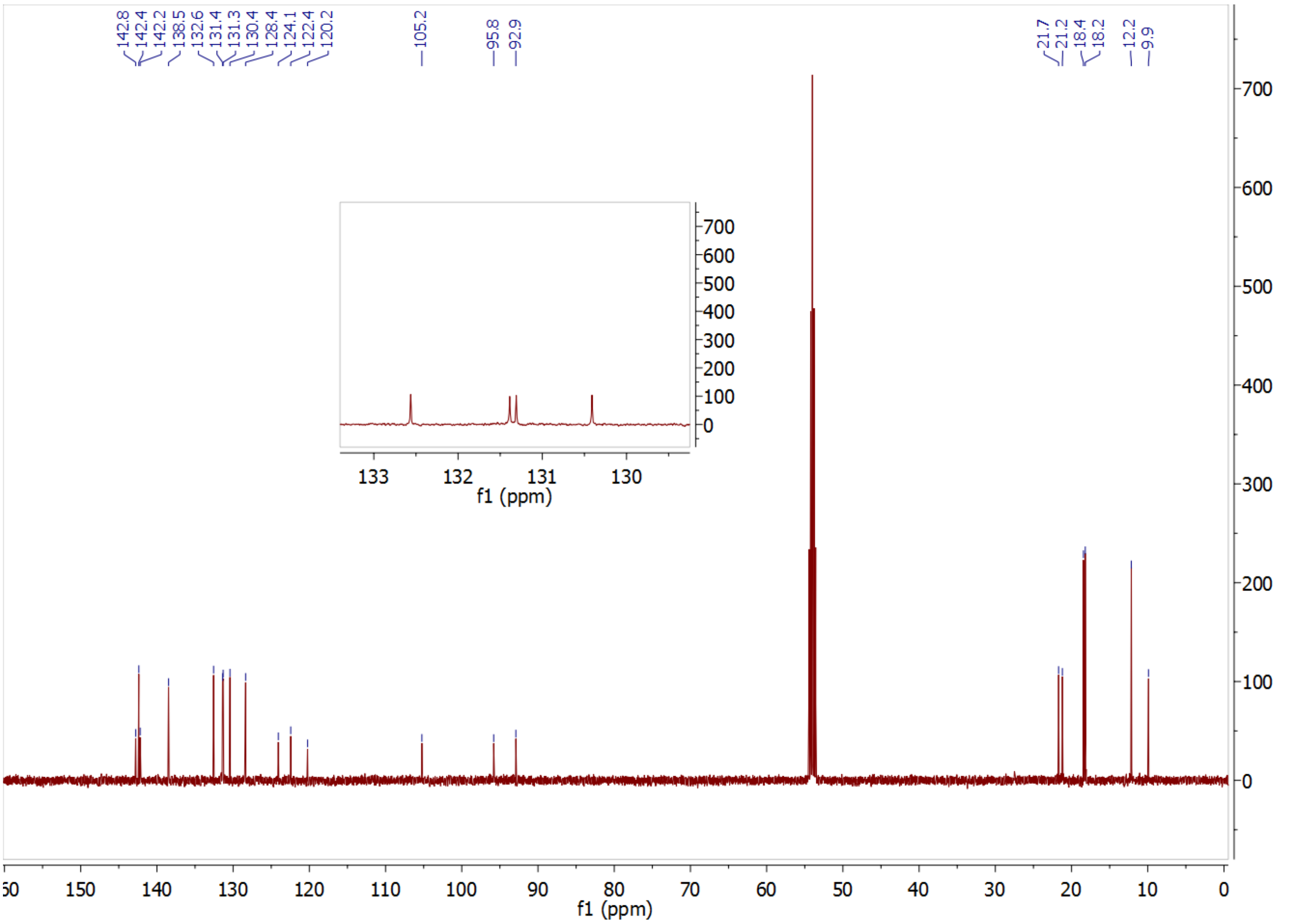

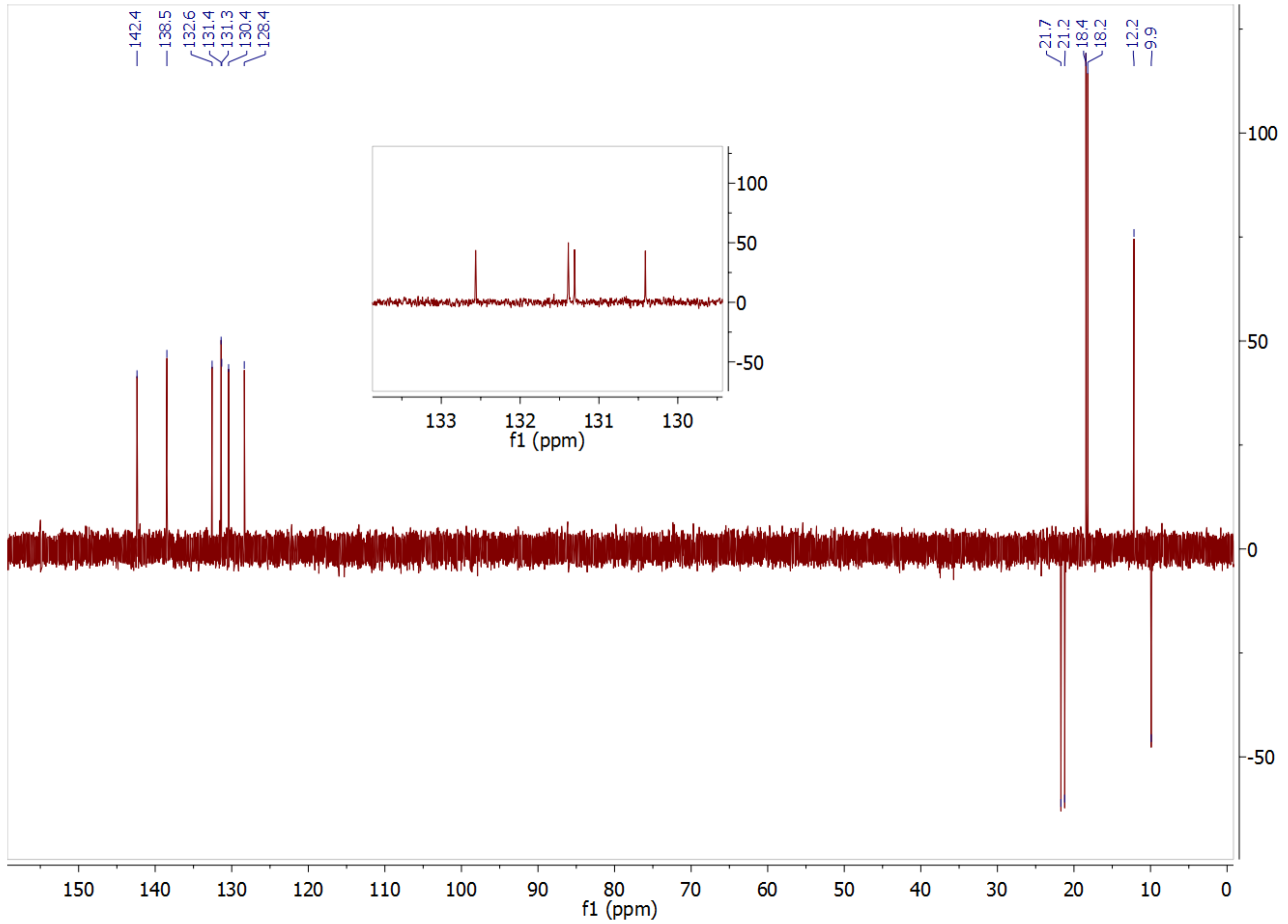
142.4
138.5
132.6
131.4
131.3
130.4
128.4
21.7
21.2
18.4
18.2
12.2
9.9
f1 (ppm)

High Resolution Mass Spectrometry Report

Sample Name	**Ba599 chr1#1**	Instrument	maXis 4G
Comment		Method	ms_nocolumn_300-600_pos.m

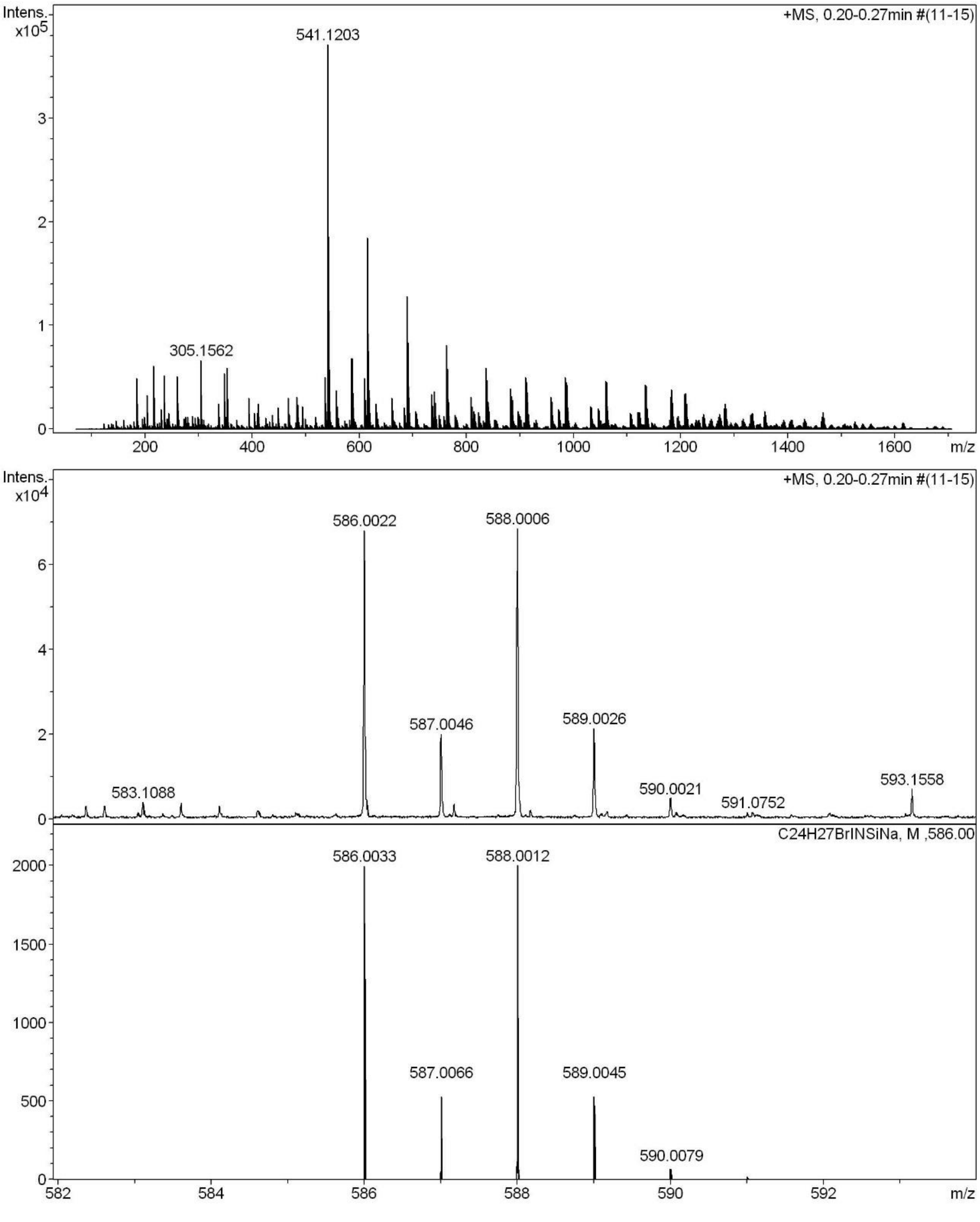

High Resolution Mass Spectrometry Report

Measured m/z vs. theoretical m/z

Meas. m/z	#	Formula	Score	m/z	err [mDa]	err [ppm]	mSigma	rdb	e⁻ Conf	z
586.0022	1	C 24 H 27 Br I N Na Si	100.00	586.0033	1.1	1.8	19.0	11.5	even	1+

Mass list

#	m/z	I %	I
1	185.1145	13.3	49468
2	205.0593	8.7	32510
3	217.0828	16.5	61443
4	217.1038	7.1	26444
5	236.0709	14.0	52044
6	261.1300	13.8	51141
7	305.1562	17.9	66691
8	337.0737	6.7	24984
9	349.1824	14.6	54147
10	353.1444	15.9	59044
11	393.2084	8.1	30174
12	411.0926	6.6	24555
13	449.2861	5.6	20804
14	467.1008	8.2	30539
15	483.1320	8.3	31014
16	485.1116	6.4	23743
17	493.3122	5.8	21681
18	536.1642	13.4	49798
19	537.1646	6.8	25280
20	541.1203	100.0	371788
21	542.1208	44.0	163425
22	543.1180	30.8	114523
23	544.1175	10.7	39918
24	557.1508	10.1	37560
25	558.1515	5.2	19508
26	559.1324	5.9	22068
27	586.0022	18.3	68067
28	587.0046	5.4	20205
29	588.0006	18.5	68613
30	589.0026	5.8	21518
31	610.1829	13.3	49327
32	611.1833	7.6	28096
33	612.1814	5.8	21731
34	615.1388	49.7	184648
35	616.1393	28.1	104608
36	617.1363	20.3	75651
37	618.1358	8.3	30756
38	631.1693	6.6	24669
39	661.1935	8.2	30388
40	684.2011	5.6	21002
41	689.1571	34.5	128332
42	690.1577	22.5	83523
43	691.1547	17.7	65850
44	692.1538	8.3	30701
45	735.2118	9.1	33918
46	736.2123	6.2	22879
47	740.2636	9.9	36745
48	741.2644	6.9	25560
49	742.2616	5.4	19911
50	763.1753	21.9	81496
51	764.1760	15.7	58554
52	765.1731	13.2	49141
53	766.1718	7.1	26567
54	809.2302	8.3	30968
55	810.2311	6.0	22299
56	837.1935	16.0	59626
57	838.1947	12.8	47453
58	839.1896	11.9	44215
59	840.1858	7.1	26291
60	883.2485	10.5	39068
61	884.2492	8.6	31908
62	885.2475	7.5	28001

#	m/z	I %	I
63	911.2118	13.5	50144
64	912.2126	12.2	45281
65	913.2097	11.0	40882
66	914.2080	6.8	25141
67	957.2667	8.3	30877
68	958.2675	7.8	28823
69	959.2655	7.0	26185
70	971.2819	5.3	19710
71	985.2303	13.5	50063
72	986.2308	12.3	45597
73	987.2285	11.6	43230
74	988.2271	7.1	26393
75	1031.2847	5.8	21560
76	1032.2854	5.9	21869
77	1033.2839	5.6	20719
78	1045.3002	5.4	20166
79	1046.3008	5.1	19108
80	1059.2482	12.4	46227
81	1060.2489	12.4	46101
82	1061.2472	12.4	46000
83	1062.2459	8.0	29925
84	1133.2661	10.4	38612
85	1134.2670	11.5	42733
86	1135.2651	11.4	42333
87	1136.2643	7.8	29046
88	1137.2626	5.3	19847
89	1181.1596	8.4	31338
90	1182.1604	9.8	36486
91	1183.1585	10.2	38033
92	1184.1582	6.9	25481
93	1207.2837	8.2	30374
94	1208.2853	9.2	34147
95	1209.2836	9.4	34887
96	1210.2833	6.7	25052
97	1281.3014	5.4	20131
98	1282.3025	6.3	23505
99	1283.3009	6.8	25126
100	1284.2996	5.3	19826

Acquisition Parameter

**General**	Fore Vacuum	2.59e+000 mBar	High Vacuum	1.26e-007 mBar	Source Type	ESI
	Scan Begin	75 m/z	Scan End	1700 m/z	Ion Polarity	Positive
**Source**	Set Nebulizer	2.0 Bar	Set Capillary	4500 V	Set Dry Gas	8.0 l/min
	Set Dry Heater	200 °C	Set End Plate Offset	-500 V		
**Quadrupole**	Set Ion Energy (MS only)	4.0 eV				
**Coll. Cell**	Collision Energy	8.0 eV	Set Collision Cell RF	350.0 Vpp		
**Ion Cooler**	Set Ion Cooler Transfer Time	75.0 µs	Set Ion Cooler Pre Pulse Storage Time	10.0 µs		

5.23 Compound **28**

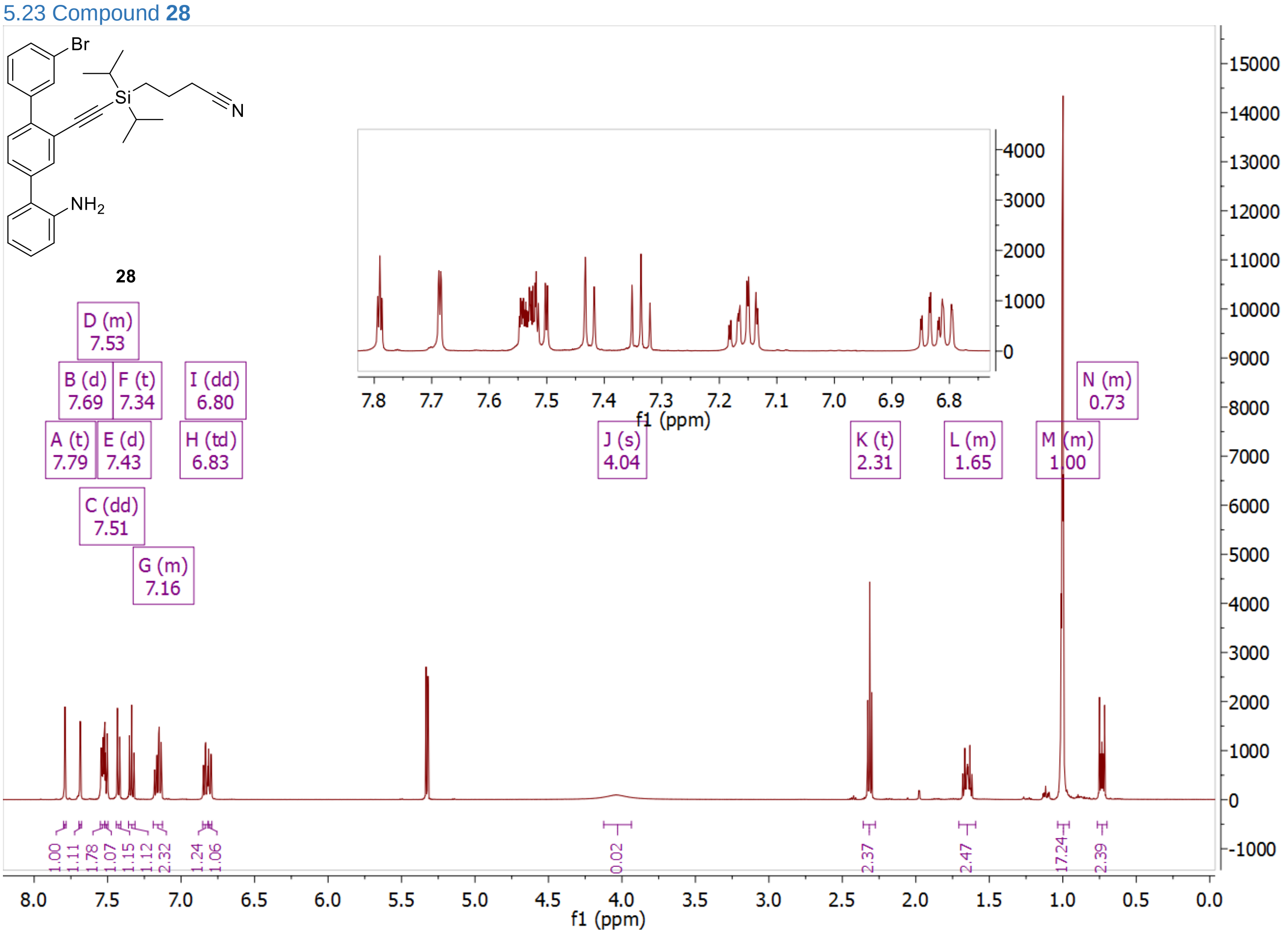

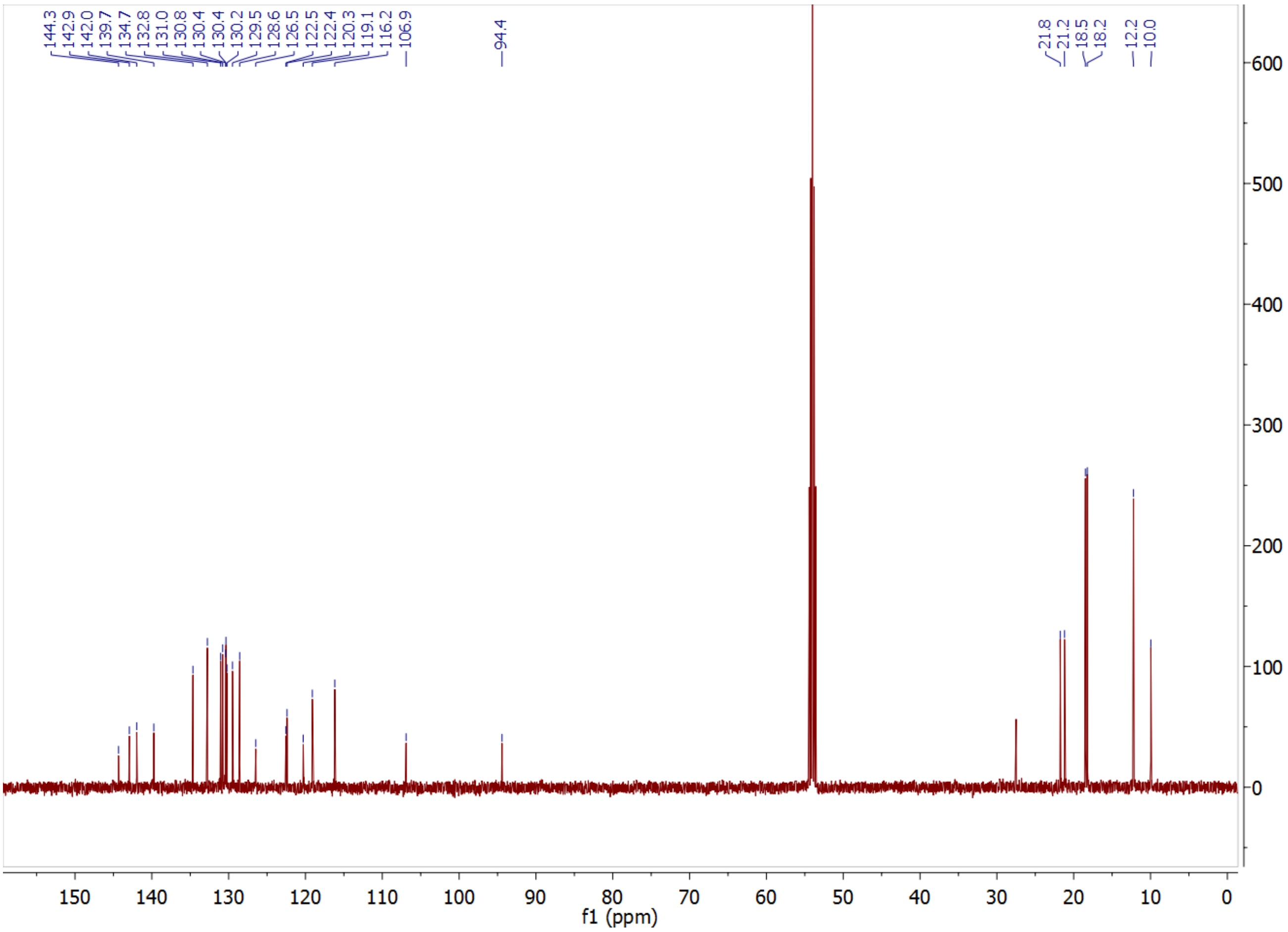
144.3
142.9
142.0
139.7
134.7
132.8
131.0
130.8
130.4
130.4
130.2
129.5
128.6
126.5
122.5
122.4
120.3
119.1
116.2
106.9
94.4
21.8
21.2
18.5
18.2
12.2
10.0
f1 (ppm)

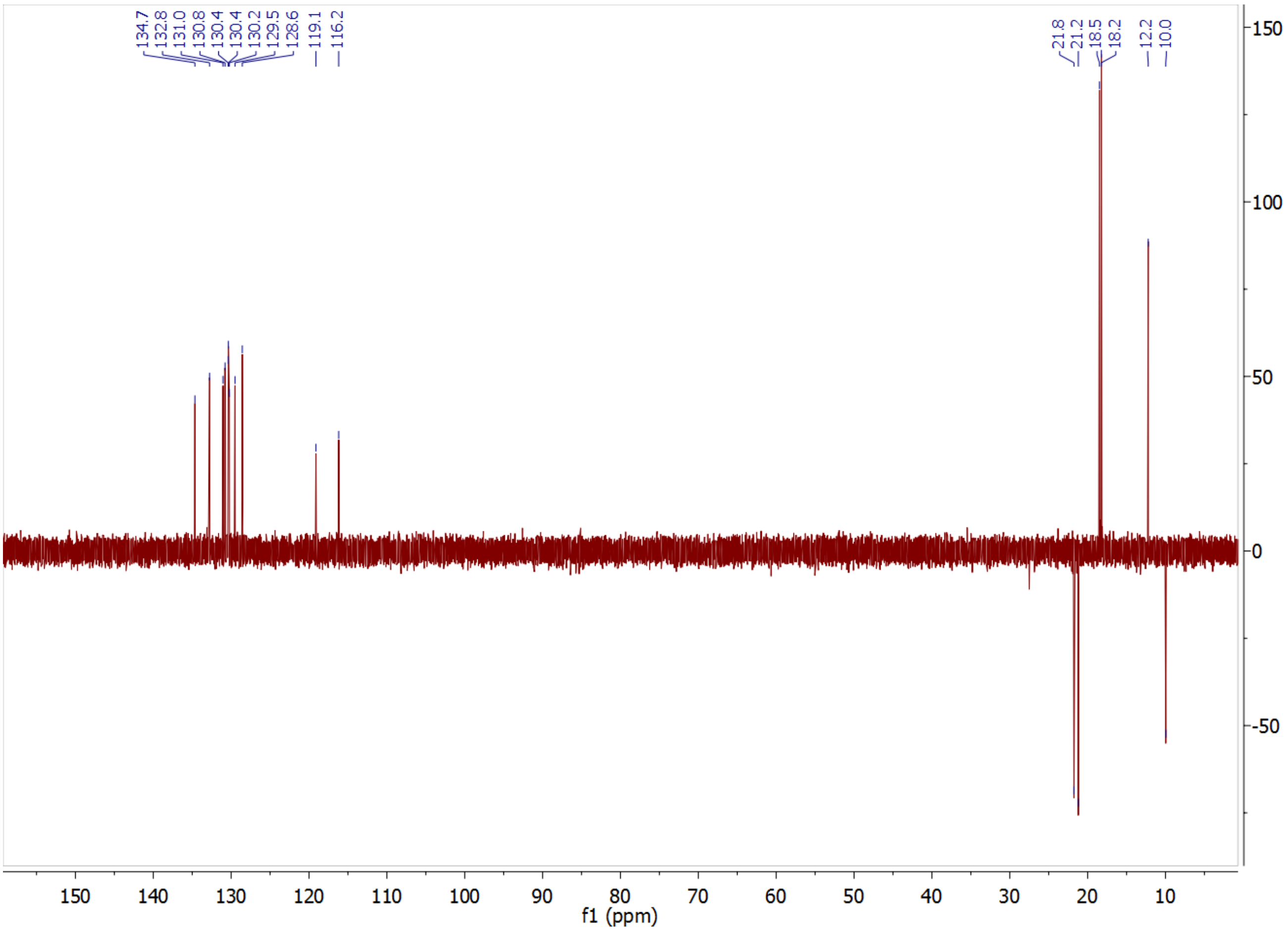
134.7
132.8
131.0
130.8
130.4
130.4
130.2
129.5
128.6
119.1
116.2
21.8
21.2
18.5
18.2
12.2
10.0
f1 (ppm)

High Resolution Mass Spectrometry Report

Sample Name	**Fäseke / FAV573**	Instrument	maXis 4G
Comment	10 ug/mL in MeOH, analyzed in MeOH	Method	22 Direct_pos_mid.m

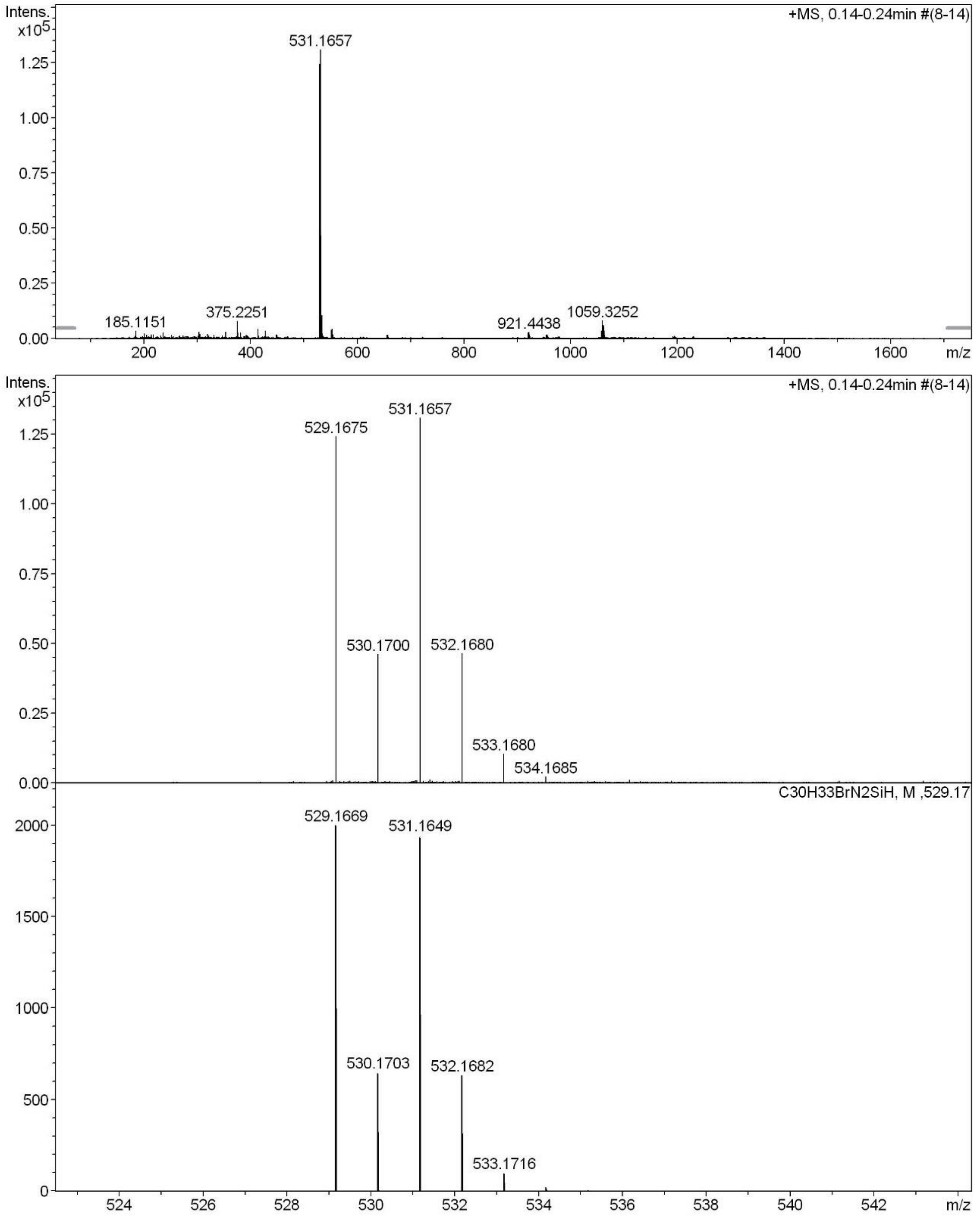

High Resolution Mass Spectrometry Report

Measured m/z vs. theoretical m/z

Meas. m/z	#	Formula	Score	m/z	err [mDa]	err [ppm]	mSigma	rdb	e⁻ Conf	z
529.1675	1	C 30 H 34 Br N 2 Si	100.00	529.1669	-0.5	-1.0	12.6	15.5	even	1+

Mass list

#	m/z	I %	I
1	183.0779	0.6	824
2	183.0993	0.7	972
3	185.1151	2.6	3419
4	197.1151	0.6	744
5	201.1026	1.7	2177
6	205.0604	1.2	1546
7	208.9491	0.7	883
8	214.0895	1.3	1650
9	217.1049	1.4	1781
10	225.1094	0.6	778
11	231.1810	0.9	1175
12	236.0715	1.9	2544
13	239.0891	0.6	811
14	251.1615	1.1	1476
15	255.1820	0.6	816
16	265.1772	0.7	913
17	267.1573	1.0	1355
18	273.1676	0.9	1154
19	273.1913	1.0	1370
20	277.2142	0.6	845
21	279.1580	0.7	951
22	279.2292	0.6	848
23	281.1736	0.6	773
24	283.1508	0.6	841
25	293.2084	0.6	772
26	295.1932	0.6	846
27	301.1409	0.8	1078
28	303.1778	2.3	3024
29	305.2083	1.5	1966
30	305.2448	0.6	761
31	315.1932	0.6	833
32	319.2241	1.5	1930
33	321.1357	0.9	1221
34	321.2046	0.6	809
35	331.2097	1.2	1556
36	347.2555	1.0	1333
37	353.1458	0.9	1196
38	353.2300	0.6	745
39	353.2664	2.3	3039
40	354.2702	0.6	731
41	373.2708	0.6	757
42	375.2251	6.0	7794
43	376.2280	1.9	2492
44	381.2973	2.0	2587
45	387.2863	0.6	847
46	389.2557	0.7	869
47	391.2835	1.2	1506
48	393.2973	1.0	1296
49	395.1806	0.6	824
50	413.2659	3.3	4255
51	414.2695	0.9	1193
52	422.2539	0.6	779
53	425.3629	0.6	814
54	427.2092	2.6	3432
55	428.2123	0.7	912
56	433.1031	0.6	790
57	447.3445	1.3	1731
58	448.3480	0.6	764
59	449.3741	1.1	1384
60	469.3295	0.6	774
61	529.0851	0.7	890
62	529.1675	94.9	124060

High Resolution Mass Spectrometry Report

#	m/z	I %	I
63	530.1700	35.2	46049
64	531.0592	0.6	835
65	531.0915	0.7	864
66	531.1657	100.0	130749
67	531.4047	0.9	1200
68	532.1680	35.5	46422
69	533.1680	7.9	10347
70	534.1685	1.6	2130
71	535.1731	0.6	747
72	536.1647	0.7	980
73	551.1487	2.8	3723
74	552.1523	1.2	1614
75	553.1471	3.2	4141
76	554.1495	1.3	1658
77	610.1840	0.6	735
78	655.0631	1.2	1550
79	657.0621	1.1	1468
80	919.4437	1.9	2490
81	920.4463	1.3	1650
82	921.4438	2.2	2831
83	922.4455	1.3	1713
84	923.4475	0.6	829
85	953.5227	1.1	1468
86	954.5259	0.7	956
87	955.5237	1.2	1586
88	956.5268	0.8	1014
89	977.5056	0.7	920
90	1057.3267	2.7	3474
91	1058.3306	2.2	2926
92	1059.3252	6.1	7988
93	1060.3287	4.4	5817
94	1061.3248	4.2	5533
95	1062.3262	2.6	3343
96	1063.3271	1.2	1533
97	1191.6151	0.7	863
98	1193.6135	0.9	1115
99	1194.6168	0.6	785
100	1229.7577	0.6	849

Acquisition Parameter

Source Type	ESI	Ion Polarity	Positive	Set Nebulizer	0.4 Bar
Focus	Not active	Set Capillary	3600 V	Set Dry Heater	180 °C
Scan Begin	75 m/z	Set End Plate Offset	-500 V	Set Dry Gas	4.0 l/min
Scan End	1700 m/z	Set Collision Cell RF	350.0 Vpp	Set Ion Energy (MS only)	4.0 eV

5.24 Compound **29**

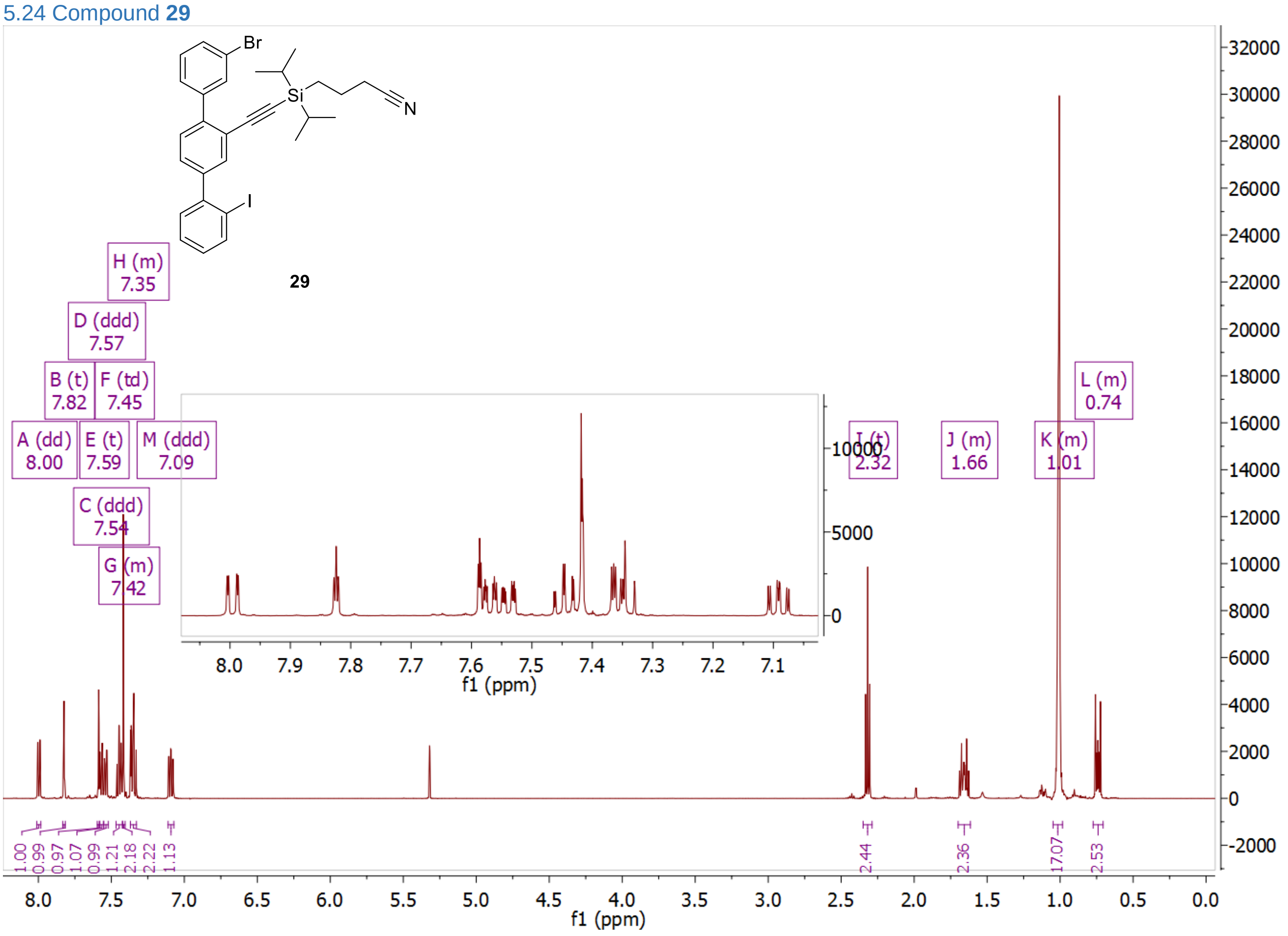

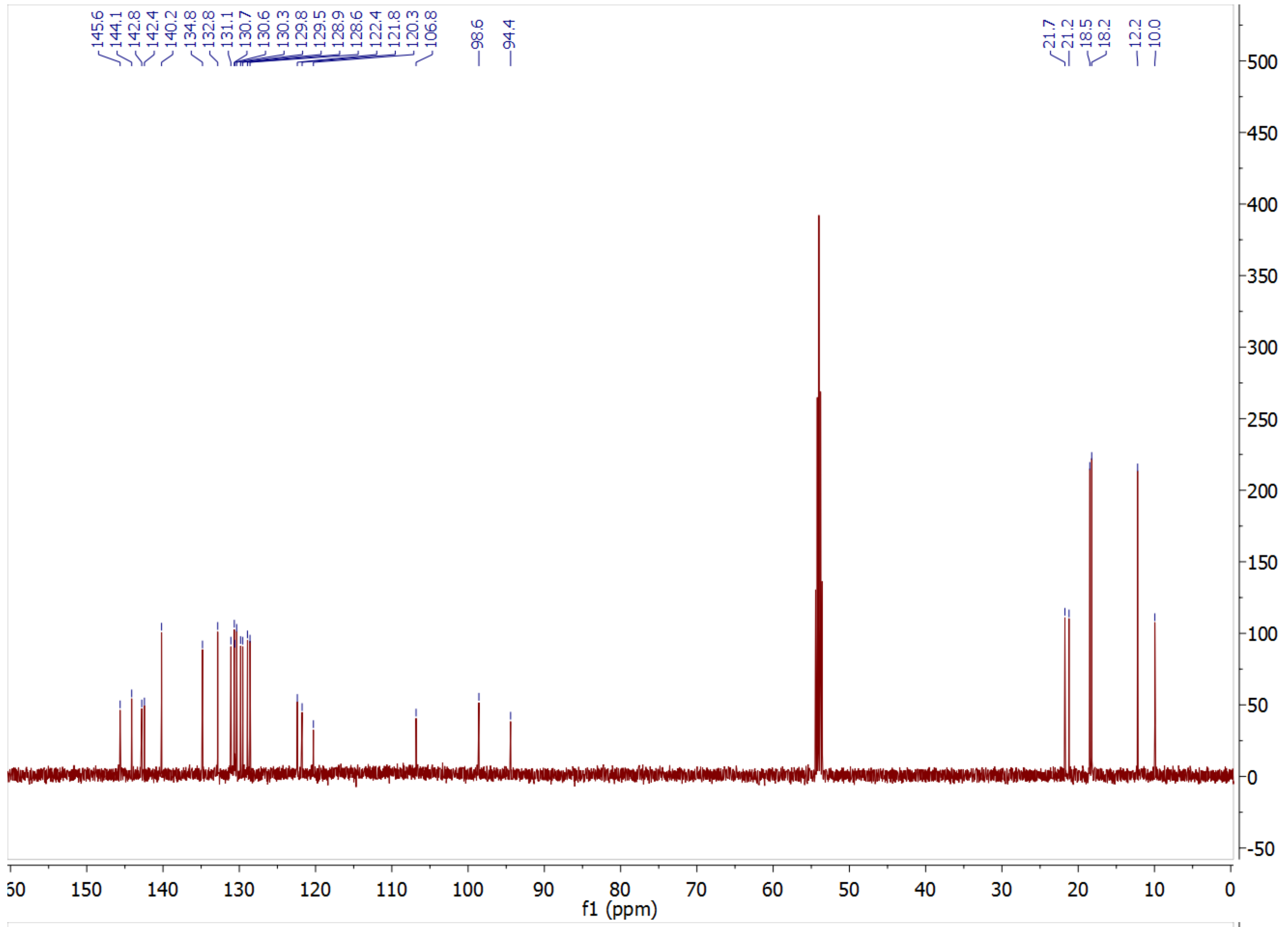
145.6
144.1
142.8
142.4
140.2
134.8
132.8
131.1
130.7
130.6
130.3
129.8
129.5
128.9
128.6
122.4
121.8
120.3
106.8
98.6
94.4
21.7
21.2
18.5
18.2
12.2
10.0
f1 (ppm)

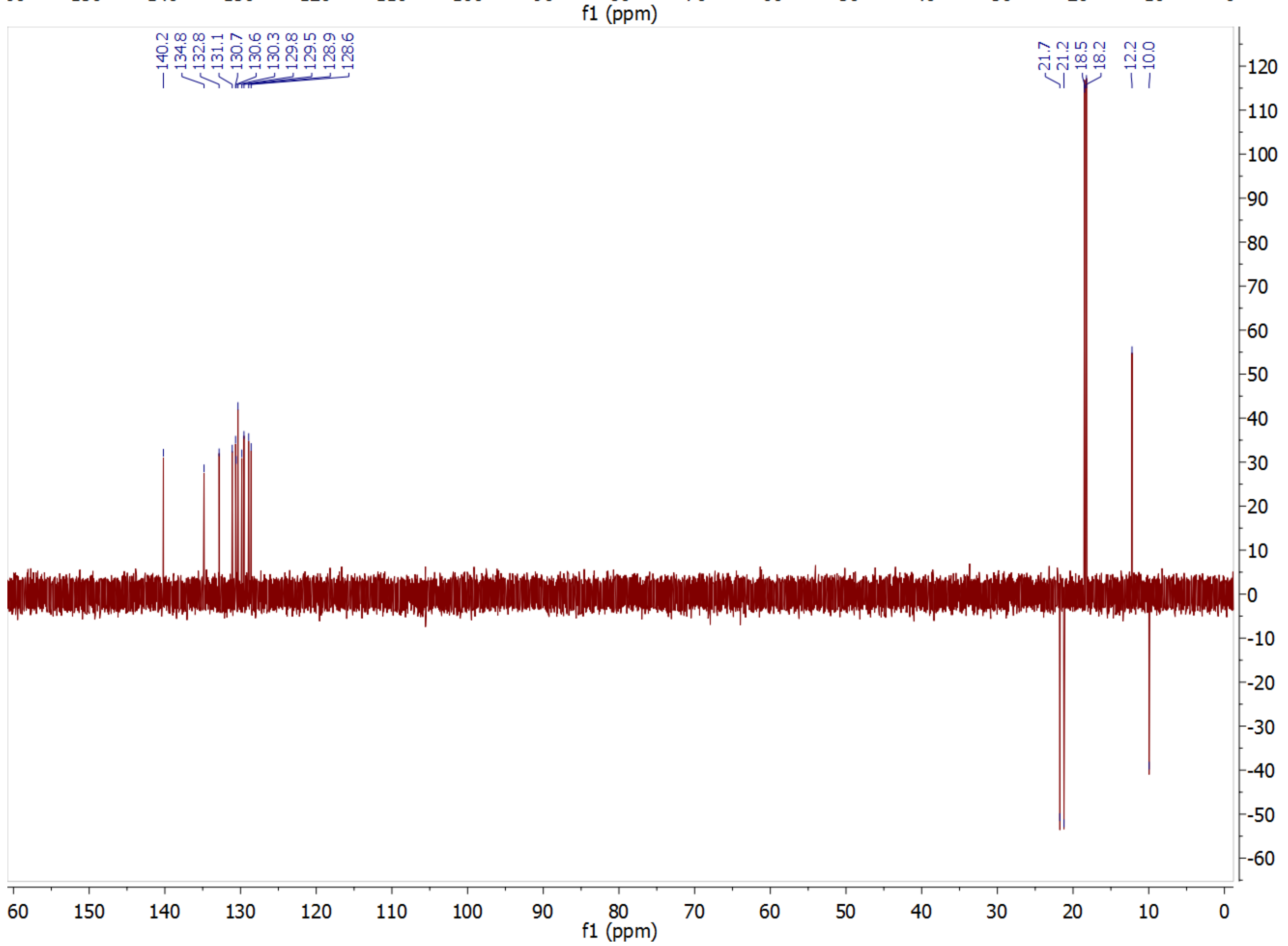
140.2
134.8
132.8
131.1
130.7
130.6
130.3
129.8
129.5
128.9
128.6
21.7
21.2
18.5
18.2
12.2
10.0
f1 (ppm)

High Resolution Mass Spectrometry Report

Sample Name	**Linda Bannwart / Ba561**	Instrument	maXis 4G
Comment	10 ug/mL in MeOH, analyzed in MeOH	Method	22 Direct_pos_mid.m

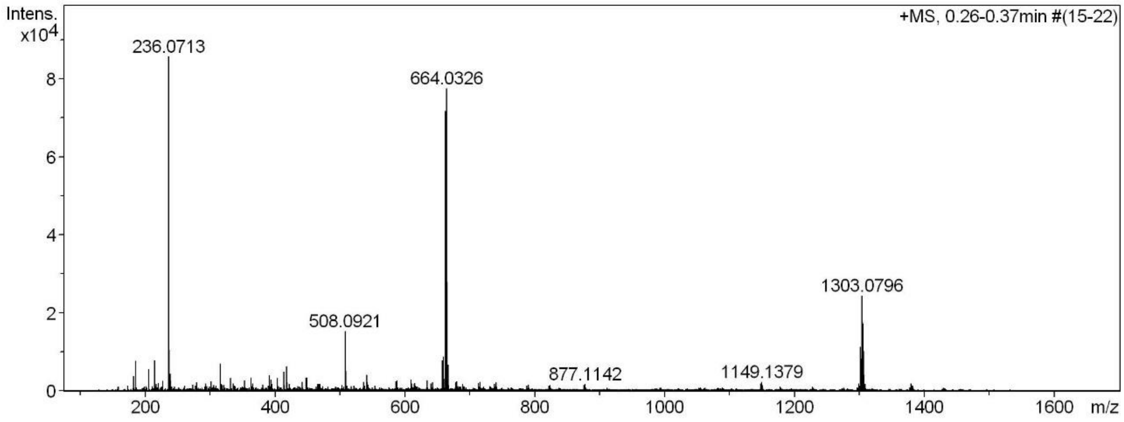

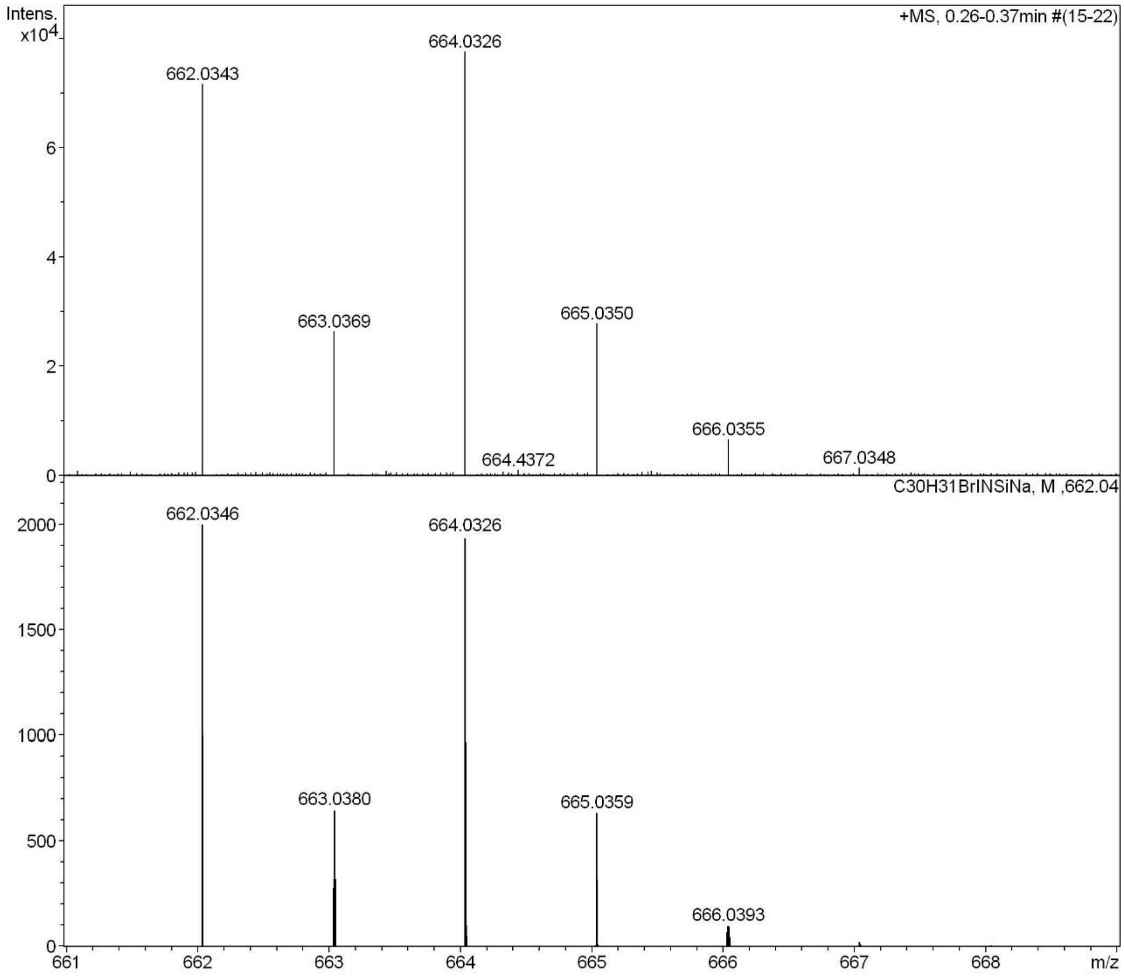

High Resolution Mass Spectrometry Report

Measured m/z vs. theoretical m/z

Meas. m/z	#	Formula	Score	m/z	err [mDa]	err [ppm]	mSigma	rdb	e⁻ Conf	z
662.0343	1	C 30 H 31 Br I N Na Si	100.00	662.0346	0.3	0.5	8.0	15.5	even	1+

Mass list

#	m/z	I %	I
1	182.1359	4.4	3766
2	185.1145	8.9	7637
3	205.0597	6.5	5576
4	214.0894	9.0	7679
5	217.1043	1.9	1597
6	220.1671	2.3	1978
7	226.9511	3.0	2542
8	236.0713	100.0	85510
9	237.0742	12.1	10326
10	238.0675	5.1	4321
11	239.0884	3.0	2599
12	273.1449	1.8	1509
13	277.2133	1.5	1303
14	279.2289	2.4	2057
15	293.2446	2.1	1831
16	301.1401	2.8	2395
17	301.2107	1.8	1525
18	305.2441	1.6	1353
19	315.1923	8.2	6972
20	316.1952	1.6	1356
21	317.1720	1.9	1663
22	319.2242	1.6	1342
23	321.2759	1.7	1440
24	331.1871	3.8	3219
25	331.2063	1.6	1375
26	335.1360	2.2	1843
27	353.2656	3.1	2667
28	363.2243	3.9	3371
29	365.1049	2.0	1749
30	365.2677	1.9	1644
31	381.2343	1.8	1498
32	381.2961	1.6	1329
33	391.2085	4.6	3893
34	393.2969	3.2	2738
35	395.3624	1.9	1625
36	403.2172	3.7	3150
37	413.2659	5.7	4885
38	414.2695	1.7	1418
39	417.3446	7.2	6152
40	418.3477	2.4	2011
41	421.3274	1.9	1614
42	441.2971	2.6	2234
43	447.3435	3.9	3316
44	449.1541	3.9	3326
45	449.3729	2.6	2197
46	465.3700	2.0	1715
47	467.1018	2.0	1704
48	469.3264	1.9	1600
49	503.1375	1.6	1360
50	508.0921	17.9	15272
51	509.0945	5.8	4977
52	536.1637	2.6	2262
53	541.1197	4.7	4036
54	542.1209	2.4	2069
55	543.1186	1.7	1454
56	586.0022	2.7	2303
57	588.0009	3.1	2639
58	609.3392	3.2	2771
59	610.1837	2.1	1798
60	614.5224	2.0	1689
61	615.1384	2.3	1964
62	633.9886	3.1	2621

High Resolution Mass Spectrometry Report

#	m/z	I %	I
63	640.0520	2.0	1750
64	642.0501	2.4	2035
65	657.0788	9.1	7810
66	658.0813	3.7	3137
67	659.0767	10.2	8746
68	660.0808	3.6	3087
69	662.0343	83.7	71572
70	663.0369	30.8	26363
71	664.0326	90.6	77450
72	665.0350	32.5	27812
73	666.0355	7.7	6586
74	667.0348	1.6	1394
75	678.0085	2.4	2069
76	680.0059	2.8	2385
77	689.1583	2.0	1669
78	713.1400	2.3	1990
79	715.1394	2.6	2256
80	738.0654	2.0	1684
81	740.0624	2.4	2039
82	789.9282	1.8	1503
83	823.1802	1.6	1337
84	875.1137	1.6	1341
85	877.1142	1.9	1609
86	1147.1375	2.1	1813
87	1148.1416	1.6	1360
88	1149.1379	2.6	2242
89	1150.1394	1.7	1430
90	1298.1250	2.1	1764
91	1301.0807	13.0	11134
92	1302.0836	9.6	8169
93	1303.0796	28.5	24355
94	1304.0809	20.5	17546
95	1305.0788	20.0	17108
96	1306.0796	11.8	10056
97	1307.0830	4.8	4072
98	1308.0810	1.9	1626
99	1379.1083	2.2	1849
100	1380.1134	1.7	1421

Acquisition Parameter

Source Type	ESI	Ion Polarity	Positive	Set Nebulizer	0.4 Bar
Focus	Not active	Set Capillary	3600 V	Set Dry Heater	180 °C
Scan Begin	75 m/z	Set End Plate Offset	-500 V	Set Dry Gas	4.0 l/min
Scan End	1700 m/z	Set Collision Cell RF	350.0 Vpp	Set Ion Energy (MS only)	4.0 eV

5.25 Compound **31**

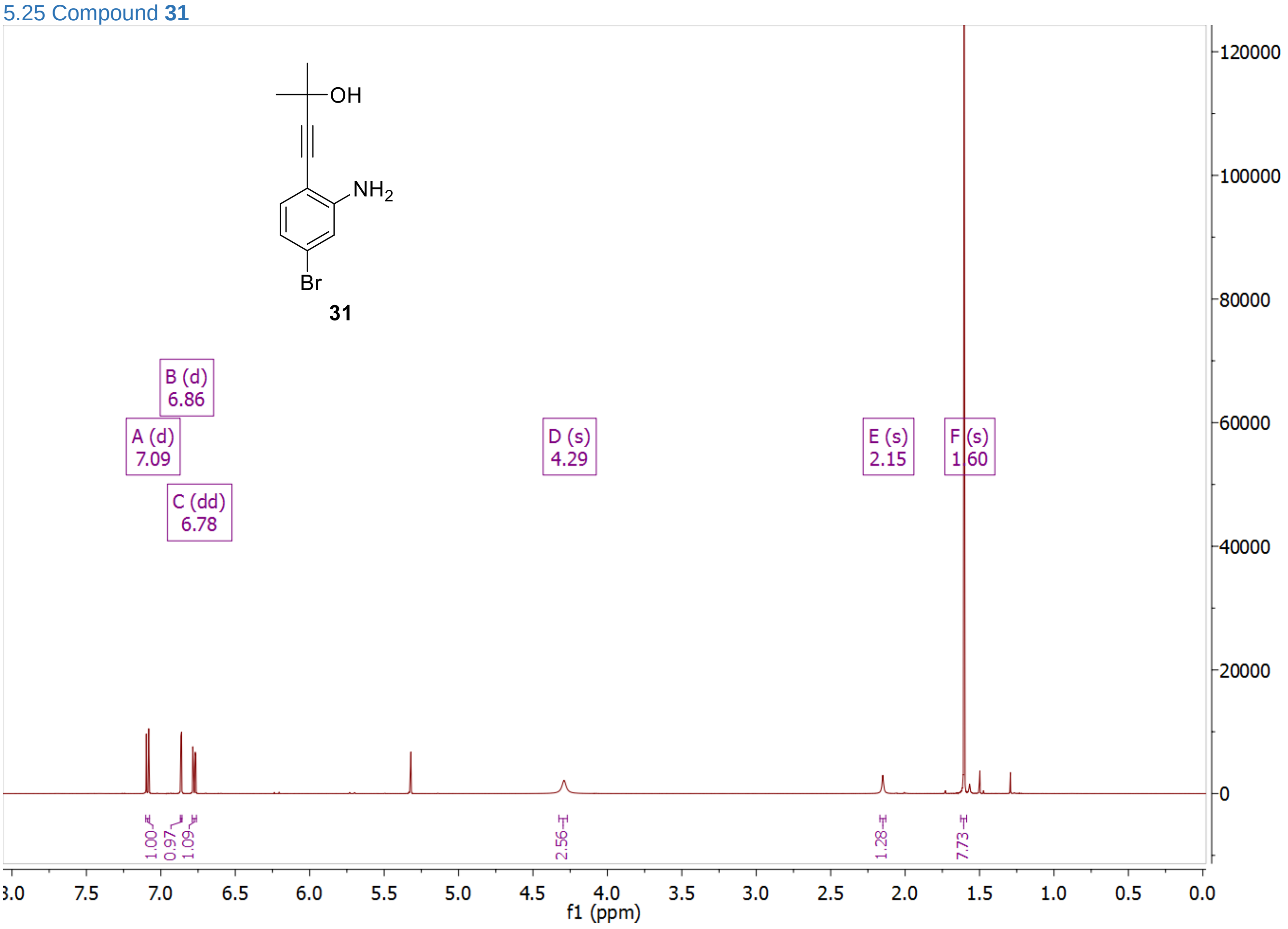

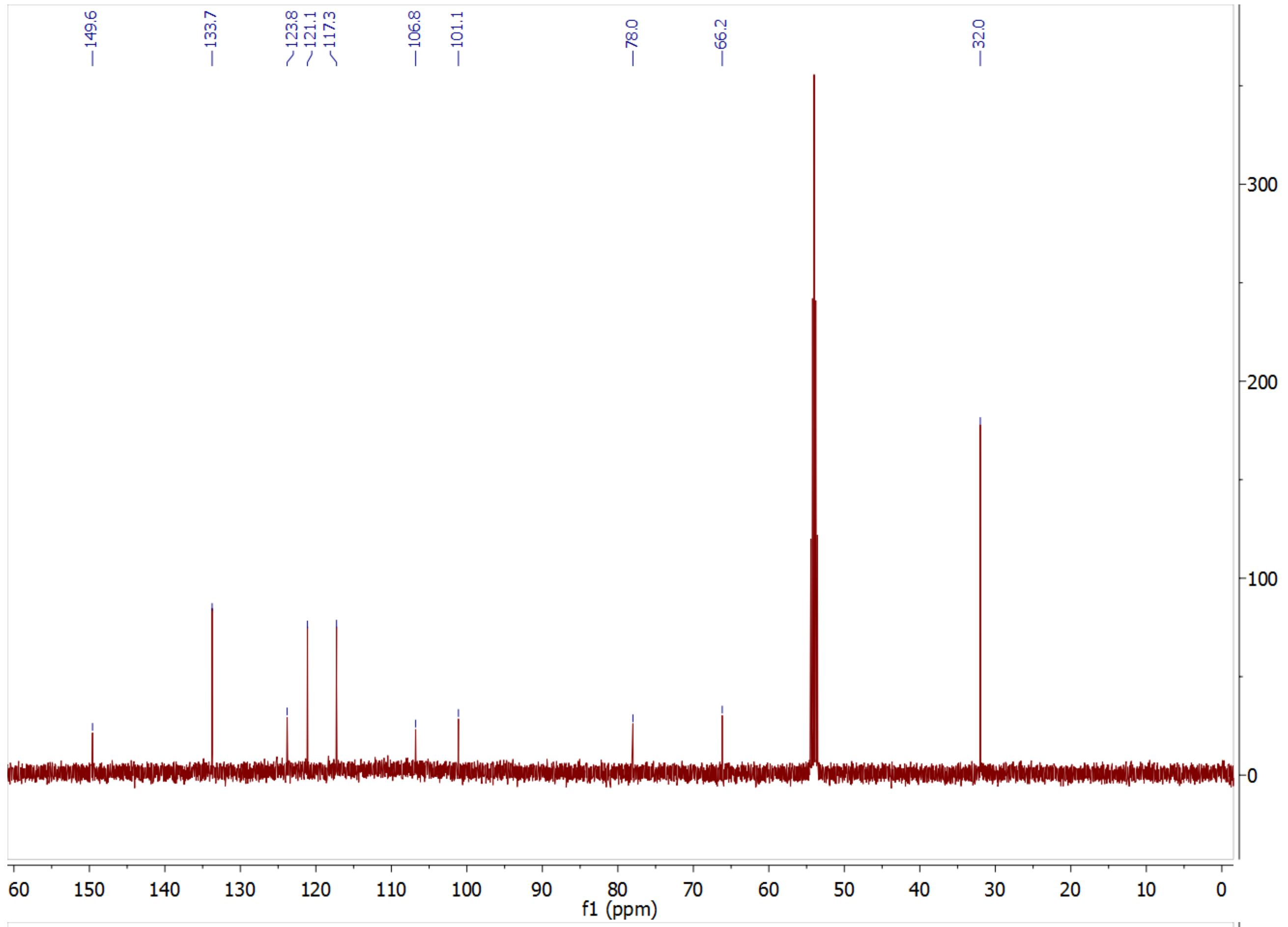
149.6
133.7
123.8
121.1
117.3
106.8
101.1
78.0
66.2
32.0
f1 (ppm)

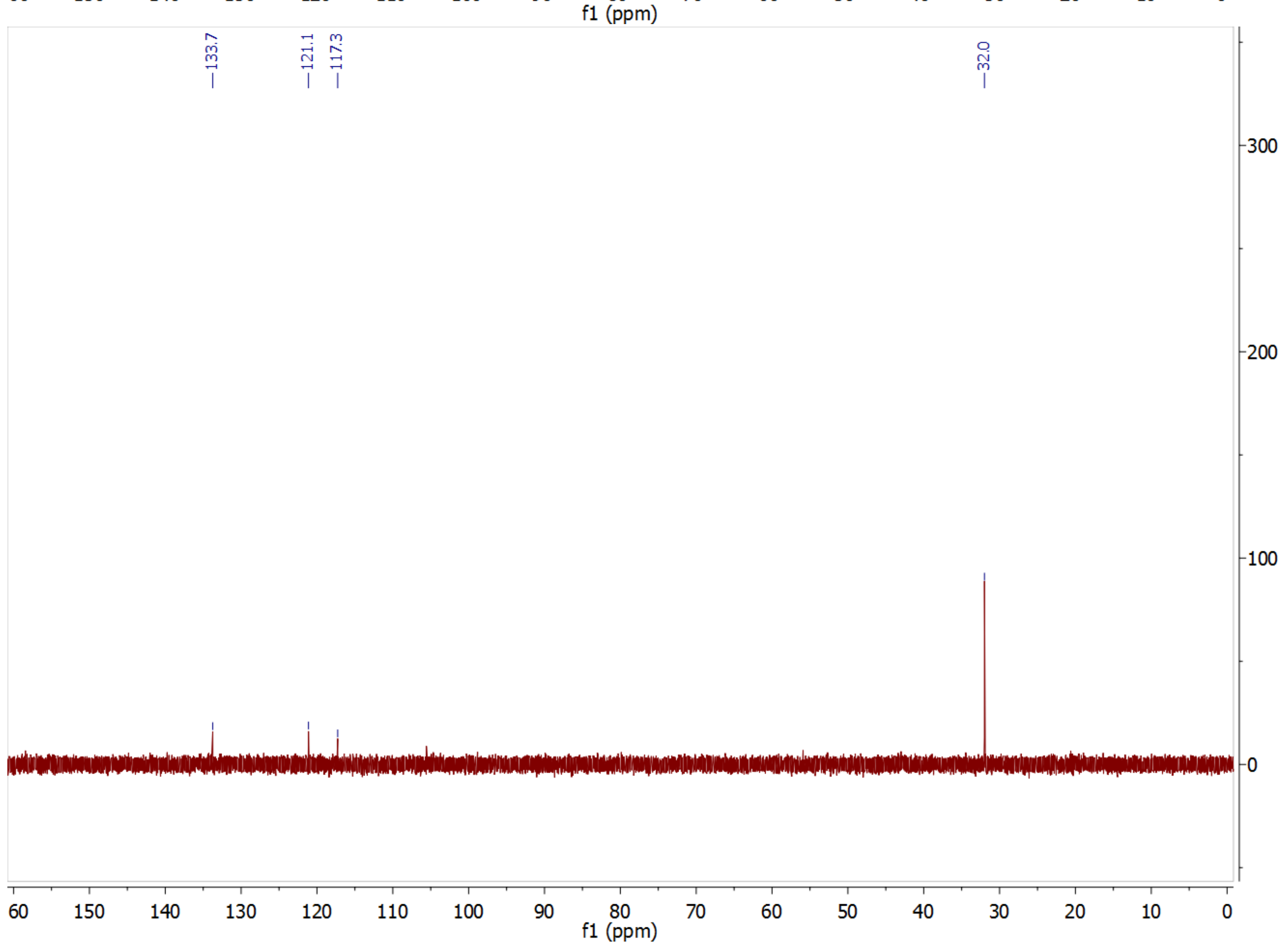
133.7
121.1
117.3
32.0
f1 (ppm)

High Resolution Mass Spectrometry Report

Sample Name	**Linda Bannwart / Ba557**	Instrument	maXis 4G
Comment	10 ug/mL in MeOH, analyzed in MeOH	Method	21 Direct_pos_low.m

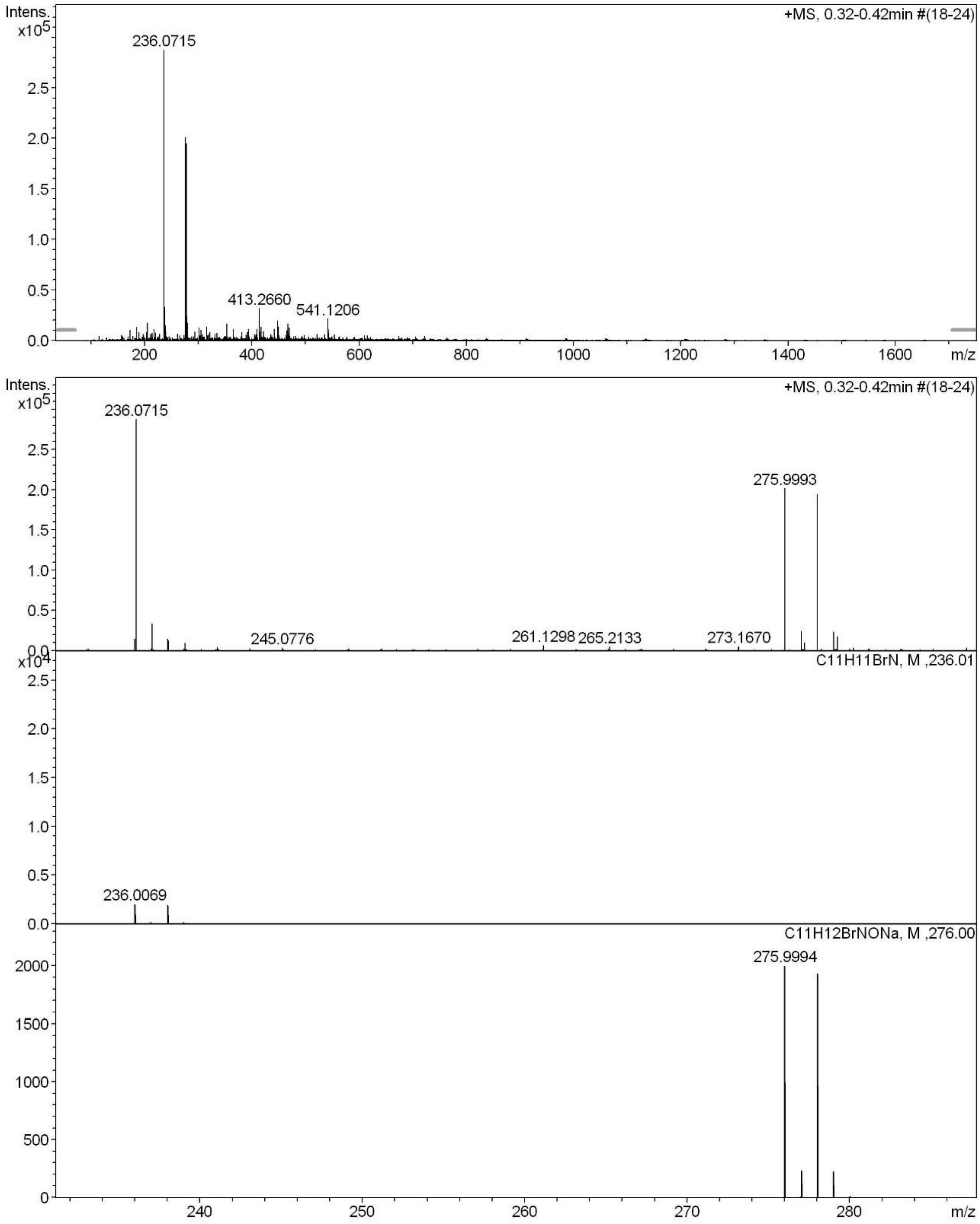

High Resolution Mass Spectrometry Report

Measured m/z vs. theoretical m/z

Meas. m/z	#	Formula	Score	m/z	err [mDa]	err [ppm]	mSigma	rdb	e⁻ Conf	z
236.0065	1	C 11 H 11 Br N	100.00	236.0069	0.4	1.8	15.5	6.5	even	1+
275.9993	1	C 11 H 12 Br N Na O	100.00	275.9994	0.2	0.6	6.9	5.5	even	

Mass list

#	m/z	I %	I
1	115.0370	1.4	3963
2	157.0861	1.8	5092
3	169.0468	1.3	3865
4	173.0781	3.5	10095
5	178.1337	1.4	4003
6	185.1146	4.5	12903
7	189.0883	2.7	7884
8	197.0780	2.1	5931
9	198.0871	1.6	4592
10	203.0526	2.7	7859
11	205.0615	5.9	17040
12	211.0938	2.0	5885
13	214.0891	2.4	6888
14	217.0829	1.8	5178
15	217.1041	3.8	10956
16	220.1668	2.6	7561
17	228.0988	2.1	6139
18	236.0065	5.2	14795
19	236.0715	100.0	287145
20	237.0744	11.5	32976
21	238.0045	5.1	14737
22	238.0674	4.3	12343
23	239.0884	3.2	9062
24	241.0678	1.4	3916
25	261.1298	2.2	6348
26	265.2133	1.5	4419
27	273.1454	1.4	3916
28	273.1670	1.8	5203
29	275.9993	70.0	201098
30	277.0022	8.2	23631
31	277.2132	3.4	9724
32	277.9972	67.7	194260
33	279.0003	8.1	23306
34	279.2290	6.1	17400
35	293.2080	1.9	5568
36	293.2446	3.0	8565
37	301.0748	2.3	6697
38	301.1407	4.4	12762
39	301.2111	3.0	8534
40	303.2290	1.6	4577
41	305.1569	1.4	4015
42	305.2448	3.5	10166
43	307.2602	1.9	5553
44	315.1924	4.8	13669
45	317.1719	1.7	4770
46	319.2236	1.9	5481
47	321.2395	1.8	5198
48	321.2758	2.7	7815
49	331.1876	2.3	6663
50	331.2076	1.9	5474
51	335.1364	2.4	6972
52	349.1827	1.4	3997
53	353.2657	5.7	16373
54	365.1051	3.7	10550
55	365.1352	1.7	4762
56	365.2750	4.0	11370
57	381.2968	2.8	7985
58	389.2507	1.8	5133
59	391.2083	2.8	8058
60	393.2096	1.5	4352
61	393.2983	4.0	11360

#	m/z	I %	I
62	395.2765	1.4	3905
63	405.1220	2.0	5723
64	405.2031	1.4	3946
65	407.3130	2.0	5777
66	409.2919	3.9	11273
67	413.2660	11.1	31734
68	414.2691	2.9	8439
69	417.3446	4.5	12995
70	421.3288	3.0	8638
71	435.3444	2.0	5603
72	441.2969	3.8	10998
73	447.2919	1.5	4410
74	447.3443	6.8	19628
75	448.3479	2.1	5904
76	449.1532	1.7	4865
77	449.3732	4.7	13525
78	450.3764	1.6	4552
79	463.3747	1.8	5308
80	465.3694	3.4	9633
81	467.1016	5.6	16115
82	468.1028	2.3	6657
83	469.0999	1.5	4402
84	469.3284	4.4	12554
85	470.3312	1.4	3989
86	477.3903	1.4	3887
87	481.3648	1.5	4307
88	493.3498	1.4	3954
89	497.3590	1.8	5149
90	521.3808	2.2	6217
91	535.3964	2.0	5791
92	541.1206	7.5	21593
93	542.1209	3.7	10696
94	543.1194	2.6	7462
95	553.3891	2.0	5678
96	553.4584	1.9	5399
97	609.3398	1.7	4745
98	615.1391	1.6	4663
99	673.3915	1.4	4066
100	721.5757	1.4	4113

Acquisition Parameter

Source Type	ESI	Ion Polarity	Positive	Set Nebulizer	0.4 Bar
Focus	Not active	Set Capillary	3600 V	Set Dry Heater	180 °C
Scan Begin	75 m/z	Set End Plate Offset	-500 V	Set Dry Gas	3.0 l/min
Scan End	1700 m/z	Set Collision Cell RF	350.0 Vpp	Set Ion Energy (MS only)	4.0 eV

5.26 Compound **32**

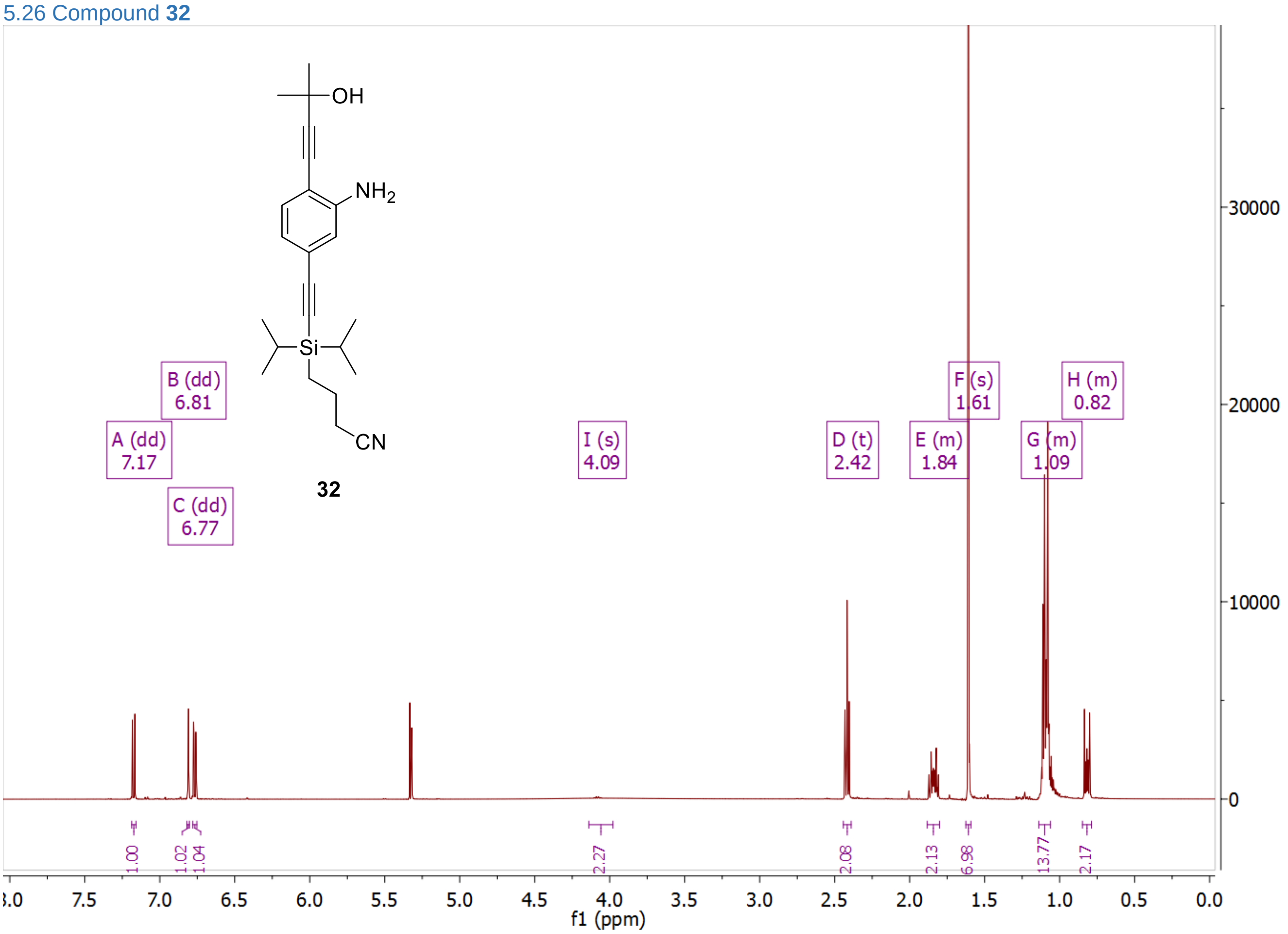

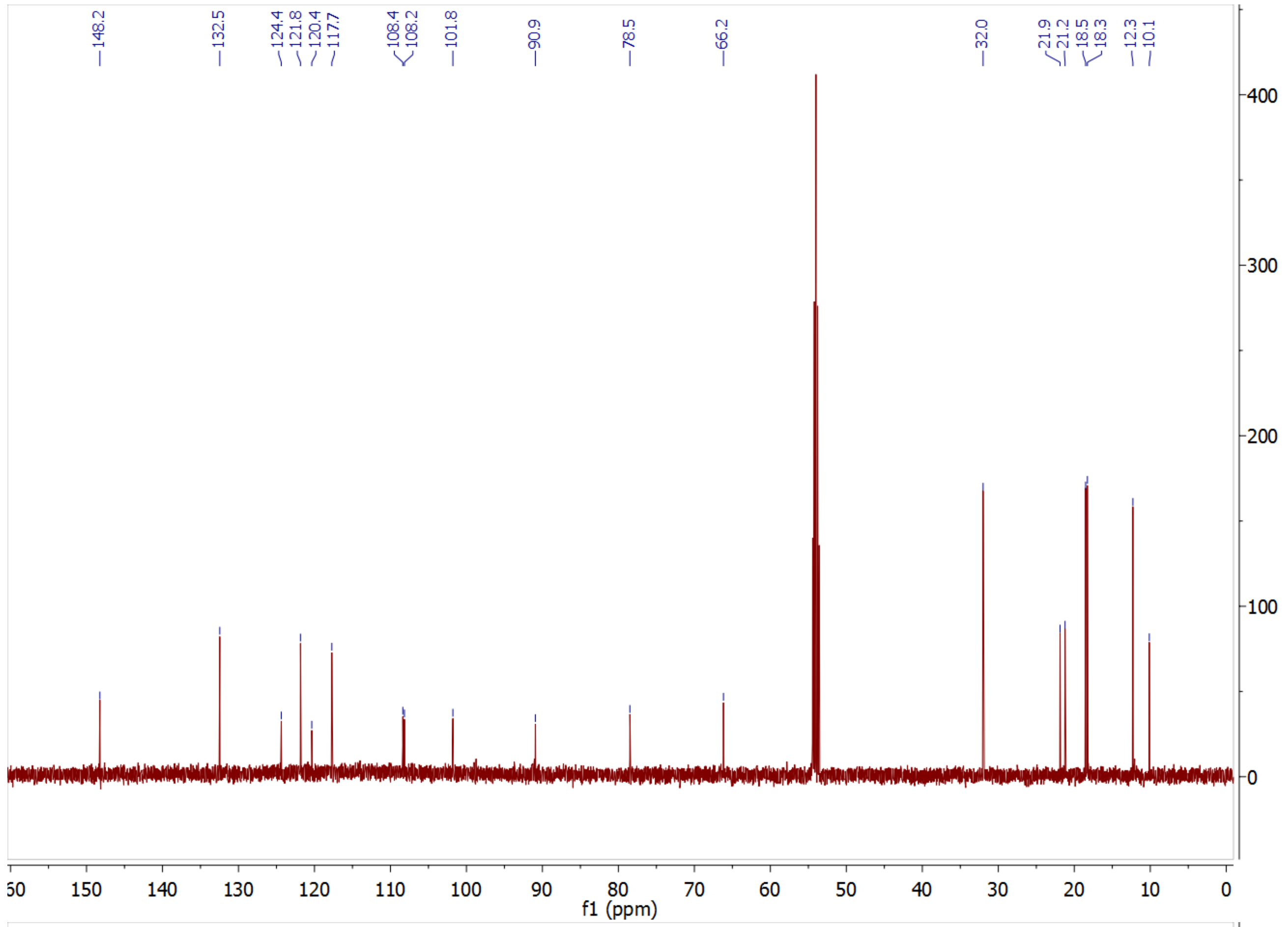
148.2
132.5
124.4
121.8
120.4
117.7
108.4
108.2
101.8
90.9
78.5
66.2
32.0
21.9
21.2
18.5
18.3
12.3
10.1
f1 (ppm)

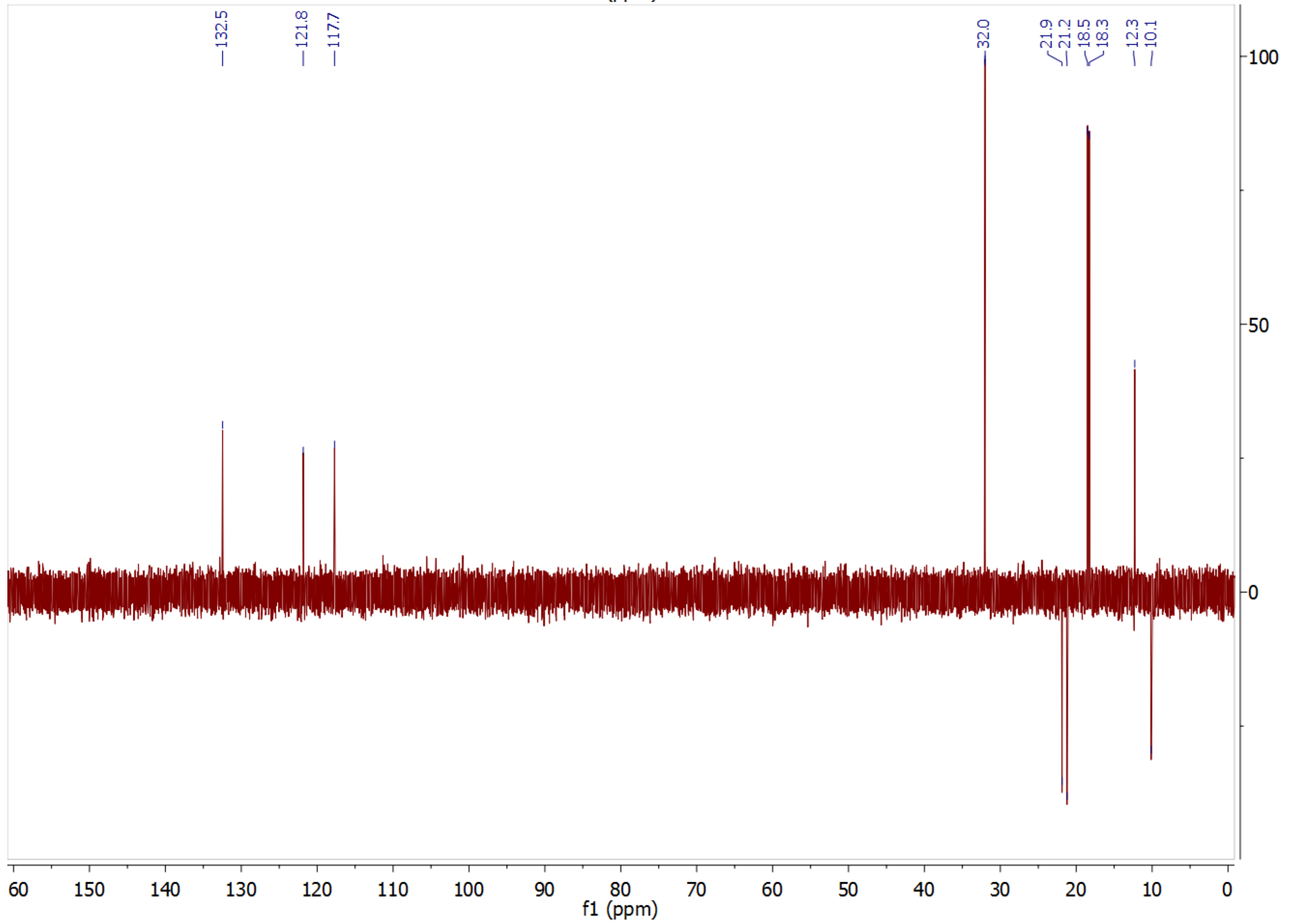
132.5
121.8
117.7
32.0
21.9
21.2
18.5
18.3
12.3
10.1
f1 (ppm)

High Resolution Mass Spectrometry Report

Sample Name	**Linda Bannwart / Ba559**	Instrument	maXis 4G
Comment	10 ug/mL in MeOH, analyzed in MeOH	Method	22 Direct_pos_mid.m

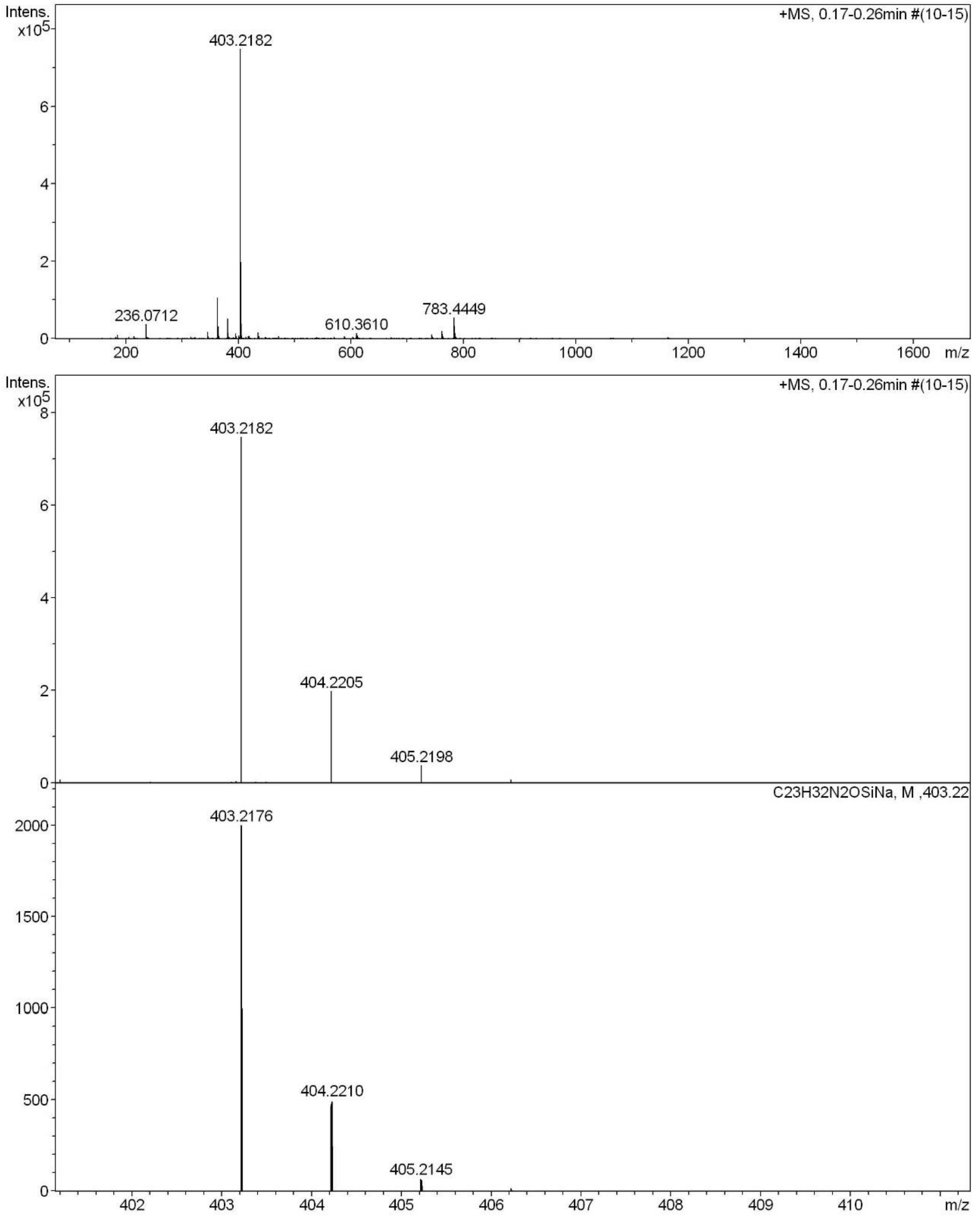

High Resolution Mass Spectrometry Report

Measured m/z vs. theoretical m/z

Meas. m/z	#	Formula	Score	m/z	err [mDa]	err [ppm]	mSigma	rdb	e⁻ Conf	z
403.2182	1	C 23 H 32 N 2 Na O Si	100.00	403.2176	-0.6	-1.4	27.4	9.5	even	1+

Mass list

#	m/z	I %	I
1	182.1358	0.5	3750
2	185.1147	1.1	8205
3	205.0601	0.5	3479
4	214.0896	0.6	4768
5	217.1039	0.2	1442
6	236.0712	4.8	35705
7	237.0742	0.5	3875
8	238.0671	0.3	1885
9	239.0885	0.2	1702
10	279.0925	0.2	1429
11	301.0747	0.2	1517
12	301.1405	0.2	1466
13	315.1924	0.5	3447
14	323.1927	0.3	2496
15	331.1882	0.2	1534
16	345.1753	2.3	17326
17	346.1774	0.6	4693
18	347.1799	0.2	1413
19	363.2249	14.1	105381
20	364.2274	4.0	29755
21	365.2272	0.8	6001
22	366.2268	0.2	1454
23	381.2354	6.8	51118
24	382.2379	1.8	13804
25	383.2384	0.4	3230
26	391.2087	0.4	2734
27	393.2980	0.2	1433
28	395.2509	1.7	12388
29	396.2537	0.6	4289
30	401.2019	0.9	6694
31	402.2045	0.3	2188
32	402.4921	0.2	1502
33	403.1078	0.3	2268
34	403.1565	0.4	3084
35	403.2182	100.0	747051
36	403.3792	0.2	1646
37	403.4470	0.2	1499
38	403.4916	0.2	1650
39	403.5212	0.2	1467
40	403.6051	0.2	1358
41	403.6653	0.2	1268
42	404.1667	0.2	1429
43	404.2205	26.4	197321
44	405.2198	5.0	37452
45	406.2201	1.0	7231
46	407.2210	0.2	1398
47	413.2659	0.5	3424
48	417.3446	0.7	5424
49	418.3486	0.2	1500
50	419.1912	0.7	5438
51	420.1945	0.2	1636
52	435.2435	2.0	14619
53	436.2460	0.7	4896
54	437.2433	0.2	1502
55	447.3440	0.3	2402
56	449.1542	0.2	1394
57	449.3733	0.2	1854
58	454.3251	0.2	1654
59	465.3703	0.2	1674
60	471.2049	0.6	4229
61	472.2076	0.2	1449
62	501.2148	0.2	1677

High Resolution Mass Spectrometry Report

#	m/z	I %	I
63	509.2448	0.2	1558
64	536.1649	0.2	1595
65	539.1925	0.3	2588
66	541.1206	0.2	1795
67	543.3132	0.2	1387
68	552.3208	0.2	1591
69	570.3683	0.4	3350
70	571.3712	0.2	1691
71	588.3793	0.6	4651
72	589.3817	0.3	2244
73	603.3770	0.4	3323
74	604.3801	0.2	1534
75	609.3401	0.3	1946
76	610.3610	1.8	13698
77	611.3641	0.9	6986
78	612.3642	0.3	2360
79	614.5213	0.2	1268
80	671.4524	0.3	2411
81	672.4551	0.2	1300
82	721.5743	0.2	1605
83	743.4519	1.3	9557
84	744.4542	0.7	5293
85	745.4554	0.3	2408
86	761.4629	2.4	18016
87	762.4656	1.5	11087
88	763.4653	0.6	4480
89	764.4673	0.2	1481
90	781.4282	0.2	1549
91	783.4449	7.1	53052
92	784.4475	4.2	31541
93	785.4482	1.7	12875
94	786.4488	0.5	3921
95	793.4938	0.2	1523
96	827.5710	0.2	1431
97	851.4314	0.2	1377
98	919.4191	0.2	1536
99	1163.6679	0.3	1928
100	1164.6712	0.2	1597

Acquisition Parameter

Source Type	ESI	Ion Polarity	Positive	Set Nebulizer	0.4 Bar		
Focus	Not active	Set Capillary	3600 V	Set Dry Heater	180 °C		
Scan Begin	75 m/z	Set End Plate Offset	-500 V	Set Dry Gas	4.0 l/min		
Scan End	1700 m/z	Set Collision Cell RF	350.0 Vpp	Set Ion Energy (MS only)	4.0 eV		

5.27 Compound **33**

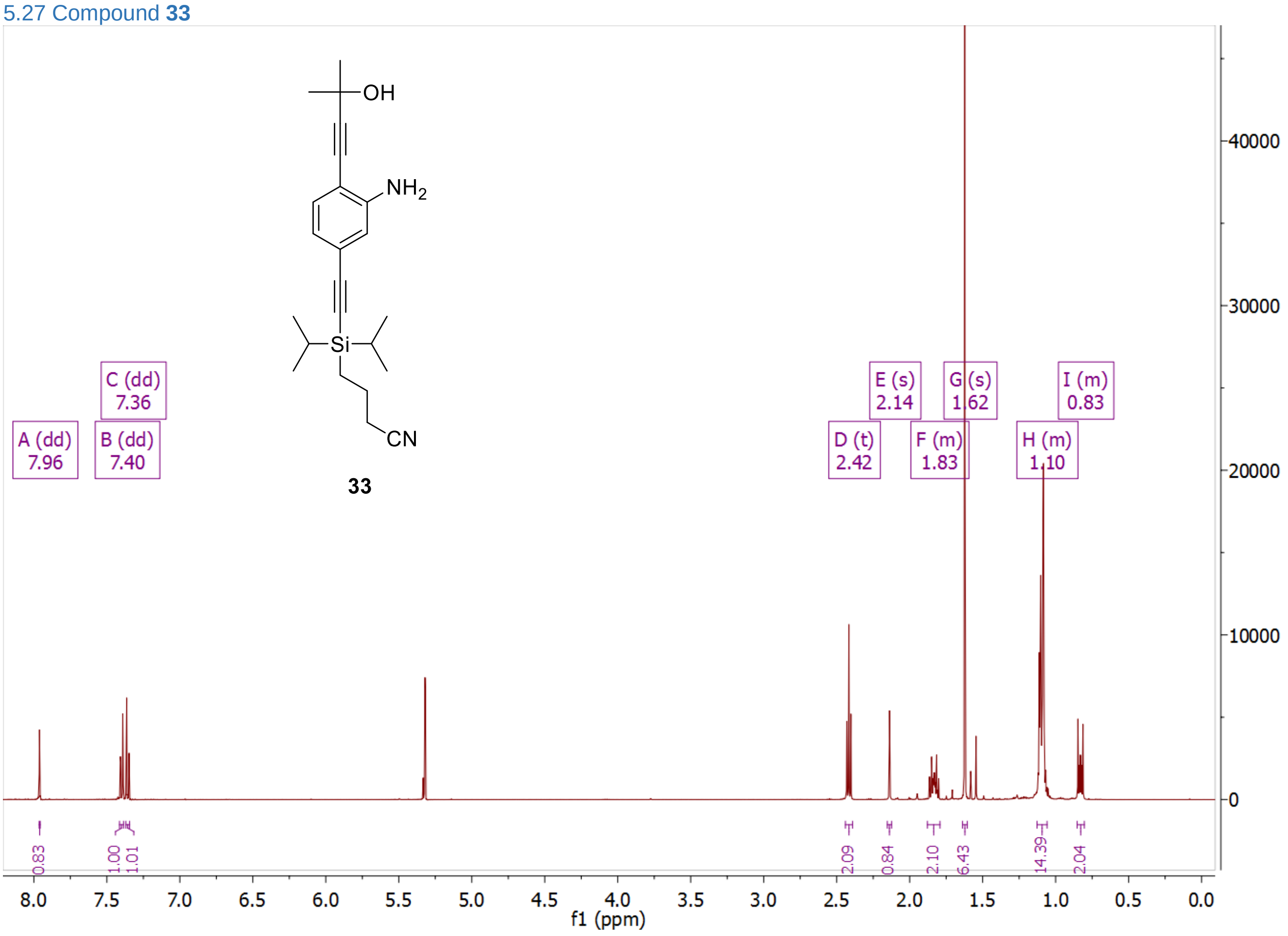

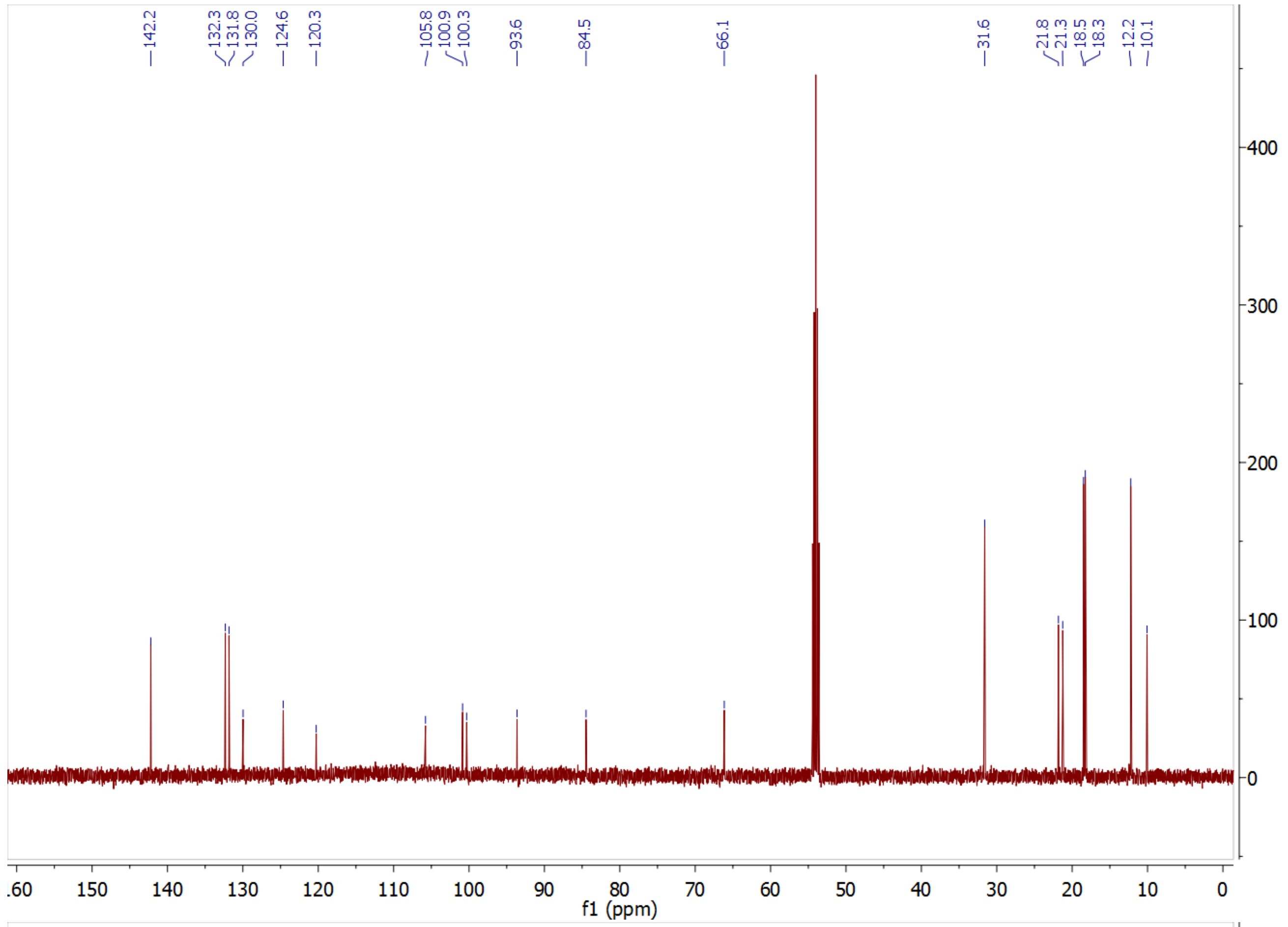
142.2
132.3
131.8
130.0
124.6
120.3
105.8
100.9
100.3
93.6
84.5
66.1
31.6
21.8
21.3
18.5
18.3
12.2
10.1
400
300
200
100
0
f1 (ppm)

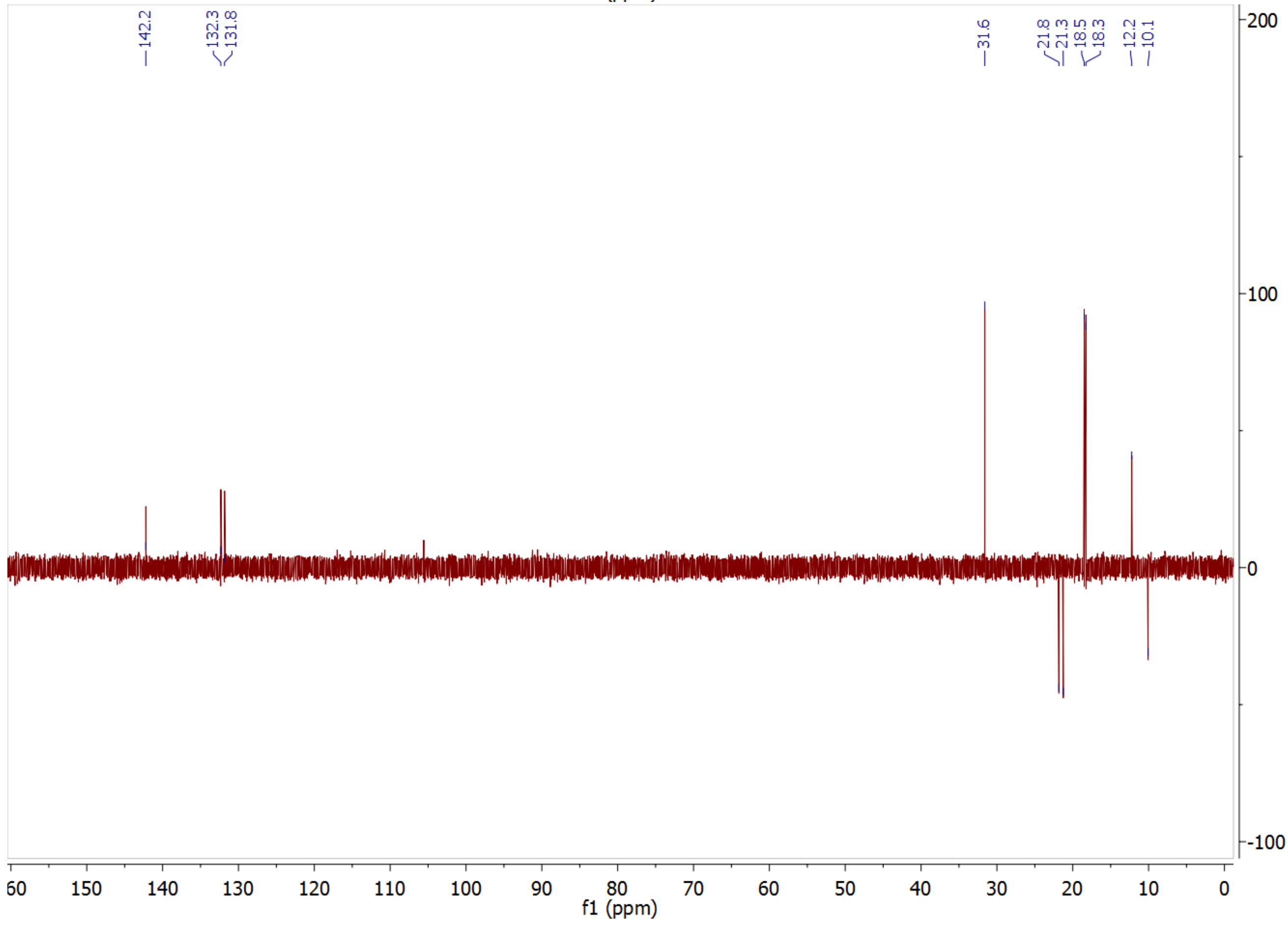
142.2
132.3
131.8
31.6
21.8
21.3
18.5
18.3
12.2
10.1
200
100
0
-100
f1 (ppm)

High Resolution Mass Spectrometry Report

Sample Name	**Linda Bannwart / Ba560**	Instrument	maXis 4G
Comment	10 ug/mL in MeOH, analyzed in MeOH	Method	22 Direct_pos_mid.m

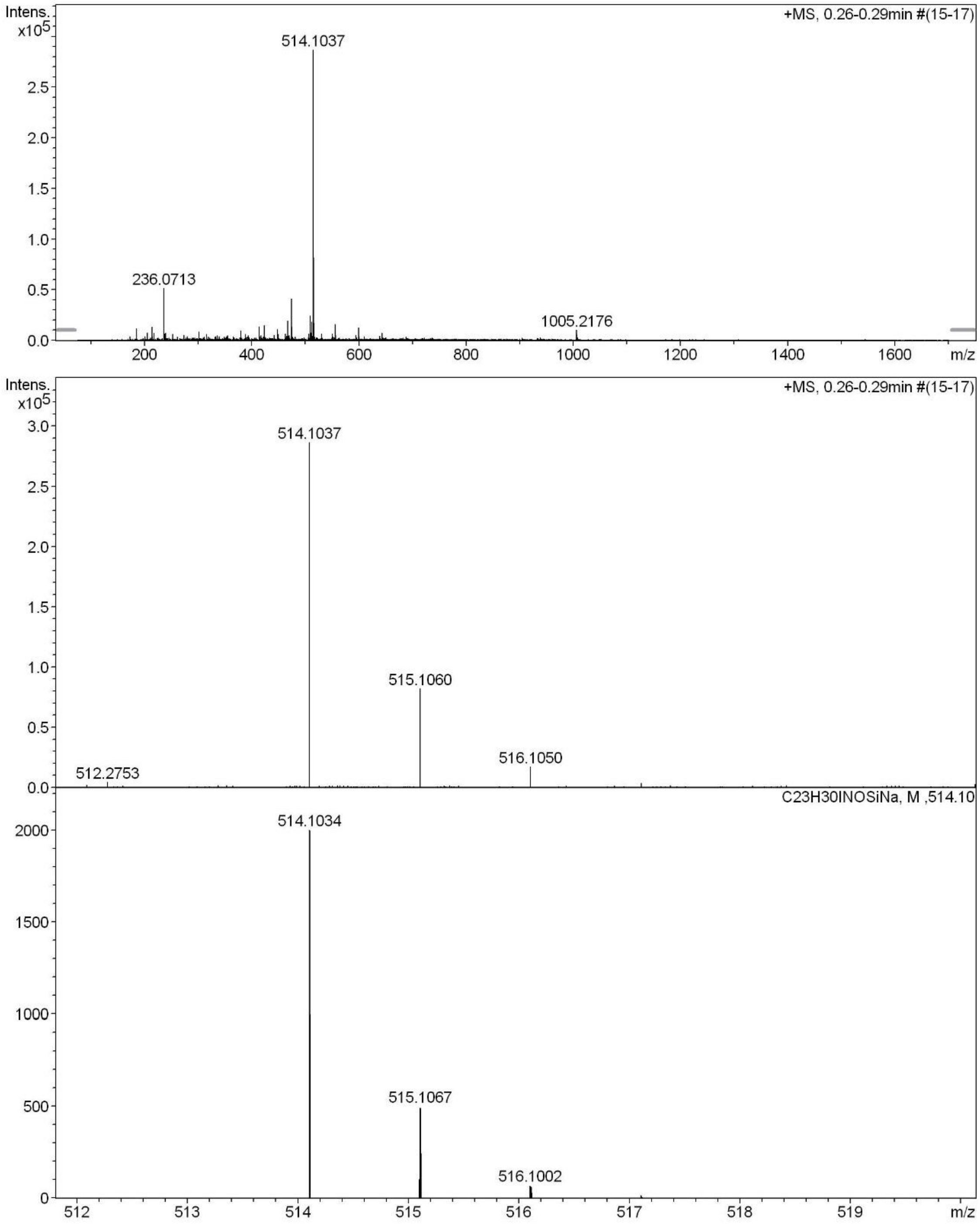

Bruker Compass DataAnalysis 4.0 Acquisition Date 21.02.2019 09:33:08 Page 1 of 3

High Resolution Mass Spectrometry Report

Measured m/z vs. theoretical m/z

Meas. m/z	#	Formula	Score	m/z	err [mDa]	err [ppm]	mSigma	rdb	e⁻ Conf	z
514.1037	1	C 23 H 30 I N Na O Si	100.00	514.1034	-0.4	-0.7	12.5	9.5	even	1+

Mass list

#	m/z	I %	I
1	173.0784	1.3	3723
2	185.1147	4.0	11397
3	201.1024	1.3	3863
4	205.0600	2.5	7246
5	211.0937	0.9	2477
6	214.0893	4.5	12972
7	215.1249	1.2	3482
8	217.1046	2.4	6739
9	225.1099	0.8	2425
10	236.0713	17.9	51233
11	237.0740	2.2	6375
12	238.0683	1.0	2850
13	239.0886	2.4	6864
14	252.0847	2.0	5862
15	261.1301	1.2	3304
16	273.1667	1.8	5278
17	277.2123	0.8	2408
18	279.2296	1.3	3675
19	301.1410	2.9	8406
20	311.1569	1.2	3296
21	315.1923	2.0	5821
22	319.2242	1.2	3413
23	331.2088	1.2	3441
24	333.1694	1.3	3731
25	335.1676	1.6	4571
26	339.1778	1.3	3583
27	348.9903	1.2	3380
28	353.2662	1.2	3477
29	355.1841	1.6	4658
30	365.1060	1.3	3842
31	367.2081	1.0	2787
32	377.1954	0.9	2676
33	379.1935	3.3	9400
34	381.2963	0.9	2450
35	388.2064	2.1	5953
36	389.2093	0.9	2462
37	389.2501	1.0	2885
38	391.2092	1.1	3228
39	391.2833	0.9	2578
40	393.2964	1.6	4608
41	395.2758	0.9	2586
42	413.2658	4.8	13650
43	414.2686	1.3	3773
44	417.3435	1.7	4865
45	418.2639	1.0	2870
46	421.3275	1.2	3380
47	423.2202	5.2	14830
48	424.2235	1.2	3474
49	433.1024	0.9	2544
50	441.2973	1.8	5160
51	447.3443	3.8	10809
52	448.3475	1.2	3407
53	449.3724	2.0	5816
54	462.2907	2.3	6684
55	463.3759	1.0	2877
56	465.3710	1.5	4251
57	467.2459	6.6	18969
58	468.2502	1.5	4159
59	469.3284	1.6	4479
60	474.1105	14.3	40957
61	475.1133	4.6	13082
62	476.1122	1.1	3021

High Resolution Mass Spectrometry Report

#	m/z	I %	I
63	481.3646	1.1	3044
64	497.3575	1.0	2758
65	505.3350	0.9	2705
66	506.3176	2.2	6341
67	509.1478	8.4	24106
68	510.1507	2.5	7237
69	511.2720	6.4	18228
70	512.2753	1.5	4256
71	514.1037	100.0	286286
72	515.1060	28.5	81709
73	516.1050	5.9	16976
74	517.1056	1.2	3377
75	520.1269	1.0	2852
76	521.3815	1.0	2826
77	530.0771	2.3	6458
78	531.0808	0.8	2409
79	550.3426	2.2	6317
80	551.3484	1.1	3066
81	553.3887	0.9	2706
82	555.2984	5.5	15684
83	556.3021	1.8	5068
84	563.3799	0.9	2532
85	594.3698	1.7	5010
86	595.3750	0.9	2679
87	599.3249	4.4	12619
88	600.3273	1.3	3848
89	609.3461	1.2	3508
90	638.3959	1.4	4132
91	643.3510	2.4	6913
92	644.3545	0.8	2422
93	650.0783	0.9	2719
94	687.3767	1.1	3263
95	721.5751	0.8	2402
96	904.3799	0.9	2454
97	938.4582	1.0	2853
98	1005.2176	3.5	9974
99	1006.2203	1.9	5516
100	1007.2244	0.9	2642

Acquisition Parameter

Source Type	ESI	Ion Polarity	Positive	Set Nebulizer	0.4 Bar
Focus	Not active	Set Capillary	3600 V	Set Dry Heater	180 °C
Scan Begin	75 m/z	Set End Plate Offset	-500 V	Set Dry Gas	4.0 l/min
Scan End	1700 m/z	Set Collision Cell RF	350.0 Vpp	Set Ion Energy (MS only)	4.0 eV

5.28 Compound **C₂**

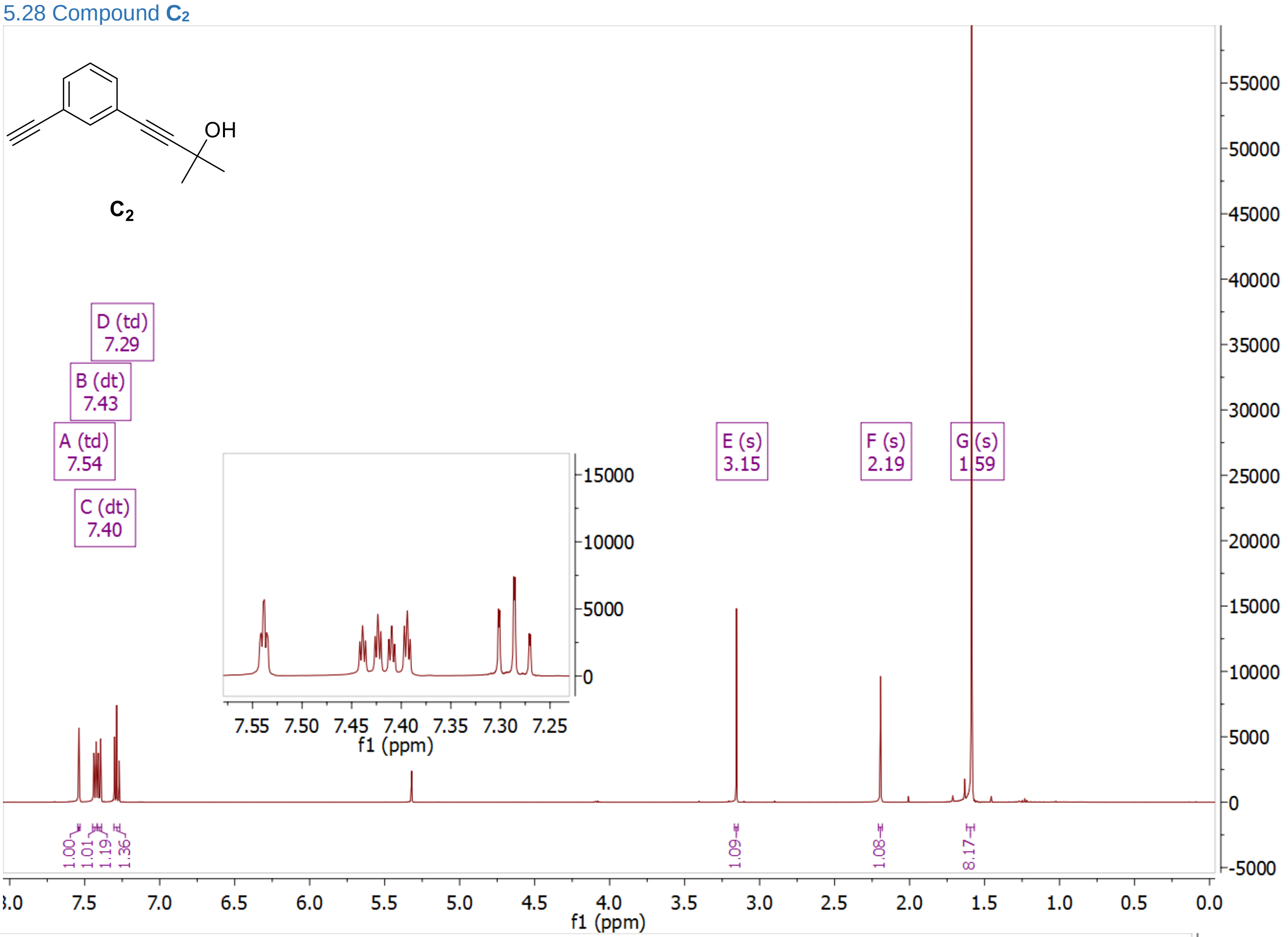

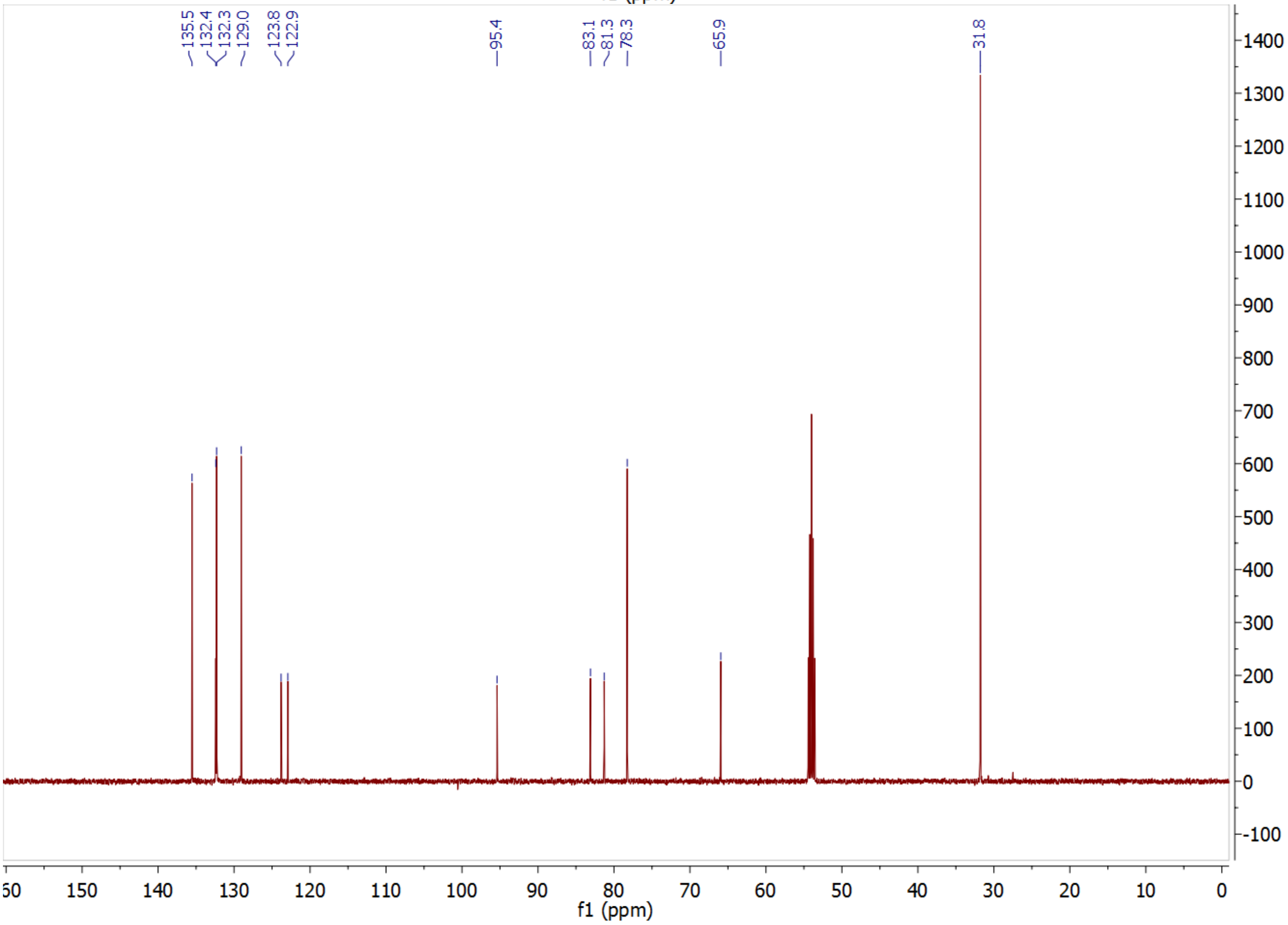

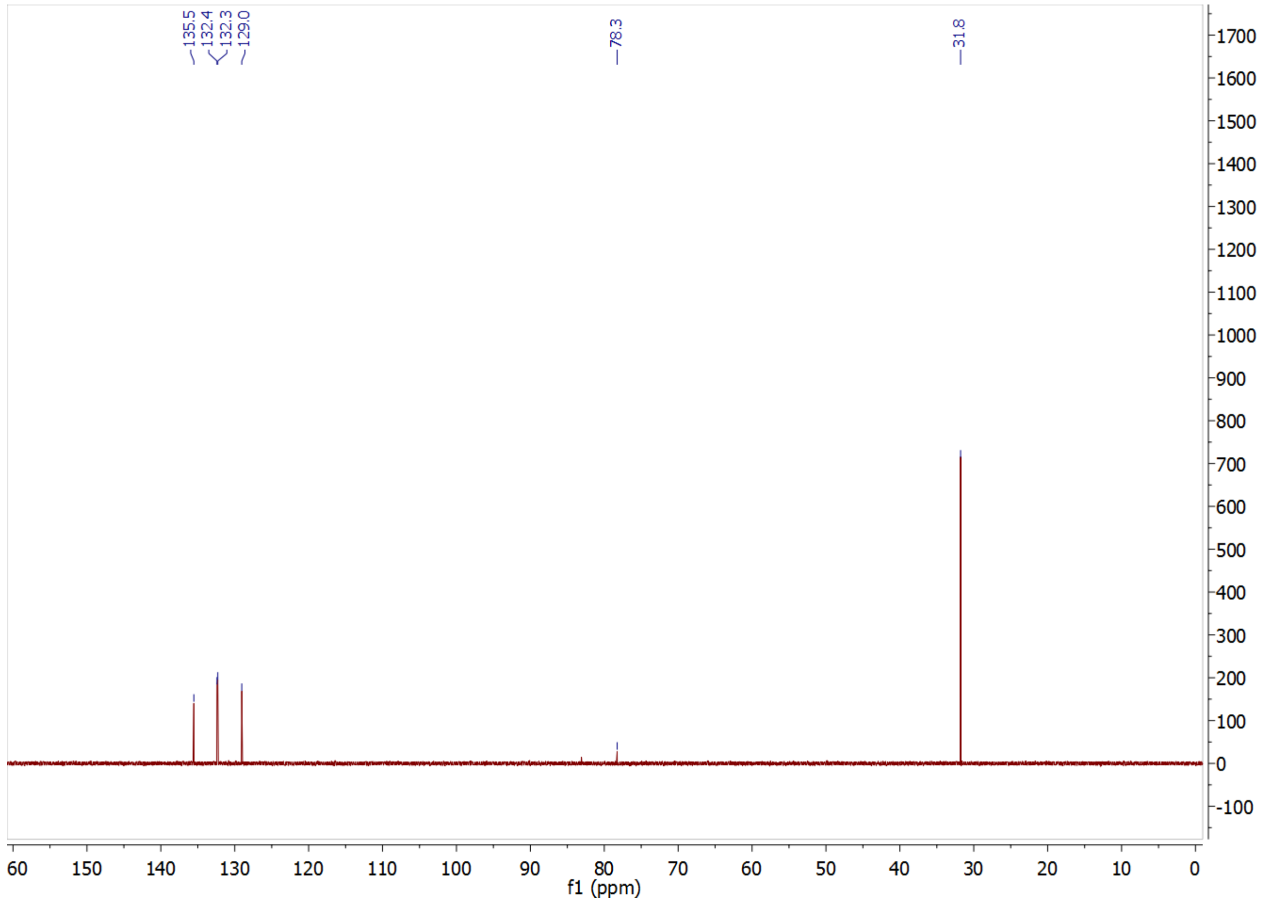

DEPT-135: $^{1}J_{CH}$ coupling= 145 Hz

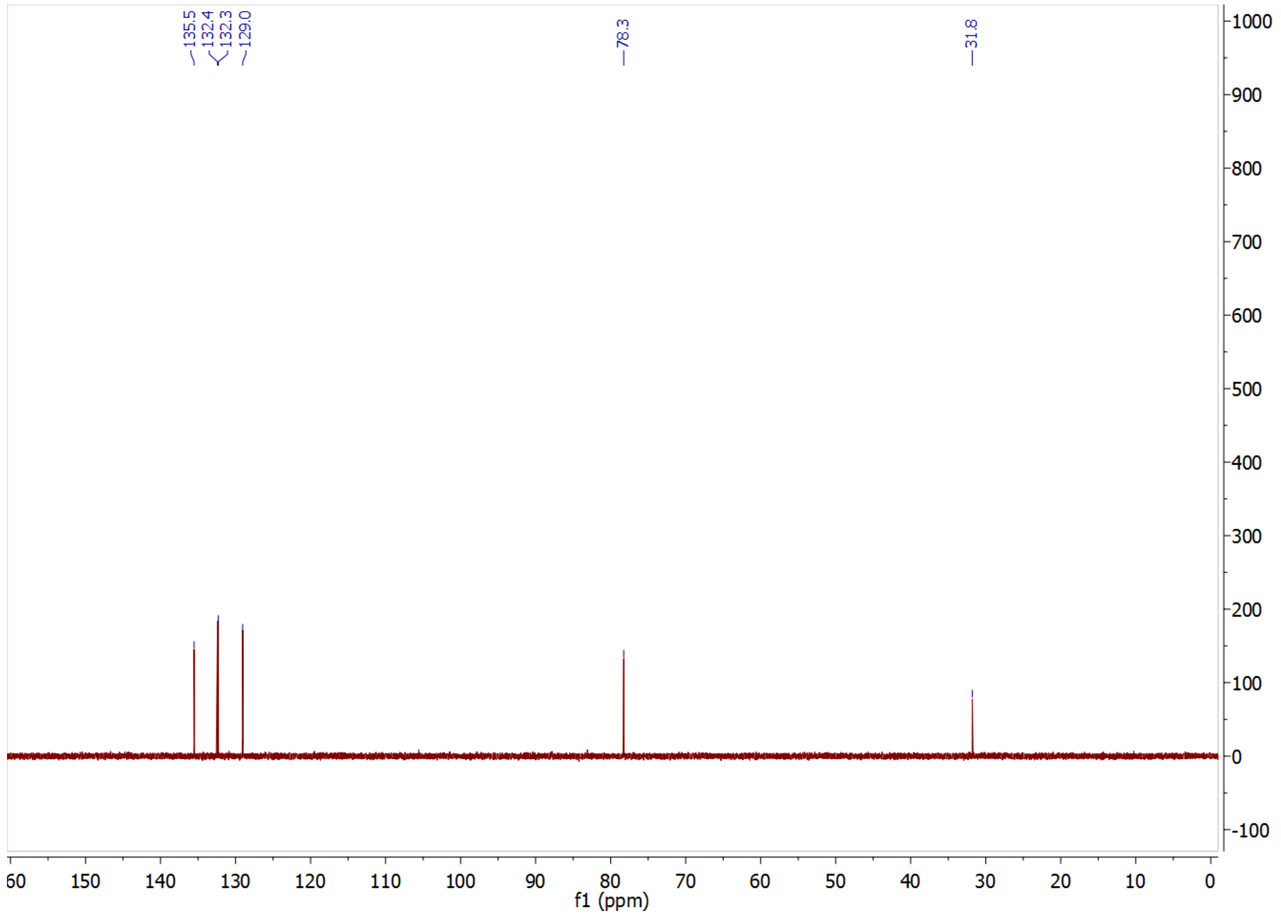

DEPT-135: $^{1}J_{CH}$ coupling= 200 Hz

High Resolution Mass Spectrometry Report

Sample Name	**Linda Bannwart / Ba572 chr1-1**	Instrument	maXis 4G
Comment	10 ug/mL in MeOH, analyzed in MeOH	Method	22 Direct_pos_mid.m

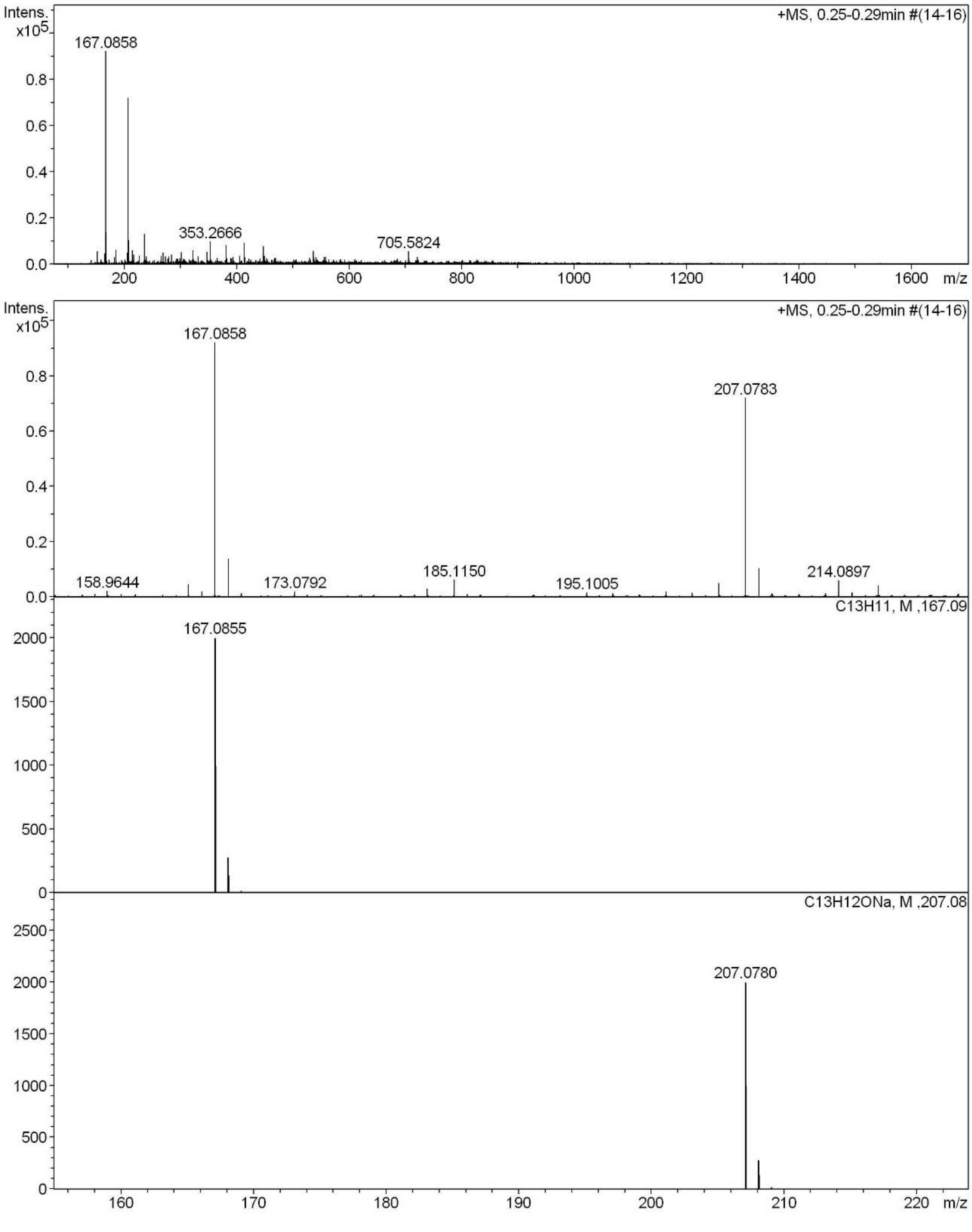

High Resolution Mass Spectrometry Report

Measured m/z vs. theoretical m/z

Meas. m/z	#	Formula	Score	m/z	err [mDa]	err [ppm]	mSigma	rdb	e⁻ Conf	z
167.0858	1	C 13 H 11	100.00	167.0855	-0.3	-1.9	5.1	8.5	even	1+
207.0783	1	C 13 H 12 Na O	100.00	207.0780	-0.3	-1.4	3.8	7.5	even	

Mass list

#	m/z	I %	I
1	140.9618	1.8	1678
2	152.0622	5.9	5381
3	158.9644	2.3	2106
4	165.0699	4.8	4403
5	166.0771	1.9	1777
6	167.0858	100.0	91935
7	168.0891	14.8	13638
8	173.0792	2.0	1850
9	183.0782	3.1	2824
10	185.1150	6.7	6153
11	195.1005	1.7	1588
12	201.1031	2.0	1882
13	205.0601	5.3	4869
14	207.0783	78.2	71877
15	208.0816	11.1	10219
16	214.0897	6.3	5834
17	215.1255	1.7	1590
18	217.1049	4.5	4103
19	226.9513	3.9	3629
20	236.0718	14.1	12990
21	237.0745	2.1	1944
22	238.0190	1.7	1604
23	239.0891	3.5	3201
24	266.0138	3.8	3483
25	269.0485	5.3	4854
26	273.1674	3.6	3302
27	277.2144	2.5	2276
28	279.2292	3.6	3326
29	284.0244	4.4	4071
30	293.2455	2.5	2261
31	294.9393	2.3	2129
32	299.1616	2.6	2374
33	301.1415	5.4	4950
34	301.2116	2.6	2360
35	305.2453	2.3	2149
36	307.2611	1.9	1743
37	315.1932	2.3	2116
38	321.1820	1.9	1761
39	321.2763	1.8	1684
40	322.1912	6.4	5902
41	323.1951	2.3	2103
42	331.1888	2.1	1955
43	331.2088	3.6	3305
44	347.2018	5.8	5322
45	349.0605	1.7	1567
46	353.1458	3.3	2998
47	353.2666	10.6	9750
48	354.2705	2.1	1913
49	365.1057	2.9	2666
50	381.2978	8.8	8100
51	382.3019	2.3	2113
52	383.1412	2.6	2379
53	389.2520	2.9	2679
54	391.2846	2.3	2123
55	391.3541	1.7	1574
56	393.2978	3.3	3036
57	405.1234	3.8	3468
58	413.2669	9.8	9031
59	414.2693	3.0	2753
60	421.3290	2.3	2158
61	425.3629	2.0	1830

High Resolution Mass Spectrometry Report

#	m/z	I %	I
62	441.2979	2.7	2481
63	447.2927	2.3	2107
64	447.3454	8.3	7649
65	448.3487	2.5	2303
66	449.3733	3.7	3377
67	453.1369	2.6	2416
68	455.1350	1.7	1573
69	462.1477	1.9	1719
70	463.3767	1.7	1604
71	467.1031	2.6	2382
72	469.3289	2.9	2675
73	501.4652	2.0	1831
74	505.3356	2.3	2078
75	529.4963	2.8	2569
76	536.1660	6.1	5619
77	537.1672	3.3	3006
78	538.1649	2.0	1802
79	541.1216	3.1	2819
80	542.1216	1.7	1602
81	543.5124	2.0	1858
82	553.4594	1.8	1629
83	555.5117	3.2	2952
84	557.5267	3.2	2929
85	563.3766	2.2	1991
86	571.5425	1.9	1756
87	583.5426	2.1	1901
88	585.5578	2.3	2103
89	591.4973	1.9	1741
90	610.1845	2.2	2030
91	621.4186	1.7	1593
92	663.4543	1.8	1625
93	680.4775	1.8	1612
94	684.2032	1.9	1708
95	685.4350	2.5	2271
96	705.5824	5.8	5353
97	706.5858	2.4	2246
98	721.5775	3.3	3011
99	801.6917	1.8	1635
100	827.7085	1.9	1723

Acquisition Parameter

**General**	Fore Vacuum	2.80e+000 mBar	High Vacuum	9.59e-008 mBar	Source Type	ESI
	Scan Begin	75 m/z	Scan End	1700 m/z	Ion Polarity	Positive
**Source**	Set Nebulizer	0.4 Bar	Set Capillary	3600 V	Set Dry Gas	4.0 l/min
	Set Dry Heater	180 °C	Set End Plate Offset	-500 V		
**Quadrupole**	Set Ion Energy (MS only)	4.0 eV				
**Coll. Cell**	Collision Energy	8.0 eV	Set Collision Cell RF	350.0 Vpp		
**Ion Cooler**	Set Ion Cooler Transfer Time	75.0 µs	Set Ion Cooler Pre Pulse Storage Time	10.0 µs		

5.29 Compound **34**

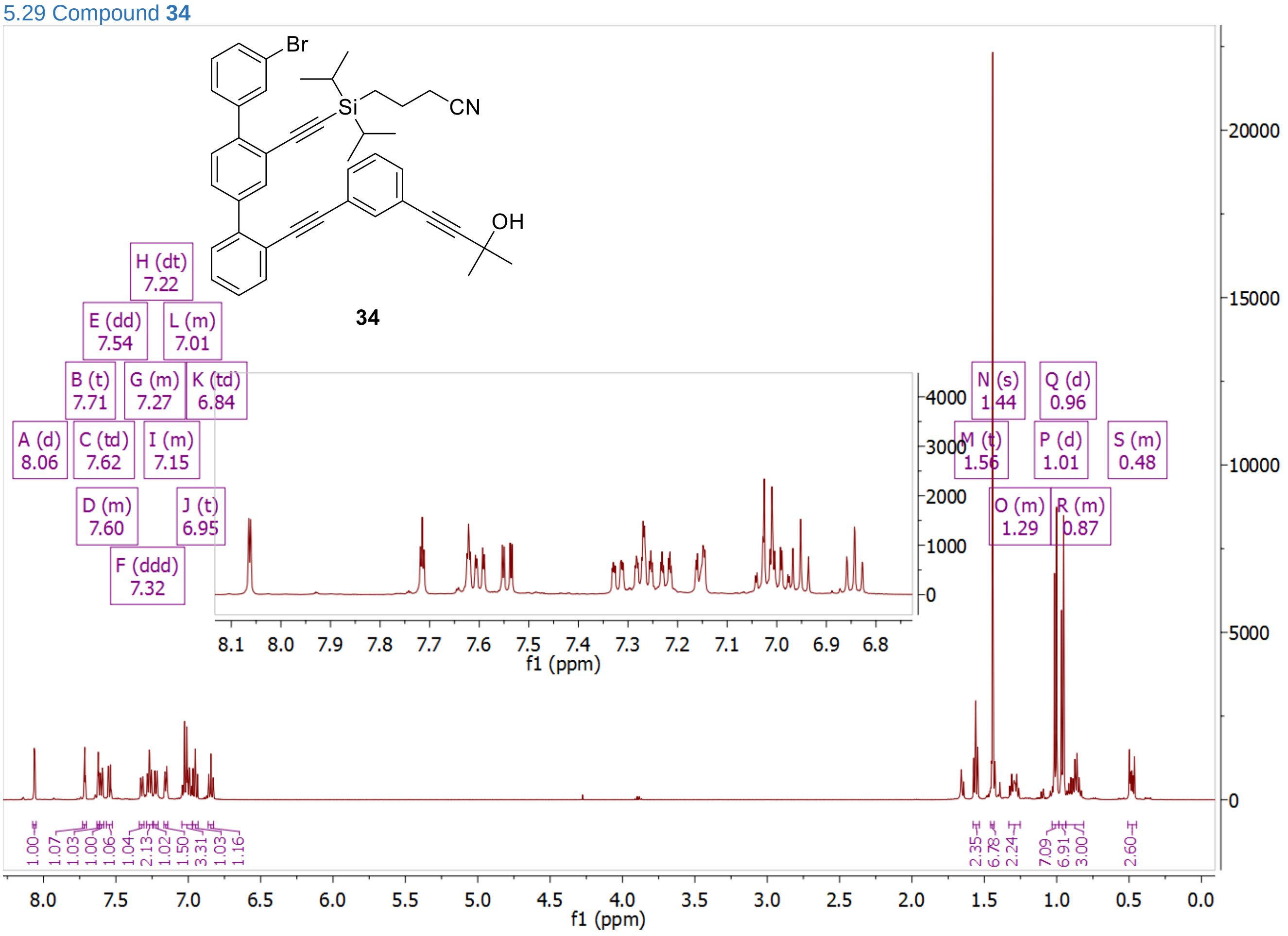

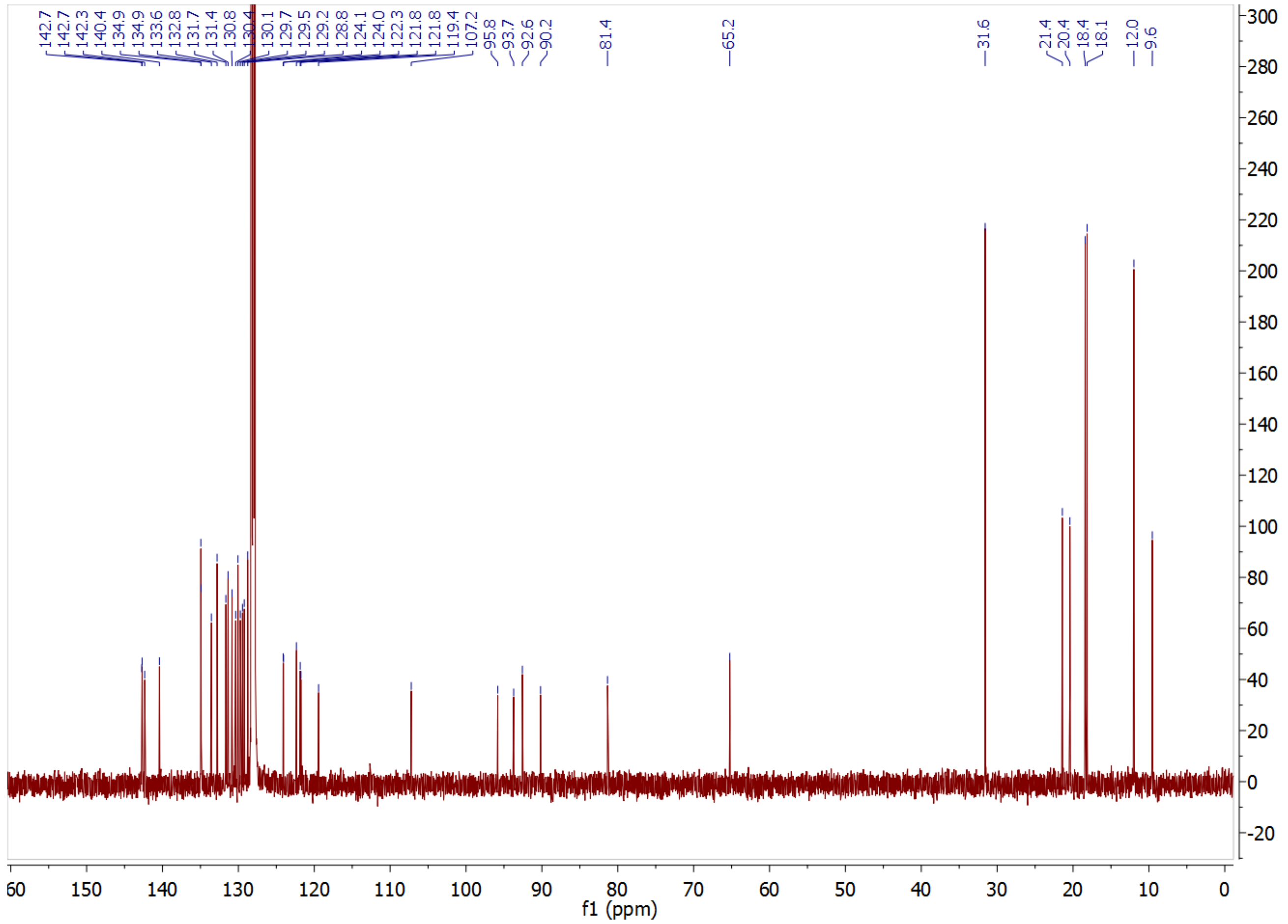

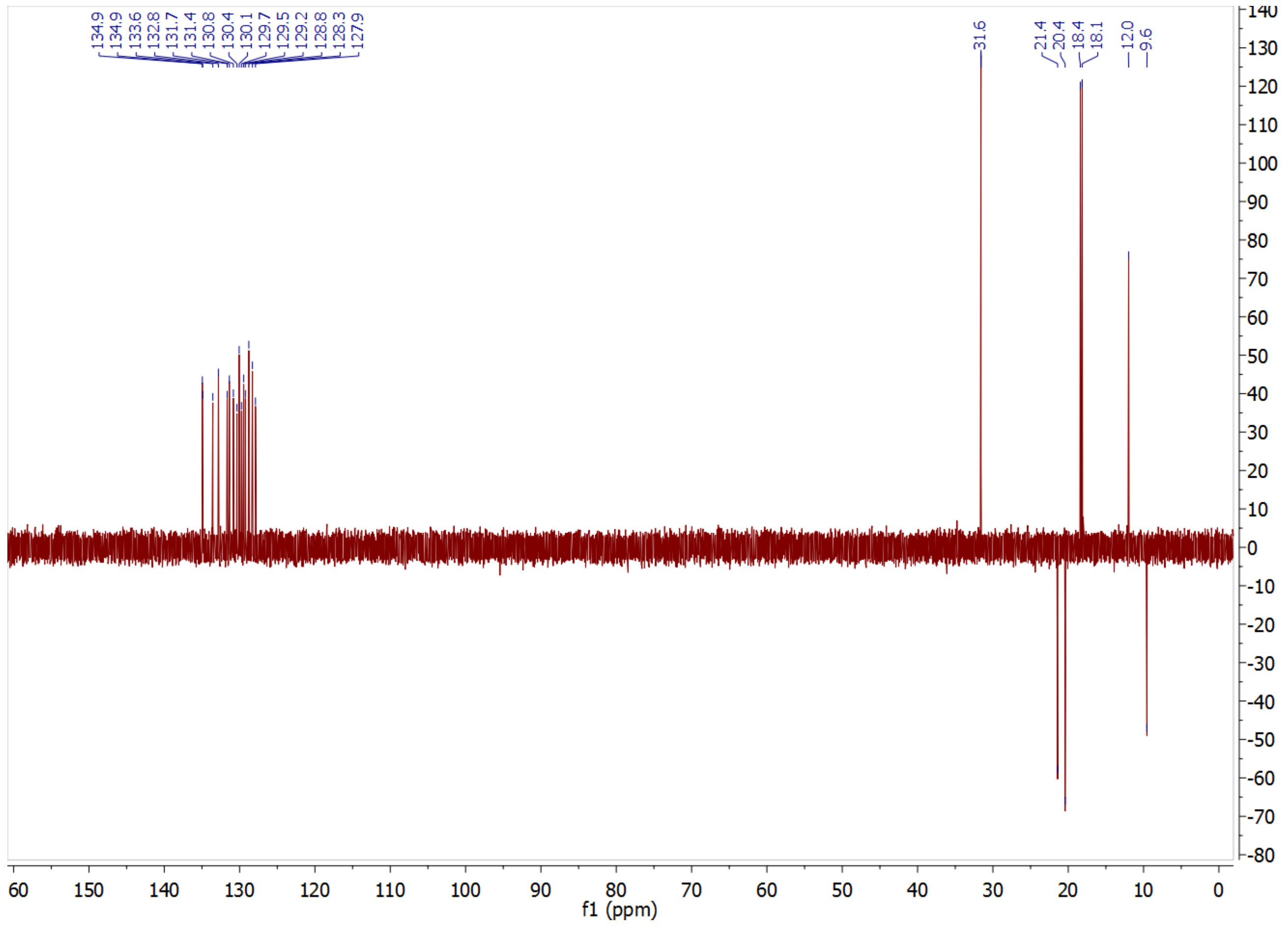

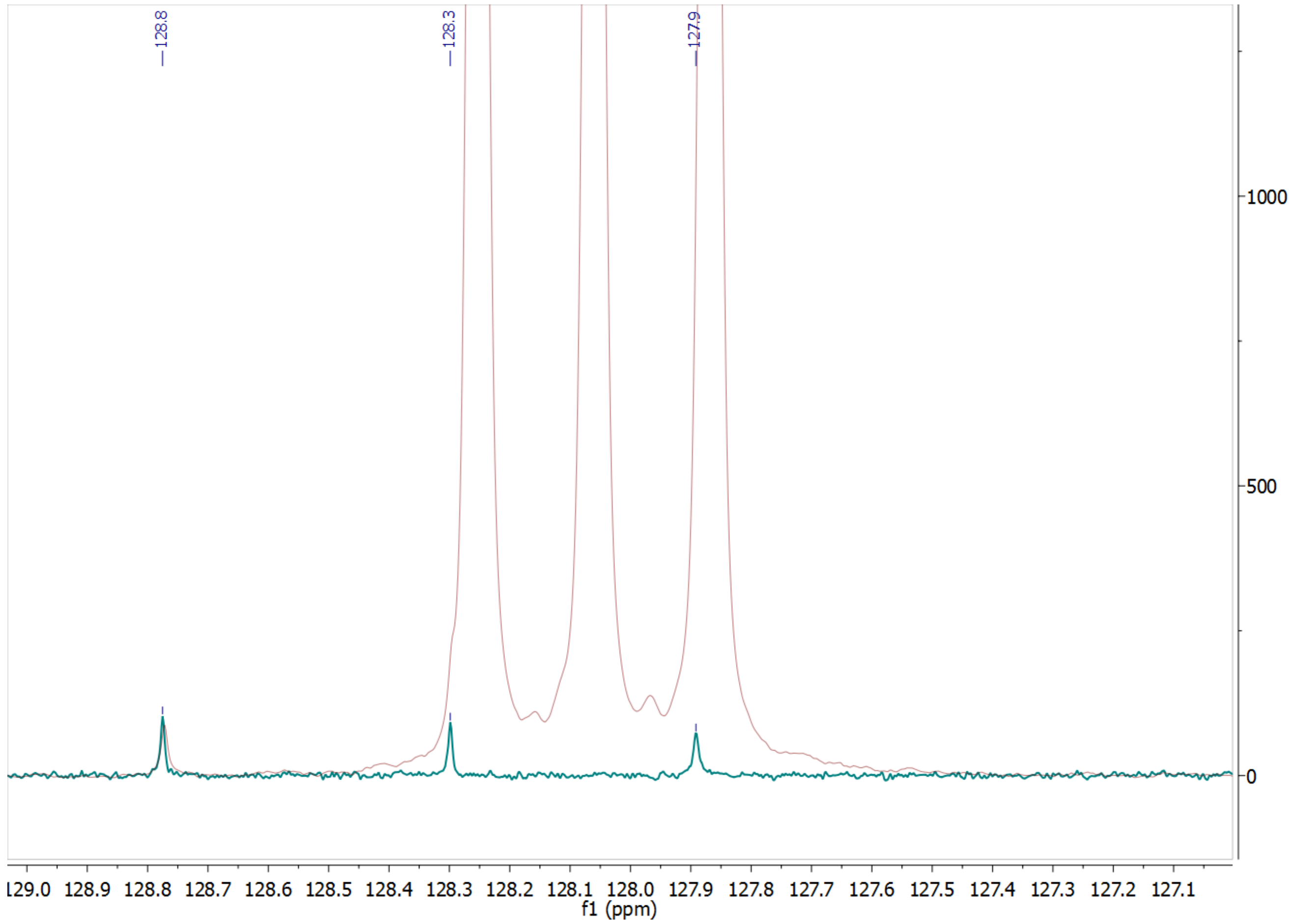

^{13}C-NMR (in red) is superimposed by DEPT-135 (in turquois). The peak of 128.3 and 127.9 are only visible in the DEPT-135 experiment, as the signal are overlain by C_6D_6.

High Resolution Mass Spectrometry Report

Sample Name	**Ba604 chr1_1**	Instrument	maXis 4G
Comment		Method	ms_nocolumn_300-600_pos.m

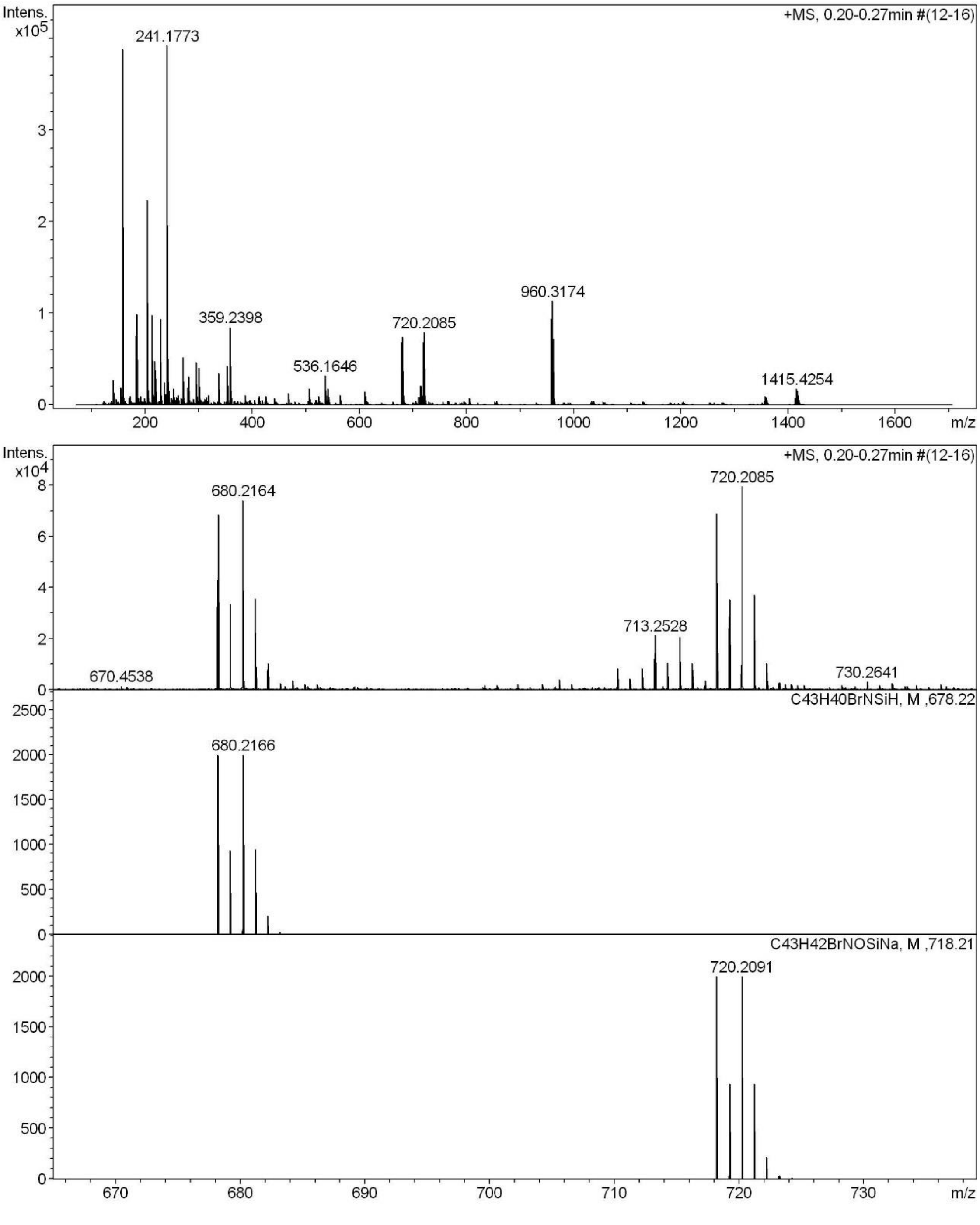

Bruker Compass DataAnalysis 4.0 Acquisition Date 22.05.2019 12:06:45 Page 1 of 3

High Resolution Mass Spectrometry Report

Measured m/z vs. theoretical m/z

Meas. m/z	#	Formula	Score	m/z	err [mDa]	err [ppm]	mSigma	rdb	e⁻ Conf	z
678.2175	1	C 43 H 41 Br N Si	100.00	678.2186	1.2	1.7	20.2	24.5	even	1+
718.2098	1	C 43 H 42 Br N Na O Si	100.00	718.2111	1.3	1.8	10.3	23.5	even	

Mass list

#	m/z	I %	I
1	140.9621	7.0	27568
2	155.0470	4.8	18827
3	158.0968	98.9	388055
4	159.0998	12.2	47933
5	161.1076	2.1	8376
6	172.1120	2.4	9384
7	183.0779	19.2	75388
8	185.1146	25.4	99605
9	186.1182	2.2	8668
10	187.1224	2.1	8162
11	191.9823	2.5	9926
12	205.0599	57.0	223642
13	206.0630	4.3	16694
14	213.1458	25.0	97935
15	214.0891	2.6	10377
16	214.1490	3.0	11861
17	217.1046	2.3	9202
18	219.1950	12.2	47803
19	229.0499	2.8	11107
20	229.1406	23.9	93941
21	230.1440	2.7	10424
22	236.0711	6.3	24631
23	236.2214	2.3	9165
24	239.0706	2.9	11571
25	239.1250	2.2	8781
26	241.1773	100.0	392424
27	242.1803	12.9	50756
28	245.0777	4.1	16033
29	253.1403	4.5	17634
30	259.0928	2.2	8602
31	259.8950	2.0	7942
32	262.0603	2.7	10468
33	267.0299	2.1	8093
34	271.1145	4.1	16113
35	271.1872	13.2	51984
36	272.1906	2.0	7903
37	273.2051	2.1	8167
38	279.0927	5.0	19458
39	281.1715	3.3	12908
40	282.0053	8.0	31241
41	282.2781	3.3	13139
42	295.1874	12.0	46948
43	297.2393	2.4	9303
44	301.1403	10.2	40121
45	315.2132	2.0	7848
46	315.2520	2.2	8596
47	318.1225	2.8	10923
48	337.2341	8.7	34308
49	353.1445	10.8	42464
50	354.1480	3.2	12657
51	354.2843	5.5	21440
52	359.2398	21.7	85169
53	360.2429	4.0	15877
54	387.1296	2.7	10650
55	412.3774	2.0	7794
56	413.2651	2.4	9416
57	425.2867	2.4	9587
58	441.2963	2.0	7870
59	467.3154	3.2	12395
60	506.5284	4.7	18255
61	524.2757	2.6	10040

High Resolution Mass Spectrometry Report

#	m/z	I %	I
62	536.1646	8.2	32222
63	537.1649	4.2	16599
64	538.1628	2.7	10486
65	541.1197	4.4	17459
66	542.1207	2.2	8592
67	564.2680	2.8	10995
68	610.1827	3.9	15356
69	611.1835	2.4	9551
70	678.2175	17.5	68668
71	679.2203	8.6	33576
72	680.2164	18.9	74174
73	681.2183	9.1	35689
74	682.2199	2.7	10435
75	710.2431	2.2	8703
76	712.2410	2.2	8607
77	713.2528	5.5	21394
78	714.2560	2.7	10608
79	715.2527	5.3	20691
80	716.2549	2.7	10460
81	718.2098	17.5	68803
82	719.2124	9.1	35622
83	720.2085	20.3	79475
84	721.2106	9.5	37249
85	722.2120	2.7	10514
86	804.3763	2.0	8031
87	958.3178	24.1	94464
88	959.3207	17.0	66597
89	960.3174	28.9	113336
90	961.3192	18.4	72029
91	962.3207	6.8	26793
92	963.3226	2.0	7690
93	1357.4225	2.5	9619
94	1358.4239	2.2	8653
95	1359.4227	2.2	8568
96	1414.4288	2.0	7917
97	1415.4254	4.5	17511
98	1416.4265	4.1	15989
99	1417.4250	4.1	15966
100	1418.4254	2.7	10740

Acquisition Parameter

**General**	Fore Vacuum	2.60e+000 mBar	High Vacuum	1.19e-007 mBar	Source Type	ESI
	Scan Begin	75 m/z	Scan End	1700 m/z	Ion Polarity	Positive
**Source**	Set Nebulizer	2.0 Bar	Set Capillary	4500 V	Set Dry Gas	8.0 l/min
	Set Dry Heater	200 °C	Set End Plate Offset	-500 V		
**Quadrupole**	Set Ion Energy (MS only)	4.0 eV				
**Coll. Cell**	Collision Energy	8.0 eV	Set Collision Cell RF	350.0 Vpp		
**Ion Cooler**	Set Ion Cooler Transfer Time	75.0 µs	Set Ion Cooler Pre Pulse Storage Time	10.0 µs		

5.30 Compound **35**

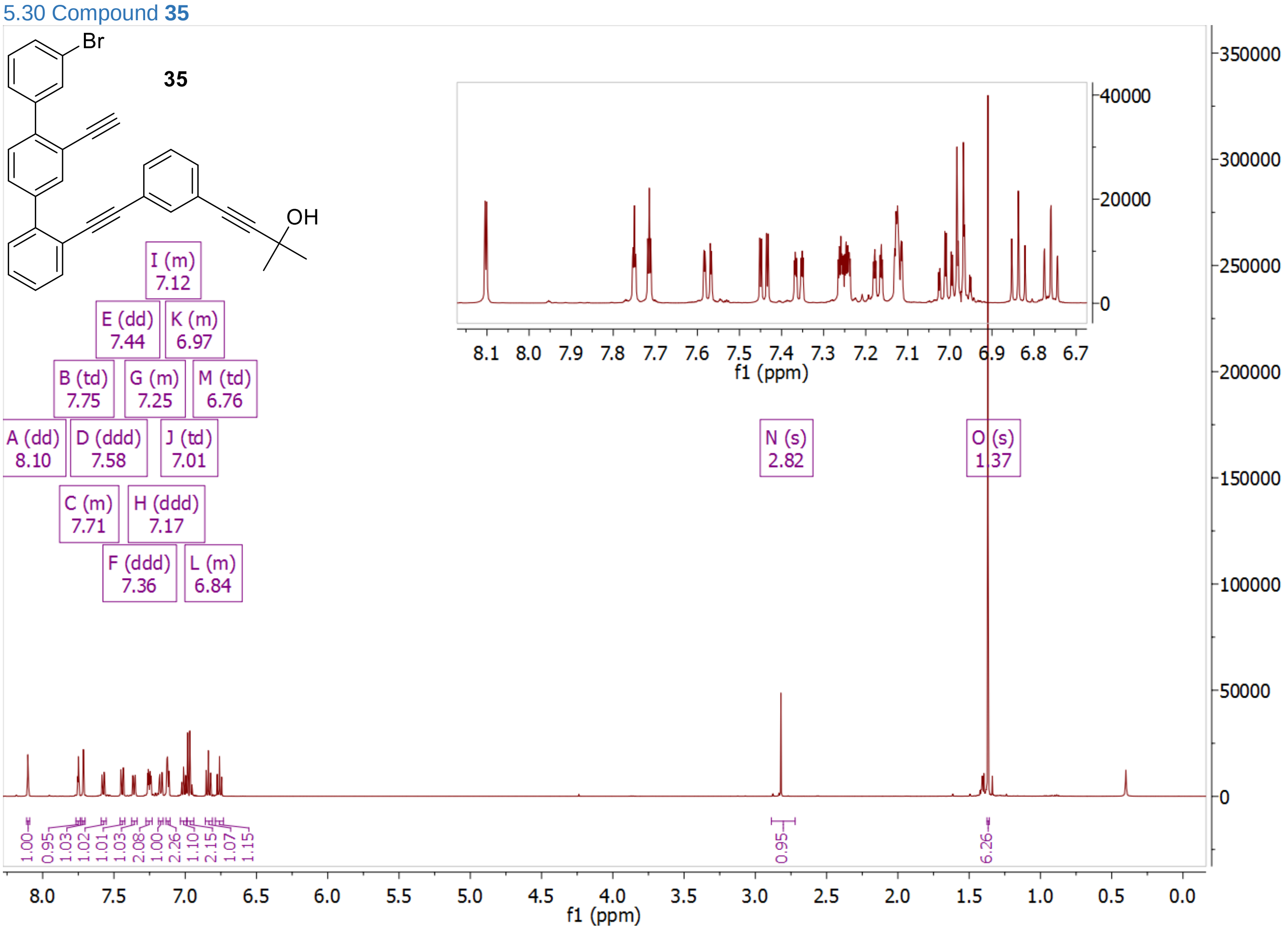

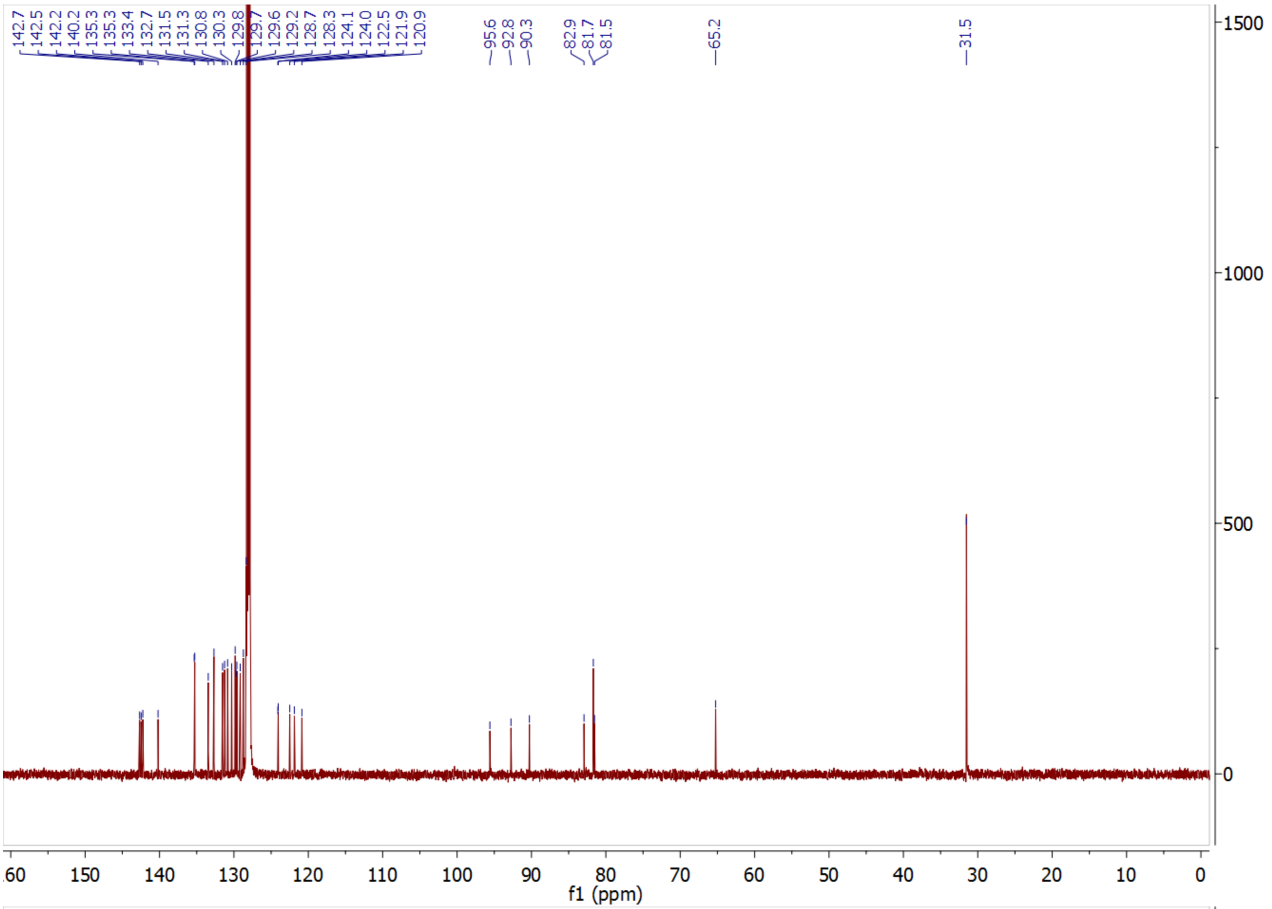

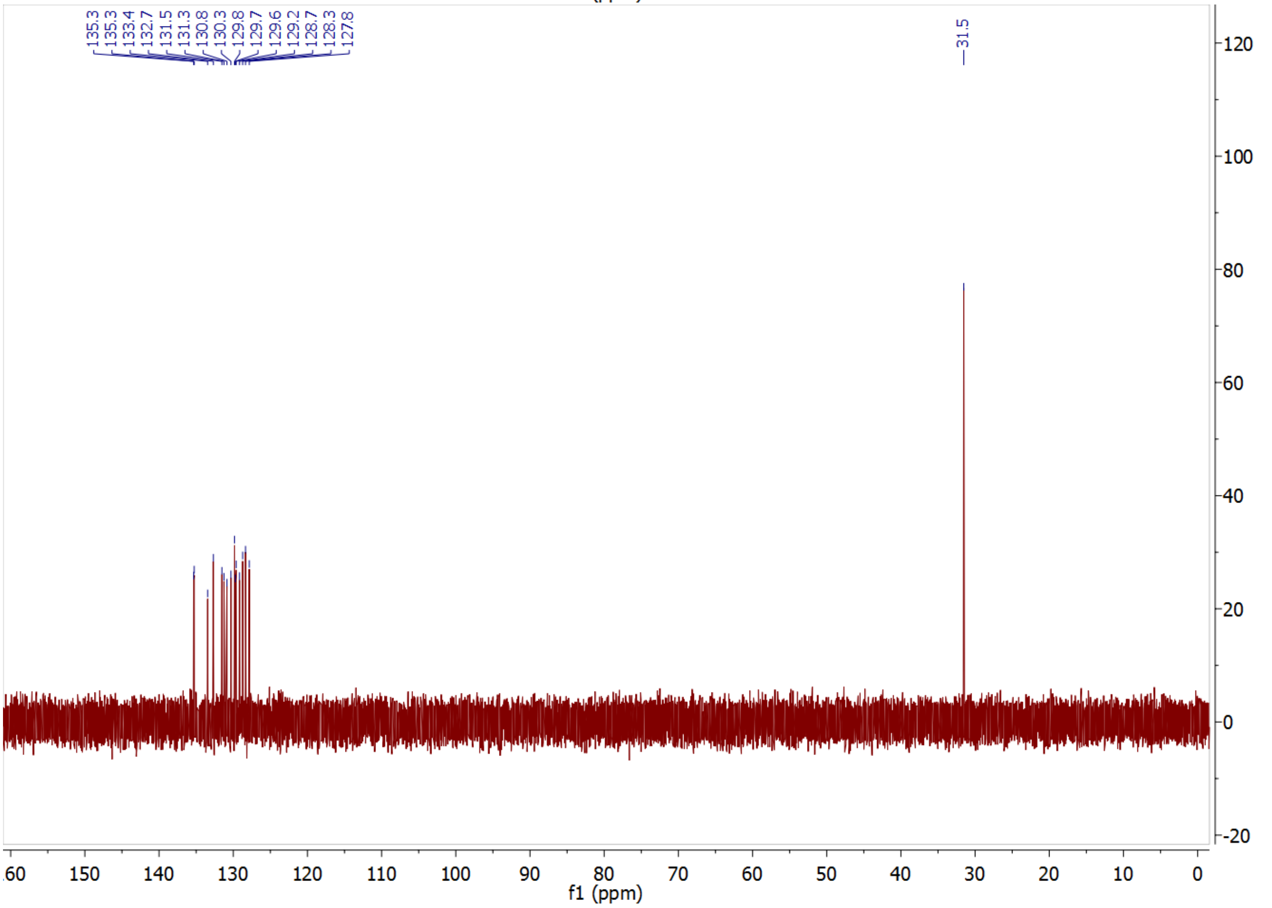

DEPT-135: $^{1}J_{CH}$ coupling= 145 Hz

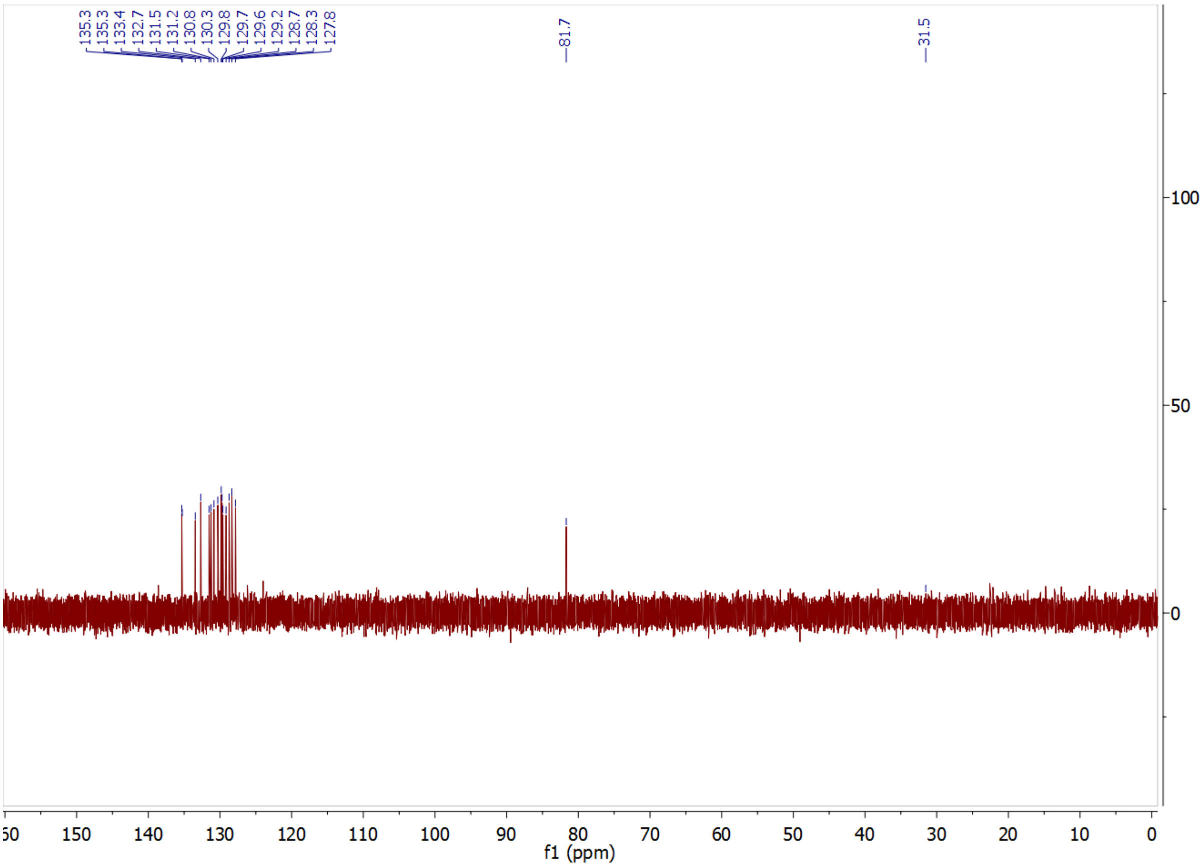

DEPT-135: $^{1}J_{CH}$ coupling= 200 Hz

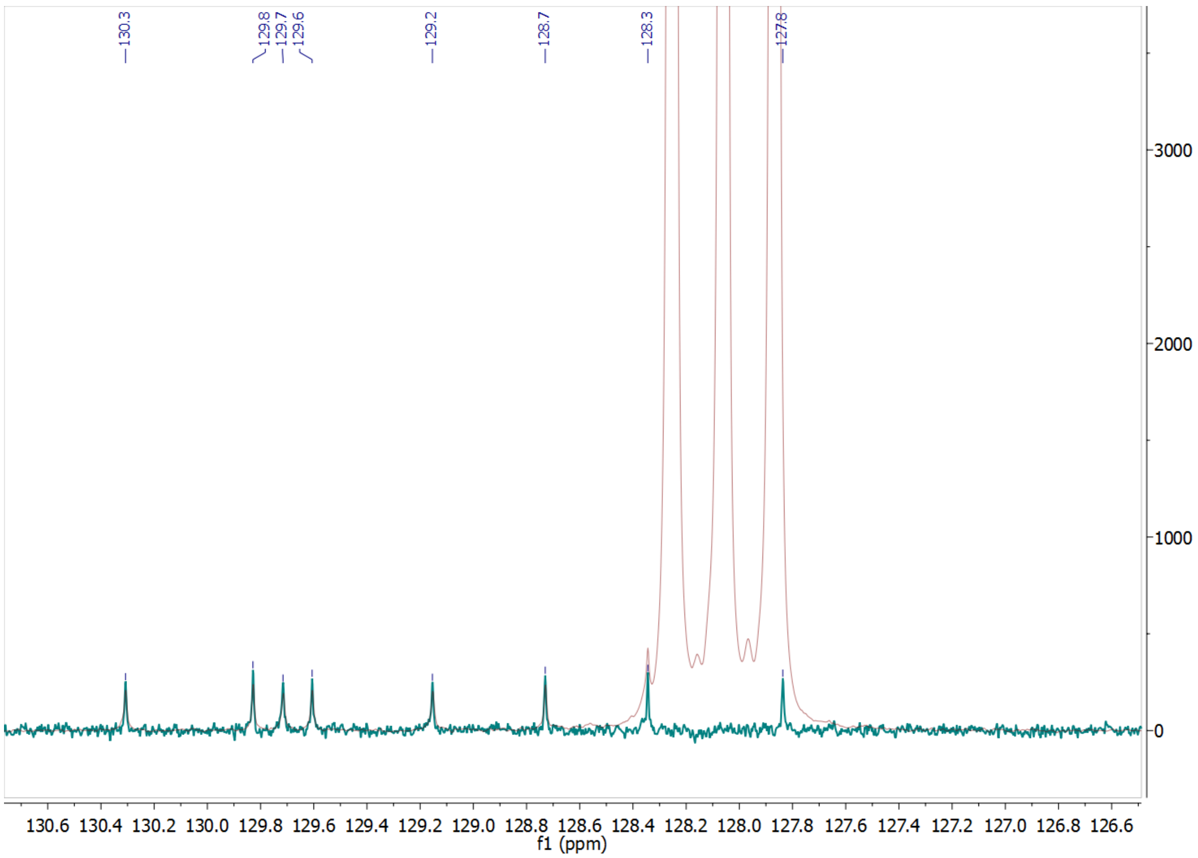

^{13}C-NMR (in red) is superimposed by DEPT-135 (in turquois). The peak of 127.8 is only visible in the DEPT-135 experiment, as the signal is overlain by C_6D_6.

High Resolution Mass Spectrometry Report

Sample Name **Ba607 SE1_1**
Comment

Instrument maXis 4G
Method ms_nocolumn_300-600_pos.m

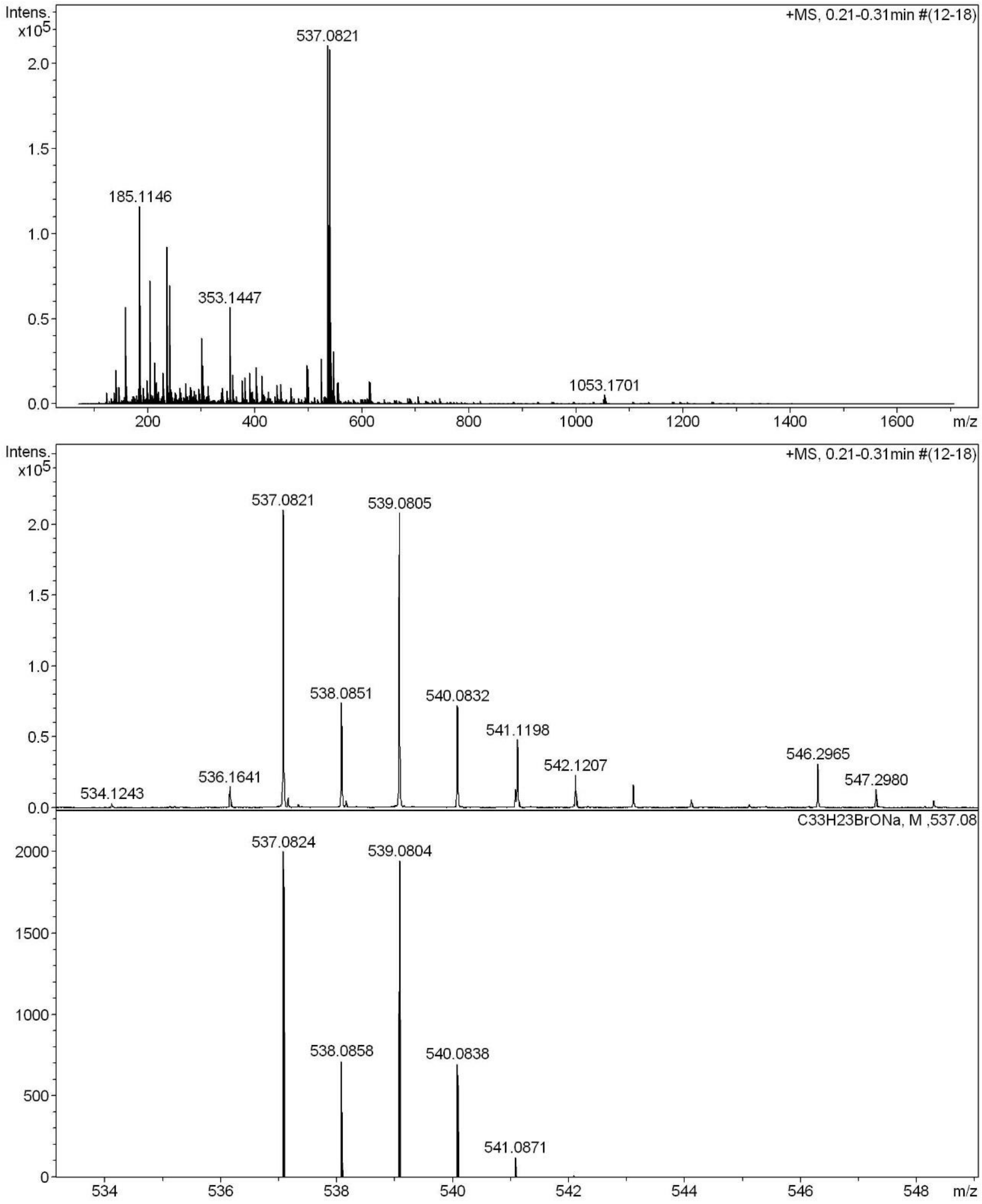

High Resolution Mass Spectrometry Report

Measured m/z vs. theoretical m/z

Meas. m/z	#	Formula	Score	m/z	err [mDa]	err [ppm]	mSigma	rdb	e⁻ Conf	z
537.0821	1	C 33 H 23 Br Na O	100.00	537.0824	0.4	0.7	15.0	21.5	even	1+

Mass list

#	m/z	I %	I
1	123.0925	3.2	6820
2	137.1079	3.2	6663
3	138.0918	3.3	7019
4	140.9622	9.6	20322
5	147.0920	4.8	10035
6	158.0967	27.0	56867
7	159.0999	3.8	7936
8	161.1074	5.2	10938
9	183.0778	4.2	8875
10	185.1146	55.2	116436
11	186.1179	5.3	11164
12	187.1224	5.0	10636
13	191.9823	4.6	9619
14	199.1300	6.6	13871
15	201.1383	2.9	6038
16	203.8933	4.1	8580
17	205.0597	34.4	72542
18	206.0631	2.8	5849
19	213.1457	11.5	24323
20	214.0892	4.5	9464
21	217.1043	6.2	13096
22	219.9613	3.5	7366
23	229.0500	3.5	7290
24	229.1405	8.8	18604
25	236.0712	43.8	92295
26	237.0742	5.3	11086
27	239.0882	3.4	7068
28	241.1769	33.1	69753
29	242.1804	4.4	9181
30	242.2837	3.4	7072
31	243.1349	3.6	7610
32	244.8681	2.8	6001
33	245.0774	3.5	7428
34	251.0525	3.3	6993
35	253.1404	2.7	5747
36	259.8952	3.0	6423
37	261.1302	4.5	9510
38	262.0602	3.3	7021
39	271.1873	6.0	12644
40	279.0747	2.7	5679
41	279.0923	4.9	10269
42	282.0054	4.0	8377
43	286.1047	3.9	8231
44	295.1873	4.2	8856
45	297.2390	2.9	6050
46	301.1402	18.4	38805
47	302.0998	10.9	23054
48	302.1435	3.6	7578
49	304.2603	5.0	10625
50	305.1564	4.0	8451
51	313.0167	5.1	10658
52	337.2338	3.3	7057
53	340.1727	4.5	9524
54	349.1824	3.7	7847
55	353.1447	27.1	57013
56	353.2652	4.3	9056
57	354.1479	7.7	16266
58	359.2395	8.1	17085
59	377.2042	6.6	13938
60	381.2966	7.5	15756
61	391.2187	8.9	18703
62	392.2210	2.7	5659

High Resolution Mass Spectrometry Report

#	m/z	I %	I
63	393.2101	3.1	6556
64	393.2966	3.3	6990
65	395.3623	3.6	7541
66	403.2562	10.2	21544
67	404.2584	3.3	7011
68	413.2654	7.9	16645
69	425.2135	3.5	7443
70	425.2865	3.6	7690
71	441.2966	5.3	11174
72	449.3594	5.6	11738
73	467.1013	4.7	9801
74	467.3156	3.9	8283
75	497.0890	10.9	22943
76	498.0923	3.7	7800
77	499.0873	9.9	20943
78	500.0903	3.8	8047
79	523.3231	12.7	26747
80	524.3267	3.8	7984
81	536.1641	7.1	15000
82	537.0821	100.0	210752
83	537.1655	3.6	7579
84	538.0851	35.2	74231
85	539.0805	98.8	208318
86	540.0832	34.4	72456
87	541.0861	6.2	13118
88	541.1198	23.0	48380
89	542.1207	11.1	23377
90	543.1184	7.9	16623
91	544.1183	2.9	6083
92	546.2965	14.9	31298
93	547.2980	6.2	13171
94	553.0552	5.8	12242
95	555.0538	6.1	12888
96	613.1122	6.4	13462
97	614.0272	3.2	6686
98	614.1160	2.8	5875
99	615.1109	6.1	12844
100	616.0252	3.2	6652

Acquisition Parameter

**General**	Fore Vacuum	2.60e+000 mBar	High Vacuum	1.19e-007 mBar	Source Type	ESI
	Scan Begin	75 m/z	Scan End	1700 m/z	Ion Polarity	Positive
**Source**	Set Nebulizer	2.0 Bar	Set Capillary	4500 V	Set Dry Gas	8.0 l/min
	Set Dry Heater	200 °C	Set End Plate Offset	-500 V		
**Quadrupole**	Set Ion Energy (MS only)	4.0 eV				
**Coll. Cell**	Collision Energy	8.0 eV	Set Collision Cell RF	350.0 Vpp		
**Ion Cooler**	Set Ion Cooler Transfer Time	75.0 µs	Set Ion Cooler Pre Pulse Storage Time	10.0 µs		

5.31 Compound **36**

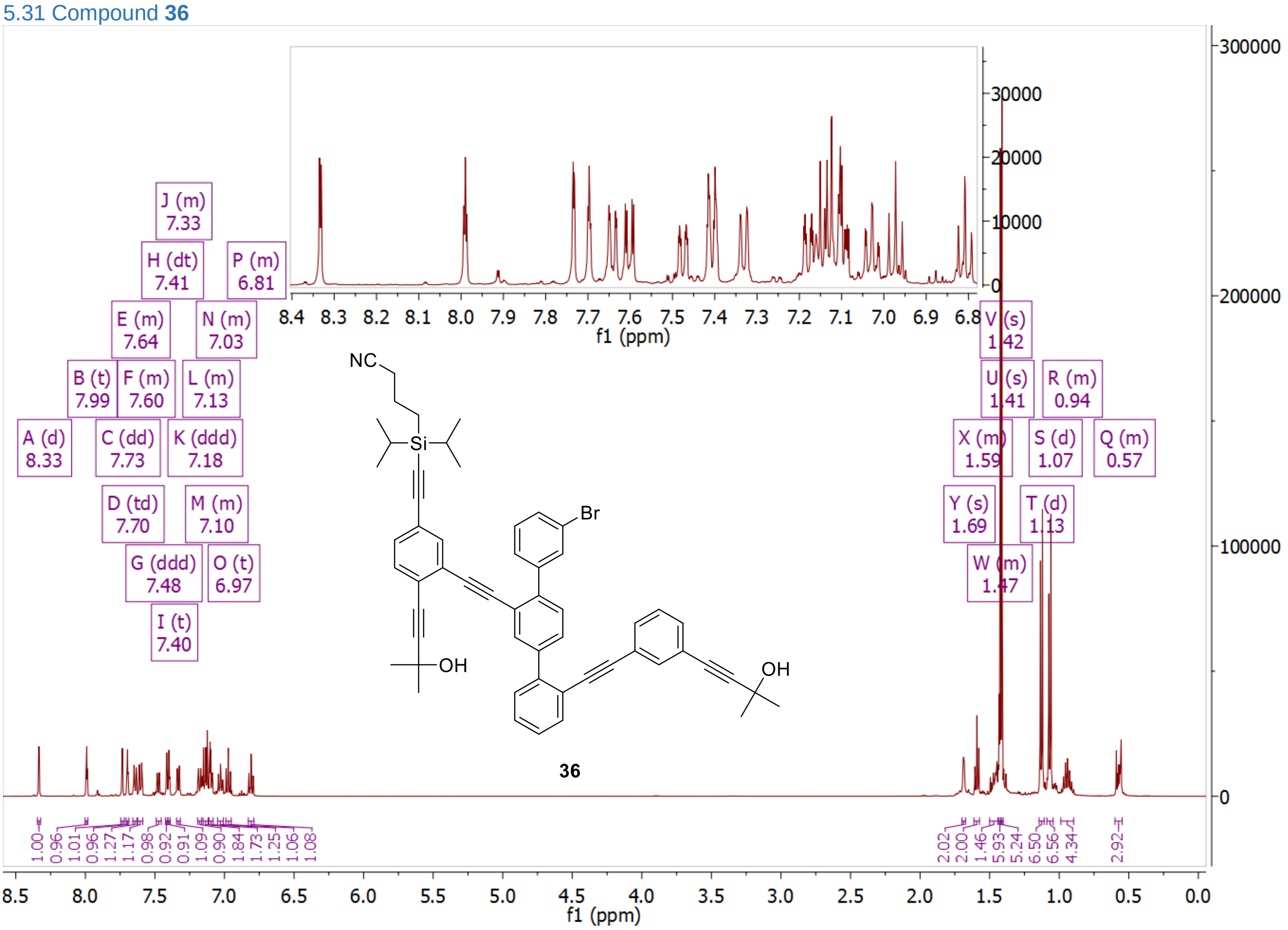

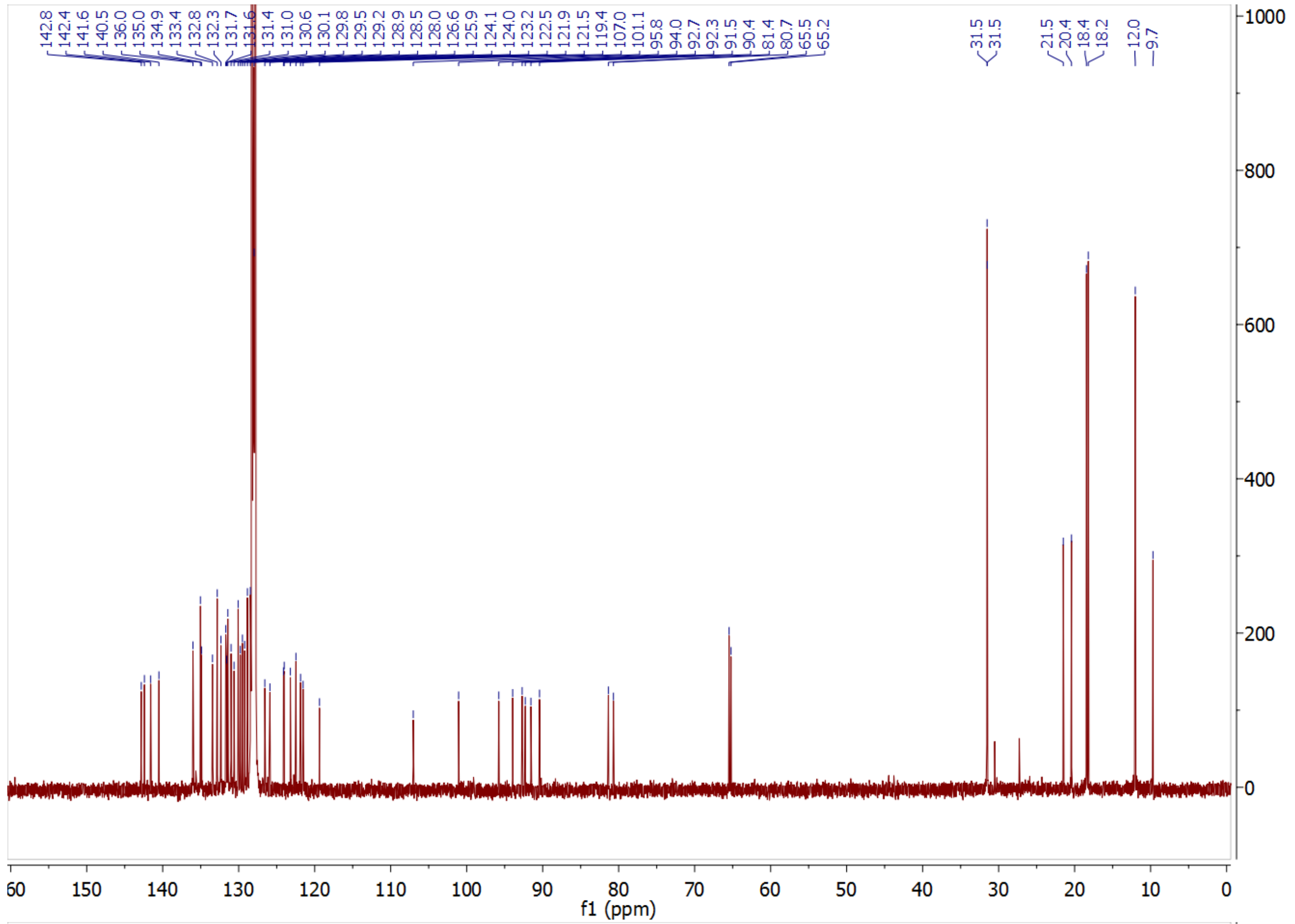
142.8 142.4 141.6 140.5 136.0 135.0 134.9 133.4 132.8 132.3 131.7 131.6 131.4 131.0 130.6 130.1 129.8 129.5 129.2 128.9 128.5 128.0 126.6 125.9 124.1 124.0 123.2 122.5 121.9 121.5 119.4 107.0 101.1 95.8 94.0 92.7 92.3 91.5 90.4 81.4 80.7 65.5 65.2
31.5 31.5
21.5 20.4 18.4 18.2
12.0
9.7
f1 (ppm)

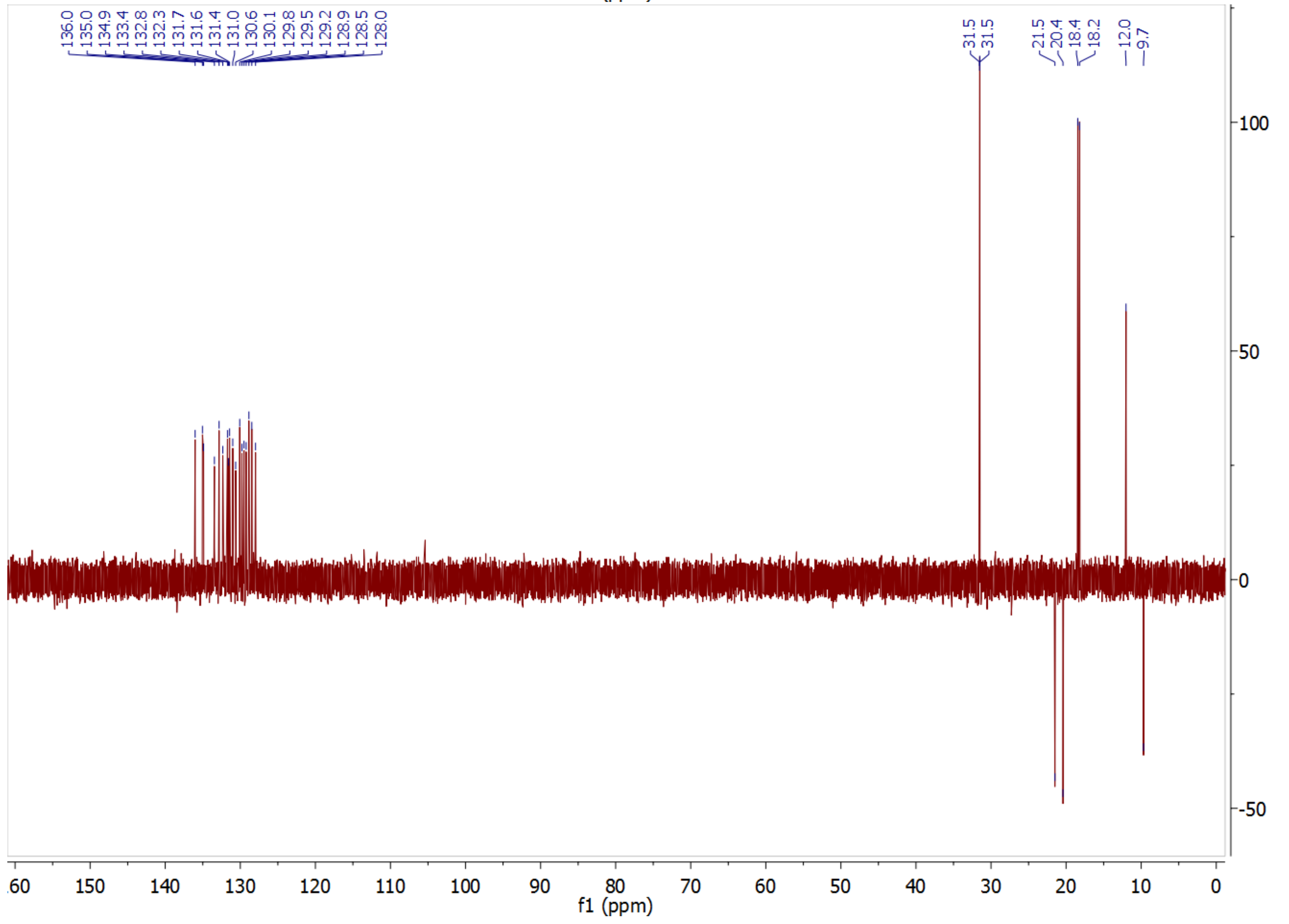
136.0 135.0 134.9 133.4 132.8 132.3 131.7 131.6 131.4 131.0 130.6 130.1 129.8 129.5 129.2 128.9 128.5 128.0
31.5 31.5
21.5 20.4 18.4 18.2
12.0
9.7
f1 (ppm)

High Resolution Mass Spectrometry Report

Sample Name **Linda Bannwart / Ba 610 chr1#1**
Comment

Instrument maXis 4G
Method 23 Direct_pos_higher.m

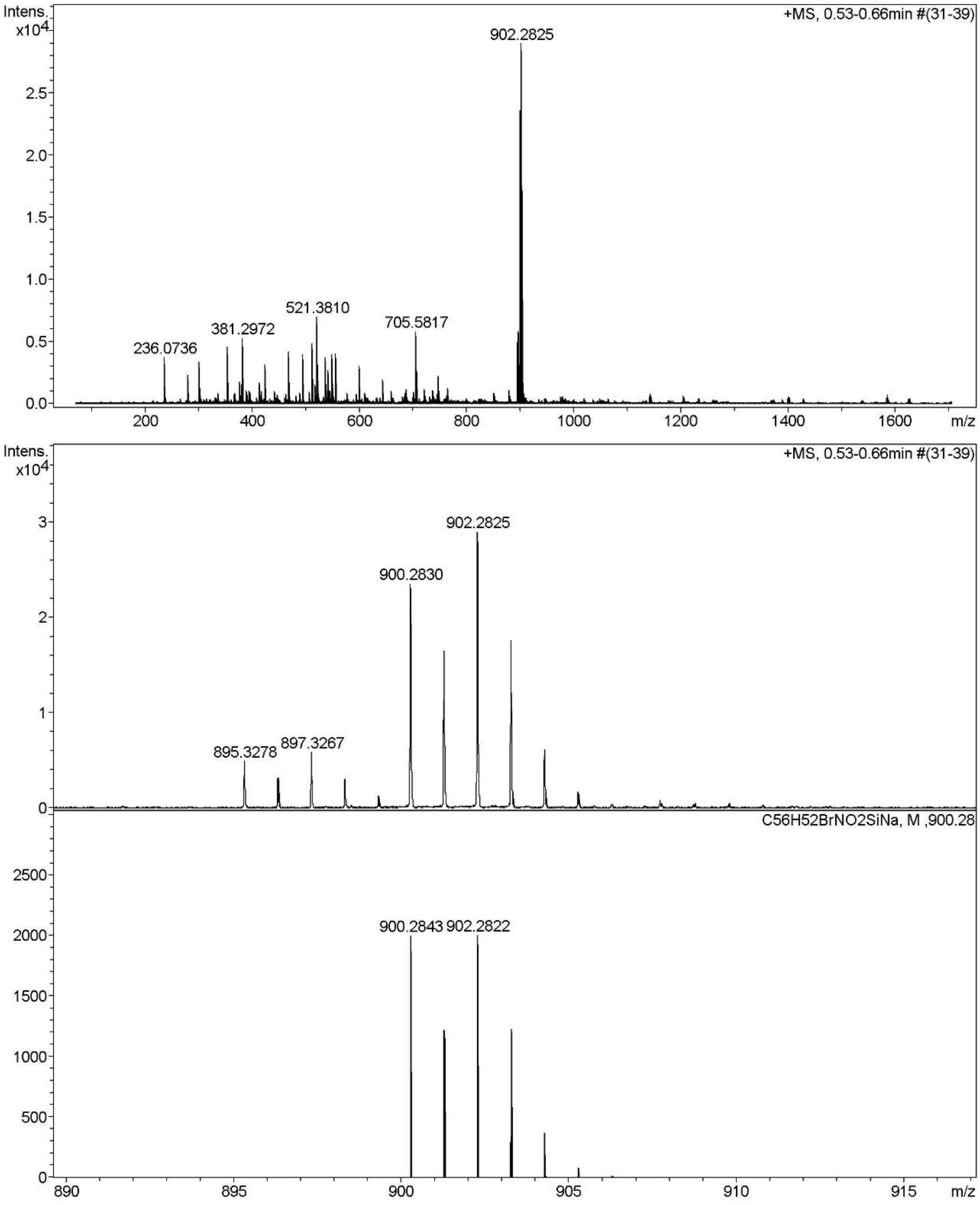

Bruker Compass DataAnalysis 4.0 Acquisition Date 04.06.2019 09:05:25 Page 1 of 3

High Resolution Mass Spectrometry Report

Measured m/z vs. theoretical m/z

Meas. m/z	#	Formula	Score	m/z	err [mDa]	err [ppm]	mSigma	rdb	e⁻ Conf	z
900.2830	1	C 56 H 52 Br N Na O 2 Si	100.00	900.2843	1.2	1.4	13.9	31.5	even	1+

Mass list

#	m/z	I %	I
1	236.0736	12.9	3743
2	279.0930	8.0	2321
3	301.0749	11.6	3364
4	353.2660	15.8	4572
5	354.2691	4.1	1182
6	377.2044	6.1	1779
7	379.1941	5.3	1526
8	381.2972	18.3	5296
9	382.3005	3.7	1062
10	388.2061	3.7	1079
11	413.2662	5.8	1695
12	423.2199	10.9	3155
13	467.1013	6.0	1742
14	467.2464	14.6	4220
15	493.3495	13.7	3964
16	494.3531	4.0	1153
17	511.2725	16.9	4902
18	512.2755	4.1	1184
19	516.4259	5.3	1532
20	521.3810	24.1	6995
21	522.3850	7.0	2035
22	536.1649	13.0	3762
23	537.1652	6.9	2010
24	538.1640	4.0	1165
25	541.1206	9.3	2687
26	542.1211	5.6	1631
27	543.1186	3.8	1105
28	549.4121	13.8	3987
29	550.4157	4.7	1357
30	555.2983	14.0	4050
31	556.3026	4.4	1274
32	599.3238	10.6	3058
33	643.3507	6.8	1960
34	687.3769	4.1	1182
35	705.5817	20.1	5820
36	706.5846	9.5	2761
37	721.5759	4.0	1152
38	736.5419	3.7	1059
39	746.3422	7.7	2225
40	747.3440	4.9	1429
41	764.5726	4.4	1277
42	879.6876	3.8	1087
43	895.3278	17.1	4952
44	896.3308	11.1	3217
45	897.3267	20.3	5894
46	898.3303	10.8	3138
47	899.3301	4.4	1271
48	900.2830	81.3	23560
49	901.2857	57.0	16520
50	902.2825	100.0	28982
51	903.2853	60.6	17561
52	904.2865	21.1	6114
53	905.2890	5.9	1719

Acquisition Parameter

**General**	Fore Vacuum	2.68e+000 mBar	High Vacuum	1.21e-007 mBar	Source Type	ESI
	Scan Begin	75 m/z	Scan End	1700 m/z	Ion Polarity	Positive
**Source**	Set Nebulizer	0.4 Bar	Set Capillary	3600 V	Set Dry Gas	4.0 l/min
	Set Dry Heater	180 °C	Set End Plate Offset	-500 V		

High Resolution Mass Spectrometry Report

**Quadrupole**	Set Ion Energy (MS only)	4.0 eV		
**Coll. Cell**	Collision Energy	8.0 eV	Set Collision Cell RF	500.0 Vpp
**Ion Cooler**	Set Ion Cooler Transfer Time	80.0 µs	Set Ion Cooler Pre Pulse Storage Time	18.0 µs

5.32 Compound **37**

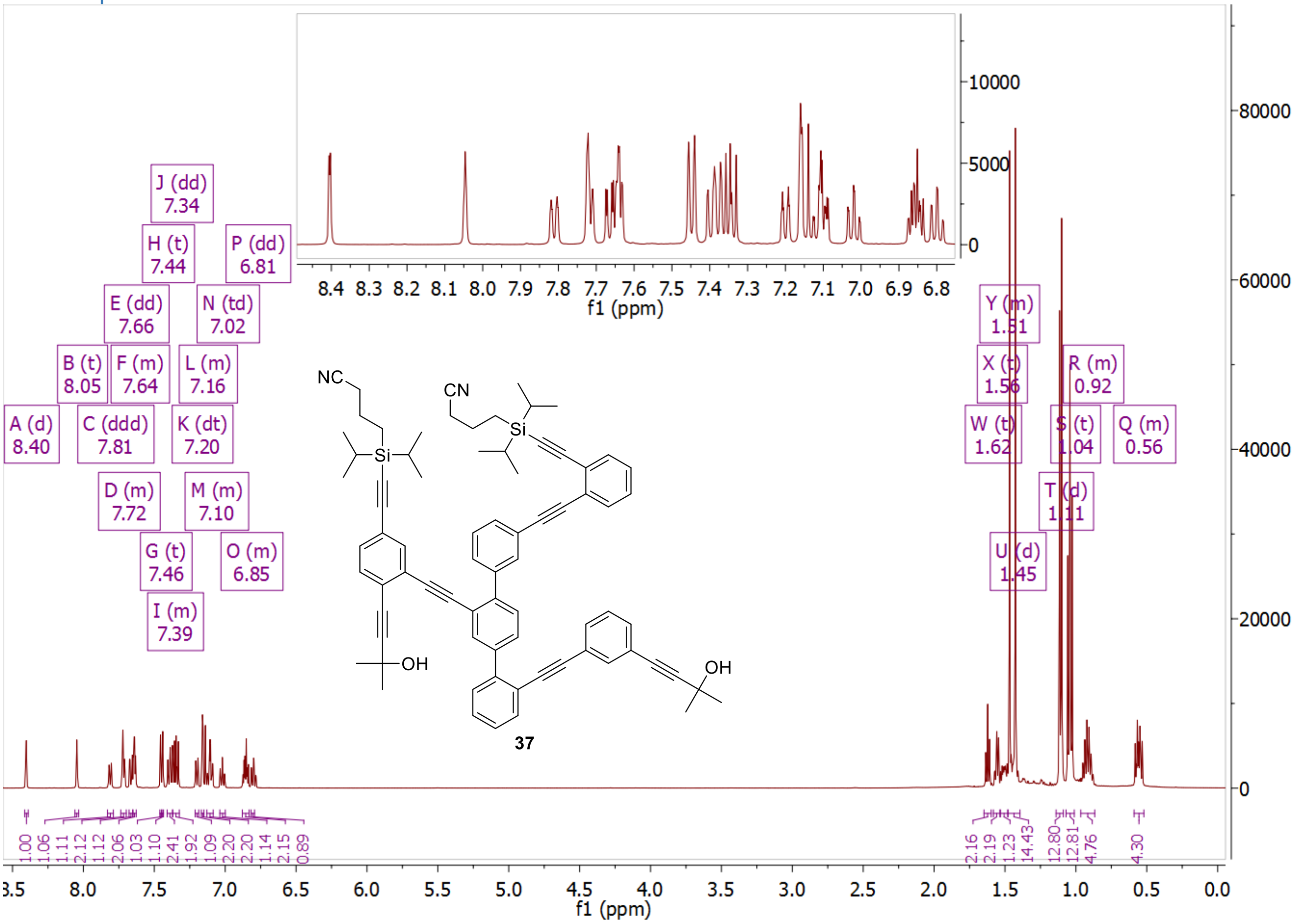

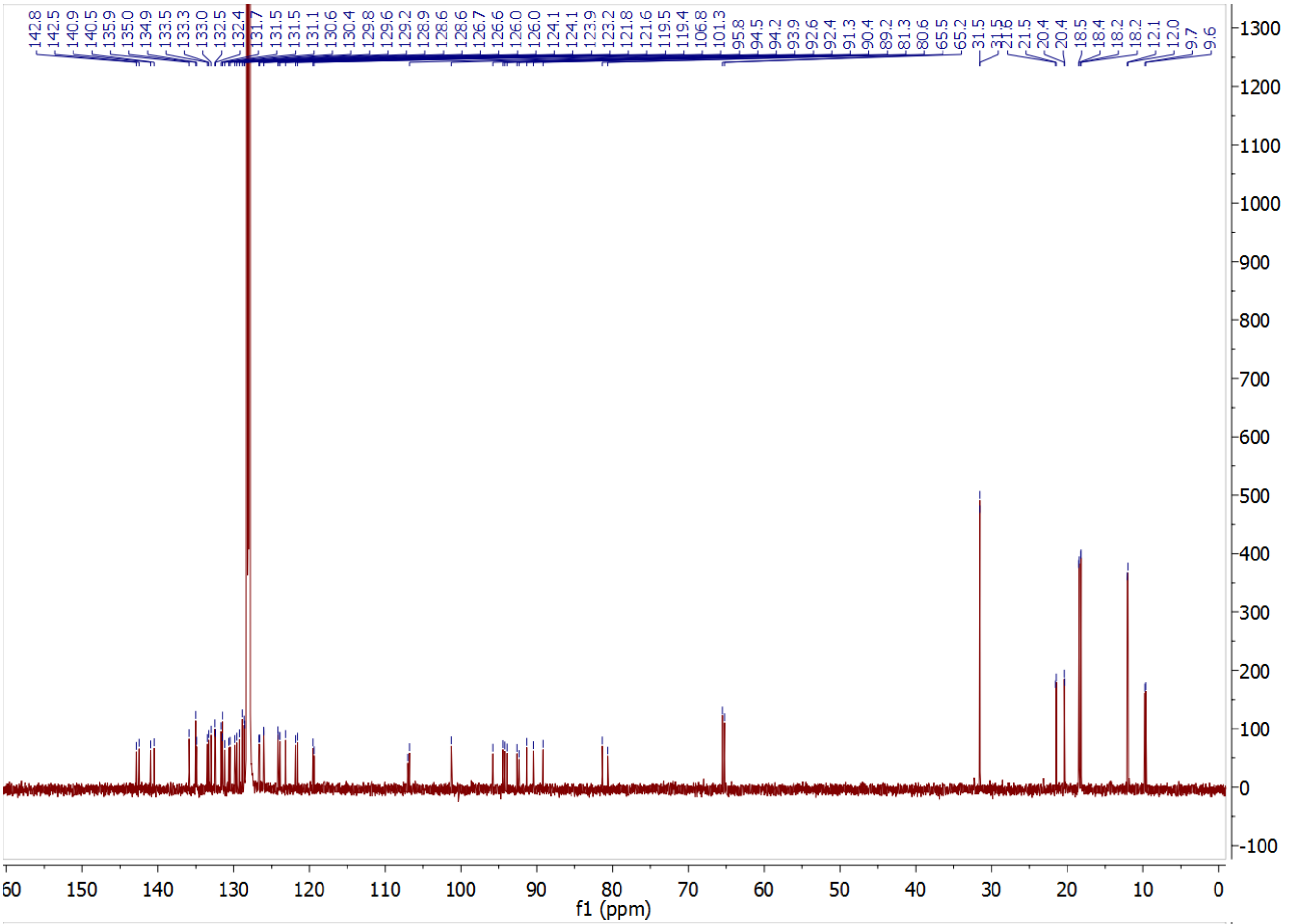
142.8 142.5 140.9 140.5 135.9 135.0 134.9 133.5 133.3 133.0 132.5 132.4 131.7 131.5 131.5 131.1 130.6 130.4 129.8 129.6 129.2 128.9 128.6 126.7 126.6 126.0 126.0 124.1 124.1 123.9 123.2 121.8 121.6 119.5 119.4 106.8 101.3 95.8 94.5 94.2 93.9 92.6 92.4 91.3 90.4 89.2 81.3 80.6 65.5 65.2 31.5 31.5 21.6 21.5 20.4 20.4 18.5 18.4 18.2 18.2 12.1 12.0 9.7 9.6
f1 (ppm)

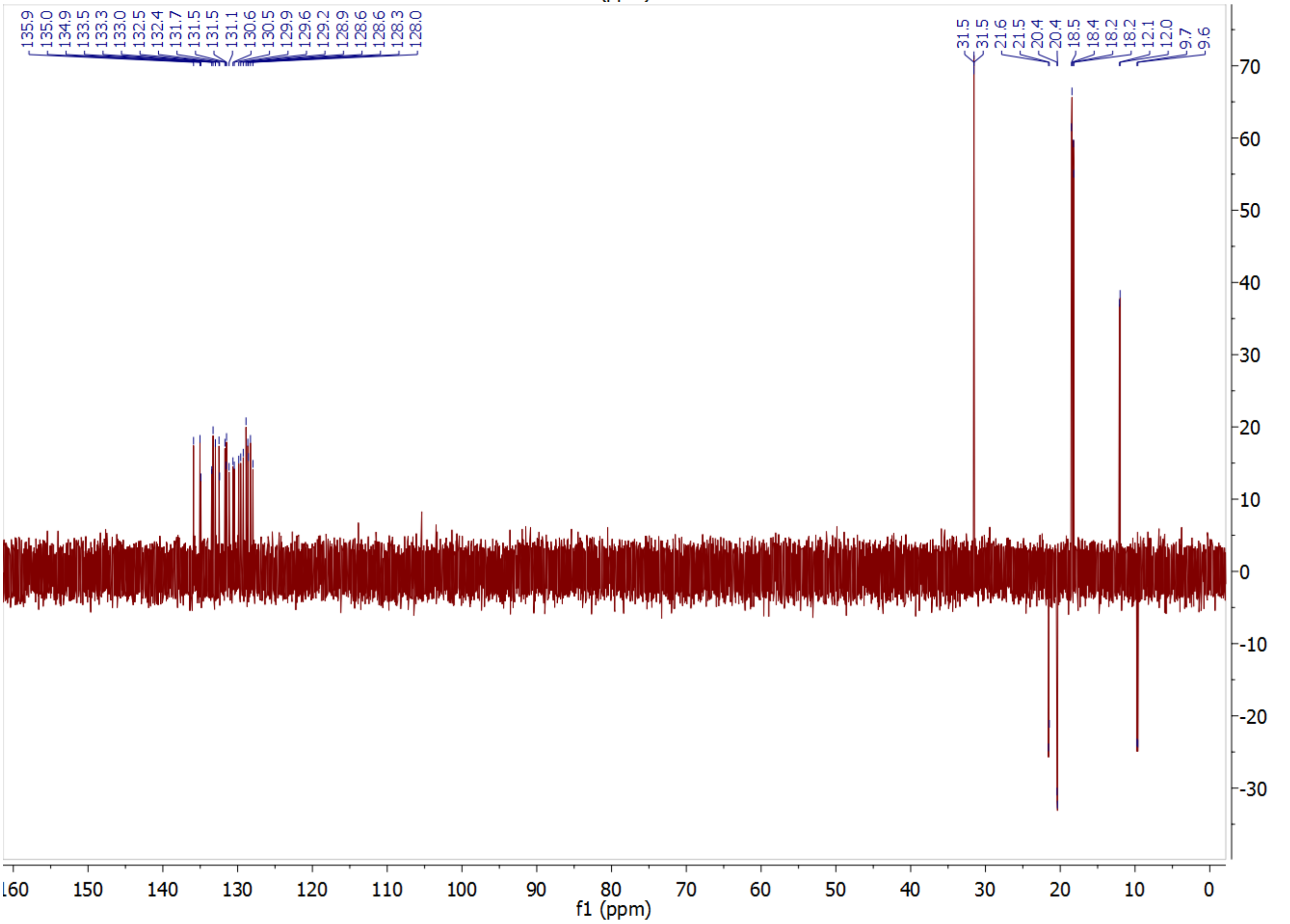
135.9 135.0 134.9 133.5 133.3 133.0 132.5 132.4 131.7 131.5 131.5 131.1 130.6 130.5 129.9 129.6 129.2 128.9 128.6 128.6 128.3 128.0
31.5 31.5 21.6 21.5 20.4 20.4 18.5 18.4 18.2 18.2 12.1 12.0 9.7 9.6
f1 (ppm)

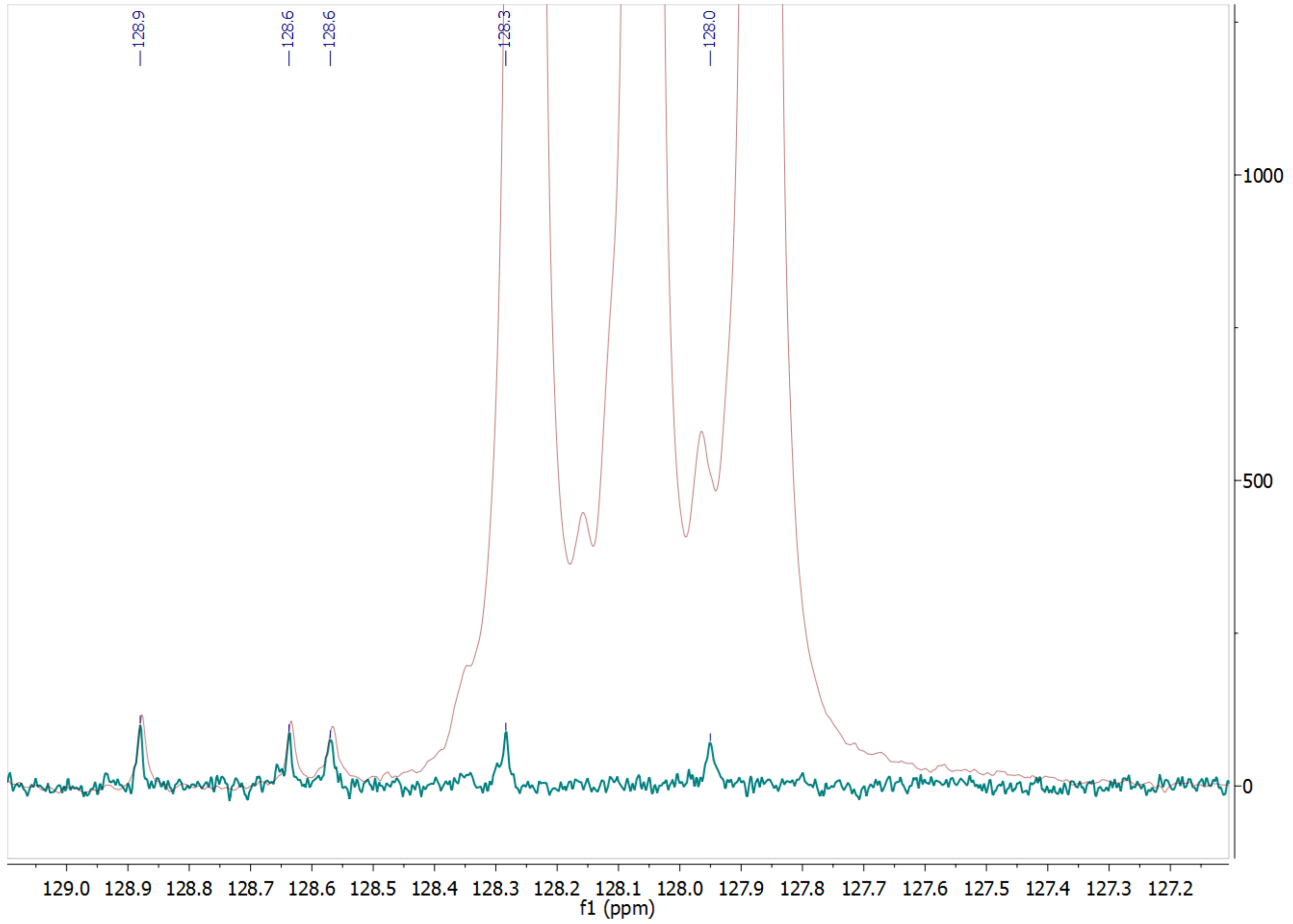

^{13}C-NMR (in red) is superimposed by DEPT-135 (in turquois).The peaks of 128.0 and 128.3 are only visible in the DEPT-135 experiment, as the signals are overlain by C_6D_6.

High Resolution Mass Spectrometry Report

Sample Name **Linda Bannwart / Ba 613 chr1#1**
Comment

Instrument maXis 4G
Method 23 Direct_pos_higher.m

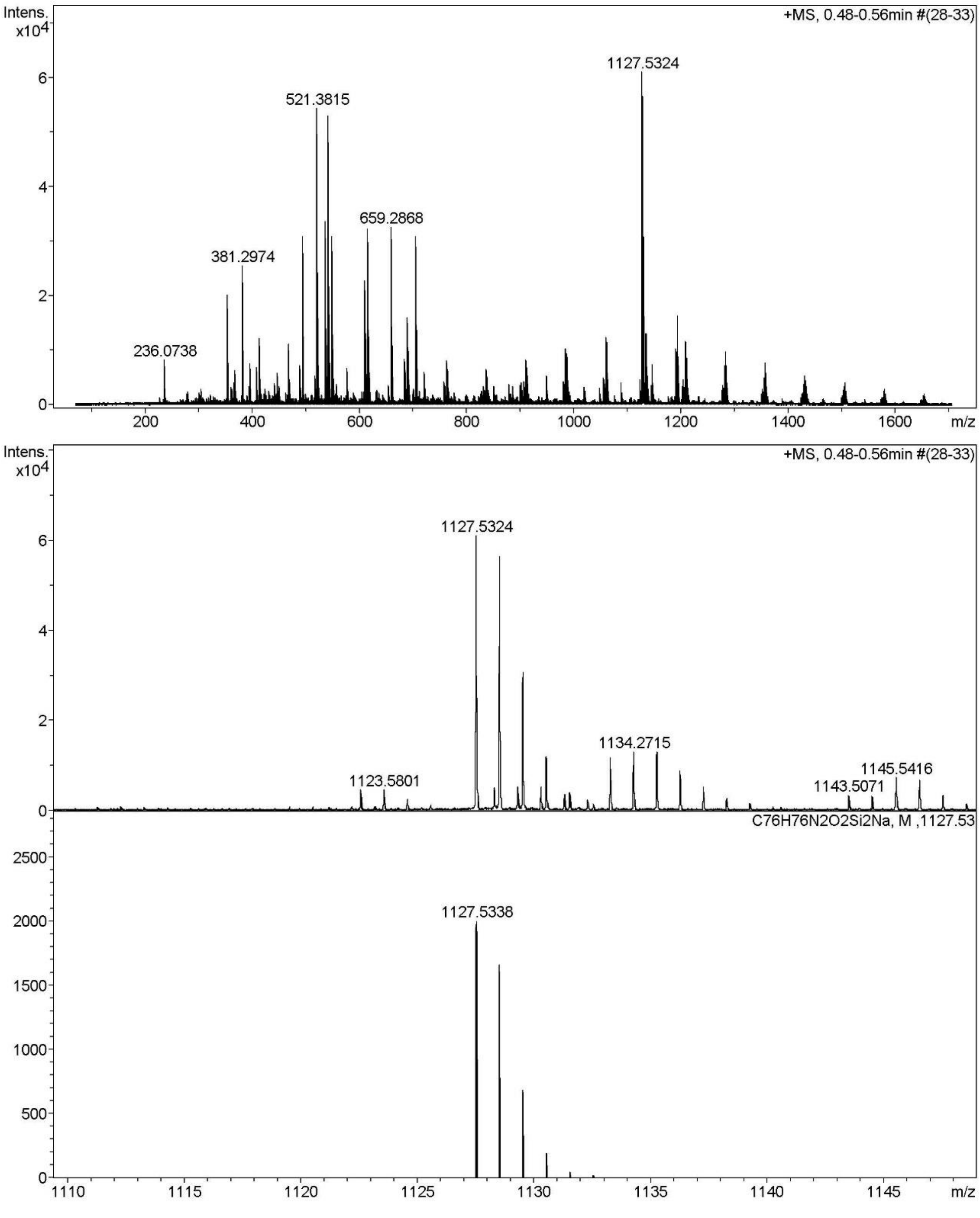

Bruker Compass DataAnalysis 4.0 Acquisition Date 04.06.2019 08:41:30 Page 1 of 3

High Resolution Mass Spectrometry Report

Measured m/z vs. theoretical m/z

Meas. m/z	#	Formula	Score	m/z	err [mDa]	err [ppm]	mSigma	rdb	e⁻ Conf	z
1127.5324	1	C 76 H 76 N 2 Na O 2 Si 2	100.00	1127.5338	1.4	1.2	9.8	41.5	even	1+

Mass list

#	m/z	I %	I
1	236.0738	13.4	8195
2	353.2663	33.1	20190
3	354.2697	7.8	4754
4	367.2452	10.3	6301
5	381.2974	41.6	25424
6	382.3012	8.8	5390
7	395.2766	12.3	7504
8	408.3086	11.2	6868
9	413.2660	20.0	12241
10	447.3444	9.5	5782
11	467.1021	18.3	11171
12	468.1026	8.6	5277
13	489.3300	11.9	7273
14	493.3498	50.6	30899
15	494.3533	15.6	9496
16	516.4257	8.6	5258
17	521.3815	89.1	54422
18	522.3845	28.8	17573
19	536.1653	55.0	33569
20	537.1662	27.6	16826
21	538.1639	19.6	11980
22	541.1208	86.7	52920
23	542.1216	44.1	26958
24	543.1194	29.8	18196
25	544.1192	11.6	7108
26	549.4123	50.4	30784
27	550.4161	18.2	11143
28	577.4436	11.0	6740
29	610.1838	37.2	22727
30	611.1846	21.4	13097
31	612.1826	15.6	9542
32	615.1393	52.9	32299
33	616.1399	30.6	18709
34	617.1382	22.2	13582
35	618.1377	8.8	5360
36	659.2868	53.3	32564
37	660.2900	19.3	11756
38	684.2025	13.8	8430
39	685.2029	10.1	6177
40	685.4345	10.3	6271
41	686.2004	7.7	4691
42	689.1579	26.2	15982
43	690.1584	17.2	10516
44	691.1567	13.8	8441
45	705.5818	50.5	30857
46	706.5851	24.1	14704
47	721.5751	9.7	5935
48	763.1765	13.2	8041
49	764.1771	10.8	6566
50	765.1749	7.9	4807
51	837.1951	10.6	6447
52	838.1962	8.1	4925
53	839.1931	7.9	4826
54	911.2133	13.4	8213
55	912.2141	11.1	6798
56	913.2117	10.0	6101
57	948.4194	8.7	5287
58	985.2323	16.9	10315
59	986.2332	15.5	9486
60	987.2319	14.4	8789
61	988.2313	9.0	5523
62	1054.2958	8.2	4991

High Resolution Mass Spectrometry Report

#	m/z	I %	I
63	1059.2512	19.6	11994
64	1060.2521	20.2	12348
65	1061.2505	18.8	11503
66	1062.2508	13.1	8000
67	1127.5324	100.0	61068
68	1128.3142	8.2	5035
69	1128.5361	92.5	56505
70	1129.3138	8.8	5395
71	1129.5381	50.6	30896
72	1130.3143	8.8	5391
73	1130.5389	19.6	11948
74	1133.2705	19.3	11782
75	1134.2715	21.5	13126
76	1135.2702	21.3	13038
77	1136.2686	14.7	8984
78	1137.2676	8.7	5323
79	1145.5416	12.1	7416
80	1146.5458	11.1	6803
81	1190.6382	16.8	10243
82	1191.6417	16.0	9769
83	1192.6523	26.9	16438
84	1193.6561	22.6	13796
85	1194.6589	11.8	7208
86	1207.2892	16.4	10003
87	1208.2902	19.0	11630
88	1209.2891	18.9	11543
89	1210.2872	14.0	8537
90	1211.2866	8.7	5304
91	1281.3071	11.7	7152
92	1282.3083	14.0	8577
93	1283.3069	16.0	9760
94	1284.3068	11.7	7155
95	1285.3062	7.7	4703
96	1355.3273	8.1	4955
97	1356.3271	10.6	6445
98	1357.3246	12.7	7785
99	1358.3253	9.0	5520
100	1431.3445	8.6	5226

Acquisition Parameter

**General**	Fore Vacuum	2.68e+000 mBar	High Vacuum	1.22e-007 mBar	Source Type	ESI
	Scan Begin	75 m/z	Scan End	1700 m/z	Ion Polarity	Positive
**Source**	Set Nebulizer	0.4 Bar	Set Capillary	3600 V	Set Dry Gas	4.0 l/min
	Set Dry Heater	180 °C	Set End Plate Offset	-500 V		
**Quadrupole**	Set Ion Energy (MS only)	4.0 eV				
**Coll. Cell**	Collision Energy	8.0 eV	Set Collision Cell RF	500.0 Vpp		
**Ion Cooler**	Set Ion Cooler Transfer Time	80.0 µs	Set Ion Cooler Pre Pulse Storage Time	18.0 µs		

5.33 Compound **38**

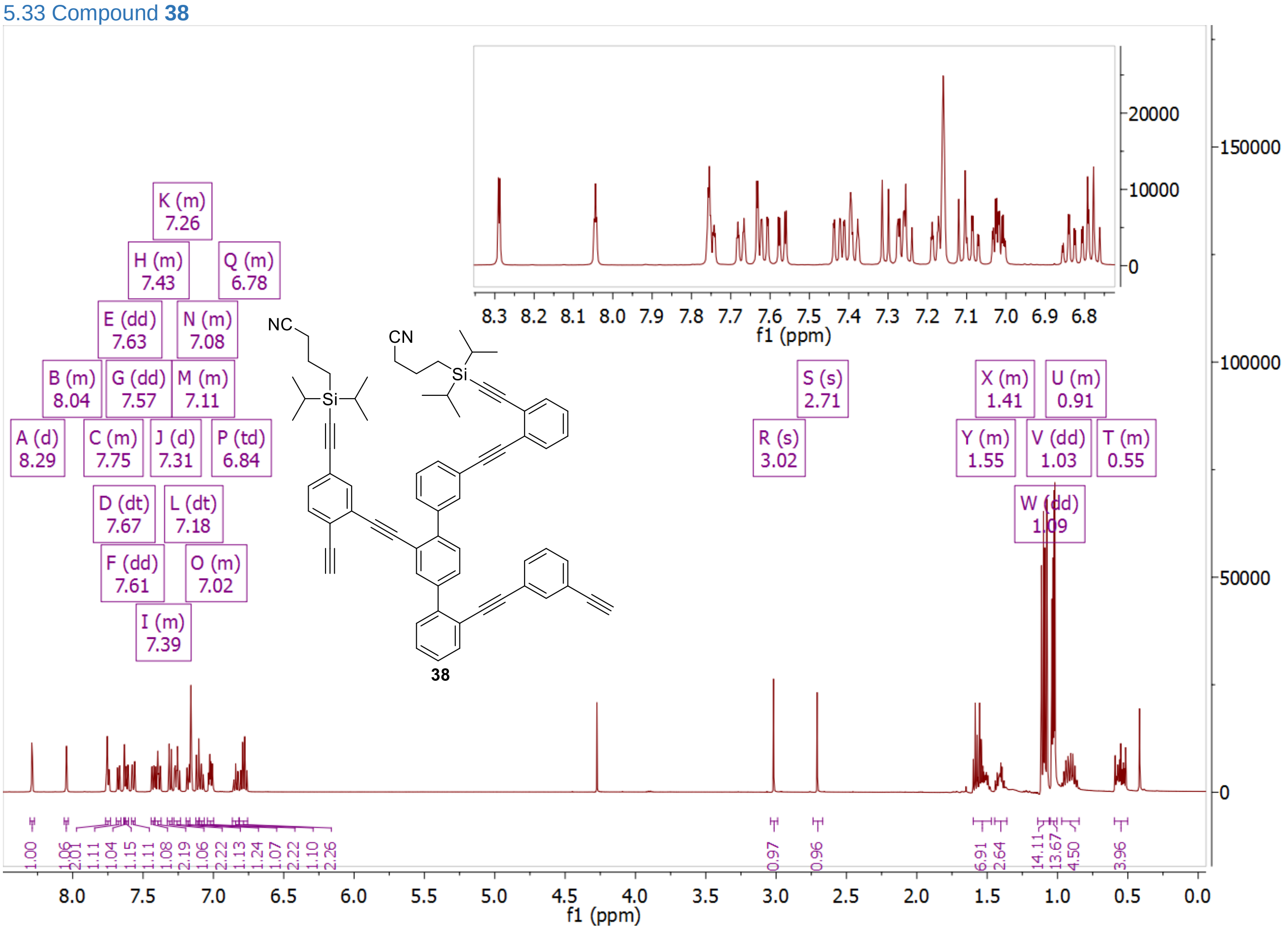

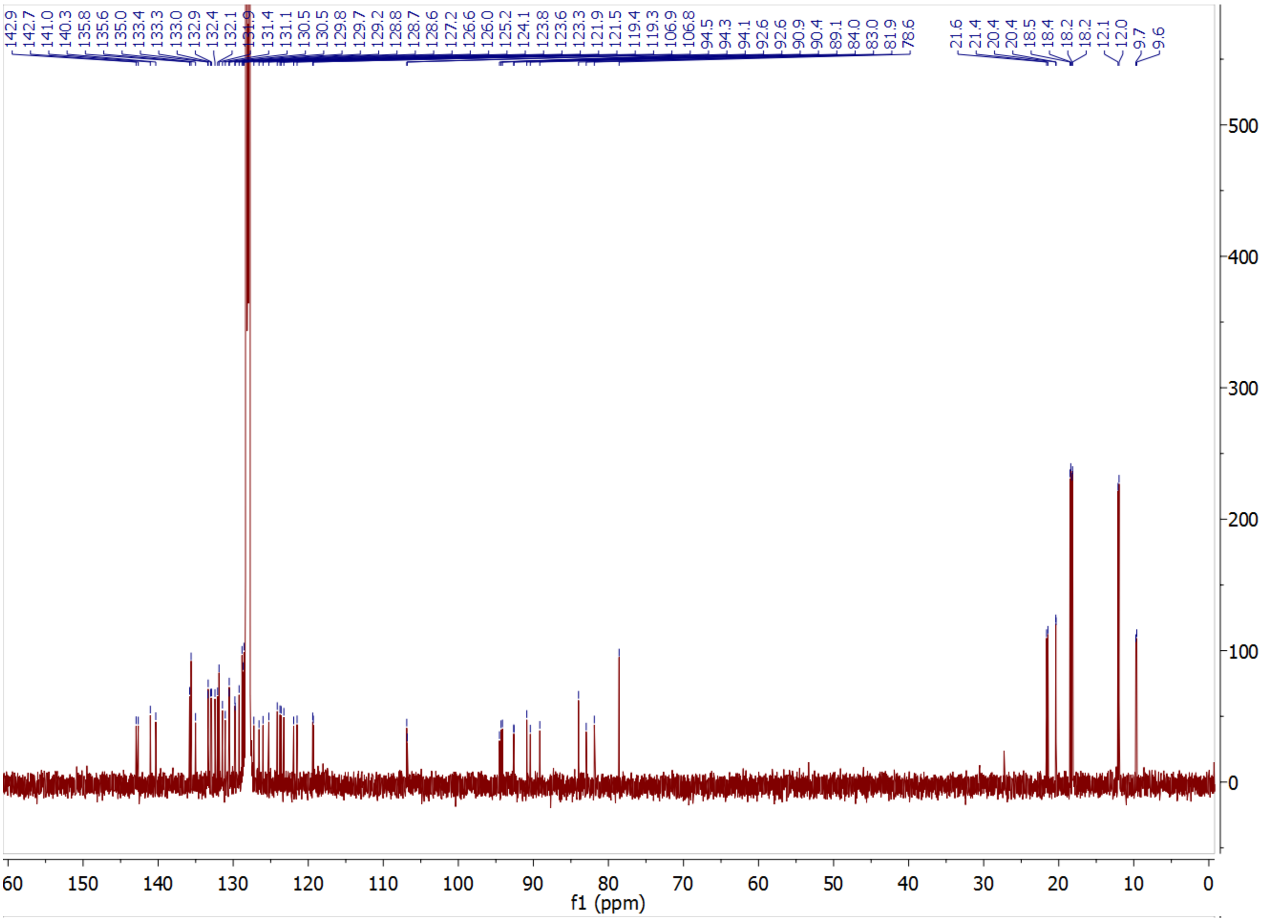

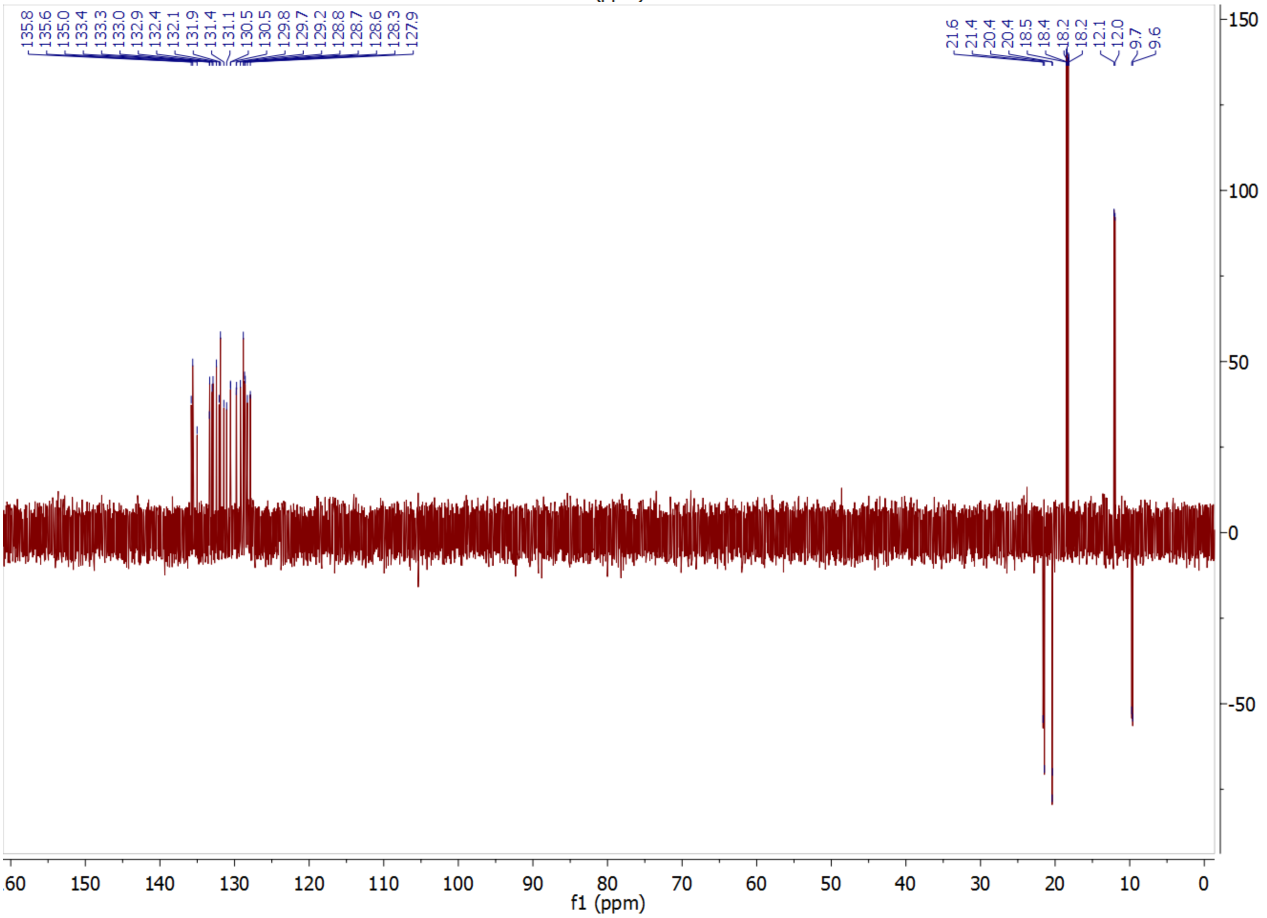

DEPT-135: ${}^{1}J_{CH}$ coupling= 145 Hz

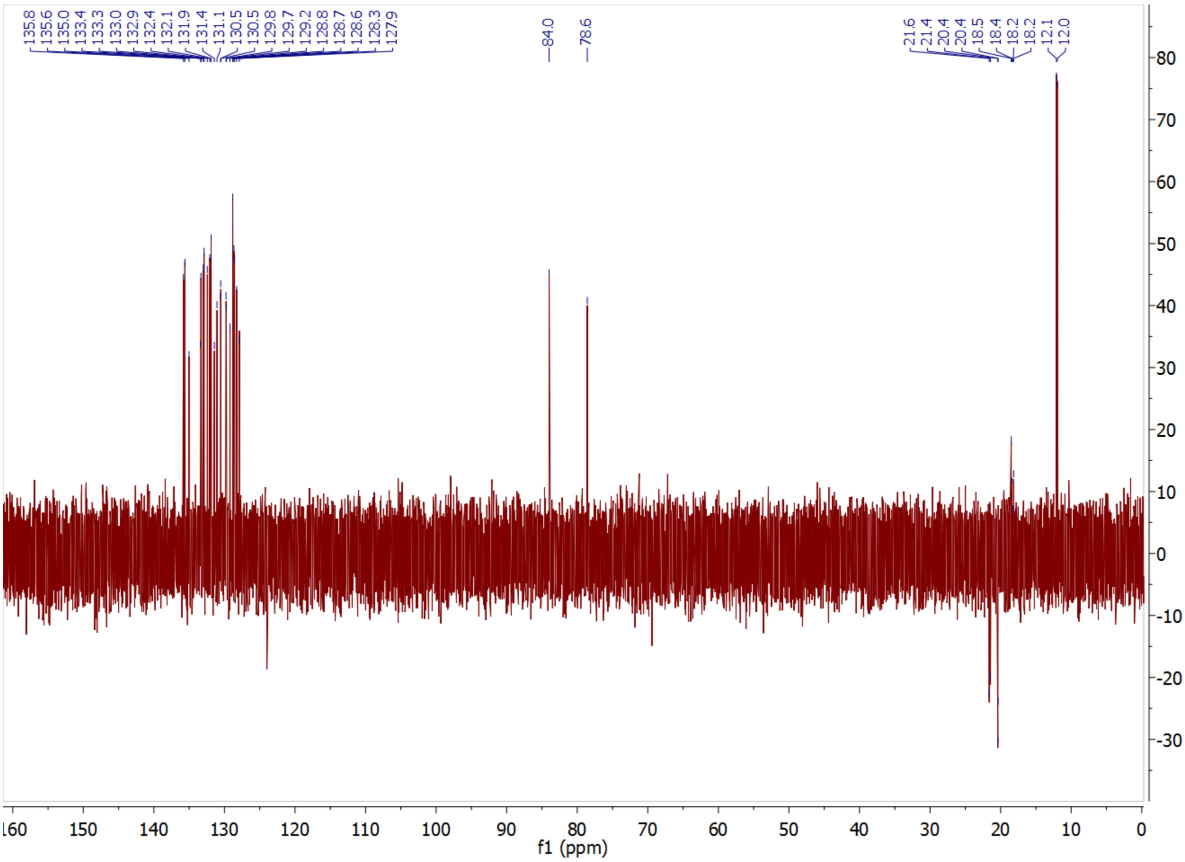

DEPT-135: ${}^{1}J_{CH}$ coupling= 200 Hz

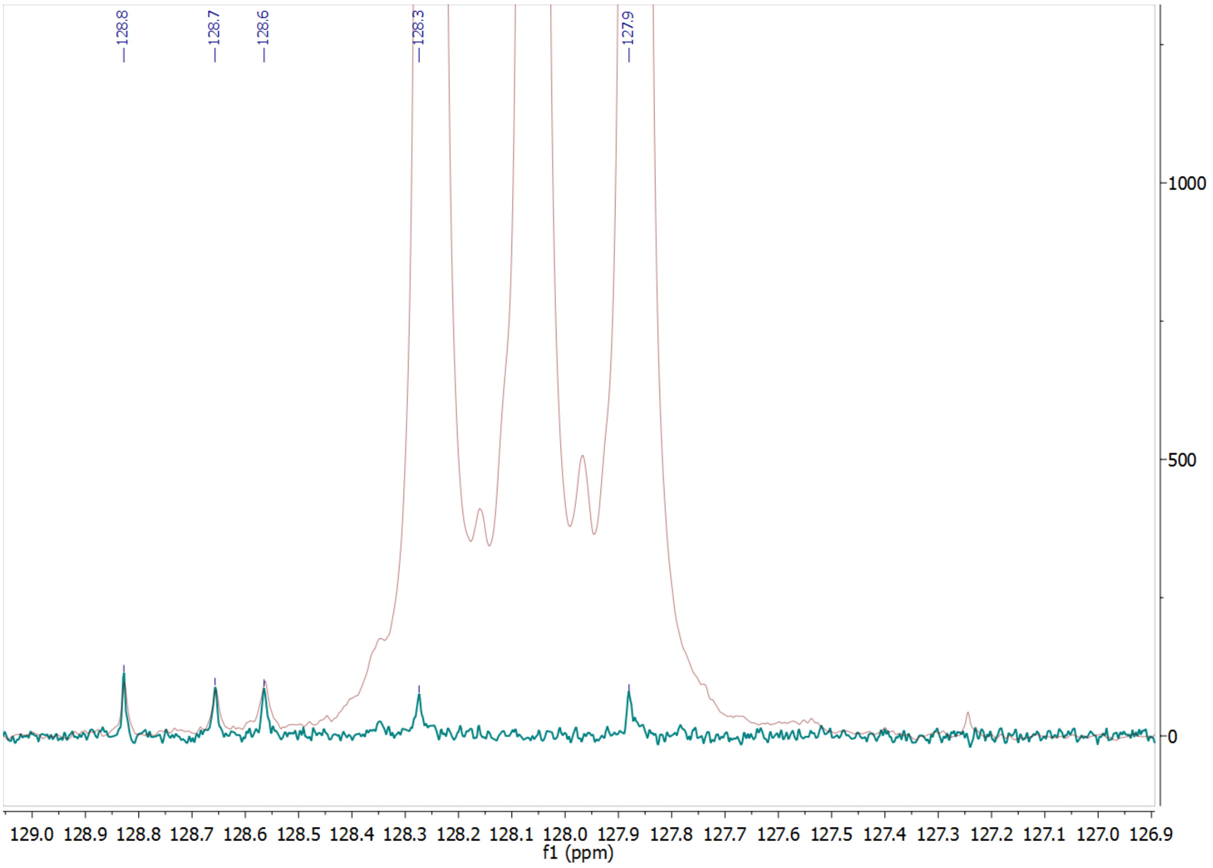

^{13}C-NMR (in red) is superimposed by DEPT-135 (in turquois).The peaks of 127.9 and 128.3 are only visible in the DEPT-135 experiment, as the signals are overlain by C_6D_6.

High Resolution Mass Spectrometry Report

Sample Name **Ba615 chr1#1 / Linda Bannwart**
Comment

Instrument maXis 4G
Method 23 Direct_pos_higher.m

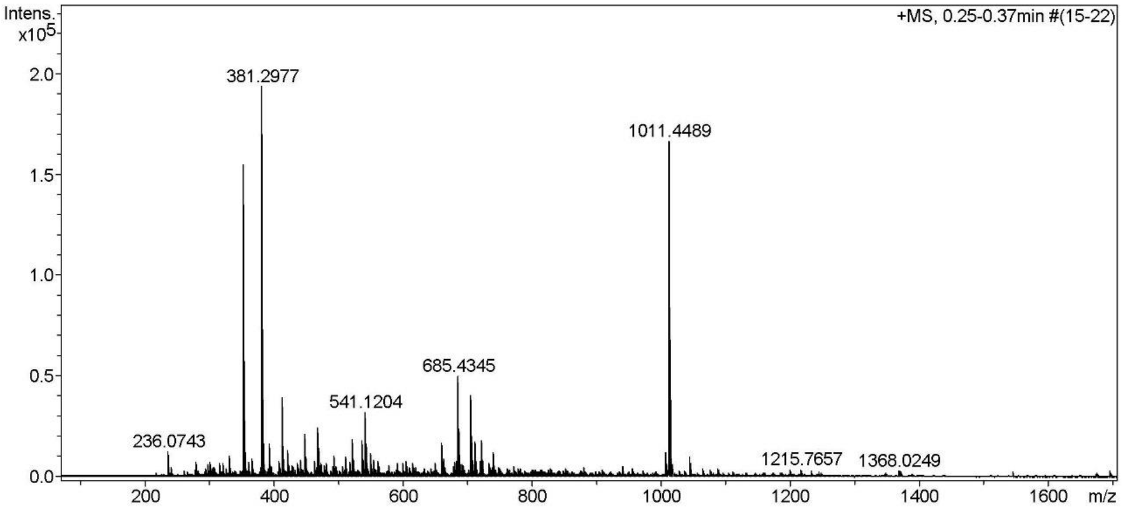

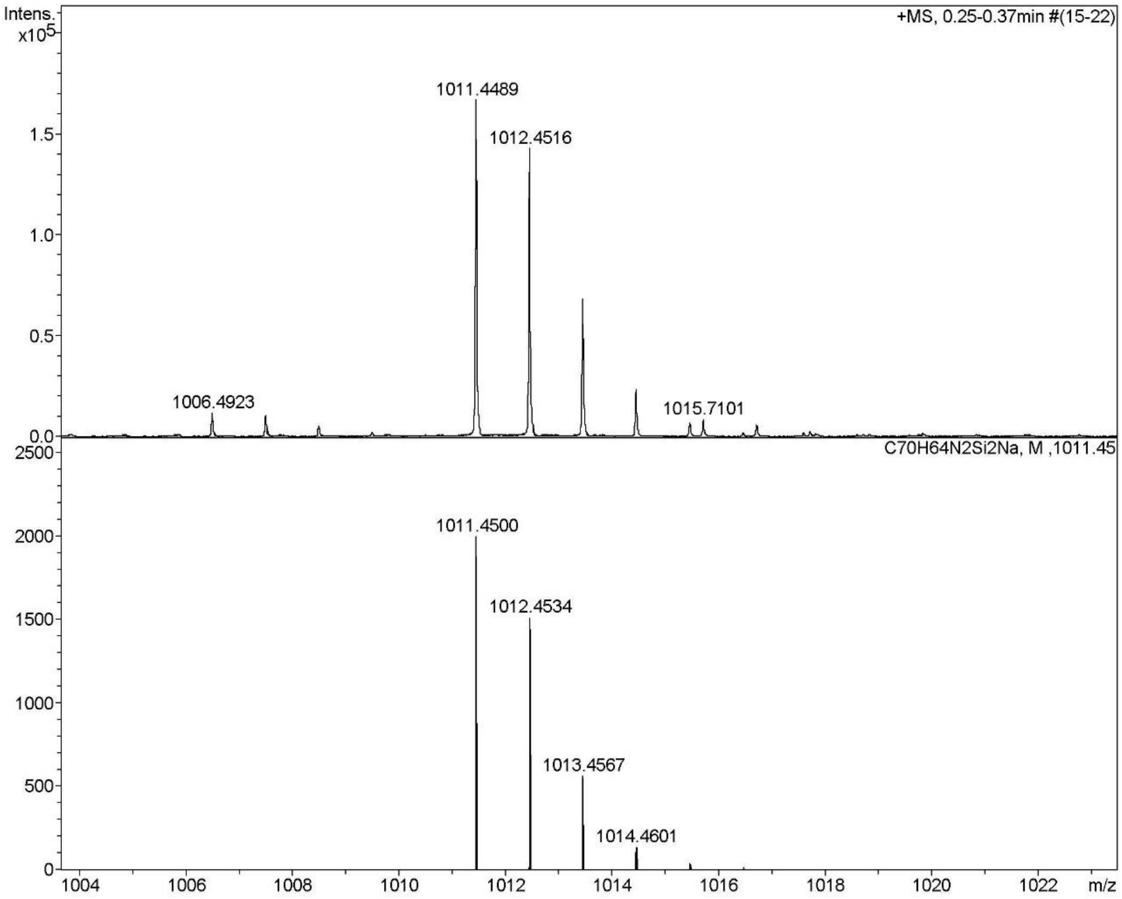

High Resolution Mass Spectrometry Report

Measured m/z vs. theoretical m/z

Meas. m/z	#	Formula	Score	m/z	err [mDa]	err [ppm]	mSigma	rdb	e^- Conf	z
1011.4489	1	C 70 H 64 N 2 Na Si 2	100.00	1011.4500	1.1	1.1	16.0	41.5	even	1+

Mass list

#	m/z	I %	I
1	236.0743	6.4	12447
2	279.2297	3.7	7249
3	297.0307	3.2	6118
4	301.1409	3.7	7208
5	315.1933	3.4	6658
6	321.2762	3.4	6547
7	331.1879	5.4	10548
8	353.2665	79.8	154768
9	354.2696	16.5	32005
10	360.3233	3.6	7071
11	365.1054	4.2	8091
12	365.2668	4.5	8696
13	381.2977	100.0	193933
14	382.3008	22.5	43623
15	383.3039	3.6	7075
16	384.1934	3.7	7086
17	393.2973	8.4	16218
18	395.2766	2.7	5292
19	407.3134	3.2	6183
20	408.3092	4.1	7972
21	413.2660	20.3	39334
22	414.2690	5.3	10224
23	421.3287	6.8	13151
24	423.2199	3.3	6426
25	427.2085	3.0	5860
26	429.3186	2.8	5340
27	435.3440	3.4	6498
28	441.2969	4.2	8104
29	447.3442	11.0	21361
30	448.3474	3.3	6409
31	449.3604	5.1	9814
32	463.3752	4.0	7823
33	467.1015	12.6	24386
34	467.2460	4.8	9400
35	468.1021	5.5	10631
36	469.0998	3.7	7250
37	469.3282	7.4	14368
38	470.3318	2.9	5617
39	473.3444	3.3	6489
40	477.3909	2.8	5509
41	481.3127	3.5	6726
42	491.4067	2.6	5116
43	493.3494	5.5	10602
44	495.3286	2.7	5294
45	511.2723	5.1	9822
46	517.3707	3.8	7376
47	521.3806	9.6	18605
48	522.3836	3.2	6254
49	523.3234	4.5	8632
50	536.1647	9.4	18229
51	537.1656	4.7	9078
52	538.1632	3.4	6628
53	541.1204	16.5	31957
54	542.1209	8.3	16141
55	543.1189	5.7	11096
56	549.4116	6.1	11751
57	555.2985	4.4	8488
58	561.3966	4.2	8090
59	577.4787	2.9	5656
60	591.4944	3.6	6889
61	599.3243	3.4	6499
62	605.4228	4.1	7994

High Resolution Mass Spectrometry Report

#	m/z	I %	I
63	615.1384	3.5	6782
64	649.4504	3.6	6949
65	659.2863	8.8	16974
66	660.2894	3.3	6348
67	663.4543	4.5	8666
68	677.4903	2.7	5160
69	679.4173	2.9	5565
70	680.4789	3.7	7246
71	683.5420	3.9	7587
72	685.4345	25.8	49969
73	686.4377	12.3	23901
74	687.4403	3.5	6829
75	691.5099	3.3	6369
76	693.4744	3.1	5936
77	705.5252	3.8	7294
78	705.5813	21.0	40724
79	706.5844	9.4	18254
80	707.5844	3.4	6547
81	711.5729	8.9	17276
82	712.5763	4.0	7725
83	719.5422	3.9	7587
84	721.5753	9.4	18248
85	722.5785	4.4	8439
86	733.5556	3.4	6687
87	739.6042	6.2	11981
88	740.6076	2.8	5485
89	1006.4923	6.2	11973
90	1007.4955	5.6	10953
91	1008.4969	2.8	5479
92	1011.4489	86.1	166951
93	1012.4516	73.8	143063
94	1013.4529	35.3	68381
95	1014.4540	12.3	23818
96	1015.4554	3.6	6924
97	1015.7101	4.6	8847
98	1016.7138	3.1	5968
99	1043.7417	5.0	9707
100	1044.7441	3.6	6926

Acquisition Parameter

**General**	Fore Vacuum	2.69e+000 mBar	High Vacuum	1.25e-007 mBar	Source Type	ESI
	Scan Begin	75 m/z	Scan End	1700 m/z	Ion Polarity	Positive
**Source**	Set Nebulizer	0.4 Bar	Set Capillary	3600 V	Set Dry Gas	4.0 l/min
	Set Dry Heater	180 °C	Set End Plate Offset	-500 V		
**Quadrupole**	Set Ion Energy (MS only)	4.0 eV				
**Coll. Cell**	Collision Energy	8.0 eV	Set Collision Cell RF	500.0 Vpp		
**Ion Cooler**	Set Ion Cooler Transfer Time	80.0 µs	Set Ion Cooler Pre Pulse Storage Time	18.0 µs		

5.34 Compound **39**

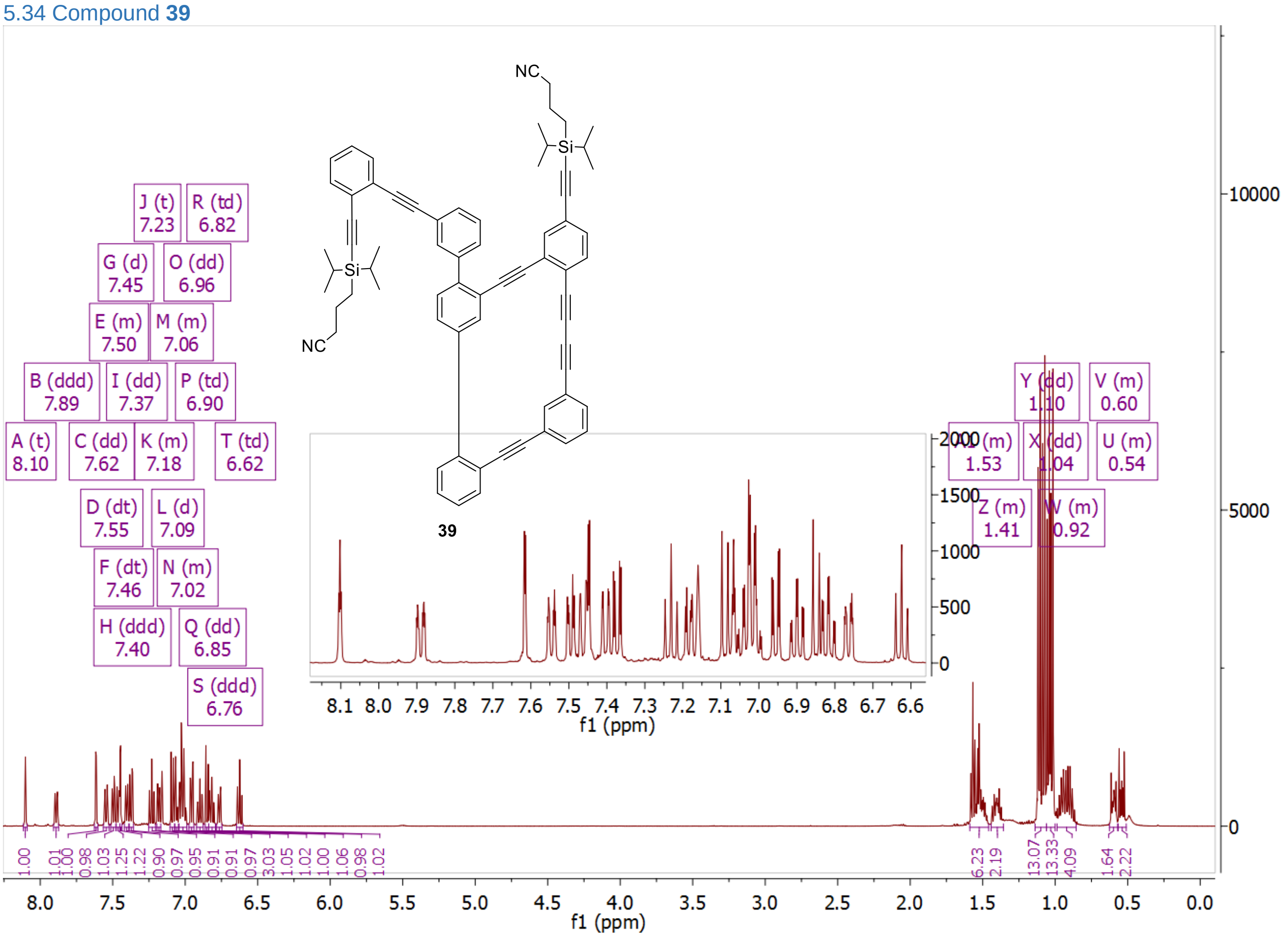

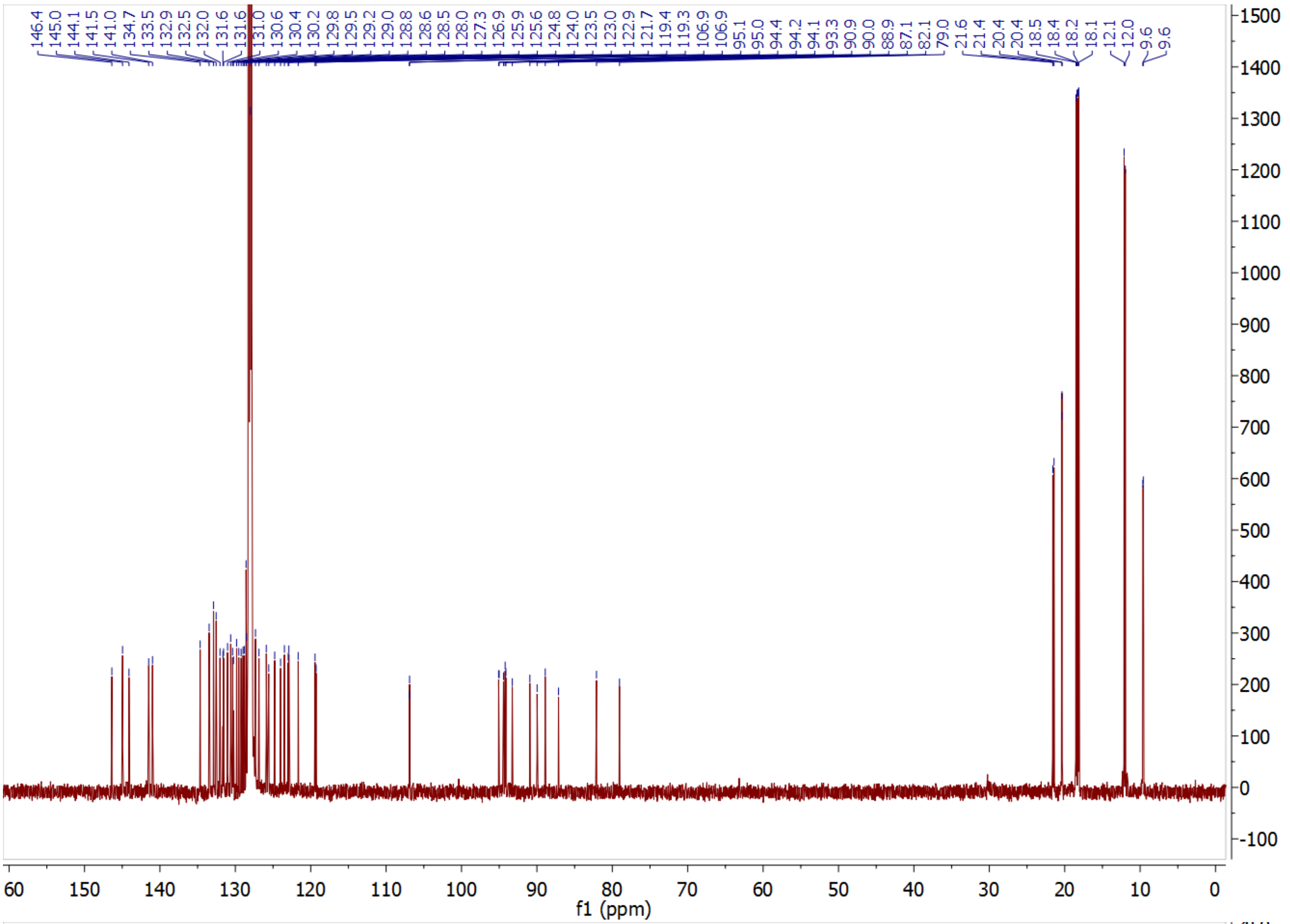
146.4
145.0
144.1
141.5
141.0
134.7
133.5
132.9
132.5
132.0
131.6
131.6
131.0
130.6
130.4
130.2
129.8
129.5
129.2
129.0
128.8
128.6
128.5
128.0
127.3
126.9
125.9
125.6
124.8
124.0
123.5
123.0
122.9
121.7
119.4
119.3
106.9
106.9
95.1
95.0
94.4
94.2
94.1
93.3
90.9
90.0
88.9
87.1
82.1
79.0
21.6
21.4
20.4
20.4
18.5
18.4
18.2
18.1
12.1
12.0
9.6
9.6
f1 (ppm)

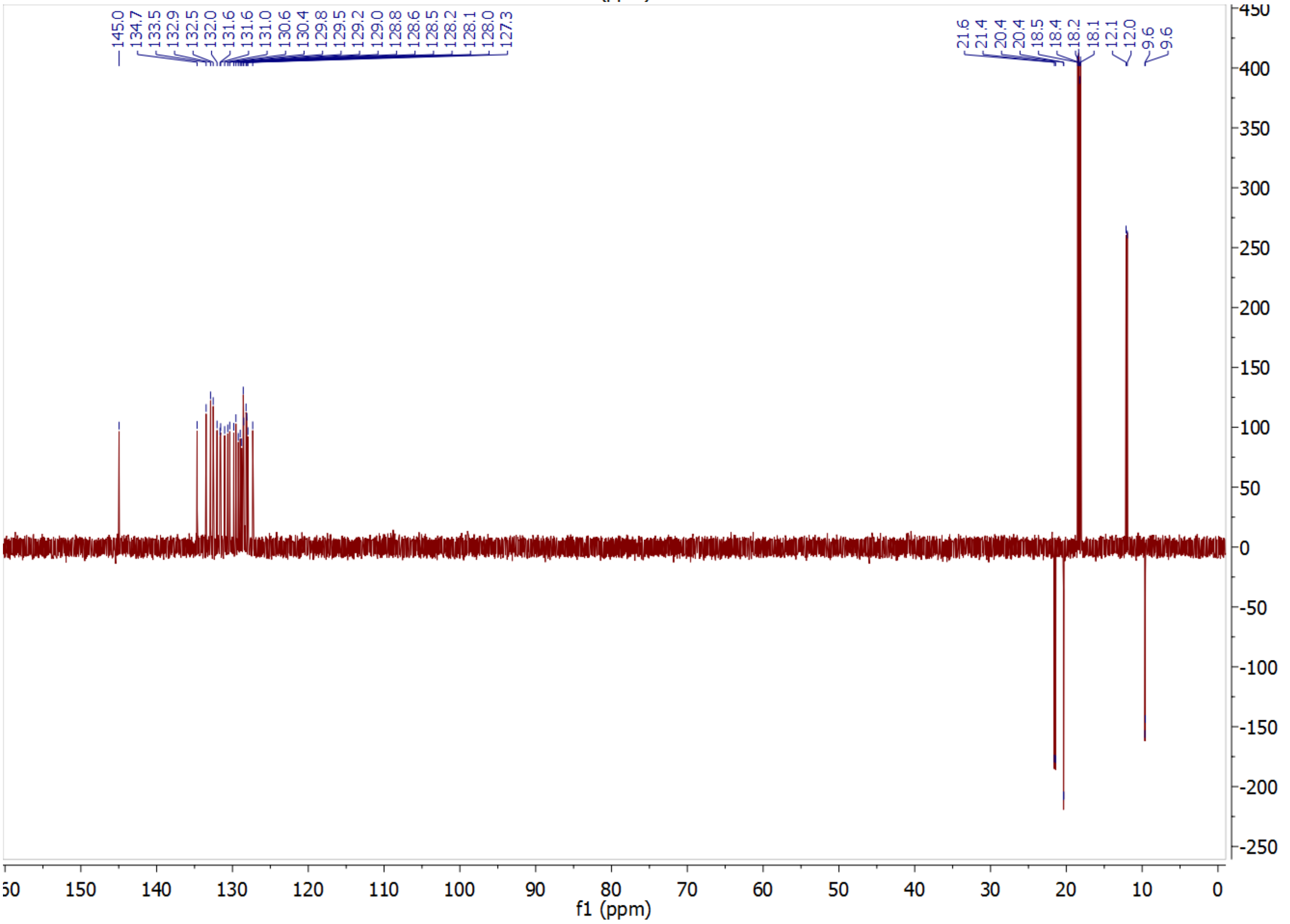
145.0
134.7
133.5
132.9
132.5
132.0
131.6
131.6
131.0
130.6
130.4
129.8
129.5
129.2
129.0
128.8
128.6
128.5
128.2
128.1
128.0
127.3
21.6
21.4
20.4
20.4
18.5
18.4
18.2
18.1
12.1
12.0
9.6
9.6
f1 (ppm)

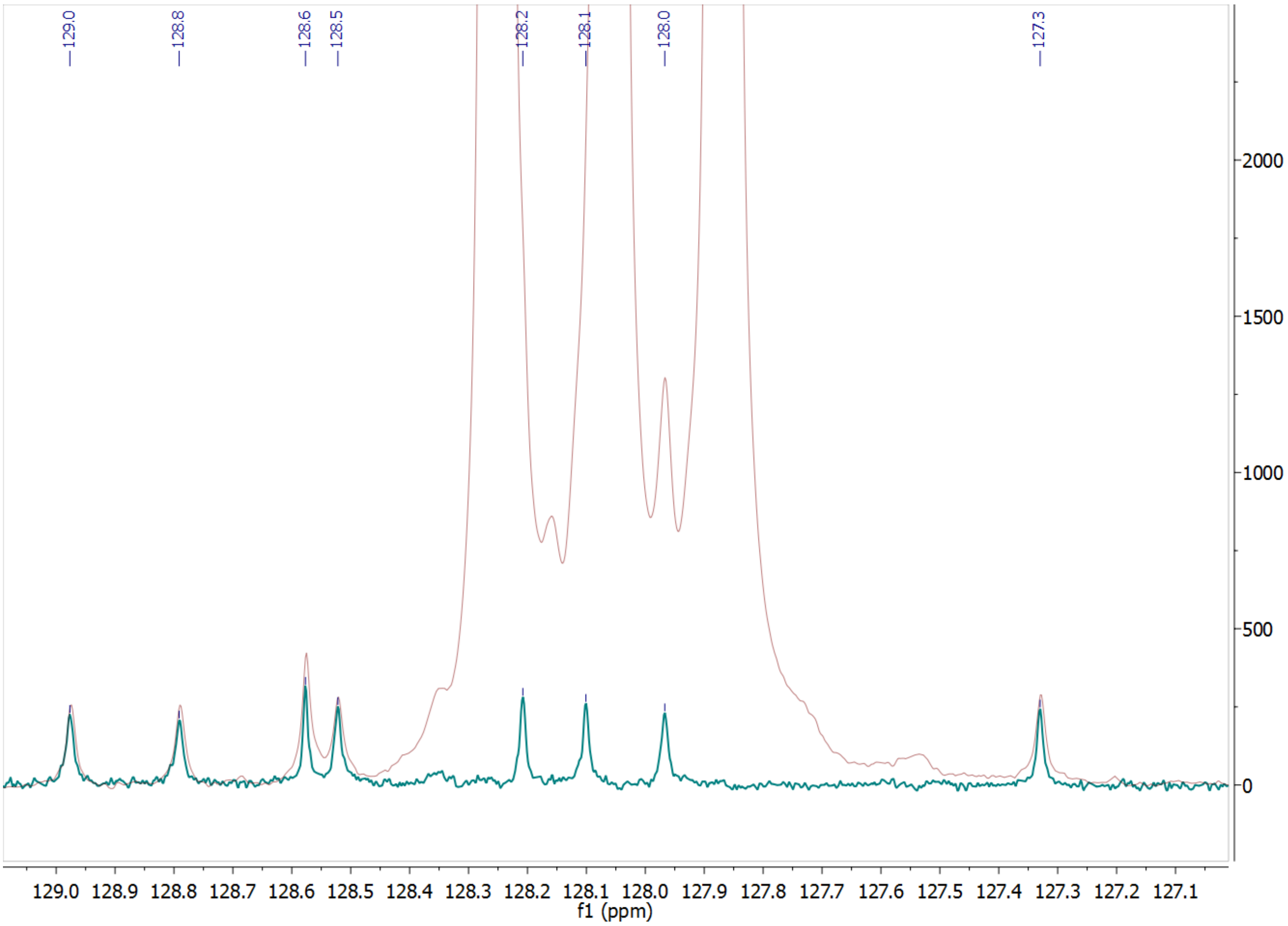

^{13}C-NMR (in red) is superimposed by DEPT-135 (in turquois).The peaks of 128.1 and 128.2 are only visible in the DEPT-135 experiment, as the signals are overlain by C_6D_6.

High Resolution Mass Spectrometry Report

Sample Name **Ba616 chr1#1 / Linda Bannwart**
Comment

Instrument maXis 4G
Method 23 Direct_pos_higher.m

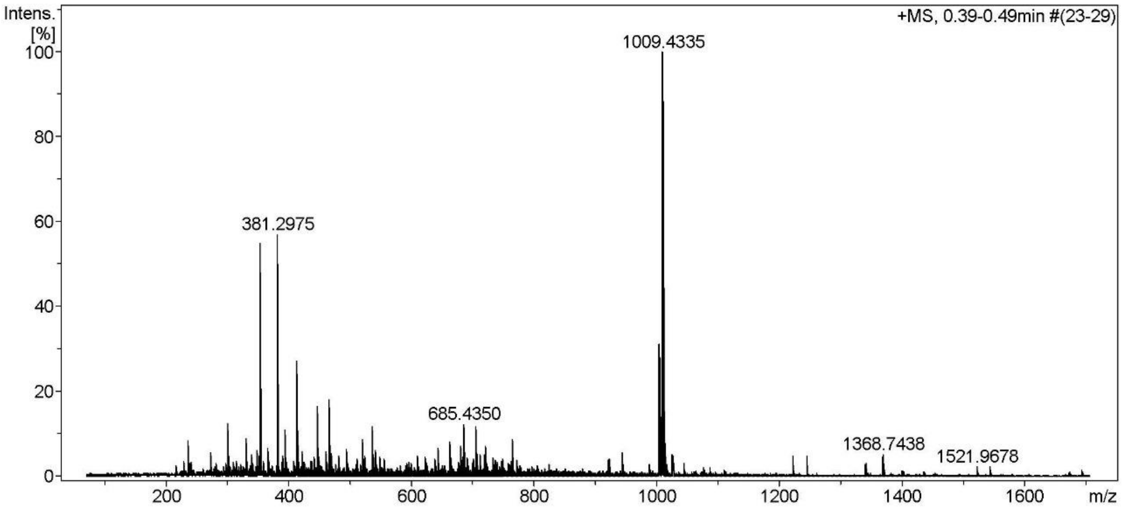

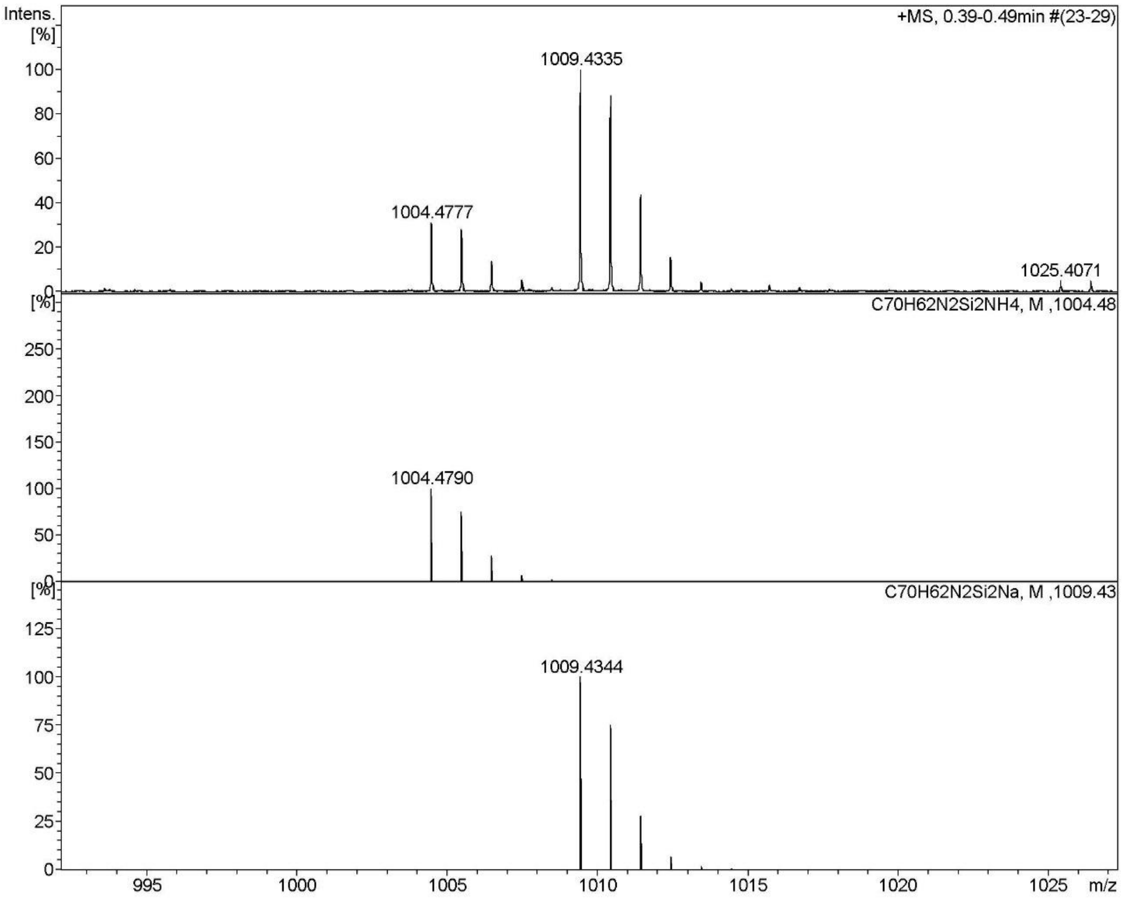

High Resolution Mass Spectrometry Report

Measured m/z vs. theoretical m/z

Meas. m/z	#	Formula	Score	m/z	err [mDa]	err [ppm]	mSigma	rdb	e⁻ Conf	z
1004.4777	1	C 70 H 66 N 3 Si 2	100.00	1004.4790	1.3	1.3	12.6	41.5	even	1+
1009.4335	1	C 70 H 62 N 2 Na Si 2	100.00	1009.4344	0.9	0.9	5.8	42.5	even	

Mass list

#	m/z	I %	I
1	228.1226	3.7	3103
2	236.0741	8.5	7213
3	273.1678	5.7	4811
4	301.1412	12.6	10713
5	310.2352	3.5	2953
6	315.1787	3.5	2984
7	331.1883	5.9	5002
8	331.2093	9.0	7660
9	339.1776	5.2	4446
10	348.9900	6.1	5183
11	350.9873	4.8	4105
12	353.2663	54.9	46555
13	354.2696	11.9	10080
14	359.2408	3.6	3079
15	365.1056	6.6	5581
16	365.2668	5.1	4294
17	367.2087	3.8	3246
18	367.2458	3.5	2939
19	381.2975	56.9	48305
20	382.3008	13.7	11645
21	391.2836	4.9	4168
22	393.2976	11.1	9404
23	395.2762	3.8	3209
24	407.3130	3.6	3022
25	413.2659	27.3	23181
26	414.2693	8.1	6860
27	421.3285	6.0	5115
28	423.2197	3.4	2903
29	435.3441	3.7	3162
30	437.2876	3.7	3150
31	441.2971	4.5	3850
32	447.3442	16.6	14055
33	448.3483	4.9	4193
34	449.3601	4.9	4176
35	460.3629	5.8	4943
36	463.3752	3.5	2956
37	465.3184	18.0	15301
38	466.3218	5.3	4458
39	467.1017	4.2	3565
40	467.2464	4.0	3379
41	469.3274	5.4	4608
42	481.3125	4.8	4112
43	493.3501	6.5	5512
44	495.3294	3.5	2932
45	511.2724	4.1	3481
46	521.3808	8.6	7307
47	523.3234	5.0	4258
48	536.1652	11.8	10039
49	537.1658	5.9	5005
50	538.1634	4.9	4120
51	541.1209	6.1	5146
52	549.4128	4.8	4036
53	555.2994	4.0	3417
54	595.3441	3.5	2929
55	609.3595	5.0	4203
56	610.1834	3.7	3147
57	622.0284	4.5	3848
58	637.3912	4.1	3483
59	644.0103	6.6	5618
60	663.4530	8.1	6898
61	664.4555	4.4	3715

High Resolution Mass Spectrometry Report

#	m/z	I %	I
62	680.4789	7.2	6118
63	681.4822	4.0	3353
64	684.2018	4.7	3973
65	685.4350	12.4	10490
66	686.4384	5.7	4801
67	691.5099	4.3	3634
68	700.6261	4.1	3501
69	705.5254	4.8	4106
70	705.5814	11.8	9978
71	706.5846	5.6	4778
72	711.5733	5.1	4364
73	719.5418	5.1	4306
74	721.5743	7.2	6094
75	733.5564	4.4	3708
76	736.5418	3.8	3189
77	739.6037	3.5	2963
78	747.5726	3.6	3034
79	748.5408	4.1	3459
80	762.5569	3.7	3098
81	764.5724	8.6	7279
82	765.5760	5.2	4371
83	771.5735	3.8	3187
84	922.0084	4.1	3494
85	943.9908	5.6	4749
86	1004.4777	31.3	26604
87	1005.4806	28.0	23792
88	1006.4823	13.7	11648
89	1007.4840	5.5	4675
90	1009.4335	100.0	84870
91	1010.4362	88.3	74903
92	1011.4378	43.7	37113
93	1012.4384	15.4	13078
94	1013.4407	4.5	3852
95	1025.4071	5.1	4350
96	1026.4099	4.8	4070
97	1221.9891	4.8	4101
98	1243.9722	4.9	4132
99	1367.7406	4.7	4030
100	1368.7438	5.2	4430

Acquisition Parameter

**General**	Fore Vacuum	2.68e+000 mBar	High Vacuum	1.26e-007 mBar	Source Type	ESI
	Scan Begin	75 m/z	Scan End	1700 m/z	Ion Polarity	Positive
**Source**	Set Nebulizer	0.4 Bar	Set Capillary	3600 V	Set Dry Gas	4.0 l/min
	Set Dry Heater	180 °C	Set End Plate Offset	-500 V		
**Quadrupole**	Set Ion Energy (MS only)	4.0 eV				
**Coll. Cell**	Collision Energy	8.0 eV	Set Collision Cell RF	500.0 Vpp		
**Ion Cooler**	Set Ion Cooler Transfer Time	80.0 µs	Set Ion Cooler Pre Pulse Storage Time	18.0 µs		

5.35 Compound **40**

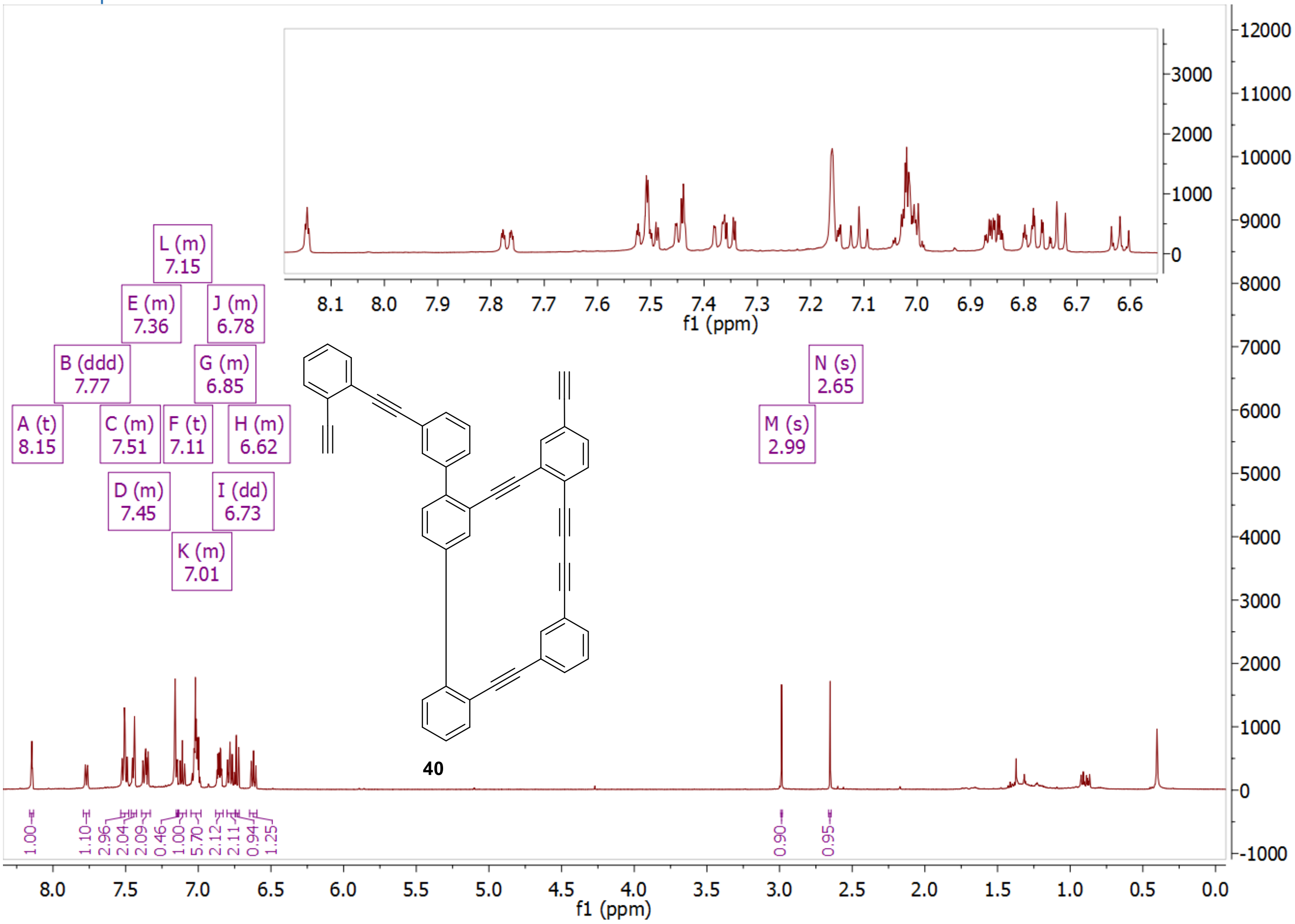

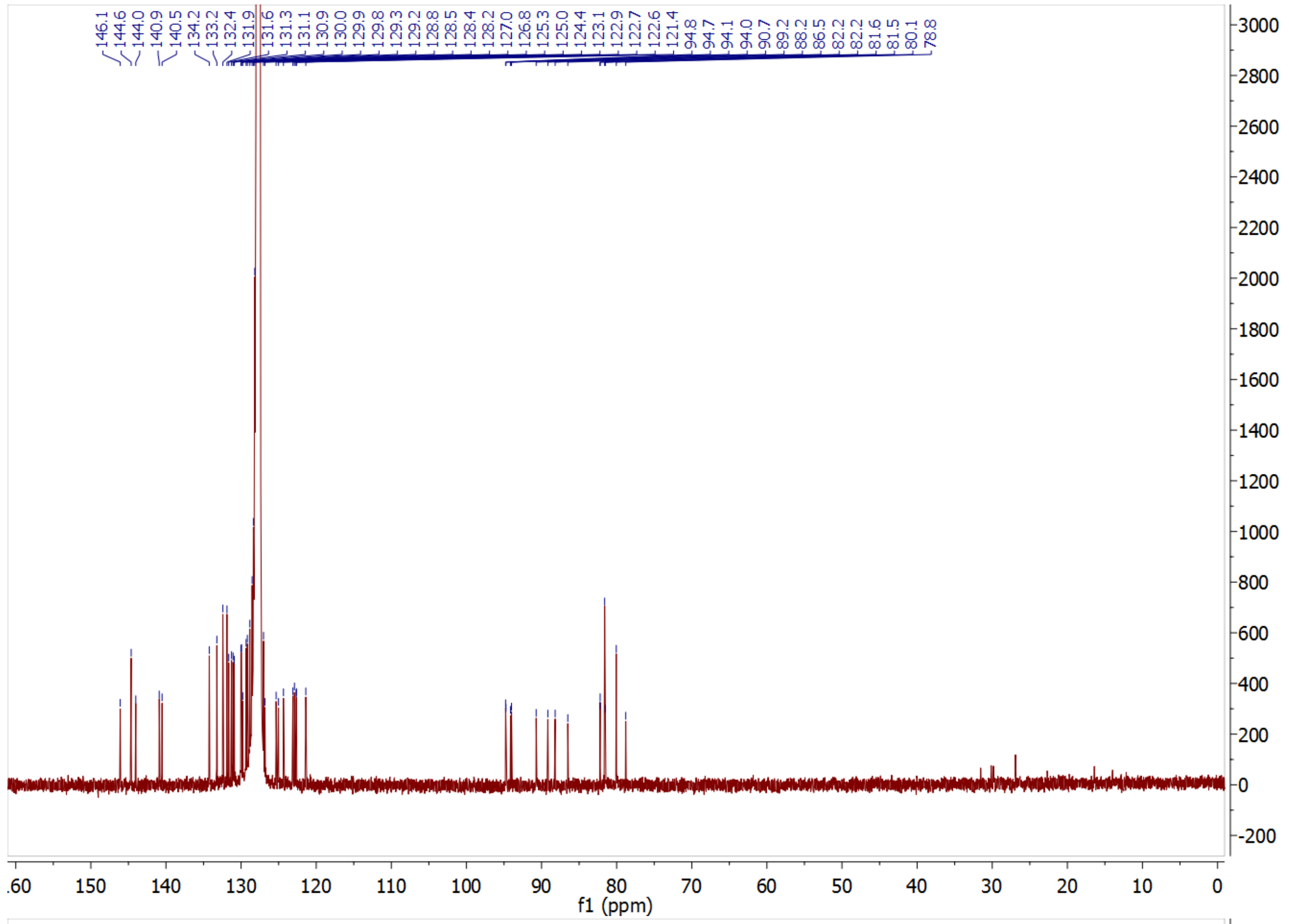

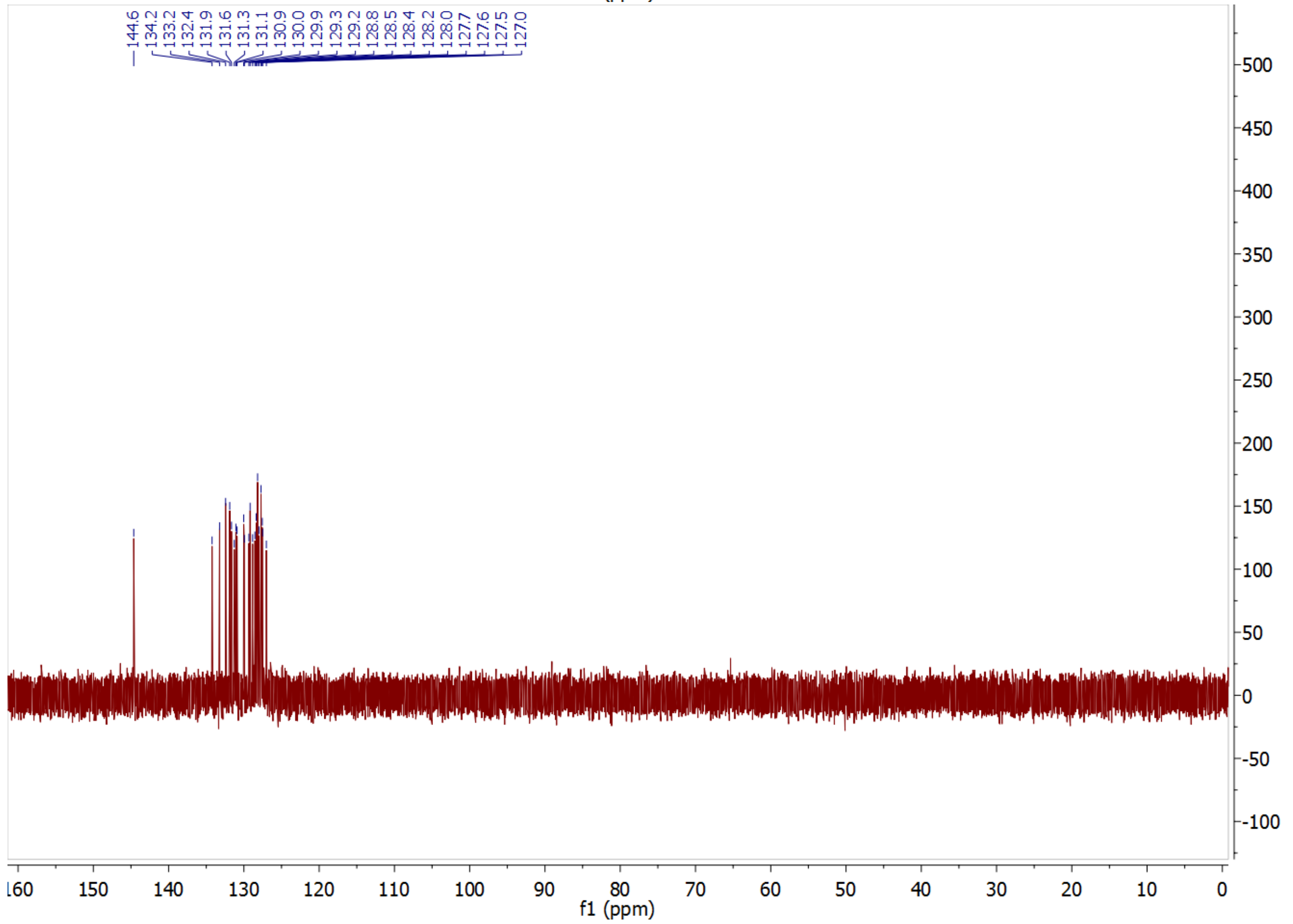

DEPT-135: $^1J_{CH}$ coupling= 145 Hz

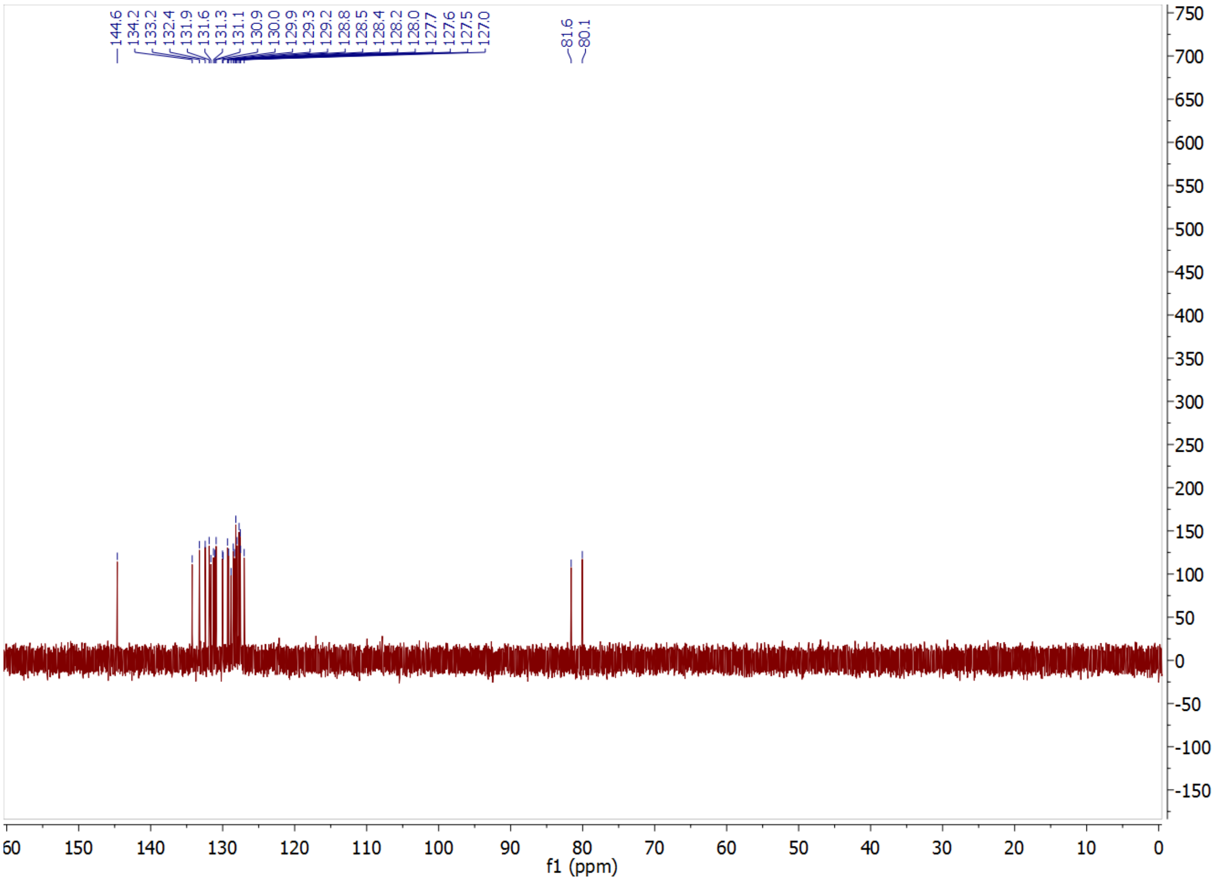

DEPT-135: $^{1}J_{CH}$ coupling= 200 Hz

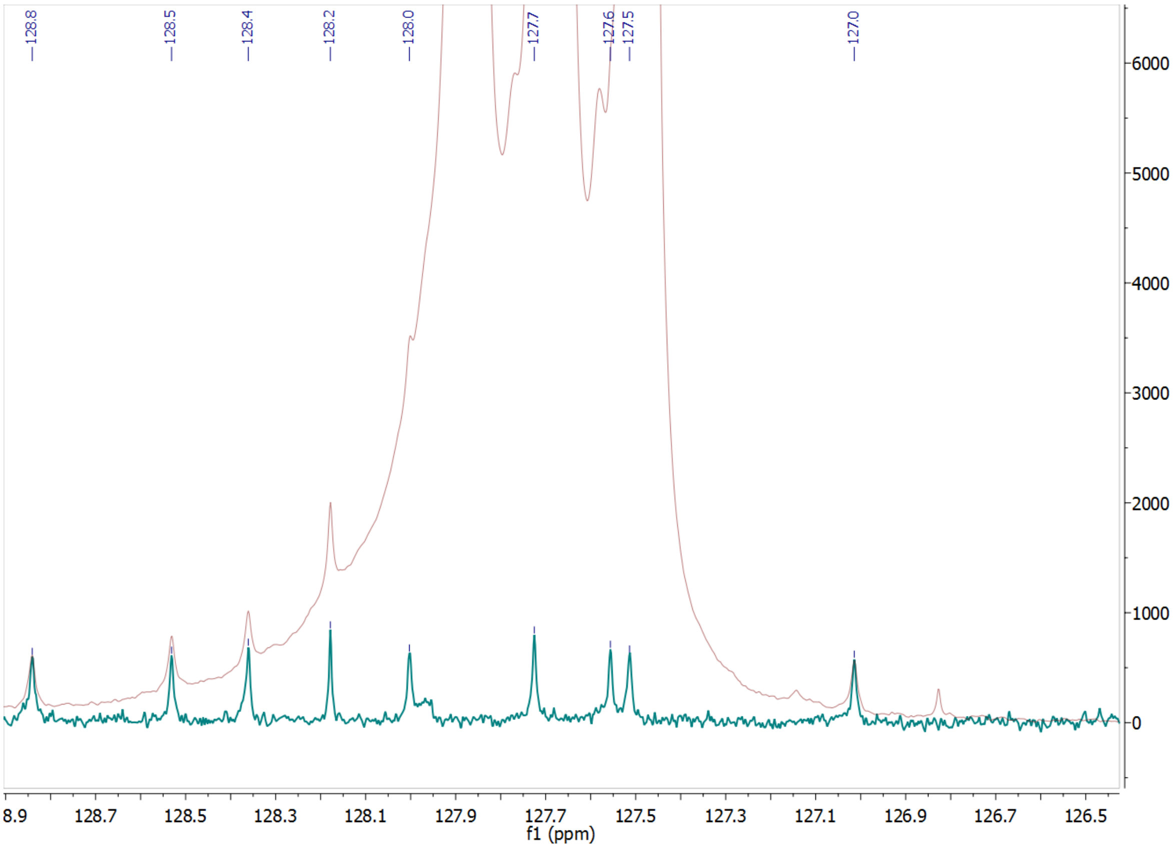

^{13}C-NMR (in red) is superimposed by DEPT-135 (in turquois).The peaks of 128.0, 127.7, 127.6, and 127.5 are only visible in the DEPT-135 experiment, as the signals are overlain by C_6D_6.

High Resolution Mass Spectrometry Report

Sample Name **Ba617 chr1#1**
Comment

Instrument maXis 4G
Method ms_nocolumn_600-1000_pos.m

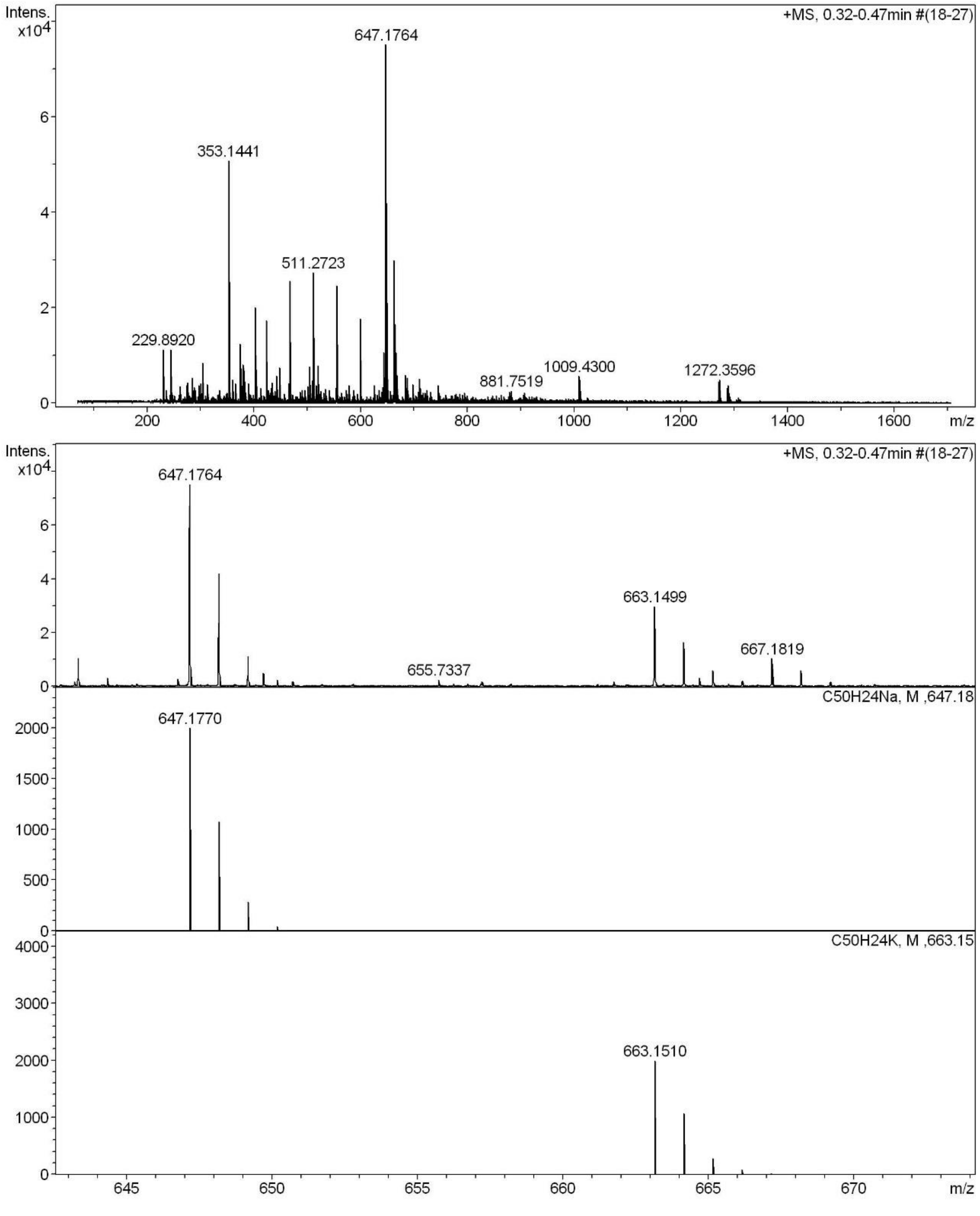

High Resolution Mass Spectrometry Report

Measured m/z vs. theoretical m/z

Meas. m/z	#	Formula	Score	m/z	err [mDa]	err [ppm]	mSigma	rdb	e⁻ Conf	z
647.1764	1	C 50 H 24 Na	100.00	647.1770	0.6	0.9	10.1	38.5	even	1+
663.1499	1	C 50 H 24 K	100.00	663.1510	1.1	1.6	10.9	38.5	even	

Mass list

#	m/z	I %	I
1	229.8920	15.1	11380
2	236.0710	3.9	2931
3	244.8664	14.9	11206
4	247.0832	3.3	2516
5	262.0586	4.2	3164
6	262.8770	4.8	3617
7	275.1596	5.0	3748
8	276.8927	5.9	4460
9	285.8930	7.2	5384
10	288.9207	4.5	3363
11	291.1545	3.7	2746
12	297.8785	5.0	3761
13	301.1386	5.6	4183
14	303.8957	11.3	8502
15	312.8544	5.4	4086
16	335.1661	3.7	2786
17	353.1441	67.7	50864
18	354.1474	20.2	15183
19	359.8496	6.5	4921
20	365.8666	5.6	4219
21	374.8254	16.5	12376
22	375.8221	9.7	7329
23	379.1927	10.7	8052
24	380.8422	10.5	7872
25	381.2967	3.5	2599
26	384.2421	6.1	4603
27	389.8009	5.5	4128
28	403.2562	26.9	20201
29	404.2584	9.5	7118
30	413.2649	4.3	3215
31	423.2194	23.1	17335
32	424.2225	4.6	3461
33	425.2148	3.3	2519
34	427.8378	3.6	2741
35	433.2911	4.2	3187
36	433.8546	6.0	4500
37	439.8711	3.6	2718
38	442.8130	7.6	5720
39	448.8301	10.0	7488
40	465.3179	5.2	3894
41	466.3636	5.6	4185
42	467.2459	34.0	25590
43	468.2489	7.5	5638
44	487.3594	3.3	2471
45	489.8082	3.7	2767
46	495.8252	3.9	2914
47	501.8422	4.7	3558
48	504.7838	10.3	7758
49	510.8010	6.5	4893
50	511.2723	36.5	27445
51	512.2755	8.1	6120
52	516.8171	5.0	3731
53	519.7592	10.6	7955
54	525.7766	3.4	2593
55	534.7349	4.1	3074
56	541.1202	3.7	2753
57	553.4583	4.3	3209
58	555.2986	32.7	24597
59	556.3016	9.0	6749
60	572.7715	3.8	2874
61	578.7884	5.1	3807

High Resolution Mass Spectrometry Report

#	m/z	I %	I
62	587.7472	3.9	2934
63	599.3242	23.7	17831
64	600.3276	6.9	5205
65	625.1941	5.2	3913
66	634.7418	3.8	2868
67	642.2201	4.4	3338
68	643.3505	14.3	10768
69	644.3530	4.5	3401
70	646.7751	3.8	2895
71	647.1764	100.0	75184
72	648.1795	55.8	41941
73	649.1825	15.1	11338
74	649.7172	6.9	5223
75	655.7337	3.5	2667
76	663.1499	39.9	29982
77	664.1531	22.2	16691
78	664.6917	4.2	3180
79	665.1538	8.0	6050
80	667.1819	14.1	10612
81	668.1854	8.1	6062
82	683.1560	7.9	5938
83	684.1594	4.3	3217
84	685.4340	3.5	2607
85	687.3762	7.3	5479
86	697.2120	5.3	4008
87	710.2825	7.0	5244
88	711.2858	4.3	3269
89	724.1214	3.7	2802
90	731.4026	3.3	2488
91	745.5045	5.2	3881
92	881.7519	3.6	2722
93	1009.4300	7.6	5707
94	1010.4322	6.5	4914
95	1011.4360	3.6	2693
96	1271.3572	6.2	4672
97	1272.3596	6.7	5072
98	1273.3637	3.8	2873
99	1287.3313	4.4	3297
100	1288.3345	5.0	3739

Acquisition Parameter

**General**	Fore Vacuum	2.60e+000 mBar	High Vacuum	1.27e-007 mBar	Source Type	ESI
	Scan Begin	75 m/z	Scan End	1700 m/z	Ion Polarity	Positive
**Source**	Set Nebulizer	2.0 Bar	Set Capillary	4500 V	Set Dry Gas	8.0 l/min
	Set Dry Heater	200 °C	Set End Plate Offset	-500 V		
**Quadrupole**	Set Ion Energy (MS only)	4.0 eV				
**Coll. Cell**	Collision Energy	8.0 eV	Set Collision Cell RF	500.0 Vpp		
**Ion Cooler**	Set Ion Cooler Transfer Time	80.0 µs	Set Ion Cooler Pre Pulse Storage Time	18.0 µs		

5.36 Compound **1**

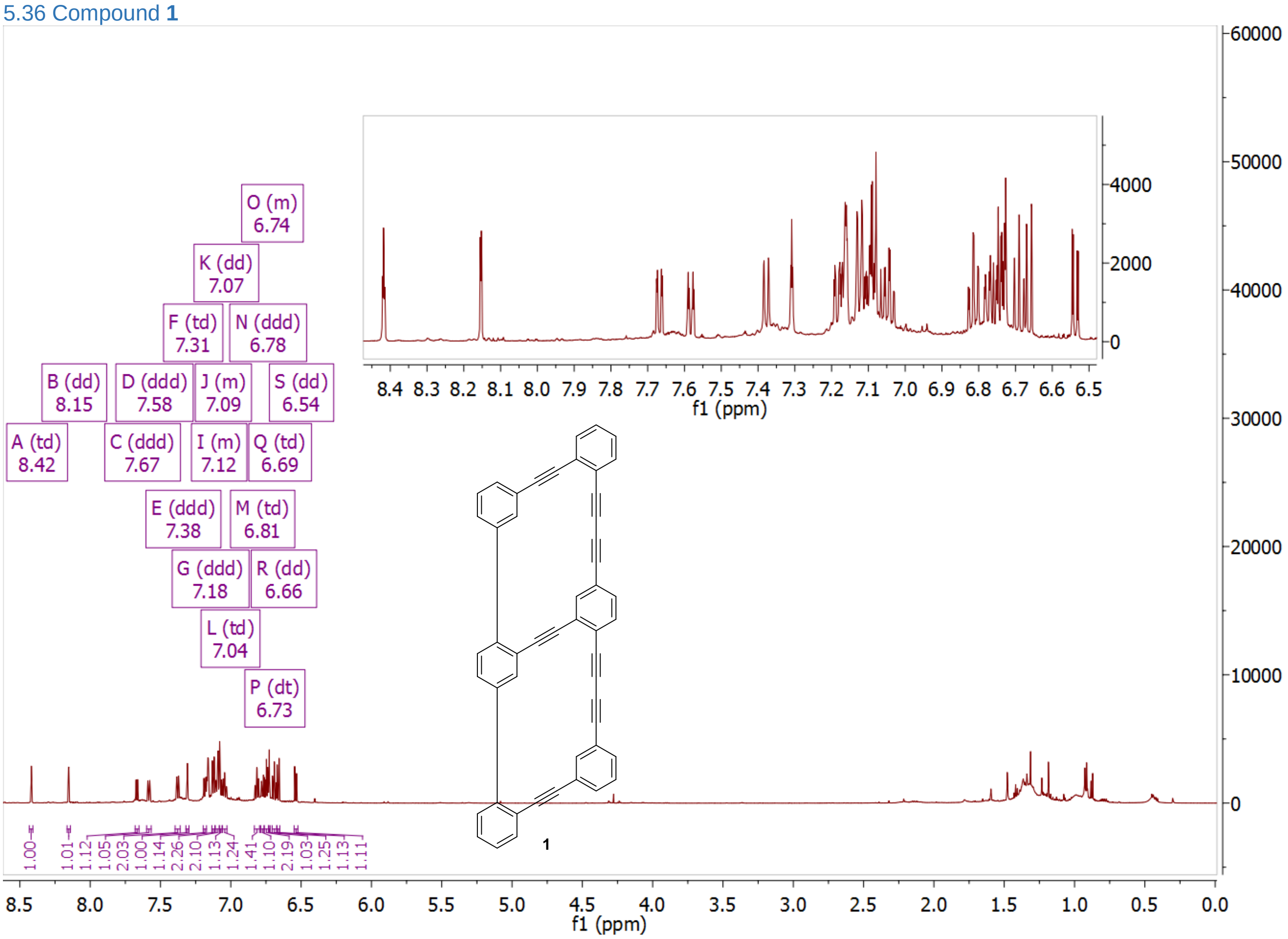

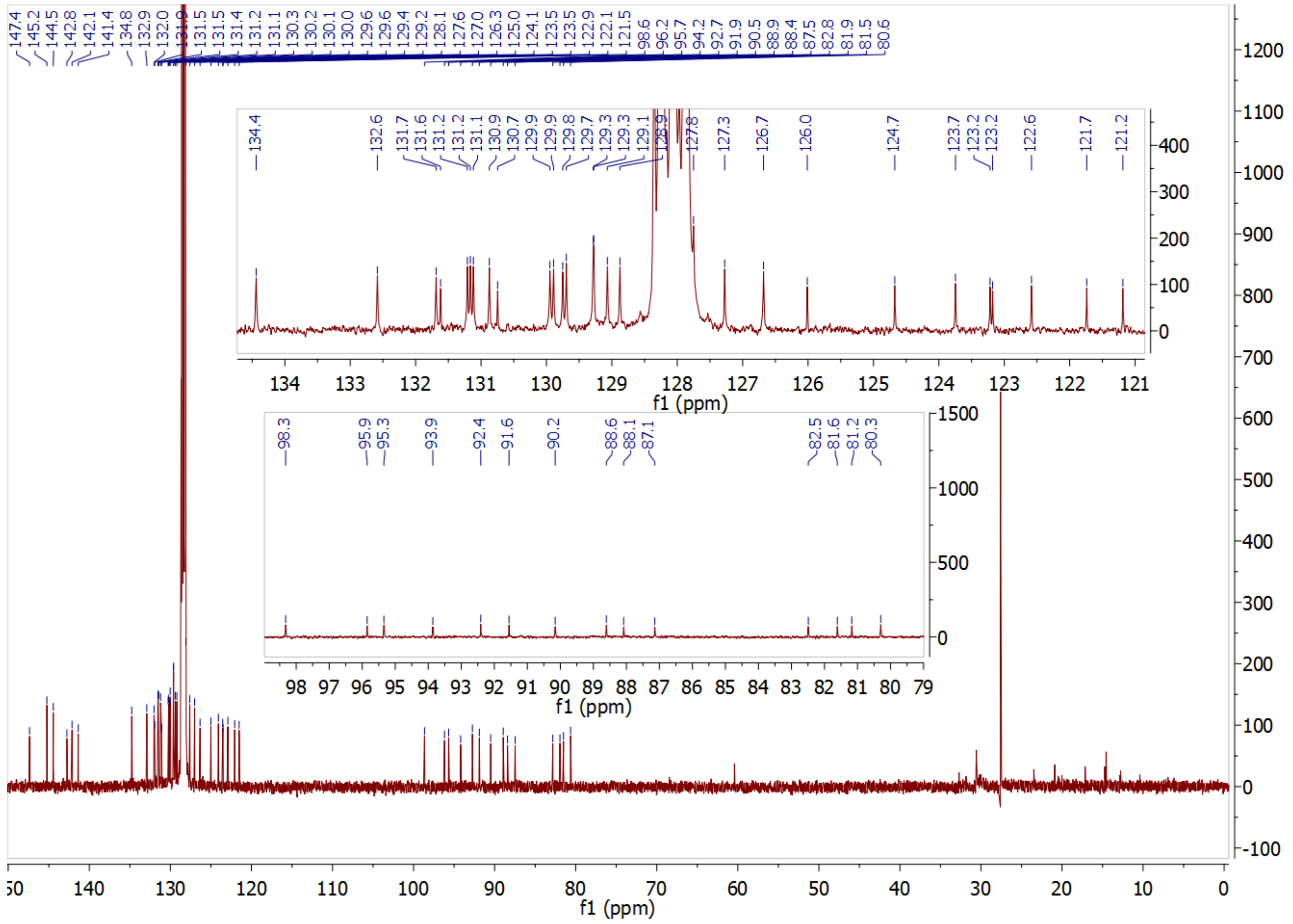

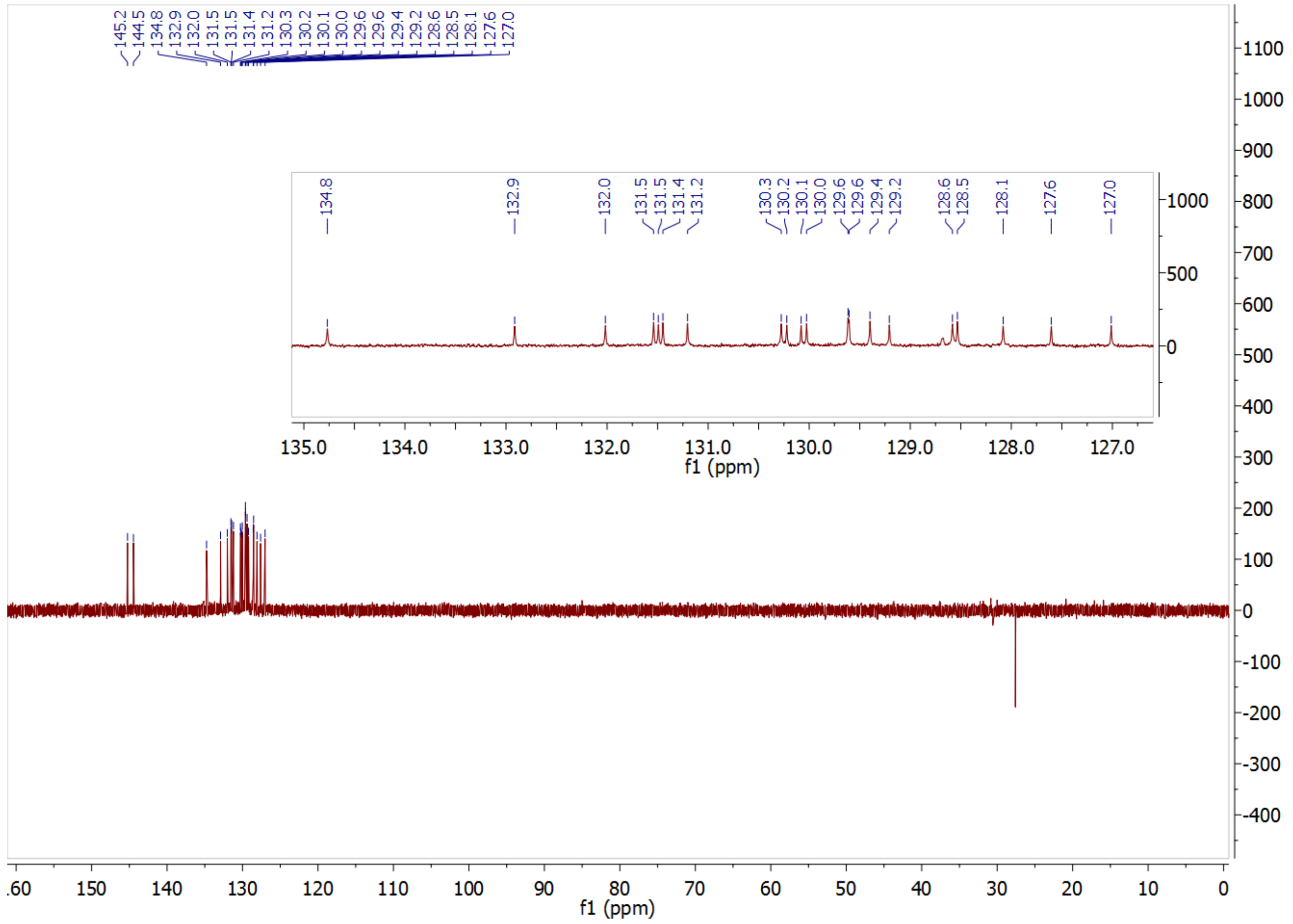

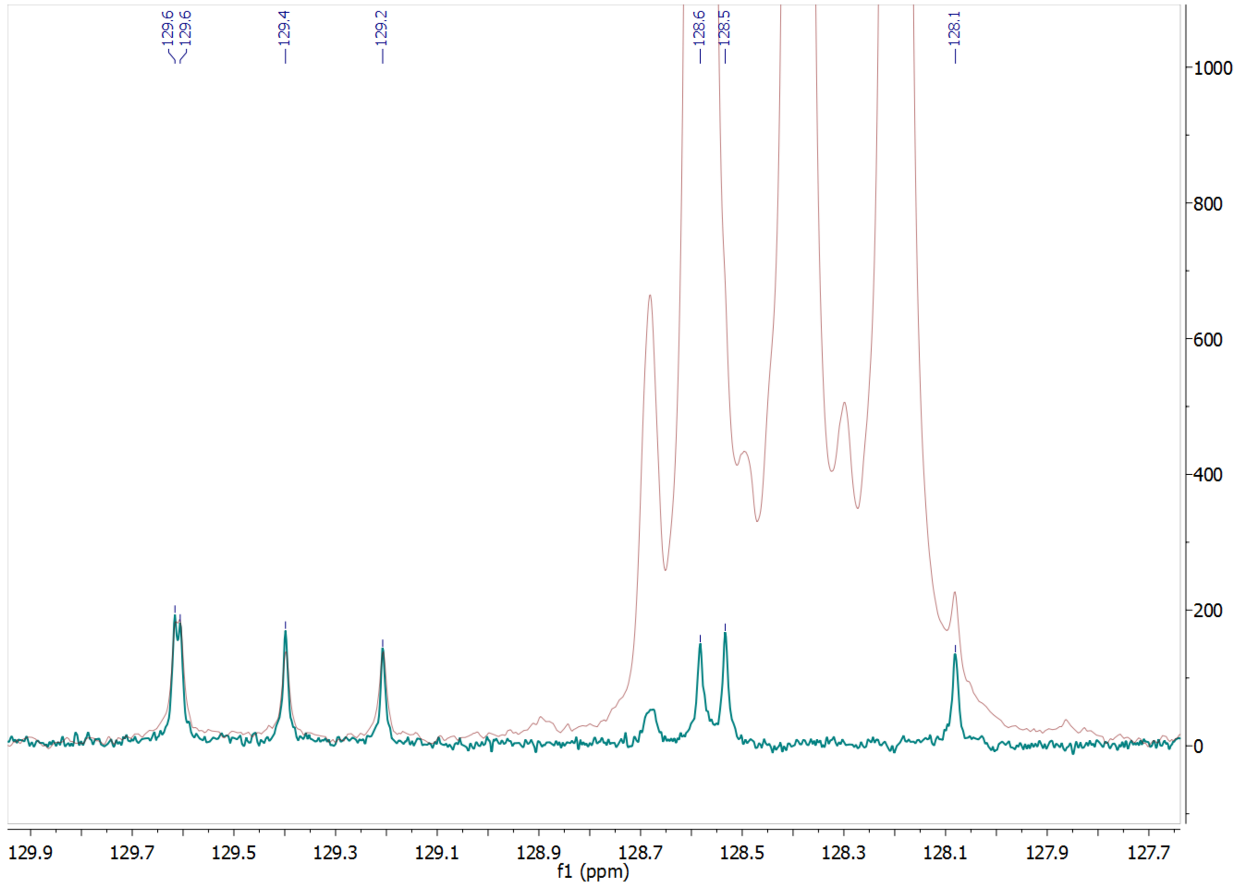

^{13}C-NMR (in red) is superimposed by DEPT-135 (in turquois).The peaks of 128.6 and 128.5 are only visible in the DEPT-135 experiment, as the signals are overlain by C_6D_6.

HMBC in C_6D_6, 600 MHz, 298 K, d6 = 100 ms (below)

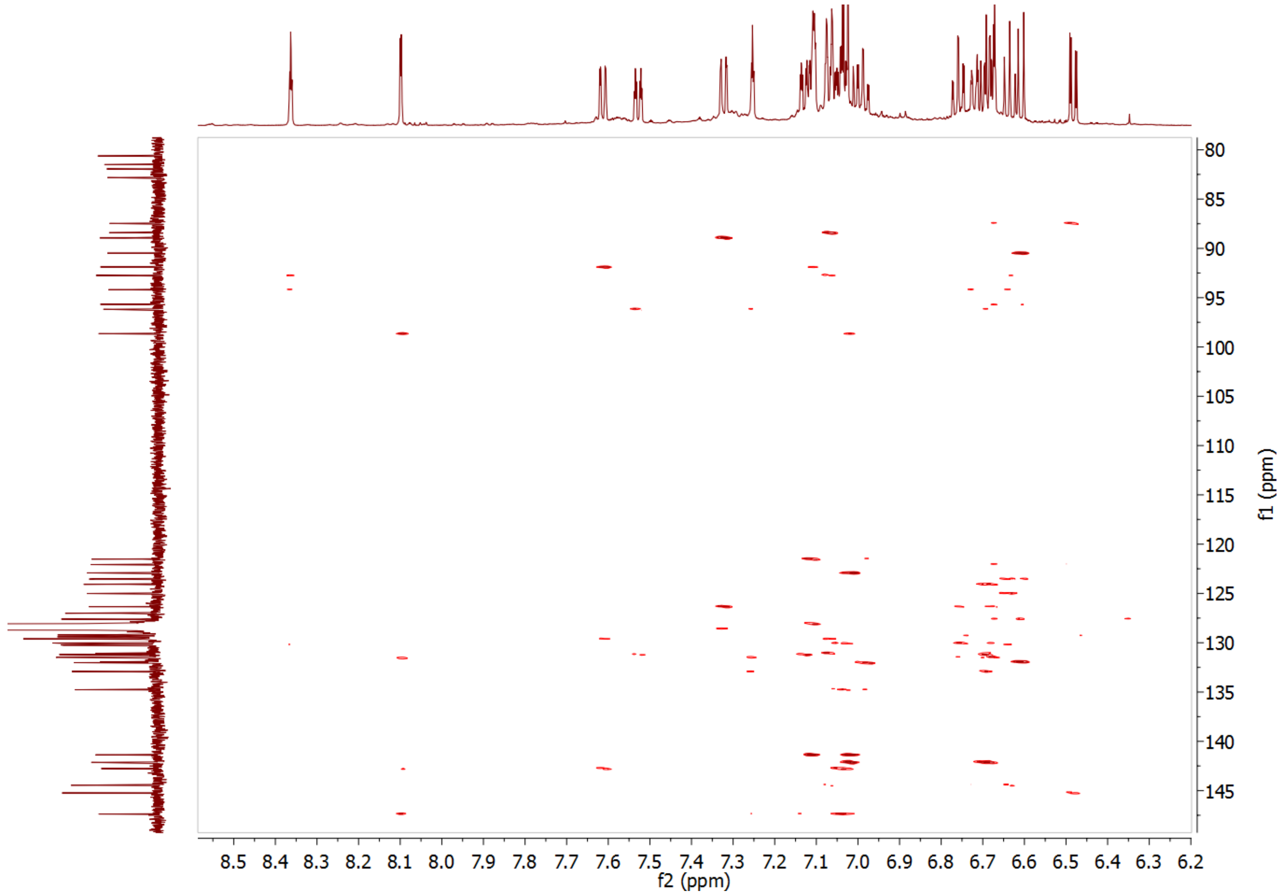

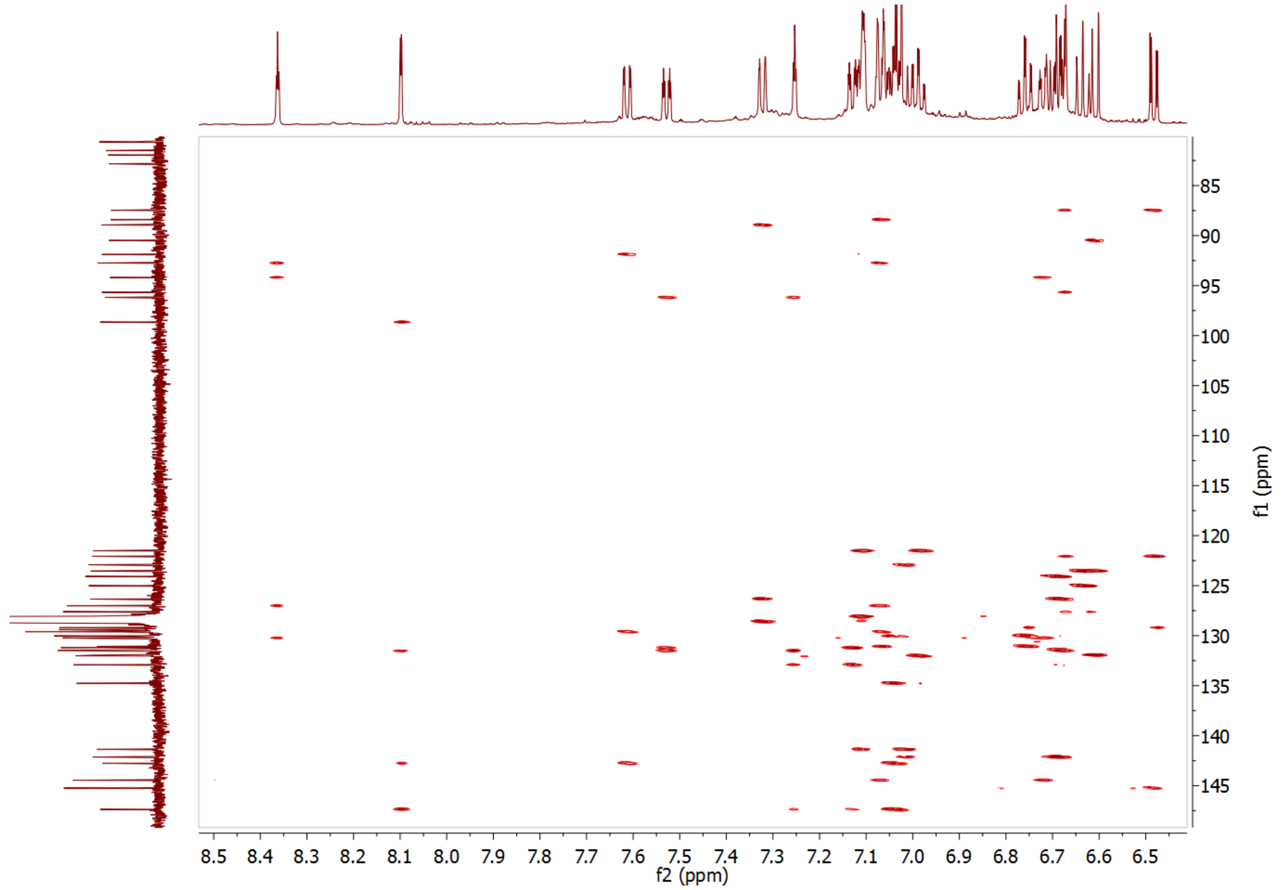

HMBC in C_6D_6, 600 MHz, 298 K, d6 = 50 ms (above)

HSQC in C_6D_6, 600 MHz, 298 K, (below)

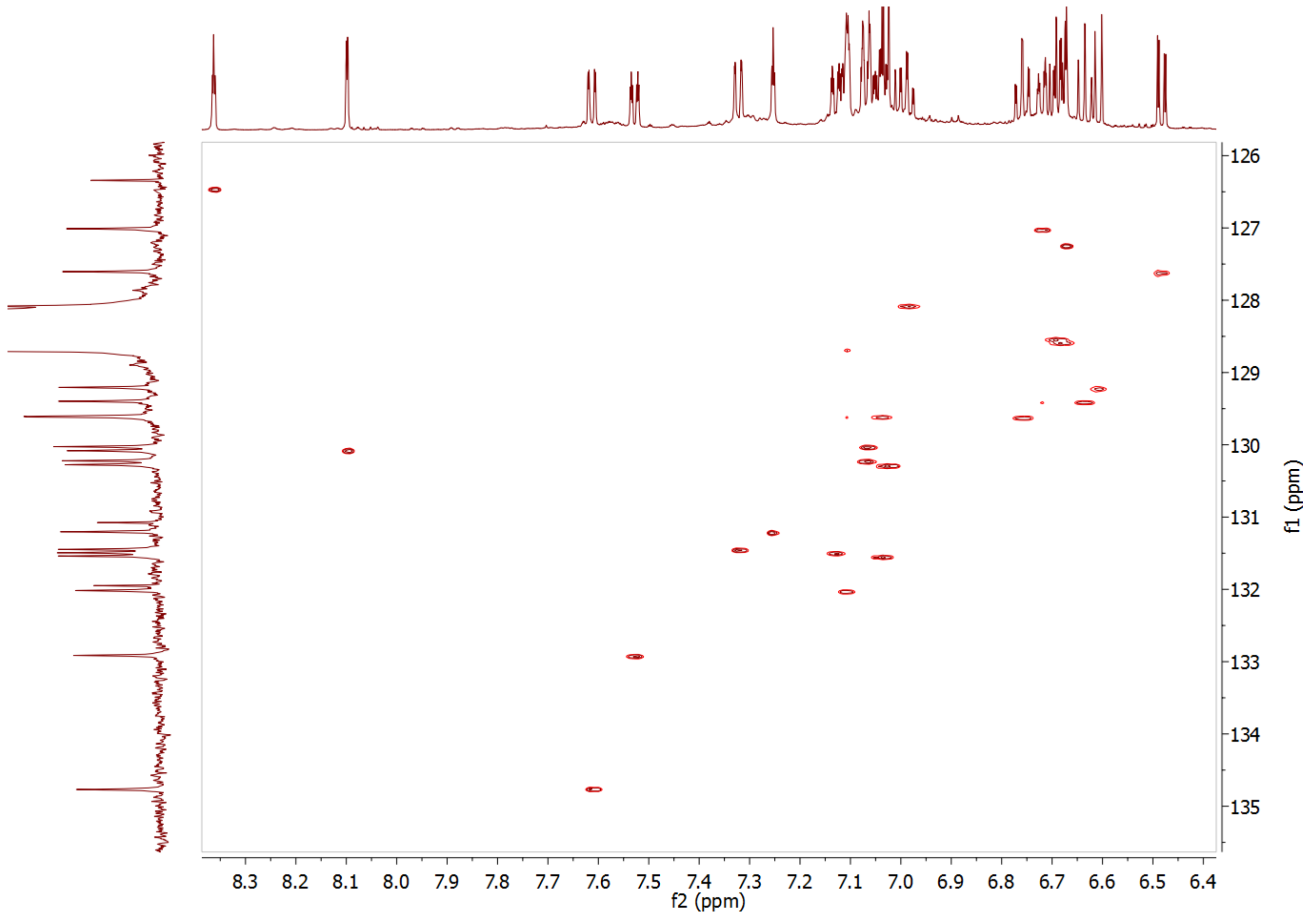

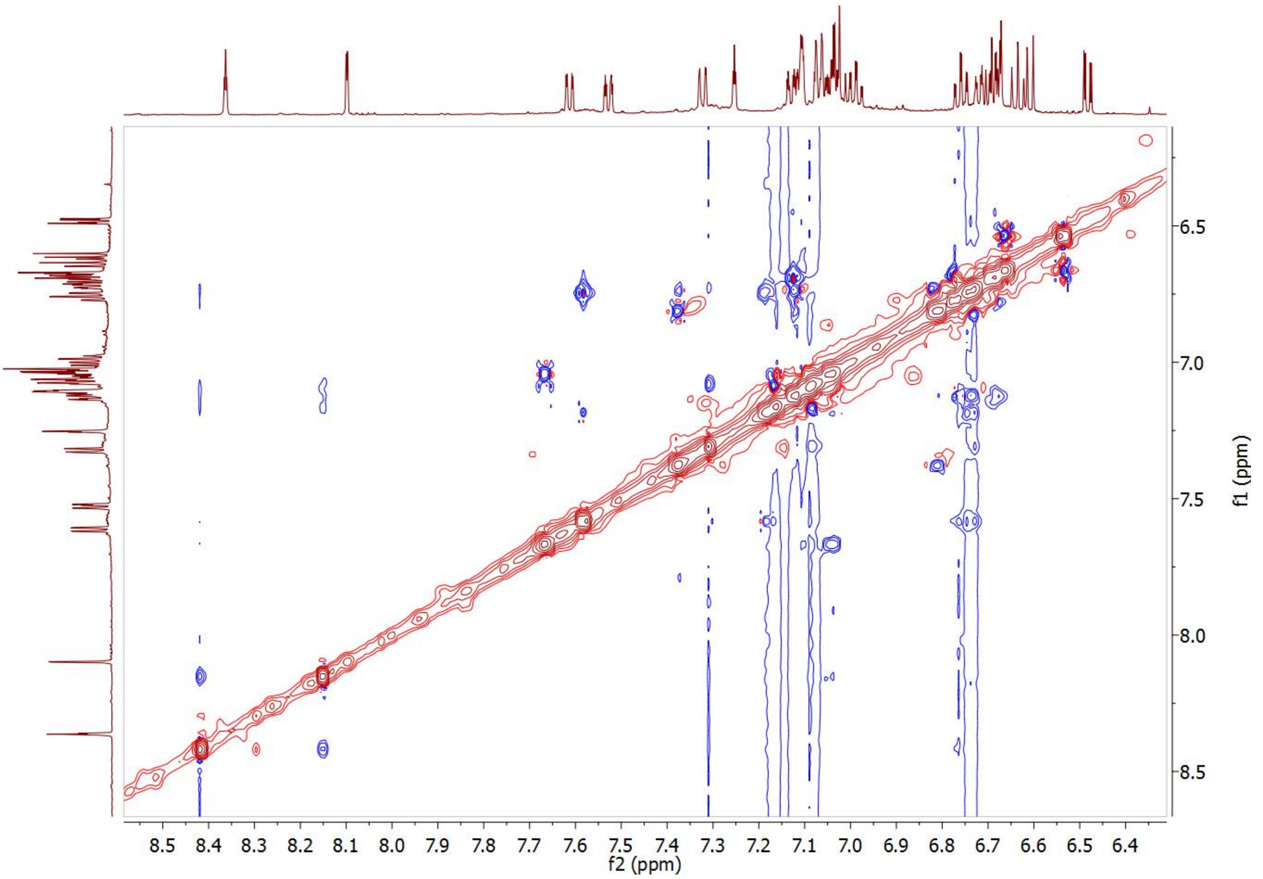

NOESY in C_6D_6, 600 MHz, 298 K (above)

TOSCY in C_6D_6, 600 MHz, 298 K (below)

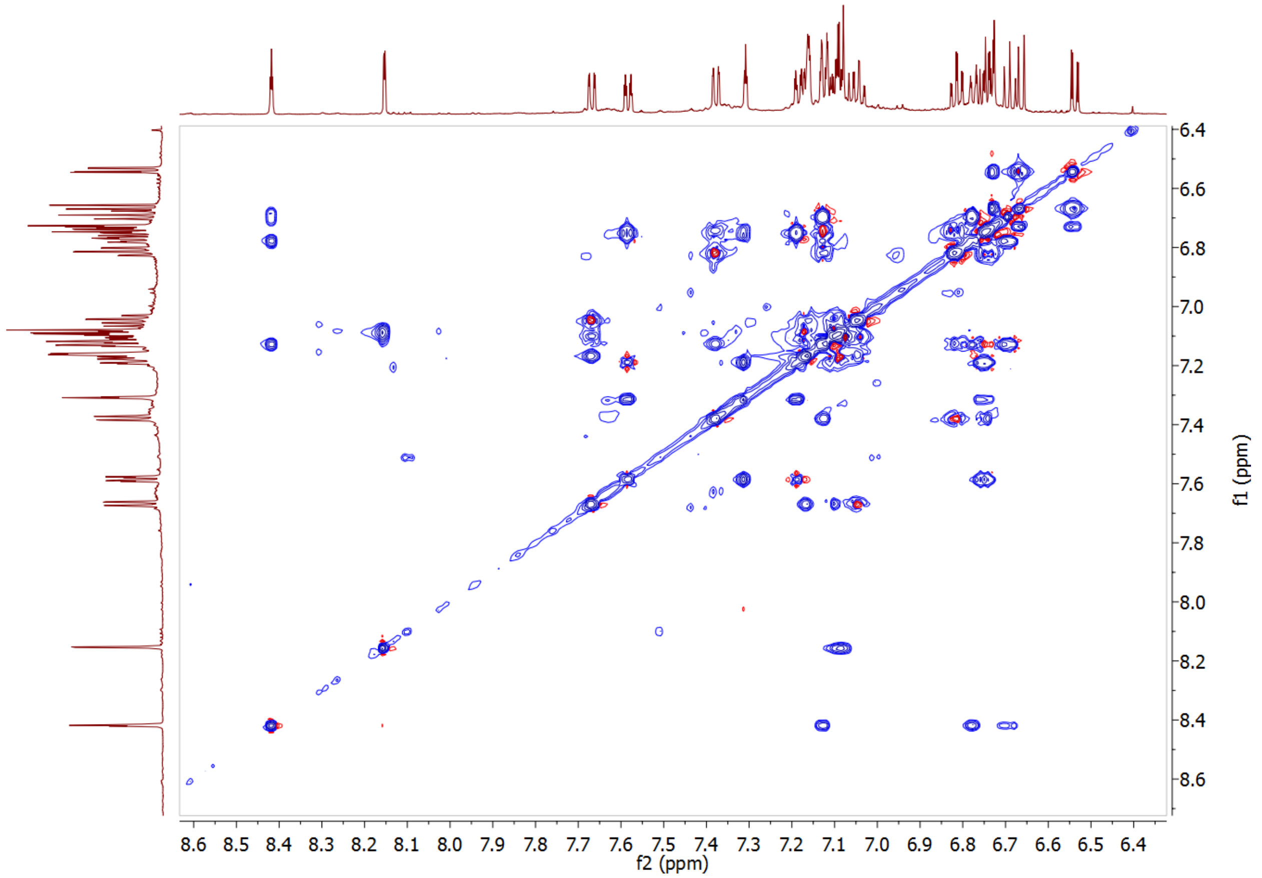

COSY in C_6D_6, 600 MHz, 298 K (below)

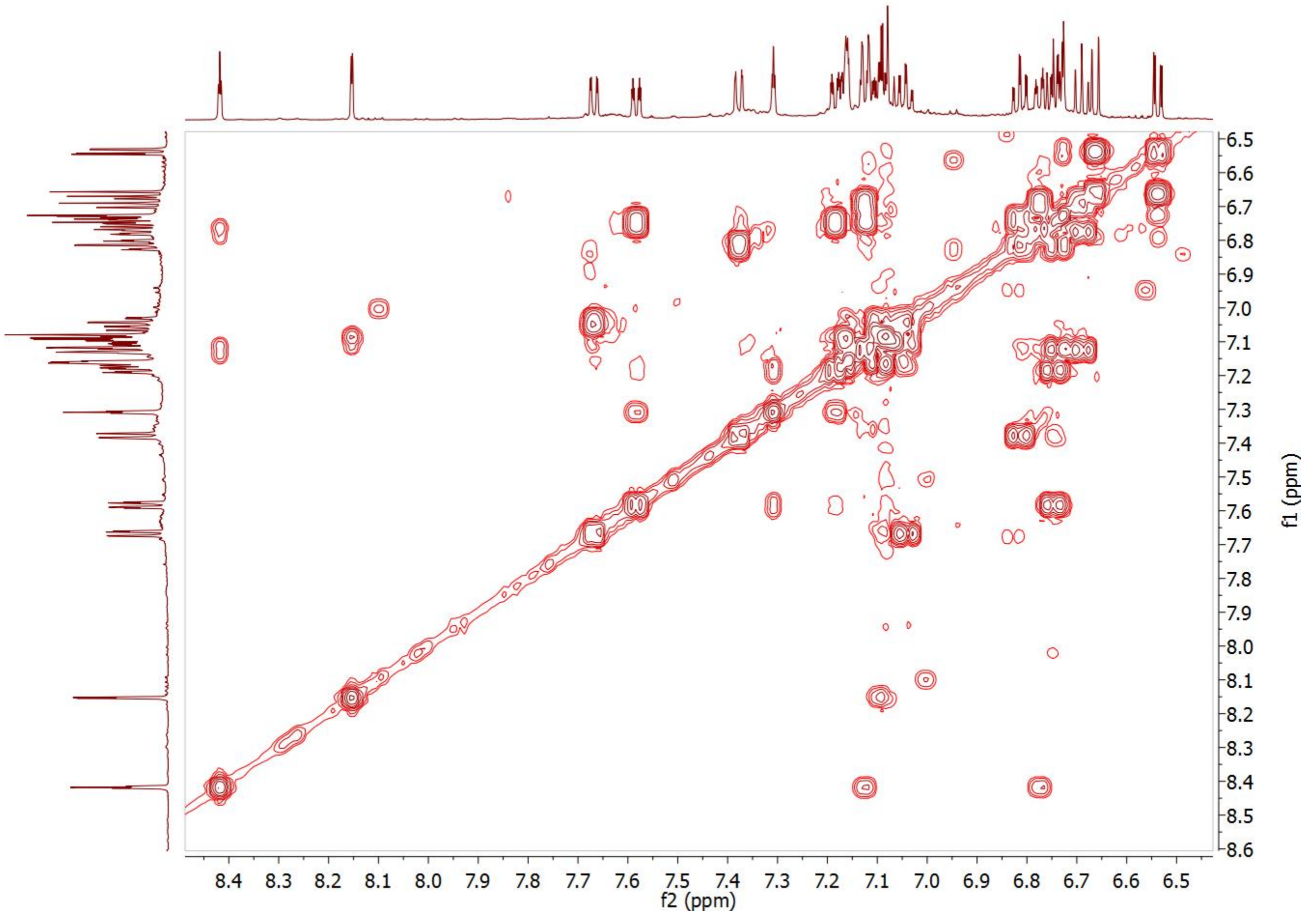

High Resolution Mass Spectrometry Report

Sample Name	**Linda Bannwart / Ba618 chr1#1**	Instrument	maXis 4G
Comment	10 ug / mL in MeOH	Method	22 Direct_pos_mid.m

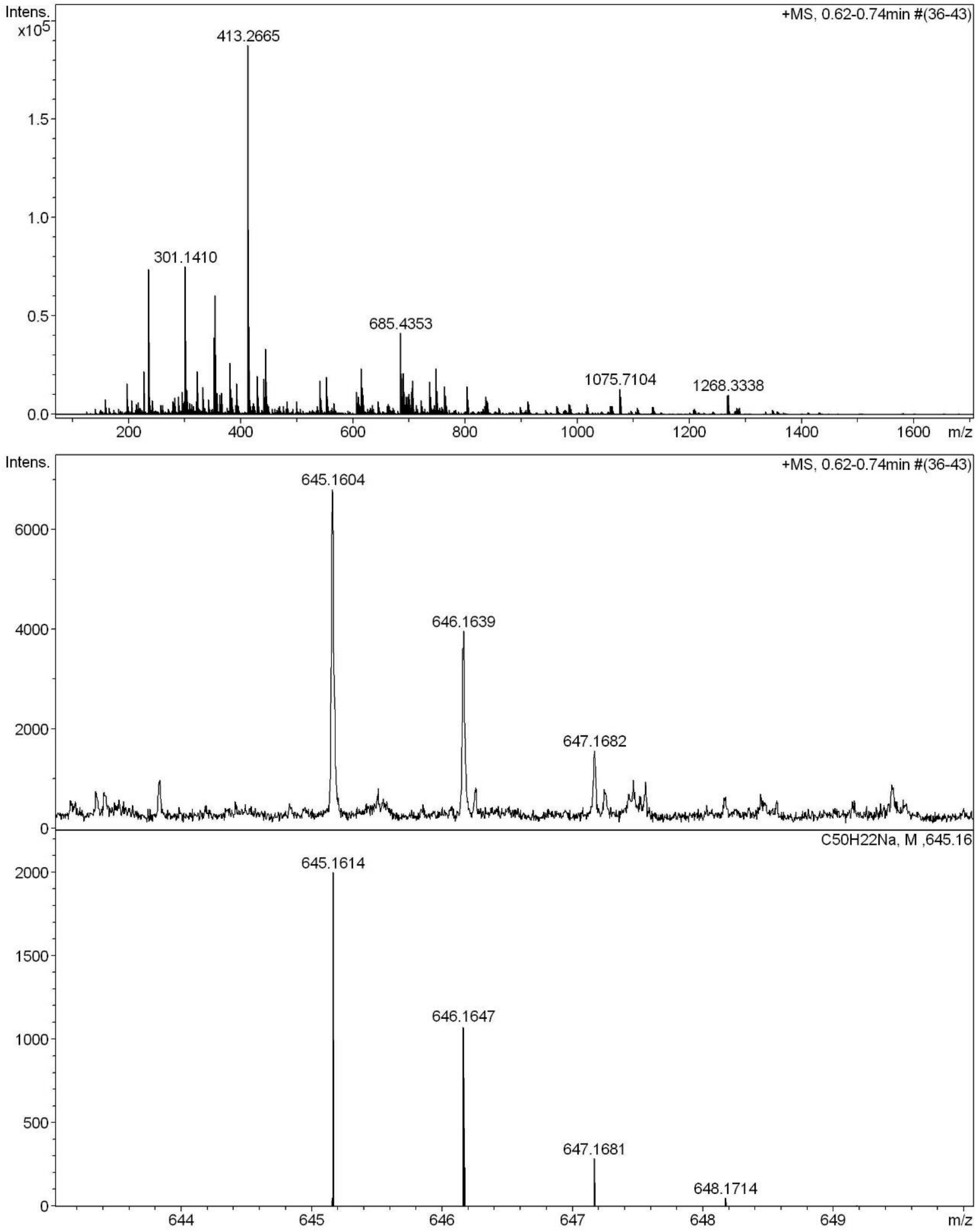

High Resolution Mass Spectrometry Report

Measured m/z vs. theoretical m/z

Meas. m/z	#	Formula	Score	m/z	err [mDa]	err [ppm]	mSigma	rdb	e⁻ Conf	z
645.1604	1	C 50 H 22 Na	100.00	645.1614	1.0	1.5	50.1	39.5	even	1+

Mass list

#	m/z	I %	I
1	158.9640	4.1	7707
2	198.0039	8.3	15658
3	205.0598	3.8	7173
4	217.1046	3.3	6216
5	226.9513	11.8	22204
6	236.0715	39.4	73885
7	237.0744	4.9	9136
8	242.2840	3.9	7366
9	279.1571	3.5	6604
10	280.0767	3.0	5672
11	283.0389	4.7	8741
12	288.9218	4.8	9079
13	294.9389	6.2	11653
14	299.1615	3.7	6920
15	300.9921	4.8	9028
16	301.1410	40.1	75211
17	302.1443	7.1	13403
18	304.2611	6.7	12552
19	309.2034	3.2	5933
20	321.2761	3.5	6607
21	322.9741	11.7	21848
22	332.9640	7.4	13888
23	342.0590	4.2	7887
24	353.2660	20.9	39253
25	354.2693	4.9	9149
26	354.9462	32.3	60559
27	355.9487	5.6	10462
28	356.9422	5.9	11089
29	362.9260	5.3	9899
30	363.0984	4.6	8571
31	365.2659	6.0	11209
32	381.2974	14.1	26467
33	382.3009	3.6	6786
34	384.2430	4.7	8870
35	393.2973	8.4	15842
36	413.2665	100.0	187529
37	414.2696	24.0	44980
38	415.2723	4.1	7646
39	423.0650	3.1	5741
40	429.2186	10.4	19508
41	430.2222	3.4	6308
42	430.9139	4.5	8483
43	441.2974	9.8	18399
44	442.3007	3.0	5583
45	445.0473	17.8	33401
46	445.1926	3.2	5950
47	446.0501	5.2	9710
48	447.0440	2.9	5442
49	483.3441	3.7	6878
50	498.9008	3.6	6722
51	541.1206	9.1	17089
52	542.1213	4.2	7860
53	543.1191	3.1	5854
54	553.4586	10.1	18993
55	554.4622	4.3	8017
56	566.8885	3.2	5962
57	607.0422	6.2	11585
58	609.0404	4.1	7725
59	610.1836	4.9	9199
60	615.1392	12.4	23326
61	616.1401	7.4	13870
62	617.1378	5.3	10023

#	m/z	I %	I
63	645.1604	3.6	6814
64	663.4535	2.9	5399
65	684.2024	3.3	6163
66	685.4353	22.1	41385
67	686.4383	10.0	18707
68	687.4418	2.9	5416
69	687.5313	3.4	6441
70	689.1579	11.3	21253
71	690.1587	7.1	13330
72	691.1564	5.6	10538
73	691.4172	4.1	7750
74	692.1569	2.9	5464
75	695.5274	4.8	9062
76	697.5388	4.9	9241
77	699.5673	5.7	10637
78	700.5711	2.9	5450
79	705.5819	9.2	17280
80	706.5853	4.3	7992
81	721.5764	3.8	7190
82	736.5421	8.9	16669
83	737.5459	4.9	9207
84	748.5421	12.6	23562
85	749.5453	6.3	11808
86	763.1769	7.6	14267
87	764.1771	5.2	9771
88	765.1760	4.1	7710
89	803.5423	7.8	14641
90	804.5458	4.1	7650
91	837.1948	4.9	9210
92	838.1959	3.9	7330
93	839.1937	3.3	6259
94	911.2139	3.6	6779
95	912.2145	3.1	5859
96	1017.3909	2.9	5434
97	1075.7104	7.0	13102
98	1076.7145	4.9	9152
99	1267.3308	5.1	9530
100	1268.3338	5.4	10124

Acquisition Parameter

**General**	Fore Vacuum	2.59e+000 mBar	High Vacuum	1.26e-007 mBar	Source Type	ESI
	Scan Begin	75 m/z	Scan End	1700 m/z	Ion Polarity	Positive
**Source**	Set Nebulizer	0.4 Bar	Set Capillary	3600 V	Set Dry Gas	4.0 l/min
	Set Dry Heater	180 °C	Set End Plate Offset	-500 V		
**Quadrupole**	Set Ion Energy (MS only)	4.0 eV				
**Coll. Cell**	Collision Energy	8.0 eV	Set Collision Cell RF	350.0 Vpp		
**Ion Cooler**	Set Ion Cooler Transfer Time	75.0 µs	Set Ion Cooler Pre Pulse Storage Time	10.0 µs		

Acquisition Parameter

Method:	MALDI_MS_POS_100-1000_2M_16AvScans			Acquisition Date:	25.09.2019 13:57:30
File Name:	D:\ETH\Data\BSOL0006xx\BSOL000608_0_K2_000001.d			Operator:	
Source	Dual (MALDI/ESI)	Polarity	Positive	Nebulizer Gas	1.0 bar
Broadband Low Mass	77.0 m/z	n/a	n/a	Drying Gas Flow Rate	3.7 L/min
Broadband High Mass	1050.0 m/z	Laser Power	27.8 lp	Capillary	3000.0 V
No. of Cell Fills	1	n/a	n/a	Drying Gas Temperature	200.0 °C
Apodization	Full-Sine	Time of Flight to Detector	0.001 sec		

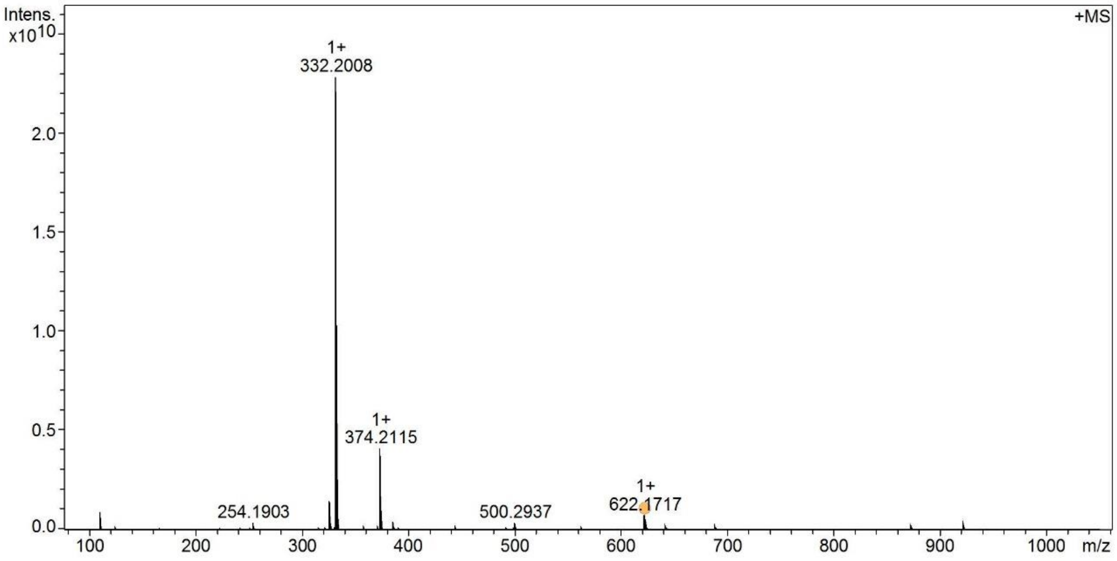

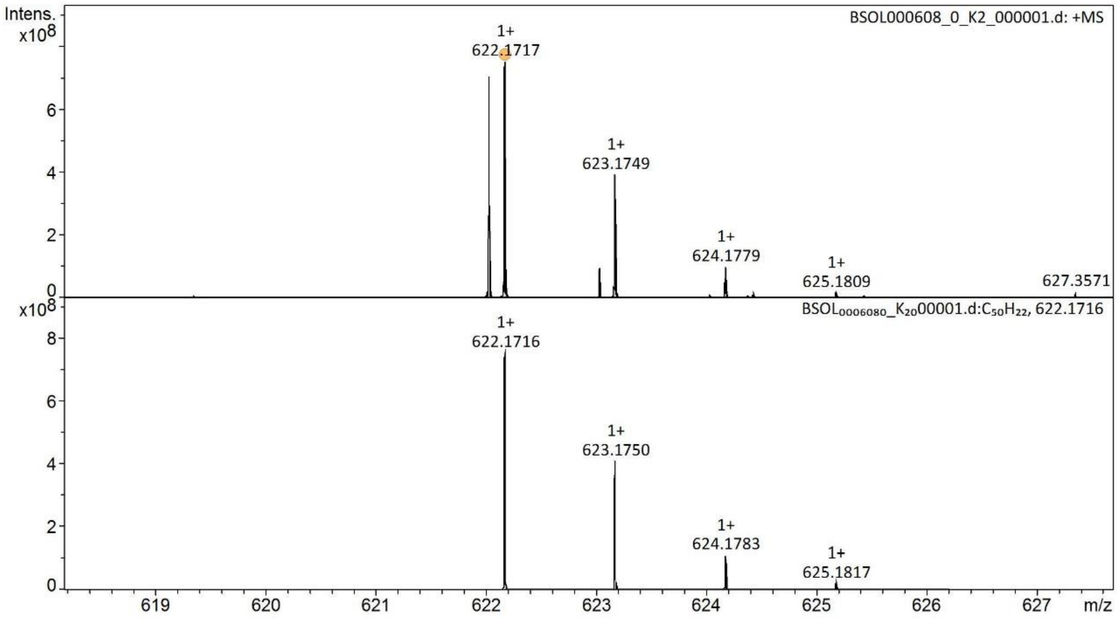

Evaluation Spectra / Validation Formula:

#	Ion Formula	Adduct	m/z	z	Meas. m/z	mSigma	N-Rule	err [mDa]	err [ppm]
1	C50H22	M	622.1716	1+	622.1717	12.5	ok	-0.1	-0.2

Calibration Info:

Date: 25.09.2019 14:13:22
Polarity: Positive
Calibration spectrum: +MS: Scan
Reference mass list: MALDI: DCTB Matrix + HP-Mix (pos)
Calibration mode: Quadratic

Reference m/z	Resulting m/z	Intensity	Error [ppm]
118.0863			
250.1464			
251.1543	251.1543	44127436	0.078
273.1362	273.1362	25189142	0.100
322.0481	322.0481	101148288	-0.148
332.2009	332.2008	22846550016	-0.380
500.2934	500.2937	354490720	0.583
501.3013			
523.2832			
622.0290	622.0291	708280320	0.183
750.4404	750.4405	20394604	0.011
751.4483	751.4484	34609860	0.185
773.4302			
922.0098	922.0091	224358832	-0.733
1000.5874			
1001.5953			
1023.5772			
1221.9906			
1521.9715			
1821.9523			
2121.9332			
2421.9140			
2721.8948			

Standard deviation: 0.447

Mass List:

#	m/z	Res.	S/N	I %	FWHM
1	242.2842	198448	1092.1	0.4	0.0012
2	254.1903	190920	4221.8	1.7	0.0013
3	255.1937	188655	797.8	0.3	0.0014
4	316.1697	148793	817.2	0.4	0.0021
5	322.0481	145290	967.8	0.4	0.0022
6	326.3780	145821	13910.5	6.4	0.0022
7	327.3814	143271	3375.5	1.5	0.0023
8	331.1930	137883	914.0	0.4	0.0024
9	332.1898	205934	2417.7	1.1	0.0016
10	332.2008	147740	218969.5	100.0	0.0022
11	333.1931	223890	643.4	0.3	0.0015
12	333.2041	145224	54651.9	25.0	0.0023
13	334.2074	139633	5608.3	2.6	0.0024
14	358.1915	132474	1295.8	0.6	0.0027
15	372.2322	127623	1444.8	0.7	0.0029
16	374.2115	129866	39432.0	18.0	0.0029
17	375.2148	127740	10285.0	4.7	0.0029
18	376.2183	128084	1196.8	0.5	0.0029
19	386.2843	123208	3824.3	1.7	0.0031
20	387.2877	122690	1096.7	0.5	0.0032
21	391.2844	121534	806.5	0.4	0.0032
22	444.2900	109229	1329.6	0.6	0.0041
23	492.2901	98973	580.5	0.3	0.0050
24	499.2859	98032	768.3	0.4	0.0051
25	500.2937	98777	3031.8	1.6	0.0051
26	501.2969	82934	1051.7	0.5	0.0060
27	501.3018	94529	995.3	0.5	0.0053
28	563.2234	86814	787.4	0.5	0.0065
29	622.0291	78957	5132.2	3.1	0.0079
30	622.1717	78954	5461.4	3.3	0.0079
31	623.0326	80031	673.9	0.4	0.0078
32	623.1749	82806	2856.4	1.7	0.0075
33	624.1779	86389	709.0	0.4	0.0072
34	642.1780	76349	1409.4	0.9	0.0084
35	643.1812	78826	722.4	0.4	0.0082
36	688.4010	71012	1365.9	0.8	0.0097
37	689.4046	71812	654.0	0.4	0.0096
38	872.3180	56102	1295.6	0.8	0.0155
39	873.3220	54919	983.9	0.6	0.0159
40	922.0091	53083	1682.0	1.0	0.0174

#	m/z	Res.	S/N	I %	FWHM
1	622.1716	78954		100.0	0.0079
2	623.1750	79081		54.3	0.0079
3	624.1783	79208		14.5	0.0079
4	625.1817	79336		2.5	0.0079
5	626.1851	79463		0.3	0.0079

5.37 Compound **41**

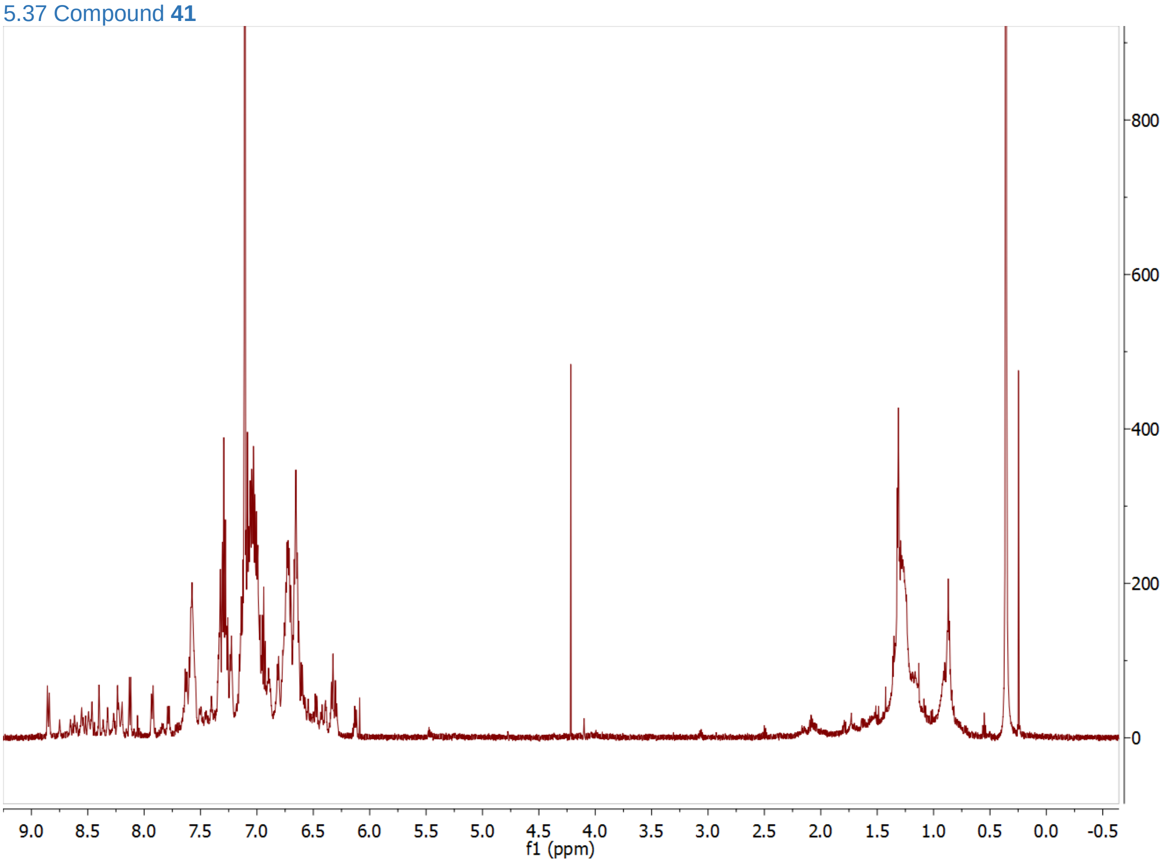

^{1}H-NMR in C_6D_6

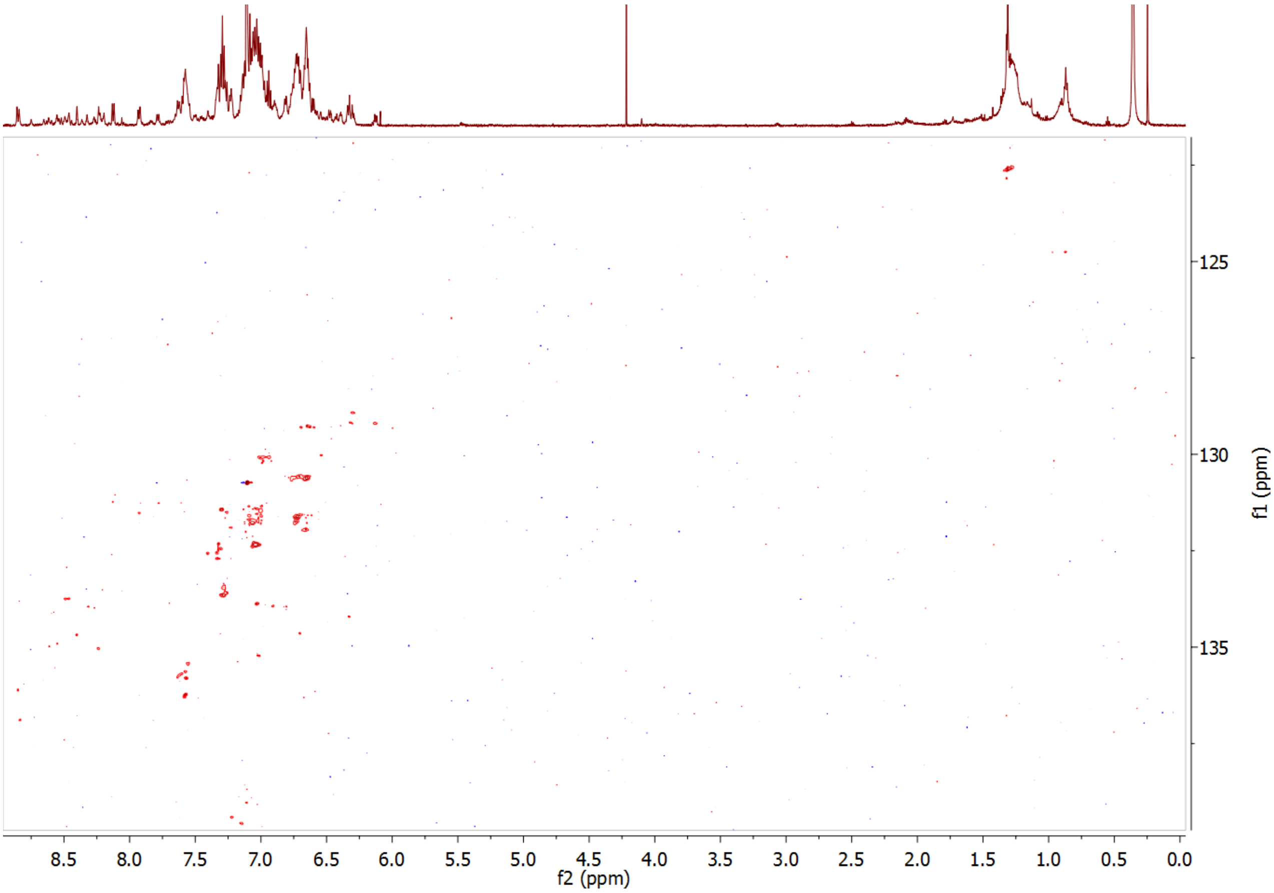

HMBC d6=100 ms in C_6D_6

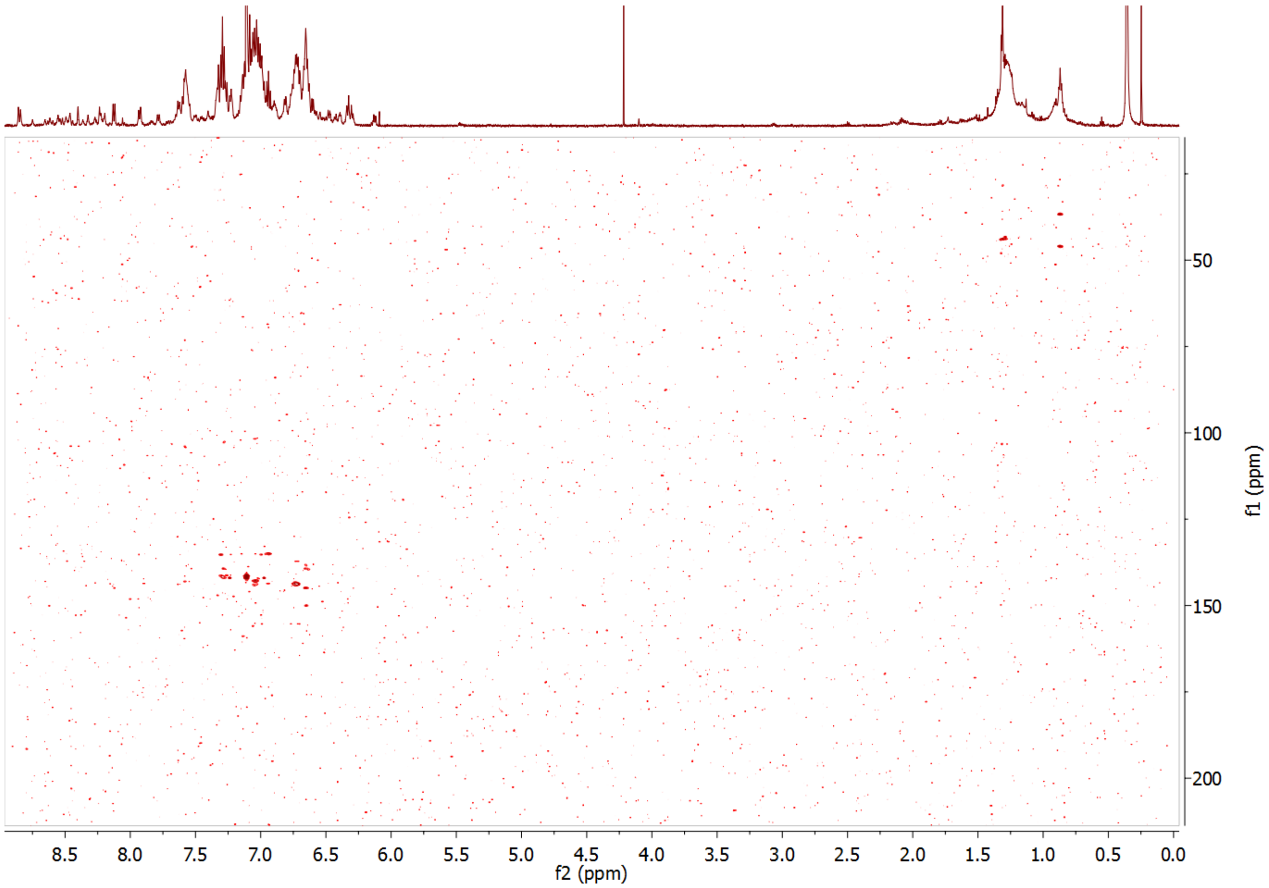

HSQC in C_6D_6

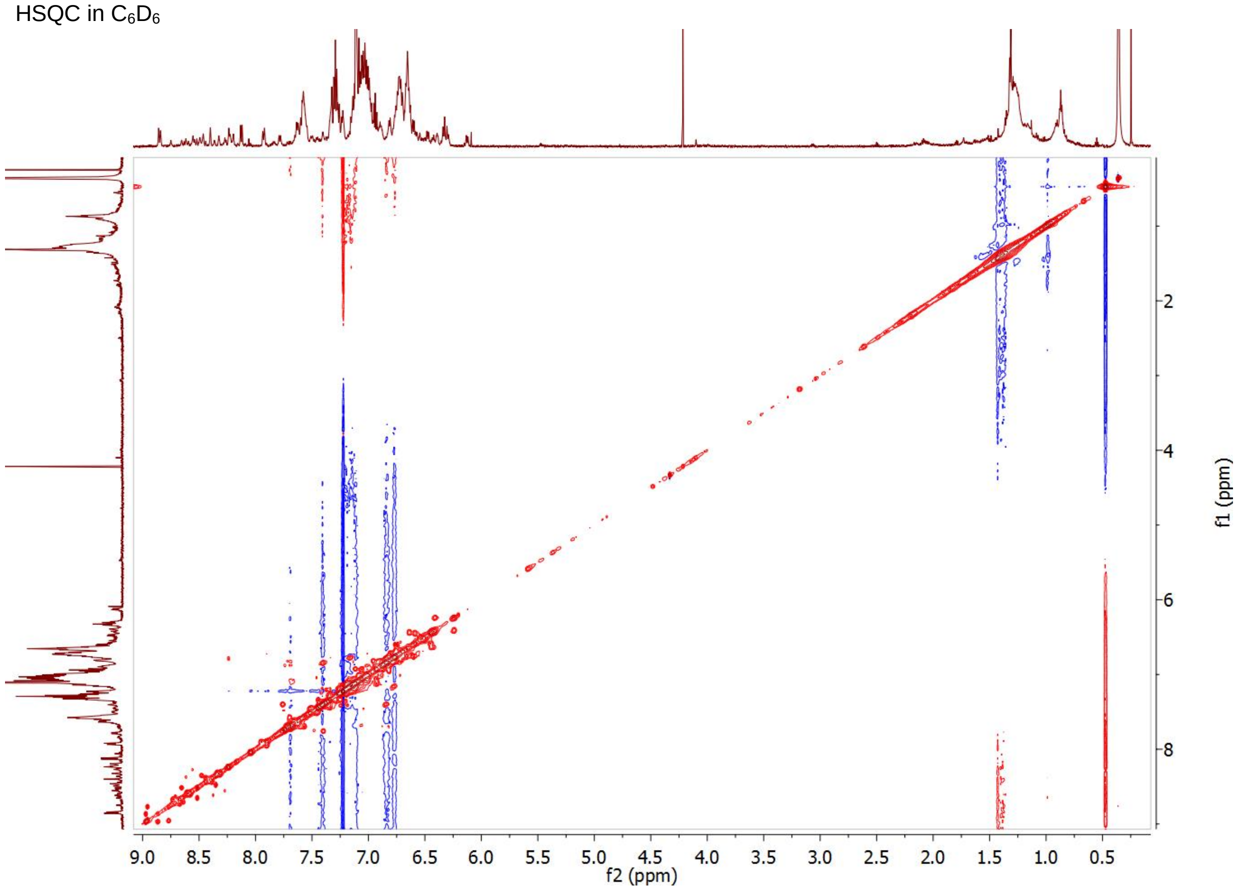

NOESY in C_6D_6

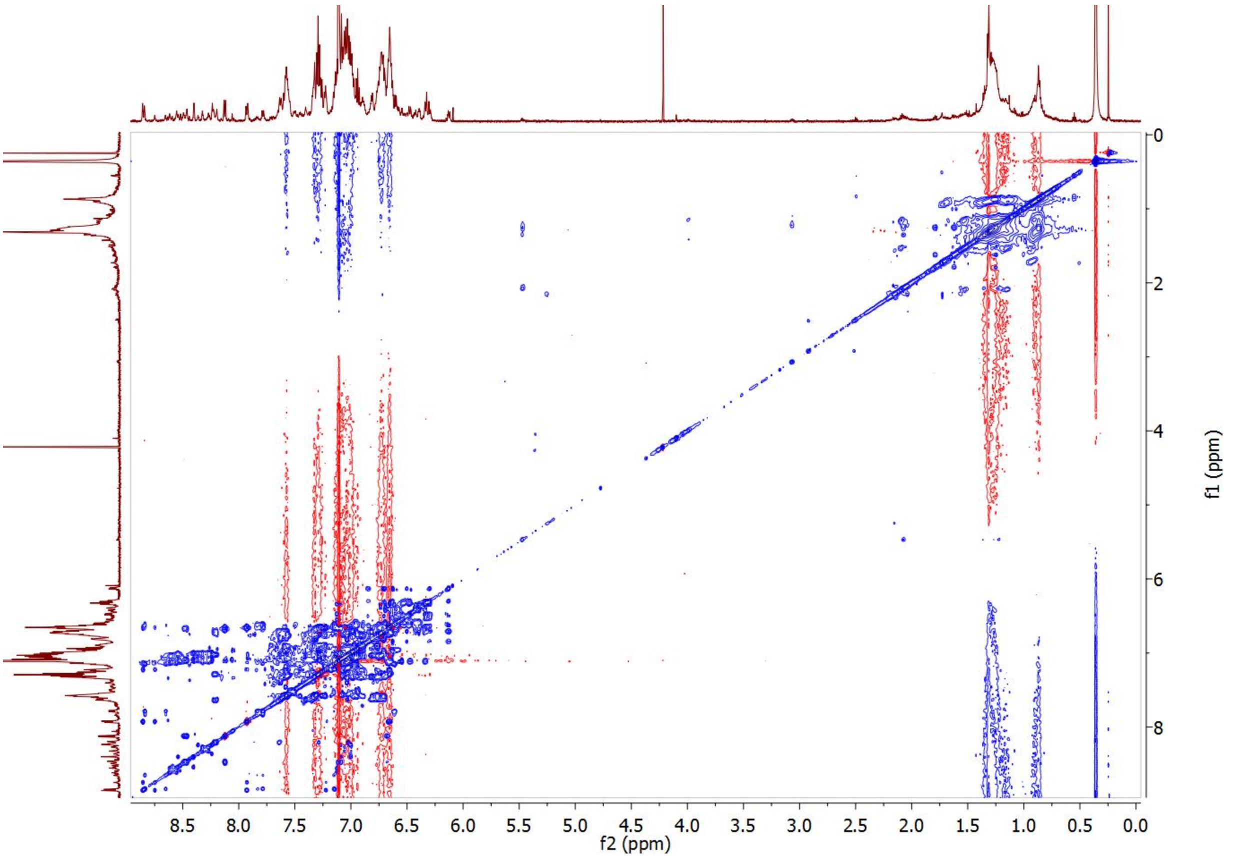

TOSCY in C_6D_6

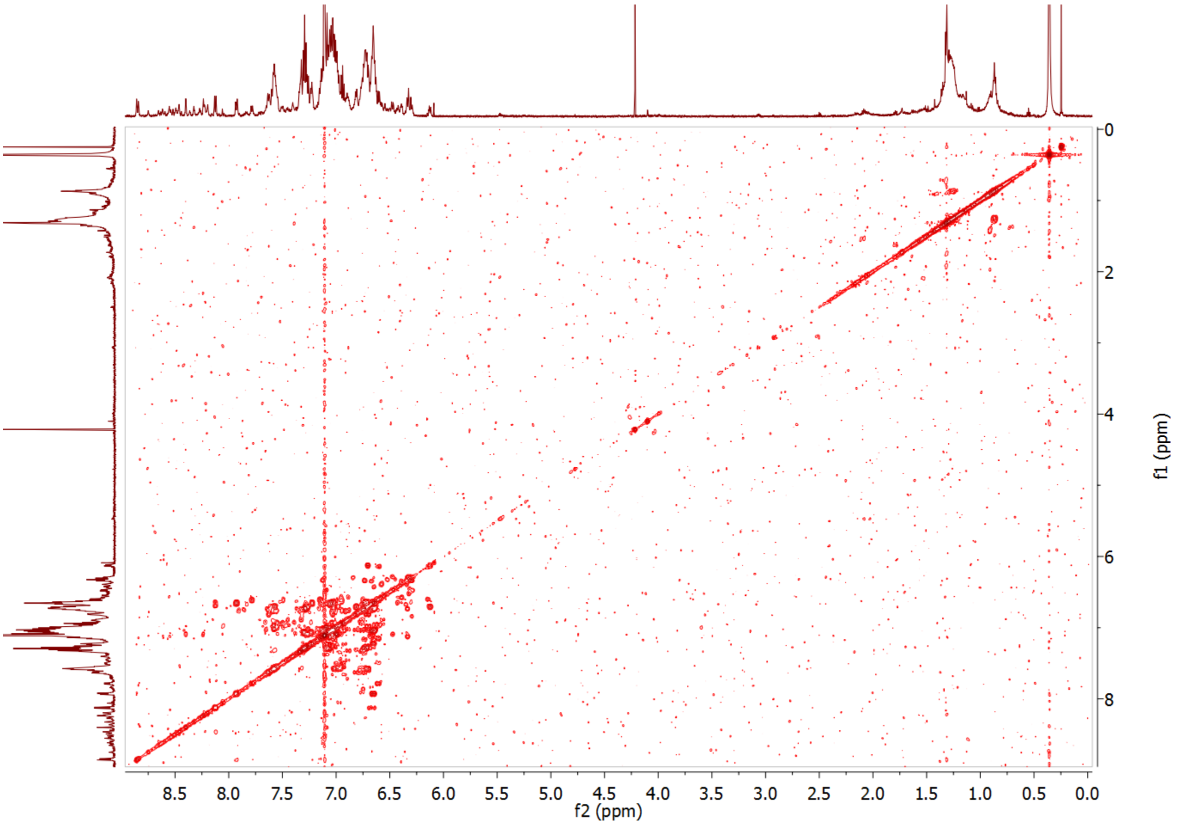

COSY in C_6D_6

Acquisition Parameter

Method:	MALDI_MS_POS_300-2600_2M_16AvScans			Acquisition Date:	25.09.2019 14:11:26
File Name:	D:\ETH\Data\BSOL0006xx\BSOL000609_0_K6_000001.d			Operator:	
Source	Dual (MALDI/ESI)	Polarity	Positive	Nebulizer Gas	1.3 bar
Broadband Low Mass	303.1 m/z	n/a	n/a	Drying Gas Flow Rate	3.7 L/min
Broadband High Mass	2600.0 m/z	Laser Power	23.6 lp	Capillary	3000.0 V
No. of Cell Fills	1	n/a	n/a	Drying Gas Temperature	200.0 °C
Apodization	Full-Sine	Time of Flight to Detector	0.001 sec		

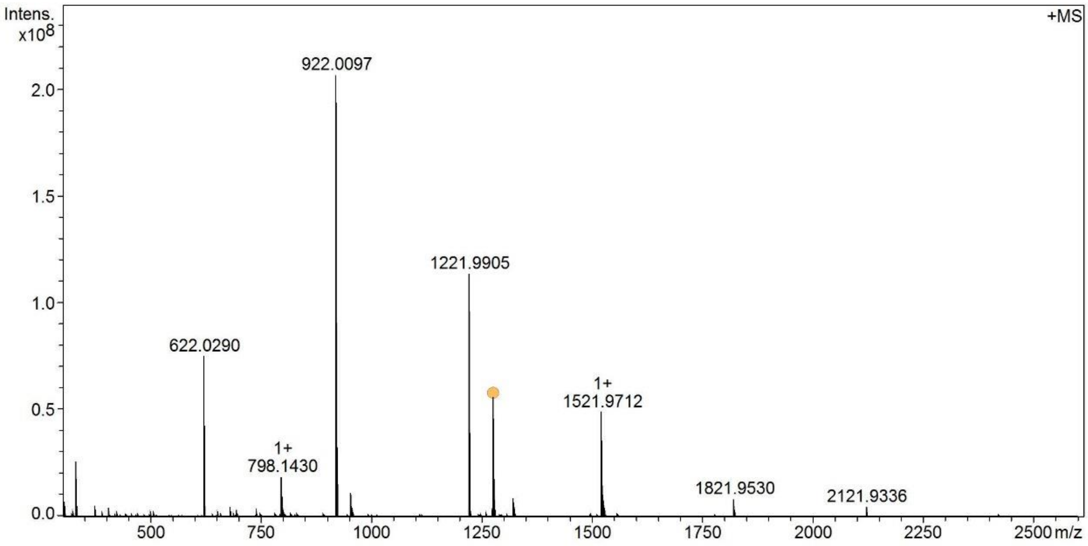

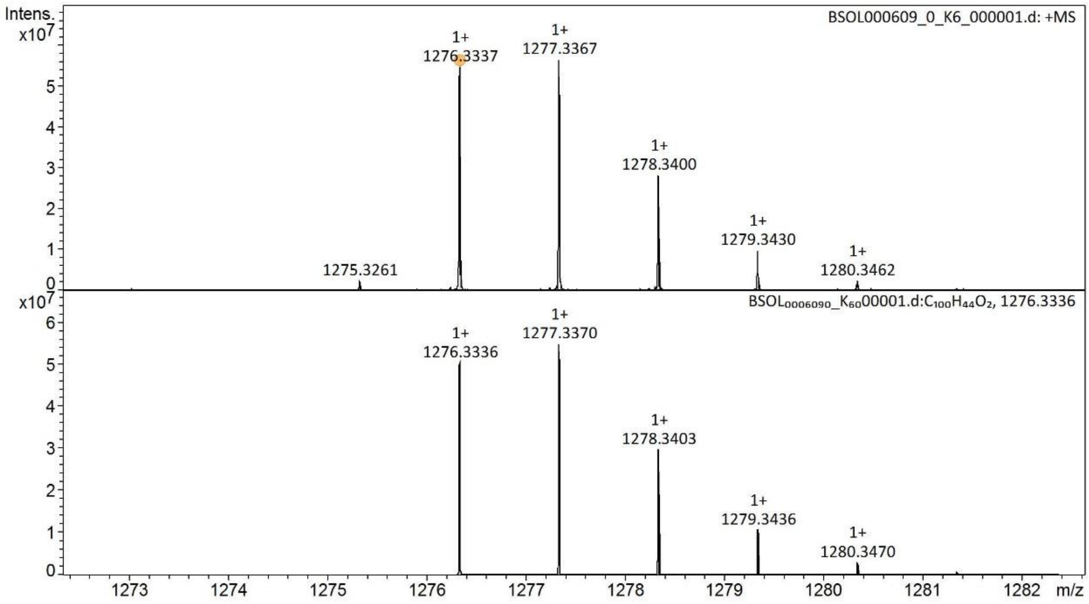

Evaluation Spectra / Validation Formula:

#	Ion Formula	Adduct	m/z	z	Meas. m/z	mSigma	N-Rule	err [mDa]	err [ppm]
1	C100H44O2	M	1276.3336	1+	1276.3337	30.7	ok	-0.1	-0.1

Calibration Info:

Date: 25.09.2019 14:15:24
Polarity: Positive
Calibration spectrum: +MS: Scan
Reference mass list: MALDI: DCTB Matrix + HP-Mix (pos)
Calibration mode: Quadratic

Reference m/z	Resulting m/z	Intensity	Error [ppm]
118.0863			
250.1464			
251.1543			
273.1362			
322.0481			
332.2009	332.2009	26289486	-0.005
500.2934			
501.3013			
523.2832			
622.0290	622.0290	75399072	0.069
750.4404			
751.4483			
773.4302			
922.0098	922.0097	206905888	-0.074
1000.5874			
1001.5953			
1023.5772			
1221.9906	1221.9905	113774296	-0.095
1521.9715	1521.9712	49366668	-0.210
1821.9523	1821.9530	8368600	0.366
2121.9332			
2421.9140			
2721.8948			

Standard deviation: 0.270

Mass List:

#	m/z	Res.	S/N	I %	FWHM
1	332.2003	570808	312.0	3.7	0.0006
2	332.2009	621388	1064.0	12.7	0.0005
3	333.2043	624862	278.0	3.3	0.0005
4	374.2114	455751	215.9	2.6	0.0008
5	622.0290	310375	2469.3	36.4	0.0020
6	623.0324	307398	281.3	4.2	0.0020
7	680.4803	279378	143.5	2.2	0.0024
8	696.5258	273287	91.8	1.4	0.0025
9	740.5520	254216	73.3	1.2	0.0029
10	798.1430	241654	544.4	9.1	0.0033
11	799.1425	185312	112.2	1.9	0.0043
12	799.1465	200015	107.2	1.8	0.0040
13	800.1398	258334	85.7	1.5	0.0031
14	922.0097	212669	5597.4	100.0	0.0043
15	923.0131	209112	916.6	16.7	0.0044
16	924.0173	207117	71.5	1.3	0.0045
17	954.1649	204952	293.4	5.5	0.0047
18	955.1642	250106	82.0	1.6	0.0038
19	955.1690	260648	68.5	1.3	0.0037
20	1221.9905	158173	2560.8	55.0	0.0077
21	1222.9861	215109	89.7	2.0	0.0057
22	1222.9942	158471	611.0	13.2	0.0077
23	1223.9976	149077	72.2	1.6	0.0082
24	1276.3337	150946	1208.9	26.5	0.0085
25	1277.3367	151968	1249.2	27.4	0.0084
26	1278.3400	151051	623.1	13.7	0.0085
27	1278.3482	180992	97.8	2.2	0.0071
28	1279.3430	149226	214.7	4.7	0.0086
29	1322.3756	140292	170.2	3.8	0.0094
30	1323.3788	145804	188.4	4.2	0.0091
31	1324.3820	142154	100.7	2.3	0.0093
32	1521.9712	127668	1010.0	23.9	0.0119
33	1522.9749	127497	297.0	7.0	0.0119
34	1523.9779	121214	58.7	1.4	0.0126
35	1526.4808	124204	122.9	2.9	0.0123
36	1527.4844	121204	157.7	3.8	0.0126
37	1528.4879	115247	85.7	2.1	0.0133
38	1821.9530	102448	152.4	4.0	0.0178
39	1822.9563	107247	55.4	1.5	0.0170
40	2121.9336	89599	77.7	2.3	0.0237

#	m/z	Res.	S/N	I %	FWHM
1	1276.3336	150946		92.0	0.0085
2	1277.3370	151064		100.0	0.0085
3	1278.3403	151183		54.2	0.0085
4	1279.3436	151302		19.5	0.0085
5	1280.3470	151420		5.3	0.0085
6	1281.3503	151539		1.1	0.0085
7	1282.3535	151658		0.2	0.0085

5.38 List of DEPT-135 Signals

Compound **6**: DEPT-135 (126 MHz, CD_2Cl_2) δ = 141.3 (1C, C_{Ar}), 137.3 (1C, C_{Ar}), 120.4 (1C, C_{Ar}), 51.9 (1C, CH_2), 47.6 (1C, CH_2), 24.5 (1C, CH_2), 24.0 (1C, CH_2).

Compound **7**: DEPT-135 (126 MHz, CD_2Cl_2) δ = 134.3 (1C, C_{Ar}), 131.8 (1C, C_{Ar}), 130.5 (1C, C_{Ar}), 129.7 (1C, C_{Ar}), 129.3 (1C, C_{Ar}), 127.5 (1C, C_{Ar}), 118.3 (1C, C_{Ar}), 51.8 (1C, CH_2), 47.5 (1C, CH_2), 24.5 (1C, CH_2), 24.1 (1C, CH_2).

Compound **8**: DEPT135 (126 MHz, CD_2Cl_2) δ = 140.5 (1C, C_{Ar}), 134.0 (1C, C_{Ar}), 131.6 (1C, C_{Ar}), 130.6 (1C, C_{Ar}), 130.3 (1C, C_{Ar}), 130.0 (1C, C_{Ar}), 127.7 (1C, C_{Ar}).

Compound **9**: DEPT-135 (126 MHz, CD_2Cl_2) δ = 134.3 (1C, C_{Ar}), 131.9 (1C, C_{Ar}), 131.3 (1C, C_{Ar}), 130.6 (1C, C_{Ar}), 129.7 (1C, C_{Ar}), 129.5 (1C, C_{Ar}), 129.1 (1C, C_{Ar}), 127.7 (1C, C_{Ar}), 120.0 (1C, C_{Ar}), 116.4 (1C, C_{Ar}), 114.8 (1C, C_{Ar}).

Compound **10**: DEPT-135 (126 MHz, CD_2Cl_2) δ = 138.2 (1C, C_{Ar}), 136.7 (1C, C_{Ar}), 133.9 (1C, C_{Ar}), 131.3 (1C, C_{Ar}), 130.7 (1C, C_{Ar}), 130.0 (1C, C_{Ar}), 129.7 (1C, C_{Ar}), 129.3 (1C, C_{Ar}), 128.8 (1C, C_{Ar}), 128.7 (1C, C_{Ar}), 127.1 (1C, C_{Ar}).

Compound **14**: DEPT-135 (101 MHz, $CDCl_3$) δ = 135.8 (1C, C_{Ar}), 133.7 (1C, C_{Ar}), 132.6 (1C, C_{Ar}), 31.4 (2C, CH_3).

Compound **15**: DEPT-135 (126 MHz, CD_2Cl_2) δ = 136.3 (1C, C_{Ar}), 133.5 (1C, C_{Ar}), 131.7 (1C, C_{Ar}), 31.8 (2C, CH_3), 21.9 (2C, CH_2), 21.3 (2C, CH_2), 18.7 (2C, CH_3), 18.5 (2C, CH_3), 18.4 (2C, CH_3), 18.3 (2C, CH_3), 12.3 (2C, CH), 12.3 (2C, CH), 10.1 (1C, CH_2), 10.1 (1C, CH_2).

Compound **16**: DEPT-135 (126 MHz, CD_2Cl_2) δ = 136.4 (1C, C_{Ar}), 132.9 (1C, C_{Ar}), 132.3 (1C, C_{Ar}), 82.4 (1C, $C_{alkyine}$), 21.9 (2C, CH_2), 21.8 (2C, CH_2), 21.3 (2C, CH_2), 21.2 (2C, CH_2), 18.5 (2C, CH_3), 18.5 (2C, CH_3), 18.3 (2C, CH_3), 18.3 (2C, CH_3), 12.3 (2C, CH), 12.2 (2C, CH), 10.1 (2C, CH_2), 10.1 (2C, CH_2). The peak of 82.4 is only visible in the DEPT-135 experiment with $^1J_{CH}$ coupling = 200 Hz.

Compound **17**: DEPT-135 (126 MHz, C_6D_6) δ = 134.3 (1C, C_{Ar}), 133.3 (1C, C_{Ar}), 133.0 (1C, C_{Ar}), 132.4 (1C, C_{Ar}), 131.6 (1C, C_{Ar}), 131.1 (1C, C_{Ar}), 131.0 (1C, C_{Ar}), 130.3 (1C, C_{Ar}), 130.1 (1C, C_{Ar}), 129.3 (1C, C_{Ar}), 129.0 (1C, C_{Ar}), 128.6 (1C, C_{Ar}), 128.5 (1C, C_{Ar}), 128.3 (1C, C_{Ar}), 127.2 (1C, C_{Ar}), 21.6 (1C, CH_2), 20.4 (1C, CH_2), 18.5 (2C, CH_3), 18.2 (2C, CH_3), 12.1 (2C, CH), 9.7 (1C, CH_2).

DEPT-135 (126 MHz, CD_2Cl_2) δ = 134.4 (1C, C_{Ar}), 133.3 (1C, C_{Ar}), 133.2 (1C, C_{Ar}), 132.6 (1C, C_{Ar}), 131.9 (1C, C_{Ar}), 131.3 (1C, C_{Ar}), 131.2 (1C, C_{Ar}), 130.6 (1C, C_{Ar}), 130.3 (1C, C_{Ar}), 129.8 (1C, C_{Ar}), 129.3 (1C, C_{Ar}), 129.0 (1C, C_{Ar}), 128.8 (1C, C_{Ar}), 128.7 (1C, C_{Ar}), 127.7 (1C, C_{Ar}), 21.9 (1C, CH_2), 21.2 (1C, CH_2), 18.6 (2C, CH_3), 18.3 (2C, CH_3), 12.3 (2C, CH), 10.1 (1C, CH_2).

Compound **18**: DEPT-135 (126 MHz, C_6D_6) δ = 135.9 (1C, C_{Ar}), 134.5 (1C, C_{Ar}), 133.2 (1C, C_{Ar}), 133.1 (1C, C_{Ar}), 133.0 (1C, C_{Ar}), 132.5 (1C, C_{Ar}), 131.6 (1C, C_{Ar}), 131.6 (1C, C_{Ar}), 131.2 (1C, C_{Ar}), 130.7 (1C, C_{Ar}), 130.4 (1C, C_{Ar}), 130.2 (1C, C_{Ar}), 129.7 (1C, C_{Ar}), 129.5 (1C, C_{Ar}), 128.7 (1C, C_{Ar}), 128.4 (1C, C_{Ar}), 128.3 (1C, C_{Ar}), 127.3 (1C, C_{Ar}), 21.6 (1C, CH_2), 21.5 (1C, CH_2), 21.4 (1C, CH_2), 20.4 (1C, CH_2), 20.4 (1C, CH_2), 20.3 (1C, CH_2), 18.5 (2C, CH_3), 18.4 (2C, CH_3), 18.4 (2C, CH_3), 18.2 (2C, CH_3), 18.2 (2C, CH_3), 18.2 (2C, CH_3), 12.1(2C, CH), 12.0(2C, CH), 12.0(2C, CH), 9.7 (1C, CH_2), 9.7 (1C, CH_2), 9.6 (1C, CH_2).

Compound **21**: DEPT-135 (126 MHz, CD_2Cl_2) δ = 129.7 (1C, C_{Ar}), 122.5 (1C, C_{Ar}), 118.4 (1C, C_{Ar}), 116.0 (1C, C_{Ar}), 21.9 (1C, CH_2), 21.2 (1C, CH_2), 18.5 (2C, CH_3), 18.3 (2C, CH_3), 12.3 (2C, CH), 10.2 (1C, CH_2).

Compound **22**: DEPT-135 (126 MHz, CD_2Cl_2) δ = 139.6 (1C, C_{Ar}), 119.9 (1C, C_{Ar}), 117.9 (1C, C_{Ar}), 21.9 (1C, CH_2), 21.3 (1C, CH_2), 18.6 (2C, CH_3), 18.3 (2C, CH_3), 12.3 (2C, CH), 10.1 (1C, CH_2).

Compound **23**: DEPT-135 (126 MHz, CD_2Cl_2) δ = 139.4 (1C, C_{Ar}), 123.5 (1C, C_{Ar}), 117.9 (1C, C_{Ar}), 21.9 (1C, CH_2), 21.2 (1C, CH_2), 18.5 (2C, CH_3), 18.3 (2C, CH_3), 12.3 (2C, CH), 10.1 (1C, CH_2).

Compound **24**: DEPT-135 (126 MHz, CD_2Cl_2) δ = 129.2 (1C, C_{Ar}), 123.7 (1C, C_{Ar}), 114.9 (1C, C_{Ar}), 22.0 (1C, CH_2), 21.3 (1C, CH_2), 18.6 (2C, CH_3), 18.3 (2C, CH_3), 12.3 (2C, CH), 10.1 (1C, CH_2).

Compound **25**: DEPT-135 (126 MHz, CD_2Cl_2) δ = 139.1 (1C, C_{Ar}), 116.1 (1C, C_{Ar}), 22.0 (1C, CH_2), 21.3 (1C, CH_2), 18.7 (2C, CH_3), 18.4 (2C, CH_3), 12.4 (2C, CH), 10.0 (1C, CH_2).

Compound **26**: DEPT-135 (126 MHz, CD_2Cl_2) δ = 132.7 (1C, C_{Ar}), 130.9 (1C, C_{Ar}), 130.1 (1C, C_{Ar}), 130.1 (1C, C_{Ar}), 128.5 (1C, C_{Ar}), 119.7 (1C, C_{Ar}), 116.3 (1C, C_{Ar}), 21.8 (1C, CH_2), 21.2 (1C, CH_2), 18.5 (2C, CH_3), 18.2 (2C, CH_3), 12.2 (2C, CH), 10.0 (1C, CH_2).

Compound **27**: DEPT-135 (126 MHz, CD_2Cl_2) δ = 142.4 (1C, C_{Ar}), 138.5 (1C, C_{Ar}), 132.6 (1C, C_{Ar}), 131.4 (1C, C_{Ar}), 131.3 (1C, C_{Ar}), 130.4 (1C, C_{Ar}), 128.4 (1C, C_{Ar}), 21.7 (1C, CH_2), 21.2 (1C, CH_2), 18.4 (2C, CH_3), 18.2 (2C, CH_3), 12.2 (2C, CH), 9.9 (1C, CH_2).

Compound **28**: DEPT-135 (126 MHz, CD_2Cl_2) δ = 134.7 (1C, C_{Ar}), 132.8 (1C, C_{Ar}), 131.0 (1C, C_{Ar}), 130.8 (1C, C_{Ar}), 130.4 (1C, C_{Ar}), 130.4 (1C, C_{Ar}), 130.2 (1C, C_{Ar}), 129.5 (1C, C_{Ar}), 128.6 (1C, C_{Ar}), 119.1 (1C, C_{Ar}), 116.2 (1C, C_{Ar}), 21.8 (1C, CH_2), 21.2 (1C, CH_2), 18.5 (2C, CH_3), 18.2 (2C, CH_3), 12.2 (2C, CH), 10.0 (1C, CH_2).

Compound **29**: DEPT-135 (126 MHz, CD_2Cl_2) δ = 140.2 (1C, C_{Ar}), 134.8 (1C, C_{Ar}), 132.8 (1C, C_{Ar}), 131.1 (1C, C_{Ar}), 130.7 (1C, C_{Ar}), 130.6 (1C, C_{Ar}), 130.3 (1C, C_{Ar}), 129.8 (1C, C_{Ar}), 129.5 (1C, C_{Ar}), 128.9 (1C, C_{Ar}), 128.6 (1C, C_{Ar}), 21.7 (1C, CH_2), 21.2 (1C, CH_2), 18.5 (2C, CH_3), 18.2 (2C, CH_3), 12.2 (2C, CH), 10.0 (1C, CH_2).

Compound **31**: DEPT-135 (126 MHz, CD_2Cl_2) δ = 133.7 (1C, C_{Ar}), 121.1 (1C, C_{Ar}), 117.3 (1C, C_{Ar}), 32.0 (2C, CH_3).

Compound **32**: DEPT-135 (126 MHz, CD_2Cl_2) δ = 132.5 (1C, C_{Ar}), 121.8 (1C, C_{Ar}), 117.7 (1C, C_{Ar}), 32.0 (2C, CH_3), 21.9 (1C, CH_2), 21.2 (1C, CH_2), 18.5 (2C, CH_3), 18.3 (2C, CH_3), 12.3 (2C, CH), 10.1 (1C, CH_2).

Compound **33**: DEPT-135 (126 MHz, CD_2Cl_2) δ = 142.2 (1C, C_{Ar}), 132.3 (1C, C_{Ar}), 131.8 (1C, C_{Ar}), 31.6 (2C, CH_3), 21.8 (1C, CH_2), 21.3 (1C, CH_2), 18.5 (2C, CH_3), 18.3 (2C, CH_3), 12.2 (2C, CH), 10.1 (1C, CH_2).

Compound **C2**: DEPT-135 (126 MHz, CD_2Cl_2) δ = 135.5 (1C, C_{Ar}), 132.4 (1C, C_{Ar}), 132.3 (1C, C_{Ar}), 129.0 (1C, C_{Ar}), 78.3 (1C, $C_{alkyine}$), 31.8 (2C, CH_3).

Compound **34**: DEPT-135 (126 MHz, C_6D_6) δ = 134.9 (1C, C_{Ar}), 134.9 (1C, C_{Ar}), 133.6 (1C, C_{Ar}), 132.8 (1C, C_{Ar}), 131.7 (1C, C_{Ar}), 131.4 (1C, C_{Ar}), 130.8 (1C, C_{Ar}), 130.4 (1C, C_{Ar}), 130.1 (1C, C_{Ar}), 129.7 (1C, C_{Ar}), 129.5 (1C, C_{Ar}), 129.2 (1C, C_{Ar}), 128.8 (1C, C_{Ar}), 128.3 (1C, C_{Ar}), 127.9 (1C, C_{Ar}), 31.6 (2C, CH_3), 21.4 (1C, CH_2), 20.4 (1C, CH_2), 18.4 (2C, CH_3), 18.1 (2C, CH_3), 12.0 (2C, CH), 9.6 (1C, CH_2).

Compound **35**: DEPT-135 (126 MHz, C_6D_6) δ = 135.3 (1C, C_{Ar}), 135.3 (1C, C_{Ar}), 133.4 (1C, C_{Ar}), 132.7 (1C, C_{Ar}), 131.5 (1C, C_{Ar}), 131.2 (1C, C_{Ar}), 130.8 (1C, C_{Ar}), 130.3 (1C, C_{Ar}), 129.8 (1C, C_{Ar}), 129.7 (1C, C_{Ar}), 129.6 (1C, C_{Ar}), 129.2 (1C, C_{Ar}), 128.7 (1C, C_{Ar}), 128.3 (1C, C_{Ar}), 127.8 (1C, C_{Ar}), 81.7 (1C, $C_{alkyine}$), 31.5 (2C, CH_3). The peak of 81.7 is only visible in the DEPT-135 experiment with $^1J_{CH}$ coupling = 200 Hz.

Compound **36**: DEPT-135 (126 MHz, C_6D_6) δ = 136.0 (1C, C_{Ar}), 135.0 (1C, C_{Ar}), 134.9 (1C, C_{Ar}), 133.4 (1C, C_{Ar}), 132.8 (1C, C_{Ar}), 132.3 (1C, C_{Ar}), 131.7 (1C, C_{Ar}), 131.6 (1C, C_{Ar}), 131.4 (1C, C_{Ar}), 131.0 (1C, C_{Ar}), 130.6 (1C, C_{Ar}), 130.1 (1C, C_{Ar}), 129.8 (1C, C_{Ar}), 129.5 (1C, C_{Ar}), 129.2 (1C, C_{Ar}), 128.9 (1C, C_{Ar}), 128.5 (1C, C_{Ar}), 128.0 (1C, C_{Ar}), 31.5 (2C, CH_3), 31.5 (2C, CH_3), 21.5 (1C, CH_2), 20.4 (1C, CH_2), 18.4 (2C, CH_3), 18.2 (2C, CH_3), 12.0 (2C, CH), 9.7 (1C, CH_2).

Compound **37**: DEPT-135 (126 MHz, C_6D_6) δ = 135.9 (1C, C_{Ar}), 135.0 (1C, C_{Ar}), 134.9 (1C, C_{Ar}), 133.5 (1C, C_{Ar}), 133.3 (1C, C_{Ar}), 133.0 (1C, C_{Ar}), 132.5 (1C, C_{Ar}), 132.4 (1C, C_{Ar}), 131.7 (1C, C_{Ar}), 131.5 (1C, C_{Ar}), 131.5 (1C, C_{Ar}), 131.1 (1C, C_{Ar}), 130.6 (1C, C_{Ar}), 130.5 (1C, C_{Ar}), 129.9 (1C, C_{Ar}), 129.6 (1C, C_{Ar}), 129.2 (1C, C_{Ar}), 128.9 (1C, C_{Ar}), 128.6 (1C, C_{Ar}), 128.6 (1C, C_{Ar}), 128.3 (1C, C_{Ar}), 128.0 (1C, C_{Ar}), 31.5 (2C, CH_3), 31.5 (2C, CH_3), 21.6(1C, CH_2), 21.5(1C, CH_2), 20.4(1C, CH_2), 20.4(1C, CH_2), 18.5 (2C, CH_3), 18.4 (2C, CH_3), 18.2 (2C, CH_3), 18.2 (2C, CH_3), 12.1 (2C, CH), 12.0 (2C, CH), 9.7(1C, CH_2), 9.6(1C, CH_2).

Compound **38**: DEPT-135 (126 MHz, C_6D_6) δ = 135.8 (1C, C_{Ar}), 135.6 (1C, C_{Ar}), 135.0 (1C, C_{Ar}), 133.4 (1C, C_{Ar}), 133.3 (1C, C_{Ar}), 133.0 (1C, C_{Ar}), 132.9 (1C, C_{Ar}), 132.4 (1C, C_{Ar}), 132.1 (1C, C_{Ar}), 131.9 (1C, C_{Ar}), 131.4 (1C, C_{Ar}), 131.1 (1C, C_{Ar}), 130.5 (1C, C_{Ar}), 130.5 (1C, C_{Ar}), 129.8 (1C, C_{Ar}), 129.7 (1C, C_{Ar}), 129.2 (1C, C_{Ar}), 128.8 (1C, C_{Ar}), 128.7 (1C, C_{Ar}), 128.6 (1C, C_{Ar}), 128.3 (1C, C_{Ar}), 127.9 (1C, C_{Ar}), 84.0 (1C, $C_{alkyine}$), 78.6 (1C, $C_{alkyine}$), 21.6 (1C, CH_2), 21.4 (1C, CH_2), 20.4 (1C, CH_2), 20.4 (1C, CH_2), 18.5 (2C, CH_3), 18.4 (2C, CH_3), 18.2 (2C, CH_3), 18.2 (2C, CH_3), 12.1 (2C, CH), 12.0 (2C, CH), 9.7 (1C, CH_2), 9.6 (1C, CH_2). The peaks of 84.0 and 78.6 are only visible in the DEPT-135 experiment with $^1J_{CH}$ coupling = 200 Hz.

Compound **39**: DEPT-135 (126 MHz, C_6D_6) δ = 145.0 (1C, C_{Ar}), 134.7 (1C, C_{Ar}), 133.5 (1C, C_{Ar}), 132.9 (1C, C_{Ar}), 132.5 (1C, C_{Ar}), 132.0 (1C, C_{Ar}), 131.6 (1C, C_{Ar}), 131.6 (1C, C_{Ar}), 131.0 (1C, C_{Ar}), 130.6 (1C, C_{Ar}), 130.4 (1C, C_{Ar}), 129.8 (1C, C_{Ar}), 129.5 (1C, C_{Ar}), 129.2 (1C, C_{Ar}), 129.0 (1C, C_{Ar}), 128.8 (1C, C_{Ar}), 128.6 (1C, C_{Ar}), 128.5 (1C, C_{Ar}), 128.2 (1C, C_{Ar}), 128.1 (1C, C_{Ar}), 128.0 (1C, C_{Ar}), 127.3 (1C, C_{Ar}), 21.6 (1C, CH_2), 21.4 (1C, CH_2), 20.4 (1C, CH_2), 20.4 (1C, CH_2), 18.5 (2C, CH_3), 18.4 (2C, CH_3), 18.2 (2C, CH_3), 18.1 (2C, CH_3), 12.1 (2C, CH), 12.0 (2C, CH), 9.6 (1C, CH_2), 9.6 (1C, CH_2).

Compound **40**: DEPT-135 (126 MHz, C_6D_6) δ = 144.6 (1C, C_{Ar}), 134.2 (1C, C_{Ar}), 133.2 (1C, C_{Ar}), 132.4 (1C, C_{Ar}), 131.9 (1C, C_{Ar}), 131.6 (1C, C_{Ar}), 131.3 (1C, C_{Ar}), 131.1 (1C, C_{Ar}), 130.9 (1C, C_{Ar}), 130.0 (1C, C_{Ar}), 129.9 (1C, C_{Ar}), 129.3 (1C, C_{Ar}), 129.2 (1C, C_{Ar}), 128.8 (1C, C_{Ar}), 128.5 (1C, C_{Ar}), 128.4 (1C, C_{Ar}), 128.2 (1C, C_{Ar}), 128.0 (1C, C_{Ar}), 127.7 (1C, C_{Ar}), 127.6 (1C, C_{Ar}), 127.5 (1C, C_{Ar}), 127.0 (1C, C_{Ar}), 81.6 (1C, $C_{alkyine}$), 80.1 (1C, $C_{alkyine}$). The peaks of 81.6 and 80.1 are only visible in the DEPT-135 experiment with $^1J_{CH}$ coupling = 200 Hz.

Compound **1**: DEPT-135 (126 MHz, C_6D_6) δ = 145.2 (1C, C_{Ar}, C_{23}), 144.5 (1C, C_{Ar}, C_{28}), 134.8 (1C, C_{Ar}, C_{11}), 132.9 (1C, C_{Ar}, C_{33}), 132.0 (1C, C_{Ar}, C_8), 131.5 (1C, C_{Ar}, C_3), 131.5 (1C, C_{Ar}, C_{35}), 131.4 (1C, C_{Ar}, C_{46}), 131.2 (1C, C_{Ar}, C_{37}), 130.3 (1C, C_{Ar}, C_2), 130.2 (1C, C_{Ar}, C_{24}), 130.1 (1C, C_{Ar}, C_5), 130.0 (1C, C_{Ar}, C_{43}), 129.6 (1C, C_{Ar}, C_{45}), 129.6 (1C, C_{Ar}, C_9), 129.4 (1C, C_{Ar}, C_{25}), 129.2 (1C, C_{Ar}, C_{20}), 128.6 (1C, $C_{alkyine}$, C_{44}), 128.5 (1C, $C_{alkyine}$, C_{34}) 128.1 (1C, C_{Ar}, C_{10}), 127.6 (1C, C_{Ar}, C_{21}), 127.0 (1C, C_{Ar}, C_{26}),

6. Summary Structure Calculation

6.1 Calculation with Spartan (’18 V 1.2.0, DFT, B3LYP/6-31G*)

6.1.1 Compound 1a

SPARTAN '18 MECHANICS PROGRAM: (Linux/P4E) build 18.3.0

Frequency Calculation

Adjusted 7 (out of 216) low frequency modes

Reason for exit: Successful completion

Mechanics CPU Time : .10

Mechanics Wall Time: .48

SPARTAN '18 Quantum Mechanics Program: (Linux/P4E) build 18.3.0

Job type: Geometry optimization.

Method: RB3LYP

Basis set: 6-31G(D)

Number of basis functions: 794

Number of electrons: 322

Parallel Job: 12 threads

SCF model:

A restricted hybrid HF-DFT SCF calculation will be

performed using Pulay DIIS + Geometric Direct Minimization

Optimization:

Step	Energy	Max Grad.	Max Dist.
1	-1918.193028	0.046574	0.058936
2	-1918.203646	0.003933	0.060866
3	-1918.204961	0.003031	0.014400
4	-1918.205170	0.003202	0.074847
5	-1918.205752	0.007306	0.070221
6	-1918.206212	0.005248	0.076038
7	-1918.206487	0.003975	0.074191

8	-1918.206688	0.002787	0.070401
9	-1918.206860	0.003275	0.062794
10	-1918.207013	0.003594	0.061588
11	-1918.207150	0.002905	0.052452
12	-1918.207261	0.002253	0.057124
13	-1918.207352	0.002391	0.059940
14	-1918.207433	0.001502	0.062211
15	-1918.207502	0.000962	0.066605
16	-1918.207563	0.001419	0.064346
17	-1918.207609	0.001520	0.068125
18	-1918.207656	0.001474	0.061581
19	-1918.207696	0.001305	0.073145
20	-1918.207740	0.001654	0.062426
21	-1918.207777	0.001745	0.066876
22	-1918.207816	0.001164	0.067897
23	-1918.207848	0.000632	0.068222
24	-1918.207873	0.000535	0.071189
25	-1918.207895	0.000448	0.050168
26	-1918.207905	0.000358	0.056184

<step 2>

Job type: Frequency calculation.

Method: RB3LYP

Basis set: 6-31G(D)

<step 3>

Job type: Single point.

Method: RB3LYP

Basis set: 6-31G(D)

SCF total energy: -1918.2079051 hartrees

Warning: NMR reference data missing

NMR shifts (ppm)

	Atom	Isotropic	Rel. Shift
1	C1	46.7832	
2	C4	53.7183	
3	C2	65.7135	

4	C6	71.8915
5	C5	64.2101
6	C3	65.3404
7	H2	24.9383
8	H5	24.2846
9	H3	24.9699
10	C7	51.5396
11	C8	69.2995
12	C9	73.9322
13	C10	64.0950
14	C11	68.1547
15	C12	60.3778
16	H7	24.9689
17	H8	24.9453
18	H9	24.6809
19	H10	24.9905
20	C13	53.0577
21	C14	62.2933
22	C15	64.8044
23	C16	64.5380
24	C17	71.6096
25	C18	68.8112
26	H1	24.7647
27	H11	24.9123
28	H13	25.0184
29	H14	24.7193
30	C19	97.9470
31	C20	104.3985
32	C21	97.2071
33	C22	98.4791
34	C23	102.0718
35	C24	101.7064
36	C25	63.4270
37	C26	69.3972
38	C27	68.5530
39	C28	63.9575
40	C29	68.4796
41	C30	65.5162
42	H15	24.8881

43	H16	25.0546
44	H17	25.0552
45	H18	25.0992
46	C31	71.1156
47	C32	69.7724
48	C33	66.2395
49	C34	48.1491
50	C35	71.2732
51	C36	67.9885
52	H4	25.1251
53	H19	24.1407
54	H21	25.1018
55	H22	25.3610
56	C37	62.5359
57	C38	69.5228
58	C39	73.4896
59	C40	47.1192
60	C41	72.2190
61	C42	66.7915
62	H23	25.3878
63	H25	25.4170
64	H26	25.5408
65	C43	100.6273
66	C44	111.4780
67	C45	103.8781
68	C46	111.4466
69	C47	106.9359
70	C48	111.0306
71	C49	105.7458
72	C50	113.1910

Reason for exit: Successful completion

Quantum Calculation CPU Time : 41:53:20.66

Quantum Calculation Wall Time: 4:06:41.44

SPARTAN '18 Quantum Mechanics Program: (Linux/P4E) build 18.3.0

Job type: Single point.

Excited States: 6

Method: RB3LYP

Basis set: 6-31G(D)

Number of basis functions: 794

Number of electrons: 322

Parallel Job: 12 threads

SCF model:

A restricted hybrid HF-DFT SCF calculation will be performed using Pulay DIIS + Geometric Direct Minimization

SCF total energy: -1918.2079051 hartrees

Reason for exit: Successful completion

Quantum Calculation CPU Time : 53:26.51

Quantum Calculation Wall Time: 5:09.49

SPARTAN '18 Properties Program: (Linux/P4E) build 18.3.0

Use of molecular symmetry disabled

Cartesian Coordinates (Angstroms)

Atom			X	Y	Z
1	C	C1	0.3754567	-1.5679879	0.1280661
2	C	C4	1.9649148	-1.9002490	2.4656038
3	C	C2	1.1464371	-2.7163364	0.3206472
4	C	C6	0.3930171	-0.5650251	1.1383050
5	C	C5	1.1795791	-0.7510051	2.2933494
6	C	C3	1.9249427	-2.8825229	1.4613486
7	H	H2	1.1260954	-3.4961917	-0.4360895
8	H	H5	1.2111316	0.0411068	3.0343617
9	H	H3	2.5036709	-3.7932444	1.5848967
10	C	C7	2.9130407	-2.0964630	3.6012166
11	C	C8	4.8384734	-2.6042224	5.6157358
12	C	C9	2.5746679	-1.9504734	4.9730257
13	C	C10	4.2247442	-2.4901382	3.2869584

14 C	C11	5.1794838	-2.7400558	4.2690853
15 C	C12	3.5508135	-2.2132317	5.9582193
16 H	H7	4.4999611	-2.5815134	2.2402963
17 H	H8	6.1853702	-3.0341324	3.9825977
18 H	H9	3.2695958	-2.1019798	7.0006972
19 H	H10	5.5714738	-2.7972181	6.3939137
20 C	C13	-0.4328242	-1.4204618	-1.1178740
21 C	C14	-1.9613710	-1.1486785	-3.4618965
22 C	C15	-1.8343276	-1.3884100	-1.0591887
23 C	C16	0.1967206	-1.3223129	-2.3652113
24 C	C17	-0.5557891	-1.1688911	-3.5463955
25 C	C18	-2.5888134	-1.2640648	-2.2249793
26 H	H1	-2.3289474	-1.4647488	-0.0955389
27 H	H11	1.2809457	-1.3306792	-2.4240706
28 H	H13	-3.6736428	-1.2477529	-2.1673149
29 H	H14	-2.5442402	-1.0297565	-4.3696346
30 C	C19	0.0677122	-0.9753220	-4.8189700
31 C	C20	0.5008162	-0.7377141	-5.9307209
32 C	C21	-0.3162653	0.6534526	0.8901372
33 C	C22	-0.9226292	1.6306461	0.4880913
34 C	C23	1.2859142	-1.5451621	5.4287125
35 C	C24	0.2514211	-1.1738806	5.9510979
36 C	C25	0.9584207	-0.3792794	-7.2332041
37 C	C26	1.7970858	0.3295336	-9.8289230
38 C	C27	0.7411316	0.9455110	-7.7271719
39 C	C28	1.6042470	-1.3115378	-8.0614655
40 C	C29	2.0198044	-0.9624270	-9.3444897
41 C	C30	1.1649079	1.2744737	-9.0268821
42 H	H15	1.7694120	-2.3159348	-7.6844448
43 H	H16	2.5153273	-1.7008844	-9.9684362
44 H	H17	0.9932958	2.2820278	-9.3924726
45 H	H18	2.1187533	0.6004032	-10.8305160
46 C	C31	-0.9536149	-0.5672974	6.4138110
47 C	C32	-3.2822692	0.7998842	7.2356649
48 C	C33	-1.5350234	-0.8348251	7.6695561
49 C	C34	-1.5602306	0.3794683	5.5808134
50 C	C35	-2.7159047	1.0751328	5.9792421
51 C	C36	-2.6881957	-0.1548165	8.0627487
52 H	H4	-1.0778185	-1.5680174	8.3264816

53 H	H19	-1.1269911	0.5931165	4.6106342
54 H	H21	-3.1287036	-0.3687961	9.0326222
55 H	H22	-4.1744694	1.3298042	7.5539469
56 C	C37	-1.5157799	2.6870396	-0.2686094
57 C	C38	-2.6060597	4.6803640	-1.9529498
58 C	C39	-2.4472130	3.6381003	0.2500076
59 C	C40	-1.1804490	2.7475117	-1.6244458
60 C	C41	-1.7234601	3.7191542	-2.4846174
61 C	C42	-2.9574239	4.6314985	-0.6105119
62 H	H23	-0.4985830	2.0087412	-2.0285068
63 H	H25	-3.6570073	5.3565819	-0.2069078
64 H	H26	-3.0259008	5.4430860	-2.6008649
65 C	C43	-3.1967500	2.0195956	5.0271908
66 C	C44	-3.2425799	2.6495822	3.9794058
67 C	C45	-2.8630190	3.5117748	1.5944508
68 C	C46	-3.1362464	3.2012793	2.7455089
69 C	C47	-1.3606728	3.6107694	-3.8546017
70 C	C48	-0.9166726	3.1921497	-4.9150604
71 C	C49	0.1229308	1.8970210	-6.8775483
72 C	C50	-0.3907817	2.5899794	-6.0114602

Point Group = C1 Order = 1 Nsymop = 1

Closed-Shell Molecular Orbital Coefficients

MO:	1	2	3	4	5
Eigenvalues:	-10.23070	-10.22954	-10.22839	-10.22797	-10.22739
(ev)	-278.39173	-278.35997	-278.32885	-278.31724	-278.30143

		A	A	A	A	A
1 C1	S1	-0.00000	-0.00000	0.00000	-0.00000	0.00000
2 C1	S	0.00001	0.00002	0.00001	0.00001	0.00002
3 C1	PX	0.00001	0.00001	-0.00001	-0.00000	0.00000
4 C1	PY	-0.00000	-0.00001	0.00000	-0.00000	-0.00000
5 C1	PZ	0.00001	0.00001	-0.00000	-0.00000	0.00000
6 C1	S'	-0.00021	-0.00048	-0.00019	-0.00011	-0.00026
7 C1	PX'	-0.00023	-0.00029	0.00028	0.00014	-0.00012
8 C1	PY'	0.00000	0.00001	-0.00006	-0.00001	-0.00012
9 C1	PZ'	-0.00034	-0.00046	0.00038	0.00021	-0.00027
10 C1	DXX	0.00000	-0.00000	-0.00000	-0.00000	0.00000

11	C1	DYY	-0.00000	-0.00001	0.00000	0.00000	-0.00000
12	C1	DZZ	-0.00000	0.00000	-0.00000	-0.00000	-0.00000
13	C1	DXY	0.00000	0.00001	0.00000	0.00000	0.00000
14	C1	DXZ	-0.00000	-0.00000	-0.00001	-0.00000	-0.00000
15	C1	DYZ	-0.00000	-0.00000	0.00000	0.00000	-0.00000
16	C4	S1	0.00000	0.00000	0.00000	0.00000	0.00000
17	C4	S	0.00001	0.00001	0.00000	0.00000	0.00001
18	C4	PX	-0.00001	-0.00001	0.00000	0.00000	-0.00001
19	C4	PY	0.00000	0.00000	-0.00000	-0.00000	0.00000
20	C4	PZ	-0.00001	-0.00001	-0.00000	-0.00000	-0.00001
21	C4	S'	0.00009	0.00008	-0.00007	-0.00003	0.00000
22	C4	PX'	0.00021	0.00026	0.00001	0.00002	0.00019
23	C4	PY'	-0.00009	-0.00014	-0.00000	-0.00001	-0.00008
24	C4	PZ'	0.00022	0.00024	0.00002	0.00002	0.00020
25	C4	DXX	0.00000	0.00000	0.00000	0.00000	0.00000
26	C4	DYY	-0.00000	-0.00001	0.00000	-0.00000	-0.00000
27	C4	DZZ	0.00000	0.00001	0.00000	0.00000	0.00000
28	C4	DXY	0.00001	0.00001	-0.00000	0.00000	0.00001
29	C4	DXZ	0.00000	0.00000	0.00000	0.00000	0.00000
30	C4	DYZ	-0.00000	0.00000	-0.00000	-0.00000	-0.00000
31	C2	S1	0.00000	-0.00000	0.00000	0.00000	0.00000
32	C2	S	0.00000	-0.00001	0.00001	0.00000	0.00000
33	C2	PX	-0.00000	-0.00001	0.00000	0.00000	-0.00000
34	C2	PY	0.00001	0.00001	-0.00000	-0.00000	0.00000
35	C2	PZ	-0.00000	-0.00000	-0.00000	-0.00000	-0.00000
36	C2	S'	0.00010	0.00024	-0.00017	-0.00006	-0.00000
37	C2	PX'	-0.00000	-0.00001	-0.00001	-0.00001	0.00002
38	C2	PY'	0.00006	0.00012	-0.00009	-0.00005	0.00002
39	C2	PZ'	0.00004	0.00010	-0.00009	-0.00005	0.00003
40	C2	DXX	0.00000	0.00000	0.00000	0.00000	0.00000
41	C2	DYY	0.00000	0.00000	-0.00000	-0.00000	0.00000
42	C2	DZZ	-0.00000	-0.00000	0.00000	0.00000	-0.00000
43	C2	DXY	-0.00000	-0.00000	0.00000	-0.00000	0.00000
44	C2	DXZ	-0.00000	-0.00000	0.00000	0.00000	-0.00000
45	C2	DYZ	-0.00000	-0.00000	-0.00000	-0.00000	-0.00000
46	C6	S1	-0.00001	-0.00006	0.00000	-0.00000	-0.00004
47	C6	S	0.00003	0.00005	-0.00000	0.00000	0.00001
48	C6	PX	0.00000	0.00002	0.00000	0.00000	0.00001
49	C6	PY	-0.00001	-0.00003	-0.00000	-0.00000	-0.00001

50 C6	PZ	0.00001	0.00003	-0.00000	-0.00000	0.00002
51 C6	S'	-0.00149	-0.00124	0.00016	-0.00005	-0.00121
52 C6	PX'	0.00053	0.00045	-0.00021	-0.00008	0.00034
53 C6	PY'	-0.00101	-0.00111	0.00033	0.00013	-0.00081
54 C6	PZ'	-0.00012	-0.00033	-0.00004	-0.00001	-0.00025
55 C6	DXX	-0.00001	-0.00003	0.00000	0.00000	-0.00001
56 C6	DYY	-0.00001	-0.00007	0.00000	-0.00000	-0.00001
57 C6	DZZ	-0.00000	-0.00001	0.00000	-0.00000	-0.00000
58 C6	DXY	0.00001	0.00005	-0.00000	0.00000	0.00001
59 C6	DXZ	-0.00000	-0.00001	0.00000	0.00000	-0.00000
60 C6	DYZ	-0.00000	0.00002	-0.00000	-0.00000	0.00000
61 C5	S1	0.00000	0.00000	-0.00000	-0.00000	-0.00000
62 C5	S	0.00000	-0.00001	-0.00000	-0.00000	-0.00000
63 C5	PX	-0.00001	-0.00001	-0.00000	-0.00000	-0.00001
64 C5	PY	0.00001	0.00001	-0.00000	-0.00000	0.00001
65 C5	PZ	-0.00000	-0.00001	-0.00000	-0.00000	-0.00001
66 C5	S'	0.00015	0.00036	0.00011	0.00007	0.00027
67 C5	PX'	-0.00001	-0.00004	-0.00000	0.00000	-0.00004
68 C5	PY'	-0.00001	-0.00001	-0.00005	-0.00004	-0.00003
69 C5	PZ'	0.00000	-0.00005	-0.00006	-0.00003	-0.00005
70 C5	DXX	0.00001	0.00001	-0.00000	-0.00000	0.00001
71 C5	DYY	0.00000	0.00001	-0.00000	-0.00000	0.00000
72 C5	DZZ	-0.00000	0.00000	0.00000	0.00000	-0.00000
73 C5	DXY	-0.00000	-0.00001	0.00000	0.00000	-0.00000
74 C5	DXZ	0.00000	0.00001	0.00000	0.00000	0.00000
75 C5	DYZ	-0.00001	-0.00001	0.00000	0.00000	-0.00001
76 C3	S1	0.00000	0.00000	-0.00000	0.00000	-0.00000
77 C3	S	0.00000	0.00001	-0.00000	0.00000	0.00000
78 C3	PX	-0.00000	-0.00000	-0.00000	-0.00000	-0.00000
79 C3	PY	0.00001	0.00001	0.00000	0.00000	0.00000
80 C3	PZ	0.00000	0.00001	-0.00000	-0.00000	0.00000
81 C3	S'	0.00001	-0.00007	0.00009	0.00004	0.00004
82 C3	PX'	-0.00003	-0.00001	-0.00002	-0.00001	-0.00003
83 C3	PY'	0.00003	0.00000	0.00003	0.00001	0.00004
84 C3	PZ'	-0.00002	-0.00001	-0.00001	-0.00001	-0.00001
85 C3	DXX	0.00000	0.00000	0.00000	0.00000	0.00000
86 C3	DYY	0.00000	0.00000	-0.00000	0.00000	0.00000
87 C3	DZZ	-0.00000	-0.00000	0.00000	0.00000	-0.00000
88 C3	DXY	-0.00000	-0.00000	0.00000	0.00000	-0.00000

89	C3	DXZ	-0.00000	-0.00001	0.00000	-0.00000	-0.00000
90	C3	DYZ	0.00000	0.00000	0.00000	-0.00000	0.00000
91	H2	S	0.00000	0.00001	-0.00000	-0.00000	0.00000
92	H2	S'	0.00002	0.00004	-0.00004	-0.00003	0.00001
93	H5	S	0.00000	0.00000	0.00000	0.00000	0.00000
94	H5	S'	-0.00002	-0.00004	0.00002	0.00001	-0.00001
95	H3	S	0.00000	0.00000	0.00000	0.00000	0.00000
96	H3	S'	0.00002	0.00001	0.00000	0.00000	0.00002
97	C7	S1	0.00000	0.00000	-0.00000	0.00000	0.00000
98	C7	S	0.00001	0.00001	-0.00000	0.00000	0.00001
99	C7	PX	-0.00000	-0.00000	0.00000	-0.00000	-0.00000
100	C7	PY	-0.00000	-0.00000	-0.00000	-0.00000	-0.00000
101	C7	PZ	-0.00000	-0.00000	0.00000	-0.00000	-0.00000
102	C7	S'	-0.00013	-0.00015	-0.00000	-0.00001	-0.00012
103	C7	PX'	0.00019	0.00017	0.00000	0.00001	0.00013
104	C7	PY'	-0.00003	-0.00001	-0.00000	-0.00000	-0.00002
105	C7	PZ'	0.00020	0.00024	-0.00001	0.00001	0.00015
106	C7	DXX	-0.00000	-0.00000	-0.00000	-0.00000	-0.00000
107	C7	DYY	0.00000	0.00000	-0.00000	0.00000	0.00000
108	C7	DZZ	-0.00000	-0.00000	-0.00000	-0.00000	-0.00000
109	C7	DXY	-0.00000	-0.00000	0.00000	-0.00000	-0.00000
110	C7	DXZ	-0.00000	-0.00000	0.00000	-0.00000	-0.00000
111	C7	DYZ	-0.00000	-0.00000	0.00000	-0.00000	-0.00000
112	C8	S1	0.00000	0.00000	-0.00000	0.00000	0.00000
113	C8	S	0.00000	0.00000	0.00000	0.00000	0.00000
114	C8	PX	0.00000	0.00000	-0.00000	0.00000	0.00000
115	C8	PY	-0.00000	0.00000	0.00000	0.00000	-0.00000
116	C8	PZ	-0.00000	-0.00000	0.00000	-0.00000	-0.00000
117	C8	S'	-0.00005	-0.00004	0.00000	-0.00000	-0.00003
118	C8	PX'	0.00001	0.00001	-0.00000	0.00000	0.00001
119	C8	PY'	-0.00000	-0.00001	0.00000	-0.00000	-0.00000
120	C8	PZ'	0.00001	0.00001	0.00000	0.00000	0.00001
121	C8	DXX	0.00000	0.00000	0.00000	0.00000	0.00000
122	C8	DYY	-0.00000	0.00000	-0.00000	0.00000	0.00000
123	C8	DZZ	0.00000	0.00000	-0.00000	-0.00000	0.00000
124	C8	DXY	0.00000	0.00000	-0.00000	0.00000	0.00000
125	C8	DXZ	0.00000	0.00000	0.00000	0.00000	0.00000
126	C8	DYZ	-0.00000	-0.00000	0.00000	-0.00000	-0.00000
127	C9	S1	-0.00000	-0.00000	-0.00000	-0.00000	-0.00000

128	C9	S	-0.00001	-0.00000	-0.00000	-0.00000	-0.00000
129	C9	PX	0.00000	0.00000	-0.00000	0.00000	0.00000
130	C9	PY	0.00000	-0.00000	-0.00000	0.00000	-0.00000
131	C9	PZ	0.00000	0.00000	0.00000	0.00000	0.00000
132	C9	S'	0.00020	0.00001	0.00001	0.00000	0.00006
133	C9	PX'	-0.00029	-0.00021	0.00000	-0.00001	-0.00017
134	C9	PY'	0.00008	0.00005	-0.00000	0.00000	0.00004
135	C9	PZ'	0.00006	0.00006	-0.00000	0.00000	0.00002
136	C9	DXX	0.00000	0.00000	-0.00000	0.00000	0.00000
137	C9	DYY	-0.00000	-0.00000	-0.00000	-0.00000	-0.00000
138	C9	DZZ	0.00000	-0.00000	0.00000	-0.00000	-0.00000
139	C9	DXY	-0.00000	-0.00000	0.00000	-0.00000	-0.00000
140	C9	DXZ	-0.00000	0.00000	-0.00000	-0.00000	-0.00000
141	C9	DYZ	0.00000	0.00000	0.00000	0.00000	0.00000
142	C10	S1	0.00000	0.00000	-0.00000	0.00000	0.00000
143	C10	S	0.00000	0.00000	-0.00000	0.00000	0.00000
144	C10	PX	0.00000	-0.00000	0.00000	0.00000	0.00000
145	C10	PY	-0.00000	-0.00000	0.00000	-0.00000	-0.00000
146	C10	PZ	-0.00000	-0.00000	0.00000	-0.00000	-0.00000
147	C10	S'	-0.00008	-0.00006	-0.00000	-0.00000	-0.00005
148	C10	PX'	0.00002	0.00001	-0.00000	0.00000	0.00001
149	C10	PY'	-0.00001	-0.00001	-0.00000	-0.00000	-0.00001
150	C10	PZ'	-0.00005	-0.00005	-0.00000	-0.00000	-0.00003
151	C10	DXX	0.00000	0.00000	-0.00000	-0.00000	0.00000
152	C10	DYY	0.00000	0.00000	-0.00000	0.00000	0.00000
153	C10	DZZ	-0.00000	-0.00000	-0.00000	-0.00000	-0.00000
154	C10	DXY	0.00000	0.00000	0.00000	0.00000	0.00000
155	C10	DXZ	0.00000	0.00000	-0.00000	0.00000	0.00000
156	C10	DYZ	-0.00000	-0.00000	0.00000	-0.00000	-0.00000
157	C11	S1	-0.00000	0.00000	-0.00000	-0.00000	0.00000
158	C11	S	-0.00000	0.00000	-0.00000	-0.00000	0.00000
159	C11	PX	-0.00000	-0.00000	-0.00000	-0.00000	-0.00000
160	C11	PY	0.00000	0.00000	-0.00000	0.00000	0.00000
161	C11	PZ	-0.00000	-0.00000	-0.00000	-0.00000	-0.00000
162	C11	S'	0.00004	0.00004	0.00000	0.00000	0.00003
163	C11	PX'	-0.00002	-0.00001	-0.00000	-0.00000	-0.00001
164	C11	PY'	0.00001	0.00000	0.00000	0.00000	0.00000
165	C11	PZ'	0.00001	0.00000	0.00000	0.00000	0.00000
166	C11	DXX	-0.00000	-0.00000	0.00000	-0.00000	-0.00000

167	C11	DYY	-0.00000	0.00000	-0.00000	0.00000	0.00000
168	C11	DZZ	0.00000	0.00000	0.00000	0.00000	0.00000
169	C11	DXY	0.00000	0.00000	-0.00000	-0.00000	0.00000
170	C11	DXZ	-0.00000	-0.00000	0.00000	0.00000	-0.00000
171	C11	DYZ	-0.00000	-0.00000	-0.00000	-0.00000	-0.00000
172	C12	S1	-0.00000	-0.00000	-0.00000	-0.00000	-0.00000
173	C12	S	-0.00000	-0.00000	-0.00000	-0.00000	-0.00000
174	C12	PX	-0.00000	-0.00000	0.00000	-0.00000	-0.00000
175	C12	PY	0.00000	0.00000	-0.00000	0.00000	0.00000
176	C12	PZ	-0.00000	-0.00000	0.00000	-0.00000	-0.00000
177	C12	S'	0.00010	0.00008	0.00000	0.00001	0.00007
178	C12	PX'	0.00001	0.00001	-0.00000	-0.00000	0.00000
179	C12	PY'	-0.00000	-0.00000	0.00000	0.00000	-0.00000
180	C12	PZ'	-0.00006	-0.00005	0.00000	-0.00000	-0.00004
181	C12	DXX	-0.00000	-0.00000	0.00000	-0.00000	-0.00000
182	C12	DYY	-0.00000	-0.00000	-0.00000	-0.00000	-0.00000
183	C12	DZZ	0.00000	0.00000	-0.00000	0.00000	0.00000
184	C12	DXY	-0.00000	-0.00000	-0.00000	-0.00000	-0.00000
185	C12	DXZ	-0.00000	0.00000	0.00000	0.00000	0.00000
186	C12	DYZ	-0.00000	-0.00000	-0.00000	-0.00000	-0.00000
187	H7	S	-0.00000	-0.00000	-0.00000	-0.00000	-0.00000
188	H7	S'	-0.00001	-0.00002	0.00000	-0.00000	-0.00001
189	H8	S	0.00000	-0.00000	0.00000	0.00000	0.00000
190	H8	S'	0.00000	-0.00000	0.00000	0.00000	0.00000
191	H9	S	0.00000	0.00000	-0.00000	0.00000	0.00000
192	H9	S'	0.00002	0.00001	-0.00000	0.00000	0.00001
193	H10	S	-0.00000	-0.00000	-0.00000	-0.00000	-0.00000
194	H10	S'	-0.00000	-0.00000	-0.00000	-0.00000	-0.00000
195	C13	S1	0.00000	0.00001	0.00001	0.00000	-0.00001
196	C13	S	0.00001	0.00002	0.00002	0.00001	0.00000
197	C13	PX	-0.00000	-0.00000	-0.00001	-0.00001	0.00000
198	C13	PY	-0.00000	0.00001	0.00000	0.00000	-0.00001
199	C13	PZ	0.00000	-0.00001	-0.00002	-0.00001	0.00000
200	C13	S'	-0.00023	-0.00031	0.00009	0.00006	-0.00006
201	C13	PX'	-0.00008	-0.00005	0.00027	0.00015	-0.00006
202	C13	PY'	0.00002	-0.00001	-0.00005	-0.00003	0.00006
203	C13	PZ'	-0.00016	-0.00013	0.00050	0.00028	-0.00010
204	C13	DXX	-0.00000	0.00000	0.00000	0.00000	-0.00000
205	C13	DYY	0.00000	0.00001	0.00001	0.00001	-0.00000

206	C13	DZZ	-0.00000	-0.00000	-0.00000	-0.00000	-0.00000
207	C13	DXY	0.00000	0.00000	-0.00000	0.00000	0.00000
208	C13	DXZ	-0.00000	-0.00000	0.00001	0.00001	-0.00000
209	C13	DYZ	0.00000	0.00000	0.00000	-0.00000	0.00000
210	C14	S1	-0.00000	0.00000	0.00000	0.00000	0.00000
211	C14	S	-0.00000	0.00000	0.00003	0.00002	0.00000
212	C14	PX	0.00000	-0.00000	-0.00000	-0.00000	0.00000
213	C14	PY	0.00000	0.00000	-0.00000	-0.00000	0.00000
214	C14	PZ	0.00000	0.00000	0.00000	0.00001	0.00000
215	C14	S'	0.00003	0.00001	-0.00034	-0.00030	-0.00001
216	C14	PX'	0.00001	0.00000	-0.00014	-0.00011	-0.00001
217	C14	PY'	-0.00001	-0.00001	0.00002	-0.00001	0.00000
218	C14	PZ'	0.00002	-0.00000	-0.00018	-0.00016	-0.00002
219	C14	DXX	0.00000	0.00000	0.00000	0.00000	0.00000
220	C14	DYY	0.00000	0.00000	0.00001	0.00001	0.00000
221	C14	DZZ	0.00000	0.00000	-0.00001	-0.00001	0.00000
222	C14	DXY	-0.00000	-0.00000	-0.00000	-0.00001	0.00000
223	C14	DXZ	0.00000	0.00000	-0.00001	0.00000	-0.00000
224	C14	DYZ	-0.00000	-0.00000	0.00000	-0.00000	0.00000
225	C15	S1	-0.00000	-0.00001	0.00000	0.00000	0.00000
226	C15	S	-0.00000	-0.00001	0.00000	0.00000	0.00000
227	C15	PX	0.00000	0.00001	0.00001	0.00001	-0.00000
228	C15	PY	-0.00000	-0.00002	0.00000	0.00000	0.00000
229	C15	PZ	0.00000	0.00000	-0.00001	-0.00000	0.00000
230	C15	S'	0.00002	0.00011	0.00018	0.00008	-0.00004
231	C15	PX'	-0.00000	0.00001	0.00005	0.00002	-0.00002
232	C15	PY'	0.00000	0.00004	0.00001	0.00000	-0.00002
233	C15	PZ'	0.00001	-0.00002	-0.00012	-0.00006	0.00002
234	C15	DXX	0.00000	-0.00000	0.00000	0.00000	0.00000
235	C15	DYY	-0.00000	-0.00001	0.00000	0.00000	0.00000
236	C15	DZZ	-0.00000	-0.00000	0.00000	0.00000	0.00000
237	C15	DXY	-0.00000	0.00000	0.00000	0.00000	-0.00000
238	C15	DXZ	0.00000	0.00000	-0.00001	-0.00000	0.00000
239	C15	DYZ	0.00000	-0.00000	0.00000	0.00000	0.00000
240	C16	S1	-0.00000	0.00001	0.00000	0.00000	0.00000
241	C16	S	-0.00000	0.00001	-0.00000	-0.00000	0.00001
242	C16	PX	-0.00000	-0.00000	0.00000	0.00000	0.00000
243	C16	PY	-0.00000	0.00000	0.00000	0.00000	0.00000
244	C16	PZ	0.00000	0.00000	-0.00002	-0.00001	-0.00000

245	C16	S'	-0.00001	-0.00003	0.00028	0.00018	-0.00006
246	C16	PX'	0.00001	0.00001	-0.00005	-0.00002	0.00004
247	C16	PY'	0.00001	-0.00001	0.00000	-0.00002	-0.00002
248	C16	PZ'	0.00001	-0.00000	-0.00005	-0.00005	-0.00001
249	C16	DXX	-0.00000	0.00000	-0.00001	-0.00000	-0.00000
250	C16	DYY	-0.00000	0.00001	0.00001	0.00000	0.00001
251	C16	DZZ	-0.00000	0.00001	0.00002	0.00001	0.00000
252	C16	DXY	0.00000	0.00000	0.00000	0.00000	0.00000
253	C16	DXZ	0.00000	-0.00000	-0.00000	0.00000	0.00000
254	C16	DYZ	0.00000	0.00000	-0.00000	0.00000	-0.00000
255	C17	S1	-0.00001	0.00000	-0.00003	-0.00003	0.00001
256	C17	S	-0.00001	0.00000	0.00008	0.00005	0.00001
257	C17	PX	-0.00000	-0.00000	-0.00001	-0.00001	0.00000
258	C17	PY	-0.00000	0.00000	-0.00001	-0.00002	0.00000
259	C17	PZ	0.00000	0.00000	0.00004	0.00004	0.00001
260	C17	S'	0.00013	-0.00002	-0.00256	-0.00130	-0.00032
261	C17	PX'	0.00006	0.00000	-0.00101	-0.00065	-0.00009
262	C17	PY'	0.00002	0.00000	-0.00014	0.00004	-0.00003
263	C17	PZ'	-0.00010	-0.00001	0.00130	0.00073	0.00014
264	C17	DXX	-0.00000	0.00000	-0.00002	-0.00002	0.00000
265	C17	DYY	-0.00000	0.00000	0.00001	0.00000	0.00001
266	C17	DZZ	0.00000	0.00000	-0.00007	-0.00008	0.00000
267	C17	DXY	-0.00000	-0.00000	-0.00000	0.00000	0.00000
268	C17	DXZ	-0.00000	-0.00000	0.00003	0.00004	0.00000
269	C17	DYZ	-0.00000	0.00000	0.00001	-0.00000	0.00000
270	C18	S1	0.00000	-0.00000	0.00000	0.00000	0.00000
271	C18	S	0.00000	0.00000	0.00002	0.00001	0.00000
272	C18	PX	0.00000	0.00000	0.00001	0.00000	0.00000
273	C18	PY	-0.00000	-0.00001	0.00000	0.00000	0.00000
274	C18	PZ	-0.00000	-0.00001	-0.00001	-0.00001	0.00000
275	C18	S'	-0.00002	-0.00004	-0.00001	0.00006	0.00000
276	C18	PX'	-0.00002	-0.00003	-0.00006	-0.00000	-0.00001
277	C18	PY'	0.00000	0.00000	0.00001	0.00001	0.00000
278	C18	PZ'	0.00000	-0.00001	-0.00004	-0.00004	0.00000
279	C18	DXX	-0.00000	-0.00000	-0.00000	-0.00000	0.00000
280	C18	DYY	0.00000	-0.00000	0.00001	0.00000	0.00000
281	C18	DZZ	-0.00000	-0.00000	0.00000	0.00000	0.00000
282	C18	DXY	0.00000	-0.00000	0.00000	0.00000	0.00000
283	C18	DXZ	-0.00000	0.00000	-0.00000	-0.00000	-0.00000

284	C18	DYZ	-0.00000	-0.00000	0.00000	-0.00000	0.00000
285	H1	S	-0.00000	-0.00001	0.00000	0.00000	0.00000
286	H1	S'	-0.00001	-0.00000	0.00004	0.00002	-0.00001
287	H11	S	-0.00000	0.00000	-0.00000	-0.00000	0.00000
288	H11	S'	-0.00000	0.00000	-0.00002	-0.00002	-0.00001
289	H13	S	-0.00000	-0.00000	-0.00000	-0.00000	-0.00000
290	H13	S'	-0.00001	-0.00001	-0.00004	-0.00001	-0.00001
291	H14	S	0.00000	-0.00000	-0.00001	-0.00001	-0.00000
292	H14	S'	0.00001	-0.00000	-0.00007	-0.00006	-0.00001
293	C19	S1	-0.00000	0.00000	0.00011	0.00013	0.00000
294	C19	S	0.00000	0.00000	0.00018	0.00019	0.00001
295	C19	PX	-0.00000	-0.00000	0.00006	0.00007	0.00000
296	C19	PY	-0.00000	-0.00000	0.00003	0.00004	-0.00000
297	C19	PZ	0.00000	0.00000	-0.00014	-0.00018	0.00000
298	C19	S'	0.00003	-0.00014	-0.00289	-0.00048	-0.00088
299	C19	PX'	0.00007	-0.00002	-0.00178	-0.00073	-0.00029
300	C19	PY'	0.00003	-0.00001	-0.00083	-0.00067	-0.00013
301	C19	PZ'	-0.00018	0.00006	0.00449	0.00208	0.00069
302	C19	DXX	-0.00000	0.00000	0.00004	0.00004	0.00000
303	C19	DYY	-0.00000	0.00000	0.00002	0.00001	0.00000
304	C19	DZZ	-0.00000	-0.00000	0.00008	0.00016	-0.00000
305	C19	DXY	0.00000	-0.00000	0.00000	0.00000	-0.00000
306	C19	DXZ	0.00000	0.00000	-0.00004	-0.00009	0.00000
307	C19	DYZ	-0.00000	0.00000	-0.00001	-0.00000	0.00000
308	C20	S1	0.00000	-0.00000	0.00364	0.00386	-0.00000
309	C20	S	0.00000	0.00000	-0.00013	-0.00000	0.00000
310	C20	PX	0.00000	0.00000	-0.00007	-0.00007	0.00000
311	C20	PY	-0.00000	0.00000	-0.00007	-0.00008	0.00000
312	C20	PZ	-0.00000	-0.00000	0.00022	0.00020	-0.00001
313	C20	S'	-0.00018	0.00001	0.00774	0.00578	0.00033
314	C20	PX'	0.00003	-0.00005	-0.00065	0.00016	-0.00039
315	C20	PY'	0.00002	-0.00003	-0.00047	0.00093	-0.00023
316	C20	PZ'	-0.00009	0.00014	0.00160	-0.00109	0.00104
317	C20	DXX	-0.00000	-0.00000	-0.00017	-0.00020	-0.00000
318	C20	DYY	0.00000	-0.00000	-0.00020	-0.00029	-0.00000
319	C20	DZZ	-0.00000	-0.00000	-0.00042	-0.00061	-0.00000
320	C20	DXY	0.00000	0.00000	-0.00001	0.00001	0.00000
321	C20	DXZ	0.00000	-0.00000	0.00012	0.00021	-0.00000
322	C20	DYZ	-0.00000	-0.00000	0.00008	0.00012	-0.00000

323	C21	S1	0.00002	0.00017	-0.00000	0.00000	0.00001
324	C21	S	0.00005	0.00021	-0.00000	0.00000	0.00006
325	C21	PX	0.00000	-0.00013	-0.00000	-0.00000	-0.00000
326	C21	PY	0.00000	0.00019	-0.00000	-0.00000	0.00001
327	C21	PZ	0.00001	-0.00006	0.00000	0.00000	-0.00000
328	C21	S'	-0.00370	0.00012	0.00023	-0.00020	-0.00398
329	C21	PX'	0.00182	0.00113	-0.00028	-0.00001	0.00169
330	C21	PY'	-0.00310	-0.00177	0.00048	0.00002	-0.00277
331	C21	PZ'	0.00122	0.00096	-0.00016	-0.00000	0.00114
332	C21	DXX	0.00001	0.00008	-0.00000	0.00000	0.00001
333	C21	DYY	-0.00001	0.00017	-0.00000	-0.00000	-0.00000
334	C21	DZZ	-0.00001	0.00001	0.00000	-0.00000	-0.00000
335	C21	DXY	0.00001	-0.00012	0.00000	0.00000	0.00000
336	C21	DXZ	-0.00001	0.00001	0.00000	-0.00000	-0.00001
337	C21	DYZ	0.00002	-0.00002	-0.00000	0.00000	0.00001
338	C22	S1	0.00097	0.00537	0.00000	-0.00000	-0.00006
339	C22	S	-0.00010	-0.00006	0.00001	-0.00000	-0.00009
340	C22	PX	0.00001	0.00013	0.00000	-0.00000	-0.00002
341	C22	PY	-0.00004	-0.00025	-0.00000	0.00000	0.00002
342	C22	PZ	0.00002	0.00022	-0.00000	0.00000	0.00001
343	C22	S'	0.00391	0.00710	-0.00042	-0.00005	0.00159
344	C22	PX'	0.00224	-0.00111	-0.00024	0.00007	0.00255
345	C22	PY'	-0.00326	0.00180	0.00036	-0.00012	-0.00398
346	C22	PZ'	0.00112	-0.00148	-0.00016	0.00005	0.00158
347	C22	DXX	-0.00004	-0.00032	0.00000	-0.00000	-0.00005
348	C22	DYY	-0.00004	-0.00056	0.00000	-0.00000	-0.00005
349	C22	DZZ	-0.00002	-0.00047	0.00000	-0.00000	-0.00000
350	C22	DXY	-0.00002	0.00026	0.00000	-0.00000	-0.00000
351	C22	DXZ	-0.00002	-0.00009	-0.00000	-0.00000	-0.00001
352	C22	DYZ	0.00001	0.00026	0.00000	0.00000	-0.00001
353	C23	S1	-0.00000	-0.00000	-0.00000	0.00000	-0.00000
354	C23	S	-0.00000	0.00000	-0.00000	0.00000	0.00000
355	C23	PX	0.00000	0.00000	0.00000	0.00000	0.00000
356	C23	PY	-0.00000	-0.00000	-0.00000	-0.00000	-0.00000
357	C23	PZ	-0.00000	-0.00000	0.00000	-0.00000	-0.00000
358	C23	S'	0.00030	-0.00010	0.00001	-0.00001	0.00007
359	C23	PX'	-0.00048	-0.00015	-0.00001	-0.00001	-0.00020
360	C23	PY'	0.00017	0.00006	0.00000	0.00000	0.00007
361	C23	PZ'	0.00021	0.00006	0.00000	0.00000	0.00010

362	C23	DXX	-0.00000	-0.00000	0.00000	-0.00000	-0.00000
363	C23	DYY	0.00000	-0.00000	-0.00000	-0.00000	-0.00000
364	C23	DZZ	0.00000	-0.00000	-0.00000	0.00000	0.00000
365	C23	DXY	-0.00000	0.00000	-0.00000	0.00000	0.00000
366	C23	DXZ	-0.00000	0.00000	0.00000	0.00000	0.00000
367	C23	DYZ	0.00000	-0.00000	-0.00000	-0.00000	-0.00000
368	C24	S1	0.00000	0.00000	0.00000	0.00000	0.00000
369	C24	S	0.00001	0.00000	0.00000	0.00000	0.00000
370	C24	PX	0.00000	-0.00000	0.00000	-0.00000	0.00000
371	C24	PY	0.00000	0.00000	0.00000	0.00000	-0.00000
372	C24	PZ	-0.00000	0.00000	-0.00000	0.00000	-0.00000
373	C24	S'	-0.00041	-0.00017	-0.00001	-0.00001	-0.00016
374	C24	PX'	-0.00040	0.00001	-0.00001	0.00000	-0.00015
375	C24	PY'	0.00014	-0.00001	0.00000	-0.00000	0.00006
376	C24	PZ'	0.00023	0.00001	0.00001	0.00000	0.00007
377	C24	DXX	0.00000	-0.00000	0.00000	-0.00000	-0.00000
378	C24	DYY	0.00000	-0.00000	0.00000	-0.00000	0.00000
379	C24	DZZ	0.00000	0.00000	0.00000	0.00000	-0.00000
380	C24	DXY	0.00000	0.00000	0.00000	0.00000	-0.00000
381	C24	DXZ	0.00000	0.00000	0.00000	0.00000	0.00000
382	C24	DYZ	0.00000	-0.00000	0.00000	-0.00000	0.00000
383	C25	S1	-0.00000	-0.00001	0.68670	0.71702	-0.00004
384	C25	S	0.00000	0.00000	0.03394	0.03625	-0.00000
385	C25	PX	0.00000	0.00000	-0.00008	-0.00015	0.00000
386	C25	PY	0.00000	0.00000	-0.00025	0.00020	0.00000
387	C25	PZ	-0.00000	-0.00000	0.00034	0.00018	-0.00001
388	C25	S'	-0.00007	0.00003	-0.00721	-0.02009	0.00027
389	C25	PX'	-0.00000	-0.00003	-0.00012	0.00063	-0.00022
390	C25	PY'	-0.00002	-0.00005	0.00149	-0.00335	-0.00030
391	C25	PZ'	0.00002	0.00012	-0.00059	0.00121	0.00073
392	C25	DXX	0.00000	0.00000	-0.00656	-0.00653	0.00000
393	C25	DYY	-0.00000	-0.00000	-0.00659	-0.00627	-0.00000
394	C25	DZZ	-0.00000	0.00000	-0.00641	-0.00628	0.00000
395	C25	DXY	0.00000	0.00000	-0.00001	-0.00009	0.00000
396	C25	DXZ	0.00000	0.00000	-0.00011	-0.00013	-0.00000
397	C25	DYZ	-0.00000	-0.00000	0.00004	-0.00008	-0.00000
398	C26	S1	0.00000	0.00000	-0.00000	-0.00022	0.00001
399	C26	S	0.00000	0.00000	-0.00009	-0.00019	0.00002
400	C26	PX	0.00000	-0.00000	0.00001	0.00001	-0.00000

401	C26	PY	-0.00000	-0.00000	-0.00001	0.00000	-0.00000
402	C26	PZ	0.00000	0.00000	-0.00002	-0.00001	0.00000
403	C26	S'	-0.00002	-0.00001	-0.00001	0.00400	-0.00006
404	C26	PX'	0.00001	0.00001	-0.00003	-0.00075	0.00003
405	C26	PY'	0.00000	0.00000	0.00012	-0.00046	0.00002
406	C26	PZ'	-0.00002	-0.00002	-0.00003	0.00220	-0.00009
407	C26	DXX	0.00000	0.00000	-0.00001	-0.00007	0.00001
408	C26	DYY	0.00000	0.00000	0.00002	-0.00003	0.00000
409	C26	DZZ	-0.00000	-0.00000	0.00004	-0.00006	-0.00000
410	C26	DXY	0.00000	0.00000	-0.00002	-0.00001	0.00000
411	C26	DXZ	0.00000	0.00000	-0.00004	-0.00000	0.00001
412	C26	DYZ	-0.00000	-0.00000	0.00001	-0.00002	-0.00000
413	C27	S1	-0.00001	-0.00003	0.71670	-0.68697	0.00029
414	C27	S	0.00000	0.00000	0.03548	-0.03478	0.00005
415	C27	PX	0.00000	0.00000	-0.00017	0.00010	0.00001
416	C27	PY	-0.00000	-0.00000	0.00041	0.00006	-0.00001
417	C27	PZ	-0.00000	-0.00000	0.00014	-0.00031	-0.00002
418	C27	S'	-0.00024	-0.00042	-0.00645	0.01919	-0.00225
419	C27	PX'	0.00007	0.00013	-0.00058	0.00059	0.00070
420	C27	PY'	-0.00013	-0.00024	-0.00001	-0.00355	-0.00129
421	C27	PZ'	-0.00004	-0.00012	0.00117	0.00142	-0.00071
422	C27	DXX	-0.00000	-0.00000	-0.00684	0.00624	-0.00001
423	C27	DYY	-0.00000	-0.00000	-0.00684	0.00595	-0.00002
424	C27	DZZ	-0.00000	-0.00000	-0.00669	0.00603	-0.00002
425	C27	DXY	0.00000	0.00000	-0.00005	0.00013	0.00001
426	C27	DXZ	0.00000	0.00000	-0.00012	0.00013	0.00001
427	C27	DYZ	-0.00000	-0.00000	0.00010	0.00003	-0.00001
428	C28	S1	0.00000	0.00000	0.00749	0.00794	0.00000
429	C28	S	0.00000	0.00000	0.00002	-0.00000	0.00001
430	C28	PX	-0.00000	-0.00000	0.00011	0.00011	-0.00000
431	C28	PY	0.00000	0.00000	-0.00019	-0.00020	0.00001
432	C28	PZ	0.00000	0.00000	-0.00012	-0.00011	0.00000
433	C28	S'	0.00000	0.00002	0.00277	0.00582	0.00012
434	C28	PX'	0.00000	-0.00000	-0.00066	-0.00067	-0.00001
435	C28	PY'	0.00000	0.00001	0.00075	0.00279	0.00008
436	C28	PZ'	-0.00000	-0.00001	0.00094	-0.00062	-0.00004
437	C28	DXX	0.00000	0.00000	-0.00020	-0.00027	0.00000
438	C28	DYY	0.00000	0.00000	-0.00024	-0.00037	0.00000
439	C28	DZZ	-0.00000	-0.00000	-0.00024	-0.00032	-0.00000

440	C28	DXY	0.00000	-0.00000	0.00004	0.00009	-0.00000
441	C28	DXZ	0.00000	0.00000	0.00005	0.00008	0.00000
442	C28	DYZ	0.00000	0.00000	-0.00006	-0.00009	0.00000
443	C29	S1	0.00000	0.00000	-0.00001	0.00023	0.00000
444	C29	S	0.00000	0.00000	-0.00009	0.00020	0.00000
445	C29	PX	-0.00000	-0.00000	0.00001	-0.00000	-0.00000
446	C29	PY	0.00000	0.00000	-0.00001	0.00001	0.00001
447	C29	PZ	-0.00000	-0.00000	-0.00002	0.00000	-0.00000
448	C29	S'	0.00000	0.00000	0.00012	-0.00405	0.00000
449	C29	PX'	-0.00000	-0.00000	0.00000	0.00111	-0.00000
450	C29	PY'	0.00000	0.00000	-0.00007	-0.00153	0.00001
451	C29	PZ'	-0.00000	-0.00000	0.00007	-0.00149	-0.00001
452	C29	DXX	0.00000	0.00000	-0.00001	0.00007	0.00000
453	C29	DYY	0.00000	0.00000	0.00001	0.00003	0.00000
454	C29	DZZ	-0.00000	0.00000	0.00004	0.00006	0.00000
455	C29	DXY	0.00000	0.00000	-0.00001	0.00001	0.00000
456	C29	DXZ	0.00000	0.00000	-0.00003	0.00000	0.00000
457	C29	DYZ	-0.00000	0.00000	-0.00000	0.00002	0.00000
458	C30	S1	0.00000	0.00000	0.00816	-0.00796	0.00001
459	C30	S	0.00000	0.00000	0.00004	-0.00002	0.00001
460	C30	PX	-0.00000	-0.00000	0.00007	-0.00006	-0.00001
461	C30	PY	0.00000	0.00000	0.00008	-0.00010	0.00001
462	C30	PZ	0.00000	0.00000	-0.00023	0.00023	0.00001
463	C30	S'	0.00003	0.00001	0.00309	-0.00550	0.00002
464	C30	PX'	-0.00000	-0.00001	-0.00043	-0.00030	-0.00003
465	C30	PY'	0.00000	0.00001	-0.00036	0.00251	0.00009
466	C30	PZ'	0.00000	-0.00000	0.00141	-0.00128	-0.00001
467	C30	DXX	0.00000	0.00000	-0.00022	0.00024	0.00001
468	C30	DYY	-0.00000	-0.00000	-0.00021	0.00028	-0.00001
469	C30	DZZ	0.00000	0.00000	-0.00033	0.00043	0.00001
470	C30	DXY	0.00000	0.00000	-0.00001	0.00001	0.00000
471	C30	DXZ	-0.00000	-0.00000	0.00006	-0.00008	-0.00000
472	C30	DYZ	0.00000	0.00000	0.00002	-0.00007	0.00001
473	H15	S	0.00000	0.00000	-0.00005	0.00005	0.00000
474	H15	S'	0.00000	0.00000	-0.00006	0.00048	0.00003
475	H16	S	0.00000	0.00000	-0.00001	-0.00007	0.00000
476	H16	S'	-0.00000	-0.00000	0.00007	-0.00053	-0.00000
477	H17	S	0.00000	-0.00000	-0.00005	-0.00005	-0.00000
478	H17	S'	-0.00000	-0.00001	0.00002	-0.00049	-0.00005

479	H18	S	-0.00000	-0.00000	-0.00001	0.00007	-0.00000
480	H18	S'	-0.00001	-0.00001	0.00007	0.00052	-0.00004
481	C31	S1	-0.00000	-0.00000	-0.00000	-0.00000	-0.00000
482	C31	S	-0.00000	-0.00000	-0.00000	-0.00000	0.00000
483	C31	PX	0.00000	-0.00000	0.00000	-0.00000	0.00000
484	C31	PY	-0.00000	0.00000	-0.00000	0.00000	-0.00000
485	C31	PZ	-0.00000	0.00000	-0.00000	0.00000	0.00000
486	C31	S'	-0.00020	0.00004	-0.00001	0.00000	-0.00007
487	C31	PX'	-0.00017	0.00005	-0.00001	0.00000	-0.00005
488	C31	PY'	0.00014	-0.00001	0.00000	-0.00000	0.00003
489	C31	PZ'	-0.00001	-0.00005	-0.00000	-0.00000	0.00001
490	C31	DXX	-0.00000	0.00000	-0.00000	0.00000	-0.00000
491	C31	DYY	0.00000	0.00000	0.00000	-0.00000	-0.00000
492	C31	DZZ	-0.00000	-0.00000	-0.00000	-0.00000	0.00000
493	C31	DXY	-0.00000	-0.00000	-0.00000	-0.00000	0.00000
494	C31	DXZ	0.00000	-0.00000	0.00000	-0.00000	0.00000
495	C31	DYZ	-0.00000	0.00000	-0.00000	0.00000	-0.00000
496	C32	S1	-0.00000	0.00000	-0.00000	0.00000	-0.00000
497	C32	S	-0.00001	0.00000	-0.00000	0.00000	-0.00000
498	C32	PX	-0.00000	-0.00000	-0.00000	-0.00000	0.00000
499	C32	PY	0.00001	0.00000	0.00000	-0.00000	-0.00000
500	C32	PZ	-0.00002	-0.00000	-0.00000	0.00000	0.00000
501	C32	S'	0.00050	0.00000	0.00002	-0.00000	-0.00000
502	C32	PX'	0.00018	-0.00002	0.00001	-0.00000	0.00001
503	C32	PY'	-0.00009	-0.00000	-0.00000	0.00000	0.00000
504	C32	PZ'	-0.00008	0.00002	-0.00000	0.00000	-0.00001
505	C32	DXX	0.00000	-0.00000	0.00000	-0.00000	0.00000
506	C32	DYY	0.00000	0.00000	0.00000	0.00000	-0.00000
507	C32	DZZ	0.00000	0.00000	0.00000	0.00000	-0.00000
508	C32	DXY	-0.00000	0.00000	-0.00000	0.00000	-0.00000
509	C32	DXZ	0.00000	0.00000	0.00000	0.00000	-0.00000
510	C32	DYZ	-0.00001	-0.00000	-0.00000	0.00000	0.00000
511	C33	S1	-0.00000	0.00000	-0.00000	0.00000	-0.00000
512	C33	S	-0.00001	-0.00000	-0.00000	-0.00000	0.00000
513	C33	PX	-0.00000	-0.00000	-0.00000	0.00000	0.00000
514	C33	PY	-0.00000	0.00000	-0.00000	0.00000	-0.00000
515	C33	PZ	0.00000	-0.00000	0.00000	-0.00000	-0.00000
516	C33	S'	0.00009	0.00004	0.00000	0.00000	-0.00001
517	C33	PX'	-0.00000	-0.00000	-0.00000	-0.00000	0.00000

518	C33	PY'	0.00002	0.00002	0.00000	0.00000	-0.00001
519	C33	PZ'	-0.00003	-0.00002	-0.00000	-0.00000	0.00001
520	C33	DXX	0.00000	0.00000	0.00000	-0.00000	0.00000
521	C33	DYY	-0.00000	0.00000	-0.00000	0.00000	-0.00000
522	C33	DZZ	-0.00000	0.00000	-0.00000	0.00000	-0.00000
523	C33	DXY	-0.00000	0.00000	-0.00000	0.00000	-0.00000
524	C33	DXZ	-0.00000	0.00000	-0.00000	0.00000	-0.00000
525	C33	DYZ	0.00000	-0.00000	0.00000	-0.00000	0.00000
526	C34	S1	0.00000	0.00000	0.00000	0.00000	-0.00000
527	C34	S	0.00002	0.00000	0.00000	-0.00000	-0.00000
528	C34	PX	0.00000	-0.00000	0.00000	-0.00000	-0.00000
529	C34	PY	-0.00001	0.00000	-0.00000	0.00000	-0.00000
530	C34	PZ	0.00000	0.00000	0.00000	-0.00000	0.00000
531	C34	S'	-0.00042	-0.00005	-0.00001	0.00000	0.00001
532	C34	PX'	0.00014	0.00000	0.00000	-0.00000	-0.00000
533	C34	PY'	-0.00006	0.00001	-0.00000	0.00000	0.00000
534	C34	PZ'	-0.00008	-0.00004	-0.00000	0.00000	0.00000
535	C34	DXX	-0.00000	0.00000	-0.00000	0.00000	-0.00000
536	C34	DYY	-0.00000	0.00000	-0.00000	0.00000	-0.00000
537	C34	DZZ	0.00000	-0.00000	0.00000	-0.00000	-0.00000
538	C34	DXY	0.00001	0.00000	0.00000	-0.00000	0.00000
539	C34	DXZ	-0.00000	0.00000	-0.00000	0.00000	-0.00000
540	C34	DYZ	0.00000	0.00000	0.00000	-0.00000	0.00000
541	C35	S1	-0.00004	0.00001	-0.00000	0.00000	0.00000
542	C35	S	-0.00003	0.00001	-0.00000	0.00000	-0.00000
543	C35	PX	-0.00000	-0.00000	-0.00000	0.00000	0.00000
544	C35	PY	-0.00001	0.00000	-0.00000	0.00000	0.00000
545	C35	PZ	0.00003	-0.00000	0.00000	-0.00000	-0.00000
546	C35	S'	-0.00028	-0.00042	-0.00001	-0.00001	0.00019
547	C35	PX'	0.00040	0.00013	0.00001	0.00000	-0.00006
548	C35	PY'	-0.00010	-0.00019	-0.00000	-0.00000	0.00008
549	C35	PZ'	-0.00041	0.00017	-0.00001	0.00001	-0.00006
550	C35	DXX	-0.00001	0.00000	-0.00000	0.00000	0.00000
551	C35	DYY	-0.00001	0.00000	-0.00000	0.00000	0.00000
552	C35	DZZ	-0.00000	0.00000	0.00000	-0.00000	-0.00000
553	C35	DXY	-0.00000	0.00000	-0.00000	0.00000	-0.00000
554	C35	DXZ	-0.00000	0.00000	0.00000	-0.00000	0.00000
555	C35	DYZ	-0.00000	0.00000	-0.00000	0.00000	-0.00000
556	C36	S1	0.00001	0.00000	0.00000	0.00000	-0.00000

557	C36	S	0.00002	0.00001	0.00000	0.00000	-0.00000
558	C36	PX	0.00000	0.00000	0.00000	-0.00000	-0.00000
559	C36	PY	-0.00001	0.00000	-0.00000	0.00000	-0.00000
560	C36	PZ	0.00000	-0.00000	0.00000	-0.00000	0.00000
561	C36	S'	-0.00024	-0.00003	-0.00001	0.00000	0.00001
562	C36	PX'	-0.00002	-0.00000	-0.00000	-0.00000	-0.00000
563	C36	PY'	-0.00006	-0.00001	-0.00000	0.00000	0.00001
564	C36	PZ'	0.00014	0.00003	0.00001	0.00000	-0.00001
565	C36	DXX	0.00000	0.00000	0.00000	0.00000	-0.00000
566	C36	DYY	-0.00000	0.00000	-0.00000	0.00000	-0.00000
567	C36	DZZ	-0.00000	-0.00000	-0.00000	0.00000	0.00000
568	C36	DXY	0.00000	0.00000	0.00000	0.00000	-0.00000
569	C36	DXZ	-0.00000	0.00000	-0.00000	0.00000	0.00000
570	C36	DYZ	0.00001	0.00000	0.00000	0.00000	-0.00000
571	H4	S	0.00000	0.00000	0.00000	0.00000	-0.00000
572	H4	S'	0.00000	0.00001	0.00000	0.00000	-0.00000
573	H19	S	0.00000	-0.00000	0.00000	-0.00000	0.00000
574	H19	S'	-0.00001	-0.00001	-0.00000	0.00000	-0.00000
575	H21	S	-0.00000	-0.00000	-0.00000	-0.00000	0.00000
576	H21	S'	-0.00003	-0.00001	-0.00000	-0.00000	0.00000
577	H22	S	0.00001	0.00000	0.00000	-0.00000	-0.00000
578	H22	S'	0.00003	-0.00001	0.00000	-0.00000	0.00000
579	C37	S1	0.18626	0.97517	0.00003	-0.00001	-0.00154
580	C37	S	0.00874	0.04899	-0.00000	0.00000	0.00002
581	C37	PX	0.00025	0.00007	-0.00000	-0.00000	0.00001
582	C37	PY	-0.00028	-0.00021	-0.00000	0.00000	0.00002
583	C37	PZ	-0.00010	0.00024	-0.00000	-0.00000	-0.00005
584	C37	S'	0.00602	-0.02240	-0.00009	0.00000	-0.00174
585	C37	PX'	-0.00221	0.00089	-0.00005	0.00006	0.00125
586	C37	PY'	0.00173	-0.00059	0.00014	-0.00012	-0.00322
587	C37	PZ'	0.00226	-0.00038	-0.00018	0.00012	0.00440
588	C37	DXX	-0.00202	-0.00897	-0.00000	0.00000	0.00007
589	C37	DYY	-0.00203	-0.00887	-0.00000	0.00000	0.00007
590	C37	DZZ	-0.00198	-0.00886	0.00000	-0.00000	0.00006
591	C37	DXY	0.00008	-0.00022	0.00000	-0.00000	-0.00001
592	C37	DXZ	0.00007	-0.00004	-0.00000	0.00000	0.00001
593	C37	DYZ	-0.00007	-0.00006	0.00000	-0.00000	-0.00002
594	C38	S1	0.00013	-0.00017	-0.00000	0.00000	0.01264
595	C38	S	0.00007	-0.00017	-0.00001	0.00001	0.00011

596	C38	PX	0.00000	-0.00001	0.00001	-0.00000	-0.00024
597	C38	PY	0.00001	0.00001	-0.00000	0.00000	0.00026
598	C38	PZ	-0.00001	-0.00001	-0.00002	0.00001	0.00013
599	C38	S'	-0.00274	0.00276	0.00046	-0.00026	0.00645
600	C38	PX'	-0.00069	0.00045	0.00008	-0.00006	0.00180
601	C38	PY'	0.00122	-0.00118	-0.00013	0.00008	-0.00254
602	C38	PZ'	-0.00092	0.00160	0.00009	-0.00005	0.00032
603	C38	DXX	0.00002	-0.00004	0.00000	-0.00000	-0.00043
604	C38	DYY	0.00003	-0.00003	-0.00000	0.00000	-0.00043
605	C38	DZZ	0.00008	-0.00004	0.00001	-0.00000	-0.00040
606	C38	DXY	0.00001	-0.00003	-0.00000	0.00000	0.00015
607	C38	DXZ	-0.00000	-0.00000	-0.00001	0.00000	0.00008
608	C38	DYZ	-0.00002	-0.00002	0.00000	0.00000	-0.00007
609	C39	S1	0.97507	-0.18666	0.00000	-0.00000	0.00089
610	C39	S	0.04876	-0.00987	-0.00001	0.00000	-0.00005
611	C39	PX	-0.00014	-0.00022	-0.00000	0.00000	-0.00001
612	C39	PY	-0.00005	0.00027	0.00000	-0.00000	-0.00001
613	C39	PZ	0.00041	0.00004	-0.00000	-0.00000	0.00008
614	C39	S'	-0.01763	0.01194	0.00037	-0.00005	0.00103
615	C39	PX'	-0.00097	0.00211	-0.00008	0.00003	0.00073
616	C39	PY'	0.00126	-0.00272	0.00003	-0.00002	0.00096
617	C39	PZ'	0.00082	-0.00032	0.00017	-0.00006	-0.00453
618	C39	DXX	-0.00914	0.00141	-0.00000	0.00000	-0.00004
619	C39	DYY	-0.00909	0.00140	0.00000	-0.00000	-0.00002
620	C39	DZZ	-0.00881	0.00138	0.00000	-0.00000	-0.00004
621	C39	DXY	-0.00008	0.00017	-0.00000	0.00000	-0.00002
622	C39	DXZ	-0.00007	0.00009	0.00000	-0.00000	-0.00000
623	C39	DYZ	-0.00011	-0.00002	-0.00000	0.00000	0.00000
624	C40	S1	0.00204	0.01118	0.00000	-0.00000	0.01172
625	C40	S	-0.00000	-0.00004	0.00002	-0.00001	-0.00009
626	C40	PX	0.00002	0.00012	-0.00000	0.00000	0.00014
627	C40	PY	-0.00000	-0.00005	-0.00000	0.00000	-0.00024
628	C40	PZ	-0.00005	-0.00032	0.00001	-0.00001	0.00016
629	C40	S'	-0.00009	0.00812	-0.00047	0.00028	0.01049
630	C40	PX'	0.00072	-0.00212	0.00011	-0.00007	-0.00284
631	C40	PY'	-0.00101	0.00187	-0.00015	0.00009	0.00340
632	C40	PZ'	0.00034	0.00201	0.00003	-0.00003	0.00032
633	C40	DXX	-0.00002	-0.00032	0.00000	-0.00000	-0.00033
634	C40	DYY	-0.00003	-0.00032	0.00000	-0.00000	-0.00043

635	C40	DZZ	0.00001	-0.00051	-0.00001	0.00001	-0.00037
636	C40	DXY	-0.00001	0.00002	0.00000	0.00000	0.00006
637	C40	DXZ	-0.00001	0.00007	0.00000	-0.00000	-0.00006
638	C40	DYZ	0.00001	-0.00001	0.00000	-0.00000	0.00009
639	C41	S1	-0.00065	0.00162	-0.00018	0.00024	0.99267
640	C41	S	-0.00016	0.00022	-0.00002	0.00003	0.04974
641	C41	PX	0.00002	0.00001	-0.00001	0.00001	0.00013
642	C41	PY	0.00001	-0.00002	0.00001	-0.00001	-0.00007
643	C41	PZ	-0.00006	0.00001	0.00002	-0.00001	-0.00039
644	C41	S'	0.00193	-0.00262	-0.00103	0.00006	-0.01867
645	C41	PX'	-0.00064	0.00005	0.00021	-0.00018	-0.00011
646	C41	PY'	0.00024	0.00101	-0.00046	0.00028	0.00141
647	C41	PZ'	0.00177	-0.00323	0.00051	-0.00000	-0.00295
648	C41	DXX	-0.00001	0.00004	-0.00001	0.00001	-0.00922
649	C41	DYY	0.00000	0.00003	-0.00000	0.00000	-0.00917
650	C41	DZZ	-0.00001	0.00005	-0.00001	0.00001	-0.00898
651	C41	DXY	-0.00004	0.00001	-0.00000	0.00000	-0.00018
652	C41	DXZ	-0.00001	-0.00001	0.00000	-0.00000	-0.00013
653	C41	DYZ	-0.00001	0.00000	0.00001	-0.00000	0.00003
654	C42	S1	0.01086	-0.00220	0.00001	-0.00000	0.00023
655	C42	S	0.00002	-0.00003	0.00003	-0.00001	0.00009
656	C42	PX	-0.00016	0.00003	0.00000	0.00000	-0.00001
657	C42	PY	0.00027	-0.00006	-0.00000	0.00000	0.00002
658	C42	PZ	-0.00018	0.00003	0.00000	-0.00000	0.00000
659	C42	S'	0.00564	-0.00271	-0.00026	0.00010	-0.00384
660	C42	PX'	0.00149	-0.00101	-0.00009	0.00003	-0.00149
661	C42	PY'	-0.00210	0.00091	0.00008	-0.00003	0.00176
662	C42	PZ'	0.00025	0.00117	0.00008	-0.00003	0.00037
663	C42	DXX	-0.00034	0.00011	0.00001	-0.00000	0.00004
664	C42	DYY	-0.00040	0.00015	0.00000	-0.00000	0.00002
665	C42	DZZ	-0.00040	0.00012	-0.00001	0.00000	0.00009
666	C42	DXY	0.00009	-0.00007	0.00000	-0.00000	0.00001
667	C42	DXZ	-0.00006	0.00002	0.00000	-0.00000	-0.00004
668	C42	DYZ	0.00012	-0.00006	0.00000	-0.00000	0.00002
669	H23	S	-0.00007	0.00003	-0.00000	-0.00000	0.00002
670	H23	S'	-0.00035	0.00047	-0.00002	0.00001	0.00063
671	H25	S	-0.00000	-0.00006	-0.00000	0.00000	-0.00006
672	H25	S'	0.00031	-0.00031	-0.00003	0.00001	-0.00048
673	H26	S	-0.00006	0.00006	0.00001	-0.00000	-0.00002

674	H26	S'	-0.00034	0.00055	0.00002	-0.00002	0.00040
675	C43	S1	0.00002	-0.00000	0.00000	-0.00000	0.00000
676	C43	S	0.00009	-0.00001	0.00000	-0.00000	-0.00000
677	C43	PX	0.00001	-0.00000	0.00000	-0.00000	0.00000
678	C43	PY	0.00001	-0.00001	-0.00000	0.00000	-0.00000
679	C43	PZ	-0.00002	0.00002	0.00000	0.00000	-0.00000
680	C43	S'	-0.00467	-0.00146	-0.00019	0.00001	0.00077
681	C43	PX'	-0.00002	0.00018	-0.00000	0.00001	-0.00008
682	C43	PY'	-0.00111	-0.00068	-0.00005	-0.00001	0.00032
683	C43	PZ'	0.00201	0.00087	0.00008	0.00001	-0.00043
684	C43	DXX	0.00001	-0.00000	0.00000	-0.00000	0.00000
685	C43	DYY	0.00002	-0.00001	0.00000	-0.00000	0.00000
686	C43	DZZ	0.00003	-0.00002	-0.00000	-0.00000	-0.00000
687	C43	DXY	0.00001	0.00000	0.00000	-0.00000	0.00000
688	C43	DXZ	0.00001	-0.00000	0.00000	0.00000	-0.00000
689	C43	DYZ	-0.00001	0.00001	0.00000	-0.00000	0.00000
690	C44	S1	-0.00004	0.00001	-0.00000	0.00000	-0.00000
691	C44	S	0.00000	0.00002	0.00000	0.00000	-0.00001
692	C44	PX	-0.00003	0.00000	-0.00000	0.00000	-0.00000
693	C44	PY	0.00002	0.00000	0.00000	-0.00000	0.00000
694	C44	PZ	0.00002	-0.00002	-0.00000	-0.00000	0.00000
695	C44	S'	-0.00197	-0.00095	-0.00013	0.00002	0.00066
696	C44	PX'	0.00018	-0.00012	0.00001	-0.00000	0.00005
697	C44	PY'	-0.00314	-0.00109	-0.00014	0.00000	0.00060
698	C44	PZ'	0.00534	0.00227	0.00024	0.00000	-0.00120
699	C44	DXX	-0.00002	0.00000	-0.00000	0.00000	-0.00000
700	C44	DYY	-0.00005	0.00002	-0.00000	0.00000	0.00000
701	C44	DZZ	-0.00012	0.00004	-0.00000	-0.00000	0.00001
702	C44	DXY	-0.00000	-0.00000	0.00000	-0.00000	0.00000
703	C44	DXZ	-0.00000	0.00000	-0.00000	0.00000	-0.00000
704	C44	DYZ	0.00007	-0.00002	0.00000	0.00000	-0.00001
705	C45	S1	0.00869	-0.00177	0.00000	-0.00000	0.00002
706	C45	S	0.00001	-0.00013	0.00000	0.00000	-0.00002
707	C45	PX	-0.00012	0.00002	0.00000	-0.00000	-0.00000
708	C45	PY	0.00000	0.00000	0.00000	-0.00000	0.00000
709	C45	PZ	0.00031	-0.00003	-0.00000	0.00000	-0.00002
710	C45	S'	0.01161	0.00112	0.00031	-0.00002	0.00037
711	C45	PX'	-0.00034	-0.00133	-0.00013	0.00002	0.00106
712	C45	PY'	-0.00116	-0.00015	-0.00011	0.00003	0.00042

713	C45	PZ'	0.00232	0.00347	0.00050	-0.00009	-0.00315
714	C45	DXX	-0.00033	0.00007	0.00000	-0.00000	0.00000
715	C45	DYY	-0.00031	0.00010	0.00000	-0.00000	-0.00001
716	C45	DZZ	-0.00077	0.00028	0.00000	-0.00000	0.00003
717	C45	DXY	0.00005	-0.00004	0.00000	0.00000	0.00000
718	C45	DXZ	0.00018	-0.00008	-0.00000	0.00000	-0.00002
719	C45	DYZ	0.00008	-0.00007	-0.00000	-0.00000	0.00000
720	C46	S1	0.00040	-0.00011	0.00000	-0.00000	0.00000
721	C46	S	0.00024	-0.00006	0.00000	-0.00000	-0.00000
722	C46	PX	0.00005	-0.00002	0.00000	-0.00000	-0.00000
723	C46	PY	0.00006	-0.00001	-0.00000	0.00000	-0.00000
724	C46	PZ	-0.00017	0.00004	0.00000	-0.00000	0.00002
725	C46	S'	-0.00163	-0.00119	-0.00031	0.00009	0.00148
726	C46	PX'	-0.00164	-0.00041	-0.00009	0.00001	0.00030
727	C46	PY'	-0.00238	-0.00138	-0.00015	0.00001	0.00084
728	C46	PZ'	0.00795	0.00328	0.00047	-0.00005	-0.00217
729	C46	DXX	0.00001	0.00001	0.00000	-0.00000	-0.00000
730	C46	DYY	0.00007	-0.00001	0.00000	-0.00000	-0.00000
731	C46	DZZ	0.00019	-0.00009	-0.00000	0.00000	-0.00001
732	C46	DXY	0.00001	-0.00001	-0.00000	-0.00000	-0.00000
733	C46	DXZ	-0.00000	0.00002	0.00000	-0.00000	0.00000
734	C46	DYZ	-0.00013	0.00005	0.00000	-0.00000	0.00000
735	C47	S1	-0.00001	-0.00004	0.00001	-0.00001	0.00890
736	C47	S	-0.00005	-0.00009	0.00008	-0.00006	-0.00001
737	C47	PX	-0.00001	-0.00001	0.00001	-0.00001	0.00006
738	C47	PY	0.00000	-0.00000	-0.00001	0.00002	0.00001
739	C47	PZ	0.00001	0.00001	-0.00000	0.00002	-0.00036
740	C47	S'	0.00181	0.00193	-0.00667	0.00237	0.01476
741	C47	PX'	0.00014	0.00107	-0.00146	0.00044	0.00216
742	C47	PY'	-0.00018	-0.00096	0.00127	-0.00050	-0.00235
743	C47	PZ'	0.00009	-0.00230	0.00347	-0.00086	-0.00413
744	C47	DXX	-0.00001	-0.00004	0.00001	-0.00001	-0.00032
745	C47	DYY	-0.00000	-0.00003	0.00000	-0.00000	-0.00031
746	C47	DZZ	0.00003	-0.00001	0.00001	-0.00004	-0.00084
747	C47	DXY	-0.00001	-0.00001	0.00000	0.00000	0.00007
748	C47	DXZ	-0.00000	0.00001	-0.00000	0.00002	0.00015
749	C47	DYZ	-0.00000	-0.00002	0.00000	-0.00001	-0.00003
750	C48	S1	-0.00000	-0.00000	-0.00003	0.00004	0.00046
751	C48	S	0.00000	0.00002	0.00004	-0.00000	0.00019

752	C48	PX	0.00000	-0.00000	-0.00001	0.00001	-0.00008
753	C48	PY	0.00000	0.00001	0.00002	-0.00003	0.00009
754	C48	PZ	-0.00000	0.00001	-0.00002	-0.00000	0.00019
755	C48	S'	0.00023	-0.00114	-0.00313	0.00103	-0.00079
756	C48	PX'	0.00037	0.00113	-0.00356	0.00111	0.00488
757	C48	PY'	-0.00038	-0.00115	0.00363	-0.00096	-0.00487
758	C48	PZ'	-0.00100	-0.00271	0.00830	-0.00274	-0.01166
759	C48	DXX	-0.00000	0.00000	-0.00002	0.00003	0.00006
760	C48	DYY	0.00000	0.00000	-0.00002	0.00002	0.00010
761	C48	DZZ	-0.00001	-0.00000	-0.00009	0.00011	0.00014
762	C48	DXY	-0.00000	-0.00001	0.00002	-0.00002	-0.00009
763	C48	DXZ	0.00000	-0.00001	0.00004	-0.00005	-0.00010
764	C48	DYZ	-0.00000	0.00001	-0.00004	0.00005	0.00014
765	C49	S1	0.00000	0.00000	0.00648	-0.00633	0.00001
766	C49	S	0.00001	0.00001	-0.00004	-0.00010	0.00008
767	C49	PX	0.00000	-0.00000	-0.00011	0.00008	-0.00002
768	C49	PY	-0.00000	0.00000	0.00017	-0.00017	0.00003
769	C49	PZ	-0.00000	0.00000	0.00014	-0.00010	0.00003
770	C49	S'	-0.00085	-0.00151	0.01289	-0.00692	-0.00777
771	C49	PX'	0.00028	0.00051	-0.00278	-0.00004	0.00271
772	C49	PY'	-0.00040	-0.00073	0.00400	0.00074	-0.00384
773	C49	PZ'	-0.00047	-0.00081	0.00448	-0.00051	-0.00428
774	C49	DXX	0.00000	0.00000	-0.00023	0.00025	0.00001
775	C49	DYY	-0.00000	-0.00000	-0.00036	0.00046	0.00002
776	C49	DZZ	-0.00000	-0.00000	-0.00032	0.00045	0.00001
777	C49	DXY	0.00000	0.00000	0.00012	-0.00018	-0.00001
778	C49	DXZ	0.00000	0.00000	0.00011	-0.00018	-0.00001
779	C49	DYZ	-0.00000	-0.00000	-0.00013	0.00017	0.00002
780	C50	S1	-0.00000	-0.00000	0.00029	-0.00032	-0.00004
781	C50	S	-0.00000	0.00000	0.00016	-0.00017	0.00003
782	C50	PX	-0.00000	-0.00000	0.00007	-0.00006	-0.00001
783	C50	PY	0.00000	0.00000	-0.00009	0.00009	0.00002
784	C50	PZ	0.00000	-0.00000	-0.00012	0.00011	-0.00002
785	C50	S'	-0.00000	-0.00047	-0.00236	0.00016	-0.00156
786	C50	PX'	0.00051	0.00096	-0.00531	0.00166	0.00483
787	C50	PY'	-0.00064	-0.00121	0.00672	-0.00242	-0.00614
788	C50	PZ'	-0.00087	-0.00174	0.00971	-0.00280	-0.00880
789	C50	DXX	-0.00000	-0.00000	0.00003	-0.00003	-0.00004
790	C50	DYY	0.00000	-0.00000	0.00002	-0.00003	-0.00004

791	C50	DZZ	0.00000	-0.00001	0.00013	-0.00017	-0.00012
792	C50	DXY	-0.00000	0.00000	-0.00001	0.00004	0.00003
793	C50	DXZ	-0.00000	0.00000	-0.00005	0.00009	0.00006
794	C50	DYZ	0.00000	-0.00000	0.00005	-0.00009	-0.00007

MO: 6 7 8 9 10

Eigenvalues: -10.22419 -10.22376 -10.22291 -10.22264 -10.21845

(ev) -278.21454 -278.20265 -278.17979 -278.17232 -278.05828

			A	A	A	A	A
1	C1	S1	0.00000	0.01787	0.00000	0.00000	-0.00009
2	C1	S	-0.00000	0.00036	-0.00002	-0.00000	-0.00007
3	C1	PX	0.00000	-0.00006	0.00001	0.00000	0.00001
4	C1	PY	0.00000	-0.00022	-0.00000	-0.00000	0.00001
5	C1	PZ	0.00000	-0.00025	0.00001	0.00000	0.00004
6	C1	S'	0.00006	0.00748	0.00036	0.00006	-0.00014
7	C1	PX'	0.00000	0.00076	-0.00014	-0.00002	0.00001
8	C1	PY'	0.00001	0.00234	0.00048	0.00005	-0.00017
9	C1	PZ'	0.00003	0.00332	0.00022	0.00002	-0.00050
10	C1	DXX	0.00000	-0.00035	-0.00000	0.00000	-0.00004
11	C1	DYY	0.00000	-0.00048	0.00000	-0.00000	-0.00003
12	C1	DZZ	0.00000	-0.00050	0.00001	0.00000	-0.00001
13	C1	DXY	-0.00000	0.00003	0.00000	0.00000	-0.00001
14	C1	DXZ	0.00000	-0.00000	0.00000	0.00000	-0.00000
15	C1	DYZ	0.00000	-0.00012	0.00001	0.00000	-0.00001
16	C4	S1	0.00000	0.00025	0.00016	0.00001	0.00000
17	C4	S	0.00001	0.00010	-0.00004	0.00004	0.00001
18	C4	PX	0.00000	-0.00001	0.00002	0.00001	-0.00000
19	C4	PY	-0.00000	0.00001	0.00003	-0.00000	0.00000
20	C4	PZ	0.00000	0.00001	-0.00001	0.00001	-0.00000
21	C4	S'	-0.00014	-0.00299	0.00001	-0.00054	0.00006
22	C4	PX'	-0.00007	0.00117	0.00021	-0.00037	0.00003
23	C4	PY'	0.00002	-0.00073	-0.00077	0.00002	0.00005
24	C4	PZ'	-0.00009	0.00108	-0.00078	-0.00055	0.00012
25	C4	DXX	-0.00000	0.00006	0.00004	0.00000	0.00000
26	C4	DYY	0.00000	0.00005	-0.00003	0.00001	-0.00000
27	C4	DZZ	-0.00000	0.00003	0.00001	-0.00001	0.00000
28	C4	DXY	0.00000	-0.00002	0.00000	0.00000	-0.00000
29	C4	DXZ	-0.00000	0.00000	-0.00001	-0.00001	-0.00000
30	C4	DYZ	-0.00000	-0.00000	0.00002	0.00000	-0.00000

31 C2	S1	0.00000	0.00021	-0.00000	0.00000	-0.00002
32 C2	S	0.00000	0.00006	-0.00000	0.00000	0.00000
33 C2	PX	0.00000	0.00001	0.00001	0.00000	-0.00000
34 C2	PY	-0.00000	-0.00001	0.00000	0.00000	-0.00000
35 C2	PZ	0.00000	0.00002	0.00000	0.00000	0.00002
36 C2	S'	-0.00002	-0.00237	0.00015	0.00007	-0.00009
37 C2	PX'	-0.00000	-0.00046	-0.00022	-0.00001	0.00006
38 C2	PY'	-0.00001	-0.00097	0.00020	0.00002	-0.00005
39 C2	PZ'	-0.00002	-0.00163	-0.00014	-0.00000	0.00005
40 C2	DXX	0.00000	0.00005	-0.00000	0.00000	-0.00001
41 C2	DYY	0.00000	0.00006	0.00000	0.00000	-0.00000
42 C2	DZZ	-0.00000	0.00003	-0.00000	0.00000	-0.00001
43 C2	DXY	0.00000	-0.00003	-0.00000	0.00000	-0.00000
44 C2	DXZ	-0.00000	0.00000	-0.00001	-0.00000	-0.00001
45 C2	DYZ	-0.00000	0.00001	0.00000	-0.00000	0.00001
46 C6	S1	0.00000	0.99263	0.00013	-0.00002	0.00002
47 C6	S	-0.00000	0.04964	-0.00001	-0.00001	0.00002
48 C6	PX	0.00000	-0.00019	0.00001	0.00000	0.00001
49 C6	PY	-0.00000	0.00036	-0.00002	-0.00000	0.00000
50 C6	PZ	0.00000	-0.00014	-0.00002	0.00000	-0.00000
51 C6	S'	0.00003	-0.01922	0.00056	0.00031	-0.00053
52 C6	PX'	0.00001	-0.00083	0.00002	-0.00003	0.00027
53 C6	PY'	0.00006	0.00189	0.00065	0.00015	-0.00074
54 C6	PZ'	0.00006	0.00127	0.00070	0.00010	0.00002
55 C6	DXX	0.00000	-0.00915	0.00000	0.00000	-0.00000
56 C6	DYY	-0.00000	-0.00903	-0.00000	0.00000	0.00000
57 C6	DZZ	-0.00000	-0.00908	-0.00000	0.00000	-0.00000
58 C6	DXY	-0.00000	-0.00016	-0.00001	-0.00000	0.00001
59 C6	DXZ	0.00000	0.00016	0.00001	0.00000	-0.00000
60 C6	DYZ	-0.00000	-0.00000	-0.00002	-0.00000	0.00001
61 C5	S1	-0.00000	0.01162	0.00001	0.00000	0.00000
62 C5	S	0.00000	0.00003	0.00003	0.00001	0.00001
63 C5	PX	0.00000	0.00020	-0.00001	-0.00001	-0.00000
64 C5	PY	0.00000	-0.00002	0.00001	-0.00000	0.00000
65 C5	PZ	-0.00000	0.00031	0.00001	-0.00000	0.00000
66 C5	S'	-0.00008	0.00676	-0.00022	-0.00015	-0.00010
67 C5	PX'	0.00002	-0.00064	0.00051	0.00010	-0.00002
68 C5	PY'	-0.00003	-0.00120	-0.00051	0.00007	0.00012
69 C5	PZ'	0.00005	-0.00248	0.00034	0.00019	0.00003

70	C5	DXX	-0.00000	-0.00039	0.00002	0.00000	0.00000
71	C5	DYY	0.00000	-0.00033	0.00003	-0.00000	0.00000
72	C5	DZZ	-0.00000	-0.00047	-0.00001	-0.00000	0.00000
73	C5	DXY	0.00000	0.00002	-0.00001	0.00000	-0.00000
74	C5	DXZ	-0.00000	-0.00015	-0.00001	-0.00000	-0.00000
75	C5	DYZ	0.00000	0.00001	-0.00000	-0.00001	-0.00001
76	C3	S1	0.00000	-0.00015	0.00002	0.00000	-0.00000
77	C3	S	0.00000	-0.00016	0.00003	0.00001	-0.00001
78	C3	PX	0.00000	0.00000	0.00000	0.00000	0.00000
79	C3	PY	-0.00000	-0.00002	-0.00001	-0.00000	0.00000
80	C3	PZ	0.00000	-0.00000	0.00001	0.00000	-0.00000
81	C3	S'	0.00000	0.00258	-0.00057	-0.00016	0.00007
82	C3	PX'	0.00000	-0.00099	-0.00004	0.00008	-0.00003
83	C3	PY'	-0.00001	0.00117	-0.00034	-0.00013	0.00007
84	C3	PZ'	-0.00000	-0.00054	-0.00040	-0.00000	0.00004
85	C3	DXX	0.00000	-0.00003	0.00003	0.00000	0.00000
86	C3	DYY	-0.00000	-0.00002	-0.00001	-0.00000	0.00000
87	C3	DZZ	0.00000	-0.00001	0.00002	0.00001	0.00000
88	C3	DXY	0.00000	-0.00002	0.00001	0.00001	-0.00000
89	C3	DXZ	0.00000	0.00002	0.00001	0.00000	0.00000
90	C3	DYZ	-0.00000	0.00001	-0.00001	-0.00000	0.00000
91	H2	S	-0.00000	-0.00008	-0.00000	-0.00000	-0.00000
92	H2	S'	-0.00001	-0.00043	-0.00000	-0.00001	0.00001
93	H5	S	-0.00001	0.00000	-0.00004	0.00001	-0.00000
94	H5	S'	0.00003	0.00028	0.00024	-0.00011	-0.00004
95	H3	S	-0.00000	0.00004	0.00002	-0.00000	0.00000
96	H3	S'	-0.00001	0.00039	-0.00006	-0.00006	0.00002
97	C7	S1	0.00000	-0.00004	0.02518	0.00004	0.00001
98	C7	S	0.00001	-0.00004	0.00073	-0.00001	0.00001
99	C7	PX	0.00000	0.00003	0.00009	-0.00000	-0.00000
100	C7	PY	0.00000	-0.00001	-0.00003	0.00000	0.00000
101	C7	PZ	0.00000	0.00001	-0.00036	0.00002	-0.00000
102	C7	S'	-0.00008	-0.00029	0.00658	0.00023	-0.00007
103	C7	PX'	-0.00011	-0.00044	-0.00064	-0.00071	0.00010
104	C7	PY'	-0.00001	0.00028	0.00040	0.00015	-0.00003
105	C7	PZ'	-0.00020	0.00021	0.00340	-0.00057	0.00004
106	C7	DXX	0.00000	-0.00003	-0.00049	0.00001	0.00000
107	C7	DYY	0.00000	-0.00001	-0.00043	0.00001	0.00000
108	C7	DZZ	-0.00000	-0.00001	-0.00069	0.00001	-0.00000

109	C7	DXY	0.00000	-0.00002	0.00002	0.00000	0.00000
110	C7	DXZ	-0.00000	-0.00000	0.00006	-0.00001	-0.00000
111	C7	DYZ	-0.00000	-0.00002	-0.00004	-0.00000	0.00000
112	C8	S1	0.00000	-0.00000	0.00016	0.00000	0.00000
113	C8	S	0.00001	0.00000	0.00012	0.00002	0.00000
114	C8	PX	-0.00000	-0.00000	0.00002	-0.00002	-0.00000
115	C8	PY	0.00000	0.00000	-0.00001	0.00001	-0.00000
116	C8	PZ	-0.00000	-0.00000	-0.00002	-0.00000	-0.00000
117	C8	S'	-0.00001	0.00009	-0.00314	0.00023	-0.00002
118	C8	PX'	0.00002	0.00000	0.00138	-0.00004	0.00001
119	C8	PY'	-0.00000	-0.00001	-0.00038	0.00002	-0.00000
120	C8	PZ'	0.00004	-0.00004	0.00134	-0.00000	0.00001
121	C8	DXX	-0.00000	-0.00000	0.00007	0.00000	0.00000
122	C8	DYY	0.00000	0.00000	0.00005	0.00001	0.00000
123	C8	DZZ	-0.00000	0.00000	0.00003	-0.00000	-0.00000
124	C8	DXY	0.00000	0.00000	-0.00001	0.00000	0.00000
125	C8	DXZ	-0.00000	-0.00000	0.00000	-0.00000	0.00000
126	C8	DYZ	0.00000	0.00000	-0.00000	0.00000	-0.00000
127	C9	S1	-0.00003	-0.00012	0.99246	0.00161	0.00000
128	C9	S	0.00002	0.00003	0.04965	0.00019	-0.00000
129	C9	PX	0.00001	0.00000	-0.00038	0.00005	0.00000
130	C9	PY	-0.00001	-0.00000	0.00013	-0.00002	-0.00000
131	C9	PZ	0.00000	0.00000	0.00012	-0.00003	0.00000
132	C9	S'	-0.00086	-0.00075	-0.01964	-0.00325	0.00007
133	C9	PX'	0.00058	0.00048	-0.00064	0.00261	-0.00008
134	C9	PY'	-0.00013	-0.00018	0.00018	-0.00070	0.00003
135	C9	PZ'	-0.00032	0.00012	0.00068	-0.00050	-0.00001
136	C9	DXX	-0.00002	-0.00001	-0.00899	-0.00013	0.00000
137	C9	DYY	0.00001	0.00001	-0.00930	-0.00001	-0.00000
138	C9	DZZ	-0.00000	-0.00001	-0.00903	-0.00003	0.00000
139	C9	DXY	0.00000	0.00000	-0.00013	0.00004	-0.00000
140	C9	DXZ	0.00000	-0.00001	-0.00005	0.00003	-0.00000
141	C9	DYZ	-0.00000	0.00000	0.00003	-0.00001	-0.00000
142	C10	S1	0.00000	-0.00001	0.00027	0.00001	0.00000
143	C10	S	0.00001	-0.00001	0.00003	0.00003	0.00000
144	C10	PX	-0.00000	-0.00000	-0.00000	-0.00001	0.00000
145	C10	PY	0.00000	0.00001	0.00000	0.00000	-0.00000
146	C10	PZ	0.00000	0.00001	0.00001	0.00002	0.00000
147	C10	S'	0.00002	0.00040	-0.00181	0.00021	-0.00007

148	C10	PX'	0.00001	-0.00022	-0.00025	-0.00004	0.00001
149	C10	PY'	0.00000	0.00002	0.00001	0.00002	-0.00000
150	C10	PZ'	0.00001	0.00005	-0.00164	0.00002	-0.00003
151	C10	DXX	-0.00000	0.00001	0.00007	-0.00000	-0.00000
152	C10	DYY	0.00000	-0.00001	0.00002	0.00001	0.00000
153	C10	DZZ	-0.00000	-0.00001	0.00004	-0.00000	-0.00000
154	C10	DXY	0.00000	0.00000	-0.00002	0.00000	0.00000
155	C10	DXZ	-0.00000	-0.00001	-0.00004	-0.00000	-0.00000
156	C10	DYZ	0.00000	-0.00000	0.00001	-0.00000	0.00000
157	C11	S1	0.00000	0.00000	-0.00016	0.00000	-0.00000
158	C11	S	0.00000	0.00001	-0.00019	0.00001	-0.00000
159	C11	PX	-0.00000	-0.00000	0.00002	-0.00001	-0.00000
160	C11	PY	0.00000	0.00000	-0.00001	0.00000	0.00000
161	C11	PZ	0.00000	0.00000	0.00001	0.00000	-0.00000
162	C11	S'	0.00001	-0.00006	0.00292	-0.00007	0.00004
163	C11	PX'	0.00000	0.00006	-0.00157	0.00006	-0.00002
164	C11	PY'	-0.00000	-0.00001	0.00047	-0.00002	0.00001
165	C11	PZ'	0.00001	-0.00008	0.00029	-0.00003	-0.00000
166	C11	DXX	0.00000	0.00000	-0.00001	0.00000	0.00000
167	C11	DYY	0.00000	0.00000	-0.00006	0.00001	-0.00000
168	C11	DZZ	0.00000	0.00000	0.00000	-0.00000	0.00000
169	C11	DXY	0.00000	-0.00000	-0.00002	0.00000	-0.00000
170	C11	DXZ	-0.00000	0.00000	0.00000	0.00000	0.00000
171	C11	DYZ	0.00000	-0.00000	0.00000	-0.00000	-0.00000
172	C12	S1	0.00000	-0.00000	0.01099	0.00003	-0.00000
173	C12	S	0.00001	0.00000	0.00004	0.00005	-0.00000
174	C12	PX	-0.00000	-0.00000	0.00016	0.00000	0.00000
175	C12	PY	0.00000	0.00000	-0.00004	-0.00000	-0.00000
176	C12	PZ	0.00000	0.00000	0.00027	-0.00000	-0.00000
177	C12	S'	0.00000	-0.00016	0.00672	-0.00064	0.00004
178	C12	PX'	-0.00005	0.00002	-0.00045	-0.00011	-0.00001
179	C12	PY'	0.00001	-0.00001	0.00007	0.00002	0.00000
180	C12	PZ'	0.00004	0.00008	-0.00289	0.00046	-0.00002
181	C12	DXX	0.00001	0.00000	-0.00043	0.00000	0.00000
182	C12	DYY	0.00000	0.00000	-0.00028	0.00002	-0.00000
183	C12	DZZ	-0.00000	-0.00000	-0.00044	-0.00001	0.00000
184	C12	DXY	0.00000	-0.00000	0.00005	0.00001	-0.00000
185	C12	DXZ	0.00000	0.00000	-0.00013	0.00001	0.00000
186	C12	DYZ	0.00000	-0.00000	0.00003	0.00000	0.00000

187	H7	S	0.00000	-0.00000	-0.00007	-0.00001	-0.00000
188	H7	S'	-0.00000	0.00001	-0.00034	-0.00001	-0.00000
189	H8	S	0.00000	-0.00001	0.00004	-0.00000	0.00000
190	H8	S'	-0.00000	-0.00002	0.00040	-0.00003	0.00000
191	H9	S	-0.00000	-0.00000	0.00001	-0.00001	-0.00000
192	H9	S'	-0.00002	-0.00001	0.00033	-0.00016	0.00000
193	H10	S	-0.00000	-0.00000	-0.00006	-0.00000	-0.00000
194	H10	S'	-0.00002	-0.00000	-0.00041	-0.00003	-0.00000
195	C13	S1	0.00000	0.00010	-0.00000	-0.00000	0.00032
196	C13	S	0.00000	-0.00001	-0.00000	-0.00000	0.00013
197	C13	PX	0.00000	-0.00000	-0.00000	0.00000	0.00001
198	C13	PY	0.00000	0.00000	-0.00001	-0.00000	-0.00000
199	C13	PZ	0.00000	0.00002	-0.00000	0.00000	-0.00002
200	C13	S'	-0.00000	-0.00003	0.00001	0.00000	-0.00327
201	C13	PX'	-0.00000	0.00069	0.00002	-0.00001	0.00061
202	C13	PY'	-0.00000	-0.00040	-0.00005	-0.00000	-0.00010
203	C13	PZ'	-0.00000	-0.00046	-0.00007	-0.00002	0.00145
204	C13	DXX	0.00000	-0.00002	-0.00000	-0.00000	0.00003
205	C13	DYY	0.00000	0.00006	0.00000	0.00000	0.00005
206	C13	DZZ	-0.00000	0.00004	-0.00001	-0.00000	0.00007
207	C13	DXY	-0.00000	-0.00003	-0.00001	-0.00000	0.00000
208	C13	DXZ	-0.00000	0.00001	-0.00000	-0.00000	-0.00001
209	C13	DYZ	0.00000	-0.00001	-0.00001	-0.00000	0.00000
210	C14	S1	0.00000	0.00000	0.00000	0.00000	0.01170
211	C14	S	0.00000	0.00001	0.00000	0.00000	0.00004
212	C14	PX	0.00000	0.00001	-0.00000	0.00000	-0.00031
213	C14	PY	-0.00000	-0.00001	-0.00000	-0.00000	0.00002
214	C14	PZ	0.00000	0.00001	0.00000	0.00000	-0.00004
215	C14	S'	-0.00000	0.00000	-0.00000	-0.00000	0.00723
216	C14	PX'	-0.00000	-0.00030	0.00000	-0.00000	0.00287
217	C14	PY'	0.00000	0.00001	0.00000	0.00000	-0.00017
218	C14	PZ'	-0.00000	0.00010	-0.00001	-0.00000	0.00150
219	C14	DXX	0.00000	-0.00000	-0.00000	-0.00000	-0.00055
220	C14	DYY	0.00000	0.00000	0.00000	0.00000	-0.00028
221	C14	DZZ	-0.00000	0.00001	0.00000	0.00000	-0.00032
222	C14	DXY	0.00000	-0.00000	-0.00000	-0.00000	0.00002
223	C14	DXZ	-0.00000	0.00000	0.00000	0.00000	0.00002
224	C14	DYZ	0.00000	0.00000	0.00000	0.00000	0.00001
225	C15	S1	0.00000	0.00001	-0.00000	-0.00000	-0.00017

226	C15	S	0.00000	0.00003	0.00000	0.00000	-0.00019
227	C15	PX	-0.00000	-0.00003	0.00000	-0.00000	-0.00002
228	C15	PY	-0.00000	-0.00001	-0.00000	-0.00000	-0.00001
229	C15	PZ	-0.00000	-0.00000	0.00000	-0.00000	0.00002
230	C15	S'	-0.00001	-0.00017	0.00002	-0.00001	0.00282
231	C15	PX'	0.00000	0.00082	-0.00000	-0.00000	0.00066
232	C15	PY'	0.00000	0.00005	0.00001	0.00000	0.00013
233	C15	PZ'	0.00000	0.00031	0.00001	0.00001	-0.00165
234	C15	DXX	-0.00000	0.00005	-0.00000	-0.00000	-0.00000
235	C15	DYY	0.00000	0.00002	0.00000	0.00000	-0.00007
236	C15	DZZ	-0.00000	-0.00002	-0.00000	-0.00000	-0.00000
237	C15	DXY	0.00000	0.00001	0.00000	0.00000	-0.00000
238	C15	DXZ	-0.00000	-0.00002	0.00000	0.00000	0.00001
239	C15	DYZ	-0.00000	-0.00001	-0.00000	-0.00000	-0.00001
240	C16	S1	0.00000	-0.00000	0.00000	0.00000	0.01288
241	C16	S	0.00000	0.00000	0.00000	0.00000	0.00012
242	C16	PX	-0.00000	0.00001	-0.00000	-0.00000	0.00021
243	C16	PY	0.00000	-0.00000	0.00000	0.00000	-0.00001
244	C16	PZ	-0.00000	0.00001	0.00000	0.00000	0.00029
245	C16	S'	-0.00000	-0.00023	-0.00008	-0.00001	0.00696
246	C16	PX'	0.00000	-0.00002	0.00004	0.00001	-0.00260
247	C16	PY'	-0.00000	0.00011	0.00001	-0.00000	0.00022
248	C16	PZ'	0.00000	-0.00037	-0.00001	-0.00000	-0.00138
249	C16	DXX	0.00000	-0.00000	0.00000	0.00000	-0.00040
250	C16	DYY	0.00000	0.00001	0.00000	0.00000	-0.00028
251	C16	DZZ	0.00000	0.00001	0.00000	0.00000	-0.00048
252	C16	DXY	-0.00000	-0.00002	-0.00000	-0.00000	0.00001
253	C16	DXZ	0.00000	0.00001	0.00000	0.00000	-0.00012
254	C16	DYZ	0.00000	0.00000	-0.00000	-0.00000	0.00002
255	C17	S1	0.00000	-0.00002	-0.00000	-0.00000	0.99269
256	C17	S	0.00000	-0.00002	-0.00000	-0.00000	0.04964
257	C17	PX	-0.00000	-0.00000	0.00000	0.00000	0.00019
258	C17	PY	0.00000	-0.00001	-0.00000	-0.00000	0.00010
259	C17	PZ	0.00000	0.00000	-0.00000	0.00000	-0.00037
260	C17	S'	-0.00002	0.00035	0.00006	0.00002	-0.01805
261	C17	PX'	-0.00001	-0.00018	0.00002	0.00000	0.00118
262	C17	PY'	-0.00000	0.00006	0.00000	0.00000	-0.00007
263	C17	PZ'	0.00001	-0.00030	0.00001	-0.00000	-0.00176
264	C17	DXX	0.00000	0.00000	-0.00000	-0.00000	-0.00901

265	C17	DYY	0.00000	-0.00000	-0.00000	-0.00000	-0.00934
266	C17	DZZ	0.00000	-0.00000	0.00000	0.00000	-0.00893
267	C17	DXY	0.00000	-0.00000	-0.00000	0.00000	0.00000
268	C17	DXZ	0.00000	-0.00001	-0.00000	-0.00000	-0.00004
269	C17	DYZ	0.00000	-0.00000	-0.00000	-0.00000	-0.00006
270	C18	S1	-0.00000	-0.00002	0.00000	0.00000	0.00017
271	C18	S	-0.00000	-0.00004	0.00000	-0.00000	0.00011
272	C18	PX	0.00000	0.00001	0.00000	0.00000	-0.00000
273	C18	PY	0.00000	-0.00000	-0.00000	-0.00000	-0.00001
274	C18	PZ	0.00000	-0.00000	0.00000	0.00000	0.00003
275	C18	S'	0.00001	0.00050	-0.00000	0.00001	-0.00325
276	C18	PX'	0.00000	0.00008	-0.00000	0.00000	-0.00199
277	C18	PY'	-0.00000	-0.00007	-0.00000	-0.00000	0.00004
278	C18	PZ'	0.00000	0.00055	-0.00001	-0.00000	-0.00003
279	C18	DXX	-0.00000	-0.00000	0.00000	0.00000	0.00006
280	C18	DYY	-0.00000	-0.00002	0.00000	0.00000	0.00005
281	C18	DZZ	0.00000	0.00001	0.00000	0.00000	0.00005
282	C18	DXY	-0.00000	-0.00000	0.00000	-0.00000	0.00000
283	C18	DXZ	0.00000	0.00002	-0.00000	0.00000	-0.00003
284	C18	DYZ	-0.00000	-0.00001	-0.00000	-0.00000	0.00000
285	H1	S	-0.00000	-0.00007	-0.00000	-0.00000	0.00005
286	H1	S'	0.00000	0.00030	-0.00001	-0.00000	0.00045
287	H11	S	-0.00000	0.00002	-0.00000	-0.00000	-0.00002
288	H11	S'	-0.00000	0.00001	-0.00001	-0.00000	0.00025
289	H13	S	-0.00000	-0.00002	-0.00000	0.00000	-0.00006
290	H13	S'	0.00000	-0.00003	-0.00000	0.00000	-0.00040
291	H14	S	-0.00000	-0.00001	-0.00000	-0.00000	-0.00000
292	H14	S'	-0.00000	-0.00002	-0.00000	-0.00000	0.00036
293	C19	S1	0.00000	0.00000	0.00000	0.00000	0.00748
294	C19	S	0.00000	0.00000	0.00000	0.00000	0.00004
295	C19	PX	-0.00000	0.00000	-0.00000	-0.00000	0.00015
296	C19	PY	0.00000	0.00000	0.00000	0.00000	0.00004
297	C19	PZ	0.00000	0.00001	0.00000	0.00000	-0.00029
298	C19	S'	-0.00006	0.00030	0.00005	0.00001	0.01074
299	C19	PX'	-0.00002	0.00023	0.00002	0.00001	0.00083
300	C19	PY'	-0.00001	0.00006	0.00001	0.00000	0.00070
301	C19	PZ'	0.00005	-0.00048	-0.00006	-0.00002	-0.00260
302	C19	DXX	0.00000	-0.00000	0.00000	0.00000	-0.00045
303	C19	DYY	0.00000	0.00000	0.00000	0.00000	-0.00021

304	C19	DZZ	-0.00000	0.00000	0.00000	-0.00000	-0.00077
305	C19	DXY	-0.00000	-0.00000	0.00000	0.00000	-0.00004
306	C19	DXZ	0.00000	0.00000	-0.00000	0.00000	0.00025
307	C19	DYZ	0.00000	-0.00000	-0.00000	-0.00000	0.00011
308	C20	S1	0.00000	-0.00000	-0.00000	0.00000	0.00028
309	C20	S	0.00000	0.00000	0.00000	0.00000	0.00027
310	C20	PX	0.00000	0.00000	-0.00000	0.00000	-0.00009
311	C20	PY	0.00000	-0.00000	-0.00000	-0.00000	-0.00006
312	C20	PZ	-0.00000	0.00000	-0.00000	-0.00000	0.00026
313	C20	S'	0.00002	-0.00037	-0.00005	-0.00002	-0.00350
314	C20	PX'	-0.00002	0.00015	0.00002	0.00000	0.00270
315	C20	PY'	-0.00002	0.00013	0.00002	0.00000	0.00153
316	C20	PZ'	0.00007	-0.00044	-0.00006	-0.00001	-0.00694
317	C20	DXX	0.00000	-0.00000	-0.00000	0.00000	0.00003
318	C20	DYY	-0.00000	-0.00000	-0.00000	0.00000	0.00003
319	C20	DZZ	0.00000	-0.00000	-0.00000	-0.00000	0.00020
320	C20	DXY	-0.00000	-0.00000	-0.00000	-0.00000	0.00002
321	C20	DXZ	-0.00000	0.00000	0.00000	0.00000	-0.00005
322	C20	DYZ	0.00000	0.00000	0.00000	0.00000	-0.00007
323	C21	S1	0.00000	0.00654	-0.00001	-0.00000	0.00000
324	C21	S	0.00000	-0.00001	-0.00001	-0.00000	0.00001
325	C21	PX	-0.00000	-0.00017	-0.00000	-0.00000	-0.00001
326	C21	PY	0.00000	0.00027	-0.00001	-0.00000	0.00001
327	C21	PZ	0.00001	-0.00002	-0.00001	0.00000	-0.00000
328	C21	S'	-0.00018	0.00963	0.00029	0.00016	-0.00138
329	C21	PX'	0.00002	-0.00077	-0.00041	-0.00018	0.00090
330	C21	PY'	-0.00004	0.00120	0.00063	0.00030	-0.00138
331	C21	PZ'	-0.00003	-0.00119	-0.00032	-0.00012	0.00039
332	C21	DXX	0.00000	-0.00036	-0.00001	-0.00000	0.00000
333	C21	DYY	0.00000	-0.00070	-0.00000	-0.00000	0.00000
334	C21	DZZ	0.00000	-0.00031	-0.00001	0.00000	0.00000
335	C21	DXY	0.00000	0.00030	0.00000	0.00000	-0.00000
336	C21	DXZ	0.00000	-0.00012	-0.00000	0.00000	0.00000
337	C21	DYZ	-0.00000	0.00007	0.00001	0.00000	-0.00000
338	C22	S1	0.00000	0.00024	0.00000	0.00000	0.00001
339	C22	S	0.00001	0.00028	0.00001	0.00001	0.00001
340	C22	PX	0.00000	0.00012	0.00000	0.00000	0.00000
341	C22	PY	-0.00000	-0.00019	-0.00000	0.00000	-0.00000
342	C22	PZ	-0.00000	0.00009	-0.00000	0.00000	-0.00000

343	C22	S'	-0.00017	-0.00320	-0.00069	-0.00033	0.00022
344	C22	PX'	0.00010	-0.00294	-0.00026	-0.00012	0.00104
345	C22	PY'	-0.00018	0.00474	0.00042	0.00019	-0.00181
346	C22	PZ'	0.00015	-0.00182	-0.00015	-0.00008	0.00107
347	C22	DXX	0.00000	0.00006	0.00000	0.00000	0.00001
348	C22	DYY	-0.00000	0.00012	0.00000	-0.00000	-0.00000
349	C22	DZZ	-0.00000	0.00008	0.00000	0.00000	-0.00000
350	C22	DXY	0.00000	-0.00006	0.00000	0.00000	0.00000
351	C22	DXZ	-0.00000	0.00007	0.00000	-0.00000	-0.00001
352	C22	DYZ	0.00000	-0.00010	0.00000	0.00000	0.00001
353	C23	S1	0.00000	-0.00001	0.00686	0.00025	-0.00000
354	C23	S	0.00003	-0.00001	0.00002	0.00026	-0.00000
355	C23	PX	-0.00002	-0.00001	-0.00032	-0.00022	0.00000
356	C23	PY	0.00001	-0.00001	0.00011	0.00006	-0.00000
357	C23	PZ	-0.00000	0.00001	0.00008	0.00012	0.00000
358	C23	S'	-0.00167	-0.00046	0.00845	-0.00342	0.00004
359	C23	PX'	0.00176	0.00087	0.00033	0.00585	-0.00011
360	C23	PY'	-0.00068	-0.00022	-0.00009	-0.00214	0.00004
361	C23	PZ'	-0.00062	-0.00049	0.00016	-0.00254	0.00005
362	C23	DXX	0.00001	-0.00000	-0.00076	0.00016	-0.00000
363	C23	DYY	-0.00000	-0.00000	-0.00023	0.00001	-0.00000
364	C23	DZZ	0.00001	-0.00001	-0.00037	0.00006	-0.00000
365	C23	DXY	0.00000	-0.00000	0.00021	-0.00003	-0.00000
366	C23	DXZ	-0.00000	-0.00000	0.00016	-0.00009	0.00000
367	C23	DYZ	-0.00000	-0.00000	-0.00005	0.00001	-0.00000
368	C24	S1	-0.00003	0.00000	0.00023	0.00671	-0.00000
369	C24	S	-0.00009	-0.00000	0.00025	-0.00003	0.00000
370	C24	PX	0.00000	-0.00000	0.00020	0.00029	-0.00000
371	C24	PY	-0.00001	0.00000	-0.00008	-0.00017	-0.00000
372	C24	PZ	0.00002	-0.00000	-0.00013	-0.00008	0.00000
373	C24	S'	0.00125	0.00063	-0.00165	0.01017	-0.00010
374	C24	PX'	0.00175	0.00075	-0.00365	0.00191	-0.00007
375	C24	PY'	-0.00050	-0.00035	0.00143	-0.00031	0.00003
376	C24	PZ'	-0.00124	-0.00032	0.00169	-0.00147	0.00003
377	C24	DXX	-0.00003	0.00000	0.00014	-0.00069	-0.00000
378	C24	DYY	-0.00003	0.00000	0.00004	-0.00035	-0.00000
379	C24	DZZ	-0.00004	0.00000	0.00007	-0.00037	-0.00000
380	C24	DXY	-0.00003	-0.00000	-0.00007	0.00028	-0.00000
381	C24	DXZ	0.00001	-0.00000	-0.00008	0.00020	0.00000

382	C24	DYZ	-0.00001	0.00000	0.00004	-0.00003	-0.00000
383	C25	S1	0.00000	0.00000	0.00000	0.00000	0.00005
384	C25	S	-0.00000	0.00000	0.00000	0.00000	0.00012
385	C25	PX	0.00000	-0.00000	0.00000	0.00000	0.00003
386	C25	PY	0.00000	0.00000	0.00000	0.00000	0.00000
387	C25	PZ	-0.00000	0.00000	0.00000	-0.00000	-0.00005
388	C25	S'	0.00003	-0.00018	-0.00003	-0.00000	-0.00345
389	C25	PX'	-0.00002	0.00008	0.00001	0.00000	0.00093
390	C25	PY'	-0.00001	0.00001	0.00000	0.00000	0.00116
391	C25	PZ'	0.00004	-0.00019	-0.00003	-0.00000	-0.00312
392	C25	DXX	0.00000	0.00000	0.00000	0.00000	-0.00001
393	C25	DYY	0.00000	-0.00000	-0.00000	0.00000	-0.00002
394	C25	DZZ	0.00000	-0.00000	-0.00000	-0.00000	-0.00012
395	C25	DXY	-0.00000	-0.00000	0.00000	-0.00000	-0.00001
396	C25	DXZ	-0.00000	0.00000	0.00000	0.00000	0.00005
397	C25	DYZ	-0.00000	0.00000	0.00000	0.00000	0.00003
398	C26	S1	0.00000	-0.00000	-0.00000	0.00000	0.00000
399	C26	S	0.00000	-0.00000	-0.00000	0.00000	-0.00000
400	C26	PX	-0.00000	-0.00000	-0.00000	-0.00000	0.00000
401	C26	PY	0.00000	0.00000	0.00000	0.00000	-0.00001
402	C26	PZ	0.00000	0.00000	-0.00000	0.00000	0.00001
403	C26	S'	0.00000	-0.00000	-0.00000	0.00000	-0.00009
404	C26	PX'	0.00000	-0.00000	-0.00000	-0.00000	0.00001
405	C26	PY'	0.00000	0.00000	0.00000	-0.00000	-0.00000
406	C26	PZ'	-0.00000	0.00000	0.00000	0.00000	-0.00003
407	C26	DXX	0.00000	-0.00000	-0.00000	0.00000	0.00000
408	C26	DYY	0.00000	-0.00000	-0.00000	0.00000	0.00000
409	C26	DZZ	-0.00000	0.00000	0.00000	-0.00000	0.00001
410	C26	DXY	0.00000	-0.00000	-0.00000	-0.00000	0.00000
411	C26	DXZ	0.00000	-0.00000	-0.00000	0.00000	-0.00000
412	C26	DYZ	0.00000	0.00000	0.00000	0.00000	-0.00000
413	C27	S1	0.00000	-0.00000	-0.00000	0.00000	0.00000
414	C27	S	0.00000	-0.00000	-0.00000	0.00000	-0.00002
415	C27	PX	0.00000	0.00000	-0.00000	0.00000	0.00001
416	C27	PY	-0.00000	-0.00000	-0.00000	-0.00000	-0.00002
417	C27	PZ	-0.00000	0.00000	0.00000	0.00000	-0.00001
418	C27	S'	-0.00013	0.00020	0.00003	-0.00001	0.00099
419	C27	PX'	0.00004	-0.00008	-0.00001	0.00000	-0.00097
420	C27	PY'	-0.00006	0.00012	0.00002	-0.00000	0.00159

421	C27	PZ'	-0.00006	0.00009	0.00002	-0.00001	0.00119
422	C27	DXX	0.00000	-0.00000	-0.00000	0.00000	0.00000
423	C27	DYY	-0.00000	0.00000	0.00000	-0.00000	0.00001
424	C27	DZZ	-0.00000	0.00000	0.00000	-0.00000	0.00001
425	C27	DXY	0.00000	-0.00000	-0.00000	0.00000	-0.00001
426	C27	DXZ	0.00000	-0.00000	-0.00000	0.00000	-0.00000
427	C27	DYZ	-0.00000	-0.00000	-0.00000	-0.00000	0.00001
428	C28	S1	0.00000	0.00000	0.00000	0.00000	0.00001
429	C28	S	0.00000	0.00000	0.00000	0.00000	0.00002
430	C28	PX	-0.00000	0.00000	0.00000	-0.00000	-0.00000
431	C28	PY	0.00000	-0.00000	-0.00000	0.00000	-0.00001
432	C28	PZ	-0.00000	-0.00000	-0.00000	-0.00000	0.00002
433	C28	S'	0.00001	-0.00007	-0.00001	-0.00000	-0.00038
434	C28	PX'	-0.00000	0.00001	0.00000	0.00000	0.00005
435	C28	PY'	0.00000	-0.00004	-0.00000	-0.00000	-0.00038
436	C28	PZ'	-0.00000	0.00000	0.00000	0.00000	0.00020
437	C28	DXX	0.00000	0.00000	0.00000	0.00000	0.00002
438	C28	DYY	0.00000	-0.00000	-0.00000	-0.00000	-0.00001
439	C28	DZZ	-0.00000	0.00000	0.00000	0.00000	0.00002
440	C28	DXY	-0.00000	0.00000	0.00000	0.00000	0.00001
441	C28	DXZ	0.00000	0.00000	0.00000	0.00000	-0.00001
442	C28	DYZ	-0.00000	0.00000	0.00000	0.00000	0.00001
443	C29	S1	0.00000	-0.00000	-0.00000	0.00000	0.00001
444	C29	S	0.00000	-0.00000	0.00000	0.00000	0.00003
445	C29	PX	-0.00000	-0.00000	-0.00000	-0.00000	-0.00001
446	C29	PY	0.00000	-0.00000	-0.00000	0.00000	0.00000
447	C29	PZ	0.00000	0.00000	0.00000	0.00000	0.00001
448	C29	S'	-0.00000	0.00002	0.00000	-0.00000	0.00010
449	C29	PX'	0.00000	-0.00000	-0.00000	0.00000	0.00003
450	C29	PY'	-0.00000	0.00000	0.00000	-0.00000	-0.00007
451	C29	PZ'	-0.00000	0.00000	0.00000	-0.00000	-0.00003
452	C29	DXX	0.00000	-0.00000	-0.00000	0.00000	0.00001
453	C29	DYY	0.00000	-0.00000	-0.00000	-0.00000	0.00000
454	C29	DZZ	0.00000	0.00000	0.00000	0.00000	-0.00000
455	C29	DXY	0.00000	0.00000	0.00000	0.00000	0.00001
456	C29	DXZ	-0.00000	0.00000	0.00000	0.00000	0.00001
457	C29	DYZ	0.00000	-0.00000	-0.00000	0.00000	-0.00000
458	C30	S1	0.00000	0.00000	0.00000	0.00000	0.00000
459	C30	S	0.00000	0.00000	-0.00000	0.00000	0.00001

460	C30	PX	-0.00000	0.00000	0.00000	0.00000	0.00000
461	C30	PY	0.00000	-0.00000	-0.00000	-0.00000	-0.00002
462	C30	PZ	0.00000	-0.00000	-0.00000	-0.00000	0.00001
463	C30	S'	-0.00001	0.00001	0.00000	-0.00000	0.00042
464	C30	PX'	-0.00000	0.00000	-0.00000	0.00000	0.00004
465	C30	PY'	0.00001	-0.00001	-0.00000	0.00000	-0.00027
466	C30	PZ'	-0.00001	0.00001	0.00000	-0.00000	0.00011
467	C30	DXX	0.00000	-0.00000	-0.00000	0.00000	-0.00000
468	C30	DYY	-0.00000	0.00000	0.00000	-0.00000	0.00001
469	C30	DZZ	0.00000	-0.00000	-0.00000	0.00000	-0.00000
470	C30	DXY	0.00000	0.00000	0.00000	0.00000	0.00001
471	C30	DXZ	0.00000	0.00000	0.00000	0.00000	0.00001
472	C30	DYZ	0.00000	-0.00000	-0.00000	0.00000	-0.00001
473	H15	S	0.00000	-0.00000	-0.00000	-0.00000	-0.00001
474	H15	S'	0.00000	-0.00001	-0.00000	-0.00000	-0.00016
475	H16	S	-0.00000	0.00000	0.00000	-0.00000	-0.00001
476	H16	S'	-0.00000	0.00000	0.00000	-0.00000	-0.00006
477	H17	S	-0.00000	-0.00000	-0.00000	-0.00000	0.00000
478	H17	S'	-0.00000	0.00000	0.00000	-0.00000	0.00009
479	H18	S	-0.00000	0.00000	0.00000	0.00000	0.00000
480	H18	S'	-0.00000	0.00000	0.00000	-0.00000	-0.00000
481	C31	S1	0.00219	0.00002	-0.00160	0.99263	0.00000
482	C31	S	0.00024	-0.00000	-0.00000	0.04971	0.00000
483	C31	PX	-0.00001	-0.00000	-0.00005	0.00035	-0.00000
484	C31	PY	0.00001	-0.00000	0.00001	-0.00014	-0.00000
485	C31	PZ	-0.00002	0.00000	0.00003	-0.00016	0.00000
486	C31	S'	-0.00271	0.00027	-0.00221	-0.01859	-0.00004
487	C31	PX'	0.00257	0.00038	-0.00137	0.00173	-0.00002
488	C31	PY'	-0.00195	-0.00019	0.00100	-0.00164	0.00001
489	C31	PZ'	-0.00003	-0.00015	0.00016	0.00018	0.00001
490	C31	DXX	0.00004	0.00000	-0.00006	-0.00908	-0.00000
491	C31	DYY	0.00003	-0.00000	-0.00001	-0.00923	-0.00000
492	C31	DZZ	0.00003	-0.00000	-0.00001	-0.00907	0.00000
493	C31	DXY	-0.00001	-0.00000	0.00005	-0.00017	0.00000
494	C31	DXZ	-0.00000	-0.00000	0.00003	-0.00016	-0.00000
495	C31	DYZ	0.00001	0.00000	-0.00001	-0.00009	0.00000
496	C32	S1	0.01324	-0.00000	0.00000	-0.00024	-0.00000
497	C32	S	0.00016	-0.00001	-0.00000	-0.00018	0.00000
498	C32	PX	-0.00017	-0.00000	0.00001	-0.00002	0.00000

499 C32	PY	-0.00006	0.00000	-0.00001	0.00001	-0.00000
500 C32	PZ	0.00034	-0.00000	0.00000	0.00001	0.00000
501 C32	S'	0.00571	0.00014	0.00020	0.00250	-0.00002
502 C32	PX'	0.00183	0.00005	0.00008	0.00152	-0.00001
503 C32	PY'	-0.00026	-0.00003	-0.00005	-0.00108	0.00001
504 C32	PZ'	-0.00206	-0.00003	-0.00004	-0.00023	-0.00000
505 C32	DXX	-0.00038	-0.00000	0.00000	-0.00002	0.00000
506 C32	DYY	-0.00035	-0.00000	0.00000	-0.00004	-0.00000
507 C32	DZZ	-0.00055	0.00000	0.00000	-0.00002	-0.00000
508 C32	DXY	0.00001	-0.00000	-0.00000	-0.00003	0.00000
509 C32	DXZ	0.00011	0.00000	-0.00000	-0.00002	-0.00000
510 C32	DYZ	0.00008	-0.00000	0.00000	-0.00001	0.00000
511 C33	S1	-0.00017	-0.00000	-0.00002	0.01411	0.00000
512 C33	S	-0.00017	-0.00001	-0.00000	0.00020	0.00000
513 C33	PX	0.00001	-0.00000	0.00002	-0.00012	0.00000
514 C33	PY	-0.00001	0.00000	-0.00001	-0.00009	-0.00000
515 C33	PZ	0.00001	-0.00000	-0.00001	0.00032	-0.00000
516 C33	S'	0.00258	0.00019	0.00016	0.00621	-0.00000
517 C33	PX'	-0.00104	-0.00005	0.00011	0.00012	-0.00000
518 C33	PY'	0.00132	0.00008	-0.00009	0.00142	-0.00000
519 C33	PZ'	-0.00087	-0.00009	-0.00001	-0.00253	0.00000
520 C33	DXX	-0.00003	-0.00000	0.00001	-0.00039	0.00000
521 C33	DYY	-0.00003	-0.00000	0.00000	-0.00033	0.00000
522 C33	DZZ	-0.00001	0.00000	0.00001	-0.00055	0.00000
523 C33	DXY	-0.00002	-0.00000	0.00000	0.00001	0.00000
524 C33	DXZ	-0.00001	0.00000	-0.00001	0.00012	-0.00000
525 C33	DYZ	-0.00003	-0.00000	0.00001	0.00006	0.00000
526 C34	S1	0.01216	0.00000	-0.00002	0.01383	-0.00000
527 C34	S	-0.00002	-0.00001	0.00004	0.00006	-0.00000
528 C34	PX	0.00026	0.00000	-0.00001	-0.00010	-0.00000
529 C34	PY	-0.00012	0.00000	0.00000	0.00022	0.00000
530 C34	PZ	-0.00015	-0.00001	0.00001	-0.00024	-0.00000
531 C34	S'	0.00921	0.00008	-0.00051	0.00877	0.00001
532 C34	PX'	-0.00254	-0.00007	0.00009	-0.00027	-0.00000
533 C34	PY'	0.00037	-0.00005	0.00008	-0.00161	-0.00000
534 C34	PZ'	0.00288	0.00017	-0.00029	0.00329	0.00000
535 C34	DXX	-0.00045	0.00000	-0.00000	-0.00036	0.00000
536 C34	DYY	-0.00036	0.00000	0.00001	-0.00042	0.00000
537 C34	DZZ	-0.00035	0.00001	-0.00000	-0.00041	0.00000

538	C34	DXY	0.00009	-0.00000	0.00001	0.00006	-0.00000
539	C34	DXZ	0.00006	-0.00000	0.00001	-0.00005	0.00000
540	C34	DYZ	-0.00002	-0.00000	0.00000	0.00011	-0.00000
541	C35	S1	0.99265	-0.00000	0.00004	-0.00229	-0.00000
542	C35	S	0.04974	0.00001	0.00000	0.00001	0.00000
543	C35	PX	-0.00010	-0.00000	0.00001	0.00001	0.00000
544	C35	PY	0.00028	-0.00000	-0.00001	-0.00001	0.00000
545	C35	PZ	-0.00032	0.00000	0.00001	-0.00002	-0.00000
546	C35	S'	-0.01925	-0.00035	0.00027	-0.00264	0.00005
547	C35	PX'	-0.00189	0.00006	-0.00001	-0.00183	-0.00003
548	C35	PY'	0.00143	-0.00003	0.00015	0.00208	0.00002
549	C35	PZ'	0.00011	-0.00001	-0.00023	-0.00100	-0.00000
550	C35	DXX	-0.00920	-0.00000	0.00000	0.00007	0.00000
551	C35	DYY	-0.00920	-0.00000	0.00000	0.00008	0.00000
552	C35	DZZ	-0.00899	-0.00000	-0.00000	0.00007	-0.00000
553	C35	DXY	-0.00011	0.00000	-0.00000	-0.00000	-0.00000
554	C35	DXZ	-0.00008	-0.00000	0.00000	0.00002	0.00000
555	C35	DYZ	-0.00019	0.00000	-0.00000	-0.00000	0.00000
556	C36	S1	0.00018	0.00000	0.00001	0.00021	-0.00000
557	C36	S	0.00004	0.00000	0.00004	0.00006	-0.00000
558	C36	PX	0.00000	-0.00000	0.00000	-0.00001	-0.00000
559	C36	PY	-0.00000	0.00000	0.00000	0.00001	0.00000
560	C36	PZ	0.00000	0.00000	-0.00001	0.00000	-0.00000
561	C36	S'	-0.00261	-0.00013	-0.00014	-0.00282	0.00001
562	C36	PX'	-0.00091	-0.00003	-0.00007	-0.00045	-0.00000
563	C36	PY'	-0.00004	-0.00001	-0.00004	-0.00052	0.00000
564	C36	PZ'	0.00131	0.00006	0.00017	0.00150	-0.00001
565	C36	DXX	0.00003	0.00000	0.00000	0.00006	-0.00000
566	C36	DYY	0.00005	-0.00000	0.00001	0.00003	0.00000
567	C36	DZZ	0.00007	0.00000	0.00000	0.00005	0.00000
568	C36	DXY	-0.00001	0.00000	0.00001	-0.00002	-0.00000
569	C36	DXZ	0.00001	-0.00000	0.00001	-0.00004	0.00000
570	C36	DYZ	-0.00004	0.00000	0.00000	0.00001	-0.00000
571	H4	S	0.00006	0.00000	-0.00001	-0.00002	-0.00000
572	H4	S'	0.00053	0.00004	-0.00008	0.00033	-0.00000
573	H19	S	0.00000	-0.00000	-0.00001	0.00001	-0.00000
574	H19	S'	0.00046	0.00012	-0.00011	0.00044	0.00000
575	H21	S	-0.00005	-0.00000	-0.00001	-0.00005	0.00000
576	H21	S'	-0.00028	-0.00001	-0.00007	-0.00029	0.00000

577	H22	S	-0.00003	0.00000	0.00000	0.00006	-0.00000
578	H22	S'	0.00031	0.00001	0.00002	0.00053	-0.00000
579	C37	S1	0.00000	0.00006	0.00000	0.00000	-0.00000
580	C37	S	0.00002	0.00008	-0.00000	0.00000	0.00001
581	C37	PX	-0.00000	-0.00004	-0.00000	-0.00000	-0.00003
582	C37	PY	0.00001	0.00005	0.00000	0.00000	0.00003
583	C37	PZ	-0.00000	0.00002	0.00000	0.00000	-0.00001
584	C37	S'	-0.00022	-0.00258	-0.00015	-0.00013	-0.00007
585	C37	PX'	0.00024	-0.00032	0.00000	0.00002	0.00112
586	C37	PY'	-0.00036	0.00127	0.00004	-0.00000	-0.00182
587	C37	PZ'	0.00016	-0.00248	-0.00013	-0.00004	0.00128
588	C37	DXX	0.00000	-0.00003	-0.00000	-0.00000	-0.00000
589	C37	DYY	0.00000	-0.00006	-0.00000	-0.00000	0.00001
590	C37	DZZ	-0.00000	-0.00004	-0.00000	-0.00000	-0.00001
591	C37	DXY	0.00000	0.00005	0.00000	0.00000	0.00000
592	C37	DXZ	0.00000	-0.00002	-0.00000	0.00000	0.00001
593	C37	DYZ	-0.00000	0.00005	0.00000	0.00000	-0.00001
594	C38	S1	0.00001	0.00000	-0.00000	0.00000	-0.00002
595	C38	S	0.00002	0.00000	-0.00000	0.00000	-0.00005
596	C38	PX	0.00000	0.00000	-0.00000	0.00000	0.00001
597	C38	PY	-0.00000	-0.00000	0.00000	-0.00000	-0.00001
598	C38	PZ	0.00000	0.00000	0.00000	0.00000	-0.00000
599	C38	S'	-0.00013	-0.00002	0.00003	-0.00001	0.00113
600	C38	PX'	-0.00004	-0.00002	0.00000	-0.00000	0.00022
601	C38	PY'	0.00007	0.00005	-0.00001	0.00001	-0.00049
602	C38	PZ'	-0.00003	-0.00006	0.00000	-0.00000	0.00050
603	C38	DXX	0.00000	0.00000	-0.00000	0.00000	-0.00001
604	C38	DYY	0.00000	0.00000	-0.00000	0.00000	0.00000
605	C38	DZZ	-0.00001	0.00001	0.00000	-0.00000	-0.00000
606	C38	DXY	0.00000	0.00000	0.00000	0.00000	-0.00001
607	C38	DXZ	0.00000	0.00000	-0.00000	0.00000	0.00000
608	C38	DYZ	0.00000	-0.00000	0.00000	0.00000	-0.00000
609	C39	S1	0.00003	-0.00000	-0.00000	0.00000	0.00000
610	C39	S	0.00003	-0.00007	-0.00000	0.00000	-0.00003
611	C39	PX	0.00000	0.00001	0.00000	0.00000	0.00000
612	C39	PY	0.00001	-0.00002	-0.00000	0.00000	-0.00000
613	C39	PZ	-0.00002	-0.00001	0.00000	-0.00000	0.00001
614	C39	S'	-0.00173	0.00160	0.00011	-0.00027	0.00044
615	C39	PX'	0.00055	0.00002	-0.00000	0.00007	0.00060

616	C39	PY'	-0.00024	-0.00057	-0.00001	-0.00003	-0.00008
617	C39	PZ'	-0.00109	0.00135	0.00002	-0.00016	-0.00166
618	C39	DXX	-0.00000	0.00001	0.00000	-0.00000	0.00000
619	C39	DYY	-0.00000	0.00001	0.00000	0.00000	0.00000
620	C39	DZZ	-0.00004	0.00001	0.00000	-0.00000	-0.00001
621	C39	DXY	0.00000	-0.00002	-0.00000	0.00000	-0.00001
622	C39	DXZ	0.00001	-0.00001	0.00000	0.00000	-0.00001
623	C39	DYZ	0.00000	-0.00001	-0.00000	-0.00000	0.00001
624	C40	S1	-0.00000	0.00001	0.00000	0.00000	0.00003
625	C40	S	-0.00000	0.00007	0.00001	0.00000	-0.00006
626	C40	PX	-0.00000	-0.00000	-0.00000	-0.00000	0.00006
627	C40	PY	0.00000	0.00001	0.00000	0.00000	-0.00005
628	C40	PZ	0.00001	-0.00002	-0.00000	0.00000	-0.00002
629	C40	S'	0.00020	-0.00159	-0.00010	-0.00001	0.00110
630	C40	PX'	-0.00008	0.00044	0.00002	-0.00000	-0.00122
631	C40	PY'	0.00010	-0.00040	-0.00002	0.00000	0.00128
632	C40	PZ'	0.00001	-0.00041	-0.00002	-0.00001	0.00050
633	C40	DXX	0.00000	0.00000	0.00000	0.00000	0.00003
634	C40	DYY	0.00000	-0.00000	-0.00000	0.00000	0.00003
635	C40	DZZ	0.00000	-0.00002	-0.00000	-0.00000	0.00003
636	C40	DXY	0.00000	0.00001	0.00000	0.00000	-0.00003
637	C40	DXZ	-0.00000	0.00000	-0.00000	-0.00000	-0.00001
638	C40	DYZ	0.00000	0.00001	0.00000	0.00000	0.00000
639	C41	S1	-0.00000	0.00004	0.00000	0.00000	-0.00001
640	C41	S	-0.00001	-0.00002	-0.00000	-0.00000	0.00003
641	C41	PX	-0.00000	0.00000	-0.00000	-0.00000	-0.00002
642	C41	PY	-0.00000	-0.00001	-0.00000	-0.00000	0.00001
643	C41	PZ	0.00000	0.00001	0.00000	0.00000	0.00001
644	C41	S'	0.00030	0.00040	-0.00001	0.00006	-0.00081
645	C41	PX'	-0.00002	0.00010	0.00001	0.00000	0.00033
646	C41	PY'	0.00011	-0.00022	-0.00002	0.00001	0.00011
647	C41	PZ'	-0.00025	0.00028	0.00003	-0.00004	-0.00148
648	C41	DXX	0.00000	-0.00000	-0.00000	0.00000	0.00001
649	C41	DYY	-0.00000	0.00000	0.00000	-0.00000	-0.00001
650	C41	DZZ	0.00001	0.00000	-0.00000	0.00000	-0.00000
651	C41	DXY	0.00000	-0.00000	-0.00000	0.00000	0.00001
652	C41	DXZ	-0.00000	0.00001	0.00000	-0.00000	-0.00001
653	C41	DYZ	0.00000	-0.00001	-0.00000	0.00000	0.00002
654	C42	S1	0.00000	0.00001	0.00000	0.00000	0.00002

655	C42	S	-0.00000	0.00001	0.00000	0.00000	0.00004
656	C42	PX	-0.00000	0.00001	0.00000	-0.00000	-0.00001
657	C42	PY	-0.00000	-0.00001	0.00000	-0.00000	0.00001
658	C42	PZ	0.00001	-0.00001	-0.00000	0.00000	0.00000
659	C42	S'	0.00015	0.00043	-0.00003	0.00001	-0.00109
660	C42	PX'	-0.00001	0.00006	-0.00001	-0.00000	-0.00043
661	C42	PY'	-0.00001	-0.00011	0.00001	0.00001	0.00043
662	C42	PZ'	0.00005	0.00007	0.00001	0.00001	0.00035
663	C42	DXX	-0.00000	0.00001	0.00000	0.00000	0.00000
664	C42	DYY	-0.00000	0.00000	0.00000	-0.00000	-0.00000
665	C42	DZZ	0.00001	-0.00000	-0.00000	0.00000	0.00000
666	C42	DXY	0.00001	0.00000	0.00000	0.00000	0.00001
667	C42	DXZ	0.00000	-0.00000	-0.00000	0.00000	0.00000
668	C42	DYZ	-0.00001	0.00001	0.00000	-0.00000	-0.00000
669	H23	S	0.00000	-0.00001	-0.00000	0.00000	-0.00003
670	H23	S'	0.00003	-0.00014	-0.00000	0.00000	0.00116
671	H25	S	0.00000	-0.00001	-0.00000	-0.00000	-0.00002
672	H25	S'	-0.00003	-0.00000	-0.00001	-0.00001	-0.00017
673	H26	S	-0.00000	-0.00000	0.00000	-0.00000	0.00002
674	H26	S'	-0.00002	-0.00003	-0.00000	-0.00000	0.00019
675	C43	S1	0.00978	-0.00000	0.00001	-0.00009	-0.00000
676	C43	S	0.00006	-0.00000	0.00002	-0.00010	-0.00000
677	C43	PX	-0.00017	-0.00000	0.00000	0.00000	-0.00000
678	C43	PY	0.00025	0.00000	-0.00000	-0.00000	0.00000
679	C43	PZ	-0.00020	0.00000	-0.00000	0.00001	-0.00000
680	C43	S'	0.01192	-0.00093	-0.00014	0.00156	0.00038
681	C43	PX'	0.00083	0.00010	-0.00010	-0.00002	-0.00002
682	C43	PY'	0.00078	-0.00038	0.00019	0.00106	0.00012
683	C43	PZ'	-0.00251	0.00051	-0.00018	-0.00185	-0.00018
684	C43	DXX	-0.00039	-0.00000	0.00000	-0.00002	-0.00000
685	C43	DYY	-0.00054	-0.00000	0.00000	-0.00003	0.00000
686	C43	DZZ	-0.00058	-0.00000	0.00000	-0.00004	0.00000
687	C43	DXY	0.00019	-0.00000	0.00001	-0.00003	-0.00000
688	C43	DXZ	-0.00011	-0.00000	-0.00000	-0.00000	-0.00000
689	C43	DYZ	0.00030	0.00000	0.00000	-0.00000	-0.00000
690	C44	S1	0.00057	0.00000	-0.00000	-0.00002	0.00000
691	C44	S	0.00018	0.00002	-0.00000	0.00001	-0.00000
692	C44	PX	-0.00001	0.00000	-0.00000	-0.00001	0.00000
693	C44	PY	-0.00008	-0.00000	-0.00000	0.00000	-0.00000

694	C44	PZ	0.00016	-0.00000	0.00000	0.00001	0.00000
695	C44	S'	-0.00045	-0.00055	-0.00013	-0.00100	0.00031
696	C44	PX'	-0.00038	-0.00007	0.00004	-0.00001	0.00000
697	C44	PY'	0.00462	-0.00066	0.00003	0.00116	0.00028
698	C44	PZ'	-0.00790	0.00133	-0.00012	-0.00207	-0.00052
699	C44	DXX	0.00001	0.00000	-0.00000	-0.00001	0.00000
700	C44	DYY	0.00002	0.00000	0.00000	-0.00001	0.00000
701	C44	DZZ	0.00025	-0.00001	-0.00000	0.00001	0.00000
702	C44	DXY	0.00003	-0.00000	-0.00000	0.00000	-0.00000
703	C44	DXZ	-0.00007	0.00000	0.00000	-0.00001	0.00000
704	C44	DYZ	-0.00010	0.00000	-0.00000	-0.00000	-0.00000
705	C45	S1	0.00002	0.00002	0.00000	0.00000	0.00000
706	C45	S	0.00007	0.00003	-0.00000	0.00001	-0.00001
707	C45	PX	-0.00001	0.00001	0.00000	-0.00000	-0.00000
708	C45	PY	-0.00001	-0.00001	0.00000	-0.00000	0.00000
709	C45	PZ	0.00004	0.00000	-0.00000	0.00000	0.00000
710	C45	S'	-0.00568	0.00062	0.00004	-0.00107	-0.00010
711	C45	PX'	0.00116	-0.00072	-0.00002	0.00021	0.00041
712	C45	PY'	0.00101	-0.00020	-0.00002	0.00016	0.00027
713	C45	PZ'	-0.00454	0.00198	0.00009	-0.00076	-0.00144
714	C45	DXX	0.00001	0.00001	-0.00000	0.00000	-0.00000
715	C45	DYY	0.00000	0.00001	0.00000	0.00000	-0.00000
716	C45	DZZ	0.00004	0.00000	0.00000	0.00000	0.00000
717	C45	DXY	0.00000	0.00001	0.00000	0.00000	0.00000
718	C45	DXZ	-0.00001	0.00001	0.00000	0.00000	-0.00000
719	C45	DYZ	-0.00000	-0.00000	-0.00000	0.00000	-0.00000
720	C46	S1	-0.00005	-0.00001	0.00000	-0.00000	0.00000
721	C46	S	-0.00001	-0.00001	0.00000	0.00000	-0.00000
722	C46	PX	-0.00002	-0.00000	0.00000	-0.00000	0.00000
723	C46	PY	0.00002	0.00001	-0.00000	0.00000	-0.00000
724	C46	PZ	-0.00001	-0.00001	-0.00000	0.00000	0.00000
725	C46	S'	-0.00039	-0.00067	-0.00016	-0.00043	0.00078
726	C46	PX'	0.00154	-0.00025	-0.00002	0.00027	0.00018
727	C46	PY'	0.00254	-0.00077	0.00001	0.00052	0.00037
728	C46	PZ'	-0.00774	0.00192	0.00003	-0.00154	-0.00103
729	C46	DXX	-0.00001	-0.00000	0.00000	-0.00000	0.00000
730	C46	DYY	-0.00004	-0.00000	0.00000	-0.00000	0.00000
731	C46	DZZ	-0.00014	0.00000	-0.00000	-0.00001	0.00000
732	C46	DXY	-0.00001	-0.00000	0.00000	-0.00000	0.00000

733	C46	DXZ	0.00001	-0.00000	-0.00000	-0.00000	-0.00000
734	C46	DYZ	0.00006	-0.00000	0.00000	0.00000	0.00000
735	C47	S1	-0.00000	0.00001	0.00000	0.00000	0.00001
736	C47	S	-0.00000	0.00003	0.00000	0.00000	0.00001
737	C47	PX	-0.00000	0.00000	0.00000	-0.00000	0.00000
738	C47	PY	0.00000	-0.00000	-0.00000	-0.00000	-0.00000
739	C47	PZ	0.00000	0.00000	-0.00000	0.00000	0.00000
740	C47	S'	0.00063	-0.00064	-0.00010	0.00007	-0.00109
741	C47	PX'	0.00025	-0.00019	-0.00003	0.00003	0.00014
742	C47	PY'	-0.00020	0.00019	0.00003	-0.00002	-0.00008
743	C47	PZ'	-0.00064	0.00030	0.00007	-0.00009	-0.00056
744	C47	DXX	-0.00000	0.00001	0.00000	0.00000	0.00000
745	C47	DYY	0.00000	0.00001	0.00000	0.00000	0.00000
746	C47	DZZ	0.00000	-0.00000	-0.00000	-0.00000	-0.00001
747	C47	DXY	-0.00000	0.00000	0.00000	-0.00000	0.00000
748	C47	DXZ	-0.00000	0.00000	0.00000	-0.00000	0.00000
749	C47	DYZ	0.00000	0.00000	0.00000	0.00000	-0.00000
750	C48	S1	0.00000	0.00000	0.00000	-0.00000	-0.00000
751	C48	S	0.00000	-0.00000	-0.00000	0.00000	0.00002
752	C48	PX	-0.00000	-0.00000	0.00000	-0.00000	0.00000
753	C48	PY	0.00000	0.00000	-0.00000	0.00000	0.00001
754	C48	PZ	0.00000	-0.00000	-0.00000	0.00000	0.00000
755	C48	S'	-0.00027	0.00006	0.00002	-0.00004	-0.00159
756	C48	PX'	0.00034	-0.00024	-0.00005	0.00004	-0.00033
757	C48	PY'	-0.00036	0.00024	0.00005	-0.00005	0.00031
758	C48	PZ'	-0.00077	0.00063	0.00011	-0.00010	0.00080
759	C48	DXX	0.00000	-0.00000	-0.00000	-0.00000	0.00000
760	C48	DYY	0.00000	-0.00000	-0.00000	0.00000	-0.00000
761	C48	DZZ	-0.00000	0.00000	0.00000	-0.00000	-0.00000
762	C48	DXY	-0.00000	0.00000	0.00000	0.00000	0.00000
763	C48	DXZ	0.00000	0.00000	-0.00000	0.00000	0.00000
764	C48	DYZ	-0.00000	-0.00000	-0.00000	-0.00000	-0.00000
765	C49	S1	-0.00000	-0.00000	0.00000	-0.00000	-0.00001
766	C49	S	0.00000	-0.00000	-0.00000	0.00000	-0.00000
767	C49	PX	0.00000	-0.00000	0.00000	0.00000	0.00001
768	C49	PY	0.00000	-0.00000	-0.00000	0.00000	-0.00001
769	C49	PZ	-0.00000	-0.00000	0.00000	-0.00000	-0.00001
770	C49	S'	-0.00042	0.00045	0.00007	-0.00005	0.00132
771	C49	PX'	0.00015	-0.00020	-0.00003	0.00001	-0.00143

772	C49	PY'	-0.00022	0.00029	0.00004	-0.00002	0.00205
773	C49	PZ'	-0.00022	0.00032	0.00005	-0.00002	0.00220
774	C49	DXX	-0.00000	-0.00000	0.00000	-0.00000	-0.00000
775	C49	DYY	-0.00000	-0.00000	0.00000	-0.00000	-0.00001
776	C49	DZZ	-0.00000	0.00000	0.00000	0.00000	0.00000
777	C49	DXY	0.00000	-0.00000	-0.00000	0.00000	0.00000
778	C49	DXZ	0.00000	-0.00000	-0.00000	0.00000	-0.00000
779	C49	DYZ	-0.00000	0.00000	0.00000	-0.00000	0.00000
780	C50	S1	-0.00000	0.00000	0.00000	0.00000	-0.00001
781	C50	S	0.00000	0.00000	-0.00000	0.00000	0.00002
782	C50	PX	-0.00000	0.00000	0.00000	-0.00000	0.00000
783	C50	PY	0.00000	-0.00000	-0.00000	-0.00000	0.00000
784	C50	PZ	0.00000	-0.00000	-0.00000	0.00000	-0.00001
785	C50	S'	-0.00017	-0.00005	0.00001	-0.00003	-0.00214
786	C50	PX'	0.00027	-0.00030	-0.00005	0.00003	-0.00113
787	C50	PY'	-0.00034	0.00037	0.00006	-0.00004	0.00143
788	C50	PZ'	-0.00052	0.00052	0.00009	-0.00006	0.00209
789	C50	DXX	0.00000	0.00000	0.00000	0.00000	-0.00000
790	C50	DYY	0.00000	0.00000	-0.00000	0.00000	-0.00000
791	C50	DZZ	-0.00000	0.00000	0.00000	-0.00000	-0.00000
792	C50	DXY	-0.00000	0.00000	0.00000	0.00000	0.00000
793	C50	DXZ	0.00000	0.00000	-0.00000	0.00000	0.00000
794	C50	DYZ	-0.00000	-0.00000	0.00000	-0.00000	-0.00001

MO:			11	12	13	14	15
Eigenvalues:			-10.21394	-10.21140	-10.21023	-10.20956	-10.20901
(ev)			-277.93570	-277.86648	-277.83470	-277.81625	-277.80144
			A	A	A	A	A
1	C1	S1	-0.00003	0.99220	0.00264	0.00001	-0.00000
2	C1	S	0.00004	0.04992	-0.00003	-0.00001	-0.00001
3	C1	PX	0.00002	-0.00008	-0.00004	-0.00000	-0.00000
4	C1	PY	-0.00000	0.00005	0.00000	0.00001	0.00000
5	C1	PZ	0.00004	-0.00006	-0.00005	0.00001	-0.00000
6	C1	S'	-0.00075	-0.02027	0.00255	0.00015	0.00022
7	C1	PX'	-0.00107	-0.00140	0.00187	-0.00009	0.00007
8	C1	PY'	0.00053	-0.00035	-0.00086	0.00010	0.00007
9	C1	PZ'	-0.00140	-0.00283	0.00225	-0.00017	0.00016
10	C1	DXX	-0.00001	-0.00914	-0.00007	0.00000	-0.00000
11	C1	DYY	0.00001	-0.00895	-0.00002	0.00001	0.00000

12	C1	DZZ	-0.00002	-0.00906	-0.00005	-0.00000	-0.00000
13	C1	DXY	0.00000	-0.00013	-0.00003	-0.00000	-0.00000
14	C1	DXZ	-0.00001	0.00009	0.00001	-0.00000	0.00000
15	C1	DYZ	0.00000	0.00007	0.00001	0.00000	0.00000
16	C4	S1	0.03703	-0.00272	0.99129	0.00000	0.00001
17	C4	S	0.00180	-0.00032	0.04975	-0.00000	-0.00001
18	C4	PX	-0.00014	0.00003	0.00011	-0.00000	0.00000
19	C4	PY	0.00003	-0.00001	-0.00001	0.00000	0.00000
20	C4	PZ	-0.00017	0.00004	0.00004	-0.00000	0.00001
21	C4	S'	0.00181	0.00309	-0.01973	0.00015	0.00001
22	C4	PX'	0.00018	-0.00160	0.00121	0.00006	-0.00013
23	C4	PY'	0.00023	0.00040	-0.00044	-0.00001	0.00006
24	C4	PZ'	0.00068	-0.00219	0.00189	0.00011	-0.00013
25	C4	DXX	-0.00065	-0.00003	-0.00915	0.00000	-0.00000
26	C4	DYY	-0.00045	0.00002	-0.00900	-0.00000	0.00000
27	C4	DZZ	-0.00066	-0.00001	-0.00913	0.00000	-0.00000
28	C4	DXY	0.00008	-0.00002	-0.00014	-0.00000	-0.00000
29	C4	DXZ	-0.00021	0.00001	0.00009	0.00000	-0.00000
30	C4	DYZ	0.00000	0.00003	0.00008	-0.00000	0.00000
31	C2	S1	-0.00006	0.02059	0.00062	0.00001	0.00000
32	C2	S	-0.00020	0.00044	0.00017	0.00001	-0.00000
33	C2	PX	-0.00002	0.00022	-0.00003	-0.00000	0.00000
34	C2	PY	0.00002	-0.00029	-0.00001	0.00000	-0.00000
35	C2	PZ	0.00002	-0.00002	-0.00002	0.00000	0.00000
36	C2	S'	0.00322	0.00764	-0.00416	-0.00001	-0.00001
37	C2	PX'	0.00065	-0.00112	0.00016	-0.00002	-0.00001
38	C2	PY'	0.00047	0.00310	-0.00183	-0.00002	-0.00002
39	C2	PZ'	0.00136	0.00149	-0.00166	-0.00003	-0.00002
40	C2	DXX	-0.00001	-0.00047	0.00005	0.00000	-0.00000
41	C2	DYY	-0.00002	-0.00058	0.00001	-0.00000	-0.00000
42	C2	DZZ	0.00001	-0.00041	0.00005	0.00000	0.00000
43	C2	DXY	-0.00002	0.00015	0.00003	0.00000	0.00000
44	C2	DXZ	0.00006	-0.00003	0.00001	-0.00000	0.00000
45	C2	DYZ	0.00002	0.00004	0.00000	0.00000	0.00000
46	C6	S1	-0.00001	-0.01827	-0.00066	0.00001	-0.00001
47	C6	S	-0.00013	-0.00144	0.00009	0.00004	-0.00001
48	C6	PX	0.00000	-0.00002	0.00002	0.00000	-0.00000
49	C6	PY	0.00002	0.00026	-0.00001	-0.00000	0.00000
50	C6	PZ	-0.00004	0.00030	0.00002	-0.00001	-0.00001

51	C6	S'	0.00133	0.00834	-0.00369	-0.00101	0.00076
52	C6	PX'	0.00180	0.00074	-0.00173	0.00040	-0.00020
53	C6	PY'	-0.00196	-0.00366	0.00178	-0.00066	0.00050
54	C6	PZ'	0.00168	-0.00245	-0.00105	0.00033	0.00021
55	C6	DXX	-0.00001	-0.00006	0.00004	0.00001	0.00001
56	C6	DYY	-0.00004	-0.00022	0.00004	-0.00002	0.00000
57	C6	DZZ	0.00005	-0.00021	0.00005	0.00000	0.00000
58	C6	DXY	-0.00001	0.00002	0.00000	0.00001	-0.00000
59	C6	DXZ	0.00006	-0.00004	-0.00001	-0.00000	0.00000
60	C6	DYZ	-0.00000	-0.00015	-0.00002	0.00000	0.00000
61	C5	S1	0.00074	-0.00061	0.03540	0.00001	0.00000
62	C5	S	0.00006	0.00002	0.00116	0.00002	0.00001
63	C5	PX	-0.00014	0.00001	-0.00023	-0.00000	0.00001
64	C5	PY	0.00001	0.00000	0.00030	-0.00000	-0.00001
65	C5	PZ	-0.00007	-0.00000	-0.00000	0.00000	0.00000
66	C5	S'	-0.00250	-0.00272	0.00831	-0.00021	-0.00024
67	C5	PX'	0.00120	-0.00046	0.00098	0.00006	0.00003
68	C5	PY'	0.00025	0.00195	-0.00291	0.00008	0.00003
69	C5	PZ'	0.00173	0.00123	-0.00160	0.00010	0.00005
70	C5	DXX	-0.00005	0.00006	-0.00060	0.00000	-0.00001
71	C5	DYY	0.00006	0.00004	-0.00075	-0.00000	-0.00000
72	C5	DZZ	-0.00010	0.00009	-0.00053	0.00000	0.00000
73	C5	DXY	-0.00005	-0.00001	0.00014	-0.00000	0.00000
74	C5	DXZ	-0.00006	0.00005	-0.00001	-0.00001	-0.00000
75	C5	DYZ	0.00003	-0.00001	0.00002	-0.00001	0.00000
76	C3	S1	0.00057	0.00044	0.02241	-0.00000	0.00000
77	C3	S	-0.00001	0.00012	0.00060	-0.00000	-0.00000
78	C3	PX	-0.00002	0.00000	0.00008	-0.00000	-0.00000
79	C3	PY	0.00003	-0.00001	-0.00024	0.00000	-0.00000
80	C3	PZ	-0.00015	0.00004	-0.00022	-0.00000	-0.00000
81	C3	S'	-0.00165	-0.00357	0.00745	0.00005	-0.00002
82	C3	PX'	0.00107	0.00130	-0.00138	-0.00002	0.00002
83	C3	PY'	-0.00034	-0.00190	0.00305	0.00003	-0.00002
84	C3	PZ'	0.00180	0.00014	0.00092	-0.00002	0.00001
85	C3	DXX	-0.00004	0.00004	-0.00039	0.00000	-0.00000
86	C3	DYY	0.00007	0.00003	-0.00053	0.00000	-0.00000
87	C3	DZZ	-0.00003	0.00005	-0.00053	-0.00000	0.00000
88	C3	DXY	-0.00002	-0.00001	0.00001	-0.00000	0.00000
89	C3	DXZ	-0.00003	0.00002	-0.00001	0.00000	0.00000

90	C3	DYZ	0.00007	-0.00003	-0.00018	-0.00000	-0.00000
91	H2	S	-0.00002	0.00001	-0.00007	-0.00000	-0.00000
92	H2	S'	0.00027	0.00050	-0.00052	-0.00001	-0.00001
93	H5	S	-0.00015	-0.00009	-0.00000	0.00001	-0.00000
94	H5	S'	-0.00021	-0.00057	0.00036	-0.00007	0.00000
95	H3	S	-0.00016	-0.00008	0.00003	0.00000	-0.00000
96	H3	S'	-0.00005	-0.00048	0.00045	0.00001	-0.00001
97	C7	S1	0.99175	0.00013	-0.03722	-0.00001	-0.00000
98	C7	S	0.04975	-0.00003	-0.00192	0.00000	-0.00000
99	C7	PX	-0.00006	0.00001	0.00015	-0.00000	0.00000
100	C7	PY	0.00000	-0.00001	-0.00002	0.00000	0.00000
101	C7	PZ	-0.00001	-0.00001	0.00020	-0.00000	0.00000
102	C7	S'	-0.01993	0.00101	0.00302	-0.00001	0.00009
103	C7	PX'	-0.00083	-0.00073	-0.00142	0.00013	-0.00008
104	C7	PY'	0.00017	0.00020	0.00040	-0.00003	0.00000
105	C7	PZ'	-0.00269	-0.00052	-0.00029	0.00015	-0.00010
106	C7	DXX	-0.00904	0.00000	0.00005	0.00000	0.00000
107	C7	DYY	-0.00926	-0.00001	0.00025	0.00000	-0.00000
108	C7	DZZ	-0.00899	0.00001	-0.00002	0.00000	0.00000
109	C7	DXY	-0.00006	-0.00001	0.00007	0.00000	0.00000
110	C7	DXZ	-0.00005	0.00001	-0.00024	-0.00000	0.00000
111	C7	DYZ	0.00002	0.00000	-0.00001	0.00000	0.00000
112	C8	S1	-0.00025	-0.00000	0.00006	-0.00000	-0.00000
113	C8	S	-0.00020	-0.00001	0.00008	-0.00000	-0.00000
114	C8	PX	0.00003	0.00000	-0.00003	0.00000	-0.00000
115	C8	PY	-0.00001	-0.00000	0.00001	-0.00000	-0.00000
116	C8	PZ	0.00000	0.00000	-0.00000	0.00000	0.00000
117	C8	S'	0.00314	0.00010	-0.00079	-0.00002	0.00002
118	C8	PX'	-0.00131	-0.00005	0.00080	0.00000	-0.00001
119	C8	PY'	0.00037	0.00002	-0.00021	-0.00000	0.00000
120	C8	PZ'	-0.00112	-0.00006	0.00061	0.00000	-0.00001
121	C8	DXX	-0.00001	-0.00000	0.00000	0.00000	0.00000
122	C8	DYY	-0.00007	-0.00000	0.00002	-0.00000	-0.00000
123	C8	DZZ	-0.00000	0.00000	0.00000	-0.00000	-0.00000
124	C8	DXY	-0.00002	-0.00000	0.00001	-0.00000	-0.00000
125	C8	DXZ	-0.00001	-0.00000	-0.00000	0.00000	-0.00000
126	C8	DYZ	-0.00000	0.00000	-0.00001	-0.00000	0.00000
127	C9	S1	-0.02559	-0.00000	0.00081	-0.00000	-0.00000
128	C9	S	-0.00185	0.00001	0.00019	-0.00001	0.00000

129	C9	PX	-0.00006	-0.00001	0.00007	0.00000	-0.00000
130	C9	PY	0.00007	0.00000	0.00004	0.00000	0.00000
131	C9	PZ	0.00040	0.00000	0.00005	-0.00000	-0.00000
132	C9	S'	0.00900	-0.00038	-0.00480	0.00021	-0.00004
133	C9	PX'	0.00105	0.00056	0.00142	-0.00024	0.00011
134	C9	PY'	-0.00054	-0.00017	-0.00056	0.00006	-0.00003
135	C9	PZ'	-0.00400	0.00004	-0.00055	0.00011	-0.00002
136	C9	DXX	-0.00005	-0.00000	-0.00003	0.00000	-0.00000
137	C9	DYY	0.00002	0.00000	0.00001	-0.00000	0.00000
138	C9	DZZ	-0.00027	-0.00001	0.00004	-0.00000	0.00000
139	C9	DXY	0.00002	0.00000	0.00001	-0.00000	0.00000
140	C9	DXZ	0.00006	0.00000	-0.00007	0.00000	0.00000
141	C9	DYZ	-0.00003	0.00000	0.00002	-0.00000	-0.00000
142	C10	S1	0.01632	0.00001	-0.00089	-0.00000	-0.00000
143	C10	S	0.00028	-0.00001	-0.00005	0.00000	-0.00000
144	C10	PX	0.00027	-0.00001	0.00007	0.00000	0.00000
145	C10	PY	-0.00014	0.00000	-0.00009	-0.00000	-0.00000
146	C10	PZ	-0.00016	-0.00000	0.00012	-0.00000	0.00000
147	C10	S'	0.00694	0.00035	-0.00155	-0.00007	0.00003
148	C10	PX'	-0.00174	0.00001	-0.00156	0.00001	-0.00000
149	C10	PY'	0.00077	0.00001	0.00062	-0.00000	0.00001
150	C10	PZ'	0.00272	0.00022	-0.00171	-0.00005	0.00002
151	C10	DXX	-0.00059	0.00000	0.00009	-0.00000	-0.00000
152	C10	DYY	-0.00035	-0.00000	-0.00000	-0.00000	-0.00000
153	C10	DZZ	-0.00036	0.00001	0.00002	-0.00000	0.00000
154	C10	DXY	0.00009	-0.00000	-0.00004	0.00000	-0.00000
155	C10	DXZ	0.00006	0.00000	-0.00009	0.00000	-0.00000
156	C10	DYZ	-0.00003	-0.00000	0.00003	-0.00000	0.00000
157	C11	S1	0.00034	0.00000	-0.00010	-0.00000	-0.00000
158	C11	S	0.00011	0.00001	-0.00019	-0.00000	-0.00000
159	C11	PX	0.00003	0.00001	-0.00003	-0.00000	0.00000
160	C11	PY	0.00001	-0.00000	0.00003	-0.00000	-0.00000
161	C11	PZ	0.00004	0.00000	-0.00001	-0.00000	-0.00000
162	C11	S'	-0.00336	-0.00023	0.00289	0.00004	-0.00002
163	C11	PX'	0.00187	0.00009	-0.00137	-0.00002	0.00000
164	C11	PY'	-0.00065	-0.00003	0.00026	0.00001	-0.00000
165	C11	PZ'	-0.00043	0.00003	-0.00078	-0.00000	-0.00000
166	C11	DXX	0.00006	-0.00000	0.00002	-0.00000	0.00000
167	C11	DYY	0.00003	0.00000	-0.00006	-0.00000	-0.00000

168	C11	DZZ	0.00003	-0.00000	0.00001	0.00000	-0.00000
169	C11	DXY	0.00000	0.00000	-0.00001	-0.00000	-0.00000
170	C11	DXZ	0.00002	-0.00000	0.00004	0.00000	0.00000
171	C11	DYZ	0.00000	0.00000	-0.00000	-0.00000	0.00000
172	C12	S1	-0.00048	0.00000	-0.00004	-0.00000	0.00000
173	C12	S	-0.00001	0.00001	-0.00012	-0.00000	0.00000
174	C12	PX	-0.00001	-0.00000	0.00001	0.00000	0.00000
175	C12	PY	-0.00000	0.00000	-0.00001	-0.00000	-0.00000
176	C12	PZ	-0.00001	0.00001	-0.00001	-0.00000	0.00000
177	C12	S'	-0.00201	-0.00026	0.00145	0.00004	-0.00005
178	C12	PX'	-0.00110	0.00003	-0.00021	0.00002	-0.00000
179	C12	PY'	0.00041	-0.00001	0.00011	-0.00000	0.00000
180	C12	PZ'	0.00143	0.00011	-0.00060	-0.00004	0.00003
181	C12	DXX	0.00007	0.00000	0.00001	-0.00000	0.00000
182	C12	DYY	0.00003	0.00000	-0.00003	-0.00000	0.00000
183	C12	DZZ	0.00009	-0.00000	-0.00001	0.00000	-0.00000
184	C12	DXY	-0.00002	0.00000	-0.00003	0.00000	0.00000
185	C12	DXZ	0.00004	-0.00000	0.00003	-0.00000	-0.00000
186	C12	DYZ	-0.00001	0.00000	-0.00002	-0.00000	0.00000
187	H7	S	0.00004	0.00001	-0.00019	-0.00000	0.00000
188	H7	S'	0.00050	0.00004	-0.00010	-0.00002	0.00001
189	H8	S	-0.00006	0.00000	-0.00003	0.00000	-0.00000
190	H8	S'	-0.00040	-0.00001	0.00023	0.00000	0.00000
191	H9	S	-0.00007	0.00000	-0.00002	0.00000	-0.00000
192	H9	S'	-0.00039	-0.00002	0.00008	0.00001	-0.00001
193	H10	S	0.00004	0.00000	-0.00007	0.00000	0.00000
194	H10	S'	0.00043	0.00002	-0.00030	0.00000	0.00000
195	C13	S1	-0.00014	0.02560	0.00015	0.00000	0.00001
196	C13	S	-0.00005	0.00137	-0.00002	0.00001	-0.00000
197	C13	PX	-0.00002	-0.00007	0.00002	0.00001	0.00000
198	C13	PY	0.00001	0.00001	-0.00001	-0.00000	0.00000
199	C13	PZ	-0.00004	-0.00013	0.00001	0.00000	-0.00000
200	C13	S'	-0.00053	0.00065	0.00124	-0.00014	0.00007
201	C13	PX'	-0.00022	-0.00004	0.00031	-0.00014	0.00001
202	C13	PY'	-0.00006	0.00012	0.00002	0.00000	-0.00002
203	C13	PZ'	-0.00033	0.00012	0.00081	-0.00010	0.00003
204	C13	DXX	-0.00004	-0.00043	0.00002	0.00000	0.00000
205	C13	DYY	-0.00002	-0.00027	-0.00001	0.00000	0.00000
206	C13	DZZ	-0.00004	-0.00057	0.00002	-0.00000	0.00000

207	C13	DXY	-0.00001	0.00007	0.00001	-0.00000	-0.00000
208	C13	DXZ	-0.00000	-0.00019	0.00001	-0.00000	0.00000
209	C13	DYZ	-0.00000	0.00003	0.00001	-0.00000	-0.00000
210	C14	S1	0.00000	0.00003	-0.00000	0.00000	-0.00000
211	C14	S	0.00000	0.00005	0.00000	-0.00000	-0.00000
212	C14	PX	0.00000	0.00002	-0.00000	0.00000	-0.00000
213	C14	PY	-0.00000	0.00000	0.00000	-0.00000	-0.00000
214	C14	PZ	0.00000	0.00001	-0.00001	0.00000	-0.00000
215	C14	S'	0.00000	-0.00068	-0.00013	0.00002	0.00000
216	C14	PX'	0.00001	-0.00055	-0.00004	0.00001	0.00000
217	C14	PY'	-0.00000	0.00018	0.00001	-0.00000	0.00000
218	C14	PZ'	0.00004	-0.00133	-0.00005	0.00001	-0.00000
219	C14	DXX	-0.00000	0.00001	-0.00000	0.00000	-0.00000
220	C14	DYY	0.00000	0.00001	-0.00000	0.00000	-0.00000
221	C14	DZZ	0.00000	0.00000	-0.00000	0.00000	0.00000
222	C14	DXY	-0.00000	0.00001	0.00000	-0.00000	0.00000
223	C14	DXZ	0.00000	-0.00002	-0.00000	0.00000	-0.00000
224	C14	DYZ	0.00000	-0.00000	0.00000	-0.00000	-0.00000
225	C15	S1	0.00000	0.00031	0.00000	-0.00000	-0.00000
226	C15	S	0.00001	-0.00003	-0.00001	0.00000	-0.00000
227	C15	PX	0.00001	-0.00014	-0.00001	-0.00000	-0.00000
228	C15	PY	-0.00000	0.00007	0.00000	0.00000	-0.00000
229	C15	PZ	0.00000	-0.00014	-0.00000	0.00000	-0.00000
230	C15	S'	-0.00008	-0.00181	-0.00003	-0.00008	-0.00000
231	C15	PX'	-0.00008	0.00135	-0.00004	-0.00003	-0.00000
232	C15	PY'	0.00004	-0.00040	-0.00002	-0.00000	0.00001
233	C15	PZ'	-0.00001	0.00211	-0.00008	0.00003	-0.00000
234	C15	DXX	0.00001	0.00006	-0.00001	-0.00000	-0.00000
235	C15	DYY	0.00001	-0.00006	-0.00000	0.00000	-0.00000
236	C15	DZZ	-0.00000	-0.00007	0.00001	-0.00000	-0.00000
237	C15	DXY	0.00001	0.00000	-0.00000	0.00000	0.00000
238	C15	DXZ	-0.00000	-0.00009	-0.00001	0.00000	-0.00000
239	C15	DYZ	-0.00000	0.00001	0.00000	-0.00000	-0.00000
240	C16	S1	-0.00001	0.00048	0.00000	0.00000	0.00001
241	C16	S	-0.00000	0.00004	-0.00001	-0.00000	0.00000
242	C16	PX	-0.00000	-0.00006	0.00000	0.00000	-0.00000
243	C16	PY	0.00001	-0.00005	-0.00000	0.00000	0.00000
244	C16	PZ	0.00001	-0.00017	0.00000	0.00000	0.00000
245	C16	S'	0.00005	-0.00249	0.00021	0.00004	-0.00002

246	C16	PX'	-0.00001	0.00164	-0.00010	0.00001	0.00001
247	C16	PY'	0.00000	-0.00011	0.00000	-0.00000	-0.00000
248	C16	PZ'	-0.00008	0.00234	-0.00004	0.00002	0.00000
249	C16	DXX	-0.00000	0.00000	0.00000	-0.00000	0.00000
250	C16	DYY	-0.00000	-0.00004	-0.00000	0.00000	0.00000
251	C16	DZZ	0.00000	-0.00002	0.00000	0.00000	0.00000
252	C16	DXY	-0.00000	0.00002	0.00000	-0.00000	-0.00000
253	C16	DXZ	-0.00000	-0.00013	0.00000	0.00000	-0.00000
254	C16	DYZ	0.00000	-0.00000	-0.00000	-0.00000	0.00000
255	C17	S1	-0.00000	0.00003	-0.00000	-0.00000	-0.00001
256	C17	S	0.00000	-0.00018	0.00001	0.00000	0.00000
257	C17	PX	0.00000	-0.00003	0.00000	-0.00000	0.00000
258	C17	PY	-0.00000	0.00003	0.00000	0.00000	0.00000
259	C17	PZ	0.00000	-0.00001	-0.00001	0.00000	-0.00000
260	C17	S'	0.00010	0.00217	-0.00042	0.00009	0.00010
261	C17	PX'	0.00003	0.00049	-0.00022	0.00005	0.00002
262	C17	PY'	0.00004	-0.00054	-0.00004	0.00001	0.00000
263	C17	PZ'	-0.00021	0.00301	0.00024	-0.00009	-0.00003
264	C17	DXX	0.00000	-0.00000	0.00000	0.00000	0.00000
265	C17	DYY	0.00000	-0.00006	0.00000	0.00000	0.00000
266	C17	DZZ	0.00000	0.00004	-0.00001	0.00000	0.00000
267	C17	DXY	0.00000	-0.00002	-0.00000	0.00000	0.00000
268	C17	DXZ	-0.00000	0.00007	0.00000	-0.00000	-0.00000
269	C17	DYZ	0.00000	-0.00002	0.00000	0.00000	0.00000
270	C18	S1	0.00000	-0.00006	-0.00000	-0.00000	-0.00000
271	C18	S	0.00000	-0.00020	-0.00001	-0.00000	-0.00000
272	C18	PX	-0.00000	0.00004	-0.00000	0.00000	-0.00000
273	C18	PY	-0.00000	-0.00001	0.00000	-0.00000	-0.00000
274	C18	PZ	0.00000	0.00001	-0.00000	0.00000	0.00000
275	C18	S'	-0.00006	0.00337	0.00002	0.00001	0.00000
276	C18	PX'	-0.00001	0.00126	0.00006	0.00000	0.00001
277	C18	PY'	-0.00001	-0.00005	0.00000	-0.00000	-0.00000
278	C18	PZ'	-0.00003	0.00103	-0.00003	0.00001	-0.00000
279	C18	DXX	-0.00000	0.00001	0.00000	0.00000	0.00000
280	C18	DYY	0.00000	-0.00007	-0.00000	0.00000	-0.00000
281	C18	DZZ	-0.00000	0.00004	0.00000	0.00000	-0.00000
282	C18	DXY	-0.00000	0.00000	-0.00000	0.00000	-0.00000
283	C18	DXZ	-0.00000	0.00006	0.00000	0.00000	0.00000
284	C18	DYZ	-0.00000	-0.00000	0.00000	-0.00000	-0.00000

285	H1	S	0.00000	-0.00021	0.00000	-0.00000	0.00000
286	H1	S'	-0.00001	-0.00011	0.00002	-0.00001	0.00000
287	H11	S	0.00001	-0.00020	0.00001	-0.00000	0.00000
288	H11	S'	-0.00001	-0.00015	0.00002	-0.00001	-0.00000
289	H13	S	0.00000	-0.00004	0.00000	-0.00000	0.00000
290	H13	S'	-0.00000	0.00023	0.00003	-0.00000	0.00000
291	H14	S	0.00000	-0.00011	0.00000	0.00000	-0.00000
292	H14	S'	0.00001	-0.00047	-0.00001	0.00001	0.00000
293	C19	S1	0.00000	-0.00005	0.00000	-0.00000	0.00000
294	C19	S	0.00000	-0.00005	0.00000	-0.00000	-0.00000
295	C19	PX	-0.00000	-0.00002	0.00000	-0.00000	-0.00000
296	C19	PY	0.00000	-0.00000	-0.00000	0.00000	0.00000
297	C19	PZ	0.00000	-0.00004	-0.00000	0.00000	-0.00000
298	C19	S'	0.00013	-0.00009	-0.00034	0.00012	0.00043
299	C19	PX'	0.00014	-0.00100	-0.00025	0.00008	0.00011
300	C19	PY'	0.00004	-0.00023	-0.00010	0.00003	0.00005
301	C19	PZ'	-0.00030	0.00169	0.00063	-0.00018	-0.00027
302	C19	DXX	-0.00000	0.00002	-0.00000	-0.00000	0.00000
303	C19	DYY	0.00000	-0.00004	0.00000	-0.00000	-0.00000
304	C19	DZZ	0.00000	-0.00004	0.00000	-0.00000	0.00000
305	C19	DXY	0.00000	-0.00000	-0.00000	-0.00000	0.00000
306	C19	DXZ	0.00000	-0.00002	-0.00000	0.00000	-0.00000
307	C19	DYZ	-0.00000	0.00000	0.00000	-0.00000	-0.00000
308	C20	S1	-0.00000	0.00000	0.00000	0.00000	-0.00000
309	C20	S	0.00000	-0.00000	-0.00001	0.00000	-0.00000
310	C20	PX	0.00000	-0.00001	0.00000	-0.00000	-0.00000
311	C20	PY	-0.00000	0.00001	0.00000	-0.00000	-0.00000
312	C20	PZ	0.00000	0.00000	-0.00000	0.00000	0.00000
313	C20	S'	-0.00023	0.00123	0.00051	-0.00013	-0.00008
314	C20	PX'	0.00008	-0.00033	-0.00019	0.00006	0.00018
315	C20	PY'	0.00007	-0.00037	-0.00014	0.00004	0.00012
316	C20	PZ'	-0.00025	0.00122	0.00051	-0.00018	-0.00049
317	C20	DXX	-0.00000	-0.00000	0.00000	0.00000	-0.00000
318	C20	DYY	-0.00000	-0.00000	-0.00000	0.00000	0.00000
319	C20	DZZ	-0.00000	0.00001	0.00000	-0.00000	0.00000
320	C20	DXY	-0.00000	0.00000	0.00000	-0.00000	0.00000
321	C20	DXZ	0.00000	-0.00000	-0.00000	0.00000	0.00000
322	C20	DYZ	-0.00000	-0.00000	-0.00000	0.00000	-0.00000
323	C21	S1	-0.00003	-0.00023	-0.00004	-0.00001	0.00000

324	C21	S	-0.00004	-0.00013	-0.00003	0.00000	-0.00004
325	C21	PX	-0.00001	-0.00005	-0.00002	-0.00001	-0.00001
326	C21	PY	0.00000	0.00001	0.00002	0.00001	0.00001
327	C21	PZ	-0.00002	0.00002	-0.00002	0.00001	0.00000
328	C21	S'	-0.00010	0.00128	-0.00137	-0.00137	0.00212
329	C21	PX'	0.00116	-0.00001	0.00038	0.00112	-0.00098
330	C21	PY'	-0.00205	0.00048	-0.00024	-0.00172	0.00163
331	C21	PZ'	0.00035	0.00020	0.00043	0.00026	-0.00072
332	C21	DXX	-0.00002	0.00001	-0.00001	-0.00001	-0.00000
333	C21	DYY	0.00001	0.00010	-0.00005	0.00000	0.00002
334	C21	DZZ	-0.00002	0.00007	-0.00001	0.00001	0.00000
335	C21	DXY	-0.00001	-0.00007	0.00000	0.00001	-0.00001
336	C21	DXZ	-0.00001	0.00004	0.00001	-0.00000	0.00001
337	C21	DYZ	0.00003	-0.00001	-0.00000	-0.00001	-0.00001
338	C22	S1	0.00000	-0.00003	-0.00000	-0.00011	0.00001
339	C22	S	0.00000	-0.00001	-0.00002	-0.00003	0.00002
340	C22	PX	-0.00000	-0.00001	0.00001	0.00005	0.00002
341	C22	PY	0.00000	0.00002	-0.00001	-0.00001	-0.00003
342	C22	PZ	-0.00001	-0.00001	0.00001	-0.00009	0.00001
343	C22	S'	0.00137	-0.00025	0.00090	0.00139	-0.00250
344	C22	PX'	0.00078	-0.00054	0.00022	0.00075	-0.00114
345	C22	PY'	-0.00123	0.00071	-0.00051	-0.00149	0.00169
346	C22	PZ'	0.00080	-0.00048	0.00002	0.00125	-0.00051
347	C22	DXX	0.00001	-0.00000	-0.00000	-0.00003	0.00000
348	C22	DYY	0.00000	-0.00001	0.00002	-0.00002	-0.00002
349	C22	DZZ	-0.00000	0.00000	0.00000	-0.00005	-0.00002
350	C22	DXY	-0.00000	0.00003	-0.00001	-0.00002	0.00001
351	C22	DXZ	0.00000	-0.00002	0.00000	0.00001	-0.00000
352	C22	DYZ	-0.00000	0.00002	-0.00001	-0.00001	0.00002
353	C23	S1	-0.00027	0.00000	-0.00016	-0.00000	-0.00000
354	C23	S	-0.00008	-0.00000	-0.00005	-0.00000	-0.00000
355	C23	PX	0.00001	-0.00001	-0.00001	0.00000	-0.00000
356	C23	PY	0.00002	-0.00000	0.00005	-0.00000	0.00000
357	C23	PZ	0.00002	-0.00000	0.00016	0.00000	0.00000
358	C23	S'	0.00094	-0.00021	-0.00202	0.00023	-0.00005
359	C23	PX'	-0.00121	0.00070	0.00431	-0.00045	0.00013
360	C23	PY'	0.00036	-0.00022	-0.00151	0.00017	-0.00005
361	C23	PZ'	0.00114	-0.00034	-0.00235	0.00015	-0.00006
362	C23	DXX	0.00014	0.00000	-0.00005	-0.00000	0.00000

363	C23	DYY	-0.00001	0.00000	-0.00008	0.00000	0.00000
364	C23	DZZ	0.00007	0.00000	-0.00011	-0.00000	-0.00000
365	C23	DXY	-0.00005	0.00000	0.00001	-0.00000	-0.00000
366	C23	DXZ	-0.00001	-0.00000	0.00004	-0.00000	-0.00000
367	C23	DYZ	0.00001	0.00000	-0.00001	0.00000	0.00000
368	C24	S1	-0.00003	0.00000	0.00000	0.00001	-0.00000
369	C24	S	-0.00003	-0.00001	-0.00003	0.00002	-0.00000
370	C24	PX	-0.00004	0.00000	0.00002	0.00000	-0.00000
371	C24	PY	0.00001	0.00000	-0.00001	-0.00000	0.00000
372	C24	PZ	0.00002	-0.00000	-0.00002	-0.00000	0.00000
373	C24	S'	-0.00133	0.00059	0.00391	-0.00057	0.00010
374	C24	PX'	-0.00100	0.00046	0.00319	-0.00023	0.00010
375	C24	PY'	0.00044	-0.00019	-0.00123	0.00004	-0.00004
376	C24	PZ'	0.00029	-0.00022	-0.00138	0.00022	-0.00005
377	C24	DXX	-0.00005	0.00000	0.00005	-0.00000	0.00000
378	C24	DYY	-0.00000	0.00000	0.00000	0.00000	-0.00000
379	C24	DZZ	-0.00001	0.00000	0.00000	0.00001	0.00000
380	C24	DXY	0.00002	-0.00000	-0.00001	0.00001	0.00000
381	C24	DXZ	0.00002	-0.00000	-0.00002	-0.00000	-0.00000
382	C24	DYZ	-0.00001	0.00000	0.00000	0.00000	-0.00000
383	C25	S1	0.00000	-0.00000	-0.00000	0.00000	-0.00000
384	C25	S	0.00000	-0.00002	-0.00000	0.00000	0.00000
385	C25	PX	-0.00000	0.00000	-0.00000	-0.00000	-0.00000
386	C25	PY	0.00000	-0.00000	-0.00000	-0.00000	-0.00000
387	C25	PZ	-0.00000	0.00000	0.00000	0.00000	0.00000
388	C25	S'	-0.00012	0.00056	0.00023	-0.00006	-0.00020
389	C25	PX'	0.00004	-0.00023	-0.00006	0.00003	0.00013
390	C25	PY'	-0.00000	0.00001	-0.00004	0.00004	0.00010
391	C25	PZ'	-0.00010	0.00043	0.00019	-0.00008	-0.00037
392	C25	DXX	0.00000	-0.00000	-0.00000	-0.00000	-0.00000
393	C25	DYY	-0.00000	0.00000	0.00000	0.00000	-0.00000
394	C25	DZZ	-0.00000	-0.00000	0.00000	-0.00000	-0.00000
395	C25	DXY	0.00000	0.00000	-0.00000	-0.00000	-0.00000
396	C25	DXZ	0.00000	-0.00001	-0.00000	0.00000	0.00000
397	C25	DYZ	0.00000	-0.00000	-0.00000	0.00000	0.00000
398	C26	S1	0.00000	-0.00000	-0.00000	-0.00000	-0.00000
399	C26	S	0.00000	-0.00000	0.00000	-0.00000	-0.00001
400	C26	PX	0.00000	-0.00000	0.00000	0.00000	0.00000
401	C26	PY	-0.00000	-0.00000	0.00000	0.00000	-0.00000

402	C26	PZ	0.00000	-0.00000	-0.00000	-0.00000	-0.00000
403	C26	S'	-0.00001	0.00003	0.00001	0.00001	-0.00000
404	C26	PX'	0.00000	0.00000	-0.00000	-0.00000	-0.00001
405	C26	PY'	0.00000	-0.00001	-0.00000	-0.00000	-0.00000
406	C26	PZ'	-0.00000	0.00002	0.00000	0.00001	0.00002
407	C26	DXX	0.00000	-0.00000	-0.00000	-0.00000	-0.00000
408	C26	DYY	-0.00000	0.00000	0.00000	-0.00000	-0.00000
409	C26	DZZ	0.00000	-0.00000	-0.00000	0.00000	0.00000
410	C26	DXY	0.00000	-0.00000	-0.00000	-0.00000	-0.00000
411	C26	DXZ	-0.00000	0.00000	0.00000	-0.00000	-0.00000
412	C26	DYZ	0.00000	-0.00000	-0.00000	0.00000	-0.00000
413	C27	S1	-0.00000	0.00000	0.00000	-0.00000	-0.00001
414	C27	S	-0.00000	0.00001	0.00000	-0.00000	-0.00002
415	C27	PX	0.00000	-0.00000	-0.00000	-0.00000	-0.00000
416	C27	PY	-0.00000	0.00000	0.00000	-0.00000	0.00001
417	C27	PZ	-0.00000	-0.00000	-0.00000	0.00000	0.00000
418	C27	S'	0.00007	-0.00026	-0.00012	0.00017	0.00117
419	C27	PX'	-0.00003	0.00013	0.00006	-0.00006	-0.00037
420	C27	PY'	0.00003	-0.00011	-0.00009	0.00011	0.00059
421	C27	PZ'	0.00006	-0.00020	-0.00008	0.00005	0.00046
422	C27	DXX	-0.00000	0.00000	0.00000	0.00000	0.00000
423	C27	DYY	0.00000	-0.00000	-0.00000	0.00000	0.00001
424	C27	DZZ	0.00000	-0.00000	-0.00000	0.00000	0.00000
425	C27	DXY	-0.00000	-0.00000	0.00000	-0.00000	-0.00000
426	C27	DXZ	-0.00000	0.00000	0.00000	-0.00000	-0.00000
427	C27	DYZ	-0.00000	0.00000	-0.00000	0.00000	0.00000
428	C28	S1	0.00000	-0.00000	-0.00000	0.00000	-0.00000
429	C28	S	0.00000	-0.00001	-0.00000	-0.00000	-0.00000
430	C28	PX	0.00000	-0.00000	-0.00000	0.00000	0.00000
431	C28	PY	-0.00000	0.00000	0.00000	-0.00000	-0.00000
432	C28	PZ	-0.00000	0.00000	-0.00000	-0.00000	0.00000
433	C28	S'	-0.00003	0.00018	0.00004	-0.00001	-0.00007
434	C28	PX'	0.00001	-0.00002	-0.00001	0.00000	0.00001
435	C28	PY'	-0.00002	0.00009	0.00003	-0.00001	-0.00004
436	C28	PZ'	-0.00000	0.00000	-0.00001	0.00001	0.00001
437	C28	DXX	0.00000	-0.00000	-0.00000	0.00000	-0.00000
438	C28	DYY	-0.00000	0.00000	0.00000	-0.00000	-0.00000
439	C28	DZZ	0.00000	0.00000	-0.00000	0.00000	0.00000
440	C28	DXY	0.00000	-0.00000	-0.00000	0.00000	0.00000

441 C28	DXZ	0.00000	-0.00000	0.00000	-0.00000	-0.00000
442 C28	DYZ	0.00000	-0.00000	-0.00000	-0.00000	-0.00000
443 C29	S1	-0.00000	0.00000	-0.00000	0.00000	-0.00000
444 C29	S	-0.00000	0.00000	-0.00000	0.00000	-0.00000
445 C29	PX	-0.00000	0.00000	0.00000	0.00000	0.00000
446 C29	PY	-0.00000	0.00000	0.00000	-0.00000	-0.00000
447 C29	PZ	0.00000	-0.00000	-0.00000	0.00000	0.00000
448 C29	S'	0.00001	-0.00005	-0.00001	-0.00000	0.00001
449 C29	PX'	-0.00000	0.00001	0.00000	0.00000	-0.00000
450 C29	PY'	0.00000	-0.00002	-0.00000	-0.00000	0.00000
451 C29	PZ'	0.00000	-0.00001	0.00000	-0.00000	0.00000
452 C29	DXX	0.00000	-0.00000	-0.00000	-0.00000	-0.00000
453 C29	DYY	-0.00000	0.00000	0.00000	-0.00000	-0.00000
454 C29	DZZ	0.00000	-0.00000	0.00000	-0.00000	-0.00000
455 C29	DXY	0.00000	-0.00000	-0.00000	0.00000	-0.00000
456 C29	DXZ	0.00000	-0.00000	-0.00000	0.00000	-0.00000
457 C29	DYZ	-0.00000	0.00000	0.00000	-0.00000	-0.00000
458 C30	S1	0.00000	-0.00000	-0.00000	-0.00000	-0.00000
459 C30	S	-0.00000	-0.00000	-0.00000	-0.00000	-0.00001
460 C30	PX	0.00000	-0.00000	-0.00000	0.00000	0.00000
461 C30	PY	-0.00000	0.00000	0.00000	-0.00000	-0.00000
462 C30	PZ	0.00000	-0.00000	-0.00000	-0.00000	-0.00000
463 C30	S'	0.00002	-0.00007	-0.00002	-0.00001	0.00008
464 C30	PX'	-0.00000	0.00000	-0.00000	0.00000	0.00001
465 C30	PY'	-0.00001	0.00002	0.00001	-0.00001	-0.00006
466 C30	PZ'	0.00001	-0.00003	-0.00001	-0.00000	0.00003
467 C30	DXX	-0.00000	0.00000	0.00000	-0.00000	-0.00000
468 C30	DYY	0.00000	-0.00000	-0.00000	0.00000	0.00000
469 C30	DZZ	-0.00000	0.00000	0.00000	-0.00000	-0.00000
470 C30	DXY	-0.00000	-0.00000	-0.00000	-0.00000	-0.00000
471 C30	DXZ	0.00000	-0.00000	-0.00000	0.00000	0.00000
472 C30	DYZ	-0.00000	0.00000	0.00000	-0.00000	-0.00000
473 H15	S	-0.00000	0.00000	0.00000	-0.00000	-0.00000
474 H15	S'	-0.00000	0.00002	0.00001	-0.00000	-0.00001
475 H16	S	0.00000	-0.00000	0.00000	-0.00000	-0.00000
476 H16	S'	0.00000	-0.00000	0.00000	-0.00000	0.00000
477 H17	S	-0.00000	0.00000	-0.00000	0.00000	0.00000
478 H17	S'	0.00000	-0.00000	-0.00000	0.00000	0.00003
479 H18	S	-0.00000	0.00000	-0.00000	0.00000	0.00000

480	H18	S'	-0.00000	0.00000	-0.00000	0.00000	0.00002
481	C31	S1	0.00000	-0.00000	-0.00001	0.00001	-0.00000
482	C31	S	0.00001	-0.00000	-0.00002	-0.00001	-0.00000
483	C31	PX	0.00000	0.00000	0.00001	-0.00001	-0.00000
484	C31	PY	-0.00000	-0.00000	0.00001	0.00000	0.00000
485	C31	PZ	-0.00000	-0.00000	0.00000	0.00001	-0.00000
486	C31	S'	-0.00062	0.00019	0.00141	-0.00013	0.00004
487	C31	PX'	-0.00011	0.00014	0.00085	0.00006	0.00004
488	C31	PY'	0.00002	-0.00008	-0.00056	0.00012	-0.00003
489	C31	PZ'	0.00015	-0.00005	-0.00029	-0.00031	-0.00001
490	C31	DXX	0.00001	0.00000	-0.00000	-0.00000	0.00000
491	C31	DYY	0.00000	0.00000	0.00000	0.00000	0.00000
492	C31	DZZ	0.00000	-0.00000	-0.00000	-0.00000	-0.00000
493	C31	DXY	0.00000	-0.00000	-0.00001	-0.00000	-0.00000
494	C31	DXZ	-0.00000	-0.00000	-0.00000	0.00001	-0.00000
495	C31	DYZ	0.00000	0.00000	0.00000	-0.00001	0.00000
496	C32	S1	-0.00000	-0.00000	-0.00000	-0.00001	-0.00000
497	C32	S	-0.00000	-0.00000	-0.00001	-0.00005	0.00000
498	C32	PX	0.00000	-0.00000	-0.00000	-0.00000	-0.00000
499	C32	PY	-0.00000	0.00000	0.00000	0.00001	0.00000
500	C32	PZ	-0.00000	-0.00000	-0.00000	-0.00002	-0.00000
501	C32	S'	0.00011	0.00001	0.00002	0.00095	0.00000
502	C32	PX'	0.00007	0.00001	-0.00000	0.00030	-0.00000
503	C32	PY'	-0.00004	-0.00001	0.00001	-0.00011	0.00000
504	C32	PZ'	-0.00003	0.00000	-0.00001	-0.00021	0.00000
505	C32	DXX	0.00000	-0.00000	-0.00000	0.00000	-0.00000
506	C32	DYY	-0.00000	-0.00000	-0.00000	0.00000	0.00000
507	C32	DZZ	-0.00000	-0.00000	0.00000	0.00001	0.00000
508	C32	DXY	-0.00000	-0.00000	-0.00000	-0.00001	0.00000
509	C32	DXZ	-0.00000	0.00000	0.00000	-0.00000	0.00000
510	C32	DYZ	0.00000	-0.00000	-0.00000	-0.00001	-0.00000
511	C33	S1	-0.00000	-0.00000	-0.00001	-0.00002	0.00000
512	C33	S	0.00000	-0.00000	-0.00002	-0.00002	-0.00000
513	C33	PX	0.00000	-0.00000	-0.00001	-0.00000	-0.00000
514	C33	PY	-0.00000	0.00000	0.00000	0.00000	0.00000
515	C33	PZ	-0.00000	0.00000	0.00000	0.00000	-0.00000
516	C33	S'	0.00001	0.00003	0.00012	0.00045	0.00001
517	C33	PX'	-0.00002	-0.00000	-0.00004	-0.00002	-0.00000
518	C33	PY'	0.00002	0.00001	0.00010	0.00012	0.00001

519	C33	PZ'	-0.00001	-0.00002	-0.00008	-0.00018	-0.00001
520	C33	DXX	-0.00000	-0.00000	-0.00001	0.00000	-0.00000
521	C33	DYY	-0.00000	-0.00000	-0.00000	-0.00000	0.00000
522	C33	DZZ	0.00000	0.00000	-0.00000	0.00000	0.00000
523	C33	DXY	0.00000	-0.00000	-0.00000	-0.00000	-0.00000
524	C33	DXZ	-0.00000	0.00000	0.00000	-0.00000	0.00000
525	C33	DYZ	0.00000	-0.00000	-0.00001	-0.00000	-0.00000
526	C34	S1	0.00001	0.00000	0.00001	0.00001	-0.00000
527	C34	S	-0.00001	-0.00000	-0.00001	0.00002	0.00000
528	C34	PX	0.00001	0.00000	0.00000	0.00002	0.00000
529	C34	PY	-0.00000	-0.00000	0.00001	-0.00001	0.00000
530	C34	PZ	-0.00001	-0.00000	-0.00001	-0.00001	-0.00000
531	C34	S'	0.00022	0.00004	0.00010	-0.00078	0.00000
532	C34	PX'	-0.00007	-0.00003	-0.00006	0.00002	-0.00000
533	C34	PY'	-0.00006	0.00000	-0.00006	-0.00005	0.00000
534	C34	PZ'	0.00018	0.00005	0.00022	0.00008	0.00001
535	C34	DXX	0.00000	0.00000	-0.00000	0.00001	0.00000
536	C34	DYY	0.00000	-0.00000	-0.00000	-0.00000	0.00000
537	C34	DZZ	0.00000	0.00000	0.00001	0.00002	0.00000
538	C34	DXY	-0.00000	-0.00000	-0.00000	0.00001	-0.00000
539	C34	DXZ	0.00000	-0.00000	-0.00000	-0.00002	0.00000
540	C34	DYZ	-0.00000	-0.00000	-0.00000	0.00001	0.00000
541	C35	S1	-0.00000	-0.00000	-0.00000	-0.00032	-0.00000
542	C35	S	0.00001	0.00000	-0.00000	-0.00002	0.00000
543	C35	PX	0.00000	-0.00000	-0.00001	0.00001	-0.00000
544	C35	PY	-0.00000	0.00000	0.00000	-0.00006	0.00000
545	C35	PZ	0.00000	-0.00000	-0.00000	0.00009	-0.00000
546	C35	S'	0.00004	-0.00007	-0.00019	-0.00039	-0.00011
547	C35	PX'	-0.00015	-0.00001	0.00006	0.00063	0.00003
548	C35	PY'	0.00021	-0.00000	-0.00007	-0.00011	-0.00005
549	C35	PZ'	-0.00016	0.00001	0.00004	-0.00073	0.00004
550	C35	DXX	-0.00000	-0.00000	-0.00000	-0.00005	0.00000
551	C35	DYY	0.00000	-0.00000	-0.00000	-0.00009	0.00000
552	C35	DZZ	0.00000	0.00000	-0.00000	-0.00005	0.00000
553	C35	DXY	0.00000	0.00000	-0.00000	0.00005	-0.00000
554	C35	DXZ	0.00000	-0.00000	-0.00000	-0.00004	0.00000
555	C35	DYZ	-0.00000	0.00000	0.00000	0.00006	-0.00000
556	C36	S1	0.00000	-0.00000	-0.00000	0.00003	0.00000
557	C36	S	0.00000	-0.00000	-0.00001	0.00004	0.00000

558	C36	PX	0.00000	-0.00000	-0.00000	0.00001	0.00000
559	C36	PY	-0.00000	-0.00000	-0.00000	-0.00001	0.00000
560	C36	PZ	0.00000	0.00000	0.00000	0.00000	-0.00000
561	C36	S'	-0.00007	-0.00001	-0.00001	-0.00045	-0.00001
562	C36	PX'	-0.00002	0.00000	0.00001	-0.00009	-0.00000
563	C36	PY'	-0.00001	-0.00001	0.00000	-0.00010	-0.00000
564	C36	PZ'	0.00004	0.00000	-0.00002	0.00029	0.00001
565	C36	DXX	0.00000	0.00000	-0.00000	0.00000	0.00000
566	C36	DYY	0.00000	-0.00000	-0.00000	0.00000	0.00000
567	C36	DZZ	0.00000	0.00000	0.00000	-0.00000	-0.00000
568	C36	DXY	0.00000	-0.00000	-0.00000	0.00001	0.00000
569	C36	DXZ	0.00000	-0.00000	-0.00000	-0.00000	-0.00000
570	C36	DYZ	0.00000	0.00000	-0.00000	0.00001	0.00000
571	H4	S	0.00000	0.00000	0.00000	0.00000	0.00000
572	H4	S'	0.00001	0.00001	0.00005	0.00004	0.00000
573	H19	S	-0.00000	-0.00000	-0.00000	-0.00001	-0.00000
574	H19	S'	0.00011	0.00003	0.00015	0.00022	0.00001
575	H21	S	-0.00000	0.00000	0.00000	-0.00001	-0.00000
576	H21	S'	-0.00001	0.00000	0.00002	-0.00008	-0.00000
577	H22	S	0.00000	0.00000	-0.00000	0.00001	0.00000
578	H22	S'	0.00003	0.00000	-0.00000	0.00004	-0.00000
579	C37	S1	-0.00000	0.00000	-0.00000	0.00006	0.00022
580	C37	S	-0.00002	0.00001	0.00001	-0.00000	0.00009
581	C37	PX	0.00000	-0.00000	0.00000	-0.00002	-0.00000
582	C37	PY	0.00000	-0.00001	-0.00001	0.00002	-0.00001
583	C37	PZ	0.00000	0.00001	-0.00001	0.00003	-0.00000
584	C37	S'	0.00066	-0.00038	0.00014	0.00035	-0.00386
585	C37	PX'	0.00027	-0.00016	-0.00009	0.00072	0.00106
586	C37	PY'	-0.00052	0.00043	0.00014	-0.00102	-0.00138
587	C37	PZ'	0.00036	-0.00044	0.00019	0.00032	0.00022
588	C37	DXX	-0.00000	0.00000	0.00000	0.00002	0.00003
589	C37	DYY	0.00000	-0.00000	-0.00001	0.00002	0.00002
590	C37	DZZ	0.00000	0.00001	-0.00000	0.00003	0.00005
591	C37	DXY	-0.00000	-0.00000	-0.00000	-0.00000	-0.00000
592	C37	DXZ	0.00000	0.00000	0.00000	-0.00001	-0.00003
593	C37	DYZ	-0.00001	-0.00000	0.00001	0.00001	0.00001
594	C38	S1	0.00000	0.00001	-0.00000	-0.00032	0.33298
595	C38	S	0.00000	0.00001	0.00000	-0.00003	0.01605
596	C38	PX	-0.00000	-0.00000	0.00000	0.00000	0.00011

597	C38	PY	0.00000	0.00000	0.00000	-0.00001	-0.00005
598	C38	PZ	-0.00000	0.00000	-0.00000	0.00002	-0.00029
599	C38	S'	0.00003	-0.00014	-0.00004	0.00085	0.00319
600	C38	PX'	0.00000	-0.00004	-0.00000	-0.00006	0.00038
601	C38	PY'	0.00000	0.00006	-0.00000	-0.00003	-0.00134
602	C38	PZ'	-0.00000	-0.00004	0.00000	0.00032	0.00236
603	C38	DXX	-0.00000	0.00000	-0.00000	0.00000	-0.00337
604	C38	DYY	-0.00000	-0.00000	0.00000	-0.00000	-0.00335
605	C38	DZZ	0.00000	0.00000	-0.00000	0.00001	-0.00344
606	C38	DXY	0.00000	0.00000	-0.00000	0.00000	-0.00003
607	C38	DXZ	-0.00000	0.00000	0.00000	-0.00000	0.00002
608	C38	DYZ	0.00000	-0.00000	-0.00000	-0.00000	-0.00000
609	C39	S1	-0.00000	0.00000	0.00000	-0.00243	-0.01088
610	C39	S	0.00000	-0.00001	0.00001	-0.00008	-0.00103
611	C39	PX	-0.00000	0.00000	-0.00000	0.00005	0.00011
612	C39	PY	0.00000	-0.00000	0.00000	0.00001	-0.00024
613	C39	PZ	0.00000	-0.00000	-0.00000	-0.00012	0.00026
614	C39	S'	-0.00021	0.00014	-0.00014	-0.00012	0.00648
615	C39	PX'	0.00015	-0.00008	-0.00011	0.00060	0.00075
616	C39	PY'	0.00002	-0.00009	0.00005	-0.00003	0.00130
617	C39	PZ'	-0.00042	0.00039	0.00010	-0.00172	-0.00531
618	C39	DXX	0.00000	-0.00000	-0.00000	-0.00002	-0.00013
619	C39	DYY	-0.00000	0.00000	-0.00000	-0.00001	-0.00021
620	C39	DZZ	-0.00000	0.00000	0.00000	-0.00019	-0.00022
621	C39	DXY	-0.00000	-0.00000	0.00000	0.00001	0.00009
622	C39	DXZ	-0.00000	0.00000	0.00000	0.00008	-0.00001
623	C39	DYZ	0.00000	-0.00000	0.00000	0.00001	0.00010
624	C40	S1	0.00000	0.00000	-0.00000	0.00002	-0.00156
625	C40	S	-0.00000	0.00002	-0.00001	0.00003	-0.00014
626	C40	PX	-0.00000	-0.00000	0.00000	-0.00001	0.00002
627	C40	PY	0.00000	0.00000	-0.00000	0.00002	-0.00003
628	C40	PZ	-0.00000	0.00000	0.00000	-0.00000	0.00001
629	C40	S'	0.00007	-0.00027	0.00020	-0.00013	0.00052
630	C40	PX'	-0.00005	0.00015	-0.00004	-0.00002	-0.00043
631	C40	PY'	0.00006	-0.00016	0.00001	0.00000	0.00043
632	C40	PZ'	0.00003	-0.00013	0.00005	0.00004	0.00036
633	C40	DXX	-0.00000	-0.00000	0.00000	0.00001	-0.00000
634	C40	DYY	-0.00000	-0.00000	-0.00000	-0.00000	0.00001
635	C40	DZZ	-0.00000	-0.00001	0.00000	-0.00001	0.00001

636	C40	DXY	-0.00000	0.00001	-0.00000	0.00000	-0.00001
637	C40	DXZ	-0.00000	0.00000	0.00000	-0.00001	-0.00000
638	C40	DYZ	0.00000	-0.00000	-0.00001	0.00002	-0.00001
639	C41	S1	-0.00000	0.00000	-0.00000	-0.00000	-0.00450
640	C41	S	0.00000	-0.00001	0.00000	-0.00001	-0.00022
641	C41	PX	-0.00000	0.00000	0.00000	-0.00000	0.00007
642	C41	PY	0.00000	-0.00000	0.00000	0.00000	-0.00005
643	C41	PZ	-0.00000	-0.00000	0.00000	0.00001	-0.00007
644	C41	S'	-0.00014	0.00017	0.00006	-0.00015	-0.00420
645	C41	PX'	-0.00002	-0.00004	-0.00001	0.00009	0.00019
646	C41	PY'	-0.00001	0.00000	0.00004	-0.00013	-0.00070
647	C41	PZ'	0.00010	0.00013	-0.00010	0.00005	0.00139
648	C41	DXX	-0.00000	-0.00000	0.00000	-0.00000	-0.00001
649	C41	DYY	0.00000	0.00000	-0.00000	0.00000	-0.00002
650	C41	DZZ	-0.00000	0.00000	0.00000	0.00001	-0.00004
651	C41	DXY	-0.00000	-0.00000	0.00000	-0.00000	0.00005
652	C41	DXZ	0.00000	0.00000	-0.00000	0.00000	0.00003
653	C41	DYZ	-0.00000	-0.00000	0.00000	-0.00001	-0.00001
654	C42	S1	0.00000	-0.00000	-0.00002	-0.00146	0.93510
655	C42	S	0.00000	-0.00000	-0.00001	-0.00007	0.04684
656	C42	PX	-0.00000	0.00000	0.00000	-0.00000	0.00017
657	C42	PY	0.00000	-0.00000	-0.00000	0.00001	-0.00024
658	C42	PZ	-0.00000	-0.00000	0.00000	-0.00003	0.00005
659	C42	S'	-0.00009	0.00019	0.00004	-0.00082	-0.01643
660	C42	PX'	-0.00005	0.00007	0.00004	-0.00043	-0.00285
661	C42	PY'	0.00005	-0.00007	-0.00003	0.00026	0.00324
662	C42	PZ'	0.00003	-0.00002	-0.00003	0.00074	0.00111
663	C42	DXX	-0.00000	0.00000	0.00000	0.00002	-0.00893
664	C42	DYY	-0.00000	0.00000	-0.00000	0.00003	-0.00888
665	C42	DZZ	0.00000	-0.00000	-0.00000	0.00005	-0.00876
666	C42	DXY	0.00000	-0.00000	-0.00000	0.00001	-0.00007
667	C42	DXZ	-0.00000	0.00000	0.00000	0.00001	-0.00002
668	C42	DYZ	-0.00000	0.00000	-0.00000	-0.00002	-0.00008
669	H23	S	0.00000	0.00000	0.00000	-0.00001	0.00003
670	H23	S'	0.00004	-0.00013	-0.00001	0.00004	0.00024
671	H25	S	-0.00000	0.00000	0.00000	-0.00006	-0.00046
672	H25	S'	-0.00002	0.00002	0.00002	-0.00017	0.00068
673	H26	S	-0.00000	-0.00000	0.00000	-0.00001	-0.00011
674	H26	S'	-0.00001	-0.00002	0.00001	-0.00001	0.00095

675	C43	S1	-0.00000	-0.00000	-0.00000	0.02538	0.00008
676	C43	S	-0.00000	-0.00000	-0.00000	0.00144	0.00000
677	C43	PX	-0.00000	0.00000	0.00000	0.00001	-0.00000
678	C43	PY	0.00000	-0.00000	0.00000	0.00014	-0.00000
679	C43	PZ	-0.00000	0.00000	-0.00000	-0.00025	0.00001
680	C43	S'	0.00035	-0.00013	-0.00012	-0.00249	-0.00008
681	C43	PX'	-0.00006	0.00001	0.00004	-0.00030	0.00005
682	C43	PY'	0.00026	-0.00005	-0.00015	-0.00061	-0.00011
683	C43	PZ'	-0.00036	0.00007	0.00019	0.00159	0.00014
684	C43	DXX	-0.00000	-0.00000	-0.00000	-0.00029	-0.00000
685	C43	DYY	-0.00000	-0.00000	0.00000	-0.00012	-0.00000
686	C43	DZZ	0.00000	-0.00000	-0.00000	0.00007	-0.00001
687	C43	DXY	0.00000	-0.00000	-0.00000	-0.00002	0.00000
688	C43	DXZ	-0.00000	0.00000	0.00000	0.00006	-0.00000
689	C43	DYZ	0.00000	0.00000	-0.00000	-0.00028	0.00001
690	C44	S1	0.00000	0.00000	0.00001	0.15998	0.00048
691	C44	S	0.00000	0.00000	0.00000	0.00732	0.00003
692	C44	PX	0.00000	0.00000	0.00000	-0.00011	0.00000
693	C44	PY	-0.00000	-0.00000	-0.00000	-0.00024	0.00000
694	C44	PZ	0.00000	-0.00000	-0.00000	0.00066	-0.00001
695	C44	S'	-0.00001	-0.00012	0.00010	0.00473	0.00050
696	C44	PX'	0.00001	-0.00001	-0.00001	0.00057	-0.00003
697	C44	PY'	0.00030	-0.00010	-0.00010	-0.00055	-0.00000
698	C44	PZ'	-0.00058	0.00021	0.00022	-0.00012	0.00001
699	C44	DXX	0.00000	0.00000	0.00000	-0.00195	-0.00000
700	C44	DYY	0.00000	0.00000	0.00000	-0.00217	0.00001
701	C44	DZZ	0.00000	-0.00000	0.00000	-0.00271	0.00002
702	C44	DXY	0.00000	0.00000	0.00000	-0.00002	-0.00000
703	C44	DXZ	-0.00000	0.00000	-0.00000	0.00001	0.00000
704	C44	DYZ	0.00000	0.00000	0.00000	0.00047	-0.00002
705	C45	S1	0.00000	0.00000	-0.00001	0.22572	-0.00041
706	C45	S	-0.00000	0.00000	-0.00000	0.01000	-0.00014
707	C45	PX	-0.00000	-0.00000	-0.00000	0.00022	0.00002
708	C45	PY	0.00000	-0.00000	-0.00000	0.00020	-0.00003
709	C45	PZ	0.00000	-0.00000	-0.00000	-0.00088	0.00004
710	C45	S'	-0.00032	0.00008	0.00008	0.00344	0.00085
711	C45	PX'	0.00018	-0.00013	-0.00001	0.00029	0.00074
712	C45	PY'	0.00011	-0.00005	-0.00003	-0.00056	0.00037
713	C45	PZ'	-0.00065	0.00044	0.00012	0.00037	-0.00251

714 C45	DXX	-0.00000	0.00000	-0.00000	-0.00259	0.00001
715 C45	DYY	-0.00000	0.00000	-0.00000	-0.00253	0.00002
716 C45	DZZ	0.00000	-0.00000	-0.00000	-0.00228	0.00015
717 C45	DXY	-0.00000	0.00000	-0.00000	0.00002	-0.00003
718 C45	DXZ	-0.00000	0.00000	-0.00000	-0.00001	-0.00005
719 C45	DYZ	0.00000	-0.00000	0.00000	-0.00015	-0.00004
720 C46	S1	0.00001	-0.00001	-0.00001	0.95262	0.00153
721 C46	S	0.00000	-0.00000	-0.00000	0.04689	0.00004
722 C46	PX	0.00000	-0.00000	0.00000	0.00004	-0.00001
723 C46	PY	-0.00000	0.00000	0.00000	-0.00019	-0.00001
724 C46	PZ	0.00000	-0.00000	0.00000	0.00034	0.00003
725 C46	S'	0.00020	-0.00020	0.00003	-0.00942	0.00180
726 C46	PX'	0.00011	-0.00006	-0.00003	-0.00062	0.00021
727 C46	PY'	0.00025	-0.00014	-0.00006	0.00072	0.00038
728 C46	PZ'	-0.00068	0.00035	0.00016	-0.00008	-0.00100
729 C46	DXX	0.00000	-0.00000	-0.00000	-0.00979	-0.00001
730 C46	DYY	0.00000	-0.00000	0.00000	-0.00959	-0.00001
731 C46	DZZ	0.00000	-0.00000	0.00000	-0.00777	-0.00006
732 C46	DXY	0.00000	-0.00000	-0.00000	0.00014	-0.00000
733 C46	DXZ	-0.00000	0.00000	0.00000	-0.00039	0.00002
734 C46	DYZ	0.00000	0.00000	-0.00000	-0.00084	0.00002
735 C47	S1	-0.00000	0.00000	-0.00000	-0.00001	-0.00034
736 C47	S	-0.00000	0.00000	-0.00000	0.00001	-0.00001
737 C47	PX	0.00000	-0.00000	-0.00000	0.00000	0.00002
738 C47	PY	0.00000	-0.00000	-0.00000	-0.00000	-0.00002
739 C47	PZ	-0.00000	0.00000	0.00000	-0.00000	-0.00004
740 C47	S'	-0.00014	0.00016	0.00025	-0.00080	-0.00662
741 C47	PX'	-0.00007	0.00001	0.00009	-0.00024	-0.00255
742 C47	PY'	0.00005	-0.00000	-0.00007	0.00022	0.00206
743 C47	PZ'	0.00021	-0.00003	-0.00022	0.00048	0.00633
744 C47	DXX	-0.00000	0.00000	-0.00000	0.00000	0.00000
745 C47	DYY	-0.00000	0.00000	-0.00000	-0.00000	-0.00001
746 C47	DZZ	0.00000	0.00000	0.00000	-0.00001	0.00001
747 C47	DXY	0.00000	0.00000	-0.00000	0.00000	0.00000
748 C47	DXZ	-0.00000	-0.00000	-0.00000	0.00000	-0.00001
749 C47	DYZ	-0.00000	0.00000	0.00000	-0.00000	-0.00001
750 C48	S1	0.00000	-0.00001	-0.00001	-0.00002	-0.00046
751 C48	S	0.00000	-0.00000	-0.00000	-0.00000	-0.00006
752 C48	PX	0.00000	0.00000	-0.00000	-0.00000	0.00001

753	C48	PY	-0.00000	0.00000	0.00000	0.00000	-0.00001
754	C48	PZ	-0.00000	-0.00000	0.00000	-0.00000	-0.00003
755	C48	S'	0.00006	0.00017	-0.00003	0.00015	0.00297
756	C48	PX'	-0.00010	0.00008	0.00014	-0.00033	-0.00334
757	C48	PY'	0.00011	-0.00010	-0.00015	0.00034	0.00351
758	C48	PZ'	0.00022	-0.00018	-0.00032	0.00082	0.00765
759	C48	DXX	0.00000	-0.00000	0.00000	-0.00000	0.00000
760	C48	DYY	0.00000	-0.00000	0.00000	-0.00000	-0.00000
761	C48	DZZ	0.00000	-0.00000	-0.00000	0.00001	0.00002
762	C48	DXY	0.00000	-0.00000	-0.00000	0.00000	0.00000
763	C48	DXZ	0.00000	-0.00000	-0.00000	-0.00000	-0.00001
764	C48	DYZ	-0.00000	0.00000	0.00000	0.00000	-0.00000
765	C49	S1	0.00000	-0.00000	-0.00000	0.00000	0.00001
766	C49	S	0.00000	-0.00000	0.00000	-0.00000	-0.00002
767	C49	PX	0.00000	0.00000	-0.00000	-0.00000	-0.00000
768	C49	PY	-0.00000	0.00000	0.00000	0.00000	-0.00000
769	C49	PZ	0.00000	0.00000	0.00000	0.00000	0.00000
770	C49	S'	0.00011	-0.00016	-0.00021	0.00055	0.00408
771	C49	PX'	-0.00006	0.00017	0.00012	-0.00020	-0.00140
772	C49	PY'	0.00010	-0.00029	-0.00018	0.00029	0.00204
773	C49	PZ'	0.00009	-0.00026	-0.00019	0.00033	0.00214
774	C49	DXX	0.00000	-0.00000	-0.00000	-0.00000	0.00000
775	C49	DYY	-0.00000	0.00000	0.00000	0.00000	0.00000
776	C49	DZZ	0.00000	-0.00000	-0.00000	0.00000	0.00000
777	C49	DXY	0.00000	-0.00000	-0.00000	-0.00000	-0.00000
778	C49	DXZ	0.00000	0.00000	0.00000	-0.00000	-0.00000
779	C49	DYZ	-0.00000	0.00000	-0.00000	0.00000	0.00000
780	C50	S1	-0.00000	0.00001	-0.00000	0.00001	0.00001
781	C50	S	-0.00000	0.00000	0.00000	0.00000	-0.00002
782	C50	PX	0.00000	-0.00000	-0.00000	0.00000	0.00001
783	C50	PY	0.00000	-0.00000	0.00000	-0.00000	-0.00001
784	C50	PZ	-0.00000	0.00000	0.00000	-0.00000	-0.00000
785	C50	S'	0.00002	0.00019	0.00004	0.00005	0.00172
786	C50	PX'	-0.00009	0.00017	0.00015	-0.00034	-0.00267
787	C50	PY'	0.00011	-0.00018	-0.00019	0.00044	0.00331
788	C50	PZ'	0.00018	-0.00031	-0.00029	0.00062	0.00498
789	C50	DXX	-0.00000	0.00000	0.00000	0.00000	0.00000
790	C50	DYY	-0.00000	0.00000	0.00000	-0.00000	0.00000
791	C50	DZZ	0.00000	0.00000	-0.00000	0.00000	0.00002

792 C50	DXY	-0.00000	-0.00000	-0.00000	0.00000	-0.00000
793 C50	DXZ	-0.00000	-0.00000	0.00000	0.00000	-0.00001
794 C50	DYZ	0.00000	0.00000	0.00000	-0.00000	0.00001

MO:	16	17	18	19	20
Eigenvalues:	-10.20856	-10.20810	-10.20751	-10.20728	-10.20646
(ev)	-277.78930	-277.77653	-277.76047	-277.75432	-277.73213

		A	A	A	A	A
1 C1	S1	0.00000	-0.00000	-0.00000	-0.00000	0.00000
2 C1	S	-0.00001	0.00000	0.00000	0.00000	-0.00000
3 C1	PX	-0.00000	-0.00000	0.00000	-0.00000	0.00000
4 C1	PY	0.00000	0.00000	-0.00000	0.00000	-0.00000
5 C1	PZ	0.00000	-0.00000	0.00000	-0.00000	0.00000
6 C1	S'	0.00009	-0.00003	-0.00003	-0.00006	0.00003
7 C1	PX'	0.00004	0.00000	-0.00003	0.00007	-0.00003
8 C1	PY'	0.00004	0.00001	0.00003	-0.00002	0.00002
9 C1	PZ'	0.00008	0.00001	-0.00003	0.00009	-0.00004
10 C1	DXX	-0.00000	0.00000	0.00000	0.00000	-0.00000
11 C1	DYY	0.00000	0.00000	0.00000	0.00000	-0.00000
12 C1	DZZ	-0.00000	0.00000	-0.00000	-0.00000	0.00000
13 C1	DXY	-0.00000	0.00000	0.00000	0.00000	-0.00000
14 C1	DXZ	0.00000	-0.00000	-0.00000	-0.00000	0.00000
15 C1	DYZ	0.00000	0.00000	0.00000	-0.00000	0.00000
16 C4	S1	0.00001	-0.00000	-0.00001	-0.00000	0.00000
17 C4	S	-0.00000	0.00000	0.00000	0.00000	-0.00000
18 C4	PX	0.00000	-0.00000	0.00000	0.00000	0.00000
19 C4	PY	0.00000	0.00000	0.00000	-0.00000	0.00000
20 C4	PZ	0.00000	-0.00000	0.00000	-0.00000	0.00000
21 C4	S'	0.00001	0.00001	-0.00000	-0.00001	0.00001
22 C4	PX'	-0.00004	0.00001	0.00000	0.00000	-0.00000
23 C4	PY'	0.00002	-0.00000	-0.00009	0.00000	0.00000
24 C4	PZ'	-0.00003	0.00001	-0.00005	0.00001	-0.00001
25 C4	DXX	-0.00000	0.00000	0.00000	0.00000	-0.00000
26 C4	DYY	0.00000	-0.00000	-0.00000	-0.00000	0.00000
27 C4	DZZ	-0.00000	0.00000	0.00000	0.00000	-0.00000
28 C4	DXY	-0.00000	0.00000	0.00000	-0.00000	-0.00000
29 C4	DXZ	0.00000	0.00000	-0.00000	0.00000	-0.00000
30 C4	DYZ	-0.00000	-0.00000	0.00000	-0.00000	0.00000
31 C2	S1	0.00000	0.00000	-0.00000	0.00000	-0.00000

32	C2	S	0.00000	0.00000	-0.00000	0.00000	-0.00000
33	C2	PX	0.00000	-0.00000	0.00000	0.00000	-0.00000
34	C2	PY	-0.00000	0.00000	0.00000	-0.00000	0.00000
35	C2	PZ	0.00000	0.00000	-0.00000	0.00000	0.00000
36	C2	S'	-0.00002	-0.00000	0.00004	-0.00005	0.00003
37	C2	PX'	-0.00000	-0.00001	-0.00001	-0.00000	-0.00000
38	C2	PY'	-0.00002	0.00000	0.00003	-0.00002	0.00001
39	C2	PZ'	-0.00002	-0.00002	0.00001	-0.00002	0.00001
40	C2	DXX	0.00000	0.00000	-0.00000	0.00000	-0.00000
41	C2	DYY	-0.00000	0.00000	0.00000	-0.00000	0.00000
42	C2	DZZ	0.00000	-0.00000	-0.00000	0.00000	-0.00000
43	C2	DXY	0.00000	0.00000	-0.00000	0.00000	-0.00000
44	C2	DXZ	0.00000	-0.00000	0.00000	-0.00000	0.00000
45	C2	DYZ	-0.00000	-0.00000	-0.00000	-0.00000	0.00000
46	C6	S1	-0.00000	0.00000	-0.00000	-0.00000	-0.00000
47	C6	S	0.00000	0.00001	-0.00001	-0.00000	0.00000
48	C6	PX	-0.00000	-0.00000	0.00000	0.00000	-0.00000
49	C6	PY	0.00000	0.00000	-0.00000	-0.00000	0.00000
50	C6	PZ	-0.00001	-0.00000	0.00000	-0.00000	-0.00000
51	C6	S'	0.00016	-0.00007	-0.00004	0.00003	-0.00002
52	C6	PX'	-0.00004	0.00004	0.00005	-0.00005	0.00003
53	C6	PY'	0.00014	-0.00005	-0.00008	0.00007	-0.00003
54	C6	PZ'	0.00012	0.00002	0.00006	-0.00002	0.00002
55	C6	DXX	0.00000	0.00000	-0.00000	0.00000	0.00000
56	C6	DYY	-0.00000	0.00000	-0.00000	0.00000	-0.00000
57	C6	DZZ	0.00000	0.00000	0.00000	0.00000	-0.00000
58	C6	DXY	-0.00000	-0.00000	-0.00000	-0.00000	0.00000
59	C6	DXZ	0.00000	-0.00000	0.00000	0.00000	-0.00000
60	C6	DYZ	-0.00000	-0.00000	-0.00000	-0.00000	0.00000
61	C5	S1	0.00000	-0.00000	0.00001	-0.00000	0.00000
62	C5	S	0.00000	0.00000	-0.00000	-0.00000	0.00000
63	C5	PX	0.00000	-0.00000	-0.00001	-0.00000	0.00000
64	C5	PY	-0.00000	0.00000	-0.00000	-0.00000	0.00000
65	C5	PZ	0.00000	-0.00000	0.00000	-0.00000	0.00000
66	C5	S'	-0.00011	-0.00001	0.00003	0.00003	-0.00002
67	C5	PX'	0.00002	-0.00000	0.00009	-0.00000	0.00000
68	C5	PY'	0.00001	0.00001	0.00001	-0.00001	0.00001
69	C5	PZ'	0.00002	0.00001	0.00008	-0.00001	0.00001
70	C5	DXX	-0.00000	0.00000	0.00000	-0.00000	0.00000

AbridgedAbridged***Abridged***

650	C41	DZZ	0.40561	0.29690	0.06256	0.63356
651	C41	DXY	-0.05318	-0.01856	-0.00202	-0.00007
652	C41	DXZ	-0.03451	-0.03908	-0.01273	-0.10608
653	C41	DYZ	-0.00393	-0.00422	-0.00890	-0.01860
654	C42	S1	0.06296	0.01519	-0.02226	0.01381
655	C42	S	-0.30516	-0.06992	0.11237	-0.06908
656	C42	PX	0.02855	0.00582	-0.01854	0.00599
657	C42	PY	-0.03316	-0.00541	0.02355	-0.00370
658	C42	PZ	-0.00766	-0.00526	-0.00191	-0.00672
659	C42	S'	-1.71586	-0.45237	0.51735	-0.58800
660	C42	PX'	-0.45307	-0.12405	0.19076	-0.16959
661	C42	PY'	0.48189	0.12518	-0.16781	0.16430
662	C42	PZ'	0.27296	0.09050	-0.21017	0.13684
663	C42	DXX	0.23551	0.05498	-0.09533	0.05508
664	C42	DYY	0.27103	0.06045	-0.12037	0.06047
665	C42	DZZ	0.30855	0.07700	-0.09088	0.07039
666	C42	DXY	-0.05193	-0.00908	0.04799	-0.01943
667	C42	DXZ	0.00023	-0.00396	-0.00601	-0.01124
668	C42	DYZ	-0.05136	-0.00851	0.02050	0.00005
669	H23	S	0.08957	0.01866	-0.01474	0.04212
670	H23	S'	0.16082	0.07385	-0.04046	0.12844
671	H25	S	-0.04220	-0.01033	0.03663	-0.01702
672	H25	S'	-0.07305	-0.02458	0.05654	-0.04074
673	H26	S	0.04526	0.01876	-0.00693	0.03350
674	H26	S'	0.12240	0.03220	-0.02985	0.06335
675	C43	S1	0.05405	0.01018	0.15880	-0.03199
676	C43	S	-0.11657	-0.03292	0.12277	-0.03168
677	C43	PX	0.00538	0.00177	-0.00608	0.00134
678	C43	PY	0.08901	0.01094	0.47235	-0.09674
679	C43	PZ	-0.16424	-0.02213	-0.81302	0.16631
680	C43	S'	-0.33070	-0.01013	-2.17130	0.60181
681	C43	PX'	-0.01523	-0.00664	-0.05974	0.00820
682	C43	PY'	-0.11302	-0.00819	-0.63024	0.17119
683	C43	PZ'	0.22421	0.02682	1.21998	-0.31646
684	C43	DXX	0.12682	0.02771	0.20851	-0.03981
685	C43	DYY	0.22933	0.04482	0.62892	-0.12521

686	C43	DZZ	0.31599	0.05852	1.01648	-0.20441
687	C43	DXY	-0.03581	-0.00707	-0.11723	0.02331
688	C43	DXZ	0.04860	0.00848	0.17340	-0.03500
689	C43	DYZ	-0.17149	-0.02913	-0.70173	0.14244
690	C44	S1	-0.07235	-0.00991	-0.29057	0.05884
691	C44	S	0.27570	0.03075	1.00265	-0.20198
692	C44	PX	-0.00951	-0.00073	-0.06892	0.01462
693	C44	PY	0.05840	0.01112	0.09272	-0.01761
694	C44	PZ	-0.08351	-0.01789	-0.00805	-0.00199
695	C44	S'	0.93530	0.22042	1.48989	-0.15137
696	C44	PX'	0.03145	0.00615	0.17284	-0.03612
697	C44	PY'	0.00946	0.03761	-0.95714	0.31664
698	C44	PZ'	-0.07715	-0.08297	1.40677	-0.50854
699	C44	DXX	-0.18168	-0.02339	-0.64045	0.12860
700	C44	DYY	-0.26536	-0.03408	-1.12447	0.22878
701	C44	DZZ	-0.51337	-0.06814	-2.47910	0.50824
702	C44	DXY	-0.01140	-0.00186	-0.03894	0.00783
703	C44	DXZ	0.02115	0.00382	0.06820	-0.01344
704	C44	DYZ	0.19412	0.02582	1.09279	-0.22587
705	C45	S1	0.07261	0.01218	-0.18600	0.04426
706	C45	S	-0.54883	-0.08402	0.05977	-0.03855
707	C45	PX	0.04514	0.00494	0.22202	-0.04534
708	C45	PY	0.00328	0.00002	0.24084	-0.05163
709	C45	PZ	-0.12118	-0.01272	-0.88112	0.18093
710	C45	S'	-0.40376	-0.12918	2.40491	-0.63708
711	C45	PX'	0.31655	0.11064	-0.46731	0.26543
712	C45	PY'	0.18385	0.06670	-0.60154	0.23348
713	C45	PZ'	-1.02254	-0.37562	2.14705	-1.02922
714	C45	DXX	0.28907	0.04630	-0.38151	0.09779
715	C45	DYY	0.28552	0.04475	-0.29311	0.07696
716	C45	DZZ	0.51320	0.08790	-1.62219	0.38371
717	C45	DXY	-0.00109	0.00073	-0.08699	0.02054
718	C45	DXZ	-0.07275	-0.01431	0.47238	-0.10800
719	C45	DYZ	-0.05057	-0.00819	0.26584	-0.06011
720	C46	S1	0.03315	0.00292	0.29916	-0.06293
721	C46	S	-0.33443	-0.04331	-0.92054	0.17415
722	C46	PX	-0.02860	-0.00504	0.13759	-0.03171
723	C46	PY	-0.09244	-0.01460	-0.06206	0.00779
724	C46	PZ	0.22375	0.03625	-0.16295	0.04954

725	C46	S'	0.68853	0.24465	-2.45157	0.85279
726	C46	PX'	0.11979	0.04870	-0.53579	0.19187
727	C46	PY'	0.31960	0.11172	-0.51341	0.26612
728	C46	PZ'	-0.84467	-0.30284	2.05500	-0.88426
729	C46	DXX	0.13893	0.01582	0.68303	-0.13869
730	C46	DYY	0.15932	0.01731	0.91625	-0.18834
731	C46	DZZ	0.28910	0.02859	2.68779	-0.56311
732	C46	DXY	0.00858	0.00073	0.13990	-0.02994
733	C46	DXZ	-0.01552	-0.00099	-0.36493	0.07703
734	C46	DYZ	-0.06473	-0.00510	-0.88093	0.18712
735	C47	S1	-0.04483	-0.06453	-0.02762	-0.16566
736	C47	S	0.28974	0.17321	-0.05290	-0.09439
737	C47	PX	0.01240	-0.07674	-0.07825	-0.36128
738	C47	PY	-0.00380	0.07935	0.07477	0.35388
739	C47	PZ	-0.05633	0.16343	0.18138	0.81412
740	C47	S'	0.72024	0.50811	0.18399	2.62086
741	C47	PX'	0.30419	0.20383	-0.06145	0.78597
742	C47	PY'	-0.27595	-0.17636	0.02479	-0.74447
743	C47	PZ'	-0.65622	-0.50638	0.21311	-1.78165
744	C47	DXX	-0.16934	-0.18587	-0.04826	-0.34982
745	C47	DYY	-0.16337	-0.16693	-0.03588	-0.27647
746	C47	DZZ	-0.29173	-0.47195	-0.23640	-1.34252
747	C47	DXY	0.01264	0.03285	0.02725	0.12625
748	C47	DXZ	0.04391	0.12970	0.09424	0.48333
749	C47	DYZ	-0.01900	-0.09121	-0.07381	-0.36392
750	C48	S1	-0.01123	0.07585	0.06285	0.28975
751	C48	S	0.15745	-0.27384	-0.23375	-0.97608
752	C48	PX	-0.04984	-0.05139	0.00472	-0.01771
753	C48	PY	0.05692	0.04648	-0.01629	-0.02968
754	C48	PZ	0.10575	0.12302	0.00605	0.10527
755	C48	S'	-0.53657	-1.24739	-0.00582	-1.54626
756	C48	PX'	0.31363	0.07622	-0.06061	0.93270
757	C48	PY'	-0.32140	-0.08207	0.08775	-0.86857
758	C48	PZ'	-0.75679	-0.16767	0.08624	-2.31159
759	C48	DXX	-0.06263	0.25101	0.21423	0.96534
760	C48	DYY	-0.05654	0.26345	0.22178	1.00784
761	C48	DZZ	-0.12239	0.48609	0.50035	2.28519
762	C48	DXY	0.00384	-0.08514	-0.08830	-0.42022
763	C48	DXZ	0.02726	-0.16489	-0.18685	-0.87222

764	C48	DYZ	-0.02275	0.18035	0.19733	0.92832
765	C49	S1	-0.02950	-0.09341	0.04170	0.18075
766	C49	S	0.08538	0.61724	-0.01217	-0.01337
767	C49	PX	0.03980	-0.04966	-0.09090	-0.40785
768	C49	PY	-0.05216	0.09324	0.12160	0.54778
769	C49	PZ	-0.06816	0.05338	0.15220	0.67923
770	C49	S'	-0.05271	0.82217	-0.36458	-2.76208
771	C49	PX'	-0.01728	-0.62522	0.17842	1.18722
772	C49	PY'	0.01900	0.83755	-0.23216	-1.61074
773	C49	PZ'	0.02584	1.02124	-0.31043	-1.96078
774	C49	DXX	-0.10033	-0.38907	0.12253	0.52264
775	C49	DYY	-0.13840	-0.45938	0.19344	0.83817
776	C49	DZZ	-0.14752	-0.47243	0.19934	0.86209
777	C49	DXY	0.05789	0.09680	-0.10820	-0.48009
778	C49	DXZ	0.06142	0.10194	-0.11062	-0.48986
779	C49	DYZ	-0.08842	-0.14399	0.16229	0.71920
780	C50	S1	0.03465	-0.02303	-0.06709	-0.29567
781	C50	S	-0.12054	0.34014	0.20790	0.92279
782	C50	PX	0.02898	0.11229	-0.02165	-0.08614
783	C50	PY	-0.04069	-0.13746	0.04046	0.16854
784	C50	PZ	-0.04775	-0.21839	0.01321	0.03707
785	C50	S'	-0.55759	-1.32768	0.69646	2.27921
786	C50	PX'	0.12521	-0.46565	0.10100	1.24959
787	C50	PY'	-0.15256	0.61361	-0.15215	-1.66945
788	C50	PZ'	-0.22400	0.83519	-0.14067	-2.15238
789	C50	DXX	0.11673	-0.14116	-0.22875	-1.01207
790	C50	DYY	0.13820	-0.15368	-0.27410	-1.21398
791	C50	DZZ	0.20294	-0.21225	-0.45849	-2.06002
792	C50	DXY	-0.05231	0.03531	0.12387	0.55704
793	C50	DXZ	-0.07648	0.05536	0.19180	0.86781
794	C50	DYZ	0.09664	-0.07202	-0.23744	-1.07166

Standard Thermodynamic quantities at 298.15 K and 1.00 atm

*Modifying values for 28 low frequency terms

Enthalpy

Term	ZPE kJ/mol	Correction kJ/mol	Entropy J/mol.K	Cv J/mol.K	% in Ground
--	----------	-------	-------	-------	----
Total Vibrations	1402.5877	75.9135	621.8095	622.8227	-Unscaled-
Ideal Gas		2.4789			
Translation		3.7184	188.9896	12.4716	
Rotation		3.7184	166.2264	12.4716	
----------		-------	-------	-------	
Totals		1488.4169	977.0255	647.7660	

Vibrational(v) Corrections:

Temp. Correction Hv 1488.4169

Entropy Correction (Hv-TSv) 1197.1168

Reason for exit: Successful completion

Properties CPU Time : 9.03

Properties Wall Time: 9.77

6.1.2 Compound 1b

SPARTAN '18 MECHANICS PROGRAM: (Linux/P4E) build 18.3.0

Frequency Calculation

Adjusted 10 (out of 216) low frequency modes

Reason for exit: Successful completion

Mechanics CPU Time : .11

Mechanics Wall Time: .12

SPARTAN '18 Quantum Mechanics Program: (Linux/P4E) build 18.3.0

Job type: Geometry optimization.

Method: RB3LYP

Basis set: 6-31G(D)

Number of basis functions: 794

Number of electrons: 322

SCF model:

A restricted hybrid HF-DFT SCF calculation will be

performed using Pulay DIIS + Geometric Direct Minimization

Optimization:

Step	Energy	Max Grad.	Max Dist.
1	-1918.174746	0.050161	0.074213
2	-1918.187781	0.007012	0.094910
3	-1918.189785	0.005907	0.118284
4	-1918.190671	0.005505	0.096809
5	-1918.191364	0.005356	0.082112
6	-1918.192201	0.005636	0.093187
7	-1918.193140	0.006107	0.138891
8	-1918.194232	0.006179	0.143081
9	-1918.195242	0.005273	0.136850
10	-1918.196119	0.004068	0.128126
11	-1918.196726	0.002443	0.126422
12	-1918.197036	0.001849	0.105443
13	-1918.197140	0.001206	0.037484
14	-1918.197262	0.000650	0.069107
15	-1918.197313	0.001059	0.106741
16	-1918.197344	0.001073	0.088038
17	-1918.197357	0.000752	0.067235
18	-1918.197364	0.000476	0.025970

<step 2>

Job type: Frequency calculation.

Method: RB3LYP

Basis set: 6-31G(D)

<step 3>

Job type: Single point.

Method: RB3LYP

Basis set: 6-31G(D)

SCF total energy: -1918.1973639 hartrees

Warning: NMR reference data missing

NMR shifts (ppm)

	Atom	Isotropic	Rel. Shift
1	C1	45.7726	
2	C4	54.7607	
3	C2	65.0708	
4	C6	72.0942	
5	C5	65.5250	
6	C3	65.2305	
7	H2	24.9466	
8	H5	23.8836	
9	H3	24.8272	
10	C7	54.6383	
11	C8	69.3854	
12	C9	74.1817	
13	C10	63.2003	
14	C11	68.0698	
15	C12	57.9023	
16	H7	24.8942	
17	H8	24.9809	
18	H9	24.6288	
19	H10	25.0377	
20	C13	52.5337	
21	C14	60.6631	
22	C15	65.5741	
23	C16	63.3462	
24	C17	68.5640	
25	C18	70.8520	
26	H1	24.6674	
27	H11	24.8179	
28	H14	24.4755	
29	C19	96.5779	
30	C20	103.2398	
31	C21	95.7451	
32	C22	97.6167	
33	C23	101.4041	

34	C24	98.2590
35	C25	63.4680
36	C26	69.2894
37	C27	67.5896
38	C28	64.8848
39	C29	68.4964
40	C30	65.6588
41	H15	24.9235
42	H16	25.0687
43	H17	25.0565
44	H18	25.1103
45	C31	71.3846
46	C32	70.2014
47	C33	66.1641
48	C34	48.1620
49	C35	70.8100
50	C36	67.8910
51	H4	25.0730
52	H19	24.1458
53	H21	25.0860
54	H22	25.3894
55	C37	63.8697
56	C38	68.9495
57	C39	73.8922
58	C40	48.4012
59	C41	72.4918
60	C42	66.0075
61	H23	26.1537
62	H25	25.3829
63	H26	25.5407
64	C43	98.2500
65	C44	109.5568
66	C45	101.8364
67	C46	112.4941
68	C47	107.5362
69	C48	111.7882
70	C49	106.4129
71	C50	111.2909
72	H24	24.8934

Reason for exit: Successful completion

Quantum Calculation CPU Time : 28:49:18.07

Quantum Calculation Wall Time: 31:25:58.75

SPARTAN '18 Quantum Mechanics Program: (Linux/P4E)   build 18.3.0

Job type: Single point.

Excited States: 6

Method: RB3LYP

Basis set: 6-31G(D)

Number of basis functions: 794

Number of electrons: 322

SCF model:

A restricted hybrid HF-DFT SCF calculation will be performed using Pulay DIIS + Geometric Direct Minimization

SCF total energy: -1918.1973635 hartrees

Reason for exit: Successful completion

Quantum Calculation CPU Time :   58:24.71

Quantum Calculation Wall Time: 58:40.95

SPARTAN '18 Properties Program: (Linux/P4E)   build 18.3.0

Use of molecular symmetry disabled

Cartesian Coordinates (Angstroms)

Atom		X	Y	Z
1 C	C1	1.4633971	-1.0643805	0.1088876
2 C	C4	1.5087113	-2.2456067	2.7036707
3 C	C2	1.8610447	-2.3897621	0.2919834
4 C	C6	0.9305479	-0.3630714	1.2305961
5 C	C5	0.9876723	-0.9532012	2.5130287

6 C	C3	1.8644849	-2.9738038	1.5513691
7 H	H2	2.2222581	-2.9547531	-0.5630133
8 H	H5	0.6402191	-0.3780345	3.3640348
9 H	H3	2.1805818	-4.0068695	1.6502325
10 C	C7	1.7980739	-2.8841264	4.0250212
11 C	C8	2.5197926	-4.3183433	6.3773566
12 C	C9	1.0093777	-2.7897416	5.2042223
13 C	C10	2.9665633	-3.6701107	4.1009145
14 C	C11	3.3290858	-4.3768261	5.2421779
15 C	C12	1.3824385	-3.5256617	6.3526098
16 H	H7	3.6238933	-3.7046150	3.2387729
17 H	H8	4.2436784	-4.9631575	5.2448273
18 H	H9	0.7519580	-3.4448097	7.2321826
19 H	H10	2.7811143	-4.8680227	7.2771324
20 C	C13	1.7096915	-0.4108317	-1.2064305
21 C	C14	2.3770777	0.7938774	-3.6590180
22 C	C15	1.2335648	-0.9489142	-2.4089233
23 C	C16	2.5277602	0.7314501	-1.2505894
24 C	C17	2.8608909	1.3228044	-2.4669208
25 C	C18	1.5515214	-0.3481911	-3.6427396
26 H	H1	0.5917478	-1.8247274	-2.3939273
27 H	H11	2.9112533	1.1456626	-0.3234922
28 H	H14	2.6196828	1.2556374	-4.6103465
29 C	C19	1.0283831	-0.8165661	-4.8870620
30 C	C20	0.6195360	-1.0492556	-6.0088934
31 C	C21	0.3088499	0.9026928	0.9718330
32 C	C22	-0.2320215	1.8918702	0.5045905
33 C	C23	-0.1343441	-1.9551181	5.3716302
34 C	C24	-1.0385675	-1.2504278	5.7821778
35 C	C25	0.0891021	-1.1457460	-7.3276494
36 C	C26	-0.9273004	-1.2581867	-9.9509390
37 C	C27	-0.5951036	-0.0186574	-7.8873215
38 C	C28	0.2348555	-2.3053168	-8.1044162
39 C	C29	-0.2687272	-2.3625232	-9.4020126
40 C	C30	-1.0897244	-0.0974823	-9.2002383
41 H	H15	0.7522307	-3.1584938	-7.6766110
42 H	H16	-0.1453051	-3.2692763	-9.9874578
43 H	H17	-1.6073343	0.7609287	-9.6169920
44 H	H18	-1.3170622	-1.3038292	-10.9638783

45	C	C31	-1.9836078	-0.2666647	6.1924112
46	C	C32	-3.6946987	1.8339628	6.9816846
47	C	C33	-2.7734755	-0.3852641	7.3547049
48	C	C34	-2.0765507	0.9040974	5.4339510
49	C	C35	-2.9213320	1.9613874	5.8160244
50	C	C36	-3.6155973	0.6598502	7.7335774
51	H	H4	-2.7134385	-1.2885412	7.9535669
52	H	H19	-1.4808876	1.0071478	4.5344668
53	H	H21	-4.2181534	0.5601012	8.6320437
54	H	H22	-4.3505887	2.6433194	7.2862951
55	C	C37	-0.7727866	2.9223367	-0.3266484
56	C	C38	-1.7775001	4.8383451	-2.1575259
57	C	C39	-1.4902686	4.0663356	0.1406586
58	C	C40	-0.6167488	2.7549455	-1.7100044
59	C	C41	-1.1382949	3.6766393	-2.6364134
60	C	C42	-1.9464531	5.0200172	-0.7937077
61	H	H23	-0.1154212	1.8677194	-2.0770731
62	H	H25	-2.4773042	5.8915175	-0.4241645
63	H	H26	-2.1693843	5.5662565	-2.8605385
64	C	C43	-2.9055835	3.0697324	4.9203203
65	C	C44	-2.6119756	3.6612611	3.8901832
66	C	C45	-1.8410683	4.1669939	1.5041783
67	C	C46	-2.2305063	4.0844471	2.6606115
68	C	C47	-1.0600177	3.3294437	-4.0118869
69	C	C48	-0.9794691	2.7489948	-5.0854532
70	C	C49	-0.7813249	1.1297994	-7.0757448
71	C	C50	-0.8825059	1.9677292	-6.1911126
72	H	H24	3.4993933	2.2016071	-2.4847838

Point Group = C1 Order = 1 Nsymop = 1

Closed-Shell Molecular Orbital Coefficients

MO:		1	2	3	4	5
Eigenvalues:		-10.23106	-10.23059	-10.22832	-10.22784	-10.22733
(ev)		-278.40136	-278.38865	-278.32677	-278.31384	-278.29994
		A	A	A	A	A
1 C1	S1	0.00000	0.00000	0.00000	0.00000	0.00000
2 C1	S	0.00003	0.00000	0.00001	0.00001	0.00002

3	C1	PX	0.00001	-0.00000	-0.00000	0.00000	0.00000
4	C1	PY	-0.00002	0.00001	0.00000	0.00000	-0.00000
5	C1	PZ	0.00001	-0.00000	-0.00000	-0.00000	0.00000
6	C1	S'	-0.00056	0.00007	-0.00014	-0.00020	-0.00037
7	C1	PX'	0.00009	0.00000	-0.00003	-0.00004	0.00011
8	C1	PY'	0.00012	-0.00018	-0.00017	-0.00021	-0.00018
9	C1	PZ'	-0.00059	0.00012	0.00023	0.00026	-0.00020
10	C1	DXX	0.00000	0.00000	0.00000	0.00000	0.00000
11	C1	DYY	-0.00001	0.00001	-0.00000	-0.00000	-0.00000
12	C1	DZZ	-0.00000	-0.00000	-0.00000	-0.00000	-0.00000
13	C1	DXY	0.00001	-0.00000	-0.00000	-0.00000	0.00000
14	C1	DXZ	0.00000	-0.00000	0.00000	0.00000	0.00000
15	C1	DYZ	-0.00000	0.00000	0.00000	0.00001	-0.00000
16	C4	S1	0.00001	0.00000	0.00000	0.00000	0.00001
17	C4	S	0.00002	0.00000	0.00000	0.00000	0.00001
18	C4	PX	-0.00000	0.00000	-0.00000	-0.00000	-0.00000
19	C4	PY	0.00001	-0.00000	-0.00000	-0.00000	0.00000
20	C4	PZ	-0.00001	-0.00000	-0.00000	-0.00000	-0.00001
21	C4	S'	0.00003	-0.00008	-0.00002	-0.00004	-0.00009
22	C4	PX'	0.00011	-0.00001	0.00000	0.00001	0.00007
23	C4	PY'	-0.00029	0.00001	-0.00000	-0.00002	-0.00018
24	C4	PZ'	0.00025	0.00005	0.00003	0.00004	0.00020
25	C4	DXX	0.00001	-0.00000	0.00000	0.00000	0.00000
26	C4	DYY	-0.00001	0.00000	0.00000	-0.00000	-0.00001
27	C4	DZZ	0.00001	-0.00000	-0.00000	0.00000	0.00000
28	C4	DXY	0.00001	0.00000	0.00000	0.00000	0.00000
29	C4	DXZ	0.00000	0.00000	0.00000	0.00000	0.00000
30	C4	DYZ	0.00000	-0.00000	-0.00000	-0.00000	0.00000
31	C2	S1	-0.00000	0.00000	0.00000	0.00000	-0.00000
32	C2	S	-0.00001	0.00001	0.00001	0.00001	0.00000
33	C2	PX	-0.00000	0.00000	0.00000	0.00000	-0.00000
34	C2	PY	0.00001	-0.00000	-0.00000	-0.00000	0.00000
35	C2	PZ	-0.00000	0.00000	0.00000	0.00000	-0.00000
36	C2	S'	0.00021	-0.00020	-0.00013	-0.00013	-0.00009
37	C2	PX'	-0.00005	0.00004	0.00003	0.00004	0.00001
38	C2	PY'	0.00010	-0.00007	-0.00005	-0.00006	-0.00003
39	C2	PZ'	0.00014	-0.00006	-0.00006	-0.00006	0.00003
40	C2	DXX	-0.00000	0.00000	0.00000	0.00000	0.00000
41	C2	DYY	0.00001	-0.00000	-0.00000	0.00000	0.00000

42	C2	DZZ	-0.00000	-0.00000	0.00000	0.00000	-0.00000
43	C2	DXY	-0.00000	0.00000	0.00000	0.00000	-0.00000
44	C2	DXZ	0.00000	-0.00000	0.00000	0.00000	0.00000
45	C2	DYZ	0.00000	0.00000	-0.00000	-0.00000	0.00000
46	C6	S1	-0.00005	0.00004	0.00000	0.00001	-0.00006
47	C6	S	0.00004	-0.00001	0.00000	0.00000	-0.00000
48	C6	PX	0.00001	-0.00002	0.00000	0.00000	0.00000
49	C6	PY	-0.00003	0.00002	-0.00000	-0.00000	-0.00001
50	C6	PZ	0.00003	-0.00001	-0.00000	-0.00000	0.00002
51	C6	S'	-0.00125	-0.00024	0.00005	0.00002	-0.00081
52	C6	PX'	0.00057	0.00003	-0.00005	-0.00005	0.00029
53	C6	PY'	-0.00105	0.00008	0.00015	0.00018	-0.00043
54	C6	PZ'	-0.00052	0.00001	-0.00001	-0.00005	-0.00049
55	C6	DXX	-0.00001	0.00001	0.00000	0.00000	-0.00000
56	C6	DYY	-0.00006	0.00005	-0.00000	-0.00000	-0.00001
57	C6	DZZ	-0.00001	0.00001	0.00000	0.00000	-0.00000
58	C6	DXY	0.00003	-0.00002	-0.00000	-0.00000	0.00000
59	C6	DXZ	-0.00001	0.00000	-0.00000	-0.00000	-0.00000
60	C6	DYZ	0.00002	-0.00001	-0.00000	-0.00000	0.00000
61	C5	S1	0.00000	0.00000	0.00000	-0.00000	-0.00000
62	C5	S	-0.00002	0.00000	0.00000	-0.00000	-0.00002
63	C5	PX	-0.00001	0.00000	0.00000	-0.00000	-0.00001
64	C5	PY	0.00002	-0.00000	-0.00000	0.00000	0.00001
65	C5	PZ	-0.00001	0.00000	-0.00000	-0.00000	-0.00001
66	C5	S'	0.00051	0.00004	0.00004	0.00010	0.00049
67	C5	PX'	0.00001	0.00000	0.00001	0.00001	0.00003
68	C5	PY'	0.00000	-0.00004	-0.00001	-0.00003	-0.00002
69	C5	PZ'	-0.00007	-0.00004	-0.00003	-0.00005	-0.00015
70	C5	DXX	0.00000	0.00000	0.00000	0.00000	-0.00000
71	C5	DYY	0.00002	-0.00000	-0.00000	-0.00000	0.00001
72	C5	DZZ	0.00000	-0.00000	0.00000	0.00000	0.00000
73	C5	DXY	-0.00000	0.00000	0.00000	-0.00000	-0.00000
74	C5	DXZ	0.00001	0.00000	-0.00000	0.00000	0.00000
75	C5	DYZ	-0.00001	0.00000	0.00000	0.00000	-0.00001
76	C3	S1	0.00000	-0.00000	-0.00000	0.00000	-0.00000
77	C3	S	0.00001	-0.00001	-0.00000	-0.00000	-0.00000
78	C3	PX	-0.00000	0.00000	-0.00000	-0.00000	-0.00000
79	C3	PY	0.00000	0.00000	0.00000	0.00000	0.00000
80	C3	PZ	0.00001	-0.00000	-0.00000	-0.00000	0.00000

81	C3	S'	-0.00011	0.00011	0.00007	0.00006	0.00004
82	C3	PX'	-0.00000	-0.00002	-0.00001	-0.00001	-0.00002
83	C3	PY'	0.00000	0.00006	0.00003	0.00003	0.00006
84	C3	PZ'	-0.00003	-0.00000	-0.00001	-0.00000	-0.00001
85	C3	DXX	0.00000	-0.00000	-0.00000	0.00000	0.00000
86	C3	DYY	0.00000	-0.00000	-0.00000	-0.00000	0.00000
87	C3	DZZ	-0.00001	0.00000	0.00000	0.00000	-0.00000
88	C3	DXY	-0.00000	-0.00000	0.00000	0.00000	-0.00000
89	C3	DXZ	-0.00000	-0.00000	0.00000	0.00000	-0.00000
90	C3	DYZ	0.00000	-0.00000	-0.00000	0.00000	0.00000
91	H2	S	0.00001	-0.00000	-0.00000	-0.00000	0.00000
92	H2	S'	0.00005	-0.00002	-0.00002	-0.00003	0.00001
93	H5	S	0.00001	0.00000	0.00000	0.00000	0.00000
94	H5	S'	-0.00006	0.00002	0.00001	0.00001	-0.00001
95	H3	S	0.00000	0.00000	0.00000	0.00000	0.00000
96	H3	S'	0.00001	0.00001	0.00000	0.00001	0.00002
97	C7	S1	0.00000	-0.00000	-0.00000	-0.00000	0.00000
98	C7	S	0.00001	0.00000	0.00000	0.00000	0.00000
99	C7	PX	-0.00000	-0.00000	0.00000	0.00000	0.00000
100	C7	PY	-0.00000	-0.00000	0.00000	-0.00000	-0.00000
101	C7	PZ	-0.00000	0.00000	-0.00000	-0.00000	-0.00000
102	C7	S'	-0.00014	-0.00003	-0.00001	-0.00001	-0.00011
103	C7	PX'	0.00001	0.00003	0.00001	-0.00000	-0.00000
104	C7	PY'	-0.00006	-0.00002	-0.00001	-0.00001	-0.00003
105	C7	PZ'	0.00028	-0.00002	0.00001	0.00002	0.00015
106	C7	DXX	0.00000	-0.00000	-0.00000	0.00000	-0.00000
107	C7	DYY	0.00000	-0.00000	-0.00000	-0.00000	0.00000
108	C7	DZZ	-0.00000	-0.00000	0.00000	-0.00000	-0.00000
109	C7	DXY	0.00000	0.00000	0.00000	0.00000	0.00000
110	C7	DXZ	-0.00000	0.00000	0.00000	-0.00000	-0.00000
111	C7	DYZ	0.00000	-0.00000	0.00000	0.00000	0.00000
112	C8	S1	0.00000	0.00000	0.00000	0.00000	0.00000
113	C8	S	0.00000	0.00000	0.00000	0.00000	0.00000
114	C8	PX	0.00000	0.00000	0.00000	0.00000	0.00000
115	C8	PY	-0.00000	-0.00000	-0.00000	-0.00000	0.00000
116	C8	PZ	-0.00000	0.00000	0.00000	-0.00000	-0.00000
117	C8	S'	-0.00003	-0.00001	-0.00000	-0.00000	-0.00001
118	C8	PX'	0.00001	0.00000	0.00000	0.00000	0.00000
119	C8	PY'	-0.00001	-0.00000	-0.00000	-0.00000	-0.00001

120	C8	PZ'	0.00001	0.00000	0.00000	0.00000	0.00001
121	C8	DXX	0.00000	-0.00000	-0.00000	-0.00000	0.00000
122	C8	DYY	0.00000	-0.00000	0.00000	0.00000	0.00000
123	C8	DZZ	0.00000	0.00000	0.00000	0.00000	0.00000
124	C8	DXY	0.00000	0.00000	0.00000	0.00000	0.00000
125	C8	DXZ	0.00000	0.00000	0.00000	0.00000	0.00000
126	C8	DYZ	-0.00000	-0.00000	-0.00000	0.00000	0.00000
127	C9	S1	-0.00000	-0.00000	-0.00000	-0.00000	-0.00000
128	C9	S	0.00000	-0.00000	-0.00000	0.00000	0.00000
129	C9	PX	0.00000	-0.00000	0.00000	0.00000	0.00000
130	C9	PY	-0.00000	0.00000	-0.00000	-0.00000	-0.00000
131	C9	PZ	0.00000	0.00000	0.00000	0.00000	0.00000
132	C9	S'	0.00000	0.00010	0.00001	-0.00001	-0.00003
133	C9	PX'	-0.00019	-0.00003	-0.00001	-0.00001	-0.00008
134	C9	PY'	0.00010	0.00003	0.00001	0.00001	0.00004
135	C9	PZ'	0.00003	-0.00003	-0.00000	0.00001	0.00001
136	C9	DXX	0.00000	0.00000	0.00000	-0.00000	-0.00000
137	C9	DYY	0.00000	-0.00000	0.00000	0.00000	0.00000
138	C9	DZZ	-0.00000	0.00000	-0.00000	-0.00000	-0.00000
139	C9	DXY	-0.00000	-0.00000	-0.00000	0.00000	-0.00000
140	C9	DXZ	0.00000	-0.00000	0.00000	0.00000	0.00000
141	C9	DYZ	0.00000	-0.00000	-0.00000	0.00000	0.00000
142	C10	S1	0.00000	-0.00000	-0.00000	0.00000	0.00000
143	C10	S	0.00000	-0.00000	0.00000	0.00000	0.00000
144	C10	PX	0.00000	0.00000	0.00000	-0.00000	-0.00000
145	C10	PY	0.00000	-0.00000	-0.00000	0.00000	0.00000
146	C10	PZ	-0.00000	0.00000	0.00000	-0.00000	-0.00000
147	C10	S'	-0.00002	-0.00002	-0.00001	0.00000	-0.00000
148	C10	PX'	0.00000	0.00000	0.00000	-0.00000	-0.00000
149	C10	PY'	0.00000	-0.00000	-0.00000	0.00000	0.00000
150	C10	PZ'	-0.00004	0.00001	-0.00000	-0.00001	-0.00002
151	C10	DXX	0.00000	-0.00000	-0.00000	0.00000	0.00000
152	C10	DYY	0.00000	-0.00000	0.00000	0.00000	0.00000
153	C10	DZZ	-0.00000	-0.00000	0.00000	0.00000	-0.00000
154	C10	DXY	0.00000	-0.00000	-0.00000	-0.00000	0.00000
155	C10	DXZ	0.00000	-0.00000	0.00000	0.00000	0.00000
156	C10	DYZ	-0.00000	0.00000	0.00000	-0.00000	-0.00000
157	C11	S1	0.00000	-0.00000	-0.00000	0.00000	0.00000
158	C11	S	0.00000	-0.00000	-0.00000	0.00000	0.00000

159	C11	PX	-0.00000	0.00000	-0.00000	-0.00000	-0.00000
160	C11	PY	0.00000	-0.00000	0.00000	0.00000	0.00000
161	C11	PZ	0.00000	-0.00000	-0.00000	-0.00000	0.00000
162	C11	S'	0.00003	0.00000	0.00000	0.00000	0.00001
163	C11	PX'	-0.00000	-0.00000	-0.00000	-0.00000	0.00000
164	C11	PY'	0.00001	0.00000			

6.2 Calculation with ORCA (B3LYP RIJCOSX D3BJ def2-TZVP def2/J TIGHTSCF Opt NumFreq Grid5 FinalGrid6) Program Version 4.1.2

Calculation: B3LYP RIJCOSX D3BJ def2-TZVP def2/J TIGHTSCF Opt NumFreq Grid5 FinalGrid6

Structure 1a

FINAL SINGLE POINT ENERGY: -1917.896782035197 E_h

Structure 1b

FINAL SINGLE POINT ENERGY: -1917.886403451657 E_h

Structure LB_1 is (0.010378584 E_h, 27.24 KJ/mol, 6.51 Kcal/mol) lower in Energy.

E_h = 1 hartree

Geometry Scan at a lower level of theory (RI BP86 def2-SVP def2/J D3BJ TIGHTSCF Opt Grid3 FinalGrid5)

Dihedral angle from 0-360° between rings A and E.

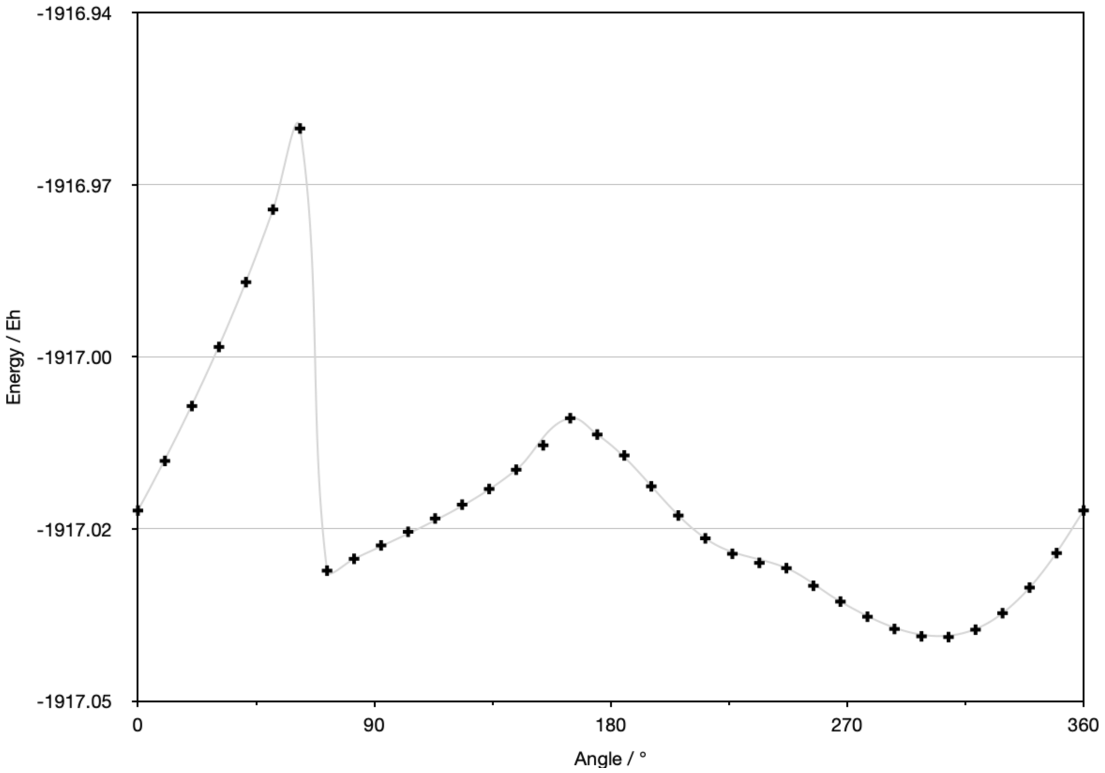

Energy difference between **1b** and transition state: 0.02436632 E_h, 63.97 KJ/mol, 15.29 Kcal/mol

Structur 1a

IR SPECTRUM

Mode	freq (cm**-1)	T**2	TX	TY	TZ
6:	6.32	0.176054	(0.114211	0.381710	0.131556)
7:	19.11	0.058404	(0.219568	0.078278	-0.063771)
8:	26.71	0.049697	(0.164848	0.025899	-0.147821)
9:	30.84	0.226639	(-0.220701	-0.370272	0.202061)
10:	42.30	0.052033	(0.017963	0.183359	-0.134500)
11:	46.12	0.217800	(-0.102052	0.241743	-0.385935)
12:	53.41	1.427992	(0.574142	1.017120	0.252626)
13:	58.70	0.805456	(0.610810	0.622194	0.212704)
14:	62.23	0.375615	(-0.467978	-0.069672	-0.389560)
15:	64.31	0.093881	(-0.189378	0.116676	0.210721)
16:	81.69	0.210944	(0.281209	0.257603	0.255941)
17:	89.33	0.097453	(-0.299947	-0.083897	-0.021116)
18:	91.20	0.191166	(-0.034800	-0.408499	-0.151933)
19:	108.59	0.380521	(-0.485734	-0.365456	-0.105001)
20:	124.24	0.391972	(0.382515	0.410405	0.277888)
21:	130.44	0.120419	(-0.323644	-0.084840	0.092061)
22:	136.69	0.571350	(-0.249949	0.436668	-0.564089)
23:	143.77	0.043953	(-0.024714	-0.060999	-0.199052)
24:	153.82	0.171722	(0.238100	-0.288252	0.178721)
25:	155.95	0.075797	(0.122404	0.183107	-0.165184)
26:	163.62	0.113433	(0.304269	-0.004642	0.144333)
27:	179.91	0.193617	(-0.044675	-0.351938	-0.260309)
28:	194.19	0.197818	(-0.343593	-0.280420	0.033557)
29:	209.81	0.018437	(0.052573	-0.119703	-0.036659)
30:	218.91	0.521015	(0.232456	-0.023993	-0.682937)
31:	227.32	1.124284	(0.428439	-0.868415	0.431949)
32:	237.43	1.185651	(-0.029110	0.928092	-0.568725)
33:	259.87	0.139277	(-0.323565	-0.164700	0.086350)
34:	268.22	0.015461	(0.064576	-0.077620	0.072569)
35:	272.16	0.265902	(0.047016	0.174145	0.483079)
36:	282.69	0.709732	(0.578621	0.489552	0.367788)
37:	307.95	0.161992	(0.164229	-0.201223	-0.307457)
38:	312.35	0.423703	(0.325840	0.100985	-0.554376)
39:	325.48	0.616213	(-0.341952	0.057483	0.704256)
40:	340.03	1.038271	(0.235062	-0.340201	-0.931279)
41:	347.51	0.102916	(-0.170604	0.238724	0.129695)
42:	359.99	0.252768	(0.042454	0.382498	-0.323513)
43:	377.94	1.305497	(-0.410905	-0.685981	-0.816140)

44:	382.35	2.301737	(-0.815977	0.045160	-1.278232)
45:	392.31	1.295307	(0.777383	0.684948	-0.470988)
46:	397.46	1.392359	(-0.505247	0.570951	-0.900610)
47:	402.56	2.199459	(-0.800611	0.312597	1.208621)
48:	418.51	1.314258	(0.033657	-0.005541	1.145903)
49:	433.38	0.122085	(0.072782	0.293489	-0.175077)
50:	440.71	1.934777	(1.297977	0.126901	0.483663)
51:	445.21	0.146017	(0.177485	0.328534	0.081128)
52:	456.98	2.797106	(-0.857248	0.385466	1.383346)
53:	471.68	2.934562	(-0.609894	-1.427387	0.724677)
54:	473.96	1.369692	(-0.214043	0.135922	1.142542)
55:	484.11	3.294547	(-0.295222	-1.757935	0.342135)
56:	490.35	7.289232	(-1.036332	-0.384129	2.463268)
57:	499.24	5.966927	(0.767105	1.503701	1.765605)
58:	506.58	7.449741	(0.258828	-0.559538	-2.658884)
59:	511.01	5.463215	(-0.101275	-1.913867	-1.337935)
60:	513.29	2.612766	(-0.396004	0.402573	-1.514557)
61:	523.61	32.905782	(2.024415	0.673169	-5.324882)
62:	528.98	7.629330	(0.746564	-2.659262	-0.017327)
63:	532.81	21.719774	(-2.856518	0.883504	3.574843)
64:	540.72	11.011948	(-1.905225	1.673266	2.140618)
65:	543.92	5.878474	(0.305730	0.602632	-2.328484)
66:	556.25	41.451211	(-1.943995	2.822452	-5.450308)
67:	558.82	5.764140	(1.007020	-0.649412	-2.080460)
68:	563.56	4.798412	(-0.867748	-0.459895	1.958040)
69:	564.70	1.626079	(1.086127	-0.260362	-0.615321)
70:	573.78	4.807645	(0.512125	0.284248	-2.112954)
71:	583.43	4.158540	(0.758879	-0.316959	1.866060)
72:	592.56	2.343474	(1.164010	0.037056	-0.993570)
73:	595.31	4.445840	(-1.076375	1.573623	-0.900538)
74:	603.06	2.674247	(-0.067814	-0.408768	-1.581947)
75:	605.73	4.290201	(1.320039	-1.245112	0.998696)
76:	629.66	0.791199	(0.606238	-0.245063	-0.603008)
77:	636.56	4.162392	(-1.183712	-1.194619	-1.155034)
78:	640.73	2.876973	(0.754513	0.353933	-1.477300)
79:	648.11	2.742749	(0.725497	-0.959913	1.137967)
80:	652.59	11.000633	(0.102003	0.128809	3.312648)
81:	666.61	6.905455	(0.222524	-1.350998	2.242932)
82:	680.39	1.423462	(-0.352559	0.776509	-0.834385)
83:	689.51	1.269563	(-0.244062	-1.085487	-0.178090)
84:	702.25	18.385548	(-2.247952	-3.024441	-2.045732)
85:	717.59	5.736740	(-1.040310	-2.071816	0.601727)
86:	721.35	4.900009	(-0.841063	-1.426895	-1.468534)
87:	722.77	9.979900	(-0.276056	2.521560	1.882931)
88:	726.35	4.261728	(0.059675	-1.670357	1.211641)

89:	762.22	1.889338	(-0.054337	0.898225	-1.039027)
90:	767.19	35.020438	(1.945836	5.541106	-0.728217)
91:	768.02	2.648610	(0.582863	1.405862	0.576569)
92:	773.15	23.130213	(-0.528618	-4.738865	0.627640)
93:	776.45	58.607145	(-5.670716	-3.264739	-3.973866)
94:	781.05	8.719683	(1.552609	2.110339	-1.362189)
95:	792.77	3.202981	(0.997560	-0.998271	-1.100595)
96:	807.02	1.780765	(1.080880	-0.193594	0.758278)
97:	811.69	26.903399	(-3.396686	-3.497450	-1.770246)
98:	813.38	24.263438	(-2.208268	-4.240882	-1.184021)
99:	846.73	0.103232	(0.036455	0.103257	0.302062)
100:	849.65	26.820593	(-4.204597	-3.008762	0.298840)
101:	855.48	13.491788	(-2.860698	-1.402931	1.827561)
102:	892.15	0.182043	(-0.044407	-0.416263	-0.082439)
103:	897.86	0.792498	(-0.269573	0.058619	0.846399)
104:	911.29	5.789461	(0.162047	0.885382	2.231435)
105:	921.50	5.409338	(0.076092	-2.280928	0.448237)
106:	922.05	0.591612	(-0.215294	-0.729167	0.116516)
107:	926.66	5.566627	(2.228400	-0.292152	-0.717988)
108:	928.74	8.089212	(1.945891	0.784974	1.920035)
109:	932.56	5.368583	(0.699939	1.677056	-1.437411)
110:	934.71	11.062171	(-1.870090	-2.710373	-0.467777)
111:	941.50	1.033367	(-0.243559	-0.274721	-0.947932)
112:	945.97	1.669207	(0.711346	-0.272761	1.043454)
113:	962.76	0.528912	(0.174268	0.387150	0.590473)
114:	967.38	2.268978	(0.587792	1.222486	0.654986)
115:	971.79	0.314813	(0.199813	0.451466	0.266582)
116:	976.25	1.194587	(0.004009	-0.761102	0.784407)
117:	980.16	0.317240	(-0.013099	0.535129	0.175229)
118:	980.71	0.073301	(-0.174284	-0.190902	0.080514)
119:	981.66	0.061268	(-0.035099	-0.079631	0.231722)
120:	982.33	0.996247	(-0.336899	-0.927794	-0.148134)
121:	1017.31	0.896041	(-0.418442	-0.109282	0.842024)
122:	1022.01	0.483745	(-0.454296	-0.354419	-0.389547)
123:	1029.94	0.644203	(0.195089	-0.565660	-0.534950)
124:	1041.77	0.380856	(0.547135	-0.085783	-0.272288)
125:	1049.00	0.445152	(-0.243839	0.384753	-0.487503)
126:	1063.67	4.808509	(-0.502464	0.170312	2.127683)
127:	1068.67	5.005621	(-1.581476	-0.093265	-1.579828)
128:	1082.19	1.023535	(-0.716427	0.670463	0.246468)
129:	1105.93	8.967370	(1.143755	0.332585	-2.747468)
130:	1114.58	1.756604	(1.179338	0.257320	-0.547313)
131:	1122.65	3.707551	(-1.373160	0.249922	-1.326470)
132:	1124.69	4.594657	(0.563961	-1.291722	1.614949)
133:	1135.53	2.192772	(0.370478	-0.676046	-1.264310)

134:	1155.56	2.487588	(-0.148797	1.490320	0.494363)
135:	1170.40	0.254182	(0.155012	-0.464403	-0.120345)
136:	1173.59	1.785299	(0.771944	-0.634903	0.886735)
137:	1180.10	0.344371	(-0.134755	-0.555799	0.131527)
138:	1191.11	0.295528	(-0.101028	0.430919	-0.315641)
139:	1192.52	0.745033	(-0.046679	0.227675	-0.831275)
140:	1197.38	0.185644	(-0.059668	-0.421563	0.066095)
141:	1197.93	0.108289	(-0.087566	0.114817	0.295700)
142:	1224.48	1.433410	(0.457275	-0.550578	-0.959778)
143:	1236.54	6.622602	(-0.127792	-0.378433	2.542255)
144:	1257.85	1.917386	(0.590639	-1.028659	-0.714417)
145:	1266.23	0.717769	(-0.562646	0.376570	0.509307)
146:	1277.54	2.382852	(0.397435	-0.391486	-1.439318)
147:	1281.67	1.197690	(-0.421685	0.910263	-0.437371)
148:	1290.21	0.677851	(-0.338948	0.598949	-0.451913)
149:	1292.38	0.163727	(-0.215995	-0.323719	0.110812)
150:	1301.59	1.659810	(-0.693666	1.057010	0.247721)
151:	1304.34	3.192533	(0.467342	0.628459	-1.605978)
152:	1305.02	0.476139	(-0.307315	0.616250	-0.043959)
153:	1311.02	1.076278	(-0.205253	-0.049463	1.015728)
154:	1314.64	2.076897	(0.591241	-0.177020	-1.302304)
155:	1318.80	1.164976	(-0.454751	-0.015454	0.978744)
156:	1343.88	0.005528	(0.069740	-0.008211	0.024434)
157:	1346.78	0.442935	(0.048573	0.560171	-0.356067)
158:	1347.42	3.916993	(0.559160	-0.747980	1.744953)
159:	1352.19	2.323190	(0.636436	-0.366182	-1.335683)
160:	1355.85	0.986785	(-0.217158	-0.858107	0.450865)
161:	1382.13	1.686118	(0.235004	-0.056716	-1.275803)
162:	1397.66	1.513391	(0.717173	-0.997893	0.057131)
163:	1417.80	1.040190	(-0.016549	0.394382	0.940414)
164:	1430.50	6.156464	(1.282755	-1.350432	-1.639310)
165:	1435.44	10.682277	(-1.403262	2.445162	1.653577)
166:	1439.87	7.545034	(-2.579242	-0.407778	-0.852210)
167:	1478.72	5.824548	(0.219957	-0.935650	2.213759)
168:	1485.89	5.186976	(0.358398	2.246417	0.110175)
169:	1499.78	21.448261	(2.874155	0.403501	-3.608972)
170:	1508.50	17.617085	(-0.306415	-0.652535	-4.134899)
171:	1516.50	100.573555	(-3.102647	5.175025	-8.010384)
172:	1518.93	11.193605	(2.932593	-0.542104	-1.516453)
173:	1524.45	23.191397	(-3.629196	2.894496	-1.281493)
174:	1532.25	15.088186	(-3.683656	1.097444	0.560783)
175:	1566.33	27.622127	(2.863184	-2.372795	-3.714048)
176:	1575.31	3.828022	(0.454405	1.569294	1.076501)
177:	1589.35	0.385137	(0.462678	-0.412116	0.035014)
178:	1602.65	2.243651	(0.281096	0.680320	-1.304531)

179:	1610.94	25.695556	(-2.144691	-0.543605	4.560740)
180:	1613.25	6.556739	(-2.504444	-0.374373	0.379928)
181:	1618.36	3.074227	(0.539704	-0.494694	-1.593181)
182:	1627.43	19.831592	(-3.197813	2.715934	-1.493079)
183:	1632.89	5.219958	(-0.500473	-0.316990	-2.206582)
184:	1636.47	4.931392	(-0.738860	0.484835	2.037256)
185:	1637.22	9.929259	(-0.512541	-0.717478	3.025192)
186:	1638.09	7.580976	(2.137478	-1.466036	0.928925)
187:	2224.21	2.381581	(0.226541	0.775048	-1.315128)
188:	2230.96	4.529630	(1.046068	-0.635239	-1.741219)
189:	2280.85	27.177197	(2.917035	-4.277558	0.608767)
190:	2288.92	3.565790	(-0.638317	1.272336	-1.240767)
191:	2295.45	3.311352	(-0.486662	-0.762068	1.579165)
192:	2303.97	7.989649	(-1.572525	0.963341	2.142146)
193:	2311.86	1.614336	(0.832169	0.423997	-0.861428)
194:	3172.83	2.866022	(0.921843	0.018852	1.419814)
195:	3173.99	0.631466	(0.261128	-0.553163	0.507236)
196:	3175.20	6.091287	(-0.632243	-0.879964	-2.217480)
197:	3175.93	8.773313	(-1.206131	-0.394957	2.676298)
198:	3177.96	4.066273	(-2.001582	-0.190981	-0.153197)
199:	3183.61	5.772656	(-1.576034	0.945204	-1.547696)
200:	3185.73	5.773288	(1.510735	-0.578315	-1.776659)
201:	3189.44	1.605847	(-1.162869	0.488817	0.120996)
202:	3190.28	7.078187	(0.921112	-0.520025	-2.441171)
203:	3191.60	4.422255	(1.321906	0.390706	-1.588134)
204:	3193.75	1.047385	(-0.313455	-0.528037	0.818723)
205:	3194.43	11.145701	(-1.883767	2.042419	1.850851)
206:	3194.97	5.928432	(1.430751	-1.942513	0.328677)
207:	3196.36	9.897976	(0.801939	-2.803049	1.182280)
208:	3201.86	15.885415	(-2.791803	0.268369	2.831824)
209:	3202.20	22.407684	(2.313304	-1.205871	3.949960)
210:	3202.26	16.147424	(2.307344	-1.167185	-3.075917)
211:	3202.76	1.905687	(-0.904111	-0.946971	-0.437626)
212:	3203.09	1.931632	(1.386455	-0.076892	0.058843)
213:	3206.03	8.306420	(-1.553395	2.324844	-0.698917)
214:	3210.22	0.417421	(-0.009241	0.316301	0.563284)
215:	3211.65	0.743779	(0.296077	0.800614	-0.123025)

The first frequency considered to be a vibration is 6

The total number of vibrations considered is 210

Structure 1b

IR SPECTRUM

Mode	freq (cm**-1)	T**2	TX	TY	TZ
6:	13.93	0.063834	(0.179109	-0.032353	-0.175236)
7:	21.10	0.119766	(0.319235	-0.047570	-0.124871)
8:	30.52	0.088724	(0.233457	0.039367	-0.180753)
9:	42.89	0.490372	(-0.482113	-0.474172	-0.181932)
10:	47.96	0.458171	(-0.503578	-0.294015	-0.343708)
11:	49.12	0.322086	(0.134990	0.485639	0.260805)
12:	54.11	0.318712	(-0.421228	-0.374499	0.032090)
13:	55.19	0.044719	(-0.060178	-0.072657	0.189258)
14:	62.65	0.827033	(-0.844380	0.336888	0.023703)
15:	79.31	0.408689	(-0.592440	0.216913	-0.103210)
16:	87.63	0.282251	(-0.325653	0.418732	-0.029404)
17:	96.94	1.230159	(1.065502	0.060225	-0.302054)
18:	102.82	0.386798	(0.238029	0.385785	0.425805)
19:	113.61	0.159129	(0.348662	0.129382	-0.144304)
20:	120.73	0.229976	(-0.409911	-0.015837	0.248391)
21:	132.46	0.194665	(0.166960	0.227532	-0.339143)
22:	135.66	0.203503	(0.342806	0.046254	-0.289565)
23:	150.54	0.375231	(0.105010	-0.197158	-0.570379)
24:	155.54	0.829529	(0.272217	0.866778	-0.064208)
25:	163.62	0.199860	(0.311834	-0.177011	0.266994)
26:	170.97	0.281323	(0.352623	0.396148	0.006850)
27:	179.43	0.330443	(-0.352826	-0.180961	0.416185)
28:	197.09	0.021673	(0.077443	0.125196	-0.001060)
29:	207.51	0.031342	(-0.083660	0.005404	0.155930)
30:	216.18	0.240130	(0.231747	0.098234	0.420444)
31:	230.26	1.125236	(0.249361	-0.980913	0.317594)
32:	247.26	0.453377	(-0.081972	-0.642973	-0.182328)
33:	264.61	0.106840	(-0.090795	0.305171	-0.073937)
34:	269.27	0.075516	(-0.206464	-0.039723	0.176949)
35:	278.28	0.378906	(-0.555738	-0.262304	-0.035463)
36:	283.20	0.312418	(0.516247	-0.040716	-0.210354)
37:	300.11	0.294796	(0.350420	-0.387966	-0.146576)
38:	315.46	0.318295	(0.269196	0.097012	0.486228)
39:	322.40	0.023869	(0.093186	-0.079500	-0.094157)
40:	330.14	1.098730	(0.171059	-0.001282	-1.034151)
41:	346.09	0.239784	(-0.172679	-0.147487	-0.433836)
42:	374.84	0.510194	(-0.036925	0.540098	-0.465966)
43:	385.47	2.776710	(0.352919	-0.366547	1.586758)

44:	388.34	3.305804	(-1.273010	-0.288788	1.265643)
45:	398.74	1.844280	(0.864883	0.387717	0.972591)
46:	402.28	2.235938	(-0.474069	1.281107	0.608244)
47:	415.49	0.471705	(0.276394	0.628587	-0.013776)
48:	420.55	1.748515	(-0.237199	0.429778	1.227820)
49:	435.31	0.264060	(0.229798	0.376028	0.264303)
50:	441.52	2.516866	(0.601989	-0.308055	-1.435123)
51:	445.19	1.631447	(-0.391131	-0.363818	-1.160216)
52:	457.37	0.412231	(-0.308690	0.559539	-0.062113)
53:	470.12	0.681281	(0.783578	0.003257	0.259377)
54:	471.85	2.795411	(1.121196	0.146442	1.231619)
55:	483.04	2.518126	(-0.389956	0.218152	-1.522652)
56:	491.09	9.509157	(-0.903846	1.109884	2.731369)
57:	495.56	3.658087	(-0.198178	1.901944	-0.037710)
58:	503.28	9.423583	(-1.471758	-1.175883	2.423801)
59:	507.23	0.241473	(0.222534	0.425327	-0.105113)
60:	514.55	7.593955	(-1.176013	-2.094403	-1.350713)
61:	525.72	60.160372	(2.852922	-0.008505	-7.212568)
62:	531.95	20.781615	(-0.628501	-2.280308	-3.897024)
63:	533.32	5.808190	(1.350552	-1.918666	0.550384)
64:	543.91	2.854255	(-0.088036	0.500820	-1.611113)
65:	548.97	7.776551	(0.822619	1.204211	2.376915)
66:	553.42	5.876886	(-0.986653	1.258541	1.821943)
67:	555.59	42.900161	(1.050052	-2.477844	5.971419)
68:	565.18	4.678052	(-0.895915	-0.603085	1.873947)
69:	566.68	0.694908	(-0.460012	0.350327	0.600473)
70:	575.28	4.375179	(1.360896	0.059033	-1.587342)
71:	588.13	7.020603	(-0.267464	0.647638	-2.555314)
72:	592.78	6.129039	(0.544869	-1.231669	-2.077294)
73:	599.17	2.552427	(0.345642	-1.469470	0.523085)
74:	603.17	4.379453	(1.612683	-1.249652	-0.465915)
75:	605.47	3.511213	(-0.846893	1.411875	-0.894760)
76:	626.24	3.432816	(1.385115	-0.852366	-0.887549)
77:	639.26	6.689317	(1.425135	1.168827	-1.814428)
78:	640.90	4.131637	(0.942842	0.143764	-1.794998)
79:	648.64	5.096737	(-0.657434	0.628304	-2.066338)
80:	653.24	3.921506	(1.088300	-1.023629	1.299728)
81:	661.40	1.867204	(-0.857117	-0.148938	1.053742)
82:	680.64	1.481791	(0.293691	-0.550917	1.045001)
83:	692.44	2.319310	(-0.859857	0.204092	1.240283)
84:	696.26	20.552744	(-3.107360	-2.311409	-2.356787)
85:	717.98	0.043925	(-0.159161	-0.093088	-0.099637)
86:	720.62	17.749389	(3.254720	-2.670083	-0.163855)
87:	724.21	1.834679	(-0.014257	-1.185264	0.655457)
88:	725.96	4.779334	(-0.392578	-0.264179	2.134345)

89:	765.92	2.244422	(-0.881202	0.365915	1.154994)
90:	766.19	13.199137	(1.568660	-0.811233	-3.174956)
91:	767.16	52.215752	(7.117578	0.503198	-1.141326)
92:	769.72	18.439577	(2.789436	1.774444	2.740433)
93:	773.04	33.672830	(2.673874	4.758754	1.969134)
94:	780.94	44.130959	(2.998529	4.652206	-3.673794)
95:	790.31	3.683127	(0.049621	-1.472089	-1.230292)
96:	805.01	27.918977	(4.167267	2.214361	2.376861)
97:	809.16	3.767443	(-1.332552	0.065379	-1.409778)
98:	822.28	35.938136	(5.586884	-2.076031	0.644169)
99:	843.52	0.150578	(-0.157460	0.192529	0.297854)
100:	849.12	27.443246	(-4.438042	-2.781241	0.108288)
101:	853.87	5.832459	(2.167097	0.795924	0.708980)
102:	891.35	0.570938	(-0.047896	0.194894	0.728464)
103:	893.76	0.482032	(-0.097128	0.055884	0.685183)
104:	908.74	6.808651	(0.731260	-0.556719	-2.442125)
105:	916.85	0.105243	(0.240544	-0.014411	-0.217195)
106:	921.42	6.436233	(-1.315536	2.153550	-0.260425)
107:	921.88	4.109231	(-0.209772	1.600671	-1.226001)
108:	927.26	11.143774	(2.972073	-0.302307	1.489687)
109:	929.47	9.403207	(-2.162115	-2.174107	0.041567)
110:	939.42	3.320175	(-1.390702	-0.414163	1.102086)
111:	941.77	6.344723	(2.176081	0.026035	1.268353)
112:	948.57	0.299275	(0.384488	0.134240	-0.365272)
113:	961.48	2.650730	(1.402690	0.799995	0.207839)
114:	967.11	0.574823	(-0.067409	-0.434424	-0.617701)
115:	968.92	0.219979	(-0.259568	-0.381992	-0.081762)
116:	969.99	0.290144	(0.453944	0.081542	-0.278261)
117:	975.73	0.251573	(0.164404	0.024922	-0.473205)
118:	977.78	0.004928	(-0.048743	-0.043441	0.025790)
119:	978.98	0.620476	(-0.490874	0.018137	-0.615784)
120:	1002.22	0.497802	(-0.547057	0.440516	0.066901)
121:	1016.45	0.573054	(-0.462144	0.061647	0.596386)
122:	1020.67	0.120834	(0.086986	-0.099973	-0.321360)
123:	1029.78	0.870097	(0.425462	0.306547	0.771432)
124:	1040.78	0.171614	(0.265587	-0.286904	0.136980)
125:	1047.29	0.839039	(0.350698	-0.082255	0.842190)
126:	1061.60	6.292172	(0.055553	-0.809352	-2.373612)
127:	1067.05	1.542762	(-0.197109	-0.384577	1.164479)
128:	1087.50	2.516028	(0.727608	-1.055462	0.934138)
129:	1103.20	9.153007	(-1.384069	-0.369427	2.664748)
130:	1117.40	2.105029	(-0.295700	0.144086	-1.413092)
131:	1123.90	5.286594	(-0.054558	1.508927	-1.734000)
132:	1127.76	0.963879	(-0.735453	-0.649983	0.022599)
133:	1136.49	5.284916	(-1.101558	0.533697	1.945933)

134:	1155.37	2.160230	(0.203606	-1.198170	-0.826536)
135:	1168.86	1.055155	(0.557544	-0.700024	-0.504248)
136:	1171.66	1.777422	(0.464269	-1.030317	0.707335)
137:	1178.29	0.704649	(-0.144533	0.675848	-0.476434)
138:	1187.29	0.080823	(-0.082762	0.189502	-0.195096)
139:	1192.05	0.064534	(-0.082264	0.046559	0.235794)
140:	1197.36	0.101769	(-0.052230	-0.224580	0.220466)
141:	1207.42	0.534112	(-0.057772	0.074943	0.724678)
142:	1220.48	2.260653	(0.199441	0.871146	1.209124)
143:	1235.02	6.168467	(0.611984	0.137537	-2.403128)
144:	1251.92	2.033582	(1.168468	-0.797864	-0.177983)
145:	1258.40	0.831147	(-0.119851	-0.718173	-0.548644)
146:	1274.71	1.214742	(0.076964	-0.202392	-1.080674)
147:	1279.68	0.927522	(-0.372081	0.887070	0.046748)
148:	1286.76	1.720941	(-0.335857	1.265314	-0.084391)
149:	1292.49	0.847491	(0.012908	0.607323	-0.691725)
150:	1296.56	0.578272	(0.414103	-0.581331	-0.262382)
151:	1301.05	0.888151	(-0.100590	-0.476911	-0.806591)
152:	1304.48	1.831153	(-0.093556	0.012671	1.349903)
153:	1310.08	1.706050	(0.876848	-0.450018	0.857131)
154:	1310.63	0.791474	(0.760720	-0.330058	-0.322245)
155:	1316.92	2.327655	(-0.671942	-0.075021	1.367670)
156:	1341.56	0.386071	(0.604881	0.014841	0.141316)
157:	1348.30	2.613781	(0.437197	-0.723725	1.377992)
158:	1349.83	0.354371	(0.539191	0.070119	-0.242336)
159:	1353.34	1.617598	(0.464742	-0.064584	-1.182134)
160:	1356.12	0.513248	(0.151643	-0.380530	0.587750)
161:	1379.93	3.997858	(-0.802342	0.095928	1.828908)
162:	1394.92	2.641254	(-0.591064	1.430611	0.495226)
163:	1416.79	0.187574	(0.161677	0.371371	0.153357)
164:	1428.70	3.060623	(0.793114	-1.135117	-1.069160)
165:	1434.36	13.162314	(-1.940729	2.614120	1.600706)
166:	1446.84	7.726136	(1.601079	2.110426	-0.841894)
167:	1475.97	5.390754	(-0.383333	0.904022	-2.103938)
168:	1485.43	2.886559	(-0.949845	1.349177	0.405061)
169:	1496.13	14.969482	(0.400838	1.178209	3.663418)
170:	1506.93	22.959678	(0.910824	0.363230	4.690218)
171:	1515.11	64.523354	(-2.328106	2.959973	-7.095198)
172:	1518.89	1.456704	(0.132860	0.888015	-0.806525)
173:	1521.85	58.621624	(2.663622	-5.431862	4.692719)
174:	1532.28	16.663749	(-2.971422	2.796182	-0.125570)
175:	1560.82	22.604394	(2.125085	-3.233780	-2.762440)
176:	1572.27	1.824815	(1.111810	-0.421322	-0.641233)
177:	1588.08	0.730923	(-0.002820	-0.731430	-0.442634)
178:	1601.79	1.340718	(0.098400	-0.406899	1.079569)

179:	1608.69	5.855416	(-1.662856	-1.605580	0.715849)
180:	1610.91	27.798691	(-2.877390	0.171781	4.414726)
181:	1617.33	3.281361	(0.418310	0.824787	1.557595)
182:	1626.62	14.273043	(1.697681	-2.932539	1.670670)
183:	1631.43	15.134038	(2.140351	-1.558401	2.850319)
184:	1632.54	5.813218	(1.986954	-0.744393	-1.145038)
185:	1635.89	2.417674	(-0.382152	0.418800	1.447840)
186:	1638.08	24.283631	(-1.149496	0.132409	-4.790069)
187:	2221.72	3.044149	(0.682350	0.609217	-1.485733)
188:	2225.98	4.220141	(0.491542	-0.662683	-1.881324)
189:	2264.49	19.236813	(1.959362	-3.912310	0.302570)
190:	2281.03	12.282875	(-0.940494	2.868262	-1.780848)
191:	2290.14	3.574414	(0.117520	0.586250	1.793576)
192:	2298.04	7.943637	(0.878140	1.961829	1.823111)
193:	2303.28	1.235885	(-1.047527	-0.156591	0.337714)
194:	3173.47	0.793852	(-0.238613	-0.318430	0.797194)
195:	3176.32	0.474800	(0.324835	-0.073740	-0.603196)
196:	3176.63	6.251212	(-0.514534	0.417070	2.410917)
197:	3181.18	9.461443	(-1.472086	0.040947	2.700506)
198:	3181.46	4.100119	(-1.427970	-1.426111	0.165009)
199:	3185.58	12.434249	(2.218333	-2.419198	1.288691)
200:	3186.92	5.195041	(-0.182524	-0.870055	-2.098745)
201:	3189.11	3.250859	(0.543408	1.670544	0.406019)
202:	3191.73	5.185866	(0.609389	-0.650310	-2.095616)
203:	3195.34	11.310178	(-1.244403	-1.373086	2.806470)
204:	3196.47	11.794456	(-0.525236	2.994583	-1.597203)
205:	3197.84	2.778805	(-0.225046	1.516906	-0.653571)
206:	3199.37	2.668624	(-0.651077	-0.507837	-1.409548)
207:	3199.53	7.632085	(-0.959635	0.626060	-2.513809)
208:	3202.14	21.220487	(0.058443	1.857730	4.214963)
209:	3204.30	16.016198	(-3.019754	2.500359	0.803423)
210:	3204.62	12.582508	(-0.819307	-0.819925	3.352457)
211:	3206.91	5.642556	(-0.806268	2.131203	-0.671163)
212:	3207.72	1.843900	(-1.218506	-0.586402	0.123596)
213:	3210.32	0.364495	(0.393927	0.422583	-0.175328)
214:	3211.12	1.673569	(0.404143	-0.187428	-1.214540)
215:	3248.68	4.095460	(-1.506154	1.283396	0.424093)

The first frequency considered to be a vibration is 6

The total number of vibrations considered is 210

7. Literature

[1] H. Shen, K. Vollhardt, *Synlett* **2012**, *2012*, 208–214.

[2] J. J. S. Lamba, J. M. Tour, *J. Am. Chem. Soc.* **1994**, *116*, 11723–11736.

[3] P. Karastatiris, J. A. Mikroyannidis, I. K. Spiliopoulos, A. P. Kulkarni, S. A. Jenekhe, *Macromolecules* **2004**, *37*, 7867–7878.

[4] Y. Kubo, M. Ikeda, A. Sugasaki, M. Takeuchi, S. Shinkai, *Tetrahedron Lett.* **2001**, *42*, 7435–7438.

[5] L. M. Bannwart, L. Jundt, T. Müntener, M. Neuburger, D. Häussinger, M. Mayor, *Eur. J. Org. Chem.* **2018**, *2018*, 3391–3402.

[6] O. Trapp, *Chirality* **2006**, *18*, 489–497.

[7] O. Trapp, *J. Chromatogr. B* **2008**, *875*, 42–47.

[8] O. Trapp, V. Schurig, *Chirality* **2002**, *14*, 465–470.

[9] M. Rickhaus, L. Jundt, M. Mayor, *Chim. Int. J. Chem.* **2016**, *70*, 192–202.
